# Direct asymmetric α C(*sp*^*3*^)‒H alkylation of benzylamines with MBH acetates enabled by bifunctional pyridoxal catalysts

**DOI:** 10.1038/s41467-025-65648-5

**Published:** 2025-11-27

**Authors:** Jiayao Chen, Yue Yang, Siqi Liu, Weibo Ling, Ruixin Zhang, Tongyin Chen, Wen-Wen Chen, Baoguo Zhao

**Affiliations:** 1 The Education Ministry Key Lab of Resource Chemistry, Shanghai Frontiers Science Center of Biomimetic Catalysis and College of Chemistry and Materials Science, Shanghai Normal University, Shanghai, China; 2 Frontiers Science Center for Transformative Molecules and The School of Chemistry and Chemical Engineering, Shanghai Jiao Tong University, Shanghai, China

**Keywords:** Asymmetric catalysis, Organocatalysis, Synthetic chemistry methodology

## Abstract

Organocatalytic allylic substitution of Morita-Baylis-Hillman (MBH) adducts is widely regarded as one of the most powerful transformations in organic synthesis. A range of activated carbon nucleophiles have been successfully employed in this reaction, enabling the incorporation of diverse functional moieties. Despite its potential, the use of inert C–H nucleophiles—critical for broadening the reaction’s versatility and synthetic utility—remains a significant challenge in the field. Direct α-C–H functionalization of benzyl amines with MBH adducts offers a promising route to form a new C–C bond while simultaneously establishing a chiral amine moiety, a feature highly attractive from the perspective of organic synthesis. However, this transformation is particularly challenging due to the inherent inertness of the α-C(*sp*³)–H bonds, significant nucleophilic interference from the NH₂ group, and the complexity of selectivity control. Herein, we have successfully achieved an asymmetric direct α-C–H allylic alkylation of NH₂-unprotected benzylamines with MBH adducts using a bifunctional chiral pyridoxal catalyst, producing biologically important chiral γ-amino acid derivatives in good yields with excellent diastereo- and enantioselectivities. The reaction offers a distinct strategy for synthesizing multiply functionalized compounds from readily available starting materials, significantly expanding access to complex chiral architectures.

## Abstract (web summary)

Organocatalytic allylic substitution of Morita-Baylis-Hillman (MBH) adducts constitute powerful transformations within organic synthesis. Here the authors present an asymmetric direct α-C–H allylic alkylation of NH₂-unprotected benzylamines with MBH adducts, using a bifunctional chiral pyridoxal catalyst, producing biologically important chiral γ-amino acid derivatives.

## Introduction

**Fig. 1:**
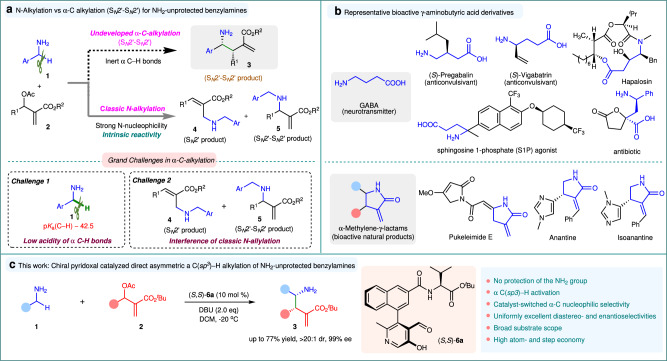
α C‒H allylic alkylation of benzylamines with MBH adducts. **a** N-Alkylation vs α-C alkylation (S_*N*_2’-S_*N*_2’) of NH_2_-unprotected benzylamines. **b** Representative bioactive γ-aminobutyric acid derivatives. **c** This work: chiral pyridoxal catalyzed direct asymmetric α C(*sp*^*3*^)‒H alkylation of NH_2_-unprotected benzylamines with MBH acetates. Ar, aromatic group; Ph, phenyl; S_*N*_2’, bimolecular allylic nucleophilic substitution; DBU, 1,8-diazabicyclo[5.4.0]undec-7-ene; DCM, dichloromethane; dr, diastereomeric ratio; ee, enantiomeric excess.

Organocatalytic allylic substitution of Morita-Baylis-Hillman (MBH) adducts has emerged as a highly versatile synthetic transformation^1–7^, allowing the introduction of a wide diversity of nucleophiles into multifunctional molecular frameworks. While activated carbon-nucleophiles have been widely utilized^8–22^, the use of inert C–H nucleophiles, essential for expanding the reaction’s versatility and synthetic utility, remains a major challenge^23^. Benzylamines are a class of readily accessible primary amines possessing two inert α C(*sp*^*3*^)‒H bonds^24^. Direct asymmetric organocatalytic α-C(*sp*^*3*^)‒H allylic alkylation of benzylamines with MBH adducts offers a promising strategy for simultaneous formation of a C–C bond and establishment of chiral amine functionality (Fig. 1a), providing an efficient and appealing approach to construct chiral substituted γ-aminobutyric acid (GABA) scaffolds that are prevailing core structures of numerous bioactive natural products and pharmaceutical molecules (Fig. 1b)^25–29^. Moreover, through simple cyclization, γ-aminobutyric acids can be easily converted into another type of biologically important γ-lactam analogs (Fig. 1b)^30–32^. In spite of its potential benefits, this transformation remains undeveloped and is a significant challenge in organic chemistry, even when employing NH_2_-protected benzylamine derivatives. The difficulties can be attributed to the following two factors (Fig. 1a). First, deprotonating the α-amino C–H bonds to produce active carbanions for initiating the addition is rather difficult due to the extremely low acidity of α-amino C−H bonds (p*K*_a_ ~ 42.5)^24^. Second, owing to the high nucleophilicity of the NH₂ group, the classical N-substitution^33,34^ usually disrupts the desired α-C alkylation, resulting in the predominant formation of the N-alkylated products **4** and/or **5** (Fig. 1a).

Carbonyl catalysis^35–40^ has become an effective strategy for direct α C‒H functionalization of NH_2_-unprotected primary amines^41–50^. With chiral pyridoxals as the carbonyl catalysts, a series of enantioselective transformations of activated primary amines, such as α-amino esters with diverse electrophiles, have been successfully accomplished, affording diverse chiral amino acid derivatives^41–43^. Nevertheless, direct asymmetric α-functionalization of primary amines containing inert α-amino C–H bonds^24,44,45^, for instance, benzylamines, presents greater difficulties. Till now, for the carbonyl-catalyzed reaction of benzylamines, only the asymmetric addition to aldehydes has been achieved^24^. Allylic alkylation of benzylamines with MBH acetates is more difficult to achieve, primarily due to the strong interference from the highly nucleophilic amino group and the more complicated functional group compatibility of MBH acetates. Herein, we would like to disclose our success on the direct asymmetric α C−H alkylation of benzylamines **1** with MBH acetates **2**, with a switched nucleophilic selectivity from N to α-C of benzylamines **1**, enabled by the chiral bifunctional pyridoxal catalysts **6**^41^ bearing an amide side chain attached to the C3 position of the naphthyl ring. This reaction produced a wide range of chiral polysubstituted γ-amino acid esters **3** with excellent diastereo- and enantioselectivities (up to >20:1 dr, 99% ee) (Fig. 1c).

## Results

### Reaction optimization

**Fig. 2:**
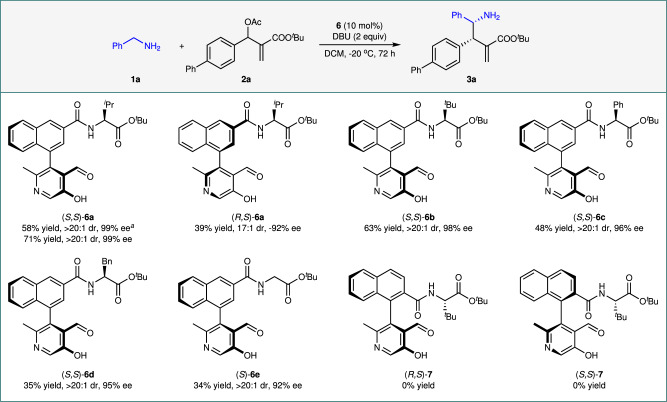
Catalyst screening. Reaction conditions: **1a** (0.20 mmol), **2a** (0.10 mmol), catalyst **6** (0.01 mmol, 10 mol%), and DBU (0.20 mmol) in DCM (0.5 mL) at -20 ^o^C for 72 h. Isolated yields were based on **2a**. The dr values were determined by ^1^H NMR analysis of the crude reaction mixtures. The ee values were determined by chiral HPLC analysis. ^*i*^Pr: isopropyl; ^*t*^Bu: *tert*-butyl; Ph: phenyl; Bn: benzyl; Ac, acetyl; DBU, 1,8-diazabicyclo[5.4.0]undec-7-ene; DCM, dichloromethane. ^*a*^The reaction time was 24 h.

Our studies commenced with the investigation of the direct asymmetric α C−H alkylation of benzylamine (**1a**) with MBH acetate **2a** (Fig. 2, Supplementary Table S1). To our delight, with pyridoxal (*S*,*S*)-**6a** as the catalyst and DBU as the base, the reaction proceeded smoothly for 24 h as anticipated to afford chiral γ-amino acid ester **3a** in a 58% yield with >20:1 diastereomeric ratio (dr) and 99% ee for the major diastereomer accompanied by the formation of some N-alkylated by-products (Fig. 2, Supplementary Table S1, entry 1). The pyridoxal catalyst is crucial for this reaction, as no desired α-C allylic alkylation but only N-alkylation can be observed in its absence (Supplementary Table S1, entries 2 and 3), demonstrating that the pyridoxal catalyst is capable of switching the nucleophilic selectivity of benzylamine **1a** from N to α-C without protecting the NH₂ group. Extending the reaction time to 72 h can effectively improve the yield of the reaction to 71% while maintaining the excellent diastereo- and enantioselectivities ( > 20:1 dr, 99% ee) (Fig. 2, Supplementary Table S1, entry 4). The diastereomeric pyridoxal (*R*,*S*)-**6a** was less effective for the reaction, resulting in the product **3a** with decreased yield and stereoselectivity (Fig. 2). Among the pyridoxals **6a-e** examined, compound (*S*,*S*)-**6a** displayed the best performance regarding activity, diastereo-, and enantioselectivities (Fig. 2). Pyridoxals (*R*,*S*)-**7** and (*S*,*S*)-**7** possessing a lateral amide chain at the C2 position of the naphthyl ring were completely ineffective for the reaction (Fig. 2), indicating the side chain is important for the activity of the pyridoxal catalyst. Reaction condition investigations exhibited that DBU was the base of choice (Supplementary Table S1, entry 1 vs 12-16) and dichloromethane was the optimal solvent (Supplementary Table S1, entry 1 vs 17-20).

### Substrate scope

**Fig. 3:**
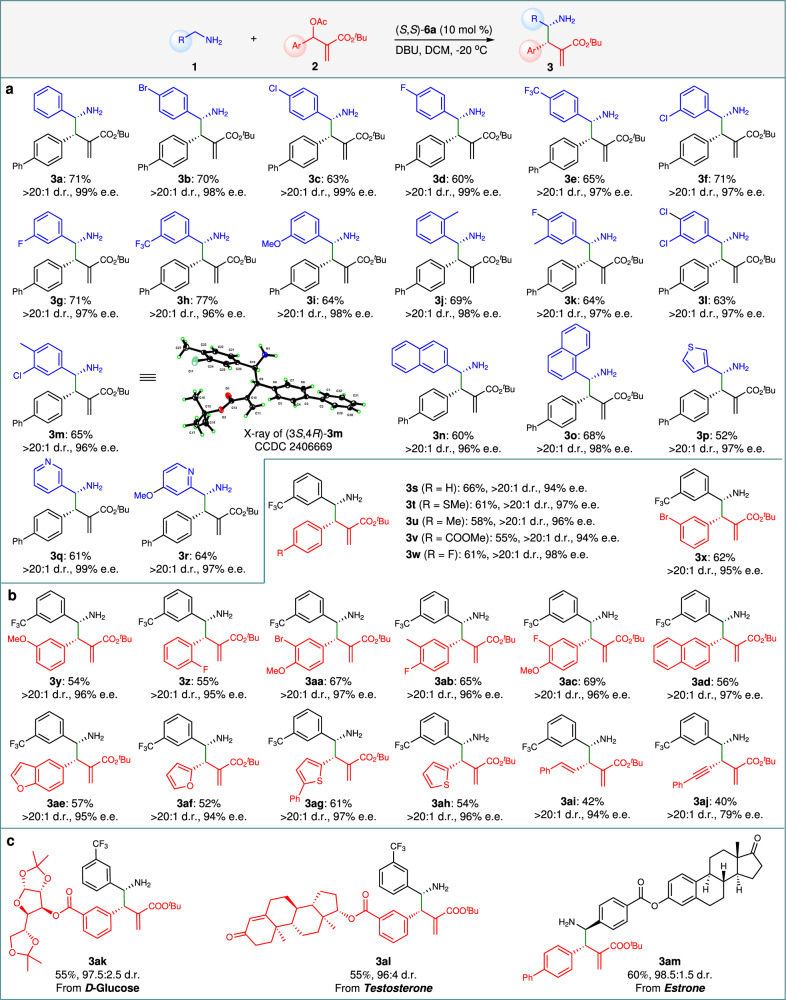
Substrate scope. **a** Investigation on benzylamines. **b** Investigation on MBH acetates. **c** Investigation on chiral substrates. Reaction conditions: **1** (0.40 mmol), **2** (0.20 mmol), (*S*,*S*)-**6a** (0.02 mmol, 10 mol%) and DBU (0.40 mmol) in DCM (1.0 mL) at −20 °C for 72 h. The isolated yields were based on **2**. The dr values were determined by ^1^H NMR analysis of crude reaction mixtures. The ee values were determined by chiral HPLC analysis. The absolute configuration for **3m** was determined as (3*S*,4*R*) by X-ray analysis, and those for **3a**-**3l,** **3n**-**3am** were tentatively assigned by analog. DBU, 1,8-diazabicyclo[5.4.0]undec-7-ene; DCM, dichloromethane.

Under the optimal reaction conditions, substrate scope on benzylamines was investigated (Fig. 3). A variety of benzylamines **1** bearing different electron-donating and/or electron-withdrawing substituents at the *ortho*-, *meta*-, or/and *para*-positions of the benzene ring all smoothly underwent the direct asymmetric α C‒H allylic alkylation with MBH acetate **2a**, producing the corresponding products **3b**-**m** in good yields (60-77%) with excellent diastereo- and enantioselectivities ( > 20:1 dr and 96-99% ee) (Fig. 3a). The absolute configuration of the major diastereomer of product **3m** was determined as (3*S*,4 *R*) by X-ray analysis (Fig. 3a). Delightfully, the reaction is not sensitive to the steric hindrance. The *ortho*-substituted benzylamine, such as *o*-tolylmethanamine (as for **3j**), still displayed good reactivity with high stereoselectivity ( > 20:1 dr, 98% ee). Naphthyl substituted methanamines (as for **3n** and **3o**) as well as heteroarylmethanamines such as thiophen-3-ylmethanamine (as for **3p**), pyridin-3-ylmethanamine (as for **3q**), and (4-methoxypyridin-2-yl)methanamine (as for **3r**) were also applicable for the reaction. The other reaction partner MBH acetates were also examined. Phenyl (as for **3s**), substituted phenyl (as for **3t**–**ad**) and heteroaromatic (as for **3ae**–**ah**) MBH acetates with different electronic properties and substitution patterns on the benzene ring were proven to be effective substrates for the α-C allylic substitution, as they all exhibited good reactivities and led to the formation of products **3s**–**ah** with uniformly high diastereomeric ratios ( > 20:1 dr) and high enantiopurities (94-98% ee) (Fig. 3b). The electronic property of the substituted phenyl groups seems to have little impact on the diastereo- and enantioselectivity. Alkenyl- and alkynyl-substituted MBH acetates are also reactive for the transformation, producing chiral γ-amino acid esters **3ai** and **3aj** with excellent diastereoselectivity (Fig. 3b). The relatively low ee value observed for **3aj** is likely due to the linear geometry of the alkyne group, which lacks sufficient steric hindrance for effective enantiocontrol. Alkyl-substituted MBH acetates, such as *tert*-butyl 3-acetoxy-2-methylene-5-phenylpentanoate, are completely ineffective for the α-C allylic alkylation. Notably, when utilizing substrates containing a biologically active chiral moiety derived from D-glucose (as for **3ak**), testosterone^51^ (as for **3al**), or estrone (as for **3am**), the reaction proceeded fluently to afford products **3ak**-**am** in good yields with excellent diastereocontrol (Fig. 3c).

### Synthetic utility

**Fig. 4:**
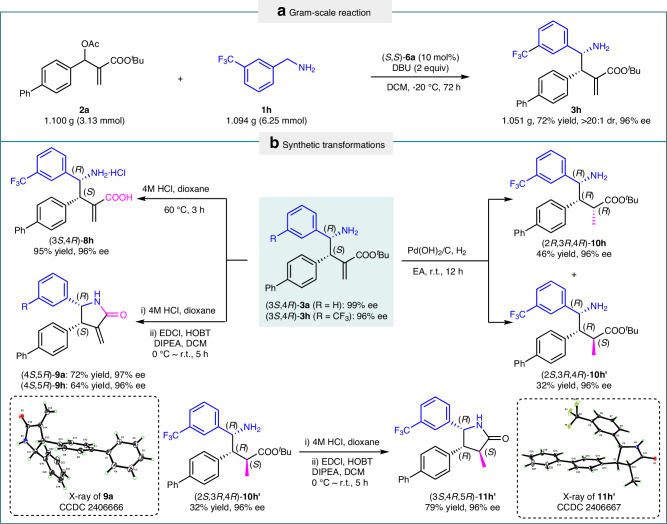
Synthetic applications. **a** Gram-scale reaction. **b** Synthetic transformations. Ac, acetyl; EDCl, 1-(3-dimethylaminopropyl)-3-ethylcarbodiimide hydrochloride; HOBT, 1-hydroxybenzotriazole; DIPEA, N, N-Diisopropylethylamine; EA, ethyl acetate; DBU, 1,8-diazabicyclo[5.4.0]undec-7-ene; DCM, dichloromethane.

To demonstrate the practical utility of the protocol, the reaction was carried out on a gram-scale. Chiral γ-amino acid ester **3h** (1.051 g) was obtained in a comparable yield with the same diastereo- and enantioselectivies (Fig. 4a). The synthetic utility of the product was further investigated. Chiral γ-amino acid esters **3** can be facilely transformed into different derivatives with potential bioactivities (Fig. 4b). Deprotection of the *tert*-butyl moiety of the major diastereomer (3*S*,4*R*)-**3h** by means of HCl produced chiral γ-aminobutyric acid **8h** in 95% yield while maintaining the same enantiopurity. As illustrated in Fig. 4b, cyclic α-methylene-γ-lactam **9a** and **9h** were successfully obtained in satisfactory yields with high enantiopurities via sequential deprotection and condensation. Notably, α-methylene-γ-lactam derivatives have been discovered to exhibit anti-inflammatory, phytotoxic, cytotoxic, and antimicrobial bioactivities^30–32^. Additionally, under Pd-catalyzed hydrogenation conditions, the reduction of **3h** proceeded smoothly to afford a pair of chromatographically separable diastereomers (2*R*,3*R*,4*R*)-**10h** (46% yield) and (2*S*,3*R*,4*R*)-**10h’** (32% yield) without any loss of enantiopurity, as depicted in Fig. 4b. Furthermore, compound **10h’** can be converted into cyclic γ-lactam **11h’** as presented in Fig. 4b. The absolute configuration of compounds **11h’** as well as **9a** were confirmed by X-ray analysis (see Supplementary Information).

### Mechanistic studies

**Fig. 5:**
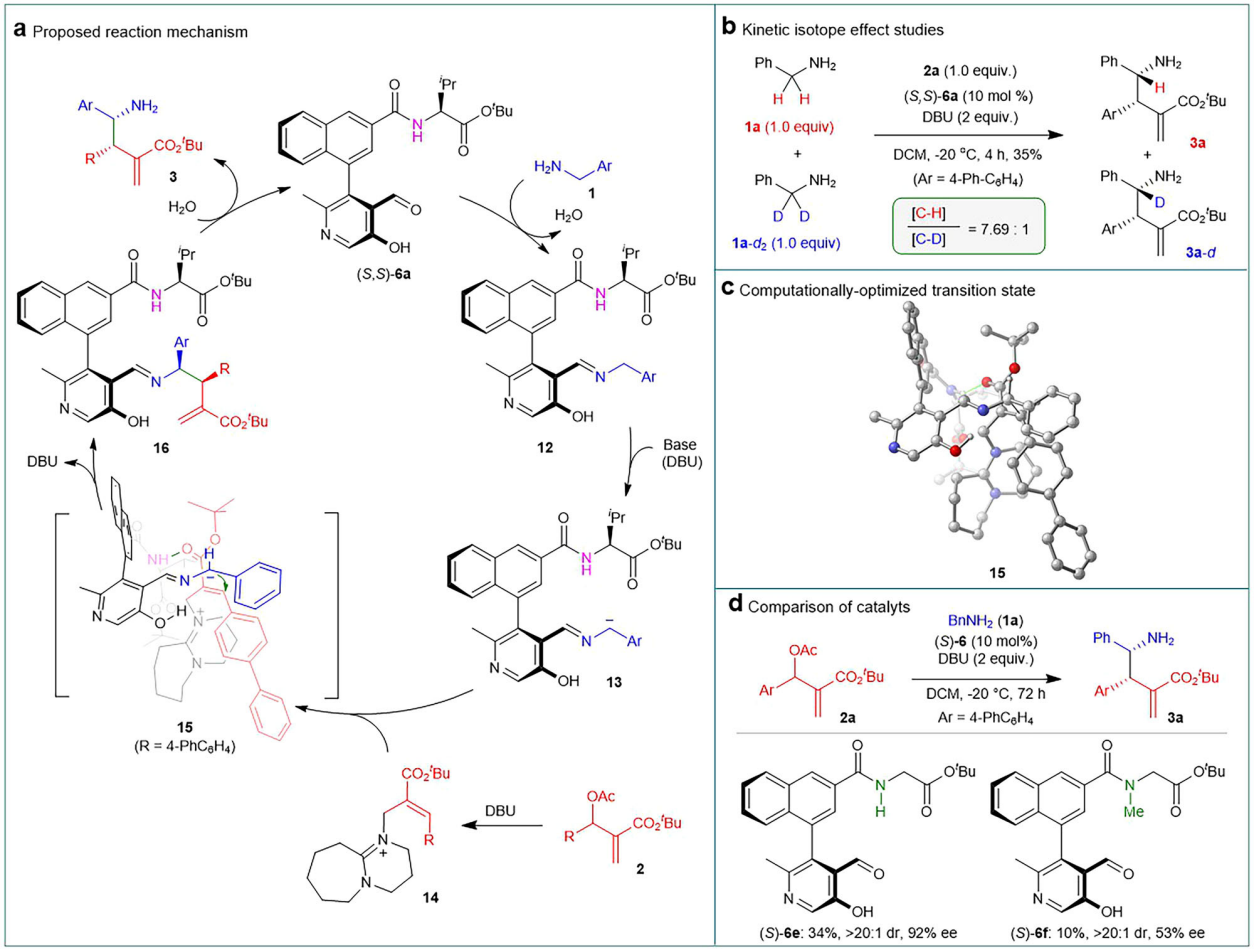
Mechanistic studies. **a** Proposed reaction mechanism. **b** Kinetic isotope effect studies. **c** Computationally-optimized transition state. **d** Comparison of catalysts. DBU, 1,8-diazabicyclo[5.4.0]undec-7-ene; DCM, dichloromethane.

The reaction was proposed to proceed through a carbonyl catalysis mechanistic pathway, in which the chiral pyridoxal catalyst served as a pivotal factor (Fig. 5a)^35–39^. The condensation between chiral pyridoxal catalyst **6a** and arylmethanamine **1** results in the formation of imine **12**, which activates the benzylic C–H bonds and remarkably increases the C–H acidity for further deprotonation to generate delocalized carbanion **13**^52,53^. Computational p*K*_a_ analysis reveals the benzylic C–H of imine **12** is more acidic than the phenolic O–H and N–H of the pyridoxal scaffold, thermodynamically favoring the formation of the carbanion via selective C–H deprotonation (see Supplementary Information for details). The carbanion **13** undergoes an asymmetric addition reaction to quaternary ammonium salt intermediate **14**, which is generated via the nucleophilic addition of DBU to MBH acetate **2**. The addition is accompanied by the expulsion of DBU, leading to the formation of species **16**. Hydrolysis of compound **16** leads to the liberation of the S_*N*_2’-S_*N*_2’ α-C alkylation product **3** and the regeneration of the pyridoxal catalyst **6a**, completing the catalytic cycle.

Kinetic isotope effect (KIE) studies were conducted with equimolar amounts of benzylamine **1a** and α-deuterated counterpart **1a**-***d*** (Fig. 5b). The reaction led to the formation of compound **3a** and the deuterated **3a-*d***, with a ratio of 7.69:1. The distinct kinetic isotope effect strongly suggests that the deprotonation of imine **12** to afford the active carbanion intermediate **13** is the rate-determining step for the entire reaction pathway.

To elucidate the origin of chirality, computational investigations have been conducted. Figure 5c depicts the optimized transition state (**15**) for the step involving the asymmetric addition of carbanion **13** to intermediate **14**. While benzylamine **1** is bound by the catalyst via the formation of the imine with the pyridoxal moiety, the MBH acetate-derived intermediate **14** is activated by the side chain of the pyridoxal catalyst. This activation occurs via a hydrogen-bonding interaction^54–56^ between the NH group of the side chain and the carbonyl group of intermediate **14**. To minimize steric repulsion, intermediate **14** adopts an orientation wherein both the bulky R group and the DBU moiety are directed away from the biaryl backbone of the pyridoxal catalyst. The carbanion originating from arylmethanamines approaches **14** from above to afford chiral γ-amino acid esters **3** with (3*S*,4*R*)-configuration from chiral pyridoxal (*S*,*S*)-**6a**.

The postulated transition state is supported by control experiments on the comparison of catalysts (Fig. 5d). Methylation of the amide N–H group on the lateral side chain of pyridoxal **6a** resulted in a significant decline in activity and enantioselectivity. As shown in Fig. 5d, the reaction with catalyst **6f** afforded a 10% yield, a diastereomeric ratio (dr) of >20:1, and an enantiomeric excess (ee) of 53%, in contrast to the 34% yield, >20:1 dr, and 92% ee obtained with catalyst **6e**. The result implies that the amide N–H group likely participates in the catalytic process via hydrogen bonding, as proposed in transition state **15**. The cooperative bifunctional activation accounts for the excellent performance of pyridoxals **6** bearing a C3 amide side chain in the reaction. This was further confirmed by the fact that pyridoxals **7**, having a C2 amide side chain, are completely inactive for the reaction (Fig. 2). It is supposed that the side chain is too close to the aldehyde moiety to guarantee effective bifunctional activation during the catalysis.

## Discussion

In summary, we have successfully developed a direct asymmetric α C−H allylic alkylation of benzylamines **1** with MBH acetates **2** by utilizing bifunctional chiral pyridoxal (*S*,*S*)-**6a** as the catalyst. This reaction furnishes diverse chiral γ-amino acid esters **3**, which are of biological significance, in 40–77% yields with excellent diastereo- ( > 20:1 dr) and enantioselectivities (79–99% ee). This work exemplifies a remarkable illustration of organocatalyzed inert C–H bond functionalization under mild conditions, offering high stereocontrol without directing or protecting group manipulations. Moreover, it also highlights the distinctive exceptional capabilities of vitamin B_6_^57,58^ based organocatalysts^59–61^ in organic synthesis.

## Methods

### General procedure for asymmetric α C(*sp*^*3*^)‒H allylic alkylation of benzylamines with MBH acetates (Fig. 3)

To a 4 mL vial equipped with a magnetic stirrer bar were successively added chiral pyridoxal (*S*,*S*)-**6a** (0.0046 g, 0.010 mmol), DBU (0.0304 g, 0.20 mmol), DCM (0.3 mL) and benzylamine **1** (0.20 mmol). The mixture was stirred at −20 °C for 5 min, and a solution of MBH acetate **2** (0.10 mmol) in DCM (0.2 mL) was added in portions over 1 h. After the reaction mixture was stirred at −20 °C for 72 h, it was allowed to warm up to room temperature and concentrated via rotary evaporator to remove most of the solvent. Then it was dried under vacuum and submitted to ^1^H NMR analysis to determine the dr values. The product **3** was purified by column chromatography on silica gel (petroleum ether: ethyl acetate = 3:1). The dr values of products **3a**-**ak** were determined by ^1^H NMR analysis of the crude reaction mixtures. The enantiomeric excesses (ee’s) of products **3a**-**ak** were determined by chiral HPLC analysis.

### Procedure for synthesis of γ-amino acid ester 3 h in gram-scale (Fig. 3a)

To a 25 mL flask equipped with a magnetic stirrer bar were successively added chiral pyridoxal (*S*,*S*)-**6a** (0.144 g, 0.312 mmol), DBU (0.950 g, 6.250 mmol), DCM (9.35 mL) and arylmethanamine **1h** (1.094 g, 6.250 mmol). The mixture was stirred at −20 °C for 5 min, and a solution of MBH acetate **2a** (1.100 g, 3.130 mmol) in DCM (6.25 mL) was added in portions over 1 h. After the reaction mixture was stirred at −20 °C for 72 h, it was allowed to warm up to room temperature and concentrated via rotary evaporator to remove most of the solvent. Then it was dried under vacuum and submitted to ^1^H NMR analysis to determine the dr values. The crude reaction mixture was purified by column chromatography on silica gel (petroleum ether: ethyl acetate = 3:1) to afford compound (*S,R*)**-3h** (1.051 g, 72% yield, >20:1 dr, 96% ee) as a white solid.

## Supplementary information

Supplementary information

Description of Additional Supplementary Files

Supplementary data 1

Transparent Peer Review file

## Data Availability

The authors declare that the data supporting the findings of this study are available within the article and Supplementary Information file, or from the corresponding author upon request. The X-ray crystallographic coordinates for structures reported in this study have been deposited at the Cambridge Crystallographic Data Center (CCDC), under deposition numbers of CCDC 2406669 [(3*S*,4*R*)-**3m** in Supplementary Fig. S8], CCDC 2406666 [(4*S*,5*R*)-**9a** in Supplementary Fig. S9] and CCDC 2406667 [(3*S*,4*R*,5*R*)-**11h’** in Supplementary Fig. S10]. These data can be obtained free of charge from The Cambridge Crystallographic Data Center via https://www.ccdc.cam.ac.uk/structures/. Coordinates of the optimized structures are available from the Supplementary Data 1.

## References

[1] Rios, R. Organocatalytic enantioselective methodologies using Morita–Baylis–Hillman carbonates and acetates. *Catal. Sci. Technol.* **2**, 267–278 (2012).

[2] Liu, T.-Y., Xie, M. & Chen, Y.-C. Organocatalytic asymmetric transformations of modified Morita–Baylis–Hillman adducts. *Chem. Soc. Rev.* **41**, 4101–4112 (2012). 22453359 10.1039/c2cs35017c

[3] Wei, Y. & Shi, M. Recent advances in organocatalytic asymmetric Morita–Baylis–Hillman/aza-Morita–Baylis–Hillman reactions. *Chem. Rev.* **113**, 6659–6690 (2013). 23679920 10.1021/cr300192h

[4] Wang, T., Han, X., Zhong, F., Yao, W. & Lu, Y. Amino acid-derived bifunctional phosphines for enantioselective transformations. *Acc. Chem. Res.* **49**, 1369–1378 (2016). 27310293 10.1021/acs.accounts.6b00163

[5] Guo, H., Fan, Y. C., Sun, Z., Wu, Y. & Kwon, O. Phosphine organocatalysis. *Chem. Rev.* **118**, 10049–10293 (2018). 30260217 10.1021/acs.chemrev.8b00081 PMC6218176

[6] Pellissier, H. Asymmetric organocatalytic Morita−Baylis−Hillman reaction and asymmetric organocatalytic transformations of Morita−Baylis−Hillman adducts. An update. *Tetrahedron* **172**, 134435 (2025).

[7] Duan, X.-H., Du, H.-R. & Song, Y.-X. Recent advances in catalytic asymmetric reactions of Morita–Baylis–Hillman adducts. *Org. Chem. Front*. 10.1039/d4qo02212b (2025).

[8] Cho, C.-W. & Krische, M. J. Regio- and stereoselective construction of γ-butenolides through phosphine-catalyzed substitution of Morita–Baylis–Hillman acetates: An organocatalytic allylic alkylation. *Angew. Chem. Int. Ed.* **43**, 6689–6691 (2004). 10.1002/anie.200461381 15593162

[9] Ramachandran, P. V., Madhi, S., Bland-Berry, L., Ram Reddy, M. V. & O’Donnell, M. J. Catalytic enantioselective synthesis of glutamic acid derivatives via tandem conjugate addition−elimination of activated allylic acetates under chiral PTC conditions. *J. Am. Chem. Soc.* **127**, 13450–13451 (2005). 16190680 10.1021/ja052638m

[10] Jiang, Y.-Q., Shi, Y.-L. & Shi, M. Chiral phosphine-catalyzed enantioselective construction of γ-butenolides through substitution of Morita−Baylis−Hillman acetates with 2-trimethylsilyloxy furan. *J. Am. Chem. Soc.* **130**, 7202–7203 (2008). 18479136 10.1021/ja802422d

[11] Wang, L., Prabhudas, B. & Clive, D. L. J. Formation of carbocycles by intramolecular conjugate displacement: scope and mechanistic insights. *J. Am. Chem. Soc.* **131**, 6003–6012 (2009). 19348481 10.1021/ja900857h

[12] Zhang, Q. & Yang, L. & Tong, X. 2-(Acetoxymethyl)buta-2,3-dienoate, a versatile 1,4-biselectrophile for phosphine-catalyzed (4 + n) annulations with 1,n-bisnucleophiles (n = 1, 2). *J*. *Am. Chem. Soc.* **132**, 2550–2551 (2010). 10.1021/ja100432m 20131904

[13] Peng, J., Huang, X., Cui, H.-L. & Chen, Y.-C. Organocatalytic and electrophilic approach to oxindoles with C3-quaternary stereocenters. *Org. Lett.* **12**, 4260–4263 (2010). 20812696 10.1021/ol101668z

[14] Furukawa, T. et al. Asymmetric allylic monofluoromethylation and methylation of Morita–Baylis–Hillman carbonates with FBSM and BSM by cooperative cinchona alkaloid/FeCl_2_ catalysis. *Angew. Chem. Int. Ed.* **50**, 9684–9688 (2011). 10.1002/anie.201103748 21882315

[15] Liu, C. et al. Chiral biscinchona alkaloid promoted asymmetric allylic alkylation of 3-substituted benzofuran-2(3*H*)-ones with Morita–Baylis–Hillman carbonates. *J. Org. Chem.* **76**, 5838–5845 (2011). 21644582 10.1021/jo200557b

[16] Zhong, F., Luo, J., Chen, G.-Y., Dou, X. & Lu, Y. Highly enantioselective regiodivergent allylic alkylations of MBH carbonates with phthalides. *J. Am. Chem. Soc.* **134**, 10222–10227 (2012). 22621622 10.1021/ja303115m

[17] Yang, W. et al. Direct asymmetric allylic alkenylation of N-itaconimides with Morita–Baylis–Hillman carbonates. *J. Org. Chem.* **77**, 6600–6607 (2012). 22816444 10.1021/jo3012539

[18] Mao, H. et al. Construction of enantiomerically enriched diazo compounds using diazo esters as nucleophiles: chiral Lewis base catalysis. *Angew. Chem. Int. Ed.* **52**, 6288–6292 (2013). 10.1002/anie.201301509 23629818

[19] Han, X. et al. Asymmetric synthesis of spiropyrazolones through phosphine-catalyzed [4+1] annulation. *Angew. Chem. Int. Ed.* **53**, 5643–5647 (2014). 10.1002/anie.201311214 24737675

[20] Zhang, L. et al. Phosphine-catalyzed highly enantioselective [3 + 3] cycloaddition of Morita–Baylis–Hillman carbonates with C,N-cyclic azomethine imines. *J. Am. Chem. Soc.* **137**, 4316–4319 (2015). 25799312 10.1021/jacs.5b01138

[21] Chen, P. et al. Phosphine-catalyzed asymmetric umpolung addition of trifluoromethyl ketimines to Morita–Baylis–Hillman carbonates. *Angew. Chem. Int. Ed.* **55**, 13316–13320 (2016). 10.1002/anie.201607918 27634055

[22] Ni, C. & Tong, X. Amine-catalyzed asymmetric (3 + 3) annulations of β′-acetoxy allenoates: enantioselective synthesis of 4*H*-pyrans. *J. Am. Chem. Soc.* **138**, 7872–7875 (2016). 27310820 10.1021/jacs.6b04935

[23] Hu, Y. et al. Organocatalytic asymmetric C(sp^2^)−H allylic alkylation: enantioselective synthesis of tetrasubstituted allenoates. *Angew. Chem. Int. Ed.* **59**, 19820–19824 (2020). 10.1002/anie.202009460 32820579

[24] Hou, C. et al. Catalytic asymmetric α C(*sp*^3^)–H addition of benzylamines to aldehydes. *Nat. Catal.* **5**, 1061–1068 (2022).

[25] Nascimento, A. A., Pereira-Figueiredo, D., Borges-Martins, V. P., Kubrusly, R. C. & Calaza, K. C. GABAergic system and chloride cotransporters as potential therapeutic targets to mitigate cell death in ischemia. *J. Neurosci. Res.* **102**, e25355 (2024). 38808645 10.1002/jnr.25355

[26] Laribi, M., Chaouali, N., Jaballah, S., Amira, D. & Hedhili, A. Drug abuse of pregabalin-state of the situation, risks and means of control. *Ann. Pharm. Fr.* **81**, 419–424 (2023). 36375531 10.1016/j.pharma.2022.11.005

[27] Stratmann, K., Burgoyne, D. L., Moore, R. E., Patterson, G. M. L. & Smith, C. D. Hapalosin, a cyanobacterial cyclic depsipeptide with multidrug-resistance reversing activity. *J. Org. Chem.* **59**, 7219–7226 (1994).

[28] Guckian, K. M. et al. Bicyclic aryl sphingosine 1-phosphate analogs, WO patent 2,010,051,030 A1 (2010).

[29] Urquilla, A., Merrer, D. C., Sumner, R. & Denton, R. W. Synthesis and biological activity of 2-(2-amino-2-phenylethyl)-5-oxotetrahydrofuran-2-carboxylic acid: a microwave-assisted 1,3-dipolar cycloaddition approach. *Synlett* **32**, 1735–1740 (2021).

[30] Konaklieva, M. I. & Plotkin, B. J. Bioisosters of β-lactams as anti-infectives. *Curr. Med. Chem. - Anti-Infect. Agents* **2**, 287–302 (2003).

[31] Cardellina, J. H. & Moore, R. E. The structures of pukeleimides A, B, D, E, F, and G. *Tetrahedron Lett.* **20**, 2007–2010 (1979).

[32] Tchissambou, L., Bénéchie, M. & Khuong-Huu, F. Alcaloïdes imidazoliques IV. Synthèse de la DL-isoanantine et de la DL-anantine. *Tetrahedron Lett.* **19**, 1801–1802 (1978).

[33] Li, D., Li, J. & Jia, X. Regio- and stereoselective synthesis of α,β-dehydro-β-amino esters. *J. Chem. Res.* **2008**, 434–436 (2008).

[34] Ghosh, S., Dey, R., Chattopadhyay, K. & Ranu, B. C. Water-promoted highly regio- and stereoselective synthesis of α-dehydro-β-amino esters and nitriles from Baylis–Hillman acetates. *Tetrahedron Lett.* **50**, 4892–4895 (2009).

[35] Chen, J. et al. Carbonyl catalysis enables a biomimetic asymmetric Mannich reaction. *Science* **360**, 1438–1442 (2018). 29954974 10.1126/science.aat4210

[36] Li, S., Chen, X.-Y. & Enders, D. Aldehyde catalysis: new options for asymmetric organocatalytic reactions. *Chem* **4**, 2026–2028 (2018).

[37] Wang, Q., Gu, Q. & You, S.-L. Enantioselective carbonyl catalysis enabled by chiral aldehydes. *Angew. Chem. Int. Ed.* **58**, 6818–6825 (2019). 10.1002/anie.201808700 30216640

[38] Xiao, X. et al. Biomimetic asymmetric catalysis. *Sci. China Chem.* **66**, 1553–1633 (2023).

[39] Xiao, X. & Zhao, B. Vitamin B_6_-based biomimetic asymmetric catalysis. *Acc. Chem. Res.* **56**, 1097–1117 (2023). 37071776 10.1021/acs.accounts.3c00053

[40] Wen, W. & Guo, Q.-X. Chiral aldehyde catalysis-enabled asymmetric α-functionalization of activated primary amines. *Acc. Chem. Res.* **57**, 776–794 (2024). 38381559 10.1021/acs.accounts.3c00804

[41] Ma, J. et al. Asymmetric α-allylation of glycinate with switched chemoselectivity enabled by customized bifunctional pyridoxal catalysts. *Angew. Chem. Int. Ed.* **134**, e202200850 (2022). 10.1002/anie.202200850 35182094

[42] Ma, J. et al. Enantioselective synthesis of pyroglutamic acid esters from glycinate via carbonyl catalysis. *Angew. Chem. Int. Ed.* **60**, 10588–10592 (2021). 10.1002/anie.202017306 33554429

[43] Cheng, A. et al. Efficient asymmetric biomimetic aldol reaction of glycinates and trifluoromethyl ketones by carbonyl catalysis. *Angew. Chem. Int. Ed.* **60**, 20166–20172 (2021). 10.1002/anie.202104031 34139067

[44] Ji, P. et al. Direct asymmetric α-C−H addition of N-unprotected propargylic amines to trifluoromethyl ketones by carbonyl catalysis. *Angew. Chem. Int. Ed.* **61**, e202206111 (2022). 10.1002/anie.202206111 36210342

[45] Zhang, R. et al. Direct enantioselective α-C–H conjugate addition of propargylamines to α,β-unsaturated ketones via carbonyl catalysis. *J. Am. Chem. Soc.* **146**, 25927–25933 (2024). 39259771 10.1021/jacs.4c09840

[46] Xu, B. et al. Catalytic asymmetric direct α-alkylation of amino esters by aldehydes via imine activation. *Chem. Sci.* **5**, 1988–1991 (2014).

[47] Wen, W. et al. Chiral aldehyde catalysis for the catalytic asymmetric activation of glycine esters. *J. Am. Chem. Soc.* **140**, 9774–9780 (2018). 29995401 10.1021/jacs.8b06676

[48] Chen, L., Luo, M.-J., Zhu, F., Wen, W. & Guo, Q.-X. Combining chiral aldehyde catalysis and transition-metal catalysis for enantioselective α-allylic alkylation of amino acid esters. *J. Am. Chem. Soc.* **141**, 5159–5163 (2019). 30896937 10.1021/jacs.9b01910

[49] Liu, J.-H. et al. Catalytic asymmetric Tsuji–Trost α−benzylation reaction of N-unprotected amino acids and benzyl alcohol derivatives. *Nat. Commun.* **13**, 2509 (2022). 35523802 10.1038/s41467-022-30277-9 PMC9076619

[50] Zhu, F. et al. Chiral aldehyde/palladium catalysis enables asymmetric branched-selective ring-opening functionalization of methylenecyclopropanes with amino acid esters. *J. Am. Chem. Soc.* **147**, 2315–2322 (2025). 39791232 10.1021/jacs.4c16934

[51] Luberti, F. R. & Carré, J. M. Testosterone’s role in modulating human behaviors relevant to mating and parenting. *Front. Neuroendocr.* **72**, 101112 (2024). 10.1016/j.yfrne.2023.101112 37972861

[52] Crugeiras, J., Rios, A., Riveiros, E. & Richard, J. P. Substituent effects on electrophilic catalysis by the carbonyl group: anatomy of the rate acceleration for PLP-catalyzed deprotonation of glycine. *J. Am. Chem. Soc.* **133**, 3173–3183 (2011). 21323335 10.1021/ja110795m PMC3060797

[53] Tang, S., Zhang, X., Sun, J., Niu, D. & Chruma, J. J. 2-Azaallyl anions, 2-azaallyl cations, 2-azaallyl radicals, and azomethine ylides. *Chem. Rev.* **118**, 10393–10457 (2018). 30302999 10.1021/acs.chemrev.8b00349

[54] Taylor, M. S. & Jacobsen, E. N. Asymmetric catalysis by chiral hydrogen-bond donors. *Angew. Chem. Int. Ed.* **45**, 1520–1543 (2006). 10.1002/anie.200503132 16491487

[55] Doyle, A. G. & Jacobsen, E. N. Small-molecule H-bond donors in asymmetric catalysis. *Chem. Rev.* **107**, 5713–5743 (2007). 18072808 10.1021/cr068373r

[56] Lu, L.-Q., An, X.-L., Chen, J.-R. & Xiao, W.-J. Dual activation in organocatalysis: design of tunable and bifunctional organocatalysts and their applications in enantioselective reactions. *Synlett* **2012**, 490–508 (2012).

[57] Breslow, R. Biomimetic chemistry and artificial enzymes: catalysis by design. *Acc. Chem. Res.* **28**, 146–153 (1995).

[58] Chen, J., Liu, Y. E., Gong, X., Shi, L. & Zhao, B. Biomimetic chiral pyridoxal and pyridoxamine catalysts. *Chin. J. Chem.* **37**, 103–112 (2019).

[59] Dalko, P. I. & Moisan, L. Enantioselective organocatalysis. *Angew. Chem. Int. Ed.* **40**, 3726–3748 (2001). 10.1002/1521-3773(20011015)40:20<3726::aid-anie3726>3.0.co;2-d 11668532

[60] List, B. Introduction: organocatalysis. *Chem. Rev.* **107**, 5413–5415 (2007).

[61] MacMillan, D. W. C. The advent and development of organocatalysis. *Nature* **455**, 304–308 (2008). 18800128 10.1038/nature07367

