# Supplementary material for: Direct asymmetric α C(sp3)‒H alkylation of benzylamines with MBH acetates enabled by bifunctional pyridoxal catalysts
Source: Nat Commun. 2025 Nov 27;16:10645. doi: 10.1038/s41467-025-65648-5 (PMC12660838; doi:10.1038/s41467-025-65648-5)
Supplement: Supplementary file 1 — Supplementary information [file 41467_2025_65648_MOESM1_ESM.pdf]

SUPPLEMENTARY INFORMATION

Direct Asymmetric α C(sp^3)–H Alkylation of Benzylamines with MBH Acetates Enabled by Bifunctional Pyridoxal Catalysts

Jiayao Chen,[1,†] Yue Yang,[1,2,†] Siqi Liu,[1] Weibo Ling,[1] Ruixin Zhang,[1] Tongyin Chen,[1]

Wen-Wen Chen[1,]* and Baoguo Zhao[1,2,]*

[1]The Education Ministry Key Lab of Resource Chemistry, Shanghai Frontiers Science Center of Biomimetic Catalysis and College of Chemistry and Materials Science, Shanghai Normal University; Shanghai 200234, China.

[2]Frontiers Science Center for Transformative Molecules and The School of Chemistry and Chemical Engineering, Shanghai Jiao Tong University, Shanghai 200240, China.

[†]These authors contributed equally: Jiayao Chen and Yue Yang.

Email: wenwen@shnu.edu.cn (W.C.); zhaobg2006@shnu.edu.cn (B.Z.)

Table of Contents

1. General Methods S-2

2. Optimization of Conditions S-3

3. Synthesis of Chiral Pyridoxal Catalysts S-4

4. Synthesis of MBH Acetates **2** S-9

5. Synthesis of Benzylamine **1am** S-17

6. Procedure for Asymmetric α-C Alkylation S-19

7. Synthetic Transformations S-37

8. Kinetic Isotope Effect Studies S-41

9. Experiments on Catalyst Comparison S-43

10. Computational Studies S-44

11. Determination of the Absolute Configuration of **3m**, **9a** and **11h'** S-118

12. ^{1}H NMR, ^{13}C NMR and ^{19}F NMR Spectra S-124

13. Chromatograms for Determination of Enantiomeric Excesses S-255

14. References S-299

1. General Methods

All commercially available reagents were used without further purification unless otherwise stated. Dry THF was obtained by fresh distillation from sodium benzophenone under argon atmosphere. CH_2Cl_2 was freshly distilled from CaH_2. Column chromatography was performed on silica gel (200-300 mesh). ^{1}H NMR spectra were recorded on a 400 NMR spectrometer, ^{13}C NMR spectra were recorded on a 100 MHz NMR spectrometer and ^{19}F NMR spectra were recorded on a 376 MHz NMR spectrometer. ^{1}H shifts were referenced to $CDCl_3$ at 7.26 ppm, ^{13}C shifts were referenced to $CDCl_3$ at 77.16 ppm, MeOD at 49.00 ppm. Melting points were uncorrected. α-Deuterated benzylamine was commercially purchased. Chiral pyridoxals (*R*,*S*)-**7** and (*S*,*S*)-**7** were prepared by following the previously reported procedure[1].

2. Optimization of Conditions

Table S1. Optimization of the reaction conditions[a]

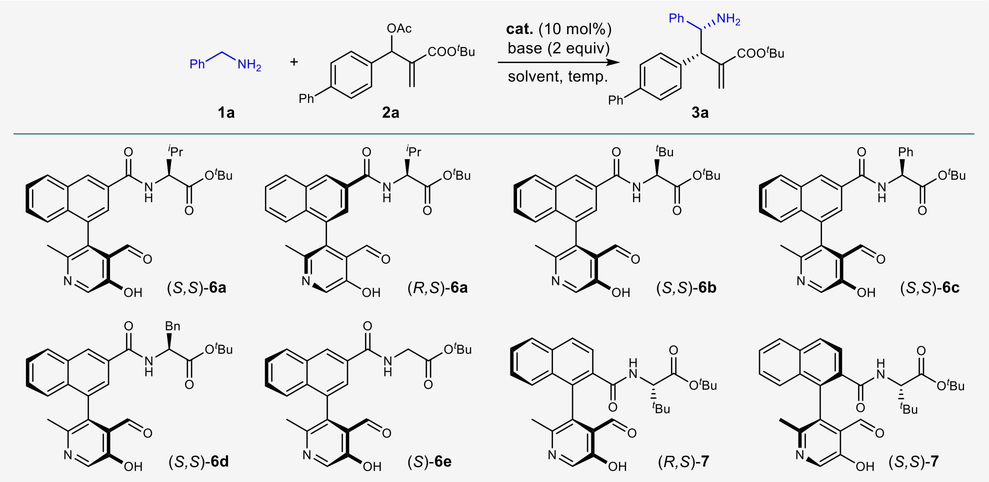

entry	cat.	base	solvent	time (h)	yield (%)	dr	ee (%)
1	(*S*,*S*)-**6a**	DBU	DCM	24	58	>20:1	99
2[b]	–	DBU	DCM	24	0	–	–
3[c]	–	–	DCM	24	0	–	–
4	(*S*,*S*)-**6a**	DBU	DCM	72	71	>20:1	99
5	(*R*,*S*)-**6a**	DBU	DCM	72	39	17:1	-92
6	(*S*,*S*)-**6b**	DBU	DCM	72	63	>20:1	98
7	(*S*,*S*)-**6c**	DBU	DCM	72	48	>20:1	96
8	(*S*,*S*)-**6d**	DBU	DCM	72	35	>20:1	95
9	(*S*)-**6e**	DBU	DCM	72	34	>20:1	92
10	(*R*,*S*)-**7**	DBU	DCM	72	0	–	–
11	(*S*,*S*)-**7**	DBU	DCM	72	0	–	–
12	(*S*,*S*)-**6a**	DBN	DCM	24	45	>20:1	98
13	(*S*,*S*)-**6a**	DMAP	DCM	24	0	–	–
14	(*S*,*S*)-**6a**	BTMG	DCM	24	trace	–	–
15	(*S*,*S*)-**6a**	MTBD	DCM	24	25	>20:1	98
16	(*S*,*S*)-**6a**	Et_3N	DCM	24	0	–	–
17	(*S*,*S*)-**6a**	DBU	$CHCl_3$	24	55	>20:1	96
18	(*S*,*S*)-**6a**	DBU	DCE	24	45	>20:1	97
19	(*S*,*S*)-**6a**	DBU	THF	24	20	>20:1	95
20	(*S*,*S*)-**6a**	DBU	MeCN	24	21	>20:1	93

[a]All reactions were carried out with **1a** (0.20 mmol), **2a** (0.10 mmol), catalyst **6** or **7** (0.010 mmol), and base (0.20 mmol) in solvent (0.50 mL) unless otherwise stated. Isolated yields were based on **2a**. The dr values were determined by ^{1}H NMR analysis of the crude reaction mixtures. The ee values were determined by chiral HPLC (high performance liquid chromatography) analysis. ^{i}Pr: isopropyl; ^{t}Bu: *tert*-butyl; Ph: phenyl; Bn: benzyl; DMAP: 4-dimethylaminopyridine; DBU: 1,8-diazabicyclo[5.4.0]undec-7-ene; DBN: 1,5-Diazabicyclo[4.3.0]non-5-ene; BTMG: 2-*tert*-butyl-1,1,3,3-tetramethyl-guanidine; MTBD: 7-Methyl-1,5,7-triazabicyclo[4.4.0]dec-5-ene; DCM: dichloromethane; DCE: dichloroethane; THF: tetrahydrofuran. [b]The corresponding N-allylation products **4** (24%) and **5** (31%) were formed. [c]The corresponding N-allylation product **4** was formed in 28% yield and the substrate **2a** was recovered in 62% yield.

3. Synthesis of Chiral Pyridoxal Catalysts

3.1 Procedure for the Synthesis of Chiral Pyridoxal Catalysts 6a-f with Compound 6a as the Representative Example[1]

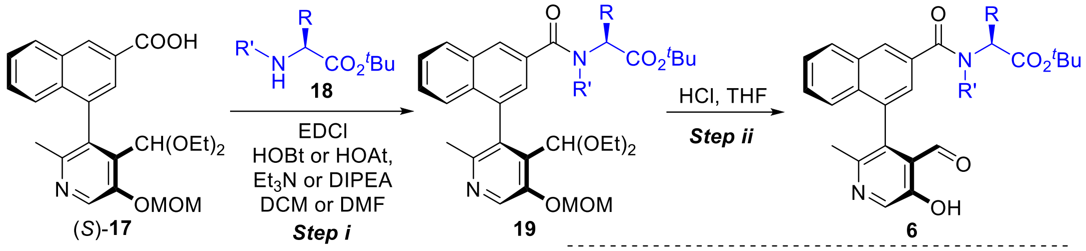

(*S*,*S*)-**6a**, R = ^{i}Pr, R' = H
(*R*,*S*)-**6a**, R = ^{i}Pr, R' = H
(*S*,*S*)-**6b**, R = ^{t}Bu, R' = H
(*S*,*S*)-**6c**, R = Ph, R' = H
(*S*,*S*)-**6d**, R = Bn, R' = H
(*S*)-**6e**, R = H, R' = H
(*S*)-**6f**, R = H, R' = Me

Step i. Synthesis of compound (*S*)-19a (R^1 = H, R^2 = ^{i}Pr)

To a 10 mL reaction flask were added compound (*S*)-**17**[1] (0.2000 g, 0.471 mmol), (*S*)-*tert*-butyl 2-amino-3-methylbutanoate (0.0936 g, 0.541 mmol), 1-(3-dimethylaminopropyl)-3-ethylcarbodiimide hydrochloride (EDCl) (0.1033 g, 0.541 mmol), 1-hydroxybenzotriazole (HOBt) (0.0730 g, 0.541 mmol), and dichloromethane (DCM) (5 mL), followed by addition of triethylamine (Et_3N) (0.1665 g, 1.649 mmol). After stirring at room temperature 24 h, then quenched by addition of water (5 mL). The resulting mixture was extracted with DCM (5 mL × 3). The combined organic layers were washed with brine, dried over Na_2SO_4, filtered, concentrated, and purified by column chromatography on silica gel (petroleum ether : ethyl acetate = 2:1) to give compound (*S*,*S*)-**19a** (0.2250 g, 82%) as a white solid.

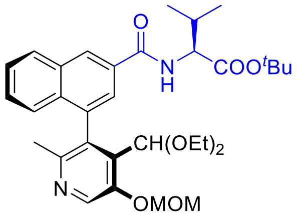

(*S*,*S*)-**19a**

(*S*,*S*)-**19a**: White solid; M.p. 126-129 °C; ^{1}H NMR (400 MHz, $CDCl_3$) δ 8.53 (s, 1H), 8.43 (s, 1H), 8.01 (d, *J* = 8.0 Hz, 1H), 7.70 (d, *J* = 2.0 Hz, 1H), 7.55 (t, *J* = 7.2 Hz, 1H), 7.47 (t, *J* = 7.6 Hz, 1H), 7.35 (d, *J* = 8.4 Hz, 1H), 6.86 (d, *J* = 8.4 Hz, 1H), 5.32 (s, 2H), 5.06 (s, 1H), 4.75 (dd, *J* = 8.8, 4.4 Hz, 1H), 3.58 (s, 3H), 3.38-3.29 (m, 2H), 3.16-2.99 (m, 2H), 2.36-2.26 (m, 1H), 2.06 (s, 3H), 1.50 (s, 9H), 1.03 (d, *J* = 6.8 Hz, 3H), 1.00 (d, *J* = 6.8 Hz, 3H), 0.90 (t, *J* = 6.8 Hz, 3H), 0.75 (t, *J* = 6.8 Hz, 3H); ^{13}C NMR (100 MHz,

CDCl3) δ 171.6, 167.0, 151.6, 149.8, 138.3, 136.4, 135.9, 133.7, 133.3, 132.8, 130.9, 129.6, 128.14, 128.08, 127.0, 125.9, 124.9, 100.3, 95.8, 82.5, 64.0, 63.7, 57.9, 56.5, 32.0, 28.2, 22.8, 19.1, 18.0, 15.0, 14.8; HRMS *m/z* Calcd. for $C_{33}H_{45}N_2O_7$ $(M+H^+)$: 581.3221; Found: 581.3222.

***Step ii.* Synthesis of compound (*S*,*S*)-6a**[1]

To a 25 mL round-bottom flask were added compound (*S*,*S*)-**19a** (0.225 g, 0.388 mmol), HCl aqueous solution (1.0 M, 4 mL), and tetrahydrofuran (THF) (4 mL). The mixture was stirred at 50 °C for 12 h and then cooled to room temperature. The reaction was quenched by addition of saturated aqueous $NaHCO_3$ solution till pH ~ 8, and extracted with DCM (10 mL × 3). The organic layers were combined, dried over anhydrous Na_2SO_4, filtered, concentrated via rotary evaporation, and purified by column chromatography on silica gel (DCM : MeOH = 30:1) to give compound (*S*,*S*)-**6a** (0.162 g, 90 %) as a yellow solid.

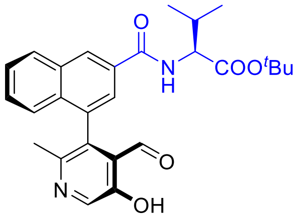

(*S*,*S*)-**6a**

(*S*,*S*)-**6a**: Yellow solid; ^{1}H NMR (400 MHz, $CDCl_3$) δ 10.89 (s, 1H), 9.36 (s, 1H), 8.58 (s, 1H), 8.42 (s, 1H), 8.06 (d, *J* = 8.0 Hz, 1H), 7.83 (s, 1H), 7.62 (t, *J* = 7.6 Hz, 1H), 7.58-7.51 (m, 1H), 7.34 (d, *J* = 8.4 Hz, 1H), 6.92 (s, 1H), 4.75 (dd, *J* = 8.4, 4.4 Hz, 1H), 2.40-2.26 (m, 1H), 2.13 (s, 3H), 1.51 (s, 9H), 1.05 (d, *J* = 6.8 Hz, 3H), 1.04 (d, *J* = 7.2 Hz, 3H); ^{13}C NMR (100 MHz, $CDCl_3$) δ 197.6, 171.6, 166.5, 153.2, 148.8, 141.7, 133.8, 133.7, 133.0, 132.9, 131.3, 129.9, 129.4, 128.4, 127.8, 126.2, 125.4, 122.9, 82.7, 58.0, 32.0, 28.3, 21.9, 19.1, 18.1.

Compounds (*R*,*S*)-**6a**, (*S*,*S*)-**6b-d** and (*S*)-**6e-f** were prepared, respectively in 65%, 76%, 65%, 61%, 55% and 48% yields in two steps by following a similar procedure. Compound (*R*,*S*)-**7** and (*S*,*S*)-**7** were synthesized according to the literature.[1]

Compound (*R*,*S*)-6a

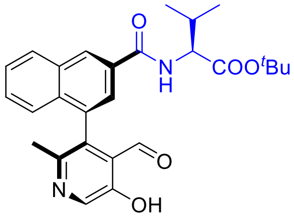

(*R*,*S*)-**6a**

Yellow solid; M.p. 64-68 °C; ^{1}H NMR (400 MHz, $CDCl_3$) δ 10.89 (s, 1H), 9.39 (s, 1H), 8.60 (s, 1H), 8.42 (s, 1H), 8.06 (d, *J* = 8.0 Hz, 1H), 7.84 (d, *J* = 1.6 Hz, 1H), 7.66-7.60 (m, 1H), 7.59-7.53 (m, 1H), 7.33 (d, *J* = 8.4 Hz, 1H), 6.95 (d, *J* = 8.4 Hz, 1H), 4.75 (dd, *J* = 8.4, 4.4 Hz, 1H), 2.36-2.29 (m, 1H), 2.14 (s, 3H), 1.51 (s, 9H), 1.05 (d, *J* = 6.8 Hz, 3H), 1.04 (d, *J* = 6.8 Hz, 3H); ^{13}C NMR (100 MHz, $CDCl_3$) δ 197.6, 171.6, 166.5, 153.2, 148.7, 141.6, 133.77, 133.75, 133.0, 132.8, 131.3, 129.9, 129.4, 128.4, 127.7, 126.3, 125.4, 122.9, 82.7, 58.0, 32.0, 28.2, 21.7, 19.1, 18.1; HRMS *m/z* Calcd. for $C_{27}H_{31}N_2O_5$ (M + H$^+$):463.2227; Found: 463.2232.

Compound (*S*,*S*)-6b[1]

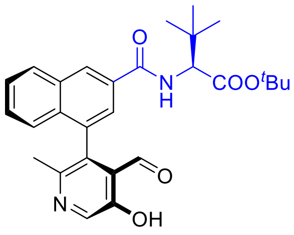

(*S*,*S*)-**6b**

Yellow solid; ^{1}H NMR (400 MHz, $CDCl_3$) δ 10.89 (s, 1H), 9.39 (s, 1H), 8.61 (s, 1H), 8.40 (s, 1H), 8.07 (d, *J* = 7.6 Hz, 1H), 7.83 (d, *J* = 2.0 Hz, 1H), 7.66-7.61 (m, 1H), 7.59-7.54 (m, 1H), 7.35 (d, *J* = 8.8 Hz, 1H), 6.90 (d, *J* = 9.2 Hz, 1H), 4.65 (d, *J* = 9.2 Hz, 1H), 2.16 (s, 3H), 1.52 (s, 9H), 1.10 (s, 9H).

Compound (*S*,*S*)-6c[1]

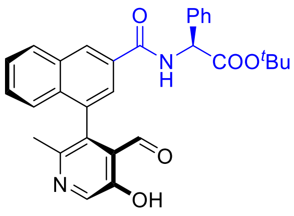

(*S*,*S*)-**6c**

Yellow solid; ^{1}H NMR (400 MHz, $CDCl_3$) δ 10.89 (s, 1H), 9.37 (s, 1H), 8.60 (s, 1H), 8.47 (s, 1H), 8.06 (d, *J* = 8.4 Hz, 1H), 7.83 (d, *J* = 1.6 Hz, 1H), 7.65-7.60 (m, 1H), 7.59-7.54 (m, 1H), 7.50-7.46 (m, 2H), 7.42-7.31 (m, 5H), 5.73 (d, *J* = 7.2 Hz, 1H), 2.13 (s, 3H), 1.44 (s, 9H).

Compound (*S*,*S*)-6d[1]

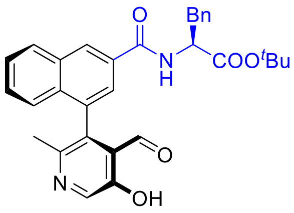

(*S*,*S*)-**6d**

Yellow solid; ^{1}H NMR (400 MHz, $CDCl_3$) δ 10.90 (s, 1H), 9.37 (s, 1H), 8.60 (s, 1H), 8.34 (s, 1H), 8.03 (d, *J* = 8.4 Hz, 1H), 7.76 (d, *J* = 1.6 Hz, 1H), 7.65-7.60 (m, 1H), 7.59-7.53 (m, 1H), 7.34 (d, *J* = 8.0 Hz, 1H), 7.31-7.26 (m, 2H), 7.26-7.18 (m, 3H), 6.84 (d, *J* = 7.6 Hz, 1H), 5.02 (td, *J* = 7.2, 5.6 Hz, 1H), 3.35-3.23 (m, 2H), 2.15 (s, 3H), 1.46 (s, 9H).

Compound (*S*)-6e[1]

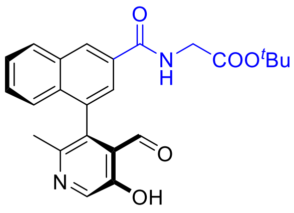

(*S*)-**6e**

Yellow solid; ^{1}H NMR (400 MHz, $CDCl_3$) δ 10.89 (s, 1H), 9.38 (s, 1H), 8.60 (s, 1H), 8.45 (s, 1H), 8.06 (d, *J* = 8.0 Hz, 1H), 7.84 (d, *J* = 1.6 Hz, 1H), 7.63 (dd, *J* = 8.0, 7.6 Hz, 1H), 7.57 (dd, *J* = 8.0, 6.8 Hz, 1H), 7.34 (d, *J* = 8.0 Hz, 1H), 6.88 (t, *J* = 4.4 Hz, 1H), 4.22 (d, *J* = 4.8 Hz, 2H), 2.14 (s, 3H), 1.52 (s, 9H).

Compound (*S*)-6f[1]

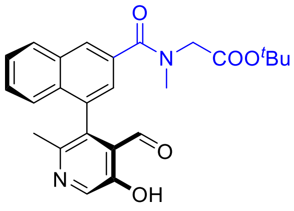

(*S*)-**6f**

Yellow solid; ^{1}H NMR (400 MHz, $CDCl_3$) δ 10.89 (s, 1H), 9.42 (s, 0.60H, major isomer), 9.40 (s, 0.40H, minor isomer), 8.59 (s, 1H), 8.14 (s, 0.60H, major isomer), 8.07 (s, 0.40H, minor isomer), 8.01 (d, *J* = 8.0 Hz, 0.60H, major isomer), 7.95 (d, *J* = 8.0 Hz, 0.40H, minor isomer), 7.61 (t, *J* = 7.2 Hz, 1H), 7.55-7.51 (m, 1.60H, major isomer), 7.44 (s, 0.40H, minor isomer), 7.34 (s, 0.60H, major isomer), 7.32 (s, 0.40H, minor isomer), 4.23 (s, 1.20H, major isomer), 3.96 (s, 0.80H, minor isomer), 3.18 (s, 1.20H, minor isomer), 3.12 (s, 1.80H, major isomer), 2.17 (s, 2H, major isomer), 2.15 (s, 1H, minor isomer), 1.52 (s, 5.40H, major isomer), 1.40 (s, 3.60H, minor isomer).

Compound (*R*,*S*)-7[1]

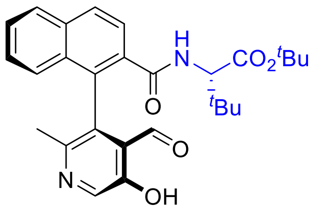

(*R*,*S*)-**7**

Yellow solid; ^{1}H NMR (400 MHz, $CDCl_3$) δ 10.65 (brs, 1H), 9.29 (s, 1H), 8.43 (s, 1H), 8.03-7.90 (m, 2H), 7.66 (d, *J* = 8.8 Hz, 1H), 7.57 (dd, *J* = 7.6, 7.2 Hz, 1H), 7.44 (t, *J* = 8.0 Hz, 1H), 7.33-7.27 (m, 1H), 6.40 (s, 1H), 4.29 (d, *J* = 9.2 Hz, 1H), 2.04 (s, 3H), 1.43 (s, 9H), 0.76 (s, 9H).

Compound (*S*,*S*)-7[1]

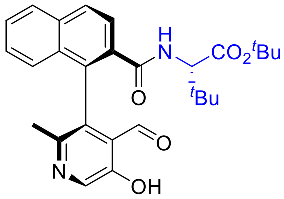

(*S*,*S*)-**7**

Yellow solid; ^{1}H NMR (400 MHz, $CDCl_3$) δ 13.65 (s, 1H), 8.50 (s, 1H), 8.02 (d, *J* = 8.4 Hz, 1H), 7.95 (d, *J* = 8.4 Hz, 1H), 7.85 (d, *J* = 8.4 Hz, 1H), 7.63 (s, 1H), 7.61-7.54 (m, 1H), 7.48-7.41 (m, 1H), 7.23 (d, *J* = 8.4 Hz, 1H), 6.13 (d, *J* = 9.2 Hz, 1H), 4.31 (d, *J* = 9.2 Hz, 1H), 2.08 (s, 3H), 1.40 (s, 9H), 0.94 (s, 9H).

4. Synthesis of MBH Acetates 2

4.1 Method A: Synthesis of 2a, 2s-2z, 2aa-2ai, 2ak-2al via Morita-Baylis-Hillman Reaction with Compound 2a as the Representative Example[1]

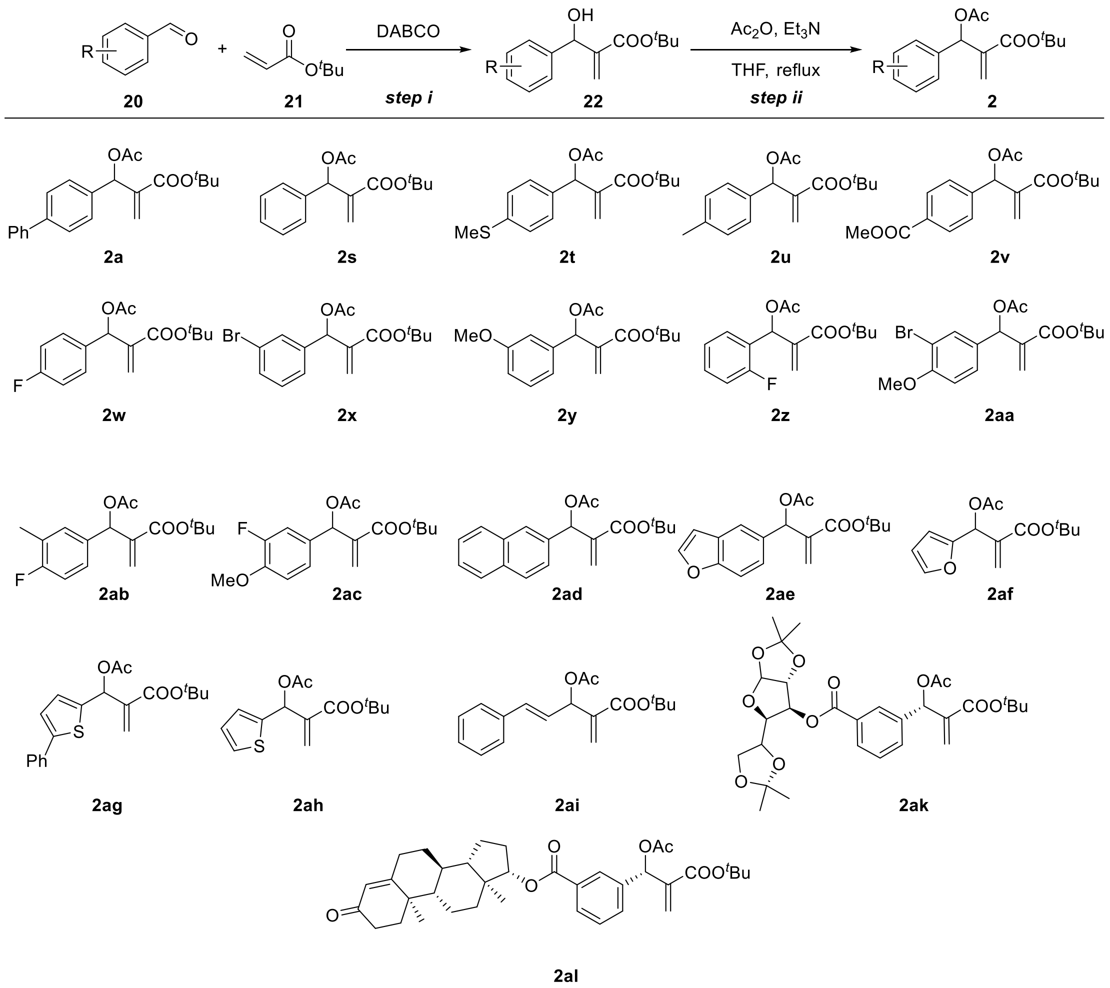

***Step i.* Synthesis of compound 22a (R = 4-Ph)**

To a 250 mL round-bottom flask were added compound 4-phenylbenzaldehyde (**20a**) (10.00 g, 54.95 mmol), *tert*-butyl acrylate (**21**) (25.32 g, 197.8 mmol), and 1,4-diazabicyclo [2.2.2] octane (DABCO) (9.231 g, 82.42 mmol). The mixture was stirred at 85 °C for 3 days. The reaction mixture was then concentrated via rotary evaporation and purified by chromatography on silica gel (petroleum ether : ethyl acetate = 10 : 1) to give compound MBH adduct **22a** (14.13 g, 83%) as a white solid.

22a[2]: ^{1}H NMR (400 MHz, $CDCl_3$) δ 7.62-7.56 (m, 4H), 7.47-7.41 (m, 4H), 7.38-7.32 (m, 1H), 6.31-6.26 (m, 1H), 5.78 (t, *J* = 1.2 Hz, 1H), 5.56 (d, *J* = 6.0 Hz, 1H), 3.15 (d, *J* = 6.0 Hz, 1H), 1.43 (s, 9H).

***Step ii.* Synthesis of compound 2a (R = 4-Ph)**

To a 100 mL round-bottom flask were added compound **22a** (3.000 g, 9.677 mmol), Ac_2O (3.948 g, 38.71 mmol), Et_3N (5.865 g, 58.06 mmol), and THF (30 mL) at room temperature. The mixture was refluxed at 80 ^{o}C for 24 h. Then the reaction mixture was cooled and concentrated via rotary evaporator to remove most of the organic solvent. To the residue was added H_2O (50 mL), and the resulting mixture was extracted with ethyl acetate (50 mL × 3). The organic layers were combined, dried over anhydrous Na_2SO_4, filtered, concentrated via rotary evaporation, and purified by chromatography on silica gel (petroleum ether : ethyl acetate = 20 : 1) to give compound **2a** (2.400 g, 70%) as a white solid.

2a[1]: White solid; ^{1}H NMR (400 MHz, $CDCl_3$) δ 7.62-7.56 (m, 4H), 7.49-7.42 (m, 4H), 7.36 (t, *J* = 7.2 Hz, 1H), 6.71 (s, 1H), 6.37 (s, 1H), 5.81 (s, 1H), 2.13 (s, 3H), 1.41 (s, 9H); ^{13}C NMR (100 MHz, $CDCl_3$) δ 169.6, 164.3, 141.3, 141.1, 140.7, 137.1, 128.9, 128.4, 127.5, 127.2, 124.9, 81.6, 73.2, 28.0, 21.2.

MBH acetates **2s-ai**, **2ak-al** were prepared by following a similar procedure.

Compound 2s[3]

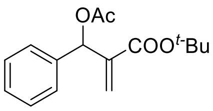

2s

Yellow oil; ^{1}H NMR (400 MHz, $CDCl_3$) δ 7.39-7.27 (m, 5H), 6.63 (s, 1H), 6.32 (s, 1H), 5.72 (s, 1H), 2.09 (s, 3H), 1.36 (s, 9H).

Compound 2t[1]

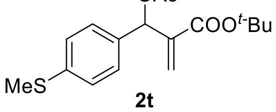

2t

Yellow oil; ^{1}H NMR (400 MHz, $CDCl_3$) δ 7.28 (d, *J* = 8.4 Hz, 2H), 7.21 (d, *J* = 8.4 Hz, 2H), 6.58 (s, 1H), 6.31 (s, 1H), 5.73 (s, 1H), 2.47 (s, 3H), 2.09 (s, 3H), 1.38 (s, 9H).

Compound 2u[3]

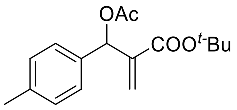

2u

Yellow oil; ^{1}H NMR (400 MHz, $CDCl_3$) δ 7.27 (d, *J* = 8.0 Hz, 2H), 7.14 (d, *J* = 7.6 Hz, 2H), 6.61 (s, 1H), 6.30 (s, 1H), 5.72 (s, 1H), 2.33 (s, 3H), 2.08 (s, 3H), 1.38 (s, 9H).

Compound 2v[4]

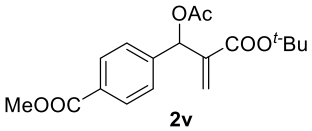

2v

White solid; ^{1}H NMR (400 MHz, $CDCl_3$) δ 8.01 (d, *J* = 8.4 Hz, 2H), 7.43 (d, *J* = 8.4 Hz, 2H), 6.65 (s, 1H), 6.35 (s, 1H), 5.76 (s, 1H), 3.91 (s, 3H), 2.11 (s, 3H), 1.37 (s, 9H).

Compound 2w[5]

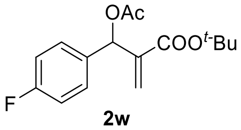

2w

Yellow oil; ^{1}H NMR (400 MHz, $CDCl_3$) δ 7.33 (dd, *J* = 8.4, 5.2 Hz, 2H), 7.00 (t, *J* = 8.8 Hz, 2H), 6.59 (s, 1H), 6.31 (s, 1H), 5.74 (s, 1H), 2.07 (s, 3H), 1.35 (s, 9H).

Compound 2x

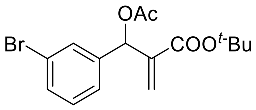

2x

Yellow oil; ^{1}H NMR (400 MHz, $CDCl_3$) δ 7.49 (s, 1H), 7.41 (d, *J* = 7.6 Hz, 1H), 7.29 (d, *J* = 7.6 Hz, 1H), 7.19 (t, *J* = 8.0 Hz, 1H), 6.56 (s, 1H), 6.33 (s, 1H), 5.75 (s, 1H), 2.09 (s, 3H), 1.37 (s, 9H); ^{13}C NMR (100 MHz, $CDCl_3$) δ 169.4, 163.9, 140.6, 140.5, 131.4, 130.9, 130.0, 126.6, 125.3, 122.4, 81.8, 72.6, 28.0, 21.1; HRMS *m/z* Calcd. for $C_{16}H_{19}BrNaO_4$ (M + Na^+): 377.0359; Found: 377.0359.

Compound 2y[3]

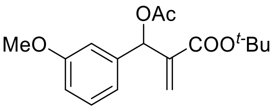

2y

Yellow oil; ^{1}H NMR (400 MHz, $CDCl_3$) δ 7.25 (t, *J* = 8.0 Hz, 1H), 6.95 (d, *J* = 7.6 Hz, 1H), 6.89 (s, 1H), 6.84 (dd, *J* = 8.4, 2.8 Hz, 1H), 6.61 (s, 1H), 6.31 (s, 1H), 5.70 (s, 1H), 3.79 (s, 3H), 2.10 (s, 3H), 1.38 (s, 9H).

Compound 2z

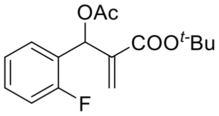

2z

Yellow oil; ^{1}H NMR (400 MHz, $CDCl_3$) δ 7.35-7.27 (m, 2H), 7.12 (dd, *J* = 8.0, 7.2 Hz, 1H), 7.05 (dd, *J* = 9.2, 8.8 Hz, 1H), 6.92 (s, 1H), 6.38 (s, 1H), 5.70 (s, 1H), 2.11 (s, 3H), 1.38 (s, 9H); ^{13}C NMR (100 MHz, $CDCl_3$) δ 169.2, 164.0, 160.4 (d, $J_{C\text{-}F}$ = 248.1 Hz), 140.0, 130.2 (d, $J_{C\text{-}F}$ = 8.3 Hz), 129.0 (d, $J_{C\text{-}F}$ = 3.3 Hz), 125.44, 125.41 (d, $J_{C\text{-}F}$ = 13.6 Hz), 124.1 (d, $J_{C\text{-}F}$ = 3.6 Hz), 115.7 (d, $J_{C\text{-}F}$ = 21.4 Hz), 81.7, 67.4 (d, $J_{C\text{-}F}$ = 3.6 Hz), 27.9, 21.0; ^{19}F NMR (376 MHz, $CDCl_3$) δ -116.6; HRMS *m/z* Calcd. for $C_{16}H_{19}FNaO_4$ (M + Na^+): 317.1160; Found: 317.1162.

Compound 2aa

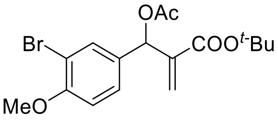

2aa

White solid; M.p. 58-62 °C; ^{1}H NMR (400 MHz, $CDCl_3$) δ 7.52 (d, *J* = 2.4 Hz, 1H), 7.28 (dd, *J* = 8.4, 2.0 Hz, 1H), 6.84 (d, *J* = 8.4 Hz, 1H), 6.52 (s, 1H), 6.31 (s, 1H), 5.76 (s, 1H), 3.87 (s, 3H), 2.08 (s, 3H), 1.37 (s, 9H); ^{13}C NMR (100 MHz, $CDCl_3$) δ 169.5, 164.1, 155.9, 140.8, 132.9, 131.8, 128.5, 124.7, 111.6, 111.5, 81.7, 72.5, 56.4, 28.0, 21.2; HRMS *m/z* Calcd. for $C_{17}H_{21}BrNaO_5$ (M + Na^+): 407.0465; Found: 407.0465.

Compound 2ab

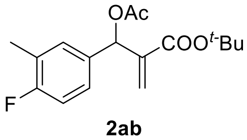

2ab

White solid; M.p. 65-69 °C; ^{1}H NMR (400 MHz, $CDCl_3$) δ 7.20-7.11 (m, 2H), 6.95 (t, J = 8.8 Hz, 1H), 6.56 (s, 1H), 6.31 (s, 1H), 5.73 (s, 1H), 2.25 (d, J = 2.0 Hz, 3H), 2.09 (s, 3H), 1.37 (s, 9H); ^{13}C NMR (100 MHz, $CDCl_3$) δ 169.5, 164.2, 161.2 (d, $J_{C\text{-}F}$ = 244.2 Hz), 141.1, 133.7 (d, $J_{C\text{-}F}$ = 3.6 Hz), 131.2 (d, $J_{C\text{-}F}$ = 5.4 Hz), 127.0 (d, $J_{C\text{-}F}$ = 8.3 Hz), 124.9 (d, $J_{C\text{-}F}$ = 17.5 Hz), 124.6, 115.0 (d, $J_{C\text{-}F}$ = 22.5 Hz), 81.6, 72.9, 28.0, 21.2, 14.6 (d, $J_{C\text{-}F}$ = 3.6 Hz); ^{19}F NMR (376 MHz, $CDCl_3$) δ -117.9; HRMS *m/z* Calcd. for $C_{17}H_{21}FNaO_4$ (M + Na^+): 331.1316; Found: 331.1315.

Compound 2ac

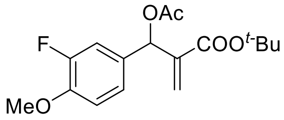

2ac

Yellow oil;^{1}H NMR (400 MHz, $CDCl_3$) δ 7.13-7.05 (m, 2H), 6.90 (t, J = 8.8 Hz, 1H), 6.54 (s, 1H), 6.31 (s, 1H), 5.74 (s, 1H), 3.87 (s, 3H), 2.08 (s, 3H), 1.38 (s, 9H); ^{13}C NMR (100 MHz, $CDCl_3$) δ 169.5, 164.2, 152.2 (d, $J_{C\text{-}F}$ = 244.7 Hz), 147.7 (d, $J_{C\text{-}F}$ = 10.6 Hz), 140.9, 131.1 (d, $J_{C\text{-}F}$ = 5.9 Hz), 124.7, 124.2 (d, $J_{C\text{-}F}$ = 3.6 Hz), 115.7 (d, $J_{C\text{-}F}$ = 18.8 Hz), 113.0 (d, $J_{C\text{-}F}$ = 2.0 Hz), 81.7, 72.6 (d, $J_{C\text{-}F}$ = 1.5 Hz), 56.3, 28.0, 21.2; ^{19}F NMR (376 MHz, $CDCl_3$) δ -134.9; HRMS *m/z* Calcd. for $C_{17}H_{21}FNaO_5$ (M + Na^+): 347.1265; Found: 347.1264.

Compound 2ad[3]

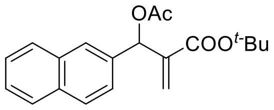

2ad

Yellow oil; ^{1}H NMR (400 MHz, $CDCl_3$) δ 7.89-7.80 (m, 4H), 7.53-7.46 (m, 3H), 6.85 (s, 1H), 6.40 (s, 1H), 5.82 (s, 1H), 2.13 (s, 3H), 1.38 (s, 9H).

Compound 2ae[1]

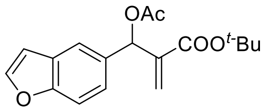

2ae

White solid; ^{1}H NMR (400 MHz, $CDCl_3$) δ 7.62 (d, J = 2.0 Hz, 2H), 7.46 (d, J = 8.8 Hz, 1H), 7.31 (dd, J = 8.4, 1.6 Hz, 1H), 6.78-6.72 (m, 2H), 6.33 (s, 1H), 5.77 (s, 1H), 2.09 (s, 3H), 1.35 (s, 9H).

Compound 2af

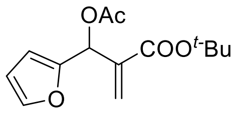

2af

Yellow oil; ^{1}H NMR (400 MHz, $CDCl_3$) δ 7.36 (d, *J* = 1.2 Hz, 1H), 6.68 (s, 1H), 6.34 (s, 1H), 6.33-6.30 (m, 1H), 6.29 (d, *J* = 3.2 Hz, 1H), 5.83 (s, 1H), 2.08 (s, 3H), 1.38 (s, 9H); ^{13}C NMR (100 MHz, $CDCl_3$) δ 169.4, 163.9, 151.1, 142.9, 138.6, 125.3, 110.5, 109.5, 81.6, 66.5, 27.9, 21.0; HRMS *m/z* Calcd. for $C_{14}H_{18}NaO_5$ (M + Na^+): 289.1046; Found: 289.1047.

Compound 2ag

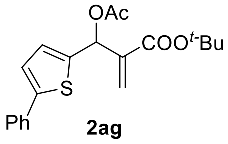

2ag

Yellow solid; M.p. 67-70 °C; ^{1}H NMR (400 MHz, $CDCl_3$) δ 7.57 (d, *J* = 7.2 Hz, 2H), 7.36 (t, *J* = 7.6 Hz, 2H), 7.28 (dd, *J* = 7.6, 6.4 Hz, 1H), 7.17 (d, *J* = 3.6 Hz, 1H), 7.03 (d, *J* = 3.6 Hz, 1H), 6.85 (s, 1H), 6.38 (s, 1H), 5.93 (s, 1H), 2.13 (s, 3H), 1.42 (s, 9H); ^{13}C NMR (100 MHz, $CDCl_3$) δ 169.6, 164.0, 145.2, 140.6, 140.5, 134.2, 129.0, 128.2, 127.8, 125.9, 124.8, 122.8, 81.9, 68.7, 28.1, 21.3; HRMS *m/z* Calcd. for $C_{20}H_{22}NaO_4S$ (M + Na^+): 381.1131; Found: 381.1134.

Compound 2ah[1]

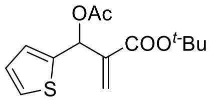

2ah

Yellow oil; ^{1}H NMR (400 MHz, $CDCl_3$) δ 7.27 (d, *J* = 5.2 Hz, 1H), 7.10-7.04 (m, 1H), 7.00-6.94 (m, 1H), 6.89 (s, 1H), 6.35 (s, 1H), 5.88 (s, 1H), 2.10 (s, 3H), 1.40 (s, 9H).

Compound 2ai[1]

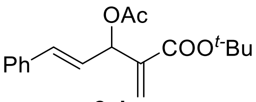

2ai

Yellow oil; ^{1}H NMR (400 MHz, $CDCl_3$) δ 7.41-7.36 (m, 2H), 7.34-7.29 (m, 2H), 7.28-7.23 (m, 1H), 6.67 (d, *J* = 15.2 Hz, 1H), 6.29 (s, 1H), 6.27-6.16 (m, 2H), 5.82 (s, 1H),

2.12 (s, 3H), 1.49 (s, 9H); ^{13}C NMR (100 MHz, $CDCl_3$) δ 169.6, 164.4, 140.7, 136.2, 133.6, 128.7, 128.2, 126.8, 125.6, 125.2, 81.6, 72.2, 28.2, 21.3.

Compound 2ak

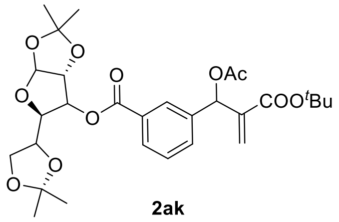

2ak

A pair of diastereomers were obtained. Colorless oil; ^{1}H NMR (400 MHz, $CDCl_3$) δ 7.99 (d, *J* = 1.6 Hz, 1H), 7.93 (d, *J* = 7.6 Hz, 1H), 7.59 (d, *J* = 7.2 Hz, 1H), 7.41 (t, *J* = 7.6 Hz, 1H), 6.63 (d, *J* = 1.6 Hz, 1H), 6.32 (s, 1H), 5.93 (d, *J* = 3.6 Hz, 0.50H, isomer one), 5.91 (d, *J* = 3.6 Hz, 0.50H, isomer two), 5.76 (s, 0.50H, isomer one), 5.73 (s, 0.50H, isomer two), 5.49-5.43 (m, 1H), 4.60 (d, *J* = 3.6 Hz, 1H), 4.37-4.26 (m, 2H), 4.13-4.02 (m, 2H), 2.08 (s, 3H), 1.53 (s, 3H), 1.38 (s, 3H), 1.364 (s, 4.5H, isomer one), 1.357 (s, 4.5H, isomer two),1.29 (s, 3H), 1.24 (s, 1.5H, isomer one), 1.23 (s, 1.5H, isomer two); ^{13}C NMR (100 MHz, $CDCl_3$) δ 169.39, 169.38, 165.0, 164.9, 163.93, 163.89, 140.66, 140.63, 139.08, 139.06, 133.1, 133.0, 129.8, 129.6, 129.1, 129.0, 128.7, 125.5, 125.3, 112.40, 112.39, 109.45, 109.42, 105.17, 105.15, 83.41, 83.39, 81.8, 80.0, 79.95, 76.8, 72.8, 72.62, 72.60, 67.31, 67.27, 28.0, 26.9, 26.8, 26.2, 25.24, 25.21, 21.12, 21.09; HRMS *m/z* Calcd. for $C_{29}H_{38}NaO_{11}$ (M + Na^+): 585.2306; Found: 585.2309.

Compound 2al

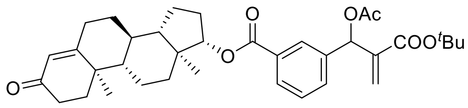

2al

A pair of diastereomers were obtained. White solid; M.p. 48-52 °C; ^{1}H NMR (400 MHz, $CDCl_3$) δ 8.02 (s, 1H), 7.96 (d, *J* = 7.6 Hz, 1H), 7.56 (dd, *J* = 8.0, 2.0 Hz, 1H), 7.41 (t, *J* = 8.0 Hz, 1H), 6.65 (s, 1H), 6.34 (s, 1H), 5.76 (s, 1H), 5.74 (s, 1H), 4.84 (t, *J* = 8.4 Hz, 1H), 2.44-2.35 (m, 2H), 2.34-2.25 (m, 2H), 2.10 (s, 3H), 2.07-1.99 (m, 1H), 1.91-1.82 (m, 2H), 1.78-1.56 (m, 6H), 1.49-1.40 (m, 2H), 1.37 (s, 9H), 1.30-1.22 (m, 2H), 1.20 (s, 3H), 1.17-1.03 (m, 2H), 0.97 (s, 3H); ^{13}C NMR (100 MHz, $CDCl_3$) δ 199.6, 171.1, 169.5, 166.2, 164.1, 140.8, 138.8, 132.44, 132.36, 130.94, 130.92, 129.5, 129.0, 128.6, 125.4, 125.3, 124.1, 83.3, 83.2, 81.8, 73.1, 53.8, 50.4, 43.01, 42.99, 38.7, 36.8,

35.8, 35.5, 34.0, 32.9, 31.6, 28.0, 27.8, 23.7, 21.2, 20.7, 17.5, 12.4; HRMS *m/z* Calcd. for $C_{36}H_{46}NaO_7$ (M + Na^+): 613.3136; Found: 613.3137.

4.2 Method B: Synthesis of 2aj via Morita-Baylis-Hillman Reaction

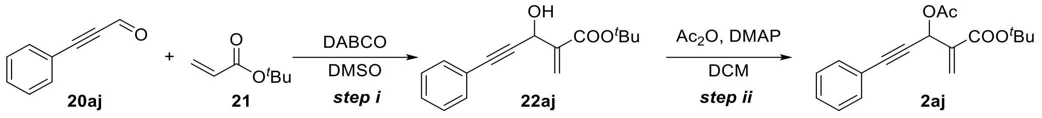

***Step i.* Synthesis of compound 22aj**

To a 5 mL vial were added compound 3-phenylpropiolaldehyde (**20aj**) (0.260 g, 2.00 mmol), *tert*-butyl acrylate (**21**) (0.206 g, 2.40 mmol), 1,4-diazabicyclo[2.2.2] octane (DABCO) (0.112 g, 1.00 mmol) and DMSO (2 mL). The mixture was stirred at room temperature for 12 hours. Then the reaction mixture was concentrated via rotary evaporation and purified by chromatography on silica gel (petroleum ether : ethyl acetate = 30 : 1) to give compound **22aj** (0.236 g, 46%) as a brown oil.

22aj[6]: ^{1}H NMR (400 MHz, $CDCl_3$) δ 7.46-7.42 (m, 2H), 7.33-7.29 (m, 3H), 6.25 (s, 1H), 6.08 (s, 1H), 5.39 (d, *J* = 6.4 Hz, 1H), 3.35 (d, *J* = 6.8 Hz, 1H), 1.54 (s, 9H).

***Step ii.* Synthesis of compound 2aj**

To a 25 mL round-bottom flask were added compound **22aj** (0.2000 g, 0.78 mmol), Ac_2O (0.119 g, 1.17 mmol), 4-dimethylaminopyridine (0.0260 g, 0.23 mmol), and DCM (5 mL) at 0 °C for 1 h. To the reaction mixture was added H_2O (5 mL), and then the resulting mixture was extracted with DCM (5 mL × 3). The organic layers were combined, dried over anhydrous Na_2SO_4, filtered, concentrated via rotary evaporation, and purified by chromatography on silica gel (petroleum ether : ethyl acetate = 20 : 1) to give compound **2aj** (0.1400 g, 60%) as a yellow oil.

2aj[6]: Yellow oil; ^{1}H NMR (400 MHz, $CDCl_3$) δ 7.47-7.43 (m, 2H), 7.34-7.29 (m, 3H), 6.51 (s, 1H), 6.42 (s, 1H), 6.22 (s, 1H), 2.13 (s, 3H), 1.50 (s, 9H); ^{13}C NMR (100 MHz, $CDCl_3$) δ 169.5, 163.8, 138.3, 132.0, 129.0, 128.4, 128.1, 122.1, 87.1, 84.1, 81.9, 62.4, 28.2, 21.1.

5. Synthesis of Benzylamine 1am

The chiral benzylamine **1am** was prepared through the following steps.

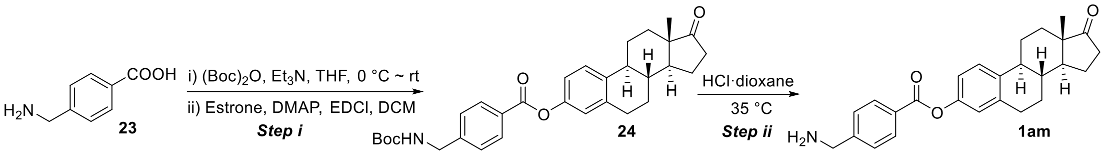

Step i. Synthesis of compound 24

To a solution of 4-(aminomethyl) benzoic acid **23** (1.500 g, 9.923 mmol) in THF (10 mL) was added $(Boc)_2O$ (2.180 g, 9.993 mmol) and Et_3N (1.000 g, 9.882 mmol) dropwise at 0 ^oC. Then the reaction was allowed to warm to room temperature and stirred at the same temperature for 12 h. Upon reaction completion, the reaction mixture was concentrated under reduced pressure, and the residue was purified by column chromatography (DCM : MeOH = 30:1) to afford N-Boc protected compound (1.301 g, 52% yield) as a white solid.

To a 5 mL vial of the above N-Boc protected compound (0.301 g, 1.199 mmol) in DCM (1.0 mL) was added Estrone (0.270 g, 1.000 mmol) and 1-(3-dimethylaminopropyl)-3-ethylcarbodiimide hydrochloride (EDCl) (0.192 g, 1.000 mmol), 4-dimethylaminopyridine (DMAP) (0.012 g, 0.0982 mmol). The reaction mixture was stirred at room temperature for 24 h. Then the mixture was concentrated via rotary evaporator to remove all volatiles. The residue was diluted with H_2O (5 mL) and extracted with DCM (5 mL × 3), dried over Na_2SO_4, filtered, evaporated in vacuo and purified by column chromatography on silica gel (petroleum ether : ethyl acetate = 30 : 1) to give compound **24** (0.420 g, 70% yield) as a white solid.

24: White solid; ^1H NMR (400 MHz, $CDCl_3$) δ 8.15 (d, *J* = 8.4 Hz, 2H), 7.41 (d, *J* = 8.4 Hz, 2H), 7.34 (d, *J* = 8.4 Hz, 1H), 6.98 (dd, *J* = 8.4, 2.4 Hz, 1H), 6.94 (d, *J* = 2.4 Hz, 1H), 4.96 (s, 1H), 4.41 (d, *J* = 6.0 Hz, 2H), 2.99-2.90 (m, 2H), 2.52 (dd, *J* = 19.2, 8.8 Hz, 1H), 2.47-2.40 (m, 1H), 2.32 (td, *J* = 10.8, 4.0 Hz, 1H), 2.21-2.03 (m, 3H), 2.00-1.94 (m, 1H), 1.70-1.58 (m, 3H), 1.56 (d, *J* = 4.8 Hz, 1H), 1.55-1.49 (m, 2H), 1.47 (s, 9H), 0.93 (s, 3H); HRMS *m/z* Calcd. for $C_{27}H_{29}NO_5$ (M + H^+): 448.2118; Found: 448.2120.

Step ii. Synthesis of compound 1am

To a solution of **24** (0.420 g, 0.835 mmol) in THF (1 mL) was added 4.0 M HCl dioxane solution (0.8 mL, 3.2 mmol) at 35 ^oC, and the reaction mixture was stirred

overnight. The solution was diluted with petroleum ether (3 mL) and a white precipitate appeared. The organic layer was removed by decantation, and the precipitate was washed with petroleum ether three times. To the suspension of the precipitate in THF (4 mL) was added 2.0 M NaOH aqueous solution (4 mL). The mixture was stirred for 0.5 h to release the free amine. Then the mixture was extracted with DCM (10 mL × 3). The combined organic layers were washed with brine, dried with anhydrous Na_2SO_4, filtered, concentrated via rotary evaporation to give compound **1am** (0.200 g, 59%) as a white solid.

1am: White solid; M.p. 163-167 °C; ^{1}H NMR (400 MHz, $CDCl_3$) δ 8.16 (d, *J* = 8.0 Hz, 2H), 7.46 (d, *J* = 8.0 Hz, 2H), 7.34 (d, *J* = 8.4 Hz, 1H), 6.98 (dd, *J* = 8.4, 2.4 Hz, 1H), 6.95 (d, *J* = 2.4 Hz, 1H), 3.98 (s, 2H), 2.99-2.87 (m, 2H), 2.57-2.39 (m, 2H), 2.37-2.27 (m, 1H), 2.21-2.12 (m, 1H), 2.07-1.97 (m, 2H), 1.66-1.45 (m, 9H), 0.92 (s, 3H); ^{13}C NMR (100 MHz, $CDCl_3$) δ 221.0, 165.5, 149.2, 148.9, 138.2, 137.5, 130.6, 128.3, 127.2, 126.6, 121.8, 119.0, 50.5, 48.1, 46.3, 44.3, 38.1, 36.0, 31.7, 29.5, 26.5, 25.9, 21.7, 13.9; HRMS *m/z* Calcd. for $C_{26}H_{30}NO_3$ $(M + H^+)$: 404.2220; Found: 404.2223.

6. Procedure for Asymmetric α-C Alkylation

6.1 Representative Procedure for Asymmetric α-C Allylation of Benzylamine 1a with MBH Acetates (Table 2, for 3a, Ar = 4-PhC6H4)

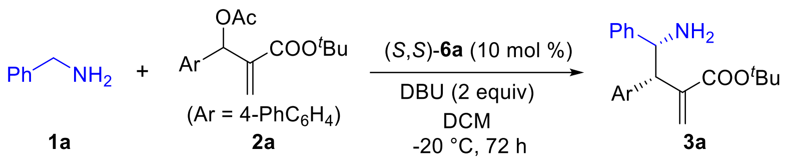

To a 4 mL vial were successively added chiral pyridoxal (*S*,*S*)-**6a** (0.0046 g, 0.010 mmol), DBU (0.0304 g, 0.20 mmol), DCM (0.3 mL) and benzylamine **1a** (0.0214 g, 0.20 mmol). The mixture was stirred at -20 °C for 5 min, and a solution of **2a** (0.0352 g, 0.10 mmol) in DCM (0.2 mL) was added in 7 portions over 1 h (the first portion: 20 mol% of the solution of **2a**; the rest of **2a** was then divided into 6 portions to be added into the reaction vial). After the reaction mixture was stirred at -20 °C for 72 h, it was allowed to warm up to room temperature and then concentrated via rotary evaporator to remove most of the solvent. The residue was submitted to purification by column chromatography on silica gel (petroleum ether : ethyl acetate = 3 : 1) to give compound **3a** (0.0283 g, 71%) as a white solid.

The dr values of products **3a-am** were determined by ^{1}H NMR analysis of the crude reaction mixtures. The enantiomeric excesses (ee's) of products **3a-3z**,**3aa-3am** were determined by chiral HPLC analysis.

Compound 3a

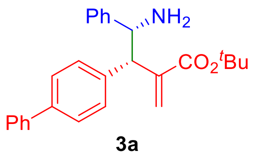

3a

White solid; M.p. 85-89 °C; ^{1}H NMR (400 MHz, $CDCl_3$) δ 7.62-7.55 (m, 4H), 7.49-7.41 (m, 4H), 7.40-7.29 (m, 5H), 7.26-7.20 (m, 1H), 6.09 (s, 1H), 5.64 (s, 1H), 4.58 (d, *J* = 9.6 Hz, 1H), 4.27 (d, *J* = 10.0 Hz, 1H), 1.72 (brs, 2H), 1.32 (s, 9H); ^{13}C NMR (100 MHz, $CDCl_3$) δ 165.9, 143.8, 143.3, 140.9, 139.9, 139.8, 129.5, 128.9, 128.5, 127.8, 127.5, 127.37, 127.36, 127.1, 125.4, 80.9, 59.0, 54.0, 28.0; HRMS *m/z* Calcd. for $C_{27}H_{30}NO_2$ (M+H$^+$): 400.2271; Found: 400.2266.

Compound 3b

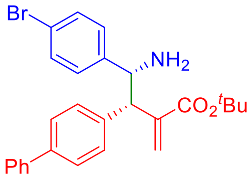

3b

Yellow solid; M.p. 90-95 °C; ^{1}H NMR (400 MHz, $CDCl_3$) δ 7.61-7.55 (m, 4H), 7.47-7.41 (m, 6H), 7.38-7.32 (m, 1H), 7.30-7.26 (m, 2H), 6.09 (s, 1H), 5.61 (s, 1H), 4.57 (d, *J* = 10.0 Hz, 1H), 4.18 (d, *J* = 9.6 Hz, 1H), 1.57 (brs, 2H), 1.33 (s, 9H); ^{13}C NMR (100 MHz, $CDCl_3$) δ 165.8, 143.2, 143.0, 140.8, 140.0, 139.5, 131.6, 129.6, 129.4, 128.9, 127.5, 127.4, 127.1, 125.6, 121.2, 81.0, 58.5, 54.2, 28.0; HRMS *m/z* Calcd. for $C_{27}H_{29}BrNO_2$ (M+H$^+$): 478.1376; Found: 478.1376.

Compound 3c

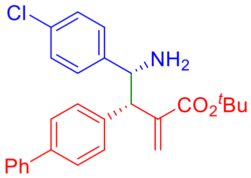

3c

White solid; M.p. 107-110 °C; ^{1}H NMR (400 MHz, $CDCl_3$) δ 7.61-7.56 (m, 4H), 7.47-7.41 (m, 4H), 7.37-7.31 (m, 3H), 7.30-7.26 (m, 2H), 6.09 (s, 1H), 5.61 (s, 1H), 4.58 (d, *J* = 9.6 Hz, 1H), 4.19 (d, *J* = 9.6 Hz, 1H), 1.57 (brs, 2H), 1.32 (s, 9H); ^{13}C NMR (100 MHz, $CDCl_3$) δ 165.8, 143.2, 142.5, 140.8, 140.0, 139.5, 133.1, 129.4, 129.2, 128.9, 128.6, 127.45, 127.42, 127.1, 125.6, 81.0, 58.4, 54.3, 28.0; HRMS *m/z* Calcd. for $C_{27}H_{29}ClNO_2$ (M+H$^+$): 434.1881; Found: 434.1881.

Compound 3d

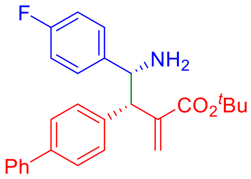

3d

White solid; M.p. 128-130 °C; ^{1}H NMR (400 MHz, $CDCl_3$) δ 7.62-7.55 (m, 4H), 7.49-7.42 (m, 4H), 7.39-7.32 (m, 3H), 6.99 (t, *J* = 8.8 Hz, 2H), 6.08 (s, 1H), 5.61 (s, 1H), 4.60 (d, *J* = 10.0 Hz, 1H), 4.21 (d, *J* = 10.0 Hz, 1H), 1.83 (brs, 2H), 1.32 (s, 9H); ^{13}C NMR (100 MHz, $CDCl_3$) δ 165.8, 162.2 (d, $J_{C\text{-}F}$ = 243.9 Hz), 143.2, 140.8, 140.0, 139.6,

139.3 (d, $J_{C\text{-}F}$ = 3.1 Hz), 129.41, 129.36 (d, $J_{C\text{-}F}$ = 7.8 Hz) ,128.9, 127.5, 127.4, 127.1, 125.6, 115.3 (d, $J_{C\text{-}F}$ = 21.0 Hz), 81.0, 58.3, 54.3, 28.0; ^{19}F NMR (376 MHz, $CDCl_3$) δ -115.4; HRMS *m/z* Calcd. for $C_{27}H_{29}FNO_2$ (M+H$^+$): 418.2177; Found: 418.2179.

Compound 3e

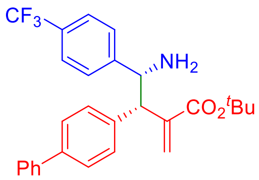

3e

White solid; M.p. 120-123 °C; ^{1}H NMR (400 MHz, $CDCl_3$) δ 7.64-7.56 (m, 6H), 7.52 (d, *J* = 8.4 Hz, 2H), 7.49-7.42 (m, 4H), 7.38-7.32 (m, 1H), 6.11 (s, 1H), 5.64 (s, 1H), 4.67 (d, *J* = 9.6 Hz, 1H), 4.25 (d, *J* = 10.0 Hz, 1H), 1.49 (brs, 2H), 1.31 (s, 9H); ^{13}C NMR (100 MHz, $CDCl_3$) δ 165.7, 148.1, 143.1, 140.8, 140.1, 139.4, 129.6 (q, $J_{C\text{-}F}$ = 32.0 Hz), 129.4, 128.9, 128.2, 127.5, 127.4, 127.1, 125.7, 125.4 (q, $J_{C\text{-}F}$ = 3.8 Hz), 124.3 (q, $J_{C\text{-}F}$ = 270.2 Hz), 81.1, 58.8, 54.1, 28.0; ^{19}F NMR (376 MHz, $CDCl_3$) δ -62.4; HRMS *m/z* Calcd. for $C_{28}H_{29}F_3NO_2$ (M+H$^+$): 468.2145; Found: 468.2141.

Compound 3f

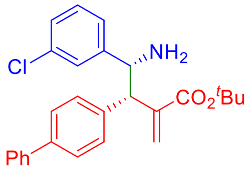

3f

Yellow solid; M.p. 71-76°C; ^{1}H NMR (400 MHz, $CDCl_3$) δ 7.63-7.55 (m, 4H), 7.48-7.41 (m, 4H), 7.39 (s, 1H), 7.37-7.32 (m, 1H), 7.29-7.26 (m, 1H), 7.25-7.20 (m, 2H), 6.10 (s, 1H), 5.63 (s, 1H), 4.58 (d, *J* =10.0 Hz, 1H), 4.19 (d, *J* = 9.6 Hz, 1H), 1.53 (brs, 2H), 1.34 (s, 9H); ^{13}C NMR (100 MHz, $CDCl_3$) δ 165.8, 146.2, 143.1, 140.9, 140.0, 139.5, 134.3, 129.8, 129.4, 128.9, 128.0, 127.6, 127.5, 127.4, 127.2, 126.0, 125.7, 81.1, 58.6, 54.2, 28.0; HRMS *m/z* Calcd. for $C_{27}H_{29}ClNO_2$ (M+H$^+$): 434.1881; Found: 434.1890.

Compound 3g

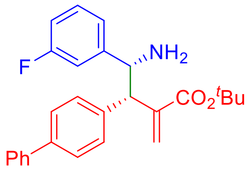

3g

Yellow solid; M.p. 130-133°C; ^{1}H NMR (400 MHz, $CDCl_3$) δ 7.63-7.55 (m, 4H), 7.50-7.41 (m, 4H), 7.35 (t, *J* = 7.2 Hz, 1H), 7.31-7.24 (m, 1H), 7.17 (d, *J* = 7.6 Hz, 1H), 7.12 (dd, *J* = 10.0, 2.0 Hz, 1H), 6.94 (td, *J* = 8.0, 2.4 Hz, 1H), 6.10 (s, 1H), 5.64 (s, 1H), 4.61 (d, *J* = 9.6 Hz, 1H), 4.21 (d, *J* = 10.0 Hz, 1H), 1.72 (brs, 2H), 1.34 (s, 9H); ^{13}C NMR (100 MHz, $CDCl_3$) δ 165.8, 163.0 (d, J_{C-F} = 244.1 Hz), 146.6 (d, J_{C-F} = 6.8 Hz), 143.1, 140.8, 140.0, 139.5, 129.9 (d, J_{C-F} = 8.0 Hz), 129.4, 128.9, 127.41, 127.39, 127.1, 125.6, 123.4 (d, J_{C-F} = 2.6 Hz), 114.6 (d, J_{C-F} = 21.4 Hz), 114.3 (d, J_{C-F} = 21.1 Hz), 81.0, 58.5 (d, J_{C-F} = 1.7 Hz), 54.2, 28.0; ^{19}F NMR (376 MHz, $CDCl_3$) δ -113.2; HRMS *m/z* Calcd. for $C_{27}H_{29}FNO_2$ (M+H^{+}): 418.2177; Found: 418.2184.

Compound 3h

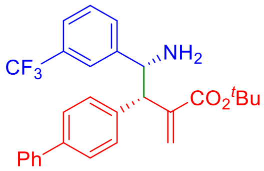

3h

Yellow solid; M.p. 58-62°C; ^{1}H NMR (400 MHz, $CDCl_3$) δ 7.65 (s, 1H), 7.64-7.56 (m, 5H), 7.53-7.40 (m, 6H), 7.35 (t, *J* = 7.2 Hz, 1H), 6.09 (s, 1H), 5.63 (s, 1H), 4.71 (d, *J* = 10.0 Hz, 1H), 4.23 (d, *J* = 10.0 Hz, 1H), 1.72 (brs, 2H), 1.33 (s, 9H); ^{13}C NMR (100 MHz, $CDCl_3$) δ 165.7, 144.8, 143.0, 140.8, 140.2, 139.3, 131.3, 130.7 (q, J_{C-F} = 31.9 Hz), 129.4, 128.9, 127.5, 127.4, 127.1, 125.9, 124.8 (q, J_{C-F} = 3.8 Hz), 124.32 (q, J_{C-F} = 3.8 Hz), 124.30 (q, J_{C-F} = 270.7 Hz), 81.1, 58.7, 54.4, 27.9; ^{19}F NMR (376 MHz, $CDCl_3$) δ -62.5; HRMS *m/z* Calcd. for $C_{28}H_{29}F_3NO_2$ (M+H^{+}): 468.2145; Found: 468.2146.

Compound 3i

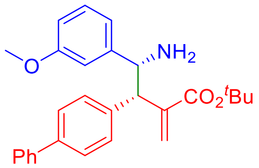

3i

Yellow solid; M.p. 53-57°C; ^{1}H NMR (400 MHz, $CDCl_3$) δ 7.62-7.55 (m, 4H), 7.48-7.41 (m, 4H), 7.37-7.31 (m, 1H), 7.23 (t, *J* = 7.6 Hz, 1H), 6.97 (d, *J* = 7.6 Hz, 1H), 6.95-6.93 (m, 1H), 6.79 (dd, *J* = 8.0, 2.4 Hz, 1H), 6.10 (s, 1H), 5.66 (s, 1H), 4.56 (d, *J* = 9.6 Hz, 1H), 4.25 (d, *J* = 10.0 Hz, 1H), 3.81 (s, 3H), 1.46 (brs, 2H), 1.33 (s, 9H); ^{13}C NMR (100 MHz, $CDCl_3$) δ 165.9, 159.8, 145.8, 143.3, 140.9, 139.88, 139.85, 129.5, 128.9, 127.3, 127.1, 125.3, 120.1, 113.1, 113.0, 80.8, 59.0, 55.3, 54.0, 28.0; HRMS *m/z* Calcd. for $C_{28}H_{32}NO_3$ $(M+H^+)$: 430.2377; Found: 430.2381.

Compound 3j

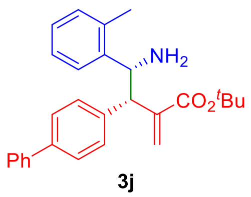

3j

Yellow solid; M.p. 107-110°C; ^{1}H NMR (400 MHz, $CDCl_3$) δ 7.63-7.57 (m, 4H), 7.47-7.42 (m, 4H), 7.37-7.32 (m, 2H), 7.21-7.12 (m, 3H), 6.10 (s, 1H), 5.62 (s, 1H), 4.74 (d, *J* = 9.6 Hz, 1H), 4.39 (d, *J* = 9.6 Hz, 1H), 2.48 (s, 3H), 1.38 (brs, 2H), 1.31 (s, 9H); ^{13}C NMR (100 MHz, $CDCl_3$) δ 166.0, 143.6, 142.1, 140.9, 139.9, 139.8, 135.5, 130.7, 129.6, 128.9, 127.33, 127.28, 127.1, 126.5, 126.3, 124.6, 80.8, 54.1, 52.4, 28.0, 19.7; HRMS *m/z* Calcd. for $C_{28}H_{32}NO_2$ $(M+H^+)$: 414.2428; Found: 414.2434.

Compound 3k

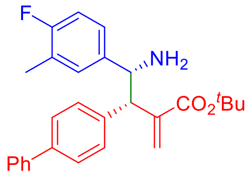

3k

Yellow solid; M.p. 70-76°C; ^{1}H NMR (400 MHz, $CDCl_3$) δ 7.65-7.55 (m, 4H), 7.52-7.41 (m, 4H), 7.35 (t, *J* = 7.2 Hz, 1H), 7.22 (d, *J* = 7.2 Hz, 1H), 7.19-7.13 (m, 1H), 6.94 (t, *J* = 8.8 Hz, 1H), 6.09 (s, 1H), 5.62 (s, 1H), 4.54 (d, *J* = 10.0 Hz, 1H), 4.21 (d, *J* = 10.0 Hz, 1H), 2.27 (s, 3H), 1.51 (brs, 2H), 1.34 (s, 9H); ^{13}C NMR (100 MHz, $CDCl_3$) δ 165.8, 160.6 (d, J_{C-F} = 242.3 Hz), 143.3, 140.9, 139.9 (d, J_{C-F} = 5.4 Hz), 139.3, 130.7 (d, J_{C-F} = 5.1 Hz), 129.4, 128.9, 127.39, 127.35, 127.1, 126.54, 126.47, 125.4, 124.7 (d, J_{C-F} = 17.3 Hz), 114.8 (d, J_{C-F} = 22.1 Hz), 80.9, 58.3, 54.2, 28.0, 14.7 (d, J_{C-F} = 3.5 Hz); ^{19}F NMR (376 MHz, $CDCl_3$) δ -119.8; HRMS *m/z* Calcd. for $C_{28}H_{31}FNO_2$ $(M+H^+)$: 432.2333; Found: 432.2335.

Compound 3l

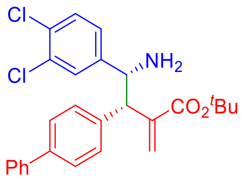

3l

Yellow solid; M.p. 95-98°C; ^{1}H NMR (400 MHz, $CDCl_3$) δ 7.62-7.56 (m, 4H), 7.50 (d, *J* = 2.0 Hz, 1H), 7.47-7.41 (m, 4H), 7.39 (d, *J* = 8.4 Hz, 1H), 7.35 (t, *J* = 7.6 Hz, 1H), 7.25 (dd, *J* = 8.4, 2.0 Hz, 1H), 6.10 (s, 1H), 5.61 (s, 1H), 4.59 (d, *J* = 10.0 Hz, 1H), 4.14 (d, *J* = 9.6 Hz, 1H), 1.59 (brs, 2H), 1.34 (s, 9H); ^{13}C NMR (100 MHz, $CDCl_3$) δ 165.7, 144.3, 142.9, 140.7, 140.1, 139.2, 132.4, 131.2, 130.4, 129.9, 129.3, 128.9, 127.5, 127.4, 127.2, 127.1, 125.9, 81.2, 58.1, 54.4, 28.0; HRMS *m/z* Calcd. for $C_{27}H_{28}Cl_2NO_2$ $(M+H^+)$: 468.1492; Found: 468.1493.

Compound 3m

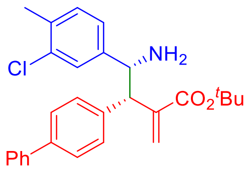

3m

Yellow solid; M.p. 91-94°C; ^{1}H NMR (400 MHz, $CDCl_3$) δ 7.63-7.56 (m, 4H), 7.50-7.41 (m, 4H), 7.40-7.32 (m, 2H), 7.21-7.15 (m, 2H), 6.11 (s, 1H), 5.63 (s, 1H), 4.54 (d, *J* = 9.6 Hz, 1H), 4.20 (d, *J* = 10.0 Hz, 1H), 2.35 (s, 3H), 1.53 (brs, 2H), 1.35 (s, 9H); ^{13}C NMR (100 MHz, $CDCl_3$) δ 165.8, 143.3, 143.2, 140.8, 139.9, 139.6, 135.0, 134.3, 131.0, 129.4, 128.9, 128.4, 127.41, 127.36, 127.1, 125.9, 125.6, 81.0, 58.3, 54.1, 28.0, 19.8; HRMS *m/z* Calcd. for $C_{28}H_{31}ClNO_2$ $(M+H^+)$: 448.2038; Found: 448.2036.

Compound 3n

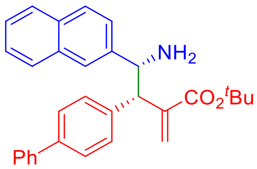

3n

Yellow solid; M.p. 73-77°C; ^{1}H NMR (400 MHz, $CDCl_3$) δ 7.86-7.78 (m, 4H), 7.66-7.57 (m, 5H), 7.52 (d, *J* = 8.0 Hz, 2H), 7.49-7.43 (m, 4H), 7.36 (t, *J* = 7.2 Hz, 1H), 6.07 (s, 1H), 5.70 (s, 1H), 4.78 (d, *J* = 10.0 Hz, 1H), 4.38 (d, *J* = 10.0 Hz, 1H), 1.67 (brs,

2H), 1.26 (s, 9H); ^{13}C NMR (100 MHz, $CDCl_3$) δ 165.9, 143.3, 141.4, 140.9, 139.9, 139.8, 133.5, 133.0, 129.5, 128.9, 128.3, 128.0, 127.7, 127.39, 127.35, 127.1, 126.7, 126.1, 125.8, 125.6, 125.5, 80.8, 59.1, 54.0, 27.9; HRMS *m/z* Calcd. for $C_{31}H_{32}NO_2$ ($M+H^+$): 450.2428; Found: 450.2429.

Compound 3o

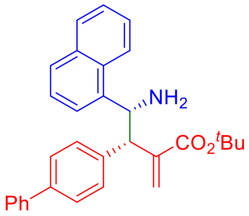

3o

Yellow solid; M.p. 50-53°C; ^{1}H NMR (400 MHz, $CDCl_3$) δ 8.35 (d, *J* = 8.4 Hz, 1H), 7.91 (d, *J* = 8.0 Hz, 1H), 7.79 (d, *J* = 7.6 Hz, 1H), 7.66-7.57 (m, 5H), 7.56-7.40 (m, 7H), 7.36 (t, *J* = 7.6 Hz, 1H), 6.11 (s, 1H), 5.73 (s, 1H), 5.39 (d, *J* = 8.4 Hz, 1H), 4.61 (d, *J* = 8.8 Hz, 1H), 1.62 (brs, 2H), 1.30 (s, 9H); ^{13}C NMR (100 MHz, $CDCl_3$) δ 166.1, 143.3, 140.9, 139.84, 139.79, 139.4, 134.1, 131.5, 129.8, 129.2, 128.9, 127.8, 127.3, 127.14, 127.11, 126.4, 125.6, 124.8, 124.1, 123.0, 80.9, 53.2, 52.3, 27.9; HRMS *m/z* Calcd. for $C_{31}H_{32}NO_2$ ($M+H^+$): 450.2428; Found: 450.2435.

Compound 3p

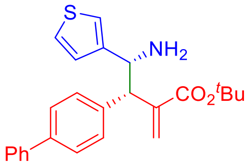

3p

Yellow oil; ^{1}H NMR (400 MHz, $CDCl_3$) δ 7.61-7.58 (m, 2H), 7.58-7.55 (m, 2H), 7.46-7.41 (m, 4H), 7.36-7.31 (m, 1H), 7.28-7.26 (m, 1H), 7.16-7.12 (m, 2H), 6.13 (s, 1H), 5.67 (s, 1H), 4.73 (d, *J* = 9.6 Hz, 1H), 4.20 (d, *J* = 9.6 Hz, 1H), 1.54 (brs, 2H), 1.35 (s, 9H); ^{13}C NMR (100 MHz, $CDCl_3$) δ 166.0, 145.4, 143.4, 140.9, 139.9, 139.7, 129.4, 128.9, 127.4, 127.1, 126.8, 125.7, 125.1, 121.7, 80.9, 54.5, 54.3, 28.1; HRMS *m/z* Calcd. for $C_{25}H_{28}NO_2S$ ($M+H^+$): 406.1835; Found: 406.1839.

Compound 3q

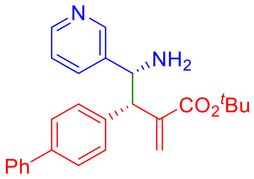

3q

Yellow solid; M.p. 71-75°C; ^{1}H NMR (400 MHz, $CDCl_3$) δ 8.60 (s, 1H), 8.49 (d, *J* = 4.4 Hz, 1H), 7.77 (d, *J* = 8.0 Hz, 1H), 7.63-7.56 (m, 4H), 7.50-7.41 (m, 4H), 7.34 (t, *J* = 7.2 Hz, 1H), 7.28-7.23 (m, 1H), 6.10 (s, 1H), 5.64 (s, 1H), 4.65 (d, *J* = 9.6 Hz, 1H), 4.23 (d, *J* = 9.6 Hz, 1H), 1.73 (s, 2H), 1.31 (s, 9H); ^{13}C NMR (100 MHz, $CDCl_3$) δ 165.6, 149.7, 148.9, 142.9, 140.7, 140.1, 139.24, 139.21, 135.1, 129.3, 128.9, 127.5, 127.4, 127.1, 125.8, 123.6, 81.1, 56.7, 54.0, 28.0; HRMS *m/z* Calcd. for $C_{26}H_{29}N_2O_2$ $(M+H^+)$: 401.2224; Found: 401.2226.

Compound 3r

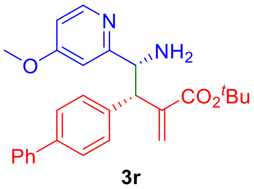

3r

Yellow solid; M.p. 77-81°C; ^{1}H NMR (400 MHz, $CDCl_3$) δ 8.36 (d, *J* = 6.0 Hz, 1H), 7.60-7.54 (m, 4H), 7.45-7.39 (m, 4H), 7.32 (t, *J* = 7.6 Hz, 1H), 6.84 (d, *J* = 2.0 Hz, 1H), 6.67 (dd, *J* = 5.6, 2.4 Hz, 1H), 6.05 (s, 1H), 5.67 (s, 1H), 4.63 (d, *J* = 9.6 Hz, 1H), 4.34 (d, *J* = 9.6 Hz, 1H), 3.81 (s, 3H), 1.33 (s, 9H); ^{13}C NMR (100 MHz, $CDCl_3$) δ 166.1, 165.9, 164.2, 150.4, 143.1, 140.9, 139.8, 139.5, 129.5, 128.8, 127.3, 127.1, 125.4, 108.7, 108.5, 80.8, 59.8, 55.2, 54.0, 28.0; HRMS *m/z* Calcd. for $C_{27}H_{31}N_2O_3$ $(M+H^+)$: 431.2329; Found: 431.2332.

Compound 3s

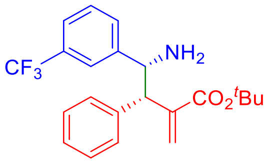

3s

Yellow oil; ^{1}H NMR (400 MHz, $CDCl_3$) δ 7.62 (s, 1H), 7.58 (d, *J* = 8.0 Hz, 1H), 7.50 (d, *J* = 7.6 Hz, 1H), 7.45-7.37 (m, 3H), 7.37-7.31 (m, 2H), 7.29-7.23 (m, 1H), 6.06 (s, 1H), 5.58 (s, 1H), 4.66 (d, *J* = 10.0 Hz, 1H), 4.18 (d, *J* = 9.6 Hz, 1H), 1.53 (s, 2H), 1.29

(s, 9H); ^{13}C NMR (100 MHz, $CDCl_3$) δ 165.7, 145.0, 143.1, 140.2, 131.2 (q, $J_{C\text{-}F}$ = 1.0 Hz), 130.6 (q, $J_{C\text{-}F}$ = 31.9 Hz), 128.95, 128.87, 128.8, 127.3, 125.7, 124.7 (q, $J_{C\text{-}F}$ = 3.8 Hz), 124.29 (q, $J_{C\text{-}F}$ = 270.7 Hz), 124.25 (q, $J_{C\text{-}F}$ = 3.7 Hz), 81.0, 58.7, 54.6, 27.9; ^{19}F NMR (376 MHz, CDCl3) δ -62.5; HRMS *m/z* Calcd. for $C_{22}H_{25}F_3NO_2$ $(M+H^+)$: 392.1832; Found: 392.1836.

Compound 3t

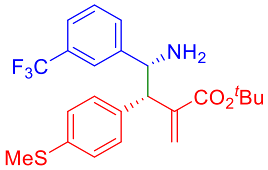

3t

Yellow oil; ^1H NMR (400 MHz, $CDCl_3$) δ 7.61 (s, 1H), 7.56 (d, *J* = 7.6 Hz, 1H), 7.49 (d, *J* = 7.6 Hz, 1H), 7.41 (t, *J* = 7.6 Hz, 1H), 7.31 (d, *J* = 8.0 Hz, 2H), 7.23 (d, *J* = 8.0 Hz, 2H), 6.04 (s, 1H), 5.56 (s, 1H), 4.63 (d, *J* = 9.6 Hz, 1H), 4.12 (d, *J* = 9.6 Hz, 1H), 2.47 (s, 3H), 1.53 (brs, 2H), 1.30 (s, 9H); ^{13}C NMR (100 MHz, $CDCl_3$) δ 165.4, 144.8, 142.8, 137.1, 136.8, 131.0 (q, $J_{C\text{-}F}$ = 0.9 Hz), 130.5 (q, $J_{C\text{-}F}$ = 31.8 Hz), 129.3, 128.7, 126.8, 125.5, 124.5 (q, $J_{C\text{-}F}$ = 3.9 Hz), 124.10 (q, $J_{C\text{-}F}$ = 270.6 Hz), 124.09 (q, $J_{C\text{-}F}$ = 3.8 Hz), 80.9, 58.4, 54.1, 27.7, 15.8; ^{19}F NMR (376 MHz, $CDCl_3$) δ -62.5; HRMS *m/z* Calcd. for $C_{23}H_{27}F_3NO_2S$ $(M+H^+)$: 438.1709; Found: 438.1707.

Compound 3u

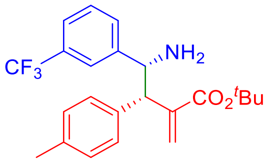

3u

Yellow oil; ^1H NMR (400 MHz, $CDCl_3$) δ 7.63 (s, 1H), 7.58 (d, *J* = 7.6 Hz, 1H), 7.49 (d, *J* = 8.0 Hz, 1H), 7.42 (t, *J* = 7.6 Hz, 1H), 7.29 (d, *J* = 8.0 Hz, 2H), 7.16 (d, *J* = 8.0 Hz, 2H), 6.04 (s, 1H), 5.57 (s, 1H), 4.63 (d, *J* = 10.0 Hz, 1H), 4.14 (d, *J* = 10.0 Hz, 1H), 2.33 (s, 3H), 1.54 (brs, 2H), 1.30 (s, 9H); ^{13}C NMR (100 MHz, $CDCl_3$) δ 165.7, 145.0, 143.3, 137.2, 136.8, 131.3 (q, $J_{C\text{-}F}$ = 1.0 Hz), 130.6 (q, $J_{C\text{-}F}$ = 31.8 Hz), 129.5, 128.84, 128.77, 125.5, 124.7 (q, $J_{C\text{-}F}$ = 3.9 Hz), 124.3 (q, $J_{C\text{-}F}$ = 270.6 Hz), 124.2 (q, $J_{C\text{-}F}$ = 3.8 Hz), 80.9, 58.8, 54.3, 27.9, 21.1; ^{19}F NMR (376 MHz, $CDCl_3$) δ -62.5; HRMS *m/z* Calcd. for $C_{23}H_{27}F_3NO_2$ $(M+H^+)$: 406.1988; Found: 406.1994.

Compound 3v

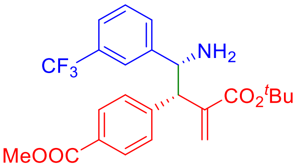

3v

Yellow solid; ^{1}H NMR (400 MHz, $CDCl_3$) δ 8.01 (d, *J* = 8.0 Hz, 2H), 7.61 (s, 1H), 7.55 (d, *J* = 7.6 Hz, 1H), 7.51-7.41 (m, 4H), 6.09 (s, 1H), 5.60 (s, 1H), 4.70 (d, *J* = 9.6 Hz, 1H), 4.22 (d, *J* = 9.2 Hz, 1H), 3.90 (s, 3H), 1.29 (s, 9H); ^{13}C NMR (100 MHz, $CDCl_3$) δ 167.0, 165.4, 145.6, 144.8, 142.5, 131.1, 130.7 (q, J_{C-F} = 31.9 Hz), 130.0, 129.13, 129.05, 129.0, 126.3, 124.6 (q, J_{C-F} = 3.7 Hz), 124.4 (q, J_{C-F} = 3.7 Hz), 124.2 (q, J_{C-F} = 270.8 Hz), 81.3, 58.4, 54.8, 52.2, 27.9; ^{19}F NMR (376 MHz, $CDCl_3$) δ -62.5; HRMS *m/z* Calcd. for $C_{24}H_{26}F_3NO_4$ (M + H^+): 450.1887; Found: 450.1888.

Compound 3w

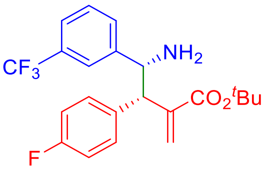

3w

Yellow oil; ^{1}H NMR (400 MHz, $CDCl_3$) δ 7.60 (s, 1H), 7.55 (d, *J* = 8.0 Hz, 1H), 7.50 (d, *J* = 7.6 Hz, 1H), 7.42 (t, *J* = 8.0 Hz, 1H), 7.35 (dd, *J* = 8.8, 5.6 Hz, 2H), 7.03 (t, *J* = 8.4 Hz, 2H), 6.06 (s, 1H), 5.57 (s, 1H), 4.64 (d, *J* = 9.6 Hz, 1H), 4.14 (d, *J* = 9.2 Hz, 1H), 1.52 (brs, 2H), 1.30 (s, 9H); ^{13}C NMR (100 MHz, $CDCl_3$) δ 165.6, 162.1 (d, J_{C-F} = 244.1 Hz), 144.9, 143.0, 135.8 (d, J_{C-F} = 3.2 Hz), 131.1 (q, J_{C-F} = 1.0 Hz), 130.7 (q, J_{C-F} = 31.9 Hz), 130.5 (d, J_{C-F} = 7.9 Hz), 128.9, 125.8, 124.6 (q, J_{C-F} = 3.9 Hz), 124.32 (q, J_{C-F} = 3.7 Hz), 124.26 (q, J_{C-F} = 270.7 Hz), 115.6 (d, J_{C-F} = 21.1 Hz), 81.2, 58.5, 54.1, 27.9; ^{19}F NMR (376 MHz, $CDCl_3$) δ -62.5, -115.6; HRMS *m/z* Calcd. for $C_{22}H_{24}F_4NO_2$ (M+H^+):410.1738; Found: 410.1738.

Compound 3x

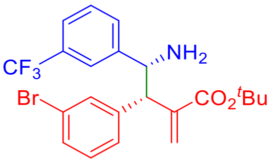

3x

Yellow oil; [1] H NMR (400 MHz, $CDCl_3$) δ 7.61 (s, 1H), 7.57-7.54 (m, 2H), 7.50 (d, *J* = 8.0 Hz, 1H), 7.45-7.39 (m, 2H), 7.35-7.32 (m, 1H), 7.22 (t, *J* = 8.0 Hz, 1H), 6.07 (s, 1H), 5.57 (s, 1H), 4.65 (d, *J* =9.6 Hz, 1H), 4.12 (d, *J* = 9.6 Hz, 1H), 1.43 (brs, 2H), 1.32 (s, 9H); ^{13}C NMR (100 MHz, $CDCl_3$) δ 165.4, 144.7, 142.7, 142.5, 131.9, 131.1 (q, J_{C-F} = 1.0 Hz), 130.8 (q, J_{C-F} = 31.8 Hz), 130.4, 130.3, 129.0, 127.8, 126.3, 124.6 (q, J_{C-F} = 3.6 Hz), 124.4 (q, J_{C-F} = 3.6 Hz), 124.3 (q, J_{C-F} = 270.6 Hz), 122.9, 81.3, 58.4, 54.6, 27.9; ^{19}F NMR (376 MHz, $CDCl_3$) δ -62.5; HRMS *m/z* Calcd. for $C_{22}H_{24}BrF_3NO_2$ (M+H$^+$): 470.0937; Found: 470.0939.

Compound 3y

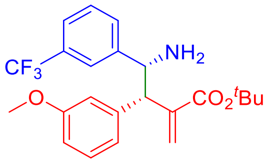

3y

Yellow oil; ^{1}H NMR (400 MHz, $CDCl_3$) δ 7.63 (s, 1H), 7.58 (d, *J* = 7.6 Hz, 1H), 7.49 (d, *J* = 7.6 Hz, 1H), 7.42 (t, *J* = 7.6 Hz, 1H), 7.30-7.23 (m, 1H), 7.00 (d, *J* = 7.6 Hz, 1H), 6.94 (s, 1H), 6.81 (dd, *J* = 8.0, 2.4 Hz, 1H), 6.05 (s, 1H), 5.58 (s, 1H), 4.63 (d, *J* = 10.0 Hz, 1H), 4.16 (d, *J* = 9.6 Hz, 1H), 3.80 (s, 3H), 1.63 (brs, 2H), 1.30 (s, 9H); ^{13}C NMR (100 MHz, $CDCl_3$) δ 165.7, 159.9, 144.8, 143.0, 141.9, 131.3 (q, J_{C-F} = 1.0 Hz), 130.6 (q, J_{C-F} = 31.8 Hz), 129.8, 128.9, 125.8, 124.8 (q, J_{C-F} = 3.7 Hz), 124.30 (q, J_{C-F} = 270.7 Hz), 124.28 (q, J_{C-F} = 3.8 Hz), 114.7, 112.6, 81.0, 58.8, 55.3, 54.6, 27.9; ^{19}F NMR (376 MHz, $CDCl_3$) δ -62.5; HRMS *m/z* Calcd. for $C_{23}H_{27}F_3NO_3$ (M+H$^+$): 422.1938; Found: 422.1936.

Compound 3z

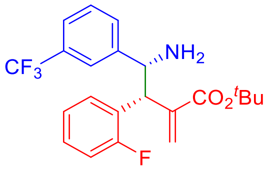

3z

Yellow oil; ^{1}H NMR (400 MHz, $CDCl_3$) δ 7.61 (s, 1H), 7.58 (d, *J* = 7.6 Hz, 1H), 7.56-7.47 (m, 2H), 7.43 (t, *J* = 7.6 Hz, 1H), 7.28-7.21 (m, 1H), 7.16 (t, *J* = 7.6 Hz, 1H), 7.04 (t, *J* = 9.6 Hz, 1H), 6.12 (s, 1H), 5.62 (s, 1H), 4.71 (d, *J* = 10.0 Hz, 1H), 4.53 (d, *J* = 9.6 Hz, 1H), 1.54 (brs, 2H), 1.30 (s, 9H); ^{13}C NMR (100 MHz, $CDCl_3$) δ 165.4, 161.5 (d, J_{C-F} = 244.8 Hz), 145.0, 141.9, 131.0 (q, J_{C-F} = 1.0 Hz), 130.7 (q, J_{C-F} = 31.9 Hz), 129.7

(d, $J_{C\text{-}F}$ = 4.0 Hz), 129.0, 128.7 (d, $J_{C\text{-}F}$ = 8.3 Hz), 127.3 (d, $J_{C\text{-}F}$ = 14.0 Hz), 126.8, 124.5 (q, $J_{C\text{-}F}$ = 3.8 Hz), 124.4 (d, $J_{C\text{-}F}$ = 3.7 Hz), 124.31 (q, $J_{C\text{-}F}$ = 3.9 Hz), 124.28 (q, $J_{C\text{-}F}$ = 270.7 Hz), 115.9 (d, $J_{C\text{-}F}$ = 22.8 Hz), 81.1, 57.8 (d, $J_{C\text{-}F}$ = 1.4 Hz), 47.4, 27.9; ^{19}F NMR (376 MHz, $CDCl_3$) δ -62.5, -116.2; HRMS *m/z* Calcd. for $C_{22}H_{24}F_4NO_2$ (M+H$^+$):410.1738; Found: 410.1739.

Compound 3aa

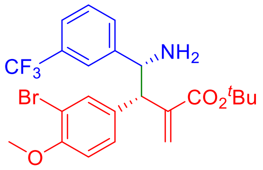

3aa

Yellow solid; M.p. 57-60 °C; ^{1}H NMR (400 MHz, $CDCl_3$) δ 7.60 (s, 1H), 7.58 (d, *J* = 2.4 Hz, 1H), 7.55 (d, *J* = 7.6 Hz, 1H), 7.49 (d, *J* = 7.6 Hz, 1H), 7.42 (t, *J* = 7.6 Hz, 1H), 7.30 (dd, *J* = 8.8, 2.4 Hz, 1H), 6.87 (d, *J* = 8.4 Hz, 1H), 6.03 (s, 1H), 5.54 (s, 1H), 4.61 (d, *J* = 9.6 Hz, 1H), 4.06 (d, *J* =10.0 Hz, 1H), 3.88 (s, 3H), 1.60 (brs, 2H), 1.32 (s, 9H); ^{13}C NMR (100 MHz, $CDCl_3$) δ 165.5, 155.1, 144.8, 142.8, 133.7, 133.5, 131.1, 130.7 (q, $J_{C\text{-}F}$ = 32.0 Hz), 129.2, 128.9, 125.9, 124.6 (q, $J_{C\text{-}F}$ = 3.6 Hz), 124.3 (q, $J_{C\text{-}F}$ = 3.7 Hz), 124.2 (q, $J_{C\text{-}F}$ = 270.7 Hz), 112.0, 111.9, 81.2, 58.5, 56.4, 53.9, 27.9; ^{19}F NMR (376 MHz, $CDCl_3$) δ -62.5; HRMS *m/z* Calcd. for $C_{23}H_{26}BrF_3NO_3$ (M+H$^+$):500.1043; Found: 500.1041.

Compound 3ab

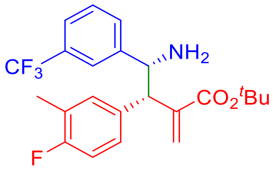

3ab

Yellow oil; ^{1}H NMR (400 MHz, $CDCl_3$) δ 7.60 (s, 1H), 7.56 (d, *J* = 8.0 Hz, 1H), 7.49 (d, *J* = 7.6 Hz, 1H), 7.42 (t, *J* = 7.6 Hz, 1H), 7.21-7.14 (m, 2H), 7.01-6.92 (m, 1H), 6.04 (s, 1H), 5.56 (s, 1H), 4.61 (d, *J* = 10.0 Hz, 1H), 4.10 (d, *J* = 9.6 Hz, 1H), 2.27 (s, 3H), 1.45 (brs, 2H), 1.31 (s, 9H); ^{13}C NMR (100 MHz, $CDCl_3$) δ 165.7, 160.6 (d, $J_{C\text{-}F}$ = 242.9 Hz), 145.0, 143.1, 135.5 (d, $J_{C\text{-}F}$ = 3.6 Hz), 132.1 (d, $J_{C\text{-}F}$ = 5.1 Hz), 131.2 (q, $J_{C\text{-}F}$ = 1.2 Hz), 130.7 (q, $J_{C\text{-}F}$ = 31.9 Hz), 128.9, 127.5 (d, $J_{C\text{-}F}$ = 7.9 Hz), 125.6, 125.1 (d, $J_{C\text{-}F}$ = 17.2 Hz), 124.7 (q, $J_{C\text{-}F}$ = 3.8 Hz), 124.283 (q, $J_{C\text{-}F}$ = 270.6 Hz), 124.280 (q, $J_{C\text{-}F}$ = 3.8 Hz), 115.2 (d, $J_{C\text{-}F}$ = 22.1 Hz), 81.1, 58.6, 54.0, 27.9, 14.8 (d, $J_{C\text{-}F}$ = 3.6 Hz); ^{19}F

NMR (376 MHz, $CDCl_3$) δ -62.5, -120.0; HRMS *m/z* Calcd. for $C_{23}H_{26}F_4NO_2$ $(M+H^+)$: 424.1894; Found: 424.1894.

Compound 3ac

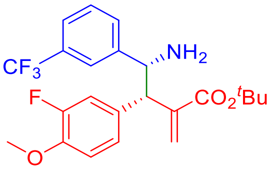

3ac

Yellow oil; ^{1}H NMR (400 MHz, $CDCl_3$) δ 7.60 (s, 1H), 7.55 (d, *J* = 7.6 Hz, 1H), 7.49 (d, *J* = 8.0 Hz, 1H), 7.41 (t, *J* = 7.6 Hz, 1H), 7.15 (dd, *J* = 12.4, 2.4 Hz, 1H), 7.09 (d, *J* = 8.8 Hz, 1H), 6.92 (t, *J* = 8.8 Hz, 1H), 6.04 (s, 1H), 5.55 (s, 1H), 4.60 (d, *J* = 9.6 Hz, 1H), 4.08 (d, *J* = 9.6 Hz, 1H), 3.88 (s, 3H), 1.57 (brs, 2H), 1.32 (s, 9H); ^{13}C NMR (100 MHz, $CDCl_3$) δ 165.6, 152.4 (d, J_{C-F} = 244.5 Hz), 146.8 (d, J_{C-F} = 10.5 Hz), 144.9, 142.9, 133.1 (d, J_{C-F} = 5.7 Hz), 131.1 (q, J_{C-F} = 1.1 Hz), 130.7 (q, J_{C-F} = 31.9 Hz), 128.9, 125.8, 124.9 (d, J_{C-F} = 3.5 Hz), 124.6 (q, J_{C-F} = 3.7 Hz), 124.31 (q, J_{C-F} = 3.6 Hz), 124.27 (q, J_{C-F} = 270.8 Hz), 116.4 (d, J_{C-F} = 18.6 Hz), 113.5 (d, J_{C-F} = 2.2 Hz), 81.2, 58.5, 56.4, 54.0, 27.9; ^{19}F NMR (376 MHz, $CDCl_3$) δ -62.5, -134.6; HRMS *m/z* Calcd. for $C_{23}H_{26}F_4NO_3$ $(M+H^+)$: 440.1843; Found: 440.1846.

Compound 3ad

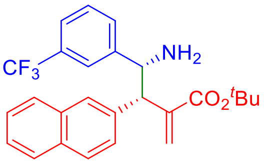

3ad

Yellow solid; M.p. 118-121°C; ^{1}H NMR (400 MHz, $CDCl_3$) δ 7.89 (s, 1H), 7.87-7.79 (m, 3H), 7.69 (s, 1H), 7.63 (d, *J* = 7.6 Hz, 1H), 7.55-7.42 (m, 5H), 6.11 (s, 1H), 5.66 (s, 1H), 4.78 (d, *J* = 10.0 Hz, 1H), 4.37 (d, *J* = 9.6 Hz, 1H), 1.58 (brs, 2H), 1.30 (s, 9H); ^{13}C NMR (100 MHz, $CDCl_3$) δ 165.7, 144.9, 143.0, 137.7, 133.6, 132.7, 131.3 (q, J_{C-F} = 1.0 Hz), 130.7 (q, J_{C-F} = 31.9 Hz), 128.9, 128.6, 127.9, 127.8, 127.7, 126.9, 126.3, 126.0, 125.9, 124.8 (q, J_{C-F} = 3.7 Hz), 124.32 (q, J_{C-F} = 3.7 Hz), 124.31 (q, J_{C-F} = 270.6 Hz), 81.1, 58.7, 54.7, 27.9; ^{19}F NMR (376 MHz, $CDCl_3$) δ -62.5; HRMS *m/z* Calcd. for $C_{26}H_{27}F_3NO_2$ $(M+H^+)$: 442.1988; Found: 442.1988.

Compound 3ae

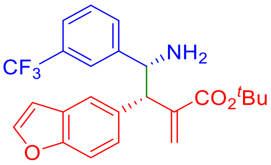

3ae

Yellow oil; ^{1}H NMR (400 MHz, $CDCl_3$) δ 7.68-7.57 (m, 4H), 7.53-7.39 (m, 3H), 7.32 (dd, *J* = 8.4, 1.6 Hz, 1H), 6.75 (d, *J* = 1.2 Hz, 1H), 6.07 (s, 1H), 5.62 (s, 1H), 4.69 (d, *J* = 9.6 Hz, 1H), 4.28 (d, *J* = 9.6 Hz, 1H), 1.58 (brs, 2H), 1.30 (s, 9H); ^{13}C NMR (100 MHz, $CDCl_3$) δ 165.8, 154.3, 145.6, 145.1, 143.5, 134.6, 131.2 (q, $J_{C\text{-}F}$ = 0.9 Hz), 130.6 (q, $J_{C\text{-}F}$ = 31.8 Hz), 128.9, 127.9, 125.5, 125.2, 124.7 (q, $J_{C\text{-}F}$ = 3.7 Hz), 124.3 (q, $J_{C\text{-}F}$ = 270.7 Hz), 124.2 (q, $J_{C\text{-}F}$ = 3.9 Hz), 121.4, 111.6, 106.7, 81.0, 59.0, 54.5, 27.9; ^{19}F NMR (376 MHz, $CDCl_3$) δ -62.5; HRMS *m/z* Calcd. for $C_{24}H_{25}F_3NO_3$ $(M+H^+)$: 432.1781; Found: 432.1781.

Compound 3af

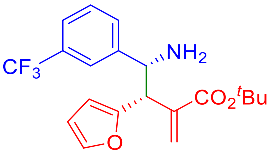

3af

Yellow oil; ^{1}H NMR (400 MHz, $CDCl_3$) δ 7.54 (s, 1H), 7.50 (d, *J* = 8.0 Hz, 1H), 7.46 (d, *J* = 7.6 Hz, 1H), 7.40-7.36 (m, 2H), 6.34-6.31 (m, 1H), 6.21-6.18 (m, 2H), 5.74 (s, 1H), 4.53 (d, *J* = 8.4 Hz, 1H), 4.33 (d, *J* = 8.4 Hz, 1H), 1.34 (s, 9H); ^{13}C NMR (100 MHz, $CDCl_3$) δ 165.3, 153.6, 144.2, 142.1, 140.7, 131.0 (q, $J_{C\text{-}F}$ = 1.1 Hz), 130.5 (q, $J_{C\text{-}F}$ = 32.0 Hz), 128.7, 127.2, 124.5 (q, $J_{C\text{-}F}$ = 3.8 Hz), 124.3 (q, $J_{C\text{-}F}$ = 270.9 Hz), 124.2 (q, $J_{C\text{-}F}$ = 3.7 Hz), 110.5, 108.3, 81.2, 58.9, 48.1, 27.9; ^{19}F NMR (376 MHz, $CDCl_3$) δ -62.5; HRMS *m/z* Calcd. for $C_{20}H_{23}F_3NO_3$ $(M+H^+)$: 382.1625; Found: 382.1625.

Compound 3ag

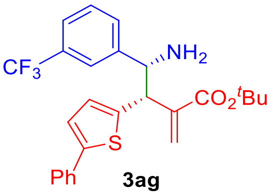

3ag

Yellow oil; ^{1}H NMR (400 MHz, $CDCl_3$) δ 7.64 (s, 1H), 7.62-7.54 (m, 3H), 7.50 (d, *J* = 7.6 Hz, 1H), 7.42 (d, *J* = 7.6 Hz, 1H), 7.37 (t, *J* = 7.6 Hz, 2H), 7.29-7.25 (m, 1H), 7.18

(d, J = 3.6 Hz, 1H), 7.01 (d, J = 3.6 Hz, 1H), 6.13 (s, 1H), 5.67 (s, 1H), 4.64 (d, J = 9.2 Hz, 1H), 4.42 (d, J = 8.8 Hz, 1H), 1.75 (brs, 2H), 1.39 (s, 9H); ^{13}C NMR (100 MHz, $CDCl_3$) δ 165.4, 144.4, 143.8, 142.8, 142.5, 134.4, 131.1 (q, J_{C-F} = 0.9 Hz), 130.6 (q, J_{C-F} = 31.9 Hz), 129.0, 128.8, 127.7, 127.5, 126.8, 125.7, 124.6 (q, J_{C-F} = 3.7 Hz), 124.32 (q, J_{C-F} = 3.8 Hz), 124.27 (q, J_{C-F} = 270.6 Hz), 122.7, 81.3, 59.8, 51.4, 28.0; ^{19}F NMR (376 MHz, $CDCl_3$) δ -62.5; HRMS *m/z* Calcd. for $C_{26}H_{27}F_3NO_2S$ (M+H$^+$):474.1709; Found: 474.1709.

Compound 3ah

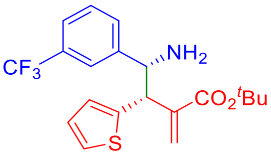

3ah

Yellow oil; ^{1}H NMR (400 MHz, $CDCl_3$) δ 7.61 (s, 1H), 7.56 (d, J = 7.6 Hz, 1H), 7.48 (d, J = 7.6 Hz, 1H), 7.40 (t, J = 7.6 Hz, 1H), 7.22 (d, J = 5.2 Hz, 1H), 7.04 (d, J = 3.6 Hz, 1H), 6.97 (dd, J = 5.2, 3.6 Hz, 1H), 6.09 (s, 1H), 5.63 (s, 1H), 4.61 (d, J = 9.2 Hz, 1H), 4.45 (d, J = 9.2 Hz, 1H), 1.83 (brs, 2H), 1.37 (s, 9H); ^{13}C NMR (100 MHz, $CDCl_3$) δ 165.4, 144.3, 143.1, 142.5, 131.1 (q, J_{C-F} = 1.0Hz), 130.6 (q, J_{C-F} = 31.9 Hz), 128.8, 126.85, 126.77, 126.70, 124.9, 124.6 (q, J_{C-F} = 3.9 Hz), 124.30 (q, J_{C-F} = 3.9 Hz), 124.26 (q, J_{C-F} = 270.7 Hz), 81.3, 59.9, 51.0, 28.0; ^{19}F NMR (376 MHz, $CDCl_3$) δ -62.5; HRMS *m/z* Calcd. for $C_{20}H_{23}F_3NO_2S$ (M+H$^+$): 398.1396; Found: 398.1395.

Compound 3ai

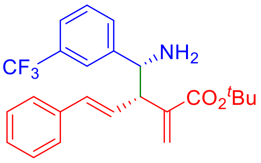

3ai

Yellow oil; ^{1}H NMR (400 MHz, $CDCl_3$) δ 7.60 (s, 1H), 7.54 (d, J = 7.6 Hz, 1H), 7.48 (d, J = 8.0 Hz, 1H), 7.43-7.36 (m, 3H), 7.34-7.29 (m, 2H), 7.26-7.21 (m, 1H), 6.52 (d, J = 7.6 Hz, 1H), 6.45 (dd, J = 16.0, 8.8 Hz, 1H), 6.03 (s, 1H), 5.43 (s, 1H), 4.39 (d, J = 8.4 Hz, 1H), 3.54 (t, J = 8.4 Hz, 1H), 1.65 (brs, 2H), 1.44 (s, 9H); ^{13}C NMR (100 MHz, $CDCl_3$) δ 165.6, 144.9, 142.4, 137.1, 133.6, 131.0 (q, J_{C-F} = 1.1 Hz), 130.5 (q, J_{C-F} = 31.8 Hz), 128.7, 128.4, 127.7, 126.5, 126.2, 124.5 (q, J_{C-F} = 3.8 Hz), 124.3 (q, J_{C-F} =

270.6 Hz), 124.1 (q, J_{C-F} = 3.8 Hz), 81.1, 58.3, 54.9, 28.1; ^{19}F NMR (376 MHz, $CDCl_3$) δ -62.5; HRMS *m/z* Calcd. for $C_{24}H_{27}F_3NO_2$ (M+H$^+$): 418.1988; Found: 418.1991.

Compound 3aj

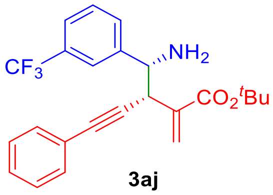

3aj

Yellow oil; ^{1}H NMR (400 MHz, $CDCl_3$) δ 7.76 (s, 1H), 7.68 (d, *J* = 7.6 Hz, 1H), 7.54 (d, *J* = 8.0 Hz, 1H), 7.47 (t, *J* = 7.6 Hz, 1H), 7.37-7.34 (m, 2H), 7.31-7.28 (m, 3H), 6.45 (d, *J* = 1.6 Hz, 1H), 6.11 (s, 1H), 4.30 (d, *J* = 3.6 Hz, 1H), 4.22 (d, *J* = 3.2 Hz, 1H), 1.69 (brs, 2H), 1.53 (s, 9H); ^{13}C NMR (100 MHz, $CDCl_3$) δ 165.1, 144.5, 139.6, 131.7, 130.4, 128.7, 128.5, 128.4, 128.1, 124.4 (q, J_{C-F} = 270.9 Hz), 124.2 (q, J_{C-F} = 3.8 Hz), 123.7 (q, J_{C-F} = 3.8 Hz), 122.9, 87.2, 86.0, 81.8, 56.9, 44.5, 28.2; ^{19}F NMR (376 MHz, $CDCl_3$) δ -62.4; HRMS *m/z* Calcd. for $C_{24}H_{25}F_3NO_2$ (M+H$^+$): 416.1832; Found: 416.1832.

Compound 3ak

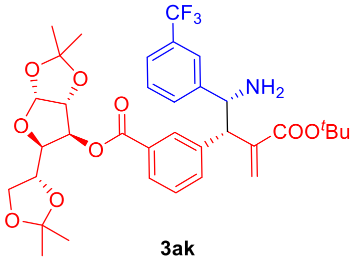

3ak

White solid; M.p. 81-84 °C; ^{1}H NMR (400 MHz, $CDCl_3$) δ 8.05 (s, 1H), 7.90 (d, *J* = 7.6 Hz, 1H), 7.64 (d, *J* = 7.6 Hz, 1H), 7.60-7.53 (m, 2H), 7.49 (d, *J* = 8.0 Hz, 1H), 7.46-7.37 (m, 2H), 6.07 (s, 1H), 5.94 (d, *J* = 4.0 Hz, 1H), 5.59 (s, 1H), 5.48 (d, *J* = 2.8 Hz, 1H), 4.73 (d, *J* = 9.6 Hz, 1H), 4.63 (d, *J* = 3.6 Hz, 1H), 4.40-4.30 (m, 2H), 4.21 (d, *J* = 9.6 Hz, 1H), 4.14-4.05 (m, 2H), 1.55 (s, 3H), 1.40 (s, 3H), 1.31 (s, 3H), 1.30 (s, 9H), 1.25 (s, 3H); ^{13}C NMR (100 MHz, $CDCl_3$) δ 165.35, 165.28, 144.7, 142.5, 141.0, 134.2, 131.1, 130.7 (q, J_{C-F} = 32.0 Hz), 130.2, 130.0, 129.0, 128.5, 126.4, 124.5 (q, J_{C-F} = 3.8 Hz), 124.4 (q, J_{C-F} = 3.7 Hz), 124.2 (q, J_{C-F} = 270.7 Hz), 112.5, 109.5, 105.2, 83.5, 81.3, 80.0, 76.9, 72.7, 67.3, 58.3, 54.8, 27.9, 26.9, 26.8, 26.3, 25.3; ^{19}F NMR (376 MHz, $CDCl_3$) δ -62.5; HRMS *m/z* Calcd. for $C_{35}H_{43}F_3NO_9$ (M+H$^+$):678.2884; Found: 678.2885.

Compound 3al

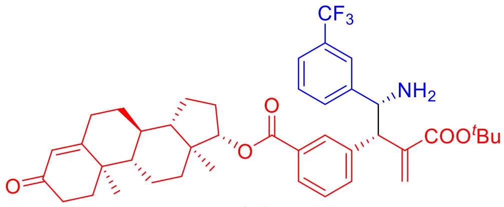

3al

Yellow solid; M.p. 85-90 °C; ^{1}H NMR (400 MHz, $CDCl_3$) δ 8.04 (s, 1H), 7.93 (d, *J* = 7.6 Hz, 1H), 7.64-7.58 (m, 2H), 7.55 (d, *J* = 7.6 Hz, 1H), 7.49 (d, *J* = 7.6 Hz, 1H), 7.45-7.36 (m, 2H), 6.08 (s, 1H), 5.73 (s, 1H), 5.61 (s, 1H), 4.85 (dd, *J* = 8.8, 8.0 Hz, 1H), 4.71 (d, *J* = 9.6 Hz, 1H), 4.23 (d, *J* = 10.0 Hz, 1H), 2.47-2.39 (m, 1H), 2.39-2.34 (m, 1H), 2.33-2.25 (m, 2H), 2.08-1.99 (m, 1H), 1.92-1.83 (m, 2H), 1.77-1.69 (m, 2H), 1.69-1.64 (m, 2H), 1.63-1.56 (m, 3H), 1.48-1.39 (m, 2H), 1.34 (d, *J* = 4.4 Hz, 1H), 1.30 (s, 9H), 1.21 (s, 3H), 1.15-1.10 (m, 1H), 1.09-1.03 (m, 1H), 0.97 (s, 3H); ^{13}C NMR (100 MHz, $CDCl_3$) δ 199.7, 171.1, 166.5, 165.4, 144.8, 142.6, 140.7, 133.7, 131.13, 131.07, 130.7 (q, J_{C-F} = 31.9 Hz), 129.8, 128.9, 128.8, 128.4, 126.3, 124.6 (q, J_{C-F} = 3.7 Hz), 124.3 (q, J_{C-F} = 4.0 Hz), 124.2 (q, J_{C-F} = 270.7 Hz), 124.1, 83.2, 81.2, 58.4, 54.5, 53.8, 50.4, 43.0, 38.7, 36.8, 35.8, 35.5, 34.0, 32.8, 31.6, 27.9, 27.7, 23.7, 20.6, 17.5, 12.4; ^{19}F NMR (376 MHz, $CDCl_3$) δ -62.5; HRMS *m/z* Calcd. for $C_{42}H_{51}F_3NO_5$ $(M+H^+)$: 706.3714; Found: 706.3717.

Compound 3am

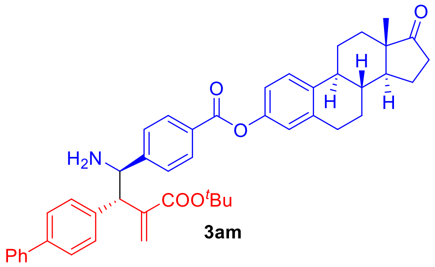

3am

Yellow solid; M.p. 178-180°C; ^{1}H NMR (400 MHz, $CDCl_3$) δ 8.14 (d, *J* = 8.0 Hz, 2H), 7.63-7.56 (m, 4H), 7.53 (d, *J* = 8.4 Hz, 2H), 7.48-7.42 (m, 4H), 7.35 (t, *J* = 7.2 Hz, 2H), 6.98 (dd, *J* = 8.4, 2.4 Hz, 1H), 6.94 (d, *J* = 2.8 Hz, 1H), 6.10 (s, 1H), 5.66 (s, 1H), 4.72 (d, *J* = 9.6 Hz, 1H), 4.25 (d, *J* = 9.2 Hz, 1H), 3.00-2.90 (m, 2H), 2.57-2.41 (m, 2H), 2.36-2.29 (m, 1H), 2.19-2.06 (m, 2H), 2.00-1.95 (m, 1H), 1.67-1.59 (m, 3H), 1.57-1.47 (m, 4H), 1.34 (s, 9H), 0.93 (s, 3H); ^{13}C NMR (100 MHz, $CDCl_3$) δ 221.0, 165.7, 165.4, 150.1, 149.0, 143.0, 140.8, 140.1, 139.2, 138.2, 137.6, 130.4, 129.5, 128.9, 128.8, 128.0, 127.4, 127.1, 126.6, 125.7, 121.8, 119.0, 81.1, 58.6, 54.3, 50.6, 48.1, 44.3, 38.2, 36.0,

31.7, 29.6, 28.0, 26.5, 25.9, 21.7, 14.0; HRMS *m/z* Calcd. for $C_{46}H_{50}NO_5$ $(M+H^+)$: 696.3684; Found: 696.3683.

6.2 Procedure for the Synthesis of α-Methylene-γ-aminobutyrate 3h in Gram-scale

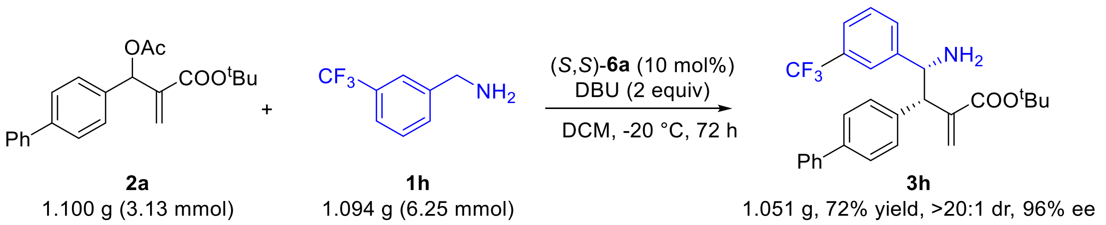

To a 25 mL flask equipped with a magnetic stirrer bar were successively added chiral pyridoxal (*S*,*S*)-**6a** (0.144 g, 0.312 mmol), DBU (0.950 g, 6.250 mmol), DCM (9.35 mL) and arylmethanamine **1h** (1.094 g, 6.250 mmol). The mixture was stirred at -20 °C for 5 min, and a solution of MBH acetate **2a** (1.100 g, 3.130 mmol) in DCM (6.25 mL) was added in 7 portions over 1 h (the first portion: 20 mol% of the solution of **2a**; the rest of **2a** was then divided into 6 portions to be added into the reaction flask). After the reaction mixture was stirred at -20 °C for 72 h, it was allowed to warm up to room temperature and concentrated via rotary evaporator to remove most of the solvent. Then it was dried under vacuum and submitted to ^{1}H NMR analysis to determine the dr values. The crude reaction mixture was purified by column chromatography on silica gel (petroleum ether: ethyl acetate = 3:1) to afford compound (*S*,*R*)-**3h** (1.051 g, 72% yield, >20:1 dr, 96% ee) as a white solid.

7. Synthetic Transformations

7.1 Synthesis of Compound 8h (Fig. 4b).

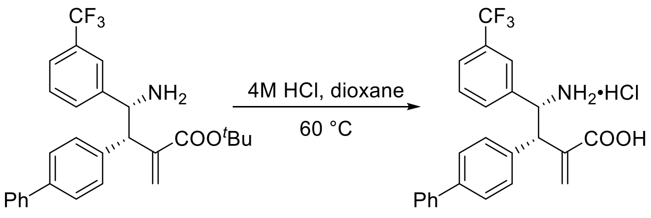

3h
(96% ee)

8h
(95% yield, 96% ee)

To a 5 mL vial were added compound **3h** (96% ee) (0.0397 g, 0.085 mmol) and 4 M HCl in dioxane (1.0 mL). The mixture was stirred at 60 °C for 3 h and then concentrated via rotary evaporator to remove most of the solvent to give crude product **8h** (0.0361 g, 95% yield). The ee value was determined (96% ee) by chiral HPLC analysis after **8h** was converted to the corresponding intramolecular cyclization product **9h**.

8h: White soild; M.p. 160-163 °C ; ^{1}H NMR (400 MHz, CD_3OD) δ 7.99 (s, 1H), 7.93 (d, J = 8.0 Hz, 1H), 7.77-7.64 (m, 8H), 7.45 (t, J = 7.6 Hz, 2H), 7.35 (t, J = 7.2 Hz, 1H), 6.18 (s, 1H), 5.96 (s, 1H), 5.46 (d, J = 12.0 Hz, 1H), 4.74 (d, J = 12.0 Hz, 1H); ^{13}C NMR (100 MHz, CD_3OD) δ 168.8, 142.6, 141.7, 141.4, 138.6, 137.7, 133.3, 132.4 (q, $J_{C\text{-}F}$ =32.4 Hz), 131.3, 130.5, 129.9, 129.0, 128.8, 128.6, 128.0, 127.3 (q, $J_{C\text{-}F}$ =3.8 Hz), 126.5 (q, $J_{C\text{-}F}$ =3.8 Hz), 125.3 (q, $J_{C\text{-}F}$ = 270.0 Hz), 58.3, 52.0; ^{19}F NMR (376 MHz, CD_3OD) δ -64.1; HRMS *m/z* Calcd. for $C_{24}H_{19}F_3NO_2$ ($M+H^+$): 412.1519; Found: 412.1521.

7.2 Synthesis of Compound 9h (Fig. 4b).

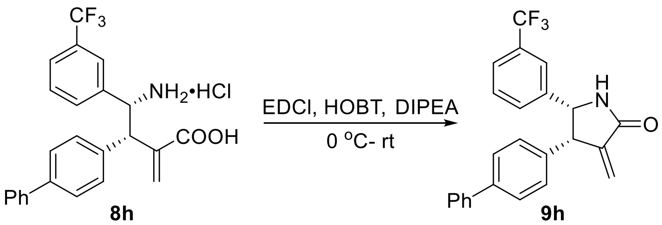

8h
(96% ee)

9h
64% yield, 96% ee

To a 5 mL vial were added compound **8h** (0.0270 g, 0.060 mmol), 1-(3-dimethylaminopropyl)-3-ethylcarbodiimide hydrochloride (EDCl) (0.0167 g, 0.0871 mmol), 1-hydroxybenzotriazole (HOBT) (0.0181 g, 0.134 mmol) and DCM (0.50 mL). Then the reaction mixture was stirred at 0 °C for 5 min, followed by the addition of *N*, *N*-diisopropylethylamine (DIPEA) (0.0217 g, 0.168 mmol) at the same temperature.

The reaction mixture was allowed to return to room temperature and stirred for 5 h. Then the mixture was concentrated via rotary evaporator to remove all volatiles. The residue was diluted with H_2O (5 mL) and extracted with DCM (5 mL × 3), dried over Na_2SO_4, filtered, evaporated in vacuo and purified by column chromatography on silica gel (petroleum ether : ethyl acetate = 3 : 1) to give compound **9h** (0.0160 g, 67% yield, 96% ee) as a white solid.

9h: White solid; M.p. 195-198 °C; ^{1}H NMR (400 MHz, $CDCl_3$) δ 7.52 (s, 1H), 7.43 (d, J = 7.2 Hz, 2H), 7.40 (d, J = 7.6 Hz, 2H), 7.37-7.29 (m, 2H), 7.29-7.22 (m, 3H), 7.14 (d, J = 7.6 Hz, 1H), 7.03 (s, 1H), 6.80 (d, J = 8.0 Hz, 2H), 6.33 (d, J = 3.2 Hz, 1H), 5.37 (d, J = 2.8 Hz, 1H), 5.16 (d, J = 8.4 Hz, 1H), 4.72 (dt, J = 8.4, 2.8 Hz, 1H); ^{13}C NMR (100 MHz, $CDCl_3$) δ 171.6, 141.4, 140.7, 140.4, 140.0, 136.2, 130.5 (q, $J_{C\text{-}F}$ = 32.2 Hz), 129.9, 128.8, 128.7, 127.4, 127.1, 127.0, 124.5 (q, $J_{C\text{-}F}$ = 3.6 Hz), 124.0 (q, $J_{C\text{-}F}$ = 3.5 Hz), 123.9 (q, $J_{C\text{-}F}$ = 270.9 Hz), 120.1, 60.4, 51.4; ^{19}F NMR (376 MHz, $CDCl_3$) δ -62.8; HRMS *m/z* Calcd. for $C_{24}H_{19}F_3NO$ (M+H$^+$): 394.1413; Found: 394.1414.

Compound **9a** was synthesized from **3a** in 72% yield for two steps by following a similar procedure.

Compound 9a[7]

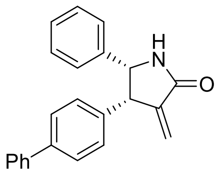

White solid; M.p. 230-235 °C; ^{1}H NMR (400 MHz, $CDCl_3$) δ 7.50-7.45 (m, 2H), 7.42-7.36 (m, 2H), 7.34-7.26 (m, 3H), 7.13-7.06 (m, 3H), 6.91-6.85 (m, 2H), 6.82 (d, J = 8.4 Hz, 2H), 6.47 (s, 1H), 6.32 (d, J = 3.2 Hz, 1H), 5.32 (d, J = 2.8 Hz, 1H), 5.06 (d, J = 8.4 Hz, 1H), 4.67 (dt, J = 8.4, 2.8 Hz, 1H); ^{13}C NMR (100 MHz, $CDCl_3$) δ 171.2, 142.0, 140.7, 139.9, 138.8, 136.5, 130.1, 128.8, 128.3, 127.9, 127.4, 127.0, 126.9, 126.6, 119.5, 60.8, 51.8; HRMS *m/z* Calcd. for $C_{23}H_{20}NO$ (M + H$^+$): 326.1539; Found: 326.1543.

7.3 Synthesis of Compounds 10h and 10h' (Fig. 4b).

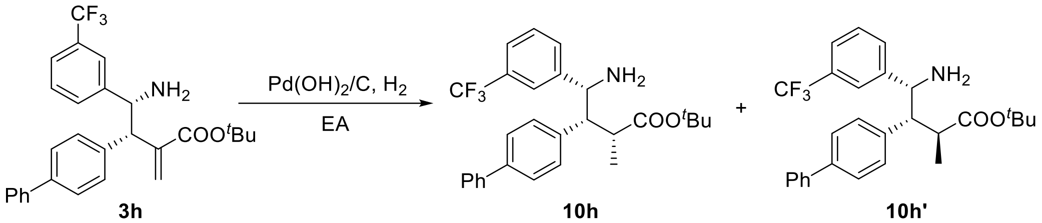

Compound (3*S*,4*R*)-**3h** (96% ee) (0.0370 g, 0.0792 mmol) was dissolved in a suspension of 10% $Pd(OH)_2/C$ (0.009 g) in 2.0 mL of EtOAc under 1 atm of hydrogen atmosphere. The mixture was stirred at room temperature for 12 h. The reaction mixture was filtered over celite and the solvent was removed under reduced pressure. Then the residue was purified by column chromatography on silica gel (petroleum ether : ethyl acetate = 3:1) to give a pair of diastereomers **10h** as yellow oil and **10h'** as a white solid (**10h**: 0.0170 g, 46% yield, 96% ee; **10h'**: 0.0120 g, 32% yield, 96% ee). The total yield for the two isomers of **10h** and **10h'** was 78%.

The diastereomeric ratio (dr) of product **10h** and **10h'** was determined to be 1.5:1 by ^1H NMR analysis of the crude reaction mixture. The absolute configuration of **10h'** was determined by X-ray analysis of its cyclization derivative **11h'**.

10h: Yellow oil; ^1H NMR (400 MHz, $CDCl_3$) δ 7.58-7.54 (m, 2H), 7.49 (d, *J* = 7.2 Hz, 1H), 7.47-7.40 (m, 4H), 7.40-7.30 (m, 4H), 7.07 (d, *J* = 8.0 Hz, 2H), 4.60 (d, *J* = 5.2 Hz, 1H), 3.01-2.88 (m, 2H), 1.27 (d, *J* = 6.0 Hz, 3H), 1.18 (s, 9H); ^{13}C NMR (100 MHz, $CDCl_3$) δ 174.4, 146.1, 140.9, 140.0, 136.9, 130.5, 130.4 (q, $J_{C\text{-}F}$ = 32.0 Hz), 130.3, 128.9, 128.6, 127.3, 127.1, 126.6, 124.3 (q, $J_{C\text{-}F}$ = 270.7 Hz), 124.1 (q, $J_{C\text{-}F}$ = 3.9 Hz), 123.9 (q, $J_{C\text{-}F}$ = 3.8 Hz), 80.4, 56.8, 56.0, 42.4, 27.8, 16.8; ^{19}F NMR (376 MHz, $CDCl_3$) δ -62.5; HRMS *m/z* Calcd. for $C_{28}H_{31}F_3NO_2$ $(M+H^+)$: 470.2301; Found: 470.2300.

10h': White solid; M.p. 66-69 °C; ^1H NMR (400 MHz, $CDCl_3$) δ 7.56 (d, *J* = 7.2 Hz, 2H), 7.47-7.41 (m, 5H), 7.38-7.28 (m, 4H), 7.0 (d, *J* = 8.0 Hz, 2H), 4.40 (d, *J* = 5.2 Hz, 1H), 3.23 (dd, *J* = 9.6, 5.2 Hz, 1H), 2.99-2.88 (m, 1H), 1.50 (s, 9H), 1.01 (d, *J* = 6.8 Hz, 3H); ^{13}C NMR (100 MHz, $CDCl_3$) δ 175.5, 145.5, 140.8, 140.0, 135.8, 130.5, 130.4 (q, $J_{C\text{-}F}$ = 31.9 Hz), 130.2, 128.9, 128.6, 127.4, 127.1, 126.8, 124.3 (q, $J_{C\text{-}F}$ = 270.7 Hz), 123.91 (q, $J_{C\text{-}F}$ = 3.9 Hz), 123.89 (q, $J_{C\text{-}F}$ = 3.8 Hz), 80.9, 57.6, 55.9, 42.4, 28.2, 15.8; ^{19}F NMR (376 MHz, $CDCl_3$) δ -62.5; HRMS *m/z* Calcd. for $C_{28}H_{31}F_3NO_2$ $(M+H^+)$: 470.2301; Found: 470.2299.

7.4 Synthesis of Compound 11h' (Fig. 4b).

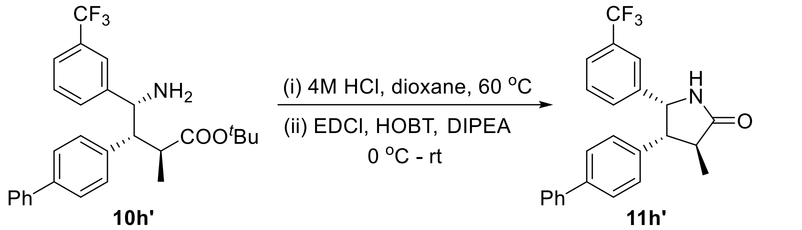

10h'
(96% ee)

11h'
79% yield for two steps, 96% ee

To a 5 mL vial were added compound **10h'** (96% ee) (0.0333 g, 0.0710 mmol), and 4 M HCl in dioxane (1.0 mL). The mixture was stirred at 60 °C for 3 h and then concentrated via rotary evaporator to remove most of the solvent to give crude product, which was used for the next step without further purification. The crude acid was dissolved in DCM (1.0 mL). To the solution was added 1-(3-dimethylaminopropyl)-3-ethylcarbodiimide hydrochloride (EDCl) (0.0180 g, 0.0939 mmol), 1-hydroxybenzotriazole (HOBT) (0.0192 g, 0.142 mmol). Then the reaction mixture was stirred at 0 °C for 5 min, followed by the addition of *N*,*N*-diisopropylethylamine (DIPEA) (0.0230 g, 0.178 mmol) at the same temperature. The reaction mixture was allowed to return to room temperature and stirred for 5 h. Then the mixture was concentrated via rotary evaporator to remove all volatiles. The residue was diluted with H_2O (5 mL) and extracted with DCM (5 mL × 3), dried over Na_2SO_4, filtered, evaporated in vacuo and purified by column chromatography on silica gel (petroleum ether : ethyl acetate = 3 : 1) to give compound **11h'** (0.0222g, 79% yield for two steps, 96% ee) as a white solid.

11h': White solid; M.p. 185-190 °C; ^{1}H NMR (400 MHz, $CDCl_3$) δ 7.50-7.45 (m, 2H), 7.44-7.37 (m, 3H), 7.36-7.30 (m, 3H), 7.27 (t, *J* = 7.6 Hz, 1H), 7.12 (d, *J* = 8.0 Hz, 1H), 7.00 (s, 1H), 6.85 (s, 1H), 6.81 (d, *J* = 8.4 Hz, 2H), 4.97 (d, *J* = 8.0, 1H), 3.75 (dd, *J* = 11.6, 8.0 Hz, 1H), 2.87 (dq, *J* = 11.6, 6.8 Hz, 1H), 1.23 (d, *J* = 7.2 Hz, 3H); ^{13}C NMR (100 MHz, $CDCl_3$) δ 180.2, 140.68, 140.67, 139.5, 135.4, 130.6 (q, J_{C-F} = 32.1 Hz), 129.8, 128.9, 128.74, 128.70, 127.5, 127.2, 127.1, 124.5 (q, J_{C-F} = 3.9 Hz), 123.91 (q, J_{C-F} =270.9 Hz), 123.88 (q, J_{C-F} = 3.7 Hz), 60.3, 55.0, 38.1, 13.6; ^{19}F NMR (376 MHz, $CDCl_3$) δ -62.8; HRMS *m/z* Calcd. for $C_{24}H_{21}F_3NO$ $(M+H^+)$: 396.1570; Found: 396.1576.

8. Kinetic Isotope Effect Studies

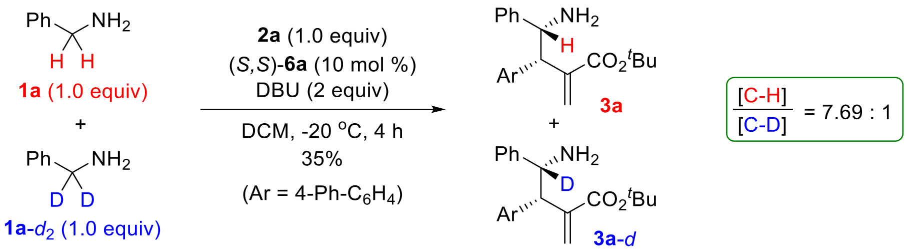

To a 5 mL vial equipped with a magnetic stirrer bar were successively added chiral pyridoxal (*S*,*S*)-**6a** (0.0092 g, 0.020 mmol), DBU (0.0609 g, 0.400 mmol), DCM (0.6 mL), benzylamine **1a** (0.0214 g, 0.20 mmol) and **1a-*d*₂** (0.0218 g, 0.20 mmol). The mixture was stirred at -20 °C for 5 min, and a solution of **2a** (0.0704 g, 0.20 mmol) in DCM (0.4 mL) was added in 7 portions over 1 h (the first portion: 20 mol% of the solution of **2a**; the rest amount of **2a** was then divided into 6 portions to be added into the reaction vial). After the reaction mixture was stirred at -20 °C for 3 h, it was allowed to warm to room temperature and concentrated via rotary evaporator to remove most of the solvent. The residue was submitted to purification by column chromatography on silica gel (petroleum ether : ethyl acetate = 5 : 1) to afford a mixture of **3a** and **3a-*d*** (0.0276 g, 35%) as a white solid. The ratio of **3a** to **3a-*d*** was determined as 7.69:1 by ^{1}H NMR analysis.

3a + **3a-*d***: ^{1}H NMR (400 MHz, $CDCl_3$) δ 7.64-7.56 (m, 4.52H), 7.49-7.42 (m, 4.52H), 7.41-7.30 (m, 5.65H), 7.28-7.23 (m, 1.33H), 6.10 (s, 1.13H), 5.65 (s, 1.13H), 4.58 (d, *J* = 10.0 Hz, 1H), 4.27 (d, *J* = 9.6 Hz, 1.13H), 1.57 (brs, 2.26H), 1.32 (s, 10.17H).

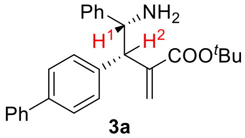

3a

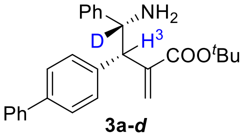

3a-d

$$\frac{[\text{C-H}]}{[\text{C-D}]} = \frac{\textbf{3a}\ (\text{H}^1)}{\textbf{3a}\ (\text{H}^2)+\textbf{3a-}\boldsymbol{d}\ (\text{H}^3)-\textbf{3a}\ (\text{H}^1)} = 1{:}(1.13\text{-}1)\ = 7.69 : 1$$

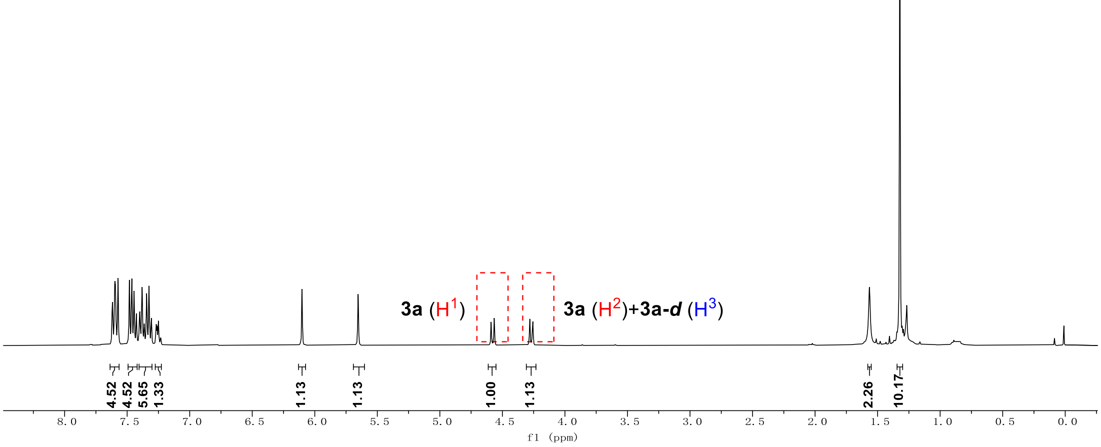

9. Experiments on Catalyst Comparison

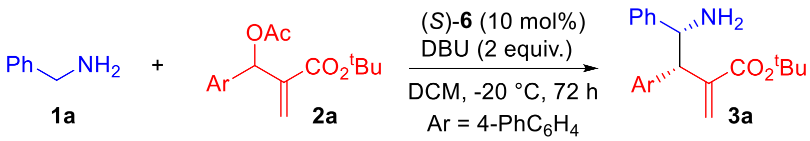

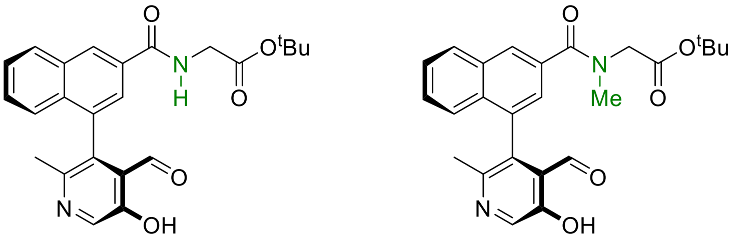

(*S*)-**6e**: 34% yield, >20:1 dr, 92% ee (*S*)-**6f:** 10% yield, >20:1 dr, 53% ee

To a 5 mL vial were successively added chiral pyridoxal **6e** or **6f** (0.010 mmol), DBU (0.0304 g, 0.20 mmol), DCM (0.3 mL) and benzylamine **1a** (0.0214 g, 0.2 mmol). The mixture was stirred at -20 °C for 5 min, and a solution of **2a** (0.0352 g, 0.10 mmol) in DCM (0.2 mL) was added in 7 portions over 1 h (the first portion: 20 mol% of the solution of **2a**; the rest amount of **2a** was then divided into 6 portions to be added into the reaction vial). After the reaction mixture was stirred at -20 °C for 72 h, it was allowed to warm up to room temperature and then concentrated via rotary evaporator to remove most of the solvent. The residue was submitted to ^{1}H NMR analysis in $CDCl_3$ to determine the dr values. The sample was collected and purified by column chromatography on silica gel (petroleum ether : ethyl acetate = 5 : 1) to give compound **3a** (for **6e**: 0.0135 g, 34% yield; for **6f**: 0.0041 g, 10% yield) as a white solid. The enantiomeric excess (ee) of product **3a** was determined by chiral HPLC analysis.

10. Computational Studies

10.1 DFT Calculations for pK_a Estimation

The pK_a values of the target acids were computed by using the proton-transfer method as reported in the literature[8]. Taking the benzylic C-H bond as an example, compound **27** with a reported pK_a of 24.3 (in DMSO) was used as the reference acid. The proton-transfer reaction in DMSO between the benzylic C-H bond and **27** was designed as shown in Eq. (1). The pK_a value of the benzylic C-H bond was derived on the basis of the free-energy change (ΔG_{soln}) of the proton-transfer reaction and the reported pK_a value of **27**, as shown in Eq. (2). The pK_a calculation method for phenolic O-H in compound **12** is the same as that for the benzylic C–H bond, with compound **29** as the reference (pK_a = 15.7) (Eq. (3)) (Table S2). The pK_a calculation method for amide N-H bond of **12** is the same as that for the benzylic C–H bond, with compound **31** as the reference (pK_a = 18.8) (Eq. (4)) (Table S2). These reference values were taken from the Bordwell pK_a Table (https://organicchemistrydata.org/hansreich/resources/pka/#references).

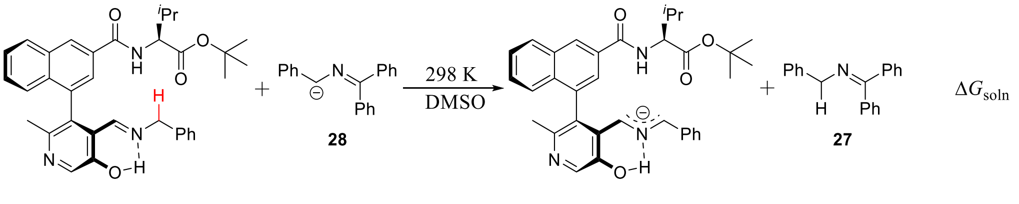

ΔG_{soln} (1)

$$\mathrm{p}K_a(\text{benzylic C-H}) = \frac{\Delta G_{soln}}{RT\ln(10)} + \mathrm{p}K_a(\mathbf{27}) = \frac{\Delta G_{soln}}{RT\ln(10)} + 24.3 \quad (2)$$

$$\mathrm{p}K_a(\text{phenolic O-H}) = \frac{\Delta G_{soln}}{RT\ln(10)} + \mathrm{p}K_a(\mathbf{29}) = \frac{\Delta G_{soln}}{RT\ln(10)} + 15.7 \quad (3)$$

$$\mathrm{p}K_a(\text{N-H bond}) = \frac{\Delta G_{soln}}{RT\ln(10)} + \mathrm{p}K_a(\mathbf{31}) = \frac{\Delta G_{soln}}{RT\ln(10)} + 18.8 \quad (4)$$

We carried out a computational pK_a comparison, and the results are summarized in Table S2. Based on our calculation results, the estimated pK_a values for the benzylic C-H, phenolic O-H and N-H bonds in compound **12** are 17.7, 21.1 and 23.3, respectively. These results indicate that the benzylic C–H bond is the most acidic among the three sites. Therefore, deprotonation at this position is thermodynamically favored, providing a rationale for the selective formation of the reactive carbanion in our proposed mechanism.

Table S2. Computational pK_a comparison for the benzylic C-H, phenolic O-H and N-H bonds of compound **12**. Calculations were finished using the Gaussian 16 computational program.[9] All Gibbs free energy values were calculated at the level of B3LYP[10-13]-D3(BJ)[14]/def2-TZVP[15] with PCM solvation[16] (DMSO), based on geometries optimized at the B3LYP-D3(BJ)/6-31G(d,p) level[17-19] at 298K.

Reference	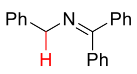 **27** pK_a (C-H) = 24.3	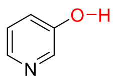 **29** pK_a (O-H) = 15.7	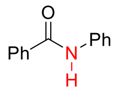 **31** pK_a (N-H) = 18.8
X-H	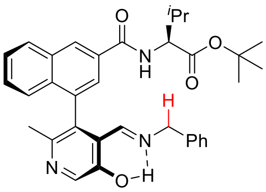 **12** pK_a (C-H) = 17.7	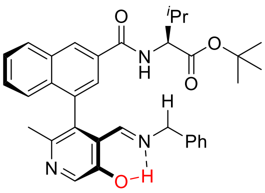 **12** pK_a (O-H) = 21.1	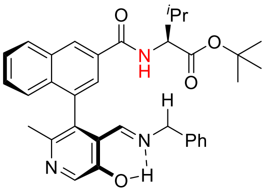 **12** pK_a (N-H) = 23.3

Table S3. Energies (Hartree) for pK_a calculation.

Geometry	H	G
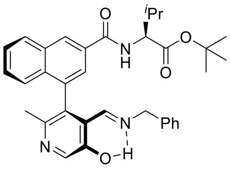 **12**	-1783.300193	-1783.413426
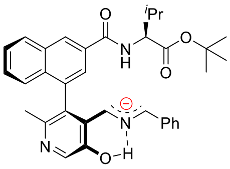 **13**	-1782.821072	-1782.938356
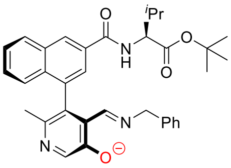 **25**	-1782.821469	-1782.935666
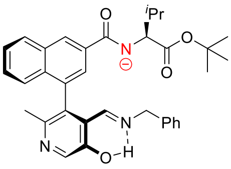 **26**	-1782.811497	-1782.923725
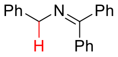 **27**	-827.169124	-827.234486

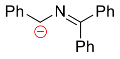<br>**28**	-826.68143	-826.745053
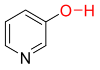<br>**29**	-323.561609	-323.596863
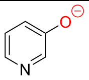<br>**30**	-323.096077	-323.130776
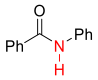<br>**31**	-632.08493	-632.137409
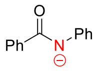<br>**32**	-631.605499	-631.657401

Table S4. Cartesian coordinates for *p*K$_a$ estimation.

Geometry	Coordinates			
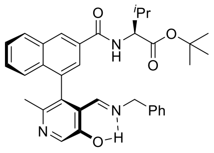<br>**12**	C	-6.26438800	-1.63018200	-1.08514800
	C	-5.32802100	-0.85407200	-0.43879400
	C	-3.99988500	-1.32024500	-0.25647500
	C	-3.64129500	-2.60044600	-0.79288000
	C	-4.63241000	-3.37735200	-1.45116000
	C	-5.91777900	-2.90690400	-1.58935900
	H	-7.27606800	-1.25935700	-1.21470600
	H	-5.59651300	0.12866600	-0.06772400
	C	-2.99936100	-0.55653300	0.42380100
	C	-2.30191600	-3.04758600	-0.69243800
	H	-4.35195500	-4.34762200	-1.84989300
	H	-6.66765500	-3.50739800	-2.09394300
	C	-1.33730800	-2.27558800	-0.07940600
	C	-1.70893200	-1.03955900	0.50696500
	H	-2.01486700	-3.99865900	-1.12773800
	H	-0.97983700	-0.44749300	1.04995800
	C	-3.30218700	0.77885200	1.01043200
	C	-2.71536300	1.93371400	0.44588900
	C	-4.10592800	0.91069800	2.15527500
	C	-2.95079700	3.18161100	1.06798900
	C	-3.79150400	3.20522200	2.18954600
	H	-3.99603800	4.15864100	2.67165300
	C	-4.72248900	-0.28255300	2.84039700
	H	-5.60208300	-0.64930700	2.30025200
	H	-4.01623900	-1.11496500	2.90452100
	H	-5.03484300	0.00169700	3.84632400
	N	-4.34337800	2.11365700	2.71407000
	O	-2.40378400	4.32573200	0.62997300
	H	-1.81437900	4.07690900	-0.14677700
	C	-1.87705200	1.86189800	-0.74943000
	H	-1.79169400	0.89633800	-1.25408100
	N	-1.25132100	2.89485800	-1.18464300
	C	-0.37558500	2.73410800	-2.33236700
	H	-0.61979000	3.51311600	-3.06331400
	C	1.09145400	2.85186900	-1.95092700
	C	2.04834700	3.00826400	-2.96281700
	C	1.51727600	2.77574400	-0.62257100
	C	3.40440000	3.08206300	-2.65116800
	H	1.72716000	3.06986000	-3.99938800

	C	2.87507600	2.86313400	-0.30844500
	H	0.78846200	2.65417900	0.17044700
	C	3.82302700	3.01351600	-1.31900700
	H	4.13446800	3.19942000	-3.44624600
	H	3.18558200	2.80513200	0.72907100
	H	4.87870800	3.07600700	-1.07400400
	C	0.08145400	-2.76012000	-0.12663600
	O	0.36149900	-3.92825100	-0.42070300
	N	1.03493800	-1.83059100	0.12764500
	H	0.80021900	-0.86520400	0.30776600
	C	2.45050200	-2.11855300	0.00513100
	H	2.63614600	-3.12527600	0.38673900
	C	2.91061100	-2.06830600	-1.48517900
	H	2.21100500	-2.74349500	-1.99112300
	C	2.75915800	-0.66803000	-2.08785300
	H	2.96687700	-0.69616600	-3.16160600
	H	1.74782300	-0.27406900	-1.95530800
	H	3.45320200	0.04742100	-1.63605600
	C	4.32412600	-2.62172200	-1.68642700
	H	4.43531900	-3.61135300	-1.23165000
	H	4.53651100	-2.71475800	-2.75595000
	H	5.07939900	-1.96499300	-1.24784300
	C	3.18933800	-1.10869400	0.87531000
	O	2.69437700	-0.04956400	1.22189800
	O	4.41764300	-1.53837700	1.15657800
	C	5.40428600	-0.69778700	1.88069400
	C	5.69620400	0.55509900	1.05767300
	C	4.88097800	-0.37682800	3.27972300
	C	6.62626300	-1.60960200	1.94808900
	H	6.03087600	0.28270900	0.05263800
	H	4.81318500	1.18680600	0.97582900
	H	6.49396200	1.12606100	1.54117600
	H	4.60653800	-1.29774100	3.80212000
	H	5.67112900	0.11798800	3.85171600
	H	4.01306800	0.28037500	3.23701700
	H	7.44144300	-1.09585700	2.46421600
	H	6.39267700	-2.52751700	2.49414700
	H	6.96411000	-1.87641300	0.94315100
	H	-0.54726700	1.76373200	-2.82344000
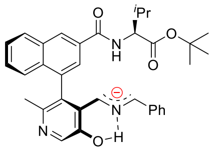<br>13	C	-2.98199900	-4.48042600	-1.09177300
	C	-2.48346400	-3.49499600	-0.26900700
	C	-1.31708400	-2.76930400	-0.62800500
	C	-0.66596700	-3.08345700	-1.86552600
	C	-1.20597000	-4.10630500	-2.69213100
	C	-2.33912500	-4.78969200	-2.31565400
	H	-3.87472400	-5.02664600	-0.80326700
	H	-2.97315800	-3.25536500	0.66796700
	C	-0.77962700	-1.73400500	0.20407100
	C	0.50070100	-2.37203400	-2.23511100
	H	-0.70487000	-4.33786000	-3.62762500
	H	-2.74396600	-5.56864300	-2.95405300
	C	1.02131800	-1.39186800	-1.41612800
	C	0.36137200	-1.07222500	-0.19973500
	H	1.00048300	-2.59994700	-3.17063400
	H	0.73062000	-0.26609900	0.42580300
	C	-1.44210600	-1.37044200	1.48916600
	C	-2.58414900	-0.51914400	1.47349300
	C	-0.94047600	-1.87068800	2.70058200
	C	-3.14962900	-0.23120100	2.75874700
	C	-2.57134500	-0.77355400	3.89400600

	H	-3.01555000	-0.54058600	4.86132400
	C	0.26258500	-2.78671300	2.72165500
	H	0.13830700	-3.63272100	2.03704800
	H	1.17786500	-2.26725500	2.41554500
	H	0.40642800	-3.16985000	3.73376700
	N	-1.48888600	-1.58140800	3.89360000
	O	-4.24394600	0.56816500	2.87467300
	H	-4.47027600	0.82967100	1.92517200
	C	-3.13282300	0.01662500	0.27326800
	H	-2.65905300	-0.21776700	-0.68170400
	N	-4.21850500	0.80522600	0.30563800
	C	-4.77759000	1.33731000	-0.76587400
	H	-4.34832600	1.13696500	-1.75436200
	C	-5.93883200	2.18629800	-0.70795900
	C	-6.47346500	2.72836900	-1.90361500
	C	-6.59852300	2.52458400	0.50189400
	C	-7.59065400	3.55642700	-1.89160200
	H	-5.99130400	2.48733200	-2.84817500
	C	-7.71457900	3.35284000	0.50522200
	H	-6.22219200	2.12791600	1.43898100
	C	-8.22612600	3.88003300	-0.68755000
	H	-7.97088000	3.95387600	-2.82924600
	H	-8.19540900	3.59221700	1.45052900
	H	-9.09808500	4.52643300	-0.67764400
	C	2.27011000	-0.69517300	-1.86238100
	O	2.63654800	-0.69698000	-3.04441400
	N	2.98007100	-0.06846700	-0.89078400
	H	2.75586800	-0.19160500	0.08712600
	C	4.21950600	0.62894600	-1.17266500
	H	4.78845300	0.06170400	-1.91541200
	C	3.94696900	2.05076000	-1.75221900
	H	3.23168000	1.86416800	-2.56089800
	C	3.28535500	2.96700100	-0.71886800
	H	2.96895200	3.90312200	-1.18800800
	H	2.40338100	2.49528200	-0.27603100
	H	3.97854700	3.22072700	0.09114000
	C	5.19413000	2.69035100	-2.36800000
	H	5.66880000	2.02047800	-3.09242300
	H	4.91928900	3.61218500	-2.88988300
	H	5.93603500	2.94034800	-1.60586600
	C	5.00828200	0.67598500	0.13073900
	O	4.49250300	0.48467200	1.21996100
	O	6.28445900	0.97467500	-0.09484900
	C	7.25271100	1.18472700	1.01244700
	C	6.79094000	2.35670600	1.87670500
	C	7.40758200	-0.11149200	1.80595600
	C	8.53781700	1.52724000	0.26389400
	H	6.63426500	3.24592700	1.25926900
	H	5.86668000	2.12130900	2.40402100
	H	7.56620400	2.58558300	2.61348200
	H	7.66819000	-0.93639900	1.13677900
	H	8.21646500	0.00788000	2.53240300
	H	6.49066600	-0.36110100	2.33923800
	H	9.34591500	1.70015900	0.97937200
	H	8.82850200	0.70670200	-0.39746000
	H	8.40586000	2.43123700	-0.33666000

	C	-6.15865000	-1.64019500	-1.17876100
	C	-5.25375400	-0.85433200	-0.50063300
	C	-3.92330300	-1.29932000	-0.28001000
	C	-3.53319500	-2.57639600	-0.80194500
	C	-4.49240900	-3.36494600	-1.49414800
	C	-5.77804700	-2.91068200	-1.67586000
	H	-7.17177700	-1.28427400	-1.33768700
	H	-5.54400600	0.11990400	-0.12388300
	C	-2.96166600	-0.51366400	0.43342600
	C	-2.19708700	-3.01357100	-0.64078200
	H	-4.18728800	-4.33262200	-1.88173500
	H	-6.50289000	-3.52042300	-2.20584600
	C	-1.26840200	-2.22605400	0.00779100
	C	-1.67069200	-0.98491800	0.56400200
	H	-1.88588900	-3.96850600	-1.05055200
	H	-0.96320400	-0.38085100	1.12256700
	C	-3.33375800	0.80658600	1.02577100
	C	-2.82701800	2.01155700	0.46319300
	C	-4.14738500	0.82937300	2.16293600
	C	-3.18028100	3.27873800	1.08345200
	C	-4.05039300	3.13427700	2.23326100
	H	-4.34774300	4.06205600	2.72741000
	C	-4.67199800	-0.42584900	2.82001500
	H	-5.53395100	-0.84867000	2.28791900
	H	-3.91336000	-1.21371100	2.86518700
	H	-4.99263400	-0.19070000	3.83755400
	N	-4.49633800	2.00412900	2.74865400
	O	-2.80930500	4.42400400	0.69874300
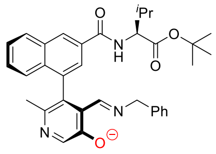 25	C	-1.99622400	1.92410700	-0.72960700
	H	-2.00561700	0.93923400	-1.21882000
	N	-1.26740600	2.85825000	-1.23211600
	C	-0.49762800	2.49151600	-2.40574900
	H	-0.77434200	3.15859000	-3.23540000
	C	1.00197900	2.61522700	-2.18218900
	C	1.88093800	2.44853900	-3.26183600
	C	1.53415000	2.88067900	-0.91781500
	C	3.25942900	2.54399000	-3.08074600
	H	1.47912500	2.24140500	-4.25089300
	C	2.91501600	2.98522100	-0.73507400
	H	0.85529700	3.01359200	-0.08334000
	C	3.78310200	2.81627900	-1.81309900
	H	3.92602300	2.40891300	-3.92749800
	H	3.30981600	3.19568100	0.25395700
	H	4.85656800	2.89327900	-1.66958000
	C	0.14842600	-2.71170700	0.05138300
	O	0.43967900	-3.90256100	-0.11782100
	N	1.09268400	-1.75939800	0.24996700
	H	0.85008500	-0.78076200	0.31454300
	C	2.51103100	-2.05870000	0.23904000
	H	2.67862500	-2.99052800	0.78554400
	C	3.04193500	-2.24536900	-1.21637700
	H	2.35037200	-2.97748300	-1.64760000
	C	2.95321200	-0.95034200	-2.02856600
	H	3.20173400	-1.14284000	-3.07650500
	H	1.94930100	-0.51788300	-1.99928000
	H	3.64987300	-0.19064100	-1.66050000
	C	4.44857700	-2.84877700	-1.26082400
	H	4.51182700	-3.76032100	-0.65751500
	H	4.70827900	-3.10733400	-2.29210900
	H	5.19945000	-2.14776300	-0.88848900

	C	3.20990200	-0.91753700	0.96842400
	O	2.68936800	0.17163400	1.13897200
	O	4.43671400	-1.27416100	1.34380900
	C	5.38650600	-0.30746700	1.95035800
	C	5.67684700	0.80622900	0.94622000
	C	4.81904500	0.21866800	3.26782800
	C	6.62320800	-1.16858300	2.19180400
	H	6.04874100	0.38593900	0.00732000
	H	4.78356800	1.39410100	0.74014300
	H	6.44725300	1.46708700	1.35377400
	H	4.54432200	-0.61415600	3.92150400
	H	5.58524100	0.81229500	3.77477000
	H	3.94269500	0.84380700	3.10012600
	H	7.41364100	-0.56086700	2.63990300
	H	6.39176400	-1.99370900	2.87076400
	H	6.99451500	-1.58314100	1.25079200
	H	-0.71812000	1.46364100	-2.75099500
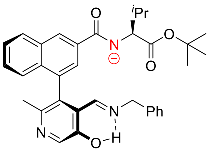<br>**26**	C	-6.14509700	-1.96183200	-1.05397300
	C	-5.22502400	-1.18172100	-0.38747400
	C	-3.85078600	-1.53659100	-0.35769800
	C	-3.42761900	-2.71114100	-1.06274300
	C	-4.40328600	-3.49518300	-1.73596000
	C	-5.73215200	-3.13460700	-1.73008300
	H	-7.19181000	-1.67401500	-1.06578200
	H	-5.54420300	-0.27642400	0.11811700
	C	-2.86160100	-0.76144100	0.33031000
	C	-2.05081600	-3.05206400	-1.09089100
	H	-4.07603000	-4.38651000	-2.26416000
	H	-6.46561700	-3.74318600	-2.24998300
	C	-1.10176500	-2.26746500	-0.47097900
	C	-1.53182100	-1.12405300	0.25008800
	H	-1.72247200	-3.93838800	-1.62429300
	H	-0.77159200	-0.53529000	0.74986200
	C	-3.24112300	0.43397400	1.13505800
	C	-2.83508500	1.72409500	0.72855000
	C	-3.95039400	0.29619000	2.34286800
	C	-3.15268300	2.82679900	1.55717900
	C	-3.88505800	2.57914100	2.72490800
	H	-4.14821300	3.41672300	3.36743900
	C	-4.37278200	-1.05207600	2.87027000
	H	-5.24668600	-1.43990900	2.33566500
	H	-3.57538200	-1.79157900	2.75554100
	H	-4.63061900	-0.96067700	3.92665900
	N	-4.26547000	1.36084700	3.10524600
	O	-2.78146000	4.08402800	1.26635600
	H	-2.24477800	4.02568100	0.41472700
	C	-2.10410000	1.93954100	-0.51852000
	H	-1.94382400	1.07529400	-1.16804800
	N	-1.66070400	3.10376700	-0.83142000
	C	-0.88029700	3.24758100	-2.05470700
	H	-1.30142000	4.07235400	-2.63761600
	C	0.56921400	3.54855300	-1.72155000
	C	1.17780900	4.72663500	-2.16414700
	C	1.31680600	2.64013800	-0.96184000
	C	2.51540200	4.99123300	-1.86069100
	H	0.60352300	5.43884100	-2.75029500
	C	2.65152800	2.90122500	-0.65474000
	H	0.86104800	1.71990800	-0.60864400
	C	3.25345200	4.07958100	-1.10457900
	H	2.97665300	5.90927100	-2.21226500

	H	3.20540200	2.17622000	-0.06890800
	H	4.29277200	4.28549500	-0.86646500
	C	0.37003300	-2.62244300	-0.58106800
	O	0.66403800	-3.76161500	-1.06207400
	N	1.18098300	-1.66214300	-0.16763000
	C	2.58431100	-1.98636600	-0.29719600
	H	2.79069300	-3.02706100	-0.00651100
	C	3.09792800	-1.85920000	-1.77404300
	H	2.43229200	-2.53698900	-2.31872200
	C	2.92491400	-0.44711400	-2.33656900
	H	3.16116300	-0.42605000	-3.40650200
	H	1.89888100	-0.09967500	-2.20003500
	H	3.58464000	0.26849100	-1.83467000
	C	4.53491900	-2.35813900	-1.95834300
	H	4.66664100	-3.36376100	-1.54517500
	H	4.79276200	-2.39361500	-3.02283100
	H	5.25785500	-1.70141400	-1.46425400
	C	3.40250700	-1.08851800	0.62256300
	O	3.13844900	0.06713700	0.90689000
	O	4.49708500	-1.74327600	1.06535000
	C	5.52261700	-1.08342800	1.89159800
	C	6.13682600	0.09506200	1.13375500
	C	4.91785800	-0.66135700	3.23164000
	C	6.55581600	-2.19174200	2.08973100
	H	6.51166600	-0.23331000	0.15984700
	H	5.40802800	0.89059200	0.98278100
	H	6.98013000	0.49299000	1.70626500
	H	4.43783100	-1.51750900	3.71492400
	H	5.71155900	-0.29734600	3.89104200
	H	4.17996300	0.12881100	3.09667500
	H	7.38425200	-1.82646600	2.70283400
	H	6.10474200	-3.05213900	2.59189900
	H	6.95461800	-2.52129300	1.12626400
	H	-0.94573600	2.33483600	-2.66527300
	C	3.43816700	-1.70088900	1.41789900
	C	2.34720000	-1.38301300	0.60376700
	C	2.53422600	-0.69118400	-0.59647500
	C	3.83488000	-0.32696700	-0.97082900
	C	4.92330500	-0.64124900	-0.15982500
	C	4.72835800	-1.33096000	1.04072200
	H	3.27559200	-2.23859500	2.34729800
	H	1.34509200	-1.67675500	0.89617500
	H	3.99208900	0.21017800	-1.90281700
	H	5.92414400	-0.34884900	-0.46323900
	H	5.57571800	-1.57615000	1.67350000
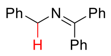 27	C	1.37161100	-0.30222400	-1.49437600
	H	1.33850500	0.79331500	-1.57025800
	H	1.57699000	-0.68341300	-2.50324200
	N	0.11132500	-0.88442600	-1.06873900
	C	-0.79239500	-0.16103000	-0.51228300
	C	-2.08415600	-0.81383600	-0.15285200
	C	-2.43225600	-2.04554200	-0.73204400
	C	-2.95898300	-0.23277900	0.77750100
	C	-3.62419700	-2.67632000	-0.39167200
	H	-1.75222600	-2.48889900	-1.45013100
	C	-4.15161100	-0.86927700	1.12264500
	H	-2.70158400	0.71356300	1.23897300
	C	-4.48923300	-2.09002900	0.53820900
	H	-3.88435200	-3.62394300	-0.85325100
	H	-4.81526000	-0.41041300	1.84878600

	H	-5.42008800	-2.58226900	0.80224800
	C	-0.64920100	1.29695200	-0.19360100
	C	-1.51606500	2.23126200	-0.77769000
	C	0.34351300	1.74340500	0.68900300
	C	-1.38033600	3.59027100	-0.49851800
	H	-2.29377000	1.88881100	-1.45308200
	C	0.46995900	3.10220900	0.97731600
	H	1.01295400	1.02430800	1.14919300
	C	-0.38847500	4.02817200	0.38202800
	H	-2.05081400	4.30598300	-0.96373400
	H	1.23915600	3.43658100	1.66635900
	H	-0.28729000	5.08568300	0.60484600
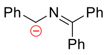 28	C	4.60726600	-1.59644200	0.10511000
	C	3.26652300	-1.23336200	0.11059400
	C	2.86744900	0.12082500	-0.04381500
	C	3.90427100	1.07764600	-0.19289800
	C	5.24499200	0.70662200	-0.19493800
	C	5.61678200	-0.63506700	-0.04767100
	H	4.87563400	-2.64386000	0.22508100
	H	2.49198100	-1.98253300	0.23875400
	H	3.63473200	2.12525500	-0.31107700
	H	6.01010300	1.47045100	-0.31327000
	H	6.66320400	-0.92438100	-0.04834800
	C	1.48726900	0.53631500	-0.04785000
	H	1.30730400	1.61307600	-0.13872400
	N	0.50208200	-0.33949600	0.03006900
	C	-0.81556800	-0.08834300	0.02334000
	C	-1.71005000	-1.24005600	0.03176100
	C	-1.21626700	-2.55418300	-0.19089000
	C	-3.10173200	-1.13378200	0.29193400
	C	-2.04887400	-3.66555100	-0.15949500
	H	-0.15727400	-2.66921700	-0.39194600
	C	-3.93174100	-2.25148800	0.31437100
	H	-3.53031800	-0.15774300	0.49176400
	C	-3.42192000	-3.53383200	0.08819400
	H	-1.62504500	-4.65118100	-0.33989200
	H	-4.99139300	-2.11870800	0.52089300
	H	-4.07188200	-4.40339100	0.10376000
	C	-1.37188800	1.28753300	0.00565200
	C	-2.35604100	1.67665700	-0.92639500
	C	-0.92500300	2.27139900	0.91176400
	C	-2.87141500	2.97161200	-0.94784800
	H	-2.71648200	0.94425100	-1.64253100
	C	-1.43304700	3.56891900	0.88963400
	H	-0.17429100	2.00379900	1.64849300
	C	-2.41159800	3.93011600	-0.04074700
	H	-3.62828200	3.23680200	-1.68134100
	H	-1.07067200	4.29991000	1.60764400
	H	-2.80832500	4.94072300	-0.05859100
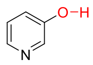 29	C	-0.19287800	-1.19558300	0.00018700
	C	-0.92483100	0.00057800	-0.00002000
	C	-0.22579000	1.20932600	0.00002800
	C	1.16670700	1.16606200	0.00007900
	C	1.80740800	-0.07200400	-0.00014700
	N	1.14191300	-1.23760100	0.00000400
	H	-0.75701900	2.15693200	0.00014000
	H	-0.72910700	-2.14201800	0.00009900
	H	1.74584000	2.08322200	0.00008100
	H	2.89275100	-0.13198200	-0.00024500
	O	-2.28225100	-0.09326000	-0.00013400

	H	-2.67154200	0.79286100	0.00020900
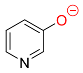 30	C	-0.25957600	-1.18119700	-0.00016200
	C	-1.05309500	0.02755500	0.00011000
	C	-0.24921400	1.22114400	0.00016200
	C	1.13738500	1.14742800	0.00020700
	C	1.77079100	-0.09972900	0.00018300
	N	1.07232400	-1.25178200	-0.00007800
	H	-0.75767000	2.18382000	0.00018600
	H	-0.80230800	-2.13008200	-0.00041900
	H	1.73809600	2.05548800	0.00034100
	H	2.85643300	-0.17931600	0.00027800
	O	-2.32732100	0.01767000	-0.00035400
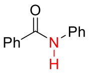 31	C	-3.61763500	-1.49193600	-0.37341000
	C	-2.27642900	-1.11271000	-0.41400300
	C	-1.89880800	0.18453000	-0.03882200
	C	-2.88382900	1.09889600	0.35750400
	C	-4.22138900	0.71599200	0.40587200
	C	-4.59081400	-0.58164300	0.04236200
	H	-3.90193200	-2.49491500	-0.67448700
	H	-1.53860500	-1.82328400	-0.77338200
	H	-2.58078800	2.10414200	0.62743700
	H	-4.97610900	1.42751300	0.72518500
	H	-5.63385000	-0.87988300	0.07613600
	C	-0.48017600	0.66986200	-0.07101100
	O	-0.21571000	1.86640000	-0.20364600
	N	0.46878300	-0.30898400	0.07287500
	H	0.12404300	-1.23236100	0.29279500
	C	1.87226000	-0.19357500	0.05003400
	C	2.61118300	-1.36050000	0.30419800
	C	2.54713700	1.00645000	-0.22145900
	C	4.00203700	-1.32993500	0.28892300
	H	2.09010900	-2.29096100	0.51277800
	C	3.94229400	1.01934600	-0.23381900
	H	1.97841000	1.90366200	-0.41235100
	C	4.67875800	-0.13808100	0.01934300
	H	4.55646900	-2.24165000	0.48801900
	H	4.45545100	1.95274100	-0.44481900
	H	5.76345900	-0.11348400	0.00747700
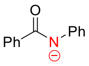 32	C	4.23055900	-0.77292000	0.00016200
	C	2.88437900	-1.13868600	0.00008300
	C	1.87325100	-0.16859000	-0.00004300
	C	2.24485600	1.18392300	-0.00006800
	C	3.58915900	1.55462000	0.00001100
	C	4.58894500	0.57719800	0.00011300
	H	5.00102800	-1.53923400	0.00025300
	H	2.58503200	-2.18107600	0.00013000
	H	1.45679900	1.92797400	-0.00015900
	H	3.86022400	2.60698400	-0.00001200
	H	5.63617400	0.86565000	0.00016400
	C	0.42004100	-0.61670800	-0.00014900
	O	0.20714700	-1.86263500	-0.00026900
	N	-0.44626100	0.40080900	-0.00009800
	C	-1.81793700	0.20896000	-0.00006100
	C	-2.52516500	-1.02463300	0.00004200
	C	-2.60945500	1.38795100	-0.00005900
	C	-3.92011200	-1.05272900	0.00014200
	H	-1.94984600	-1.93913600	0.00005400
	C	-3.99878800	1.34905400	0.00003400
	H	-2.08554100	2.34026900	-0.00011900
	C	-4.67456700	0.12296400	0.00013500

	H	-4.42710100	-2.01567800	0.00023900
	H	-4.56106200	2.27998700	0.00001900
	H	-5.76004700	0.08725400	0.00021100

10.2 Comprehensive Conformational Search for Intermediate 13

Computational Details: Calculations were finished using the Gaussian 16 computational program. Geometry optimizations were carried out with B3LYP-D3(BJ) functional. The 6-31G(d, p) basis set was used for other atoms. Frequency calculations at the same level were carried out to confirm each stationary point to be either a minimum or transition geometry. Solvent effects for DCM are calculated by using the PCM solvation model. The zero-point energy and thermal energy corrections are calculated at 253K. The single-point energies were calculated at the level of B3LYP-D3(BJ)/def2-TZVP.

We have conducted a comprehensive conformational search for carbanion intermediate **13**. The various conformers of **13** along with their relative Gibbs free energies are presented in Figure S1. Based on these results, **s13-1** was identified as the most stable form and has therefore used to in the following transition state calculations.

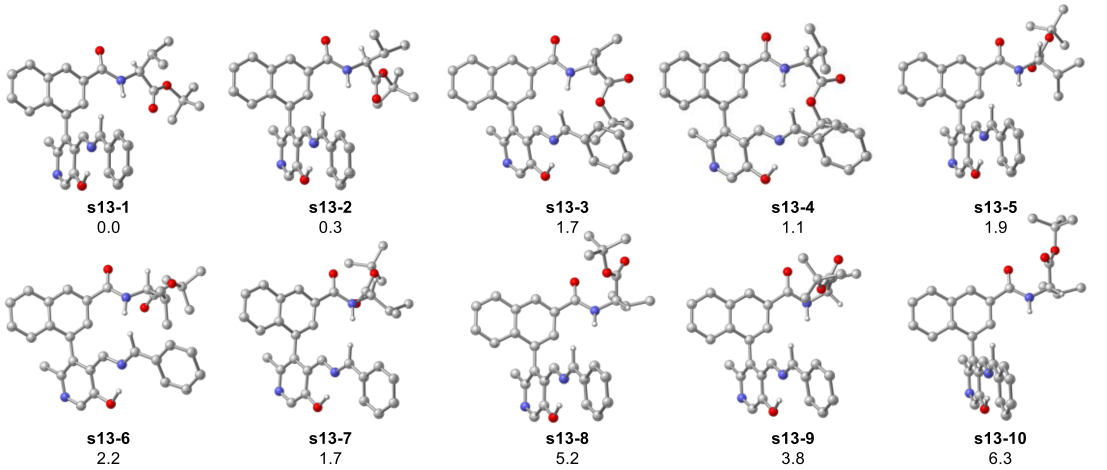

Figure S1. Conformational search and relative Gibbs free energies (kcal/mol) for intermediate **13.**

10.3 Comprehensive Transition State Calculations with B3LYP-D3(BJ) Functional and Energy Decomposition Analysis

Computational Details: Calculations were finished using the Gaussian 16 computational program. Geometry optimizations were carried out with B3LYP-D3(BJ) functional. The 6-31G(d, p) basis set was used for other atoms. Frequency calculations

at the same level were carried out to confirm each stationary point to be either a minimum or transition geometry. Solvent effects for DCM are calculated by using the PCM solvation model. The zero-point energy and thermal energy corrections are calculated at 253K. The single-point energies were calculated at the level of B3LYP-D3(BJ)/def2-TZVP.

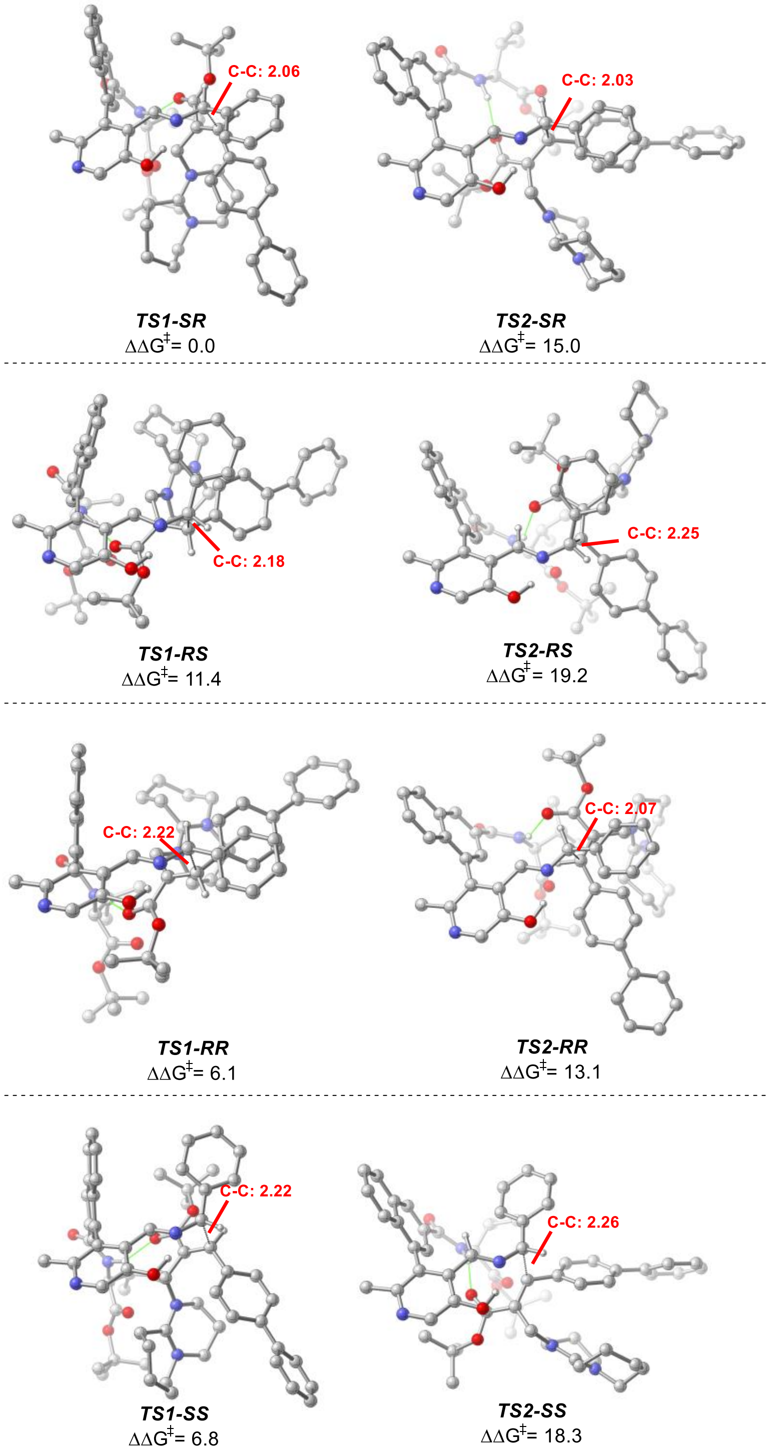

Figure S2. Relative Gibbs free energies (kcal/mol) for transition states.

Representative transition states and the relative Gibbs free energies are given in Figure S2. For clarity, the calculated transition states are labeled systematically. For example, in the transition state ***TS1-SR*** (numbered as **15** in the main text) and ***TS2-SR***, the C-C bond is formed leading to the product **3a** with (3*S*,4*R*) configuration. Among these transition states, ***TS1-SR*** was identified as the most stable one.

Energy decomposition analysis for transition state. We have performed energy decomposition analysis (EDA) using the ADF (2025.102) package[20,21] at the B3LYP(D3BJ)/DZP level of theory.[22] The EDA results summarized in Figure S3 clearly demonstrate that orbital interactions are the primary factor in favor of ***TS1-SR*** over ***TS1-RS***. ($\Delta\Delta E_{orb}$ = -14.8 kcal/mol). We have conducted a qualitative analysis using NCI plots.[23,24] NCI-plots (Figure S4a) revealed the presence of van der Waals interactions in both ***TS1-SR*** and ***TS1-RS*** qualitatively. No significant steric repulsion was observed between the aryl substituents and nearby functional groups in ***TS1-RS***. A structural comparison of ***TS1-SR*** and ***TS1-RS*** is shown in Figure S4b. The H-bond length of NH···O=C is 1.87 Å in ***TS1-SR***, and it is 1.94 Å in ***TS1-RS***. This shorter hydrogen bond in ***TS1-SR*** indicates that the hydrogen bond in ***TS1-SR*** is stronger than in ***TS1-RS***, which partly contributes to the stronger orbital interaction in ***TS1-SR***. In addition, the stronger hydrogen bond in ***TS1-SR*** shortens the distance between the imine anion and the MBH acetate intermediate, leading to better orbital overlap and enhanced orbital interaction.

The EDA-NOCV method involves the decomposition of the intrinsic interaction energy (ΔE_{int}) of transition states into electrostatic energy(ΔE_{elstat}), Pauli repulsion energy(ΔE_{Pauli}), orbital interaction energy(ΔE_{orb}) and dispersion energy(ΔE_{disp}). The energy components of four representative transition states (***TS1-SR, TS1-RS, TS1-RR* and *TS1-SS***) are summarized in Table S5. Among them, ***TS1-SR*** exhibits the most favorable interaction energy (ΔE_{int}= - 90.5 kcal/mol), primarily driven by strong orbital interactions (ΔE_{orb}= -108.0 kcal/mol). Electrostatic interactions also contribute significantly to the stabilization (ΔE_{elstat} = -96.5 kcal/mol). Although Pauli repulsion is inherently destabilizing (ΔE_{Pauli} = 158.6 kcal/mol), its effect is effectively counterbalanced by the more stabilizing orbital and electrostatic components in this configuration. Dispersion interactions provide a moderate contribution (ranging from - 42.4 to - 44.6 kcal/mol) with minimal variation across the transition states.

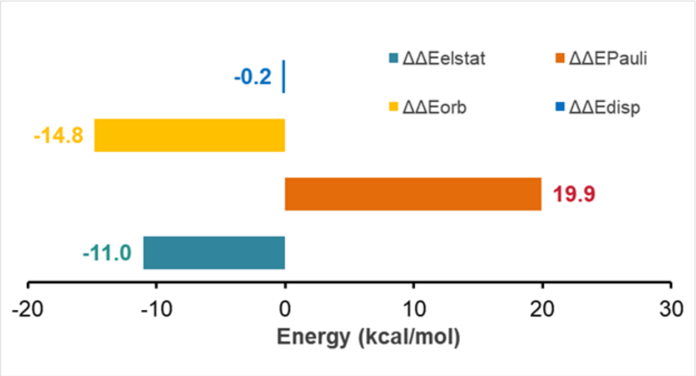

Figure S3. The EDA results for ***TS1-SR*** and ***TS1-RS***.

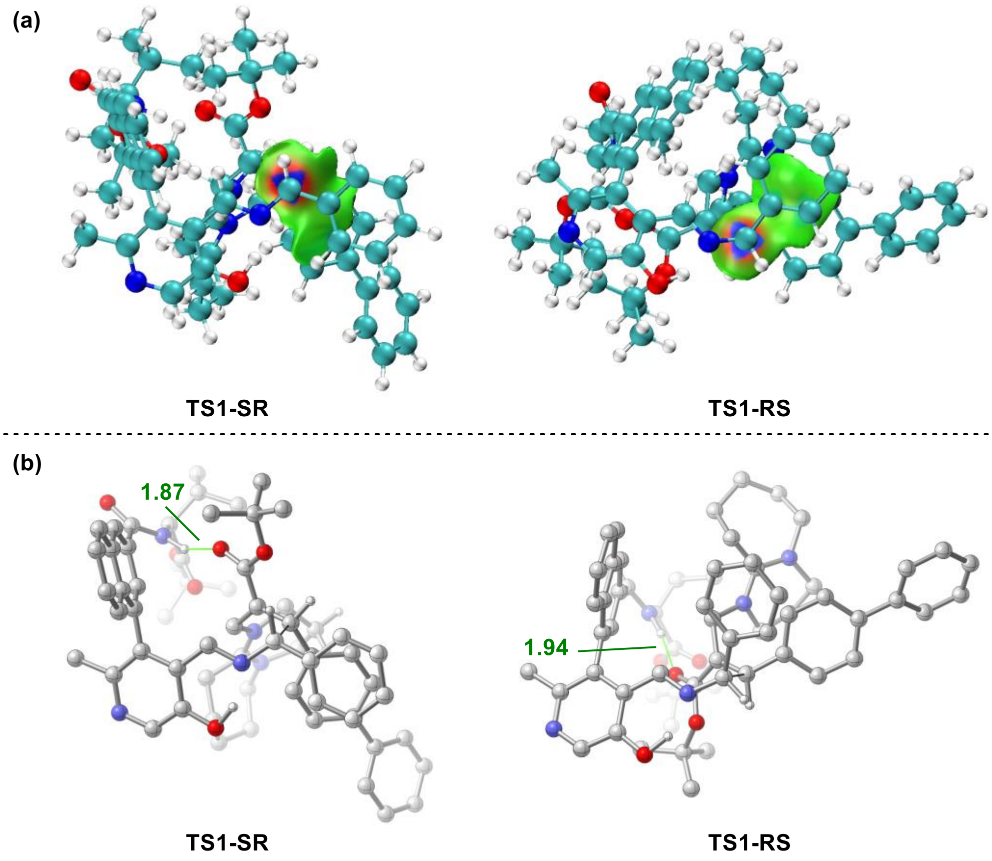

Figure S4. (a) NCI-plots of ***TS1-SR*** and ***TS1-RS***; (b) Geometries of ***TS1-SR*** and ***TS1-RS***.

Table S5. Energy decomposition analysis of four representative transition states. The unit of energy is kcal/mol.

Geometry	ΔE_{int}	ΔE_{elstat}	ΔE_{Pauli}	ΔE_{orb}	ΔE_{disp}
**TS1-SR**	-90.5	-96.5	158.6	-108.0	-44.6
**TS1-RS**	-84.5	-85.5	138.7	-93.2	-44.4
**TS1-RR**	-79.1	-77.3	125.2	-84.7	-42.4
**TS1-SS**	-86.6	-77.1	123.5	-88.7	-44.3

Table S6. Energies (Hartree) of related structures in Figure S2.

Geometry	H	G
**TS1-SR**	-3170.106228	-3170.24112
**TS1-RS**	-3170.089196	-3170.222922
**TS1-RR**	-3170.098597	-3170.23132
**TS1-SS**	-3170.096845	-3170.230337
**TS2-SR**	-3170.083554	-3170.217263
**TS2-RS**	-3170.078296	-3170.210569
**TS2-RR**	-3170.08476	-3170.220187
**TS2-SS**	-3170.078173	-3170.211979

Table S7. Cartesian coordinates of the TS structures in Figure S2. All of these species have only one imaginary frequency.

Geometry	Cartesian coordinates			
**TS1-SR**	C	4.18780100	-5.28821000	2.00518300
	C	3.43613800	-4.15640400	2.22985900
	C	3.81654400	-2.90826500	1.67034700
	C	5.01333400	-2.84199500	0.88044100
	C	5.76709700	-4.02805100	0.67370100
	C	5.36324600	-5.22543700	1.21854300
	H	3.87943900	-6.23649000	2.43410000
	H	2.53735400	-4.20509900	2.83451200
	C	3.05347700	-1.71540900	1.87099800
	C	5.41207700	-1.60444400	0.31370900
	H	6.66902000	-3.97140200	0.07113800
	H	5.94558900	-6.12559000	1.04906700
	C	4.63244300	-0.48100400	0.47220800
	C	3.45965400	-0.54805800	1.26450800
	H	6.32277300	-1.55321200	-0.27491100
	H	2.88556500	0.35781200	1.42144800
	C	1.86547500	-1.66900300	2.77306900
	C	0.60428100	-2.15506400	2.35843700
	C	1.99852000	-1.06098800	4.03445700
	C	-0.48442100	-1.97054700	3.25631600
	C	-0.24750200	-1.33241000	4.47505600
	H	-1.08381800	-1.18727300	5.15628000
	C	3.33208700	-0.55540600	4.53093700
	H	4.12735300	-1.28833200	4.36579600
	H	3.63658100	0.36289600	4.01692300
	H	3.25793600	-0.34142900	5.59848300
	N	0.95317300	-0.89530900	4.86522600
	O	-1.73414500	-2.38141700	2.96098600
	H	-1.65326500	-2.79816700	2.03181800
	C	0.38610400	-2.76083600	1.05658000
	H	1.23400800	-2.90008500	0.38812600
	N	-0.82470100	-3.15400500	0.72644600
	C	-1.11879400	-3.46367600	-0.57126900
	C	5.01415200	0.82801700	-0.15516500

	O	6.17760800	1.24440900	-0.17842800
	N	3.94790400	1.50932100	-0.64245800
	H	3.09005300	0.98564600	-0.81463500
	C	4.02207300	2.87385400	-1.10691000
	H	4.95127100	3.30172100	-0.72356100
	C	4.04456700	2.96987400	-2.66262000
	H	4.79689800	2.22802700	-2.95705100
	C	2.70247500	2.56887200	-3.28688800
	H	2.79522100	2.51320100	-4.37596100
	H	2.35967400	1.59621700	-2.92428700
	H	1.92942900	3.31372600	-3.06288800
	C	4.50960100	4.34135500	-3.16153000
	H	5.47014000	4.62267700	-2.71798700
	H	4.63140800	4.32167400	-4.24909100
	H	3.78549300	5.12216700	-2.91546200
	C	2.83950600	3.63116000	-0.50111400
	O	1.83178100	3.08296500	-0.08826900
	O	3.05720600	4.94865600	-0.51732200
	C	2.03708100	5.91470200	-0.04749900
	C	0.76678200	5.77419700	-0.88518000
	C	1.78165500	5.69765200	1.44349900
	C	2.71141600	7.26051900	-0.29840800
	H	1.00112600	5.85874500	-1.95017900
	H	0.28212500	4.81725500	-0.70066400
	H	0.07255500	6.57796400	-0.62292100
	H	2.71733000	5.76768700	2.00494000
	H	1.10265300	6.47254300	1.81100300
	H	1.33525300	4.72037600	1.62513500
	H	2.05382800	8.07036600	0.02850900
	H	3.65099000	7.32910100	0.25599100
	H	2.92344900	7.39273400	-1.36278300
	C	0.00380100	0.19791800	-0.01098300
	C	-0.05520700	-0.86359300	-1.05589000
	C	1.17006200	-1.10844300	-1.75562900
	O	2.20907500	-0.42834000	-1.66548500
	O	1.10666200	-2.20993800	-2.56026500
	C	2.27729400	-2.71867100	-3.27402900
	C	1.71862100	-3.94452200	-3.99758100
	H	1.32090900	-4.66574300	-3.27769000
	H	0.91365000	-3.65465500	-4.67935400
	H	2.50711700	-4.43200800	-4.57779400
	C	3.35714600	-3.12875700	-2.27144700
	H	4.18612200	-3.61397600	-2.79627800
	H	3.73758600	-2.26242700	-1.73423900
	H	2.95205900	-3.84071900	-1.54568000
	C	2.78554900	-1.68607100	-4.28374200
	H	3.20927100	-0.82125500	-3.77552200
	H	3.55205600	-2.14125900	-4.91883000
	H	1.96410100	-1.35328900	-4.92602800
	C	-1.23244500	-1.58838500	-1.41523300
	H	-1.19677300	-2.00212600	-2.41905300
	H	1.04624800	0.45147600	0.17067800
	C	-2.60105900	3.01447300	3.43151100
	C	-3.19857400	3.88809800	2.32246000
	C	-2.15965700	4.42049000	1.32760900
	C	-1.82096200	1.79058400	2.94201000
	C	-0.63701300	2.14097600	2.01371100
	C	-1.05277100	2.35275100	0.58076300
	H	-1.92918100	3.63490600	4.03955500
	H	-3.96436100	3.33435100	1.76517800

	H	-1.26150100	4.76799000	1.84560800
	H	-2.49037500	1.09447700	2.42656200
	H	0.12303300	1.36798800	2.06201900
	H	-3.40428300	2.68306600	4.09822400
	H	-3.70081400	4.74703900	2.78039100
	H	-2.55630300	5.28143300	0.78772100
	H	-1.41834700	1.25181900	3.80431200
	H	-0.14767400	3.05593800	2.36078100
	C	-2.43781100	3.65146600	-1.01420600
	H	-1.96385900	4.51760900	-1.48871300
	H	-3.48352500	3.90795700	-0.82142400
	C	-2.34216700	2.41806900	-1.89483700
	H	-2.54416500	2.69348800	-2.93255000
	H	-3.08074800	1.67269100	-1.59305900
	C	-0.94963400	1.82115500	-1.76055100
	H	-0.17585300	2.52705800	-2.08275600
	H	-0.84128700	0.90565500	-2.33762700
	N	-1.78758800	3.43794200	0.29329300
	N	-0.71050900	1.48372000	-0.35670300
	H	-0.43508100	-0.14365100	0.92004000
	H	-0.28160100	-3.75892200	-1.20724400
	C	-2.40529400	-4.09020100	-0.89947300
	C	-2.58412900	-4.63798000	-2.18247500
	C	-3.50997700	-4.06661000	-0.02922700
	C	-3.82077100	-5.13772100	-2.58537900
	H	-1.74016200	-4.66129100	-2.86758900
	C	-4.74519300	-4.56630200	-0.43327800
	H	-3.40140000	-3.64095400	0.96110800
	C	-4.91107500	-5.10333000	-1.71293300
	H	-3.93359000	-5.55610100	-3.58155500
	H	-5.58586100	-4.53330500	0.25394700
	H	-5.87624200	-5.49047200	-2.02470200
	C	-2.58692100	-1.10110300	-1.06807800
	C	-2.97714100	-0.73240000	0.23024900
	C	-3.55710100	-1.01466800	-2.07966200
	C	-4.25317400	-0.24778300	0.48947100
	H	-2.29378700	-0.85862500	1.05758800
	C	-4.83522500	-0.52844300	-1.82355200
	H	-3.29849900	-1.33320700	-3.08490800
	C	-5.20551200	-0.11478400	-0.53411500
	H	-4.51120600	0.04894200	1.50140800
	H	-5.56117500	-0.48504200	-2.62920800
	C	-6.55089000	0.43875500	-0.25986600
	C	-7.20739600	0.17724900	0.95450600
	C	-7.20100200	1.24832600	-1.20642900
	C	-8.46960300	0.70820300	1.21364100
	H	-6.73468500	-0.46708400	1.68883900
	C	-8.46450900	1.77703100	-0.94874000
	H	-6.70021900	1.48225300	-2.14065200
	C	-9.10448300	1.51037400	0.26324800
	H	-8.96215300	0.48708900	2.15568400
	H	-8.94607100	2.40538000	-1.69191900
	H	-10.08812200	1.92283100	0.46481900
**TS1-RS**	C	1.20224720	-0.12359550	0.00000000
	C	2.05026120	0.39815650	0.95090900
	C	2.42450220	1.76849450	0.92053500
	C	1.92589120	2.59243450	-0.14312900
	C	1.05032920	2.02246950	-1.10710400
	C	0.68998120	0.69658950	-1.03524900
	H	0.91880120	-1.17019850	0.04425800

	H	2.43470620	-0.22930250	1.74700600
	C	3.25398520	2.35843050	1.92595900
	C	2.30792020	3.95469450	-0.20465600
	H	0.67202420	2.65702750	-1.90353400
	H	0.01882020	0.27208450	-1.77507300
	C	3.10547120	4.50440450	0.77705800
	C	3.57400120	3.69712550	1.84096300
	H	1.95858720	4.57491750	-1.02404300
	H	4.16967020	4.14478050	2.62502900
	C	3.72438120	1.58913550	3.11456500
	C	2.80948320	1.27859250	4.14473000
	C	5.07603720	1.23672050	3.25649500
	C	3.32091420	0.64770250	5.30673700
	C	4.68172220	0.33586150	5.34572600
	H	5.07969920	-0.15716150	6.23073900
	C	6.08690520	1.53908450	2.17680100
	H	5.70488920	1.28418150	1.18366600
	H	6.34805820	2.60322750	2.15339200
	H	6.99854220	0.96977150	2.36680300
	N	5.53780620	0.61307550	4.35578800
	O	2.53370120	0.35021150	6.35901300
	H	1.60620620	0.67511350	6.08991200
	C	1.39784620	1.58545250	4.02304200
	H	1.06857420	2.02007450	3.09325300
	N	0.57510820	1.28087950	4.99788700
	C	-0.75960880	1.58471950	4.97519700
	H	-1.24219980	1.37075850	5.92819300
	C	-1.67718880	1.36652050	3.84292600
	C	-3.05500080	1.29289550	4.14128700
	C	-1.31179280	1.23985850	2.48531500
	C	-4.01598080	1.14507250	3.14697900
	H	-3.36850580	1.37622850	5.17780000
	C	-2.27590180	1.09312550	1.48898600
	H	-0.27541080	1.23109050	2.18497000
	C	-3.63556280	1.05798950	1.80582400
	H	-5.06652180	1.10649350	3.42038600
	H	-1.95008980	1.00297050	0.45660900
	H	-4.38246880	0.95242250	1.02512800
	C	3.47528620	5.95402150	0.68788800
	O	3.81556920	6.45625250	-0.39073300
	N	3.40577420	6.65450550	1.84789500
	H	3.06552620	6.20722850	2.70410100
	C	3.88214320	8.03118250	1.90822100
	H	4.89875620	8.06904750	1.50639200
	C	3.01678120	9.01679350	1.06288100
	H	3.05735120	8.60139450	0.05341700
	C	1.55985120	9.04735150	1.51987600
	H	0.94566520	9.60506150	0.80568800
	H	1.15577120	8.03533250	1.60266800
	H	1.46504520	9.52803750	2.49768000
	C	3.63068520	10.41981750	1.03249700
	H	4.68037120	10.39194850	0.72377300
	H	3.08660020	11.05136450	0.32360900
	H	3.58394520	10.90465350	2.01277200
	C	3.91952120	8.46767350	3.36672200
	O	3.04468020	8.20271950	4.17178800
	O	5.00396020	9.21071950	3.60240700
	C	5.24697820	9.84922750	4.91816200
	C	4.10843120	10.81474350	5.24589200
	C	5.42342620	8.76756750	5.98273400

	C	6.55253220	10.60361550	4.68003300
	H	3.98217020	11.54409050	4.44044000
	H	3.16952620	10.28274550	5.39659800
	H	4.35458520	11.35980650	6.16181000
	H	6.18480820	8.04756750	5.66944300
	H	5.75517820	9.23216250	6.91591500
	H	4.49063120	8.23724150	6.16777800
	H	6.84844320	11.12855150	5.59213800
	H	7.35168220	9.91120450	4.40228900
	H	6.43225320	11.33766950	3.87873300
	C	-0.58199680	3.74595150	5.18717900
	C	0.46443320	4.22321150	4.35582700
	C	1.78608420	4.47537450	4.86548700
	O	2.64957720	5.14783050	4.26952300
	O	2.01777120	3.94239750	6.09356500
	C	3.16302720	4.35574750	6.90557600
	C	2.99464220	3.50229450	8.16302000
	H	2.03999520	3.72281050	8.65027500
	H	3.01277320	2.44130450	7.89969900
	H	3.80201220	3.70860550	8.87156900
	C	3.03310920	5.84711450	7.22305300
	H	3.83739520	6.15776750	7.89733300
	H	3.09146220	6.43715150	6.30756200
	H	2.07633020	6.04765350	7.71567400
	C	4.49650020	4.03422050	6.22393100
	H	4.65521520	4.67079650	5.35585100
	H	5.30977820	4.19166450	6.94025900
	H	4.51888320	2.99038050	5.90600000
	C	0.24044720	4.69190950	2.95314100
	H	-0.34521180	3.95741350	2.40946800
	H	1.19561820	4.81144050	2.44970000
	H	-0.27910380	3.64939150	6.22401600
	C	-1.72066380	7.28808850	-1.22682900
	C	-2.39978980	8.28595850	-0.28413800
	C	-3.14585180	7.62191550	0.87816100
	C	-0.70112180	6.35851850	-0.56337700
	C	-1.27221080	5.50039850	0.59115500
	C	-1.35414580	6.26860250	1.88781600
	H	-2.49857580	6.67165450	-1.69667600
	H	-1.66940480	8.99289950	0.12721500
	H	-3.70290580	6.74461850	0.53361700
	H	0.16112720	6.92564850	-0.19574300
	H	-0.62535780	4.63872150	0.71958100
	H	-1.22712080	7.83465550	-2.03744400
	H	-3.12440380	8.87474350	-0.85667900
	H	-3.87938180	8.30874550	1.29861800
	H	-0.31519580	5.66681450	-1.31844000
	H	-2.26299180	5.11741450	0.32749900
	C	-2.40146880	7.97172850	3.26743700
	H	-3.00272680	7.37162850	3.95905100
	H	-2.93229680	8.90091350	3.06252100
	C	-1.01551580	8.24002450	3.82880700
	H	-1.08737980	8.74698150	4.79400000
	H	-0.47770580	8.89889750	3.14571200
	C	-0.28713280	6.92096750	4.01473400
	H	-0.64229280	6.42019950	4.91518100
	H	0.79220020	7.06894750	4.10610400
	N	-2.26698380	7.23911750	1.99821300
	N	-0.49830480	6.00755150	2.87143800
	C	-1.98212980	4.21167550	5.05557600

	C	-2.63340280	4.70262450	6.20247000
	C	-2.70904980	4.24360350	3.85125800
	C	-3.90902880	5.25433350	6.14100100
	H	-2.11513880	4.66545950	7.15661900
	C	-3.98270080	4.79238250	3.78591700
	H	-2.28732080	3.81849550	2.95187400
	C	-4.60701580	5.32654550	4.92394400
	H	-4.37626880	5.62052450	7.04967800
	H	-4.49374680	4.82366950	2.82904200
	C	-5.94879480	5.94560050	4.83781300
	C	-6.27410180	7.06937450	5.61583300
	C	-6.92605780	5.43334950	3.96785900
	C	-7.53325180	7.66035750	5.52761700
	H	-5.52536880	7.49455150	6.27679300
	C	-8.18427280	6.02567050	3.87754600
	H	-6.70381280	4.54994650	3.37767600
	C	-8.49404680	7.14208250	4.65704200
	H	-7.76135780	8.53221650	6.13326700
	H	-8.92734080	5.60884350	3.20442100
	H	-9.47447780	7.60271150	4.58756800
**TS1-RR**	C	-0.60112360	0.85393257	0.00000000
	C	0.17863540	0.93814657	-1.13133500
	C	0.74932740	-0.22668643	-1.71055700
	C	0.51264540	-1.49448643	-1.08058200
	C	-0.29689560	-1.54514543	0.08719300
	C	-0.84541360	-0.39906843	0.61417500
	H	-1.03250260	1.75341257	0.42780300
	H	0.36546740	1.89727457	-1.60120800
	C	1.54315940	-0.17906843	-2.89864100
	C	1.08753640	-2.66395243	-1.63476000
	H	-0.47139260	-2.50880543	0.55728500
	H	-1.46302360	-0.44957243	1.50529700
	C	1.83073640	-2.59937743	-2.79502300
	C	2.06041040	-1.34865343	-3.41578600
	H	0.93472640	-3.62244543	-1.14954300
	H	2.63652240	-1.30487443	-4.33011400
	C	1.82863840	1.09575357	-3.61850900
	C	0.83506840	1.69197757	-4.42832100
	C	3.11545940	1.65696757	-3.56169000
	C	1.22697740	2.82007457	-5.20090800
	C	2.53049740	3.29792357	-5.07017000
	H	2.82432040	4.17052457	-5.65069000
	C	4.19714340	1.06056757	-2.69293800
	H	3.82568040	0.82002757	-1.69215700
	H	4.59156540	0.13030257	-3.11713000
	H	5.02051240	1.77189357	-2.60798800
	N	3.45337840	2.74768657	-4.27178600
	O	0.37655740	3.44089257	-6.04562800
	H	-0.48893060	2.91368057	-5.97566600
	C	-0.52276560	1.19443457	-4.48453200
	H	-0.84091960	0.45075957	-3.75718900
	N	-1.38532960	1.74707257	-5.31329300
	C	-2.64673760	1.27583057	-5.41279300
	H	-3.07786260	0.77936157	-4.54196800
	C	-3.58843560	1.89845157	-6.33820100
	C	-4.97455960	1.72071357	-6.16724000
	C	-3.14668160	2.60853957	-7.47299300
	C	-5.88352060	2.24384757	-7.08390500
	H	-5.33201160	1.15826057	-5.30968000
	C	-4.05811460	3.13630257	-8.38288500

	H	-2.08066260	2.72547057	-7.63822000
	C	-5.43308760	2.95969457	-8.19589400
	H	-6.94821360	2.09322857	-6.92871000
	H	-3.69481460	3.68291557	-9.24881800
	H	-6.14131360	3.36954357	-8.90943200
	C	2.38962240	-3.86737443	-3.36551000
	O	2.85399440	-4.74605743	-2.62820300
	N	2.33526540	-3.98384043	-4.71691300
	H	1.88963140	-3.25953843	-5.28611400
	C	2.94319440	-5.12739143	-5.38668100
	H	3.98218940	-5.21674743	-5.05705100
	C	2.23825440	-6.47999243	-5.05807800
	H	2.34468240	-6.57714443	-3.97525000
	C	0.74963040	-6.45980843	-5.40002300
	H	0.26206540	-7.36741743	-5.02983500
	H	0.25488940	-5.59707843	-4.94643400
	H	0.59473940	-6.40623743	-6.48124900
	C	2.95485440	-7.65917443	-5.72374500
	H	4.02801640	-7.65053843	-5.50945800
	H	2.54371340	-8.60394343	-5.35552800
	H	2.83150540	-7.64626743	-6.81152000
	C	2.93028640	-4.86954843	-6.88776900
	O	1.97086740	-4.41235343	-7.48308100
	O	4.08305540	-5.26020043	-7.43723000
	C	4.31595640	-5.20294543	-8.90022300
	C	3.29377240	-6.07760343	-9.62537800
	C	4.27613840	-3.74742743	-9.36291900
	C	5.72212640	-5.78260143	-9.03070700
	H	3.32033840	-7.09919443	-9.23525400
	H	2.28514640	-5.67976443	-9.51747300
	H	3.54545540	-6.11387543	-10.68932200
	H	4.95742940	-3.13933543	-8.76101700
	H	4.59939140	-3.69232543	-10.40655000
	H	3.27177040	-3.33409243	-9.28567900
	H	6.02028740	-5.79813143	-10.08233100
	H	6.44146140	-5.17610243	-8.47399700
	H	5.75403840	-6.80487143	-8.64431300
	C	-1.06968160	-2.23809343	-4.77208300
	C	-1.06394560	-1.21977543	-5.87113900
	C	0.22497340	-1.00827243	-6.49070100
	O	1.23215440	-1.70091843	-6.26459900
	O	0.23326040	0.00956857	-7.38383200
	C	1.33679440	0.20042757	-8.32803000
	C	0.91145240	1.46935457	-9.06708100
	H	-0.06114160	1.32467957	-9.54702800
	H	0.83194940	2.30101557	-8.36159900
	H	1.64574440	1.72367857	-9.83684300
	C	1.38997540	-1.00615343	-9.26708900
	H	2.16385340	-0.85643443	-10.02638500
	H	1.61721140	-1.91404643	-8.70627000
	H	0.42947140	-1.13336843	-9.77626800
	C	2.67362540	0.42774657	-7.61593300
	H	3.02576440	-0.48407743	-7.13792200
	H	3.41449940	0.75805857	-8.35156700
	H	2.57397440	1.20752157	-6.85841900
	C	-2.23466360	-0.65910843	-6.41208300
	H	-2.09383360	-0.16240943	-7.36562800
	H	-0.04777860	-2.46606543	-4.48802500
	C	-2.54171960	-6.43828343	-1.87700500
	C	-3.27314660	-7.01660443	-3.09179100

	C	-4.13973260	-5.98987643	-3.82823700
	C	-1.62237860	-5.25156043	-2.17686400
	C	-2.33102360	-4.03695443	-2.82535500
	C	-2.50090660	-4.21615543	-4.31310500
	H	-3.29100060	-6.11962243	-1.14010400
	H	-2.56275860	-7.45212443	-3.80462600
	H	-4.69077460	-5.36697243	-3.11628800
	H	-0.78487760	-5.55880443	-2.81349800
	H	-1.72995360	-3.15514043	-2.62868100
	H	-1.95427460	-7.22907443	-1.39863700
	H	-3.92587860	-7.82991043	-2.75728600
	H	-4.88533260	-6.49233743	-4.44364600
	H	-1.18342460	-4.90483243	-1.23688200
	H	-3.30716860	-3.86987143	-2.35998600
	C	-3.57327560	-5.30852943	-6.19486600
	H	-5.75880660	-2.53912243	-3.96560400
	H	-4.05689160	-6.27237143	-6.34885600
	C	-2.21678060	-5.25279943	-6.87841700
	H	-2.32719360	-5.36487843	-7.95943800
	H	-1.60281560	-6.07980043	-6.51641100
	C	-1.57505460	-3.91001743	-6.58082900
	H	-2.02624060	-3.13648043	-7.20111900
	H	-0.49902560	-3.91836443	-6.77791900
	N	-3.37659960	-5.12649643	-4.74827600
	N	-1.74723860	-3.52265543	-5.16368500
	H	-1.58256060	-1.85003243	-3.89673600
	C	-3.57505360	-1.25844943	-6.25498300
	C	-4.36047260	-1.47495243	-7.39979300
	C	-4.12882460	-1.63851043	-5.01886500
	C	-5.59722560	-2.10881543	-7.32508000
	H	-3.97553460	-1.16563443	-8.36668300
	C	-5.36241160	-2.26964643	-4.93939300
	H	-3.59743560	-1.41980443	-4.10001700
	C	-6.11615760	-2.54183343	-6.09464900
	H	-6.15219860	-2.30021043	-8.23787800
	H	-4.23940660	-4.52087143	-6.56389900
	C	-7.40233360	-3.26895543	-6.01254500
	C	-8.46717660	-2.96340843	-6.87626300
	C	-7.58356260	-4.29657043	-5.07126400
	C	-9.67031260	-3.66291843	-6.80238200
	H	-8.35786260	-2.15629743	-7.59356300
	C	-8.78713460	-4.99463943	-4.99546300
	H	-6.76661560	-4.56235243	-4.40861700
	C	-9.83628160	-4.68179943	-5.86203200
	H	-10.48308960	-3.40557643	-7.47467300
	H	-8.90263060	-5.78958043	-4.26486400
	H	-10.77386360	-5.22567243	-5.80467300
**TS1-SS**	C	5.71118800	-2.79744400	2.17512100
	C	4.59852900	-2.04236800	2.47385900
	C	4.38577300	-0.78291700	1.85313100
	C	5.37284300	-0.29112800	0.93429800
	C	6.51385900	-1.09129100	0.65838700
	C	6.67649000	-2.32056300	1.25529700
	H	5.85219300	-3.76396400	2.64849400
	H	3.86127700	-2.40664000	3.18053100
	C	3.21652200	0.00347700	2.09785600
	C	5.17833900	0.96180200	0.29748600
	H	7.25244800	-0.71646500	-0.04423500
	H	7.54786300	-2.92640200	1.02785200
	C	4.01990200	1.67568100	0.50501000

	C	3.05200700	1.18861900	1.41662100
	H	5.92852400	1.33481700	-0.39278400
	H	2.15831900	1.77656900	1.59221800
	C	2.14870500	-0.39447600	3.06054800
	C	1.23319100	-1.42296400	2.73714600
	C	1.98678000	0.33715800	4.25099700
	C	0.14975300	-1.62518800	3.63786900
	C	0.07412300	-0.83296100	4.78331900
	H	-0.75187000	-0.99555500	5.47349600
	C	2.95919900	1.42101400	4.65275900
	H	3.99604200	1.11762500	4.48171500
	H	2.80039100	2.34333800	4.08276900
	H	2.82150400	1.64922500	5.71124700
	N	0.96499200	0.11471400	5.09668700
	O	-0.80695100	-2.54947700	3.40646400
	H	-0.50129700	-3.01658700	2.54575600
	C	1.35869700	-2.22436000	1.53427200
	H	2.18949200	-2.02625300	0.86956800
	N	0.46283700	-3.16513600	1.30591300
	C	0.36133000	-3.89529000	0.16811700
	C	3.75122000	2.96075500	-0.22119700
	O	4.61755500	3.82015700	-0.41529200
	N	2.45333400	3.08850800	-0.59374500
	H	1.89200200	2.24179000	-0.64776500
	C	1.89341800	4.30387100	-1.13504300
	H	2.49261200	5.14143300	-0.76986400
	C	1.91700300	4.33027400	-2.69383100
	H	2.93753000	4.01510300	-2.94202400
	C	0.93574000	3.32066900	-3.30032900
	H	1.09208400	3.24183400	-4.38048700
	H	1.05981100	2.32582900	-2.86547000
	H	-0.10125500	3.63988300	-3.14191000
	C	1.70228700	5.73704500	-3.25960300
	H	2.41474300	6.45072500	-2.83340200
	H	1.83944700	5.72944200	-4.34547900
	H	0.69406100	6.10262500	-3.04769700
	C	0.47317200	4.42385100	-0.58406800
	O	-0.16621800	3.46882200	-0.17570700
	O	0.03814400	5.68382900	-0.64604600
	C	-1.33718200	6.05451500	-0.23591700
	C	-2.35214300	5.30436700	-1.09725900
	C	-1.51442900	5.77221700	1.25559100
	C	-1.37749300	7.55312200	-0.52036100
	H	-2.15170500	5.47416800	-2.15897000
	H	-2.31907600	4.23573800	-0.89317100
	H	-3.35681500	5.67586200	-0.87501400
	H	-0.74878100	6.29373800	1.83642000
	H	-2.49507400	6.13402000	1.57805800
	H	-1.44570300	4.70440800	1.46185700
	H	-2.35482800	7.95621900	-0.24212700
	H	-0.60860300	8.07477500	0.05546600
	H	-1.21045200	7.74806000	-1.58309900
	C	-0.36789400	-0.09701000	0.18360900
	C	0.15249300	-1.08207900	-0.81098700
	C	1.30864300	-0.63775600	-1.55859000
	O	1.82806700	0.48205600	-1.43209000
	O	1.75961900	-1.54454600	-2.45576300
	C	2.94200500	-1.29520200	-3.28746100
	C	2.99674500	-2.53996500	-4.17190400
	H	3.07720700	-3.44166800	-3.56314900

	H	2.09405600	-2.61254300	-4.78631000
	H	3.86346600	-2.48476300	-4.83711100
	C	4.17792100	-1.20372200	-2.39349900
	H	5.07800600	-1.13080600	-3.01249400
	H	4.12321400	-0.32772200	-1.74959600
	H	4.25863400	-2.09776300	-1.77034300
	C	2.75644300	-0.04199900	-4.14793300
	H	2.80409200	0.86359700	-3.54669700
	H	3.54316800	-0.00898300	-4.90833900
	H	1.79011300	-0.07563800	-4.66119800
	C	-0.48386300	-2.29726000	-1.12471200
	H	-0.11071700	-2.77873800	-2.02131600
	H	0.43236900	0.59077900	0.45039600
	C	-4.18045800	1.37175100	3.28025500
	C	-5.02589700	1.83745200	2.08920200
	C	-4.27129900	2.75332400	1.11732100
	C	-2.89482900	0.62511400	2.91147200
	C	-1.93449900	1.44578000	2.02228100
	C	-2.29868300	1.39304800	0.56110200
	H	-3.91123000	2.25105300	3.88063500
	H	-5.41187800	0.97643500	1.52932900
	H	-3.66771600	3.48793700	1.65703400
	H	-3.13135100	-0.31958100	2.41121000
	H	-0.91296100	1.10344300	2.15616100
	H	-4.79228400	0.73224700	3.92542500
	H	-5.89648700	2.38521200	2.46554400
	H	-4.97302600	3.32147400	0.50513200
	H	-2.35612500	0.36362000	3.82622600
	H	-1.93897100	2.49447300	2.33478300
	C	-4.00755500	1.84775100	-1.17668700
	H	-3.94915700	2.81226200	-1.69241100
	H	-5.06573100	1.60509200	-1.04269600
	C	-3.30414400	0.75614100	-1.96322400
	H	-3.53588500	0.86354600	-3.02542800
	H	-3.64692500	-0.22895700	-1.64122900
	C	-1.80478100	0.86177800	-1.73000300
	H	-1.40955800	1.82529200	-2.06905200
	H	-1.26371000	0.06712600	-2.23774500
	N	-3.42033300	2.01396700	0.16718400
	N	-1.53617700	0.73365600	-0.29677300
	H	-0.70694100	-0.60068800	1.07995200
	C	-1.93419200	-2.50302800	-0.90422800
	C	-2.58501000	-2.31687200	0.32810700
	C	-2.73317600	-2.84245300	-2.01006500
	C	-3.96895700	-2.39090200	0.42840200
	H	-2.00640800	-2.13107800	1.22273800
	C	-4.11959200	-2.91646900	-1.91035700
	H	-2.25639200	-3.02418400	-2.96912900
	C	-4.77099400	-2.66564300	-0.69211300
	H	-4.43919700	-2.20768600	1.38963800
	H	-4.70407700	-3.17810300	-2.78673100
	C	-6.24779800	-2.67883800	-0.59031800
	C	-6.89120300	-3.15353400	0.56494800
	C	-7.04264600	-2.19987300	-1.64537900
	C	-8.28148900	-3.14871900	0.66177800
	H	-6.29756400	-3.55082700	1.38203200
	C	-8.43300700	-2.19758100	-1.54977900
	H	-6.56399800	-1.80509600	-2.53604200
	C	-9.05912200	-2.67121100	-0.39510000
	H	-8.75840200	-3.52714000	1.56078700

	H	-9.02784400	-1.81683100	-2.37451200
	H	-10.14196100	-2.66841700	-0.31965800
	H	-0.51397300	-4.54187800	0.15952500
	C	1.48063600	-4.43738700	-0.62224900
	C	1.16535300	-5.24608300	-1.73462600
	C	2.84224100	-4.28221500	-0.29797000
	C	2.15708700	-5.85758300	-2.49320400
	H	0.11983300	-5.39639700	-1.99231800
	C	3.83554600	-4.89994800	-1.05692700
	H	3.13930700	-3.71022200	0.57067200
	C	3.50544800	-5.68661100	-2.16152200
	H	1.88016200	-6.47329600	-3.34425600
	H	4.87574600	-4.76344700	-0.77421000
	H	4.28212900	-6.16129800	-2.75288600
**TS2-SR**	C	7.22447200	-2.44393900	-3.11264100
	C	6.14338600	-2.38313700	-2.26192200
	C	5.74718200	-1.15425300	-1.66969700
	C	6.48245100	0.03144300	-2.00324900
	C	7.59734900	-0.06745100	-2.87943000
	C	7.96563900	-1.27740000	-3.41963700
	H	7.50963300	-3.39288100	-3.55601500
	H	5.57450900	-3.27902300	-2.04047300
	C	4.63981300	-1.04882900	-0.76568800
	C	6.07362300	1.27937200	-1.47422100
	H	8.14932000	0.83680200	-3.11910800
	H	8.81800700	-1.34099100	-4.08856700
	C	4.96297200	1.37192900	-0.66308100
	C	4.26307100	0.19432900	-0.29908500
	H	6.62571700	2.17922500	-1.72446900
	H	3.41894600	0.26506800	0.37424600
	C	3.89480200	-2.27274400	-0.34587600
	C	2.56538800	-2.49330000	-0.77785000
	C	4.51518600	-3.23884100	0.46961900
	C	1.96710100	-3.73898500	-0.46124100
	C	2.67426700	-4.63040000	0.34646900
	H	2.20758400	-5.57427900	0.62125100
	C	5.90476300	-3.03843900	1.02320100
	H	6.07421800	-2.00048100	1.32138400
	H	6.04192600	-3.68902500	1.88866100
	H	6.67289400	-3.28966500	0.28329900
	N	3.89960000	-4.38410600	0.81708000
	O	0.73426800	-4.06664200	-0.90230800
	H	0.42235300	-3.26075200	-1.43361600
	C	1.78108000	-1.46777700	-1.44454400
	H	2.20159900	-0.46449500	-1.50871800
	N	0.59911000	-1.71820300	-1.92685500
	C	-0.21631500	-0.65043600	-2.28059000
	H	0.30394400	0.21612600	-2.69829600
	C	-1.46822800	-0.98907200	-2.98867400
	C	-2.14613700	0.00331300	-3.71621400
	C	-2.07353900	-2.25295800	-2.88130100
	C	-3.39641800	-0.24137600	-4.27702000
	H	-1.69659100	0.98664000	-3.81196900
	C	-3.31833900	-2.50429900	-3.45469600
	H	-1.56954900	-3.04326600	-2.33659600
	C	-3.99473800	-1.49676700	-4.14596100
	H	-3.90819200	0.55137800	-4.81443100
	H	-3.76433400	-3.48982700	-3.35445600
	H	-4.96980500	-1.68870700	-4.58325100
	C	4.54095900	2.73668900	-0.19158400

	O	5.36357700	3.65566700	-0.08733500
	N	3.21468900	2.88917300	0.04604700
	H	2.61244400	2.06979100	0.10079000
	C	2.64744700	4.11123100	0.60431200
	H	3.08819200	4.31200000	1.58681800
	C	2.88434100	5.37103200	-0.28341000
	H	3.96427000	5.52959500	-0.25052800
	C	2.47824700	5.16236600	-1.74444200
	H	2.74580300	6.04567600	-2.33328600
	H	2.98867800	4.29802800	-2.17689100
	H	1.40159900	4.99874400	-1.83885200
	C	2.19215900	6.59748400	0.32351300
	H	2.44138300	6.72109700	1.38238600
	H	2.50200300	7.50410900	-0.20464600
	H	1.10213200	6.52590700	0.24124000
	C	1.14671300	3.88625200	0.78513600
	O	0.41943000	3.49809300	-0.10930100
	O	0.75793200	4.24117500	2.01358900
	C	-0.66564900	4.20292700	2.42240000
	C	-1.49936100	5.11303800	1.52015700
	C	-1.16232200	2.75899400	2.41128300
	C	-0.61013100	4.75073500	3.84655300
	H	-1.06833400	6.11795100	1.48906800
	H	-1.56163200	4.71844300	0.50700600
	H	-2.51103700	5.18947700	1.92959000
	H	-0.53375400	2.14002000	3.05371700
	H	-2.18722900	2.73098500	2.79472100
	H	-1.15392500	2.34293400	1.40659000
	H	-1.61400600	4.76123900	4.27973700
	H	0.03237400	4.12501600	4.47208600
	H	-0.21722800	5.77119100	3.85340800
	C	-0.63024800	0.25200400	-0.50573500
	C	-0.15478300	-0.56253300	0.58834700
	C	0.95186000	-0.12703900	1.36179100
	O	1.60996700	0.92750100	1.22736600
	O	1.23838300	-1.00942400	2.38246500
	C	2.10565900	-0.67202800	3.50317200
	C	2.01308700	-1.92742200	4.37310300
	H	0.97517500	-2.11724000	4.66230000
	H	2.38304700	-2.79638500	3.82079500
	H	2.61276500	-1.80886100	5.27989600
	C	1.55737900	0.54893300	4.24607300
	H	1.59798800	1.43263900	3.60896100
	H	0.52097100	0.37543200	4.55229300
	H	2.15203600	0.73423700	5.14595700
	C	3.55287900	-0.45105800	3.05685000
	H	3.65992000	0.49354500	2.52709300
	H	4.20807500	-0.44078800	3.93386900
	H	3.87003800	-1.26484000	2.40035100
	C	-0.73155900	-1.90490500	0.86968300
	H	0.03625200	-2.58408800	1.23654300
	C	-2.05485200	0.64157300	-0.67430600
	C	-3.13807500	-0.24227900	-0.54091200
	C	-2.34754500	1.95746300	-1.07150000
	C	-4.43850900	0.15720400	-0.81589300
	H	-2.96693100	-1.26593700	-0.24892100
	C	-3.65189100	2.36537500	-1.33443000
	H	-1.53015900	2.66195300	-1.17830300
	C	-4.72453700	1.46786300	-1.23037700
	H	-5.24757300	-0.55874100	-0.70802800

	H	-3.83570600	3.38284500	-1.66633000
	H	-1.13803500	-2.30678700	-0.05402400
	H	0.00973300	1.12373000	-0.62343000
	C	-6.10556100	1.87224900	-1.57389400
	C	-6.96537100	0.98042300	-2.23760600
	C	-6.58855500	3.15306900	-1.25905600
	C	-8.26615800	1.35499800	-2.56913700
	H	-6.59538900	-0.00023500	-2.52022600
	C	-7.88856600	3.52886600	-1.59332900
	H	-5.94497900	3.84923400	-0.73020300
	C	-8.73425300	2.63098000	-2.24802100
	H	-8.91182100	0.65363400	-3.08922400
	H	-8.24448900	4.52173300	-1.33504900
	H	-9.74687000	2.92358800	-2.50767200
	C	-4.85068100	-4.92914000	0.35047000
	C	-5.57695500	-4.20922300	1.49217700
	C	-4.74006100	-4.07173200	2.77018600
	C	-3.52569900	-4.29329100	-0.08231900
	C	-2.49014400	-4.17140800	1.05981300
	C	-2.71224300	-2.95930500	1.93124100
	H	-4.65328700	-5.96323700	0.66252300
	H	-5.89824600	-3.20840500	1.17763700
	H	-4.19966700	-4.99723200	2.98797300
	H	-3.70116500	-3.30940900	-0.53067000
	H	-1.48243500	-4.15277100	0.65367300
	H	-5.51687800	-4.98965500	-0.51672900
	H	-6.48529500	-4.76851400	1.74044700
	H	-5.38564600	-3.88146600	3.62815800
	H	-3.07912000	-4.90943500	-0.86856200
	H	-2.53881700	-5.06062700	1.69639200
	C	-4.20665700	-1.75080100	3.49023400
	H	-4.00501600	-1.93933100	4.55061800
	H	-5.28805200	-1.64529900	3.36998500
	C	-3.48899800	-0.50418800	3.00406600
	H	-3.61461600	0.29946100	3.73338200
	H	-3.91034500	-0.16761000	2.05327000
	C	-2.01434900	-0.81409300	2.81154500
	H	-1.53131100	-1.06770000	3.76187700
	H	-1.48167600	0.02461100	2.36971200
	N	-3.79355800	-2.94146600	2.72264900
	N	-1.86269100	-1.94179700	1.88665900
**TS2-RS**	C	-7.15417500	0.50667000	-0.90818200
	C	-6.05885900	1.33180400	-1.03360300
	C	-5.16015000	1.52022800	0.04878000
	C	-5.40774300	0.82257200	1.27757000
	C	-6.55320800	-0.01195500	1.37709400
	C	-7.40809400	-0.16838100	0.31037600
	H	-7.82692800	0.36818400	-1.74874700
	H	-5.85953000	1.84349700	-1.96920800
	C	-4.01077000	2.36673200	-0.05002100
	C	-4.49115300	0.95082500	2.35096200
	H	-6.73426400	-0.53506100	2.31174800
	H	-8.27673300	-0.81349500	0.39712200
	C	-3.34165400	1.69218400	2.19868000
	C	-3.11735000	2.40800500	0.99831000
	H	-4.67539300	0.42162100	3.28050100
	H	-2.23025100	3.02556100	0.91231000
	C	-3.77074900	3.26065900	-1.21781200
	C	-2.64990000	3.06277800	-2.05962700
	C	-4.59698300	4.38411500	-1.41311000

	C	-2.40268800	4.04841900	-3.05522300
	C	-3.30003000	5.11134400	-3.17809200
	H	-3.12332900	5.85481100	-3.95313800
	C	-5.77890100	4.67104200	-0.51862900
	H	-6.12037700	5.69190200	-0.69720800
	H	-6.61409700	3.98969200	-0.71396000
	H	-5.51934900	4.55901800	0.53845500
	N	-4.36315700	5.28166600	-2.38774400
	O	-1.33989200	3.98305000	-3.87584100
	H	-0.86258100	3.12101400	-3.60929900
	C	-1.81622700	1.87203600	-1.98069900
	H	-2.07885200	1.10473200	-1.25423900
	N	-0.78024900	1.76673900	-2.77067100
	C	0.00977700	0.64945500	-2.97442900
	C	-2.31506000	1.74643200	3.28909800
	O	-2.60506200	1.88164400	4.48372600
	N	-1.04549200	1.65781300	2.82417300
	H	-0.90036200	1.30443400	1.87986500
	C	0.11677800	1.79154400	3.67734300
	H	-0.04835200	2.62847100	4.36345400
	C	0.36285400	0.51147000	4.52977700
	H	-0.63074000	0.27054200	4.92189000
	C	0.84027300	-0.65484300	3.66261700
	H	0.82622000	-1.58424400	4.23841000
	H	0.20038800	-0.78809500	2.78759200
	H	1.86748900	-0.49394900	3.31559900
	C	1.29016100	0.75232800	5.72366000
	H	0.93761900	1.58907200	6.33570600
	H	1.32052400	-0.13924000	6.35841300
	H	2.30916200	0.97705300	5.40085000
	C	1.29331500	2.12626500	2.76112000
	O	1.23001300	2.03940400	1.54933300
	O	2.37297400	2.48291000	3.45956900
	C	3.67665000	2.72797400	2.78883800
	C	4.14036100	1.44929900	2.09312800
	C	3.55122100	3.90392500	1.82117100
	C	4.58758300	3.07892400	3.96195400
	H	4.19291900	0.62430000	2.81051000
	H	3.47598800	1.17733700	1.27505100
	H	5.14075800	1.60864400	1.68083700
	H	3.13832100	4.77668700	2.33550200
	H	4.54594700	4.16459500	1.44748600
	H	2.91783000	3.64918600	0.97329600
	H	5.59653700	3.28649600	3.59555100
	H	4.21880700	3.96473400	4.48617900
	H	4.64020800	2.24938500	4.67256400
	C	0.87915000	0.12342000	-0.96967900
	C	0.41326200	-1.14633800	-0.57504300
	C	-0.80817600	-1.18619500	0.17778200
	O	-1.44661700	-0.20029300	0.58337200
	O	-1.20365300	-2.47090800	0.46358800
	C	-2.46732800	-2.74018100	1.15265200
	C	-2.52524600	-4.26809300	1.18064200
	H	-2.57794500	-4.67019700	0.16537700
	H	-1.64012500	-4.68278600	1.67261300
	H	-3.41211500	-4.60034200	1.72743500
	C	-3.64367200	-2.18056200	0.34978500
	H	-4.58665700	-2.47768100	0.81838600
	H	-3.60440600	-1.09471600	0.30775100
	H	-3.62532000	-2.58151900	-0.66681800

	C	-2.43000900	-2.18282200	2.57813400
	H	-2.35328000	-1.09792200	2.56358700
	H	-3.34501500	-2.46822600	3.10658600
	H	-1.57815900	-2.59412600	3.12784400
	C	0.91913600	-2.42273900	-1.16476900
	H	1.63275200	-2.21798200	-1.96125600
	H	0.08019800	-2.96111700	-1.59735100
	H	0.30739200	0.93377400	-0.53422200
	C	-0.22914900	-7.37285600	-1.20653500
	C	0.33553000	-7.55935800	0.20637800
	C	1.75796800	-7.01277300	0.38336900
	C	-0.26482500	-5.92691600	-1.71155800
	C	1.11587500	-5.23691600	-1.72295700
	C	1.50791900	-4.68685100	-0.37529200
	H	0.37676900	-7.96775000	-1.90295000
	H	-0.31651600	-7.08331300	0.94902000
	H	2.38828300	-7.27157000	-0.47198100
	H	-0.95863400	-5.32365100	-1.11788600
	H	1.12753900	-4.43692700	-2.45802800
	H	-1.24143300	-7.78927900	-1.24453600
	H	0.35255700	-8.62894300	0.44154000
	H	2.22900300	-7.45598900	1.26155800
	H	-0.64807400	-5.91748600	-2.73549100
	H	1.88697700	-5.94839800	-2.03619500
	C	1.98385800	-5.14625200	2.00791600
	H	3.03749900	-5.29886000	2.26678500
	H	1.38760700	-5.82152800	2.62754000
	C	1.56590400	-3.70446400	2.23023900
	H	1.96146600	-3.35422900	3.18494200
	H	0.47755100	-3.61575900	2.25447900
	C	2.09160900	-2.85418800	1.08528600
	H	3.18696900	-2.87640100	1.05160400
	H	1.76678200	-1.81792300	1.16166600
	N	1.79263400	-5.55455400	0.60306000
	N	1.56416200	-3.37482900	-0.18122600
	C	2.27704400	0.50880900	-1.23166800
	C	2.55064100	1.87060600	-1.47751600
	C	3.38267600	-0.35608400	-1.15179100
	C	3.84545200	2.34534100	-1.60248100
	H	1.71586100	2.56125400	-1.54384900
	C	4.68579200	0.11927600	-1.28284200
	H	3.24450400	-1.40994600	-0.95451600
	C	4.94957700	1.47912200	-1.49376600
	H	4.01145800	3.40872400	-1.74203500
	H	5.51187000	-0.58220000	-1.21802600
	C	6.33424700	1.99501900	-1.56048300
	C	6.66973100	3.06686600	-2.40517700
	C	7.34831500	1.43592400	-0.76346300
	C	7.97185800	3.56108500	-2.45173400
	H	5.90776500	3.50003400	-3.04523800
	C	8.65112200	1.92847300	-0.81162200
	H	7.10525600	0.62481600	-0.08443400
	C	8.96940200	2.99417000	-1.65576600
	H	8.20966400	4.38567800	-3.11694700
	H	9.41664100	1.48594200	-0.18130000
	H	9.98349400	3.37963600	-1.69216400
	C	-0.51841100	-0.65607000	-3.34442200
	C	0.26174100	-1.46311000	-4.20426000
	C	-1.71825400	-1.20870200	-2.84977700
	C	-0.12830800	-2.75054900	-4.55102700

	H	1.19009600	-1.05768000	-4.59791300
	C	-2.11536500	-2.49611800	-3.21287300
	H	-2.34940400	-0.63486000	-2.18460800
	C	-1.32582000	-3.28118500	-4.05389300
	H	0.49412300	-3.34089500	-5.21767200
	H	-3.05037500	-2.89187000	-2.82764900
	H	-1.64309800	-4.28128400	-4.33151900
	H	0.90436000	0.90846600	-3.53738400
**TS2-RR**	C	7.92361600	1.46019900	0.96849100
	C	6.66686100	1.82491800	0.54122400
	C	5.83034500	0.90039200	-0.13875500
	C	6.31383500	-0.43419400	-0.35304500
	C	7.61861600	-0.77526100	0.09608100
	C	8.40828800	0.14939500	0.73885300
	H	8.54802400	2.17947000	1.48910400
	H	6.29283100	2.82589100	0.72681300
	C	4.52405200	1.25006500	-0.60501100
	C	5.48456800	-1.38322000	-1.00091400
	H	7.97821200	-1.78588000	-0.07466900
	H	9.40252600	-0.12362600	1.07806100
	C	4.20302000	-1.04642400	-1.37750300
	C	3.73294800	0.27579000	-1.17975900
	H	5.85868300	-2.38574500	-1.18448300
	H	2.73399000	0.53313900	-1.51600400
	C	3.98927400	2.63883700	-0.49991200
	C	2.89416000	2.90806400	0.35507500
	C	4.50337200	3.66993300	-1.30635300
	C	2.35552800	4.22127900	0.34391300
	C	2.95275300	5.17399900	-0.48311000
	H	2.55073600	6.18536600	-0.49184100
	C	5.64136800	3.43415300	-2.26944400
	H	5.70167700	4.27302900	-2.96481400
	H	6.60247200	3.34868300	-1.75046500
	H	5.50273700	2.50772200	-2.83482400
	N	3.99187900	4.91486500	-1.28329700
	O	1.29095200	4.55997700	1.09594800
	H	1.02738100	3.71733100	1.58752200
	C	2.32304000	1.88823900	1.20738200
	H	2.75031900	0.88554300	1.18130000
	N	1.30385800	2.14478000	1.98475900
	C	0.71561100	1.09261800	2.65642000
	H	1.34106400	0.20823200	2.77493800
	C	-0.21079100	1.35842700	3.75110800
	C	-0.64943200	0.27643400	4.54167800
	C	-0.77560900	2.62489100	3.99051700
	C	-1.64030200	0.44761700	5.50506600
	H	-0.21221700	-0.70321300	4.37686100
	C	-1.75195100	2.79672900	4.96847800
	H	-0.44907000	3.47133200	3.39730400
	C	-2.20116800	1.70977700	5.72381600
	H	-1.96898100	-0.40306700	6.09581200
	H	-2.17380100	3.78385000	5.13531100
	H	-2.96781400	1.84621600	6.48038300
	C	3.31728800	-2.01615000	-2.10118100
	O	3.72416400	-2.70002300	-3.04467400
	N	2.02887500	-2.01681800	-1.66483000
	H	1.81120000	-1.56354600	-0.77729000
	C	0.92543500	-2.44696600	-2.49971400
	H	1.35210300	-2.88831100	-3.40245500
	C	0.01683300	-3.48425900	-1.79859200

	H	-0.35396500	-3.01386000	-0.87832500
	C	-1.17579400	-3.87100600	-2.67891400
	H	-1.80419900	-4.60286500	-2.16423200
	H	-1.79754100	-3.01193600	-2.94107000
	H	-0.83408400	-4.32096700	-3.61716500
	C	0.83115900	-4.72417400	-1.42709900
	H	1.72110800	-4.45321100	-0.85928800
	H	0.23066200	-5.41018300	-0.82113900
	H	1.15065700	-5.25872500	-2.32877000
	C	0.13671000	-1.18892300	-2.87668900
	O	-0.06353500	-0.28271100	-2.08853700
	O	-0.28626900	-1.22782100	-4.14248000
	C	-1.02597800	-0.08993100	-4.74615400
	C	-2.33622500	0.14984200	-3.99583600
	C	-0.12729900	1.14604200	-4.75500900
	C	-1.29188300	-0.58815300	-6.16396400
	H	-2.92368000	-0.77247800	-3.95415500
	H	-2.15797700	0.51457000	-2.98468800
	H	-2.92595100	0.89536800	-4.53709300
	H	0.82551900	0.91798100	-5.24089800
	H	-0.61825300	1.94315300	-5.32060700
	H	0.06527700	1.50305100	-3.74345200
	H	-1.83275800	0.17501100	-6.72965600
	H	-0.35160800	-0.80232800	-6.67869200
	H	-1.89485200	-1.50020200	-6.14484000
	C	-0.39505600	0.19466300	1.16008000
	C	-0.62399300	-1.17020800	1.53332700
	C	0.54982900	-1.97214000	1.60604500
	O	1.69218400	-1.61451600	1.24625000
	O	0.33041900	-3.22247900	2.14882800
	C	1.40256100	-3.91522100	2.86678500
	C	0.67360000	-5.07370200	3.54850400
	H	-0.08894400	-4.69913200	4.23765200
	H	0.18921700	-5.71374500	2.80564300
	H	1.38332600	-5.68243400	4.11575800
	C	2.01624900	-2.98444900	3.91703400
	H	2.72628300	-3.54355700	4.53360000
	H	2.54136900	-2.15442400	3.44426500
	H	1.23668900	-2.58553800	4.57365000
	C	2.46255400	-4.45042000	1.90190900
	H	2.92829500	-3.63296200	1.35335200
	H	3.23006100	-4.99152800	2.46481400
	H	2.01037400	-5.14654900	1.19178000
	C	-1.93389500	-1.72801000	1.99318000
	H	-2.69601900	-0.95473000	2.02794300
	H	-1.87133000	-2.16090900	2.99299400
	C	-5.55548000	-0.98266300	-1.57531200
	C	-5.88967600	-2.47848300	-1.59619100
	C	-4.66620800	-3.38349300	-1.78371200
	C	-4.55201800	-0.55570100	-0.49989400
	C	-3.19725700	-1.28816300	-0.59082000
	C	-3.19920500	-2.64645000	0.04832000
	H	-5.14987200	-0.70408900	-2.55709200
	H	-6.40680400	-2.77404700	-0.67502500
	H	-3.99637800	-2.98045900	-2.54679100
	H	-4.97688200	-0.69648900	0.50100800
	H	-2.40899100	-0.69233800	-0.15013300
	H	-6.48052500	-0.40942600	-1.45172000
	H	-6.58115100	-2.67528400	-2.42246100
	H	-4.97159300	-4.36996100	-2.13417200

	H	-4.35063700	0.51361300	-0.60010600
	H	-2.91297800	-1.40895400	-1.63911000
	C	-4.13053800	-4.93977700	0.08062100
	H	-3.54230200	-5.68085500	-0.47237800
	H	-5.18652000	-5.19005700	-0.04681800
	C	-3.75294800	-4.92547700	1.55019500
	H	-3.69231200	-5.95019200	1.92353500
	H	-4.51436900	-4.39752700	2.13295200
	C	-2.41443600	-4.22289500	1.70634500
	H	-1.61911400	-4.75771300	1.17898600
	H	-2.11574700	-4.14616600	2.74862400
	N	-3.91210300	-3.61835600	-0.53652400
	N	-2.50685400	-2.85913800	1.16303100
	H	0.45614200	0.30276600	0.49204900
	C	-1.44356200	1.19605400	0.87721500
	C	-2.54971900	1.45060100	1.71051500
	C	-1.31219800	1.98872700	-0.27928300
	C	-3.50256500	2.40546600	1.36956200
	H	-2.65181100	0.93153600	2.65431700
	C	-2.26720700	2.93820200	-0.62096900
	H	-0.46891500	1.81369700	-0.93549100
	C	-3.39337200	3.15799000	0.19035900
	H	-4.33035900	2.58997400	2.04688600
	H	-2.15394900	3.49731800	-1.54450400
	C	-4.43085100	4.14329400	-0.18836000
	C	-5.78784100	3.89611500	0.07990900
	C	-4.08859300	5.34429600	-0.83209900
	C	-6.76852000	4.81827900	-0.28095900
	H	-6.07488100	2.96259700	0.55375000
	C	-5.06925400	6.26549100	-1.19546100
	H	-3.04400500	5.56592600	-1.02611900
	C	-6.41392800	6.00739700	-0.92128800
	H	-7.81196800	4.60384100	-0.07012700
	H	-4.78163500	7.19087100	-1.68533200
	H	-7.17743700	6.72557500	-1.20353600
**TS2-SS**	C	-7.97366900	-0.65836300	2.81246200
	C	-6.88703900	-1.11237200	2.09971800
	C	-6.10633300	-0.22233400	1.31524600
	C	-6.45100300	1.17123600	1.30765100
	C	-7.58317900	1.60476200	2.05101800
	C	-8.33165100	0.71221400	2.78222800
	H	-8.55935500	-1.35232000	3.40735700
	H	-6.60403600	-2.15890800	2.13420200
	C	-4.97752300	-0.65407700	0.55547100
	C	-5.64735500	2.08756000	0.58813300
	H	-7.84101200	2.65985700	2.03490900
	H	-9.19294000	1.05549800	3.34661400
	C	-4.52823000	1.66024300	-0.10116400
	C	-4.21752300	0.28036100	-0.12614800
	H	-5.89919300	3.14264600	0.58798700
	H	-3.35468400	-0.07174800	-0.67589200
	C	-4.52918000	-2.07723000	0.50140600
	C	-3.27282400	-2.40642300	1.06558700
	C	-5.24330300	-3.05779100	-0.20820300
	C	-2.75410600	-3.70195200	0.80747100
	C	-3.55420000	-4.60575300	0.11007300
	H	-3.17722700	-5.61099500	-0.06979100
	C	-6.56016400	-2.74746100	-0.87726100
	H	-7.36864200	-2.60493700	-0.15207500
	H	-6.49661200	-1.82620800	-1.46528400

	H	-6.83126600	-3.57292900	-1.53742000
	N	-4.76725500	-4.30596500	-0.37576600
	O	-1.51991000	-4.05704100	1.21985900
	H	-1.13757700	-3.21962600	1.65055300
	C	-2.51551600	-1.43591600	1.81808700
	H	-3.00270900	-0.49586300	2.04562000
	N	-1.28032100	-1.67806400	2.17263800
	C	-0.38736800	-0.76212500	2.64000400
	C	-3.69585600	2.70233000	-0.79549900
	O	-4.18659000	3.80350600	-1.08326800
	N	-2.38896100	2.41797000	-1.05040000
	H	-1.99097700	1.48849600	-0.91082300
	C	-1.58012200	3.40691800	-1.75382700
	H	-2.14415400	3.77362100	-2.61612700
	C	-1.23919300	4.64382300	-0.85612000
	H	-2.17467900	4.85275800	-0.33116600
	C	-0.15170700	4.34318700	0.17845600
	H	-0.09187600	5.15466800	0.91004300
	H	-0.35980800	3.42068100	0.72286300
	H	0.83341200	4.25198400	-0.29256000
	C	-0.89208900	5.87253700	-1.70126600
	H	-1.69982900	6.11190900	-2.40056000
	H	-0.73583900	6.74203100	-1.05499200
	H	0.02072400	5.71477500	-2.28267700
	C	-0.29612900	2.75527600	-2.24150900
	O	0.25086500	1.82816100	-1.67600200
	O	0.17420600	3.39158400	-3.32181800
	C	1.49805500	3.05330300	-3.89271600
	C	2.59019800	3.26051600	-2.84159300
	C	1.46804800	1.62401300	-4.43363400
	C	1.63818900	4.06449200	-5.02764200
	H	2.53582500	4.27324800	-2.43159800
	H	2.50405800	2.54385100	-2.02567600
	H	3.56891700	3.14079400	-3.31601900
	H	0.65094900	1.50608800	-5.15070800
	H	2.40970400	1.41118100	-4.94864400
	H	1.33317700	0.90311700	-3.62924500
	H	2.58562300	3.90691900	-5.55006800
	H	0.82165500	3.95159700	-5.74567300
	H	1.62157400	5.08597500	-4.63813600
	C	0.57803700	-0.36123700	0.63507700
	C	0.38308100	-1.42392000	-0.25920200
	C	-0.71150500	-1.32408900	-1.19140000
	O	-1.52806400	-0.39612800	-1.26686100
	O	-0.73340800	-2.36394100	-2.07860900
	C	-1.55231900	-2.32895700	-3.29124000
	C	-1.16598600	-3.63623300	-3.98477400
	H	-0.09144000	-3.65959000	-4.18887200
	H	-1.41839600	-4.49143600	-3.35135400
	H	-1.70319100	-3.73476100	-4.93215300
	C	-1.15443500	-1.12597800	-4.15002700
	H	-1.69105200	-1.15994800	-5.10316300
	H	-1.39172100	-0.19169600	-3.64240300
	H	-0.08096500	-1.15036400	-4.36103800
	C	-3.04434100	-2.33286400	-2.95375000
	H	-3.35190100	-1.39123300	-2.50376500
	H	-3.62144100	-2.49253700	-3.87048900
	H	-3.27351400	-3.14354500	-2.25958600
	C	1.30811500	-2.59144000	-0.34982100
	H	0.82968300	-3.41082300	-0.88245700

	C	1.89785600	0.06938800	1.13325600
	C	2.83089300	-0.77171100	1.76159600
	C	2.27417700	1.40809100	0.92539700
	C	4.10435900	-0.32250700	2.08723900
	H	2.55573000	-1.79067000	2.00498000
	C	3.54949900	1.85997100	1.24998100
	H	1.56259800	2.08225400	0.46562000
	C	4.50333400	0.99718100	1.81357500
	H	4.81242700	-1.01043300	2.53865500
	H	3.80640600	2.89922500	1.07052900
	H	1.57263400	-2.93890400	0.64584700
	H	-0.10436700	0.46288600	0.46832100
	C	5.88274900	1.45173100	2.09819700
	C	6.59207700	0.96998800	3.21132300
	C	6.52407300	2.36726600	1.24649800
	C	7.89722400	1.38864600	3.46341800
	H	6.10758700	0.28199800	3.89677800
	C	7.82800300	2.78839800	1.50025600
	H	6.00260000	2.73140300	0.36692100
	C	8.52148900	2.30015400	2.60951000
	H	8.42412900	1.00953200	4.33378900
	H	8.30648000	3.49183200	0.82556100
	H	9.53763500	2.62690300	2.80672100
	C	5.96778100	-4.33772400	1.18764700
	C	6.67161700	-3.33841100	0.26463900
	C	6.13711400	-3.35874900	-1.17197000
	C	4.45190600	-4.15129000	1.30280900
	C	3.69693100	-4.23602800	-0.04271900
	C	3.72812900	-2.96095500	-0.84916000
	H	6.16846500	-5.35242200	0.81906900
	H	6.58618700	-2.31746600	0.65675600
	H	5.96584400	-4.38479800	-1.51044700
	H	4.22704100	-3.19555700	1.78368400
	H	2.66421000	-4.52687500	0.12514300
	H	6.40916800	-4.27867100	2.18815700
	H	7.73999700	-3.57697300	0.23244700
	H	6.86783500	-2.92501300	-1.85542700
	H	4.04891100	-4.93074600	1.95603000
	H	4.13014200	-5.03022100	-0.66045600
	C	5.11073500	-1.26475500	-2.01436000
	H	5.29862500	-1.45466000	-3.07708700
	H	6.01499400	-0.82067500	-1.58880000
	C	3.91931800	-0.34848400	-1.81687000
	H	3.96396400	0.47192200	-2.53632200
	H	3.93057700	0.08304700	-0.81493800
	C	2.63791900	-1.14344400	-2.00596200
	H	2.56247800	-1.53837000	-3.02594300
	H	1.76290100	-0.53060800	-1.80352700
	N	4.90612300	-2.56543400	-1.34895700
	N	2.61336900	-2.27133300	-1.06299600
	H	0.47019700	-1.22289100	3.12307000
	C	-0.64457800	0.61396100	3.06947600
	C	-1.59045700	1.48468300	2.47983200
	C	0.17984300	1.15596500	4.08020400
	C	-1.73744800	2.79747700	2.92330900
	H	-2.19179000	1.15905600	1.64307000
	C	0.04182700	2.47184100	4.50940600
	H	0.93799900	0.52033900	4.52981800
	C	-0.93164200	3.30209900	3.94508100
	H	-2.47959700	3.43286800	2.44819100

	H	0.69254000	2.85091000	5.29258300
	H	-1.04892500	4.32595400	4.28602500

10.4 Transition States Calculations with ωB97X-D Functional

In order to further confirm the optimal transition state for the asymmetric addition step, we also carried out calculations by using the ωB97X-D functional[25] with the 6-311G(d,p) basis set.[26] Gibbs free energy corrections were applied using the quasi-rigid rotor harmonic oscillator (QRRHO) approach proposed by Grimme. The thermal free energy corrections were performed using the Shermo software.[27] The optimized three-dimensional geometries of four transition states, along with their calculated relative Gibbs free-energy values (kcal/mol), are presented in Figure S5. The Gibbs free energies (Hartree) of related structures are summarized in Table S8. The transition state ***TS3-SR*** displays the lowest Gibbs free energy, which is consistent with the calculation by using B3LYP(D3BJ) functional.

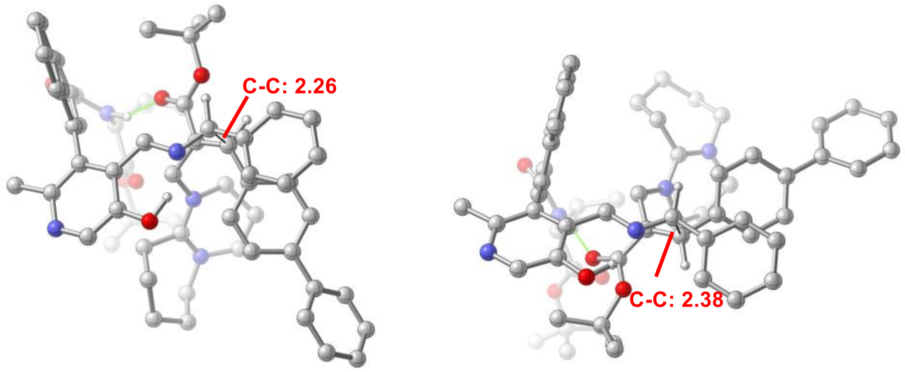

TS3-SR
$\Delta\Delta G^{\ddagger} = 0.0$

TS3-RR
$\Delta\Delta G^{\ddagger} = 6.4$

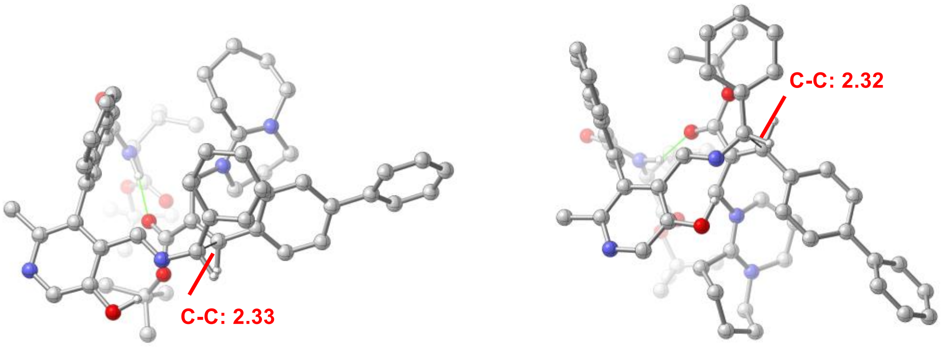

TS3-RS
$\Delta\Delta G^{\ddagger} = 12.6$

TS3-SS
$\Delta\Delta G^{\ddagger} = 6.0$

Figure S5. The optimized three-dimensional geometries were calculated at the ωB97X-D/6-311G(d,p) level with the PCM solvation model at 253 K. Relative Gibbs free energies are reported in kcal/mol.

Table S8. Gibbs free energies (Hartree) of transition states in Figure S5.

Geometry	G
**TS3-SR**	-3168.552897
**TS3-RS**	-3168.532768
**TS3-RR**	-3168.542747
**TS3-SS**	-3168.54329

Table S9. Cartesian coordinates of transition states in Figure S5.

Geometry	Cartesian coordinates			
**TS3-SR**	C	4.04709700	-5.32777500	2.01222200
	C	3.32134200	-4.18425100	2.21261100
	C	3.74549800	-2.94945900	1.65833100
	C	4.95406400	-2.91531600	0.90771500
	C	5.68526800	-4.11678000	0.72413400
	C	5.24159600	-5.29576700	1.25813400
	H	3.70592400	-6.26588400	2.43485100
	H	2.40618000	-4.21202900	2.79265800
	C	3.00483700	-1.74189800	1.83298100
	C	5.39735100	-1.69288400	0.34420200
	H	6.60320900	-4.08424100	0.14666700
	H	5.80560000	-6.20903000	1.10723600
	C	4.64088200	-0.56102500	0.47136200
	C	3.44767100	-0.59747200	1.22827900
	H	6.32568300	-1.66846200	-0.21660300
	H	2.89054500	0.32222000	1.36705600
	C	1.80814700	-1.65396400	2.72026500
	C	0.54457400	-2.12149000	2.30814900
	C	1.95494500	-1.03165000	3.96700100
	C	-0.53064200	-1.89424300	3.20281500
	C	-0.28038800	-1.25202600	4.40636600
	H	-1.11304400	-1.08268000	5.08481500
	C	3.29662600	-0.54596100	4.45589300
	H	4.08210000	-1.28578200	4.28322800
	H	3.60243900	0.37149600	3.94426500
	H	3.22882100	-0.33735200	5.52341700
	N	0.92219600	-0.83314800	4.79231800
	O	-1.79135100	-2.27818600	2.92773400
	H	-1.73908800	-2.70804900	2.02296700
	C	0.32091500	-2.75239000	1.02043400
	H	1.17004800	-2.94263700	0.36549800
	N	-0.89744000	-3.12844400	0.69485000
	C	-1.20501500	-3.51173800	-0.54832500
	C	5.06375700	0.74071500	-0.14671300
	O	6.21556900	1.15314500	-0.10038100
	N	4.03431700	1.40910400	-0.71029400
	H	3.17388600	0.89465400	-0.87986500
	C	4.10978500	2.77677100	-1.15179000
	H	5.04613800	3.19453900	-0.77713500
	C	4.10239700	2.89202400	-2.70011300
	H	4.83526100	2.14419000	-3.02095000
	C	2.74448300	2.51931800	-3.29777900
	H	2.81235500	2.47666900	-4.38776400
	H	2.39491200	1.54703200	-2.94180400
	H	1.98844100	3.27187800	-3.04580000
	C	4.57541600	4.25730900	-3.19739900
	H	5.54299600	4.52479400	-2.76420200
	H	4.68453000	4.23737600	-4.28495000
	H	3.86285100	5.04438300	-2.94159400

	C	2.94684500	3.53464300	-0.51541300
	O	1.96692100	2.99159200	-0.06028900
	O	3.14934100	4.84307200	-0.55919900
	C	2.13163700	5.80816700	-0.11790500
	C	0.86426200	5.63551500	-0.94784400
	C	1.87350200	5.64068400	1.37538300
	C	2.79038300	7.14931300	-0.40796700
	H	1.09493100	5.69440600	-2.01462200
	H	0.38795100	4.68000100	-0.73619100
	H	0.16334000	6.43774400	-0.70358400
	H	2.80911500	5.71619100	1.93408100
	H	1.20547700	6.43576300	1.71540100
	H	1.41390500	4.67634700	1.58792800
	H	2.12356200	7.96053200	-0.10782900
	H	3.72640900	7.24349300	0.14638100
	H	3.00327800	7.24943100	-1.47455900
	C	0.04473400	0.24695000	-0.03723200
	C	-0.07190000	-0.82190200	-1.07735800
	C	1.16065500	-1.14574300	-1.76455900
	O	2.20047100	-0.49714300	-1.68512000
	O	1.06457700	-2.25360000	-2.51790400
	C	2.21903400	-2.85765400	-3.17171400
	C	1.61784000	-4.09189100	-3.83432600
	H	1.17132100	-4.74851100	-3.08359300
	H	0.84458000	-3.80429500	-4.55090100
	H	2.39494700	-4.64750300	-4.36421100
	C	3.25128700	-3.26077500	-2.12318800
	H	4.05606300	-3.82636200	-2.60009800
	H	3.67751800	-2.38589000	-1.63633700
	H	2.79333900	-3.89807400	-1.36083500
	C	2.79845100	-1.91321200	-4.22202900
	H	3.25092100	-1.03965200	-3.75567800
	H	3.55840400	-2.44193800	-4.80333900
	H	2.01169800	-1.58466900	-4.90657900
	C	-1.24939400	-1.44978700	-1.47138100
	H	-1.21014200	-1.96207500	-2.42531700
	H	1.09952400	0.47337400	0.10721200
	C	-2.43364800	3.04769700	3.48038400
	C	-3.03635400	3.93949800	2.39500600
	C	-2.01187300	4.45717600	1.38447500
	C	-1.68501000	1.81883300	2.96626600
	C	-0.51932100	2.15651100	2.01927000
	C	-0.96177900	2.38655800	0.60059900
	H	-1.73978200	3.64938700	4.07991300
	H	-3.82382000	3.40566100	1.85096500
	H	-1.09729200	4.78690600	1.88322800
	H	-2.37542700	1.13353100	2.46491800
	H	0.23336700	1.37604600	2.04886000
	H	-3.22762000	2.72244900	4.15787300
	H	-3.50993900	4.80282300	2.87011600
	H	-2.40454000	5.32802800	0.85999600
	H	-1.27151200	1.27307100	3.81642800
	H	-0.01191100	3.06255200	2.36049000
	C	-2.36175600	3.71457200	-0.94225000
	H	-1.90180600	4.59165400	-1.40791300
	H	-3.40294800	3.96043900	-0.72086100
	C	-2.28098900	2.50691700	-1.85090700
	H	-2.49004200	2.80702700	-2.87809800
	H	-3.01783800	1.75577900	-1.55918900
	C	-0.89192500	1.90741900	-1.74110700

	H	-0.12109900	2.62445500	-2.04404900
	H	-0.79060900	1.01690100	-2.35476100
	N	-1.68385000	3.47771800	0.34014200
	N	-0.64978800	1.52939500	-0.35301400
	H	-0.35711100	-0.10150000	0.90803400
	H	-0.39409100	-3.77581900	-1.22799300
	C	-2.52938000	-4.02216400	-0.88014800
	C	-2.75442400	-4.53614500	-2.16559300
	C	-3.62467200	-3.93768700	-0.00765600
	C	-4.01967800	-4.94304500	-2.56641900
	H	-1.91975100	-4.60838100	-2.85742000
	C	-4.88807300	-4.34444700	-0.41042600
	H	-3.48699900	-3.53653500	0.98893100
	C	-5.09762600	-4.84826900	-1.69169400
	H	-4.16534100	-5.33649300	-3.56699500
	H	-5.71995700	-4.26365500	0.28138900
	H	-6.08721500	-5.16324900	-2.00270700
	C	-2.59848100	-0.98979300	-1.10672700
	C	-2.96309600	-0.59228200	0.18291300
	C	-3.58400600	-0.94998400	-2.09763800
	C	-4.23677600	-0.12254100	0.45534300
	H	-2.25905600	-0.68272600	0.99778000
	C	-4.85847700	-0.47490500	-1.82920900
	H	-3.34224900	-1.29420800	-3.09744900
	C	-5.20432300	-0.03619700	-0.54886600
	H	-4.48013700	0.19310500	1.46474400
	H	-5.60022300	-0.46148200	-2.62037000
	C	-6.55697100	0.49953200	-0.26068100
	C	-7.22003300	0.17279500	0.92593000
	C	-7.19541100	1.34914300	-1.16896200
	C	-8.48375200	0.68195700	1.19669800
	H	-6.75049600	-0.50236700	1.63307300
	C	-8.45961700	1.85782700	-0.89903000
	H	-6.68917100	1.62734400	-2.08717100
	C	-9.10833300	1.52669100	0.28549000
	H	-8.98543400	0.41115100	2.11887400
	H	-8.93630200	2.51989000	-1.61315800
	H	-10.09480400	1.92327200	0.49663400
**TS3-RS**	C	-0.50564800	-5.00670400	3.32246700
	C	-1.34722100	-4.51593000	2.36147900
	C	-1.74380200	-3.15297000	2.36950600
	C	-1.27503600	-2.31408900	3.41810600
	C	-0.39898400	-2.85221700	4.39706700
	C	-0.01746700	-4.16474300	4.34773500
	H	-0.20423000	-6.04737500	3.29638800
	H	-1.70966100	-5.16217300	1.57064900
	C	-2.56962600	-2.59245800	1.34834900
	C	-1.68635400	-0.96135200	3.46344100
	H	-0.04064800	-2.20149200	5.18803900
	H	0.65435600	-4.56658700	5.09743500
	C	-2.48841400	-0.44560100	2.48033100
	C	-2.92447200	-1.27099600	1.42056900
	H	-1.35787700	-0.32779000	4.28043900
	H	-3.53092800	-0.84790800	0.63046800
	C	-3.00170500	-3.38149400	0.15833200
	C	-2.06531400	-3.68142800	-0.84669100
	C	-4.33785700	-3.76590700	0.00475400
	C	-2.54555100	-4.34358200	-1.99592100
	C	-3.88992200	-4.68692300	-2.04893200
	H	-4.26181500	-5.20588800	-2.92886700

	C	-5.36869400	-3.47135400	1.06432400
	H	-4.99502100	-3.70173800	2.06525600
	H	-5.65257900	-2.41455500	1.06170900
	H	-6.26114100	-4.06588500	0.86956200
	N	-4.76608300	-4.41379500	-1.08323900
	O	-1.74703600	-4.64525200	-3.03423900
	H	-0.84067900	-4.30821800	-2.77696700
	C	-0.66000500	-3.34172700	-0.70030100
	H	-0.35977000	-2.90253300	0.23702900
	N	0.18645400	-3.63581300	-1.65177900
	C	1.50801500	-3.36480800	-1.61250400
	H	2.00992200	-3.59095900	-2.55120800
	C	2.40985700	-3.46518100	-0.46064600
	C	3.78843000	-3.52697500	-0.74245300
	C	2.04454900	-3.50619800	0.89815200
	C	4.74290000	-3.58655400	0.25849900
	H	4.10866100	-3.50826800	-1.77952600
	C	3.00468300	-3.56592900	1.90239100
	H	1.00956800	-3.50973400	1.20371800
	C	4.36033200	-3.59283700	1.59732300
	H	5.79401400	-3.62038500	-0.00786600
	H	2.67597000	-3.59496200	2.93647500
	H	5.10405300	-3.63237100	2.38503800
	C	-2.90462200	0.99226400	2.55698800
	O	-3.25719800	1.49130300	3.61997000
	N	-2.86242000	1.67925200	1.39325800
	H	-2.50366800	1.23946200	0.54399200
	C	-3.38566600	3.03257900	1.31948700
	H	-4.37859000	3.04679400	1.77426900
	C	-2.51130900	4.06622400	2.07997000
	H	-2.48871400	3.69365800	3.10610100
	C	-1.08379800	4.11774400	1.55107400
	H	-0.44985400	4.70701000	2.21950400
	H	-0.65959300	3.11353400	1.47482300
	H	-1.04904200	4.57329000	0.55779700
	C	-3.15868100	5.45012900	2.08743200
	H	-4.18816300	5.40851100	2.45263500
	H	-2.59489300	6.12264600	2.73866200
	H	-3.17473800	5.89260600	1.08670100
	C	-3.52444900	3.41425100	-0.14647600
	O	-2.68738800	3.16561700	-0.98052900
	O	-4.65369100	4.07744200	-0.35164900
	C	-5.00820900	4.63798600	-1.66509300
	C	-3.95825200	5.65468600	-2.10070300
	C	-5.18153400	3.50829400	-2.67459600
	C	-6.33862200	5.32350900	-1.38430700
	H	-3.81620700	6.41071300	-1.32414900
	H	-3.00357100	5.17607200	-2.31335200
	H	-4.30635500	6.15850900	-3.00561300
	H	-5.87964900	2.76098100	-2.28946600
	H	-5.59381900	3.91694100	-3.60041900
	H	-4.23227300	3.02442100	-2.89830400
	H	-6.71625800	5.78559800	-2.29893700
	H	-7.07502900	4.59922900	-1.02963900
	H	-6.21674200	6.09973000	-0.62556400
	C	1.24462900	-1.06798500	-1.89762500
	C	0.19668800	-0.65478500	-1.08172400
	C	-1.14321700	-0.46173100	-1.60287300
	O	-2.00771600	0.18460300	-1.01288700
	O	-1.35355500	-0.99532600	-2.81352700

	C	-2.53071100	-0.65863000	-3.60673500
	C	-2.35656600	-1.54246500	-4.83626200
	H	-1.41769900	-1.30849900	-5.34451300
	H	-2.33893900	-2.59349200	-4.53867200
	H	-3.18085900	-1.38191200	-5.53518800
	C	-2.47088800	0.82169300	-3.97480100
	H	-3.30257800	1.06883600	-4.64031400
	H	-2.54073200	1.44330700	-3.08081800
	H	-1.53578900	1.04571300	-4.49557400
	C	-3.83495400	-1.00763400	-2.89015100
	H	-4.65525800	-0.96010300	-3.61201800
	H	-3.79005700	-2.02202200	-2.49068300
	H	-4.03618300	-0.31700600	-2.07446100
	C	0.38786000	-0.15492300	0.31837400
	H	0.98932200	-0.87053300	0.87012500
	H	-0.57756400	-0.07564400	0.81123700
	H	0.95917800	-1.25170200	-2.92615700
	C	2.05934400	2.61863600	4.46366800
	C	2.75350800	3.59715900	3.51800400
	C	3.57731900	2.91386200	2.42709800
	C	1.10574700	1.63385900	3.79056300
	C	1.75736200	0.75101100	2.70608100
	C	1.87968400	1.47929100	1.39393300
	H	2.82873100	2.04744700	4.99710200
	H	2.02396700	4.26110200	3.04151600
	H	4.14419400	2.07175000	2.83411400
	H	0.24750400	2.15911600	3.35880800
	H	1.13971100	-0.13137000	2.57721200
	H	1.50769500	3.18029100	5.22221800
	H	3.42710500	4.23097000	4.10089200
	H	4.30870900	3.60747700	2.01753400
	H	0.70352300	0.96360200	4.55366100
	H	2.74245000	0.40689900	3.03198600
	C	2.93082400	3.14510300	0.00180300
	H	3.53440000	2.51787400	-0.66253100
	H	3.46442100	4.07611500	0.18038300
	C	1.55792400	3.40042200	-0.58444800
	H	1.64086400	3.88941400	-1.55557600
	H	1.00968600	4.06689400	0.08115500
	C	0.84884800	2.07376500	-0.75732700
	H	1.21308800	1.57750600	-1.65616200
	H	-0.23291100	2.20531000	-0.85177000
	N	2.77123500	2.45846000	1.28618600
	N	1.07188100	1.17400000	0.38806600
	C	2.64517800	-0.61340300	-1.76014500
	C	3.29862800	-0.16333600	-2.91450800
	C	3.35844500	-0.53683000	-0.55798300
	C	4.56860000	0.39131600	-2.86393600
	H	2.78935300	-0.23468400	-3.87025100
	C	4.62734200	0.01404600	-0.50403200
	H	2.93608600	-0.93143100	0.35549000
	C	5.25281200	0.50528200	-1.65103200
	H	5.03843200	0.72990500	-3.78105800
	H	5.13264000	0.07592100	0.45390900
	C	6.59507500	1.13141100	-1.57803300
	C	6.88821100	2.27898800	-2.32074000
	C	7.59265400	0.59546600	-0.75810800
	C	8.14173200	2.87323800	-2.24632200
	H	6.12159400	2.71951700	-2.94924300
	C	8.84579300	1.18984700	-0.68258000

	H	7.39102900	-0.30669100	-0.19058300
	C	9.12523200	2.33129300	-1.42628600
	H	8.34847500	3.76590600	-2.82589400
	H	9.60913600	0.75497900	-0.04721300
	H	10.10352700	2.79444100	-1.36818500
TS3-RR	C	-0.01419000	2.66852700	5.31966100
	C	0.79871400	2.84611200	4.23329100
	C	1.46544800	1.74413500	3.63609600
	C	1.29292200	0.45313100	4.20842700
	C	0.44322500	0.30225100	5.33617300
	C	-0.20008800	1.38166500	5.87552400
	H	-0.52006900	3.51950400	5.76099500
	H	0.93783800	3.83367300	3.80906300
	C	2.29496600	1.88856700	2.48405000
	C	1.96879800	-0.65339800	3.64418600
	H	0.31791100	-0.68632600	5.76613100
	H	-0.84795800	1.25717700	6.73551900
	C	2.74360800	-0.49822700	2.52524500
	C	2.90464800	0.78119800	1.95153100
	H	1.86418200	-1.63449500	4.09463300
	H	3.51417200	0.89489300	1.06421000
	C	2.53146500	3.20710500	1.82785600
	C	1.53824300	3.78856800	1.01604000
	C	3.78163000	3.82339300	1.96146500
	C	1.90760700	4.96362600	0.31714800
	C	3.17159600	5.49384300	0.51972800
	H	3.44155200	6.40605900	-0.00668800
	C	4.85738200	3.24058300	2.84340200
	H	4.45922500	2.91642800	3.80827300
	H	5.32623600	2.36765700	2.37913100
	H	5.62677700	3.99473300	3.00800500
	N	4.08983800	4.95505600	1.32253700
	O	1.07188900	5.58759000	-0.53623000
	H	0.23886100	5.03818200	-0.53959000
	C	0.20614100	3.23391600	0.89475700
	H	-0.11326300	2.47022000	1.60148000
	N	-0.65019800	3.76823800	0.04989300
	C	-1.89541600	3.31537200	-0.06346000
	H	-2.32988400	2.75026000	0.76099800
	C	-2.81003200	3.87395500	-1.04614400
	C	-4.19004900	3.64033600	-0.94092500
	C	-2.34942500	4.58256200	-2.16875600
	C	-5.07274000	4.10924300	-1.90397200
	H	-4.56626300	3.07566100	-0.09320700
	C	-3.23553800	5.05633100	-3.12508400
	H	-1.28371300	4.74344500	-2.29014100
	C	-4.60402300	4.82541700	-3.00147400
	H	-6.13473600	3.91437600	-1.79649600
	H	-2.85475100	5.60397600	-3.98106300
	H	-5.29327300	5.19353700	-3.75300800
	C	3.40368400	-1.71080300	1.94019900
	O	3.95554100	-2.53647100	2.65860100
	N	3.31590900	-1.83754100	0.59684200
	H	2.82283400	-1.13833200	0.04035400
	C	3.92711600	-2.96732900	-0.08223600
	H	4.97876400	-3.02782500	0.20628500
	C	3.26336800	-4.32507600	0.28496800
	H	3.41786400	-4.41715600	1.36118100
	C	1.76393000	-4.33663900	0.01057900
	H	1.31068900	-5.24151400	0.42488700

	H	1.27164700	-3.47200000	0.46441000
	H	1.56132400	-4.31489300	-1.06343500
	C	3.96679300	-5.49626900	-0.39949400
	H	5.04675700	-5.46811500	-0.23317200
	H	3.58719800	-6.44131000	-0.00338900
	H	3.79265800	-5.49592400	-1.47971000
	C	3.85356000	-2.72971400	-1.58287000
	O	2.87270800	-2.29990000	-2.14174000
	O	4.98203000	-3.10266900	-2.16894900
	C	5.15221500	-3.09801700	-3.63074600
	C	4.15426200	-4.05723400	-4.27027900
	C	5.01738400	-1.67718200	-4.16706600
	C	6.57709300	-3.60783000	-3.79925900
	H	4.25810000	-5.05734100	-3.84213200
	H	3.12963300	-3.71304700	-4.13512600
	H	4.36100100	-4.12306200	-5.34115000
	H	5.66219600	-0.99671600	-3.60549300
	H	5.33096300	-1.66058800	-5.21367800
	H	3.98985500	-1.32440800	-4.10441400
	H	6.82617300	-3.66384400	-4.86111300
	H	7.28470800	-2.93492300	-3.31017000
	H	6.68128400	-4.60370600	-3.36306300
	C	-0.16872500	-0.24251100	0.55430200
	C	-0.20625300	0.76916400	-0.55608400
	C	1.09274200	1.06401900	-1.15441200
	O	2.10871500	0.41905800	-0.91563800
	O	1.06180100	2.07209300	-2.02784400
	C	2.19883000	2.40286800	-2.88101300
	C	1.72390800	3.67755800	-3.56845600
	H	0.78917400	3.49626700	-4.10485900
	H	1.55545700	4.45805000	-2.82241800
	H	2.47575800	4.02320100	-4.28182900
	C	2.40370900	1.26881200	-3.88040600
	H	3.21449600	1.52366000	-4.56799800
	H	2.66364100	0.34613200	-3.35989100
	H	1.49381400	1.10629400	-4.46424400
	C	3.46674100	2.68324400	-2.07561100
	H	3.89496300	1.76887300	-1.67137200
	H	4.19849100	3.16086800	-2.73311400
	H	3.25152500	3.36621400	-1.25220300
	C	-1.36570000	1.24806200	-1.12276500
	H	-1.24515400	1.81131000	-2.04038800
	H	0.86402100	-0.44208000	0.82129900
	C	-1.58465900	-4.36572300	3.53727300
	C	-2.29658300	-4.98126800	2.33469800
	C	-3.17678100	-3.99016600	1.57485400
	C	-0.68853400	-3.17259800	3.21358100
	C	-1.41612400	-1.98877200	2.54266300
	C	-1.57151400	-2.20723100	1.06233600
	H	-2.34140900	-4.04466500	4.26319100
	H	-1.57361600	-5.41622100	1.63603300
	H	-3.74909400	-3.36686400	2.26808400
	H	0.15333000	-3.47905100	2.58320800
	H	-0.83379000	-1.09252300	2.72571400
	H	-0.98449000	-5.13327400	4.03287000
	H	-2.93399700	-5.79847900	2.68237100
	H	-3.90419500	-4.52417500	0.96632500
	H	-0.25732300	-2.80071800	4.14551600
	H	-2.39705500	-1.83159000	2.99806700
	C	-2.60870900	-3.34505700	-0.79205200

	H	-4.88083500	-0.58374800	1.35523300
	H	-3.10180800	-4.30420000	-0.93346900
	C	-1.24537900	-3.32077900	-1.45273600
	H	-1.33411900	-3.47371000	-2.52852200
	H	-0.64176400	-4.13267000	-1.04417900
	C	-0.61635100	-1.97007600	-1.19241200
	H	-1.05622500	-1.22755900	-1.85720800
	H	0.46413000	-1.98242700	-1.36473000
	N	-2.42813300	-3.13255700	0.64558400
	N	-0.82492500	-1.52958700	0.19864700
	H	-0.65941400	0.16542800	1.43260000
	C	-2.70743000	0.66080400	-0.94616000
	C	-3.49023000	0.44076000	-2.08274700
	C	-3.25315700	0.29548900	0.28953200
	C	-4.72947100	-0.17817500	-1.99836500
	H	-3.10898200	0.74137700	-3.05275000
	C	-4.48826000	-0.32154000	0.37840000
	H	-2.71679600	0.51550600	1.20494800
	C	-5.24351400	-0.58981100	-0.76778900
	H	-5.29216100	-0.36545300	-2.90638000
	H	-3.25733600	-2.55620900	-1.18853300
	C	-6.54203200	-1.29905800	-0.67423100
	C	-7.63087900	-0.92011700	-1.46427700
	C	-6.69913800	-2.37395800	0.20568900
	C	-8.84061400	-1.59719900	-1.37672100
	H	-7.53553600	-0.07581600	-2.13838900
	C	-7.90849500	-3.05106700	0.29450300
	H	-5.86004200	-2.69208800	0.81487500
	C	-8.98420300	-2.66513500	-0.49757200
	H	-9.67650800	-1.28432000	-1.99210700
	H	-8.00876900	-3.88593400	0.97893800
	H	-9.92873400	-3.19246100	-0.42961800
**TS3-SS**	C	5.70708100	-2.86493100	2.20541500
	C	4.59534000	-2.10655700	2.45781200
	C	4.41375900	-0.85141200	1.82215400
	C	5.42524000	-0.37738400	0.94032100
	C	6.56939500	-1.18334800	0.70877900
	C	6.70470600	-2.40072000	1.31858900
	H	5.82616800	-3.82786100	2.68877400
	H	3.83242400	-2.46424300	3.13975600
	C	3.24801200	-0.05494100	2.02784900
	C	5.26206600	0.87050700	0.28780200
	H	7.33333700	-0.81892600	0.03005100
	H	7.57903800	-3.01210500	1.12718400
	C	4.11019800	1.58850200	0.45231200
	C	3.11267100	1.11638100	1.33446400
	H	6.03897100	1.23186600	-0.37763700
	H	2.22186000	1.71574800	1.48648800
	C	2.16614300	-0.41118300	2.99156600
	C	1.21700300	-1.40476900	2.68756500
	C	2.04741500	0.33762900	4.17044600
	C	0.13795600	-1.54255300	3.59494800
	C	0.10519600	-0.73937800	4.72519200
	H	-0.72035600	-0.85915500	5.42276900
	C	3.06590100	1.38343100	4.54998400
	H	2.94921700	1.62254800	5.60697500
	H	4.08693800	1.03921000	4.36904200
	H	2.93010200	2.30347300	3.97344800
	N	1.03181500	0.16957000	5.02183300
	O	-0.86051800	-2.42417000	3.39569100

	C	1.31099600	-2.23919300	1.49910500
	H	2.16435000	-2.10543900	0.84447700
	N	0.37455900	-3.13758900	1.29196200
	C	0.22779600	-3.91390300	0.21024800
	C	3.87547100	2.87949200	-0.27796600
	O	4.74210700	3.73288000	-0.41943900
	N	2.60402200	3.00204200	-0.71482700
	H	2.03117800	2.16377200	-0.74439100
	C	2.04176600	4.21739600	-1.24134400
	H	2.66487700	5.05025000	-0.90968000
	C	1.99884100	4.22194100	-2.79378600
	H	3.00099900	3.88856600	-3.08338500
	C	0.98157700	3.21999100	-3.34187000
	H	1.09328800	3.12289800	-4.42472600
	H	1.10709800	2.22942000	-2.89766100
	H	-0.04237800	3.55895100	-3.14648100
	C	1.77938900	5.61767100	-3.37517600
	H	0.78220900	5.99589800	-3.13867800
	H	2.51128900	6.33008300	-2.98496200
	H	1.88245800	5.58725600	-4.46307000
	C	0.65007700	4.36769100	-0.63350000
	O	0.02824400	3.44223400	-0.16472300
	O	0.22180000	5.61769400	-0.72073400
	C	-1.13521800	6.00972400	-0.31108200
	C	-2.16298900	5.24096900	-1.13381300
	C	-1.30844400	5.78522600	1.18723000
	C	-1.17233200	7.49390700	-0.64639500
	H	-1.97044100	5.36834600	-2.20214400
	H	-2.14066400	4.18061400	-0.88903600
	H	-3.16056400	5.63061100	-0.91600300
	H	-0.52580100	6.30627600	1.74330000
	H	-2.27610700	6.18537800	1.49993000
	H	-1.26819200	4.72477900	1.43319600
	H	-2.14568900	7.90922500	-0.37656600
	H	-0.39829600	8.03050100	-0.09388700
	H	-1.01094700	7.64918800	-1.71541700
	C	-0.39893000	-0.03793100	0.17985300
	C	0.06921400	-1.05542300	-0.81092400
	C	1.26445700	-0.67958700	-1.54847200
	O	1.81620300	0.40975500	-1.43361300
	O	1.68932900	-1.61095600	-2.40663000
	C	2.91097700	-1.44593600	-3.18923200
	C	2.93522900	-2.71098600	-4.03668700
	H	2.91398900	-3.59349200	-3.39641100
	H	2.07071000	-2.74038800	-4.70515900
	H	3.84416200	-2.73529400	-4.64284200
	C	4.10836700	-1.40060100	-2.24686100
	H	5.03366200	-1.38251000	-2.82919600
	H	4.07355300	-0.51217400	-1.61888100
	H	4.11814400	-2.28751100	-1.60870500
	C	2.83289400	-0.20998700	-4.08446100
	H	2.91567700	0.70907600	-3.50780200
	H	3.64717500	-0.24895300	-4.81305000
	H	1.88664800	-0.20051400	-4.63282600
	C	-0.61029300	-2.21834300	-1.13217600
	H	-0.23513900	-2.75101800	-1.99740500
	H	0.43442200	0.61985300	0.42294000
	C	-4.07955900	1.56951000	3.33901000
	C	-4.92854600	2.06535500	2.16810100
	C	-4.15872700	2.93288400	1.17105900

	C	-2.83832000	0.77003200	2.94489600
	C	-1.86434200	1.54876600	2.04191700
	C	-2.25815800	1.49959500	0.59208600
	H	-3.75943200	2.43651000	3.92957300
	H	-5.36859500	1.21985200	1.62697800
	H	-3.51722500	3.65210200	1.68555500
	H	-3.12565500	-0.16225200	2.44859300
	H	-0.85320200	1.17396800	2.16328400
	H	-4.70222600	0.95811400	3.99756900
	H	-5.76036000	2.65660100	2.56035700
	H	-4.85074100	3.51893800	0.56637300
	H	-2.29641700	0.48355100	3.84823000
	H	-1.82724500	2.59816000	2.34589000
	C	-3.99488000	1.97840500	-1.09792200
	H	-3.94565900	2.93759100	-1.62216100
	H	-5.04841800	1.74513900	-0.92602400
	C	-3.33047500	0.88088000	-1.90056400
	H	-3.58334300	0.99320800	-2.95525200
	H	-3.67762100	-0.10015800	-1.57077900
	C	-1.82971100	0.96735800	-1.69746200
	H	-1.43349000	1.93259400	-2.02950000
	H	-1.31122700	0.17838700	-2.23469900
	N	-3.36239900	2.14877700	0.21811500
	N	-1.53811600	0.81665200	-0.27652800
	H	-0.72328800	-0.52824600	1.09016700
	C	-2.05819100	-2.40582000	-0.88994600
	C	-2.69349600	-2.17701700	0.33482200
	C	-2.86170200	-2.76539300	-1.97674100
	C	-4.07301500	-2.23524600	0.44704900
	H	-2.10795200	-1.96525500	1.21927700
	C	-4.24377300	-2.82358600	-1.86570000
	H	-2.39481500	-2.97961700	-2.93266800
	C	-4.87799400	-2.53552300	-0.65549200
	H	-4.53413200	-2.02392400	1.40636400
	H	-4.83630700	-3.10025800	-2.73102200
	C	-6.35700500	-2.53714500	-0.54287400
	C	-6.99248500	-3.06455400	0.58505500
	C	-7.14710600	-1.99674600	-1.56188800
	C	-8.37751600	-3.05193500	0.69051500
	H	-6.39830200	-3.50614400	1.37771400
	C	-8.53227800	-1.98494600	-1.45722500
	H	-6.67098000	-1.56588000	-2.43625000
	C	-9.15273800	-2.51218700	-0.33004000
	H	-8.85267500	-3.47247500	1.56956600
	H	-9.12793900	-1.55656500	-2.25544800
	H	-10.23350500	-2.50320800	-0.24762900
	H	-0.68208200	-4.50956600	0.22932700
	C	1.29113100	-4.51165400	-0.61109100
	C	0.89958400	-5.31872300	-1.69396400
	C	2.66375100	-4.42714100	-0.33516400
	C	1.82608700	-6.00985600	-2.45738300
	H	-0.15899100	-5.41172500	-1.92039500
	C	3.59171200	-5.12742600	-1.09777700
	H	3.02227400	-3.84577700	0.50416200
	C	3.18519000	-5.92137900	-2.16294700
	H	1.48840600	-6.62591000	-3.28402300
	H	4.64536900	-5.04850100	-0.85029200
	H	3.91344100	-6.46112600	-2.75739200
	H	-0.59970600	-2.91538900	2.55462000

10.5 DFT Calculations on Chemoselectivity of α-C-Alkylation over N-Alkylation.

We conducted a computational comparison of two pathways: (i) the addition of benzylamine to the pyridoxal catalyst (*S*,*S*)-**6a** to form imine **12**, and (ii) the S_N2 attack of free benzylamine on intermediate **14**. The corresponding potential energy surfaces are presented in Figure S6. The results reveal that the addition of benzylamine to (*S*,*S*)-**6a**, leading to intermediate **12**, is energetically more favorable. In addition, we performed calculations for the following two pathways: α-C-alkylation and N-alkylation. The potential energy surfaces for both pathways are shown in Figure S7. The results indicate that α-C-Alkylation of benzylamine with MBH-adduct-derived intermediate **14** is energetically more favorable, which aligns well with the experimentally observed chemoselectivity.

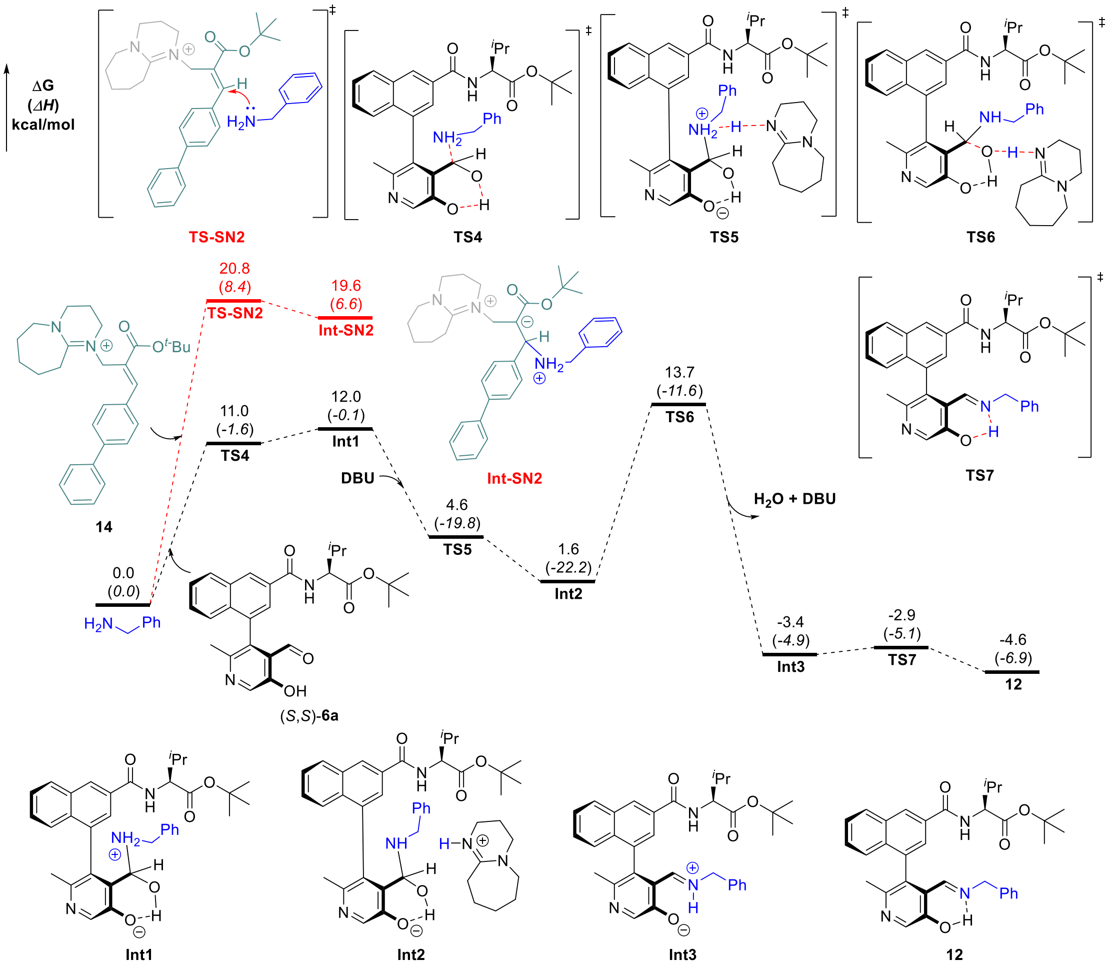

Figure S6. The potential energy surfaces.

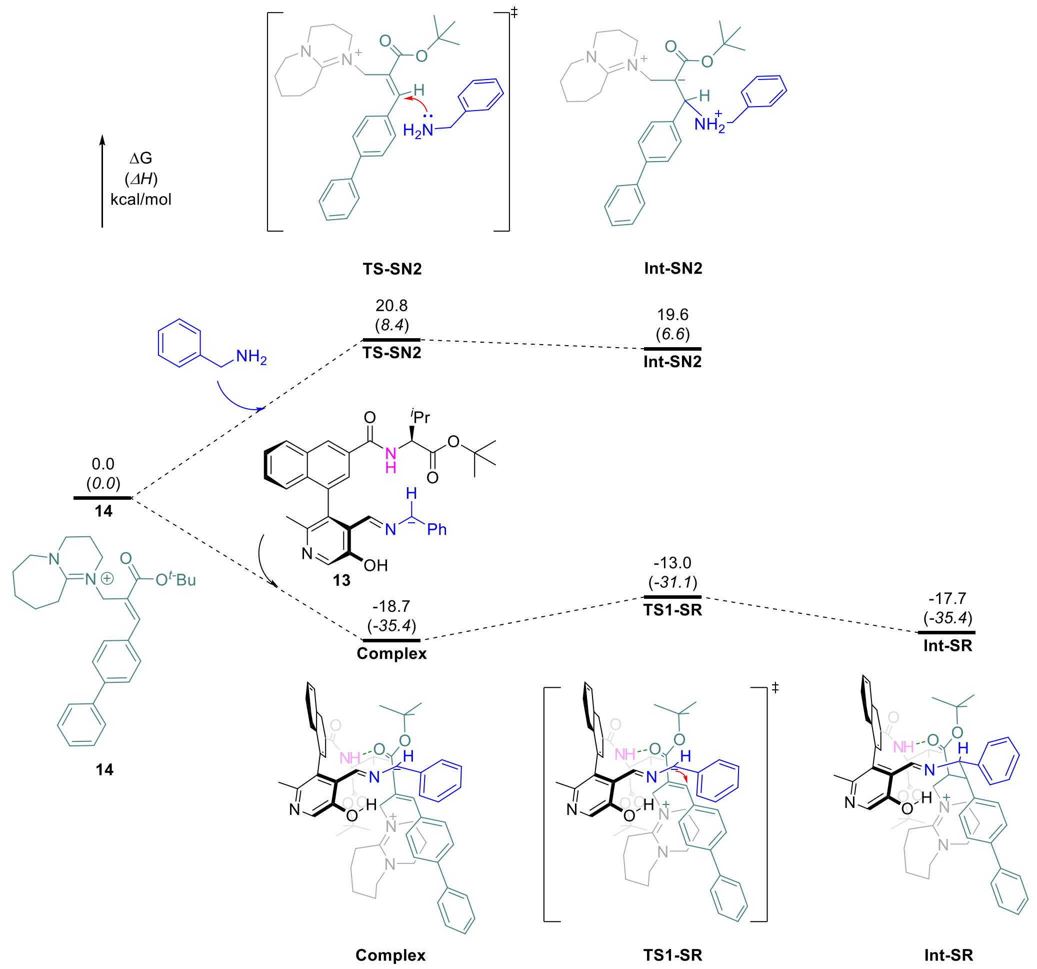

Figure S7. The potential energy surfaces.

Table S10. Enthalpies and Gibbs free energies (Hartree) of related structures in Figure S6 and Figure S7.

Geometry	H	G
**(*S*,*S*)-6a**	-1532.822124	-1532.900642
**DBU**	-462.063548	-462.099891
**H_2O**	-76.44531	-76.462975
**TS4**	-1859.741909	-1859.83265
**TS5**	-2321.834535	-2321.942692
**TS6**	-2321.821434	-2321.928238
**TS7**	-1783.302283	-1783.391709
**Int1**	-1859.739595	-1859.83097
**Int2**	-2321.838255	-2321.947461
**Int3**	-1783.301862	-1783.392578
**12**	-1783.305061	-1783.394432
**13**	-1782.822255	-1782.911946
**14**	-1387.234367	-1387.308461
**$PhCH_2NH_2$**	-326.917276	-326.949497
**Complex**	-3170.11298	-3170.250167
**TS1-SR**	-3170.106228	-3170.24112
**Int-SR**	-3170.113013	-3170.248676
**TS-SN2**	-1714.138207	-1714.22487
**Int-SN2**	-1714.1412	-1714.2267

Table S11. Cartesian coordinates of related structures in Figure S6 and Figure S7

Geometry	Cartesian coordinates			
(*S*,*S*)-**6a**	C	5.64805000	-2.22343300	0.14232500
	C	4.79964300	-1.14580600	0.01660900
	C	3.39963100	-1.33794000	-0.11493900
	C	2.88255900	-2.67413100	-0.09915800
	C	3.78539200	-3.76304100	0.03264000
	C	5.13872300	-3.54422700	0.14802300
	H	6.71646000	-2.06061900	0.24112200
	H	5.19530800	-0.13608100	0.02050900
	C	2.48358400	-0.24907600	-0.26131300
	C	1.48642900	-2.88024700	-0.20741000
	H	3.38456500	-4.77212300	0.04209000
	H	5.82046200	-4.38259100	0.24741500
	C	0.61472400	-1.81718200	-0.31266900
	C	1.12870600	-0.49472200	-0.34859600
	H	1.08582100	-3.88823100	-0.20109800
	H	0.46112700	0.34835700	-0.49340300
	C	2.98142800	1.15342100	-0.35383100
	C	2.86601900	2.03393600	0.74524400
	C	3.54564300	1.63041900	-1.54618300
	C	3.31304000	3.36687400	0.59907000
	C	3.86374100	3.74107500	-0.63624900
	H	4.21629300	4.76107500	-0.76922100
	C	3.69762300	0.75083100	-2.76000600
	H	4.47635100	-0.00452200	-2.60841900
	H	2.77083900	0.21454200	-2.98290000
	H	3.97359100	1.36425300	-3.61839200
	N	3.97331000	2.90501400	-1.66248500
	O	3.23721900	4.27392700	1.58307400
	H	2.82973000	3.81488600	2.35936600
	C	-0.85224400	-2.11996100	-0.39733100
	O	-1.26757500	-3.23363700	-0.73548100
	N	-1.68646600	-1.10426700	-0.06724600
	H	-1.33809100	-0.21791600	0.27005200
	C	-3.13011300	-1.22726700	-0.13172500
	H	-3.39910800	-1.76229900	-1.04660700
	C	-3.69765500	-2.02529000	1.08233800
	H	-3.09264400	-2.93808700	1.09423900
	C	-3.48655500	-1.28154400	2.40434700
	H	-3.76969700	-1.91939600	3.24666800
	H	-2.44025700	-0.99216800	2.53869100
	H	-4.09846200	-0.37406300	2.45547500
	C	-5.16037000	-2.43300200	0.88564000
	H	-5.30511700	-2.94920300	-0.06886500
	H	-5.46734800	-3.11231200	1.68682900
	H	-5.82624700	-1.56661500	0.90240600
	C	-3.68362200	0.19191100	-0.19566500
	O	-3.04145100	1.16276100	0.17019100
	O	-4.93069000	0.19937700	-0.65737900
	C	-5.74047600	1.44359500	-0.71516100
	C	-5.90547500	2.00942400	0.69433400
	C	-5.08497500	2.43351100	-1.67656200
	C	-7.07271100	0.94268300	-1.26588000
	H	-6.31728900	1.24854900	1.36369400
	H	-4.95463900	2.36010400	1.09514200
	H	-6.60435800	2.85022300	0.66326500
	H	-4.92299300	1.96468700	-2.65130700
	H	-5.74967300	3.29090600	-1.81604800
	H	-4.13063700	2.78873600	-1.28866900

	H	-7.76823900	1.77998300	-1.36633100
	H	-6.93516400	0.48485900	-2.24902400
	H	-7.51468900	0.20201100	-0.59409700
	C	2.33882100	1.59424000	2.03607200
	O	2.21264000	2.34920400	3.00533700
	H	2.05767700	0.53445900	2.12581500
**DBU**	C	2.76671900	0.00282900	0.39182800
	C	1.97029900	1.30968700	0.31914200
	C	0.90550900	1.31524100	-0.78671200
	C	1.92583100	-1.26326700	0.59208600
	C	0.89821900	-1.53815400	-0.52561000
	C	-0.39667000	-0.76982800	-0.36655300
	H	3.34055300	-0.10566700	-0.53937000
	H	1.47698200	1.51285800	1.27833800
	H	1.32671300	0.90830800	-1.71334400
	H	1.39917500	-1.21926100	1.55401500
	H	0.61247100	-2.59098100	-0.51845700
	H	3.50416700	0.07508800	1.19999200
	H	2.66607500	2.13818900	0.13909100
	H	0.61274000	2.34391900	-1.01310900
	H	2.60540800	-2.12094500	0.65089100
	H	1.35433900	-1.33455200	-1.50317400
	C	-1.45349900	1.43839500	-0.02259600
	H	-1.92991600	1.88687900	-0.90618200
	H	-1.06801300	2.26322600	0.58842500
	C	-2.46538100	0.62087400	0.76929300
	H	-3.39114700	1.19166000	0.89024500
	H	-2.06855900	0.41252500	1.76965600
	C	-2.70436600	-0.70486700	0.04525500
	H	-3.17216900	-0.50631300	-0.93242100
	H	-3.41275100	-1.32581500	0.60539100
	N	-0.32313600	0.60328400	-0.43991000
	N	-1.46772500	-1.45965300	-0.13820100
**H_2O**	O	0.00000000	0.00000000	0.12001000
	H	0.00000000	0.75713700	-0.48003800
	H	0.00000000	-0.75713700	-0.48003800
**TS4**	C	4.61260000	0.32056400	2.32384300
	C	3.62012100	-0.50708500	1.84391500
	C	2.27259300	-0.05796100	1.76511200
	C	1.96692300	1.27326400	2.20198700
	C	3.01291500	2.09414300	2.70342400
	C	4.30793600	1.63076500	2.76094100
	H	5.63582900	-0.03682700	2.37216500
	H	3.85639000	-1.51855400	1.53002000
	C	1.21812400	-0.87853100	1.24683400
	C	0.63499300	1.74355800	2.11259300
	H	2.77041700	3.09910000	3.03519000
	H	5.09930800	2.26849500	3.14030600
	C	-0.36714600	0.94754700	1.59990200
	C	-0.06299400	-0.37235400	1.17480600
	H	0.39080400	2.74706500	2.44361300
	H	-0.84635200	-1.02594800	0.80540400
	C	1.50867600	-2.25600200	0.74642800
	C	1.67810800	-2.48122700	-0.62690400
	C	1.63412200	-3.33438300	1.63944600
	C	1.97988800	-3.78598600	-1.09531500
	C	2.07540400	-4.79234800	-0.10541300
	H	2.30239500	-5.80932100	-0.42202200
	C	1.46187600	-3.15198100	3.12697100
	H	2.24649100	-2.51488000	3.55155100

	H	0.50426000	-2.67900300	3.36877100
	H	1.50703600	-4.12590900	3.61704400
	N	1.91348600	-4.57605100	1.19854000
	O	2.16043500	-4.03096000	-2.37652200
	H	1.86042600	-2.93762800	-2.84968500
	C	-1.74589900	1.53590800	1.51385800
	O	-2.06669300	2.53204600	2.17116200
	N	-2.60223500	0.92313200	0.66216200
	H	-2.31891400	0.13975700	0.09047800
	C	-3.97432200	1.36044600	0.48815900
	H	-4.39544200	1.60379200	1.46738600
	C	-4.06461600	2.63261500	-0.40946500
	H	-3.35343800	3.32458200	0.05425300
	C	-3.60986700	2.35271400	-1.84491600
	H	-3.55721200	3.28556400	-2.41379400
	H	-2.61987600	1.88799500	-1.86834000
	H	-4.30831600	1.68696900	-2.36393200
	C	-5.44972300	3.28390900	-0.36759300
	H	-5.77414800	3.46432600	0.66227000
	H	-5.42307300	4.24675700	-0.88701000
	H	-6.20400600	2.65861500	-0.85164500
	C	-4.73290100	0.18013200	-0.10854300
	O	-4.17044800	-0.73279500	-0.69043900
	O	-6.04403500	0.31519700	0.06732500
	C	-7.01792300	-0.64857900	-0.50758200
	C	-6.89047000	-0.65549900	-2.02994000
	C	-6.79387000	-2.02751800	0.11059000
	C	-8.35695100	-0.06112100	-0.07056900
	H	-6.99746700	0.35874700	-2.42543600
	H	-5.93024300	-1.06252500	-2.34611200
	H	-7.68869500	-1.27209000	-2.45307200
	H	-6.82365500	-1.96292700	1.20196500
	H	-7.59354800	-2.69956200	-0.21382700
	H	-5.83634700	-2.44784800	-0.19580500
	H	-9.17237500	-0.69338000	-0.43149100
	H	-8.41581500	-0.00616500	1.01966400
	H	-8.48945200	0.94429800	-0.47897500
	C	1.57460000	-1.38420900	-1.66246000
	O	1.51625800	-1.80327500	-2.92949600
	H	0.81445700	-0.63392200	-1.40234700
	C	4.28835900	2.28188400	-0.77083800
	C	5.46765200	2.96993800	-0.49498800
	C	6.50006200	2.98958300	-1.43572400
	C	6.34964200	2.31702900	-2.64979000
	C	5.17120500	1.62095200	-2.92240000
	C	4.13356400	1.60081900	-1.98488200
	H	3.48690000	2.27113100	-0.03823400
	H	5.57774900	3.48874600	0.45169200
	H	7.41798800	3.52868800	-1.22391900
	H	7.14887200	2.33282400	-3.38381700
	H	5.05510600	1.09331300	-3.86473900
	C	2.88072900	0.80540600	-2.23262700
	H	2.73583900	0.55081400	-3.28227800
	H	1.99281900	1.32908000	-1.87027500
	N	2.92726000	-0.49272800	-1.48647700
	H	3.69355100	-1.07186900	-1.83873100
	H	3.11756700	-0.31282600	-0.49525000
**TS5**	C	3.33563000	-3.80912400	0.76806900
	C	2.57583600	-2.99319300	-0.03992000
	C	1.18358000	-2.82793600	0.18709600

	C	0.59394200	-3.50191100	1.30875300
	C	1.41126200	-4.31973900	2.13601500
	C	2.75095100	-4.47969700	1.86855700
	H	4.39478500	-3.93466800	0.56784400
	H	3.03557800	-2.46645500	-0.86505500
	C	0.34949700	-2.01717700	-0.65012400
	C	-0.78337200	-3.32795300	1.58538500
	H	0.95305000	-4.82046700	2.98374100
	H	3.36360300	-5.11350800	2.50138200
	C	-1.56931300	-2.52800000	0.78330200
	C	-0.99399900	-1.90107000	-0.34906100
	H	-1.23092900	-3.81831900	2.44314600
	H	-1.61017700	-1.31249100	-1.02027700
	C	0.87826800	-1.25895200	-1.81886700
	C	0.74919300	0.13740500	-1.85336200
	C	1.43115300	-1.91627000	-2.94155400
	C	1.17154600	0.88126500	-2.99876100
	C	1.75872900	0.10278700	-4.03319400
	H	2.11552900	0.61940700	-4.92444500
	C	1.51031600	-3.42135000	-3.05212800
	H	2.45221900	-3.82388800	-2.66034900
	H	0.70107800	-3.91263100	-2.50557900
	H	1.44677400	-3.70442600	-4.10527200
	N	1.86579200	-1.22419300	-4.01168300
	O	0.99797400	2.16817700	-3.06828400
	H	-0.80759900	1.98448900	-2.12967200
	C	-3.00477300	-2.33159200	1.16923800
	O	-3.58353700	-3.09857100	1.94729800
	N	-3.61667300	-1.24426600	0.63684300
	H	-3.12386100	-0.58695900	0.04768900
	C	-4.99828700	-0.91503800	0.92822000
	H	-5.58776300	-1.83550500	0.92956700
	C	-5.14408300	-0.24232600	2.32793700
	H	-4.62516200	-0.93444400	2.99994000
	C	-4.43854800	1.11605600	2.38542300
	H	-4.44103900	1.50064000	3.40958800
	H	-3.39803700	1.04191700	2.05592400
	H	-4.94069000	1.85543500	1.75194400
	C	-6.60213000	-0.14624000	2.78525200
	H	-7.10300000	-1.11783400	2.72580400
	H	-6.64454300	0.19290300	3.82490700
	H	-7.16992800	0.56055400	2.17496200
	C	-5.48097900	-0.00672700	-0.19637300
	O	-4.71567300	0.63513900	-0.89562400
	O	-6.81047000	0.00266900	-0.26551100
	C	-7.53994300	0.88896000	-1.20744800
	C	-7.21490800	2.34771500	-0.88981400
	C	-7.18784200	0.50446000	-2.64352900
	C	-8.99947700	0.57041700	-0.89479700
	H	-7.42641500	2.56443200	0.16137700
	H	-6.16944300	2.57445800	-1.09740500
	H	-7.84381200	2.99779500	-1.50490500
	H	-7.37175700	-0.56135400	-2.80615300
	H	-7.82215600	1.06979100	-3.33235400
	H	-6.14408800	0.72394000	-2.86648900
	H	-9.65332200	1.16476600	-1.53849800
	H	-9.20839000	-0.48834200	-1.06956500
	H	-9.23118500	0.80491100	0.14763100
	C	0.06967500	0.97619200	-0.79058600
	O	-1.11233300	1.56084000	-1.29920100

	C	1.95584700	4.17478800	2.23034200
	C	2.98377500	5.05355200	2.57643500
	C	3.59899700	5.83014900	1.59414800
	C	3.17705300	5.72986100	0.26607400
	C	2.14568900	4.85662500	-0.07576500
	C	1.52825800	4.06739400	0.90376500
	H	1.48215100	3.56674100	2.99557200
	H	3.30479000	5.12782600	3.61085200
	H	4.40102100	6.51140800	1.86056600
	H	3.64882200	6.33446800	-0.50222100
	H	1.81977100	4.78576000	-1.11040400
	C	0.44885900	3.08801000	0.51319400
	H	-0.37196900	3.57724600	-0.01702100
	H	0.02817900	2.60047500	1.39703500
	N	1.02868900	2.03791700	-0.36252200
	H	1.31360900	2.45872700	-1.26797800
	H	1.98387700	1.51333700	0.23540100
	H	-0.20783600	0.40844300	0.09661200
	C	5.94141400	-0.78016800	-1.93089200
	C	6.28528300	-1.47368200	-0.60967100
	C	6.27977800	-0.53434000	0.60160900
	C	4.58169600	-0.07827600	-1.96609100
	C	4.39988300	1.02804100	-0.91170100
	C	4.05850100	0.54306600	0.47841800
	H	6.72321000	-0.03865300	-2.14595500
	H	5.59063700	-2.29791200	-0.41417800
	H	6.79225600	0.40310600	0.35796600
	H	3.76617500	-0.80043100	-1.86877000
	H	3.59734600	1.69589700	-1.22383600
	H	5.98184800	-1.51494400	-2.74269500
	H	7.28473900	-1.91578100	-0.69134700
	H	6.83687200	-0.98609500	1.42426600
	H	4.45033900	0.37978100	-2.95011100
	H	5.30703800	1.64313600	-0.85542700
	C	4.62484000	-0.82304200	2.44744300
	H	5.18160100	-0.27469900	3.21795100
	H	4.98487700	-1.85614000	2.45098000
	C	3.12900500	-0.79282000	2.73305800
	H	2.95747100	-1.00836800	3.79094100
	H	2.62739900	-1.56402900	2.14812400
	C	2.56158000	0.56929300	2.35288200
	H	2.93567900	1.34834300	3.03037100
	H	1.47049000	0.56988800	2.43807000
	N	4.94050700	-0.24391600	1.13154800
	N	2.91287600	0.92100200	0.98074600
**TS6**	C	6.88756700	-1.28619300	-0.56713500
	C	5.68993800	-1.21252800	0.10884700
	C	4.89385500	-0.03896200	0.05154200
	C	5.34245900	1.06293100	-0.75397900
	C	6.58265900	0.95125200	-1.43833600
	C	7.34172200	-0.19292700	-1.34386900
	H	7.48825300	-2.18846700	-0.50827200
	H	5.33631200	-2.05077000	0.69963200
	C	3.65884200	0.08198600	0.76149300
	C	4.51503200	2.20718600	-0.90153600
	H	6.91823000	1.78780700	-2.04460500
	H	8.28805500	-0.26409000	-1.87072700
	C	3.31161300	2.28621300	-0.23536800
	C	2.94041300	1.24975000	0.65336400
	H	4.80942400	3.00168700	-1.58032600

	H	2.03497200	1.36401400	1.23204900
	C	3.03841100	-1.04535700	1.51826800
	C	1.94310600	-1.72726900	0.93396400
	C	3.47008800	-1.38597500	2.80332400
	C	1.27693900	-2.76744700	1.67293900
	C	1.81698600	-2.99373800	2.98196100
	H	1.33677700	-3.76420600	3.58617800
	C	4.62728600	-0.68186500	3.46916500
	H	5.58667100	-0.93029000	2.99939100
	H	4.52393500	0.40725100	3.41724800
	H	4.67490100	-0.98067600	4.51811200
	N	2.84657300	-2.35384900	3.51612100
	O	0.27512700	-3.44841700	1.23415500
	H	0.43475300	0.21726400	0.75297200
	C	2.32214400	3.36852000	-0.55241300
	O	2.64997500	4.51497600	-0.88009600
	N	1.03499700	2.93988300	-0.47723400
	H	0.83143300	1.93554700	-0.38394600
	C	-0.08809800	3.80302100	-0.76289200
	H	0.22460400	4.83510600	-0.57971300
	C	-0.52046300	3.68328700	-2.25701500
	H	0.43022900	3.73619400	-2.80093300
	C	-1.16881300	2.32504900	-2.54935300
	H	-1.32313700	2.19700100	-3.62502100
	H	-0.55246100	1.49757500	-2.18719900
	H	-2.14903900	2.25163600	-2.06508800
	C	-1.39400000	4.84488700	-2.73918500
	H	-0.93183500	5.81039700	-2.50874500
	H	-1.52573900	4.78476900	-3.82438100
	H	-2.38123100	4.82463500	-2.27344400
	C	-1.20935500	3.44935100	0.21722000
	O	-1.16841600	2.50058400	0.97831400
	O	-2.23056400	4.30510700	0.10272300
	C	-3.48806400	4.11950100	0.86323800
	C	-4.13930000	2.79834600	0.45240900
	C	-3.20670400	4.19294500	2.36391800
	C	-4.33055800	5.30856300	0.40852300
	H	-4.30911700	2.77760600	-0.62811700
	H	-3.51411500	1.95114100	0.72899200
	H	-5.10789700	2.70122300	0.95133600
	H	-2.66482700	5.11271200	2.60188600
	H	-4.15636200	4.20663600	2.90683700
	H	-2.61939100	3.33892000	2.69901700
	H	-5.30277700	5.28641500	0.90813300
	H	-3.83299800	6.24964900	0.65714300
	H	-4.49458600	5.27539900	-0.67194400
	C	1.49638800	-1.40287500	-0.43208600
	O	0.35297300	0.11691900	-0.20467000
	C	-0.93391100	-4.34031700	-2.16420900
	C	-1.94286800	-5.21768600	-2.56967300
	C	-2.74420700	-4.91257400	-3.66924800
	C	-2.52812000	-3.72273300	-4.36843400
	C	-1.52449700	-2.84600700	-3.96070200
	C	-0.71977700	-3.14013900	-2.85208100
	H	-0.32126700	-4.59592300	-1.30635000
	H	-2.09993900	-6.14102100	-2.02067400
	H	-3.53014400	-5.59389600	-3.97956600
	H	-3.14519800	-3.47410400	-5.22640700
	H	-1.36705800	-1.91700300	-4.50280900
	C	0.35017700	-2.15289600	-2.43980900

	H	-0.02422500	-1.13073500	-2.58840600
	H	1.22224400	-2.25547300	-3.10250000
	N	0.76726700	-2.33441200	-1.06447000
	H	0.22575000	-2.90772600	-0.39523100
	H	-1.23441500	-0.17900400	-0.22152800
	H	2.17301200	-0.82408800	-1.05113100
	C	-2.92885100	-2.13956600	3.90964000
	C	-4.18480400	-2.55277100	3.13441600
	C	-4.77804400	-1.42750500	2.27925500
	C	-1.76965800	-1.63359100	3.04601700
	C	-2.10988100	-0.38667500	2.20502400
	C	-2.85381600	-0.70047300	0.93632700
	H	-3.20724100	-1.35463800	4.62667000
	H	-3.97371800	-3.41175100	2.48593500
	H	-4.78942200	-0.48362800	2.83326800
	H	-1.38846300	-2.42091600	2.38672800
	H	-1.20890300	0.15418900	1.92656200
	H	-2.58195200	-2.99174900	4.50417000
	H	-4.95323800	-2.87436200	3.84614200
	H	-5.81445600	-1.65045500	2.02131000
	H	-0.93564000	-1.36422200	3.70214800
	H	-2.70853800	0.32036300	2.79149800
	C	-4.77430600	-1.73344000	-0.20370900
	H	-5.57560800	-1.03090900	-0.46019700
	H	-5.23907500	-2.68579200	0.06456700
	C	-3.81170700	-1.91892200	-1.36781000
	H	-4.37423700	-2.08885800	-2.28768900
	H	-3.17411900	-2.79143700	-1.20164000
	C	-2.93671500	-0.67885400	-1.48898800
	H	-3.53974000	0.19930300	-1.74981200
	H	-2.17537800	-0.79951600	-2.25678000
	N	-4.07858700	-1.23044600	0.99746400
	N	-2.26446200	-0.44304600	-0.21563800
**TS7**	C	-4.10213800	-2.07486300	1.98510500
	C	-3.25354200	-1.00096600	1.82694800
	C	-1.87893500	-1.19839700	1.52515200
	C	-1.38797400	-2.54093300	1.41743900
	C	-2.29012200	-3.62545500	1.58940300
	C	-3.61976300	-3.39978400	1.86019600
	H	-5.14975400	-1.90370000	2.21144400
	H	-3.63257000	0.00843700	1.93282000
	C	-0.96482700	-0.11228900	1.32487100
	C	-0.01811700	-2.75995100	1.13789600
	H	-1.90728600	-4.63774700	1.50073600
	H	-4.30133000	-4.23477000	1.98571900
	C	0.84832800	-1.70414800	0.94733800
	C	0.35970600	-0.37564700	1.04528100
	H	0.36444800	-3.77233500	1.06627000
	H	1.03208100	0.46699400	0.92416700
	C	-1.44010000	1.29828800	1.40204600
	C	-2.28954200	1.80950500	0.39039600
	C	-1.09191900	2.12764300	2.46818500
	C	-2.79579600	3.14165000	0.49100100
	C	-2.34695900	3.88974700	1.61385000
	H	-2.69488500	4.91552700	1.72322500
	C	-0.21250900	1.66035000	3.60178100
	H	-0.44809500	0.63494100	3.89903300
	H	0.84900200	1.68047400	3.32856600
	H	-0.34885600	2.32163400	4.45933100
	N	-1.54205500	3.40684400	2.54524500

	O	-3.61934000	3.63608100	-0.39285400
	C	2.28191000	-2.02600800	0.64236500
	O	2.76449700	-3.13658800	0.89094600
	N	3.00763400	-1.03536800	0.06938100
	H	2.59507900	-0.14833900	-0.18255300
	C	4.41487300	-1.18567000	-0.24775900
	H	4.91093600	-1.70044800	0.57957700
	C	4.62886600	-2.03005400	-1.54147400
	H	4.03654000	-2.93276200	-1.35799600
	C	4.07431700	-1.32482400	-2.78263000
	H	4.11521200	-1.99312100	-3.64769500
	H	3.03266000	-1.02316000	-2.63944600
	H	4.65576900	-0.42916700	-3.02809400
	C	6.08743300	-2.45533500	-1.73167900
	H	6.48056600	-2.94266800	-0.83368500
	H	6.16208700	-3.16514800	-2.56126700
	H	6.73047600	-1.60114600	-1.95781700
	C	4.98373400	0.22274600	-0.37840800
	O	4.28059100	1.19649400	-0.59325700
	O	6.30849100	0.21683500	-0.26112300
	C	7.12038300	1.44667700	-0.44874100
	C	6.91845400	1.98009700	-1.86600700
	C	6.75111400	2.46816300	0.62555500
	C	8.54409200	0.93293700	-0.25306600
	H	7.13418400	1.19895400	-2.60072700
	H	5.89980000	2.33794100	-2.01413600
	H	7.61012900	2.80976300	-2.03798800
	H	6.84512600	2.02299100	1.62010800
	H	7.43853600	3.31675800	0.56462300
	H	5.73243600	2.83151100	0.49241300
	H	9.25215300	1.75877100	-0.36106900
	H	8.66373100	0.50002800	0.74359400
	H	8.78468600	0.16900700	-0.99723000
	C	-2.70149600	1.02740000	-0.74360700
	C	-5.58618900	-0.75123200	-1.28111000
	C	-6.83261400	-1.24809600	-0.90802800
	C	-7.98875600	-0.78997100	-1.54637000
	C	-7.88930100	0.16633500	-2.55677900
	C	-6.63835500	0.66572800	-2.92738100
	C	-5.47955500	0.20905000	-2.29546800
	H	-4.69525900	-1.10967900	-0.77381500
	H	-6.90135000	-1.99263400	-0.12082300
	H	-8.96058700	-1.17676000	-1.25604700
	H	-8.78330600	0.52759500	-3.05541700
	H	-6.56278600	1.41403900	-3.71123900
	C	-4.11564500	0.75451700	-2.67725900
	H	-4.19720000	1.40271400	-3.55264900
	H	-3.42998600	-0.06522200	-2.92250800
	N	-3.55353000	1.53379800	-1.57891400
	H	-2.31222800	0.02070800	-0.89368700
	H	-3.79277400	2.66728700	-1.17966500
**Int1**	C	4.50455500	-0.46592100	-2.36415300
	C	3.52094100	0.38238800	-1.90165900
	C	2.18019900	-0.06959000	-1.75322000
	C	1.86957700	-1.42352400	-2.10776900
	C	2.90653300	-2.26718400	-2.58998600
	C	4.19625100	-1.80146200	-2.71313000
	H	5.52267700	-0.10563500	-2.46735700
	H	3.75609700	1.41401100	-1.66127900
	C	1.13766500	0.77496600	-1.25059900

	C	0.54036100	-1.88880300	-1.96677000
	H	2.66096700	-3.29032400	-2.85787000
	H	4.98025600	-2.45615000	-3.07865400
	C	-0.45361100	-1.06535100	-1.48178600
	C	-0.14171400	0.27418300	-1.12891000
	H	0.29052400	-2.90828300	-2.23958800
	H	-0.91641100	0.94648100	-0.77519600
	C	1.44463400	2.17219100	-0.81934500
	C	1.71232800	2.44446400	0.53091800
	C	1.49673400	3.21653600	-1.75541600
	C	2.04688500	3.76694100	0.94683600
	C	2.05666200	4.73555400	-0.09784400
	H	2.30166600	5.76429000	0.16516600
	C	1.22244200	2.98178100	-3.22029800
	H	1.96227300	2.30958900	-3.67104200
	H	0.23996500	2.52563400	-3.38345000
	H	1.25706600	3.93549200	-3.74977000
	N	1.80021500	4.47511400	-1.37573400
	O	2.32432200	4.05804400	2.18401200
	H	1.95298700	2.81743500	2.79181300
	C	-1.83285400	-1.64500600	-1.35274700
	O	-2.15707100	-2.68042800	-1.94469700
	N	-2.68793500	-0.97741500	-0.54198100
	H	-2.40514700	-0.15714200	-0.02442000
	C	-4.06448900	-1.39358900	-0.35244000
	H	-4.47732900	-1.69976000	-1.31747800
	C	-4.17245100	-2.60206900	0.62718000
	H	-3.45986500	-3.32694800	0.21928300
	C	-3.73265400	-2.22886500	2.04587300
	H	-3.69136700	-3.12171200	2.67653300
	H	-2.74058200	-1.76805300	2.04946900
	H	-4.43351800	-1.52675800	2.51092100
	C	-5.56083100	-3.24746800	0.61239100
	H	-5.87388800	-3.49557300	-0.40685700
	H	-5.54644600	-4.17297300	1.19619400
	H	-6.31706300	-2.58671800	1.04343100
	C	-4.82121600	-0.17134200	0.15553300
	O	-4.25885800	0.77445500	0.68247300
	O	-6.13114300	-0.30926900	-0.02730200
	C	-7.10500800	0.69731800	0.46878700
	C	-6.99553000	0.80717300	1.98859600
	C	-6.86387900	2.02940800	-0.23918700
	C	-8.44289700	0.08974100	0.05654100
	H	-7.11348000	-0.17724300	2.45080500
	H	-6.03653700	1.22952300	2.28789100
	H	-7.79489600	1.45535000	2.35909600
	H	-6.88228800	1.89085900	-1.32391600
	H	-7.66181500	2.72751000	0.02969300
	H	-5.90659000	2.46308600	0.04873200
	H	-9.25799000	0.75066900	0.36272300
	H	-8.48898600	-0.04001000	-1.02795300
	H	-8.58757000	-0.88419000	0.53168700
	C	1.70422300	1.36545200	1.58129900
	O	1.63035800	1.81019300	2.86081600
	C	4.54394000	-2.19994300	0.80433200
	C	5.74502200	-2.83163700	0.48944900
	C	6.84951300	-2.69081500	1.33261600
	C	6.74992300	-1.91451700	2.48872500
	C	5.54934000	-1.27556800	2.80016600
	C	4.43939100	-1.41621900	1.96039000

	H	3.68741700	-2.31415200	0.14659600
	H	5.81598900	-3.43236500	-0.41135600
	H	7.78439300	-3.18614500	1.09041200
	H	7.60557500	-1.80566700	3.14727600
	H	5.47192600	-0.66822100	3.69740900
	C	3.15726400	-0.68457800	2.24721500
	H	3.06299400	-0.37378700	3.28716200
	H	2.28238800	-1.27907700	1.97466900
	N	3.07923900	0.56872000	1.42228400
	H	3.82617900	1.21334200	1.69518100
	H	3.23493300	0.33839800	0.43402900
	H	0.97050300	0.57845500	1.37608100
**Int2**	C	2.85112300	-3.31937200	1.21933200
	C	2.05534400	-2.68017200	0.29628700
	C	0.68557500	-2.42111400	0.56487000
	C	0.15338900	-2.81631000	1.83594800
	C	1.00677800	-3.45575400	2.77697100
	C	2.32473100	-3.70952200	2.47494400
	H	3.89254400	-3.51978600	0.98665000
	H	2.46223900	-2.36873600	-0.65683900
	C	-0.17815200	-1.78948900	-0.38864100
	C	-1.20715800	-2.56071600	2.12918000
	H	0.59341500	-3.74747800	3.73792400
	H	2.96477200	-4.20685900	3.19665500
	C	-2.02527700	-1.93933500	1.20890300
	C	-1.50025200	-1.57057500	-0.05673500
	H	-1.62038200	-2.85300100	3.08853500
	H	-2.14208200	-1.12410300	-0.80908700
	C	0.34304400	-1.34811000	-1.71448300
	C	0.44144400	0.01328500	-2.01473300
	C	0.75639400	-2.29812600	-2.67809600
	C	1.00246800	0.43987700	-3.26345900
	C	1.42380200	-0.61371200	-4.11963800
	H	1.87176200	-0.34663700	-5.07726800
	C	0.59789600	-3.78515200	-2.46312400
	H	1.38897100	-4.20534000	-1.82982400
	H	-0.35432100	-4.02673800	-1.98058200
	H	0.64180900	-4.29111600	-3.42987200
	N	1.29330000	-1.91473200	-3.84860600
	O	1.12482800	1.69901500	-3.55714200
	H	-0.24301000	2.24048400	-2.57821100
	C	-3.44866600	-1.68035400	1.60544100
	O	-3.98207300	-2.27747500	2.54796800
	N	-4.10605500	-0.74018700	0.88358900
	H	-3.65105400	-0.21740700	0.14842000
	C	-5.49204800	-0.39701600	1.13556800
	H	-6.05249800	-1.31435400	1.33449100
	C	-5.63766300	0.53821700	2.37512400
	H	-5.08591500	0.00979900	3.16008000
	C	-4.97592600	1.89999800	2.14440400
	H	-4.97888500	2.48387100	3.06955200
	H	-3.93757700	1.79047700	1.81845300
	H	-5.50901800	2.48091200	1.38369200
	C	-7.09066400	0.68209900	2.83618400
	H	-7.56155300	-0.29551800	2.98117900
	H	-7.12677400	1.21926400	3.78901100
	H	-7.69039700	1.23802100	2.11115600
	C	-6.02076200	0.25094100	-0.13884100
	O	-5.28598300	0.74910800	-0.97488900
	O	-7.35087100	0.21919900	-0.17539900

	C	-8.12157600	0.87422400	-1.26260100
	C	-7.82876700	2.37370800	-1.26441000
	C	-7.78996300	0.20607100	-2.59589800
	C	-9.56575700	0.59742100	-0.85392800
	H	-8.02094900	2.80006700	-0.27549800
	H	-6.79552000	2.57606900	-1.54550700
	H	-8.49046600	2.86673000	-1.98242200
	H	-7.94684400	-0.87418500	-2.52760000
	H	-8.45585300	0.59985700	-3.36920200
	H	-6.75836400	0.39860100	-2.88930800
	H	-10.24675100	1.03048500	-1.59127400
	H	-9.75095600	-0.47861600	-0.79897700
	H	-9.78262000	1.03970400	0.12212300
	C	0.05468200	1.14625500	-1.07800800
	O	-0.76468200	2.08892700	-1.74683500
	C	2.82838300	3.56397100	2.05972100
	C	4.11611000	3.88822900	2.49459400
	C	5.14966800	4.03716000	1.56966600
	C	4.87910000	3.89174100	0.20581600
	C	3.58977200	3.58235700	-0.22348700
	C	2.55154400	3.38934500	0.70008100
	H	2.03135800	3.43108000	2.78627800
	H	4.31133100	4.01271800	3.55530800
	H	6.15383500	4.27680800	1.90495400
	H	5.67265100	4.02314600	-0.52352800
	H	3.39661900	3.47888100	-1.28755200
	C	1.18329000	2.93790800	0.23516000
	H	0.66251700	3.73826600	-0.30678800
	H	0.56401400	2.68125500	1.10080000
	N	1.32049200	1.73522700	-0.60810700
	H	1.79018900	2.01195400	-1.47360000
	H	2.41543700	0.60732800	0.21195900
	H	-0.49905300	0.80131800	-0.20293200
	C	6.36537900	-1.09168700	-2.18335300
	C	7.10814700	-1.06538900	-0.84332400
	C	6.84695900	0.19144600	-0.00675500
	C	4.84155200	-1.00046600	-2.07422200
	C	4.33182400	0.29536700	-1.40740900
	C	4.39441800	0.24806700	0.09437100
	H	6.72376600	-0.25677400	-2.80016600
	H	6.85002000	-1.94487700	-0.24083100
	H	6.88975500	1.09098600	-0.63029000
	H	4.44989800	-1.86516600	-1.52618300
	H	3.29205400	0.44892400	-1.68936300
	H	6.63039600	-2.00879500	-2.72025200
	H	8.18534500	-1.11823500	-1.03534000
	H	7.62020700	0.30111300	0.75382800
	H	4.39970600	-1.04587400	-3.07397100
	H	4.90009800	1.15846500	-1.77025600
	C	5.60795500	0.18573300	2.19588400
	H	5.66085900	1.22939600	2.52844400
	H	6.52160100	-0.32112400	2.50968100
	C	4.38279200	-0.51518900	2.77074000
	H	4.37917800	-0.42059200	3.85893800
	H	4.42833700	-1.57926200	2.52654900
	C	3.11242300	0.09108700	2.19088800
	H	2.90797100	1.07112100	2.63138700
	H	2.25159700	-0.55638400	2.37572000
	N	5.56653800	0.16389100	0.72332800
	N	3.24668400	0.24408600	0.74515600

**Int3**	C	4.25266900	3.75327200	-1.11329000
	C	3.53919000	2.97237900	-0.23146400
	C	2.15069800	2.74684400	-0.42193500
	C	1.50772500	3.33525700	-1.55976100
	C	2.27255000	4.13792500	-2.44844500
	C	3.61479000	4.34550800	-2.22997000
	H	5.31385200	3.91603500	-0.95388400
	H	4.03182900	2.51670100	0.62007300
	C	1.37100300	1.95116800	0.47687000
	C	0.13062600	3.09470700	-1.78162300
	H	1.77562300	4.58178600	-3.30594000
	H	4.19039900	4.96038300	-2.91431200
	C	-0.59725200	2.29131200	-0.92951600
	C	0.03500800	1.72979300	0.21050100
	H	-0.36505000	3.53654000	-2.63944500
	H	-0.53954800	1.14645700	0.92270900
	C	1.98758800	1.35558600	1.69656600
	C	2.26117600	-0.04815100	1.73833100
	C	2.29602700	2.13988100	2.79549700
	C	2.84694500	-0.63027900	2.92755900
	C	3.11645600	0.31743400	3.98548100
	H	3.56143500	-0.07347600	4.90032800
	C	2.04737900	3.62631300	2.83355800
	H	2.88466400	4.18509600	2.39746700
	H	1.14997700	3.90152500	2.27417500
	H	1.93560400	3.95054800	3.87019400
	N	2.85933200	1.60110900	3.92308300
	O	3.11545400	-1.86648800	3.05228200
	C	-2.04083600	2.04902000	-1.25585100
	O	-2.67918800	2.80568300	-1.99571500
	N	-2.59106400	0.93857600	-0.70614600
	H	-2.04426700	0.29215700	-0.15490200
	C	-3.97933900	0.57154300	-0.90780700
	H	-4.60023400	1.46733800	-0.82031000
	C	-4.21434000	-0.04599400	-2.32075200
	H	-3.76185500	0.68570000	-2.99828900
	C	-3.48651000	-1.38306700	-2.48808200
	H	-3.55337300	-1.72341700	-3.52551000
	H	-2.42693600	-1.29665400	-2.23001500
	H	-3.92721500	-2.16121200	-1.85492400
	C	-5.70005800	-0.15774300	-2.67327600
	H	-6.21418600	0.79919300	-2.53765000
	H	-5.81217300	-0.45588100	-3.72024300
	H	-6.20690800	-0.90108400	-2.05294700
	C	-4.33664000	-0.40207600	0.20973800
	O	-3.49099300	-1.02663400	0.82934000
	O	-5.65400500	-0.48647800	0.36946400
	C	-6.27071100	-1.44286400	1.32475500
	C	-5.89261800	-2.87041000	0.93329300
	C	-5.84283500	-1.08638500	2.74735100
	C	-7.76254200	-1.19433100	1.12054700
	H	-6.15752900	-3.06144100	-0.11073200
	H	-4.82647100	-3.05037000	1.06959200
	H	-6.44975800	-3.57269500	1.55996500
	H	-6.07011100	-0.03785500	2.95962100
	H	-6.39973600	-1.70678800	3.45540900
	H	-4.77661400	-1.25681300	2.89449200
	H	-8.33871200	-1.84417200	1.78436500
	H	-8.01423300	-0.15480800	1.34657200
	H	-8.05194500	-1.40751600	0.08804800

	C	2.00958400	-0.87852400	0.62149800
	C	4.40435300	-2.64330500	-1.44994300
	C	5.63457100	-3.09531600	-1.92214300
	C	5.91042300	-4.46508700	-1.95895400
	C	4.95141400	-5.37771900	-1.52032100
	C	3.71985300	-4.92265600	-1.04443700
	C	3.43674000	-3.55513100	-1.00985400
	H	4.19571300	-1.57749400	-1.41918000
	H	6.37805500	-2.38101600	-2.26167300
	H	6.86940000	-4.81681900	-2.32621000
	H	5.16083500	-6.44254300	-1.54299900
	H	2.97512000	-5.63376800	-0.69831400
	C	2.09787800	-3.05898300	-0.50246700
	H	1.47201700	-3.90333300	-0.20091200
	H	1.56186600	-2.50882500	-1.28256500
	N	2.25412300	-2.16767200	0.64067000
	H	2.66109600	-2.48522600	1.54826600
	H	1.60669900	-0.45855900	-0.29647300
**12**	C	-4.25218700	-1.49608100	2.24282700
	C	-3.36308000	-0.48922600	1.93697800
	C	-1.99501000	-0.77883000	1.68824900
	C	-1.55317100	-2.13891300	1.78644200
	C	-2.49679400	-3.15294900	2.10243700
	C	-3.81920700	-2.84114600	2.32046200
	H	-5.29505800	-1.25652100	2.42279900
	H	-3.70595100	0.53695200	1.88069000
	C	-1.04051400	0.23158800	1.33783600
	C	-0.18978600	-2.44459400	1.56028000
	H	-2.15196100	-4.18054000	2.16777900
	H	-4.53272900	-3.62332400	2.55849800
	C	0.71573500	-1.46000700	1.22566000
	C	0.27544800	-0.11499600	1.11755900
	H	0.15613500	-3.46922500	1.64412900
	H	0.97900400	0.67518900	0.87740500
	C	-1.47280500	1.65097500	1.19121600
	C	-2.26458200	2.03196300	0.08716800
	C	-1.14046000	2.61231100	2.15732700
	C	-2.71897100	3.36721200	0.01059600
	C	-2.30849700	4.25447900	1.01601000
	H	-2.63329500	5.29139300	0.96541600
	C	-0.32345400	2.26510200	3.37677400
	H	-0.68397700	1.34786900	3.85156600
	H	0.73057100	2.10258600	3.12639400
	H	-0.38059700	3.08604400	4.09302500
	N	-1.54952200	3.89195900	2.04732500
	O	-3.51542400	3.80650300	-0.97635800
	C	2.13820000	-1.87361500	0.98779200
	O	2.58293600	-2.94433800	1.41633200
	N	2.89698800	-1.01273600	0.26701500
	H	2.51242900	-0.16822100	-0.13219300
	C	4.29795400	-1.25936500	-0.01558700
	H	4.77766200	-1.64793000	0.88692300
	C	4.48150400	-2.31050800	-1.15319600
	H	3.85404000	-3.14808400	-0.83047300
	C	3.95723200	-1.79604400	-2.49702100
	H	3.97158300	-2.59793300	-3.24091600
	H	2.92862300	-1.43339900	-2.41318300
	H	4.57512600	-0.97677900	-2.88122200
	C	5.92280700	-2.81608600	-1.25978100
	H	6.29394100	-3.16831300	-0.29197500

	H	5.97205600	-3.65172000	-1.96470800
	H	6.59935300	-2.03411300	-1.61349200
	C	4.91299900	0.08912000	-0.37296300
	O	4.24185400	1.03779300	-0.74487300
	O	6.23728000	0.05803600	-0.25437300
	C	7.08898100	1.21250900	-0.64044900
	C	6.89814300	1.51277100	-2.12623200
	C	6.75890300	2.40830900	0.25123500
	C	8.49539700	0.68990000	-0.36108800
	H	7.08378600	0.61493100	-2.72288500
	H	5.89117600	1.87549000	-2.33144000
	H	7.61604500	2.27930800	-2.43191800
	H	6.84301800	2.12965700	1.30546900
	H	7.47370600	3.21193900	0.05214200
	H	5.75215800	2.77869300	0.05961700
	H	9.23062700	1.46261800	-0.60037800
	H	8.60374400	0.42119800	0.69302400
	H	8.70763300	-0.19272300	-0.97032600
	C	-2.62281300	1.07137600	-0.95306200
	C	-5.17096300	-1.18947900	-1.28627400
	C	-6.36475600	-1.75984300	-0.85041600
	C	-7.56228500	-1.46383800	-1.50742000
	C	-7.55650100	-0.59484500	-2.59893500
	C	-6.35804600	-0.02285300	-3.03226600
	C	-5.15690200	-0.31762700	-2.38249200
	H	-4.24705800	-1.41935300	-0.76452500
	H	-6.35859000	-2.43394400	0.00052300
	H	-8.49340700	-1.90824000	-1.16967900
	H	-8.48336500	-0.35981900	-3.11310300
	H	-6.35574200	0.65616200	-3.88031200
	C	-3.85008400	0.32028100	-2.81694700
	H	-3.96393600	0.77506100	-3.80425700
	H	-3.06423900	-0.44507100	-2.87997100
	N	-3.47255400	1.36781200	-1.86767900
	H	-2.14253100	0.08939600	-0.91626500
	H	-3.73212300	3.00526400	-1.54095400
**13**	C	-3.48123500	-4.43370200	-1.18244100
	C	-2.90795400	-3.53478100	-0.31088900
	C	-1.58009300	-3.07443400	-0.51124500
	C	-0.84590000	-3.56184500	-1.64116800
	C	-1.46578900	-4.49080200	-2.52063000
	C	-2.75434700	-4.91817200	-2.29755400
	H	-4.49863900	-4.77461400	-1.01719400
	H	-3.45998100	-3.15961600	0.54335400
	C	-0.95605400	-2.14640800	0.38389400
	C	0.47650800	-3.10596700	-1.85644600
	H	-0.90100000	-4.85610600	-3.37365700
	H	-3.21894700	-5.62739000	-2.97554400
	C	1.05973100	-2.19355100	-1.00178400
	C	0.33137200	-1.72490600	0.12432400
	H	1.04417200	-3.46869100	-2.70668500
	H	0.79132700	-1.04234600	0.83171200
	C	-1.68235300	-1.64914500	1.58744100
	C	-2.55178300	-0.52848700	1.46529800
	C	-1.51352500	-2.29219800	2.82301200
	C	-3.20422200	-0.12663400	2.67658600
	C	-2.95922800	-0.82725900	3.84604100
	H	-3.46463000	-0.50494100	4.75593200
	C	-0.60657400	-3.49513200	2.95031500
	H	-0.87979700	-4.28359900	2.24004000

	H	0.44037000	-3.24100700	2.74938100
	H	-0.67873000	-3.89397500	3.96392800
	N	-2.13701000	-1.89398100	3.94502100
	O	-4.05815200	0.93067600	2.68944900
	H	-4.07123500	1.25957700	1.73453500
	C	-2.76913100	0.15078100	0.23180200
	H	-2.24354900	-0.18767200	-0.66331300
	N	-3.60886700	1.19402700	0.16066800
	C	-3.86061400	1.86746400	-0.94739100
	H	-3.36377400	1.57670000	-1.88037000
	C	-4.76960800	2.98132300	-1.00044100
	C	-4.98403200	3.65816400	-2.22754600
	C	-5.48616400	3.45770600	0.12812200
	C	-5.85436700	4.73847600	-2.32112400
	H	-4.45049000	3.31745600	-3.11196800
	C	-6.35379700	4.53803700	0.02589300
	H	-5.35172400	2.96602400	1.08571700
	C	-6.55112000	5.19370000	-1.19627500
	H	-5.99198500	5.23158300	-3.28032700
	H	-6.88652800	4.87623200	0.91145100
	H	-7.23055400	6.03726700	-1.26887300
	C	2.45373500	-1.74099000	-1.31408400
	O	3.19491700	-2.37563500	-2.07512300
	N	2.85180500	-0.58519700	-0.72554100
	H	2.22414700	-0.03758100	-0.15419900
	C	4.17863700	-0.03525300	-0.91932500
	H	4.90575700	-0.85127300	-0.89088600
	C	4.31069600	0.68118200	-2.29873100
	H	3.94803800	-0.06850800	-3.01009100
	C	3.41099500	1.91705600	-2.39021500
	H	3.42161500	2.31866900	-3.40777900
	H	2.37501600	1.67761300	-2.13413600
	H	3.75114300	2.71156500	-1.71666100
	C	5.76344800	1.00675200	-2.65631800
	H	6.40297200	0.12223500	-2.57077200
	H	5.82091900	1.36674600	-3.68826500
	H	6.17492300	1.78131400	-2.00417300
	C	4.43889400	0.91099700	0.24644900
	O	3.54044400	1.40248900	0.90826900
	O	5.74308500	1.13446000	0.39894400
	C	6.26124300	2.10146700	1.39869500
	C	5.71986300	3.49646800	1.08972800
	C	5.89777900	1.62504400	2.80418300
	C	7.76879700	2.03479400	1.16909400
	H	5.94781600	3.77263100	0.05606500
	H	4.64208600	3.54658600	1.24255100
	H	6.20208700	4.22260800	1.75054500
	H	6.24422700	0.59905900	2.95771500
	H	6.39211700	2.26644800	3.53957800
	H	4.82155900	1.66591600	2.96983900
	H	8.27805100	2.70930800	1.86245200
	H	8.13907400	1.01976800	1.33591600
	H	8.01745200	2.33369100	0.14721900
**14**	C	-1.22337100	-1.73711600	-0.10185200
	C	-2.01069300	-0.70728200	-0.49051100
	C	-3.49723100	-0.82837000	-0.49234200
	O	-4.21486000	0.11307800	-0.80428000
	O	-3.92240900	-2.03420300	-0.10552100
	C	-5.36318000	-2.36151100	0.00339700
	C	-5.33474200	-3.81341100	0.47415500

	H	-4.82173400	-4.44478500	-0.25629000
	H	-4.81786400	-3.89732600	1.43399800
	H	-6.35614100	-4.18305400	0.59603500
	C	-6.02245300	-2.24360800	-1.36998500
	H	-7.05025500	-2.61248000	-1.30732600
	H	-6.04078200	-1.20939300	-1.71251900
	H	-5.48488200	-2.85369900	-2.10153300
	C	-6.01336200	-1.45819600	1.05057400
	H	-6.02870400	-0.41930200	0.72203000
	H	-7.04200300	-1.78839200	1.22119000
	H	-5.47158000	-1.52674000	1.99842300
	C	-1.49006800	0.58321800	-1.06167100
	H	-2.17784900	0.90950200	-1.84432100
	C	0.23301900	-1.76635300	-0.00120500
	C	1.00898800	-0.64530700	0.34903700
	C	0.90286000	-2.97979300	-0.24583700
	C	2.39390700	-0.72552400	0.40542800
	H	0.52415800	0.28049400	0.63066900
	C	2.28891000	-3.05192500	-0.21038300
	H	0.32479100	-3.86374800	-0.49752700
	C	3.06545200	-1.92375600	0.10753400
	H	2.96369400	0.14351000	0.71689100
	H	2.77928700	-3.98885400	-0.45123200
	H	-0.51136300	0.38708500	-1.49441000
	H	-1.72879000	-2.66907200	0.13444600
	C	4.54306800	-1.99451600	0.14075800
	C	5.32046200	-0.89669300	-0.26483900
	C	5.20108200	-3.15793600	0.57272400
	C	6.71196500	-0.96087800	-0.24001400
	H	4.83079500	0.00137100	-0.62774500
	C	6.59271600	-3.22093700	0.59846500
	H	4.61907600	-4.00761000	0.91456500
	C	7.35377100	-2.12318500	0.19195100
	H	7.29551600	-0.10576900	-0.56654400
	H	7.08320500	-4.12575100	0.94342200
	H	8.43777400	-2.17326700	0.21111500
	C	2.30159800	4.08922900	-1.14420900
	C	1.81103000	4.70743100	0.16948000
	C	0.30488600	4.99040900	0.19525500
	C	1.61602000	2.77834600	-1.54183700
	C	0.07968800	2.90252900	-1.68428200
	C	-0.63716700	2.78879100	-0.36323400
	H	2.14639000	4.81794100	-1.95038700
	H	2.05995400	4.06201300	1.02068800
	H	-0.03244900	5.42665000	-0.74862100
	H	1.85384100	1.98353700	-0.82642000
	H	-0.29560100	2.15499600	-2.37520800
	H	3.38178700	3.92184700	-1.08138900
	H	2.33294700	5.65640900	0.33005800
	H	0.06602600	5.71426900	0.97406200
	H	2.01102900	2.45111200	-2.50777700
	H	-0.17529300	3.86872700	-2.12932700
	C	-1.05867800	3.75244300	1.87233800
	H	-1.95258000	4.38453100	1.89837000
	H	-0.31435800	4.19391900	2.53821700
	C	-1.37160200	2.32906000	2.28903700
	H	-1.97146800	2.33492400	3.20124400
	H	-0.44524900	1.78614600	2.50107900
	C	-2.12869700	1.62621300	1.17695100
	H	-3.11417500	2.07063400	1.00773800

	H	-2.26902700	0.57583200	1.42191800
	N	-0.50381800	3.79115100	0.50568700
	N	-1.35033000	1.69521700	-0.07261300
**$PhCH_2NH_2$**	C	-0.24783000	-1.20442200	-0.18802200
	C	-1.61557500	-1.20720300	0.08793400
	C	-2.30326600	0.00010300	0.22771700
	C	-1.61540200	1.20732000	0.08782000
	C	-0.24768000	1.20432100	-0.18813600
	C	0.45412200	-0.00011800	-0.32711000
	H	0.28327000	-2.14654200	-0.30060800
	H	-2.14541800	-2.14979600	0.18870400
	H	-3.36819300	0.00019400	0.43900800
	H	-2.14513900	2.14998300	0.18850000
	H	0.28357100	2.14634400	-0.30083000
	C	1.95245200	-0.00016300	-0.55566200
	H	2.23611500	0.88029000	-1.14228300
	H	2.23614200	-0.88091400	-1.14182200
	N	2.76626000	0.00015200	0.67551900
	H	2.49730900	0.80763600	1.23662100
	H	2.49759800	-0.80728500	1.23682800
**Complex**	C	2.76438300	-5.84137600	2.39199000
	C	2.30350400	-4.54804600	2.49693200
	C	3.00697700	-3.47368200	1.89155200
	C	4.22315900	-3.75766200	1.18352300
	C	4.67234800	-5.10297900	1.09964900
	C	3.95866200	-6.12330100	1.68524800
	H	2.21062900	-6.65267800	2.85413300
	H	1.38983200	-4.33010800	3.03818400
	C	2.54740200	-2.12072500	1.97174900
	C	4.94434200	-2.69911200	0.57392400
	H	5.59116300	-5.31083600	0.55877100
	H	4.31001800	-7.14760200	1.60996200
	C	4.45781600	-1.41177000	0.61597800
	C	3.26414700	-1.13629900	1.32827300
	H	5.86947900	-2.91305400	0.04747200
	H	2.92523600	-0.10912700	1.39761500
	C	1.35922300	-1.71942800	2.78032200
	C	0.03685800	-1.96334800	2.31588100
	C	1.57055700	-1.02513400	3.98462500
	C	-1.01339800	-1.42880200	3.12937800
	C	-0.68979300	-0.74511300	4.29545500
	H	-1.50346500	-0.35306400	4.90371900
	C	2.96293600	-0.78090400	4.52112500
	H	3.58629200	-1.67758600	4.45482800
	H	3.48419300	0.00746400	3.96652500
	H	2.89140500	-0.47117700	5.56545900
	N	0.56249900	-0.54416300	4.73462400
	O	-2.31594800	-1.56319100	2.78681000
	H	-2.29374300	-2.09316500	1.91611500
	C	-0.25451300	-2.65368900	1.09166600
	H	0.55810200	-3.06019200	0.49227400
	N	-1.53066000	-2.82088000	0.72502900
	C	-1.90804500	-3.32549300	-0.43726200
	C	5.18028700	-0.28100200	-0.05529200
	O	6.40886900	-0.15634300	-0.02845200
	N	4.33856400	0.60172000	-0.65092000
	H	3.38056700	0.30673100	-0.81467400
	C	4.74420700	1.89624100	-1.14319700
	H	5.73159300	2.11198500	-0.72910300
	C	4.84457100	1.93263900	-2.69742300

	H	5.44452800	1.04682500	-2.93910700
	C	3.47482400	1.78481600	-3.37088100
	H	3.59754200	1.67340800	-4.45251400
	H	2.92868100	0.91340300	-3.00023400
	H	2.85652700	2.67410300	-3.20168100
	C	5.59082400	3.16877600	-3.20849600
	H	6.56945900	3.27089400	-2.72881600
	H	5.74907900	3.09017300	-4.28855000
	H	5.02631100	4.08474500	-3.01490500
	C	3.74328700	2.92227500	-0.60837700
	O	2.60702700	2.63398600	-0.26937500
	O	4.27118800	4.14685800	-0.59411200
	C	3.48980800	5.33068200	-0.16196800
	C	2.27056000	5.50669100	-1.06642100
	C	3.10882300	5.17334800	1.30958200
	C	4.48340700	6.47273100	-0.35417000
	H	2.57351200	5.52352300	-2.11719600
	H	1.55272200	4.70269200	-0.91398400
	H	1.78644400	6.46002300	-0.83484800
	H	4.00191700	5.01836500	1.92095800
	H	2.61147400	6.08461800	1.65419400
	H	2.43596100	4.32770700	1.44988200
	H	4.02617700	7.41683300	-0.04652900
	H	5.38001800	6.30712900	0.24865400
	H	4.77833500	6.55432100	-1.40375300
	C	0.19336400	0.36560000	-0.11566700
	C	-0.14676900	-0.68145200	-1.13747600
	C	1.01239400	-1.35481800	-1.73067200
	O	2.17583700	-0.96065700	-1.61614400
	O	0.67878200	-2.46166200	-2.41973400
	C	1.69181400	-3.32547900	-3.05022600
	C	0.83699000	-4.41533700	-3.69487500
	H	0.25232500	-4.94341700	-2.93651900
	H	0.14899100	-3.98308600	-4.42709500
	H	1.47812200	-5.13930500	-4.20495700
	C	2.60255000	-3.91180900	-1.97226500
	H	3.27833100	-4.64413900	-2.42427800
	H	3.19623300	-3.13594500	-1.49288900
	H	2.01033500	-4.42330200	-1.20790400
	C	2.46750800	-2.54489700	-4.11254200
	H	3.10014500	-1.78338400	-3.65838600
	H	3.09652200	-3.23646600	-4.68102900
	H	1.77399600	-2.06397300	-4.80911400
	C	-1.40176100	-0.96906900	-1.61244200
	H	-1.44704200	-1.61926800	-2.47762900
	H	1.27258500	0.38563100	0.01794100
	C	-1.89824800	3.84212100	3.18773800
	C	-2.19242000	4.80608000	2.03429900
	C	-1.01007800	4.99254000	1.07754900
	C	-1.46155300	2.43936600	2.75731600
	C	-0.17610600	2.41657300	1.89899000
	C	-0.43341600	2.68469000	0.43881700
	H	-1.10789600	4.27664400	3.81421900
	H	-3.06036100	4.46426500	1.45668400
	H	-0.07277700	5.10515000	1.62890800
	H	-2.27005100	1.94092900	2.21255400
	H	0.33538500	1.46665900	2.01922400
	H	-2.78644200	3.75928700	3.82294500
	H	-2.45310200	5.78719500	2.44531100
	H	-1.13430200	5.90334000	0.49020700

	H	-1.26849600	1.83378000	3.64573800
	H	0.52675000	3.17430500	2.26000800
	C	-1.39896300	4.23135500	-1.24702500
	H	-0.73139100	4.97613700	-1.69301200
	H	-2.37676500	4.70134300	-1.10717500
	C	-1.50754200	2.99825300	-2.12630200
	H	-1.57546700	3.30033500	-3.17364900
	H	-2.40141700	2.41932800	-1.87975400
	C	-0.27939000	2.13214100	-1.89373000
	H	0.64621400	2.67031300	-2.12445700
	H	-0.30982200	1.22500800	-2.48946500
	N	-0.87323100	3.90204400	0.09320100
	N	-0.24275700	1.74414900	-0.47880900
	H	-0.25714300	0.11409800	0.83778500
	H	-1.15744400	-3.71533900	-1.12688300
	C	-3.29753300	-3.43612600	-0.81919300
	C	-3.62454500	-3.91973700	-2.10517500
	C	-4.36126100	-3.01197200	0.00884300
	C	-4.94431100	-3.96911100	-2.54478900
	H	-2.82185000	-4.24770800	-2.76157300
	C	-5.67622500	-3.05743000	-0.43751900
	H	-4.14613300	-2.62779200	0.99834100
	C	-5.98327700	-3.53661800	-1.71574400
	H	-5.16389900	-4.34477900	-3.54064000
	H	-6.47219000	-2.70792300	0.21423600
	H	-7.01296200	-3.57129100	-2.05837000
	C	-2.67546600	-0.38293800	-1.23354000
	C	-2.95952400	0.13404400	0.04489200
	C	-3.72498400	-0.40346500	-2.17311100
	C	-4.22323800	0.61380800	0.35865900
	H	-2.22105400	0.07886700	0.82955200
	C	-4.98461300	0.08495200	-1.86128500
	H	-3.54014800	-0.82157900	-3.15724600
	C	-5.26272800	0.60373400	-0.58621100
	H	-4.42479300	0.94988500	1.37098800
	H	-5.76648400	0.06495100	-2.61314100
	C	-6.61817800	1.07421800	-0.23118500
	C	-6.80225400	2.17711400	0.61942300
	C	-7.75686500	0.41620200	-0.72697000
	C	-8.08207500	2.61071400	0.96014000
	H	-5.93424000	2.70837500	0.99774300
	C	-9.03648000	0.85112900	-0.38742100
	H	-7.63154700	-0.45943000	-1.35587700
	C	-9.20514900	1.95069500	0.45692200
	H	-8.20301700	3.46939500	1.61366500
	H	-9.90355300	0.32388000	-0.77370400
	H	-10.20208800	2.28820000	0.72250200
**TS1-SR**	C	4.18780100	-5.28821000	2.00518300
	C	3.43613800	-4.15640400	2.22985900
	C	3.81654400	-2.90826500	1.67034700
	C	5.01333400	-2.84199500	0.88044100
	C	5.76709700	-4.02805100	0.67370100
	C	5.36324600	-5.22543700	1.21854300
	H	3.87943900	-6.23649000	2.43410000
	H	2.53735400	-4.20509900	2.83451200
	C	3.05347700	-1.71540900	1.87099800
	C	5.41207700	-1.60444400	0.31370900
	H	6.66902000	-3.97140200	0.07113800
	H	5.94558900	-6.12559000	1.04906700
	C	4.63244300	-0.48100400	0.47220800

	C	3.45965400	-0.54805800	1.26450800
	H	6.32277300	-1.55321200	-0.27491100
	H	2.88556500	0.35781200	1.42144800
	C	1.86547500	-1.66900300	2.77306900
	C	0.60428100	-2.15506400	2.35843700
	C	1.99852000	-1.06098800	4.03445700
	C	-0.48442100	-1.97054700	3.25631600
	C	-0.24750200	-1.33241000	4.47505600
	H	-1.08381800	-1.18727300	5.15628000
	C	3.33208700	-0.55540600	4.53093700
	H	4.12735300	-1.28833200	4.36579600
	H	3.63658100	0.36289600	4.01692300
	H	3.25793600	-0.34142900	5.59848300
	N	0.95317300	-0.89530900	4.86522600
	O	-1.73414500	-2.38141700	2.96098600
	H	-1.65326500	-2.79816700	2.03181800
	C	0.38610400	-2.76083600	1.05658000
	H	1.23400800	-2.90008500	0.38812600
	N	-0.82470100	-3.15400500	0.72644600
	C	-1.11879400	-3.46367600	-0.57126900
	C	5.01415200	0.82801700	-0.15516500
	O	6.17760800	1.24440900	-0.17842800
	N	3.94790400	1.50932100	-0.64245800
	H	3.09005300	0.98564600	-0.81463500
	C	4.02207300	2.87385400	-1.10691000
	H	4.95127100	3.30172100	-0.72356100
	C	4.04456700	2.96987400	-2.66262000
	H	4.79689800	2.22802700	-2.95705100
	C	2.70247500	2.56887200	-3.28688800
	H	2.79522100	2.51320100	-4.37596100
	H	2.35967400	1.59621700	-2.92428700
	H	1.92942900	3.31372600	-3.06288800
	C	4.50960100	4.34135500	-3.16153000
	H	5.47014000	4.62267700	-2.71798700
	H	4.63140800	4.32167400	-4.24909100
	H	3.78549300	5.12216700	-2.91546200
	C	2.83950600	3.63116000	-0.50111400
	O	1.83178100	3.08296500	-0.08826900
	O	3.05720600	4.94865600	-0.51732200
	C	2.03708100	5.91470200	-0.04749900
	C	0.76678200	5.77419700	-0.88518000
	C	1.78165500	5.69765200	1.44349900
	C	2.71141600	7.26051900	-0.29840800
	H	1.00112600	5.85874500	-1.95017900
	H	0.28212500	4.81725500	-0.70066400
	H	0.07255500	6.57796400	-0.62292100
	H	2.71733000	5.76768700	2.00494000
	H	1.10265300	6.47254300	1.81100300
	H	1.33525300	4.72037600	1.62513500
	H	2.05382800	8.07036600	0.02850900
	H	3.65099000	7.32910100	0.25599100
	H	2.92344900	7.39273400	-1.36278300
	C	0.00380100	0.19791800	-0.01098300
	C	-0.05520700	-0.86359300	-1.05589000
	C	1.17006200	-1.10844300	-1.75562900
	O	2.20907500	-0.42834000	-1.66548500
	O	1.10666200	-2.20993800	-2.56026500
	C	2.27729400	-2.71867100	-3.27402900
	C	1.71862100	-3.94452200	-3.99758100
	H	1.32090900	-4.66574300	-3.27769000

	H	0.91365000	-3.65465500	-4.67935400
	H	2.50711700	-4.43200800	-4.57779400
	C	3.35714600	-3.12875700	-2.27144700
	H	4.18612200	-3.61397600	-2.79627800
	H	3.73758600	-2.26242700	-1.73423900
	H	2.95205900	-3.84071900	-1.54568000
	C	2.78554900	-1.68607100	-4.28374200
	H	3.20927100	-0.82125500	-3.77552200
	H	3.55205600	-2.14125900	-4.91883000
	H	1.96410100	-1.35328900	-4.92602800
	C	-1.23244500	-1.58838500	-1.41523300
	H	-1.19677300	-2.00212600	-2.41905300
	H	1.04624800	0.45147600	0.17067800
	C	-2.60105900	3.01447300	3.43151100
	C	-3.19857400	3.88809800	2.32246000
	C	-2.15965700	4.42049000	1.32760900
	C	-1.82096200	1.79058400	2.94201000
	C	-0.63701300	2.14097600	2.01371100
	C	-1.05277100	2.35275100	0.58076300
	H	-1.92918100	3.63490600	4.03955500
	H	-3.96436100	3.33435100	1.76517800
	H	-1.26150100	4.76799000	1.84560800
	H	-2.49037500	1.09447700	2.42656200
	H	0.12303300	1.36798800	2.06201900
	H	-3.40428300	2.68306600	4.09822400
	H	-3.70081400	4.74703900	2.78039100
	H	-2.55630300	5.28143300	0.78772100
	H	-1.41834700	1.25181900	3.80431200
	H	-0.14767400	3.05593800	2.36078100
	C	-2.43781100	3.65146600	-1.01420600
	H	-1.96385900	4.51760900	-1.48871300
	H	-3.48352500	3.90795700	-0.82142400
	C	-2.34216700	2.41806900	-1.89483700
	H	-2.54416500	2.69348800	-2.93255000
	H	-3.08074800	1.67269100	-1.59305900
	C	-0.94963400	1.82115500	-1.76055100
	H	-0.17585300	2.52705800	-2.08275600
	H	-0.84128700	0.90565500	-2.33762700
	N	-1.78758800	3.43794200	0.29329300
	N	-0.71050900	1.48372000	-0.35670300
	H	-0.43508100	-0.14365100	0.92004000
	H	-0.28160100	-3.75892200	-1.20724400
	C	-2.40529400	-4.09020100	-0.89947300
	C	-2.58412900	-4.63798000	-2.18247500
	C	-3.50997700	-4.06661000	-0.02922700
	C	-3.82077100	-5.13772100	-2.58537900
	H	-1.74016200	-4.66129100	-2.86758900
	C	-4.74519300	-4.56630200	-0.43327800
	H	-3.40140000	-3.64095400	0.96110800
	C	-4.91107500	-5.10333000	-1.71293300
	H	-3.93359000	-5.55610100	-3.58155500
	H	-5.58586100	-4.53330500	0.25394700
	H	-5.87624200	-5.49047200	-2.02470200
	C	-2.58692100	-1.10110300	-1.06807800
	C	-2.97714100	-0.73240000	0.23024900
	C	-3.55710100	-1.01466800	-2.07966200
	C	-4.25317400	-0.24778300	0.48947100
	H	-2.29378700	-0.85862500	1.05758800
	C	-4.83522500	-0.52844300	-1.82355200
	H	-3.29849900	-1.33320700	-3.08490800

	C	-5.20551200	-0.11478400	-0.53411500
	H	-4.51120600	0.04894200	1.50140800
	H	-5.56117500	-0.48504200	-2.62920800
	C	-6.55089000	0.43875500	-0.25986600
	C	-7.20739600	0.17724900	0.95450600
	C	-7.20100200	1.24832600	-1.20642900
	C	-8.46960300	0.70820300	1.21364100
	H	-6.73468500	-0.46708400	1.68883900
	C	-8.46450900	1.77703100	-0.94874000
	H	-6.70021900	1.48225300	-2.14065200
	C	-9.10448300	1.51037400	0.26324800
	H	-8.96215300	0.48708900	2.15568400
	H	-8.94607100	2.40538000	-1.69191900
	H	-10.08812200	1.92283100	0.46481900
**Int-SR**	C	5.23402000	-4.43867200	1.88257100
	C	4.27190500	-3.49295700	2.15935900
	C	4.34636900	-2.19370900	1.59180600
	C	5.45421200	-1.87569200	0.73685400
	C	6.43015100	-2.87407500	0.47664300
	C	6.32249700	-4.12845500	1.03232800
	H	5.15939200	-5.42989400	2.31855500
	H	3.43952200	-3.73296500	2.81181900
	C	3.35917300	-1.19040800	1.84236000
	C	5.54861900	-0.58091200	0.16654900
	H	7.26332800	-2.62801100	-0.17519200
	H	7.07266300	-4.88410900	0.82201000
	C	4.55682000	0.35031400	0.37599800
	C	3.46439900	0.03468800	1.22211400
	H	6.39793300	-0.33369200	-0.46258300
	H	2.71510200	0.79503600	1.41108900
	C	2.24402800	-1.40990400	2.81013500
	C	1.06663300	-2.08501000	2.43259700
	C	2.34483000	-0.87861300	4.10793500
	C	0.01710500	-2.17438900	3.38013100
	C	0.21224500	-1.59518300	4.63883100
	H	-0.58898000	-1.65341300	5.37246400
	C	3.58990400	-0.16452500	4.57148400
	H	4.48992900	-0.74734100	4.35333900
	H	3.70983700	0.80255600	4.07140900
	H	3.52627800	0.00739600	5.64690600
	N	1.33735800	-0.97492800	4.99526000
	O	-1.15066100	-2.78209400	3.10321800
	H	-1.06044900	-3.10180000	2.14375700
	C	0.88449400	-2.61840800	1.08552900
	H	1.70649900	-2.51423400	0.37726400
	N	-0.23861000	-3.16407800	0.75168500
	C	-0.46175600	-3.39557500	-0.64664800
	C	4.63968600	1.72279300	-0.22615500
	O	5.69126100	2.37379300	-0.23707800
	N	3.45053400	2.17810700	-0.68749400
	H	2.72554600	1.48667400	-0.90555100
	C	3.25602200	3.54716700	-1.10414600
	H	4.09776400	4.12607100	-0.71667600
	C	3.23464300	3.70324300	-2.65461500
	H	4.09920100	3.11485000	-2.98569400
	C	1.97312000	3.09923600	-3.28229800
	H	2.05255100	3.11262600	-4.37400000
	H	1.81823600	2.06514500	-2.96229800
	H	1.08600600	3.68547800	-3.01348400
	C	3.44890600	5.15246100	-3.10255700

	H	4.35352100	5.57884600	-2.65682700
	H	3.55528400	5.19505600	-4.19116900
	H	2.60500900	5.78643300	-2.81897300
	C	1.97145400	4.05593900	-0.44914200
	O	1.09761000	3.32436000	-0.01807800
	O	1.94145100	5.39256900	-0.43751800
	C	0.77981600	6.14224600	0.09219700
	C	-0.47070900	5.79198800	-0.71298100
	C	0.61643800	5.84366900	1.58240000
	C	1.18898000	7.59511400	-0.13384600
	H	-0.29422700	5.95051600	-1.78073800
	H	-0.75961600	4.75587400	-0.54735400
	H	-1.29421700	6.44184000	-0.40212400
	H	1.54422300	6.06167200	2.11863400
	H	-0.17423800	6.47793500	1.99377400
	H	0.35269500	4.79900000	1.74513000
	H	0.40602900	8.26251500	0.23598100
	H	2.11771300	7.81980800	0.39711700
	H	1.33939600	7.79179500	-1.19876700
	C	-0.12518200	0.10999200	-0.05935900
	C	0.01991800	-0.89450800	-1.13586400
	C	1.19540600	-0.82809400	-1.89638800
	O	2.09052700	0.05405300	-1.83076400
	O	1.32377400	-1.88306700	-2.78726900
	C	2.60032000	-2.20807100	-3.40298700
	C	2.29318700	-3.50221900	-4.15995000
	H	1.96746100	-4.28333400	-3.46637100
	H	1.49860900	-3.33763100	-4.89390900
	H	3.18431200	-3.85617300	-4.68655500
	C	3.65856000	-2.45889900	-2.32520700
	H	4.59030300	-2.80406900	-2.78482400
	H	3.85852700	-1.54861100	-1.76336300
	H	3.31764300	-3.23362400	-1.63048300
	C	3.03147400	-1.11065600	-4.38213300
	H	3.27435500	-0.19460200	-3.84626100
	H	3.90562800	-1.44258900	-4.95222000
	H	2.22186700	-0.90224600	-5.08895200
	C	-0.95126600	-2.02451000	-1.34097000
	H	-0.92562400	-2.29638200	-2.39849400
	H	0.84386500	0.56395700	0.14353600
	C	-3.17148800	2.19198000	3.50892000
	C	-3.95412000	2.96035100	2.43695800
	C	-3.06552600	3.76997900	1.48244600
	C	-2.13927100	1.19571800	2.97111100
	C	-1.08635100	1.83630900	2.04228200
	C	-1.56545600	2.01908800	0.62381900
	H	-2.65290000	2.92000600	4.14716800
	H	-4.57191800	2.27565500	1.84237900
	H	-2.26238800	4.27487500	2.02706200
	H	-2.63889800	0.38063800	2.43976000
	H	-0.17609100	1.24545900	2.03819200
	H	-3.87748200	1.66085700	4.15643500
	H	-4.64065200	3.65490200	2.93313700
	H	-3.64713500	4.55422100	0.99633300
	H	-1.60962900	0.73894500	3.81268700
	H	-0.79798100	2.81924900	2.42666800
	C	-3.16316600	3.12910900	-0.91281400
	H	-2.85176000	4.09349600	-1.32900500
	H	-4.24020800	3.17927200	-0.72704800
	C	-2.84416800	1.98449500	-1.85905800

	H	-3.08067000	2.28105700	-2.88397100
	H	-3.45193900	1.11397500	-1.61245400
	C	-1.37623100	1.60999600	-1.73668100
	H	-0.72168900	2.43555800	-2.03808200
	H	-1.12340800	0.73388200	-2.33400000
	N	-2.49641800	2.95991600	0.39160000
	N	-1.07758200	1.27053400	-0.34806300
	H	-0.50678300	-0.33894900	0.85322800
	H	0.48510400	-3.60332200	-1.16026800
	C	-1.43714900	-4.50821500	-0.93202300
	C	-1.38010700	-5.13988200	-2.18098100
	C	-2.44668000	-4.88070700	-0.03733100
	C	-2.30926700	-6.11884100	-2.53186600
	H	-0.59921300	-4.85712800	-2.88250900
	C	-3.37477700	-5.86298800	-0.38354500
	H	-2.51134100	-4.39820000	0.93057200
	C	-3.31219400	-6.48474000	-1.63203900
	H	-2.24717900	-6.59855400	-3.50421800
	H	-4.15048300	-6.14131200	0.32382000
	H	-4.03595300	-7.24841100	-1.89992700
	C	-2.39067000	-1.67420800	-1.02238300
	C	-2.86450100	-1.44056000	0.27672900
	C	-3.30659700	-1.53428000	-2.07333100
	C	-4.17036500	-1.01908700	0.50513500
	H	-2.20971100	-1.58476300	1.12525800
	C	-4.61768100	-1.11894200	-1.85074800
	H	-2.98056900	-1.74327200	-3.08822800
	C	-5.06794100	-0.82160100	-0.55532900
	H	-4.49015200	-0.80793800	1.52082600
	H	-5.29821700	-1.02151700	-2.69076500
	C	-6.42449900	-0.27968500	-0.31265500
	C	-7.15446500	-0.63336900	0.83414300
	C	-7.00182200	0.62851200	-1.21604300
	C	-8.41919300	-0.09706100	1.06947600
	H	-6.73555600	-1.35025400	1.53317700
	C	-8.26769300	1.16299800	-0.98207300
	H	-6.44236100	0.93355100	-2.09487800
	C	-8.98187800	0.80328600	0.16246100
	H	-8.96964900	-0.38929400	1.95858000
	H	-8.69271900	1.86832600	-1.68980200
	H	-9.96701800	1.22051000	0.34602500
**TS-SN2**	C	-1.31707300	-0.13254900	-0.37438500
	C	-1.25784300	-1.33673600	0.37002800
	C	-2.34389500	-2.27549700	0.30667700
	O	-2.32830600	-3.42160500	0.76229100
	O	-3.45001400	-1.72452000	-0.30617600
	C	-4.64255600	-2.51967900	-0.61614500
	C	-5.54037500	-1.51735700	-1.34181500
	H	-5.78783200	-0.67571300	-0.68814700
	H	-5.04180300	-1.13047300	-2.23548500
	H	-6.47287000	-2.00007600	-1.64669400
	C	-5.30629200	-2.99580800	0.67723800
	H	-6.25922400	-3.48060300	0.44415000
	H	-4.66488900	-3.70104300	1.20400600
	H	-5.50927300	-2.14210800	1.33129700
	C	-4.27048100	-3.67925300	-1.54179900
	H	-3.61950600	-4.38925700	-1.03255100
	H	-5.17826200	-4.19759400	-1.86454500
	H	-3.75823600	-3.30083200	-2.43183800
	C	-0.11520200	-1.68698700	1.26128200

	H	-0.44598600	-2.40020600	2.01925100
	C	-0.08394200	0.69771800	-0.50565200
	C	0.39217500	1.51967500	0.52170500
	C	0.68090900	0.58204800	-1.67347200
	C	1.62002100	2.16429200	0.40680600
	H	-0.18098900	1.64045700	1.43510600
	C	1.92014400	1.20575600	-1.77933400
	H	0.30860100	-0.01972500	-2.49716200
	C	2.42550400	1.98988900	-0.72950000
	H	1.98545000	2.76082700	1.23564900
	H	2.49852800	1.09243300	-2.69014500
	H	0.27319900	-0.79938300	1.75038400
	H	-1.89435300	-0.19029400	-1.29526500
	C	3.79040500	2.56093100	-0.78356900
	C	4.08414300	3.80531200	-0.20372000
	C	4.83572400	1.83861500	-1.38299700
	C	5.38343400	4.30900200	-0.21880600
	H	3.28642000	4.38931500	0.24397000
	C	6.13548500	2.34109600	-1.39579500
	H	4.63063500	0.86705200	-1.82077000
	C	6.41477900	3.57827000	-0.81224600
	H	5.58970500	5.27632200	0.22853700
	H	6.93119700	1.76242200	-1.85441900
	H	7.42692600	3.96989600	-0.82029000
	C	4.97560700	-0.50011200	1.92934900
	C	5.37533000	-1.40071000	0.75759400
	C	4.71682800	-2.78331800	0.79841000
	C	3.46825900	-0.27868400	2.08044800
	C	2.64533900	-1.57307700	2.27947500
	C	2.31249100	-2.26087500	0.97955400
	H	5.36408200	-0.93952300	2.85757300
	H	5.12957900	-0.92042600	-0.19688600
	H	4.73605900	-3.19765700	1.81026400
	H	3.07843800	0.25892500	1.21371400
	H	1.73114200	-1.35700300	2.82305900
	H	5.46392000	0.47335600	1.81469400
	H	6.46132700	-1.54143700	0.76933700
	H	5.26408700	-3.48415200	0.16700200
	H	3.29096200	0.36375900	2.94826800
	H	3.19857500	-2.28073200	2.90555000
	C	3.18972500	-3.33742800	-1.07363000
	H	3.29220200	-4.42661300	-1.01543600
	H	4.02355700	-2.95483900	-1.66825300
	C	1.86172300	-2.93986200	-1.68624600
	H	1.66633900	-3.54829100	-2.57196500
	H	1.88643600	-1.89047100	-1.99200500
	C	0.76196700	-3.13348800	-0.65637200
	H	0.66873400	-4.18509500	-0.36342300
	H	-0.19892700	-2.79611100	-1.03618900
	N	3.33145600	-2.78771800	0.28687400
	N	1.05970800	-2.33022100	0.53776700
	C	-5.44718000	2.38725900	-0.61767100
	C	-6.69820300	2.85288700	-0.21312200
	C	-6.79787300	3.75311700	0.84958000
	C	-5.64301300	4.18682100	1.50409100
	C	-4.39310800	3.71813800	1.09983300
	C	-4.28492300	2.81337400	0.03733600
	H	-5.36917600	1.69303100	-1.44960600
	H	-7.59207700	2.51869100	-0.73026000
	H	-7.77040100	4.11914900	1.16301500

	H	-5.71570400	4.89200800	2.32607100
	H	-3.49459000	4.05692700	1.60857500
	C	-2.94286900	2.25098000	-0.35980100
	H	-2.13580800	2.96369000	-0.18133700
	H	-2.93079400	1.98968000	-1.42216500
	N	-2.61642500	1.01289100	0.38570000
	H	-2.40093900	1.19512700	1.36454500
	H	-3.39204300	0.34836800	0.35953500
**Int-SN2**	C	-1.37270700	-0.01740700	-0.34692200
	C	-1.31484100	-1.24071400	0.50157100
	C	-2.39804600	-2.14627000	0.50165400
	O	-2.44088900	-3.26815900	1.02558100
	O	-3.51307800	-1.60234000	-0.16599100
	C	-4.70782300	-2.39674100	-0.44658700
	C	-5.59103700	-1.43085700	-1.23761300
	H	-5.85551500	-0.56113800	-0.62863100
	H	-5.07277100	-1.08411500	-2.13676100
	H	-6.51631900	-1.92816200	-1.54123400
	C	-5.39613200	-2.79990900	0.85943800
	H	-6.34976100	-3.28999400	0.63929000
	H	-4.76412600	-3.47775900	1.43099100
	H	-5.60161200	-1.91098100	1.46427000
	C	-4.33507800	-3.60763400	-1.30478200
	H	-3.68600400	-4.28498800	-0.75082700
	H	-5.24109900	-4.14359200	-1.60339600
	H	-3.81691000	-3.27974000	-2.21154300
	C	-0.12066400	-1.60972200	1.28637200
	H	-0.41017500	-2.27578300	2.10164800
	C	-0.08872200	0.76744000	-0.43118300
	C	0.34499200	1.61174300	0.59790800
	C	0.73984100	0.59496800	-1.54397700
	C	1.58228500	2.24437500	0.52554900
	H	-0.26528600	1.76320300	1.48399800
	C	1.98561600	1.21155000	-1.61048300
	H	0.40399600	-0.03051200	-2.36574400
	C	2.43661800	2.03903400	-0.56967400
	H	1.91188000	2.86335700	1.35282700
	H	2.60818000	1.06376500	-2.48652100
	H	0.37582700	-0.73331600	1.69054900
	H	-1.72235600	-0.23557100	-1.36270800
	C	3.79355000	2.63133200	-0.59288800
	C	4.04016400	3.90517200	-0.05703000
	C	4.87405200	1.90794700	-1.12421800
	C	5.32821900	4.43724800	-0.04962500
	H	3.21486900	4.48924800	0.33747200
	C	6.16254900	2.43862400	-1.11418300
	H	4.70571400	0.91412600	-1.52553600
	C	6.39479700	3.70571600	-0.57576900
	H	5.49816300	5.42702900	0.36239100
	H	6.98581000	1.85913000	-1.52005700
	H	7.39800500	4.11964800	-0.56665400
	C	5.06939900	-0.76738800	1.54566600
	C	5.33469200	-1.74583900	0.39771900
	C	4.59360600	-3.07924400	0.54275600
	C	3.59207900	-0.44288200	1.78441700
	C	2.71149100	-1.67396500	2.10205300
	C	2.24596100	-2.38536300	0.85623400
	H	5.49173400	-1.18817900	2.46778800
	H	5.05775400	-1.29746600	-0.56430400
	H	4.65579300	-3.45310100	1.56845100

	H	3.17469200	0.07749200	0.91955300
	H	1.85277400	-1.37851500	2.69624800
	H	5.60720400	0.16626800	1.35056800
	H	6.40841200	-1.95630100	0.34938500
	H	5.05206200	-3.83954300	-0.09069400
	H	3.51491700	0.25059000	2.62714200
	H	3.26589600	-2.38797300	2.71928200
	C	2.90696000	-3.58907100	-1.20498600
	H	2.90780600	-4.68111300	-1.11413300
	H	3.73500700	-3.30846000	-1.86140700
	C	1.58695100	-3.08337200	-1.75427800
	H	1.28839700	-3.68707800	-2.61412200
	H	1.69209300	-2.04673900	-2.08694500
	C	0.53323700	-3.15516500	-0.66233400
	H	0.35376200	-4.18928200	-0.34752300
	H	-0.41478200	-2.73165800	-0.98870700
	N	3.18041800	-3.01010600	0.12250600
	N	0.96881500	-2.36972900	0.49706300
	C	-5.35357500	2.41423600	-0.71048000
	C	-6.52575400	2.98212400	-0.21225000
	C	-6.46353000	3.91296600	0.82638200
	C	-5.22684600	4.27874700	1.36193700
	C	-4.05489100	3.71074700	0.86372700
	C	-4.11093300	2.77109700	-0.17284400
	H	-5.40084800	1.69722600	-1.52460700
	H	-7.48408100	2.70226200	-0.63748700
	H	-7.37515000	4.35692900	1.21311300
	H	-5.17485500	5.00876900	2.16315500
	H	-3.09287300	4.00126000	1.27746700
	C	-2.85429500	2.11091000	-0.66849200
	H	-1.98825900	2.77094800	-0.61535300
	H	-2.95904300	1.75620800	-1.69533400
	N	-2.53420700	0.89899400	0.16140300
	H	-2.36291200	1.15497400	1.13618300
	H	-3.33255800	0.24191500	0.16819900

11. Determination of the Absolute Configurations of Compounds 3m, 9a and 11h' by X-ray Analysis

11.1 Determination of the Absolute Configuration of Compound 3m (Fig. 3a)

The single crystal of compound **3m** was obtained by recrystallization from a mixed solvent of petroleum ether and ethyl acetate.

The absolute configuration of compound **3m** obtained by (*S*,*S*)-**6a**-catalyzed asymmetric alkylation was determined as (3*S*,4*R*) by X-ray analysis (Figure S8).

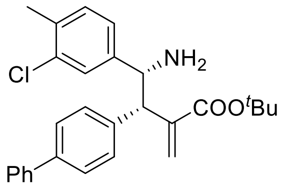

(3*S*,4*R*)-**3m**

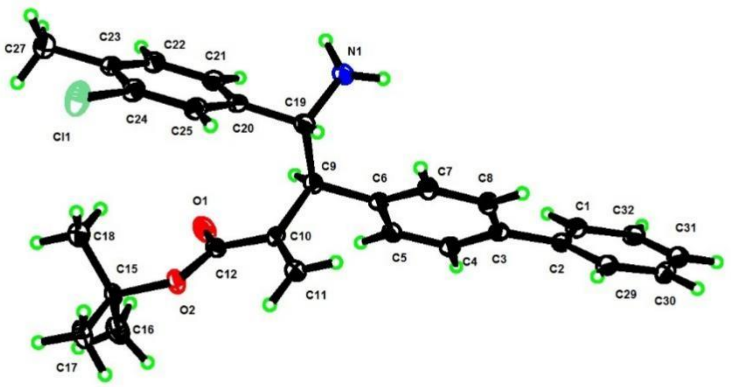

Figure S8. X-ray structure of compound (3*S*,4*R*)-3m

Table S12. Crystal data and structure refinement for 3m (CCDC 2406669)

Identification code	cu_20240938_0m
Empirical formula	$C_{28}H_{30}ClNO_2$
Formula weight	447.98
Temperature/K	150
Crystal system	orthorhombic
Space group	$P2_12_12_1$
a/Å	5.91260(10)
b/Å	19.5253(5)
c/Å	20.5726(5)
α/°	90
β/°	90
γ/°	90
Volume/Å^3	2375.01(9)
Z	4
ρ_{calc}g/cm^3	1.253
μ/mm^{-1}	1.610
F(000)	952.0
Crystal size/mm^3	0.11 × 0.04 × 0.02
Radiation	CuKα (λ = 1.54178)
2Θ range for data collection/°	6.24 to 127.318
Index ranges	$-6 \le h \le 5$, $-18 \le k \le 21$, $-23 \le l \le 23$
Reflections collected	13021
Independent reflections	3692 [R_{int} = 0.0766, R_{sigma} = 0.0669]
Data/restraints/parameters	3692/0/293
Goodness-of-fit on F^2	1.047
Final R indexes [I>=2σ (I)]	R_1 = 0.0492, wR_2 = 0.1053
Final R indexes [all data]	R_1 = 0.0627, wR_2 = 0.1146
Largest diff. peak/hole / e Å^{-3}	0.26/-0.23
Flack parameter	0.074(17)

11.2 Determination of the Absolute Configuration of Compound 9a (Fig. 4b)

The single crystal of **9a** was obtained by recrystallization from a mixed solvent of hexane and DCM.

The absolute configuration of compound **9a** obtained by (*S*,*S*)-**6a**-catalyzed asymmetric alkylation followed by the cyclization was determined as (4*S*,5*R*) by X-ray analysis (Figure S9).

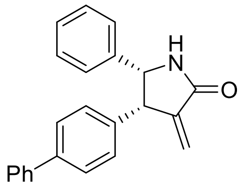

(4*S*,5*R*)-**9a**

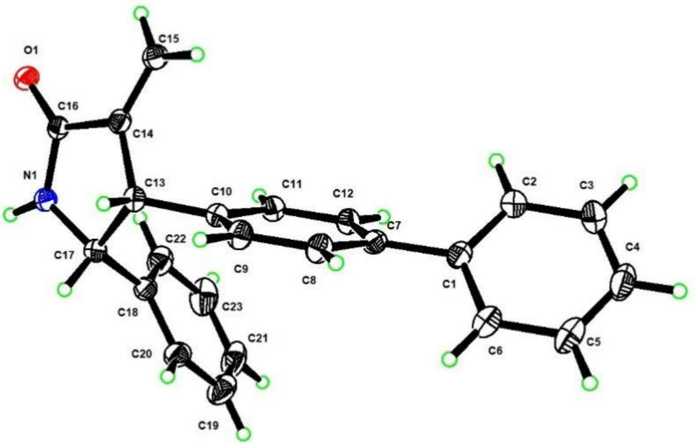

Figure S9. X-ray structure of compound (4*S*,5*R*)-9a

Table S13. Crystal data and structure refinement for 9a (CCDC 2406666)

Identification code	cu_20240920_0m
Empirical formula	$C_{23}H_{19}NO$
Formula weight	325.39
Temperature/K	170
Crystal system	orthorhombic
Space group	$P2_12_12_1$
a/Å	6.5747(9)
b/Å	7.7431(8)
c/Å	34.891(3)
α/°	90
β/°	90
γ/°	90
Volume/Å^3	1776.3(3)
Z	4
ρcalcg/cm^3	1.217
μ/mm^{-1}	0.576
F(000)	688.0
Crystal size/mm^3	0.12 × 0.08 × 0.04
Radiation	CuKα (λ = 1.54178)
2Θ range for data collection/°	5.066 to 153.546
Index ranges	$-8 \leq h \leq 8$, $-9 \leq k \leq 9$, $-43 \leq l \leq 44$
Reflections collected	37776
Independent reflections	3722 [R_{int} = 0.0836, R_{sigma} = 0.0406]
Data/restraints/parameters	3722/0/226
Goodness-of-fit on F^2	1.061
Final R indexes [I>=2σ (I)]	R_1 = 0.0378, wR_2 = 0.0968
Final R indexes [all data]	R_1 = 0.0409, wR_2 = 0.0992
Largest diff. peak/hole / e Å^{-3}	0.26/-0.26
Flack parameter	-0.09(13)

11.3 Determination of the Absolute Configuration of Compound 11h' (Fig. 4b)

The single crystal of **11h'** was obtained by recrystallization from a mixed solvent of hexane and DCM.

The absolute configuration of compound **11h'** obtained by (*S*,*S*)-**6a**-catalyzed asymmetric alkylation followed by the reduction and cyclization was determined as (3*S*,4*R*,5*R*) by X-ray analysis (Figure S10).

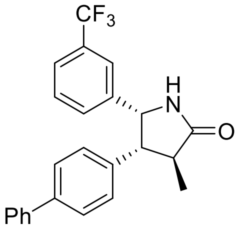

(3*S*,4*R*,5*R*)-**11h'**

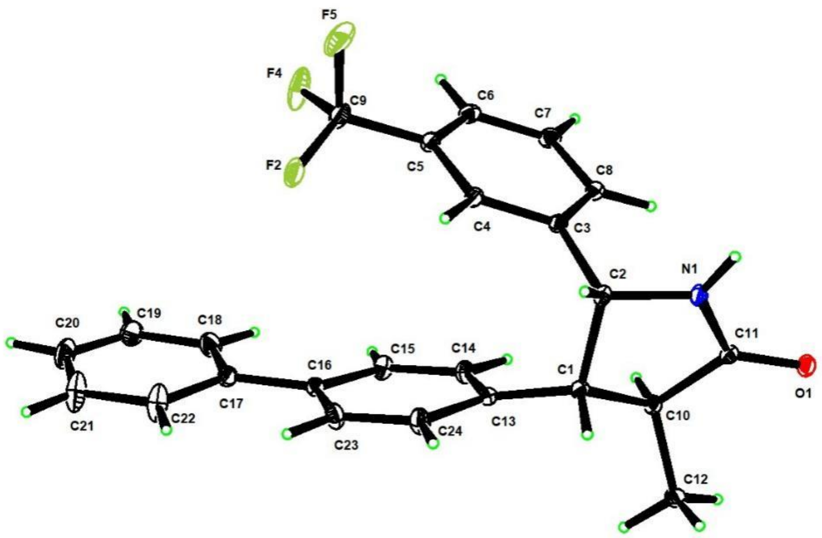

Figure S10. X-ray structure of compound (3*S*,4*R*,5*R*)-11h’

Table S14. Crystal data and structure refinement for (3*S*,4*R*,5*R*)-11h' (CCDC 2406667)

Identification code	cu_20241481_0m
Empirical formula	$C_{24}H_{20}F_3NO$
Formula weight	395.41
Temperature/K	173.00
Crystal system	monoclinic
Space group	$P2_1$
a/Å	8.9174(3)
b/Å	8.0476(2)
c/Å	13.9359(3)
α/°	90
β/°	92.984(2)
γ/°	90
Volume/Å^3	998.74(5)
Z	2
ρcalcg/cm^3	1.315
μ/mm^{-1}	0.829
F(000)	412.0
Crystal size/mm^3	0.12 × 0.05 × 0.04
Radiation	CuKα (λ = 1.54178)
2Θ range for data collection/°	6.35 to 149.288
Index ranges	-11 ≤ h ≤ 10, -10 ≤ k ≤ 10, -17 ≤ l ≤ 17
Reflections collected	23295
Independent reflections	4023 [R_{int} = 0.0646, R_{sigma} = 0.0479]
Data/restraints/parameters	4023/37/291
Goodness-of-fit on F^2	1.068
Final R indexes [I>=2σ (I)]	R_1 = 0.0506, wR_2 = 0.1275
Final R indexes [all data]	R_1 = 0.0524, wR_2 = 0.1296
Largest diff. peak/hole / e Å^{-3}	0.35/-0.29
Flack parameter	0.00(10)

12. ^{1}H NMR, ^{13}C NMR and ^{19}F NMR Spectra

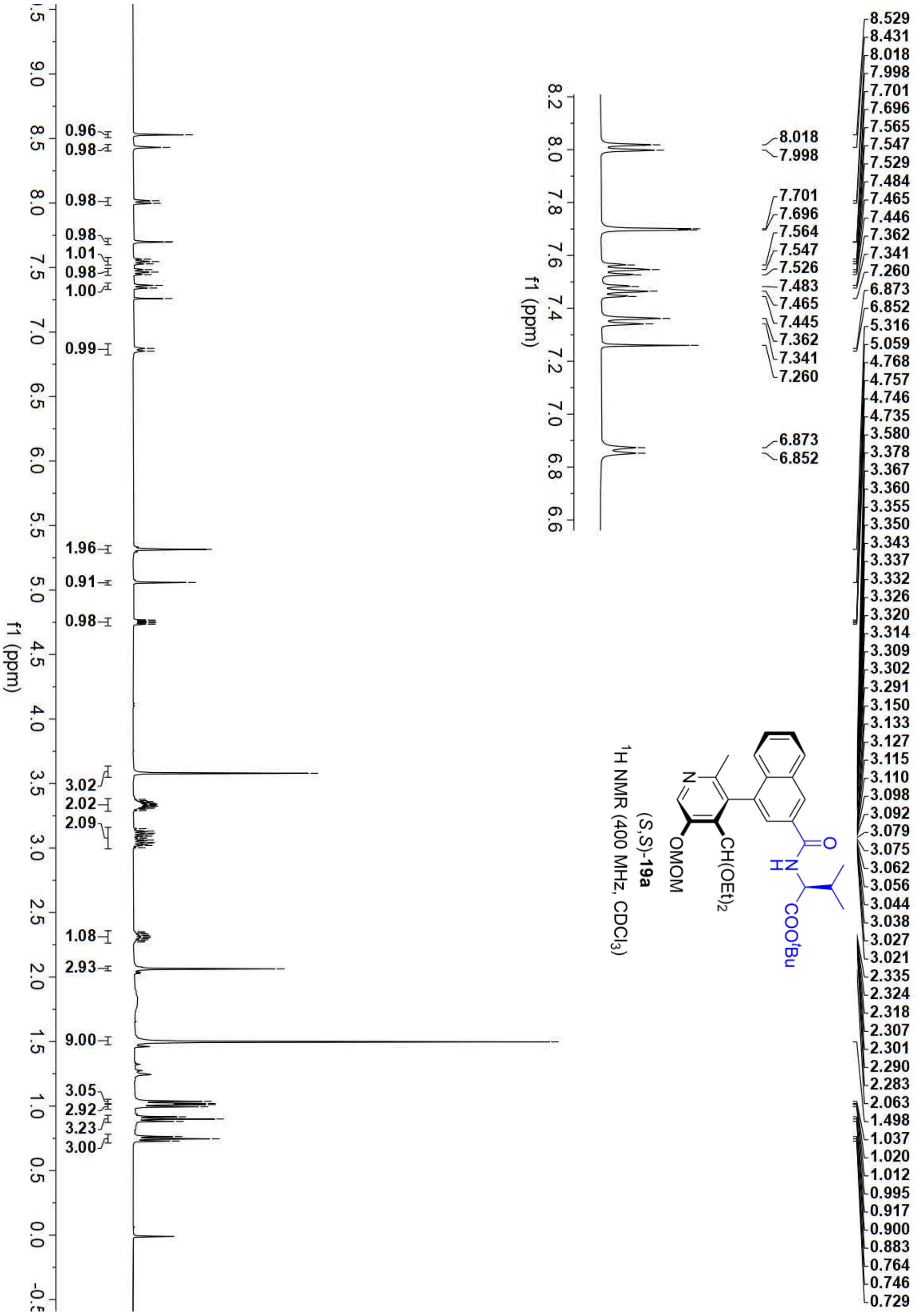

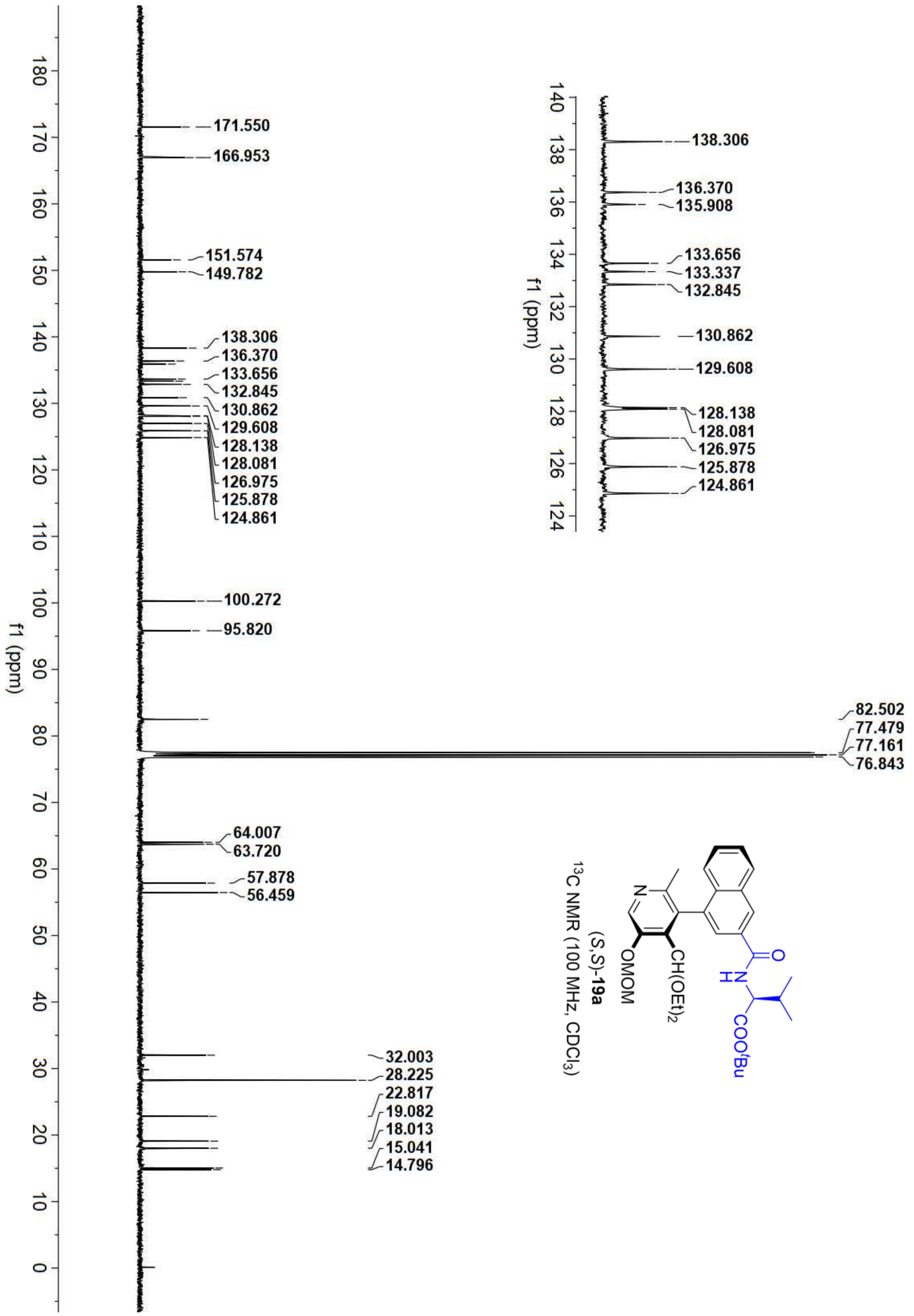

171.550
166.953
151.574
149.782
138.306
136.370
135.908
133.656
133.337
132.845
130.862
129.608
128.138
128.081
126.975
125.878
124.861
100.272
95.820
82.502
77.479
77.161
76.843
64.007
63.720
57.878
56.459
32.003
28.225
22.817
19.082
18.013
15.041
14.796
f1 (ppm)
CH(OEt)2
OMOM
COO^{t}Bu
(S,S)-19a
^{13}C NMR (100 MHz, $CDCl_3$)

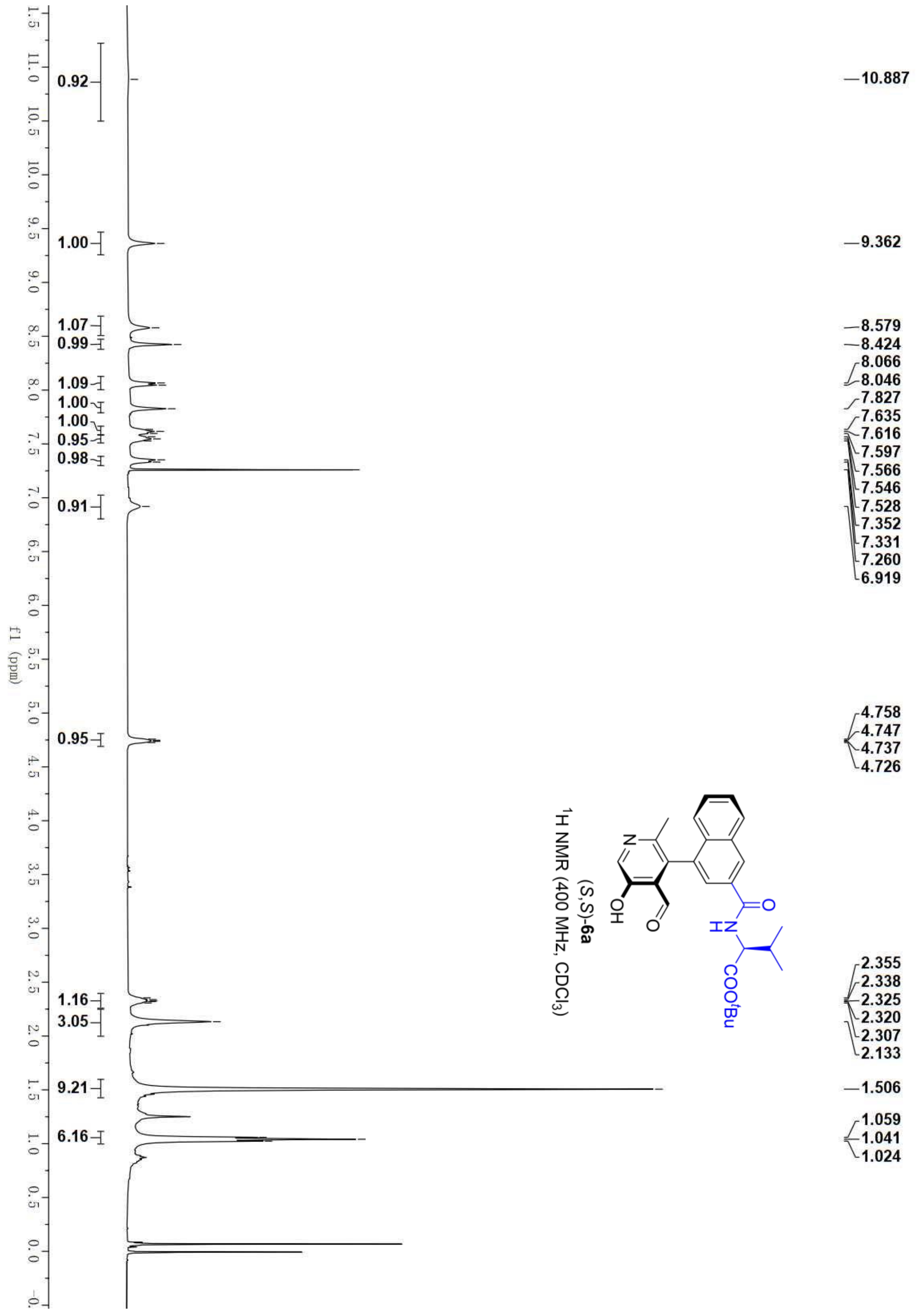
(S,S)-6a
1H NMR (400 MHz, CDCl3)
f1 (ppm)
10.887
9.362
8.579
8.424
8.066
8.046
7.827
7.635
7.616
7.597
7.566
7.546
7.528
7.352
7.331
7.260
6.919
4.758
4.747
4.737
4.726
2.355
2.338
2.325
2.320
2.307
2.133
1.506
1.059
1.041
1.024
0.92
1.00
1.07
0.99
1.09
1.00
1.00
0.95
0.98
0.91
0.95
1.16
3.05
9.21
6.16
N
OH
O
H
COOtBu

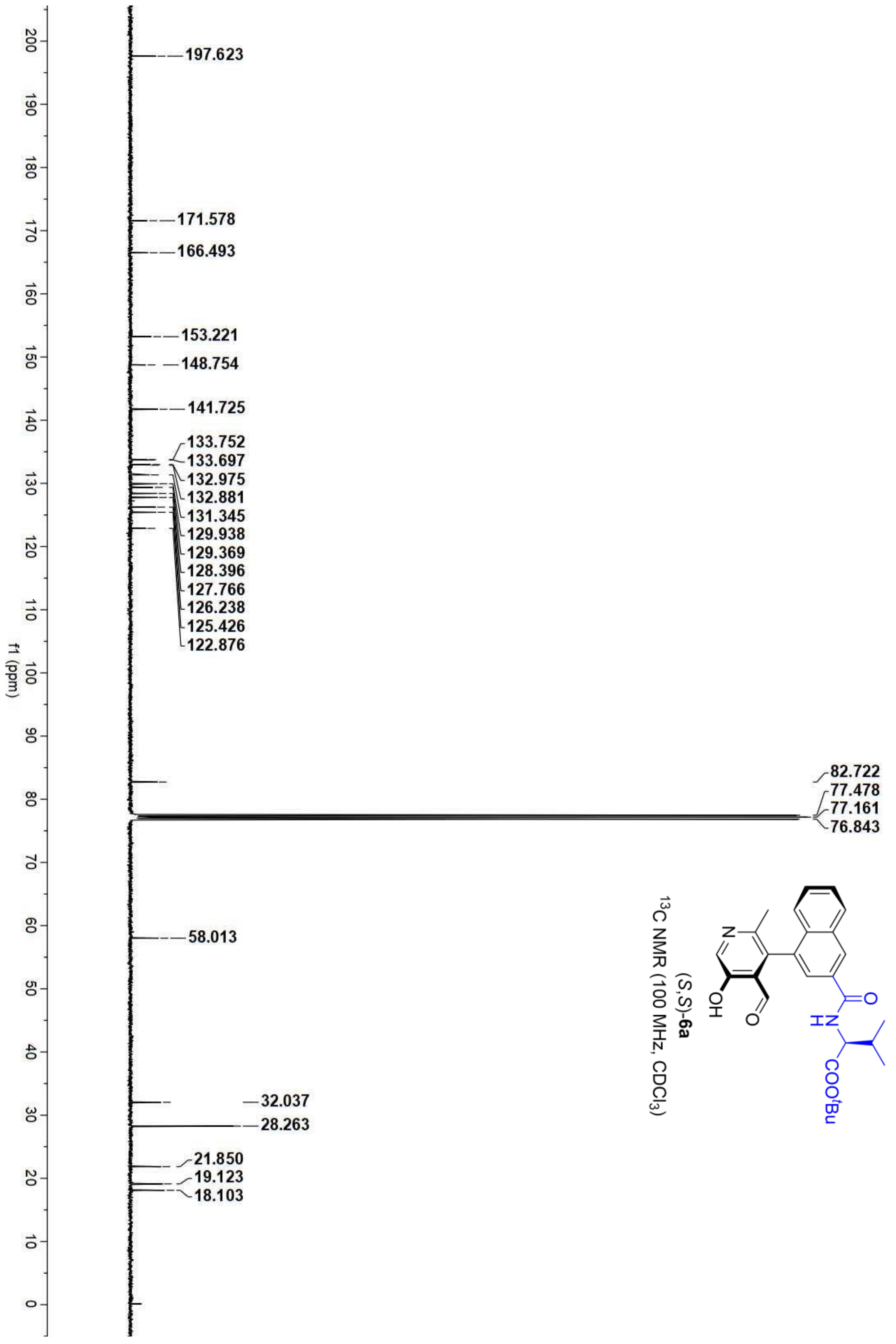
197.623
171.578
166.493
153.221
148.754
141.725
133.752
133.697
132.975
132.881
131.345
129.938
129.369
128.396
127.766
126.238
125.426
122.876
82.722
77.478
77.161
76.843
58.013
32.037
28.263
21.850
19.123
18.103
f1 (ppm)
(S,S)-6a
13C NMR (100 MHz, CDCl3)

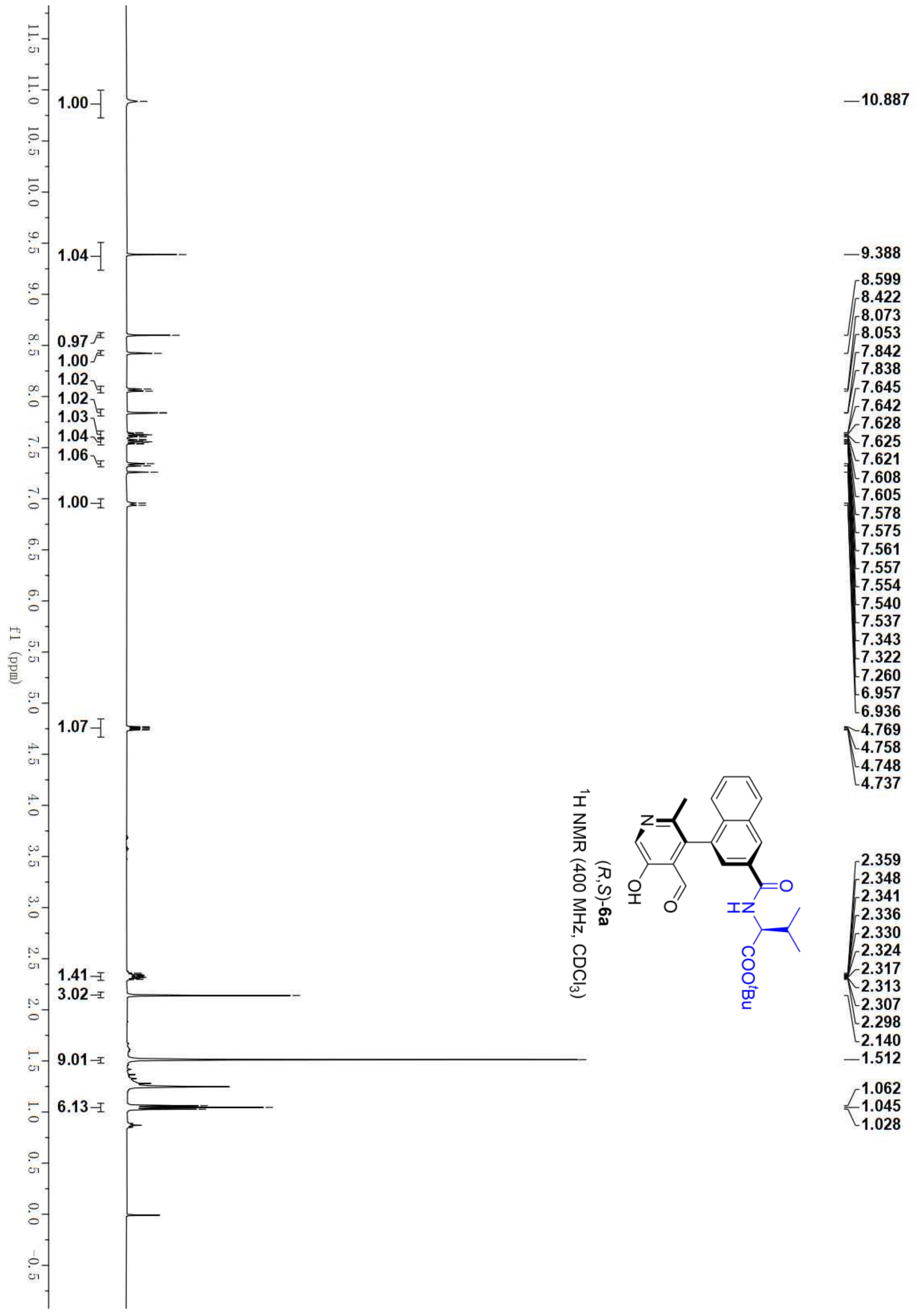
10.887
9.388
8.599
8.422
8.073
8.053
7.842
7.838
7.645
7.642
7.628
7.625
7.621
7.608
7.605
7.578
7.575
7.561
7.557
7.554
7.540
7.537
7.343
7.322
7.260
6.957
6.936
4.769
4.758
4.748
4.737
2.359
2.348
2.341
2.336
2.330
2.324
2.317
2.313
2.307
2.298
2.140
1.512
1.062
1.045
1.028
1.00
1.04
0.97
1.00
1.02
1.02
1.03
1.04
1.06
1.00
1.07
1.41
3.02
9.01
6.13
f1 (ppm)
(R,S)-6a
1H NMR (400 MHz, CDCl3)

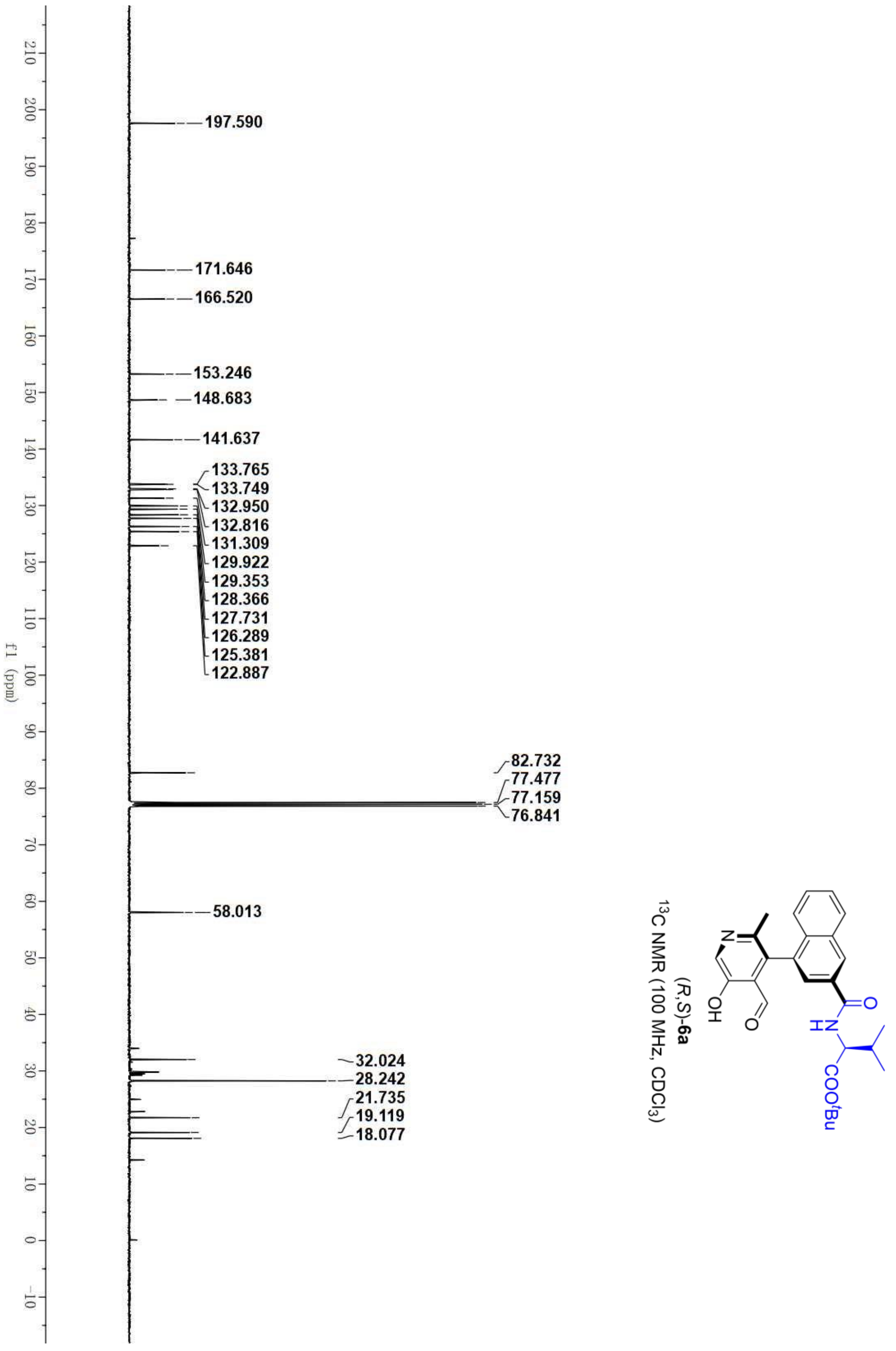

(*R,S*)-**6a**
^{13}C NMR (100 MHz, $CDCl_3$)

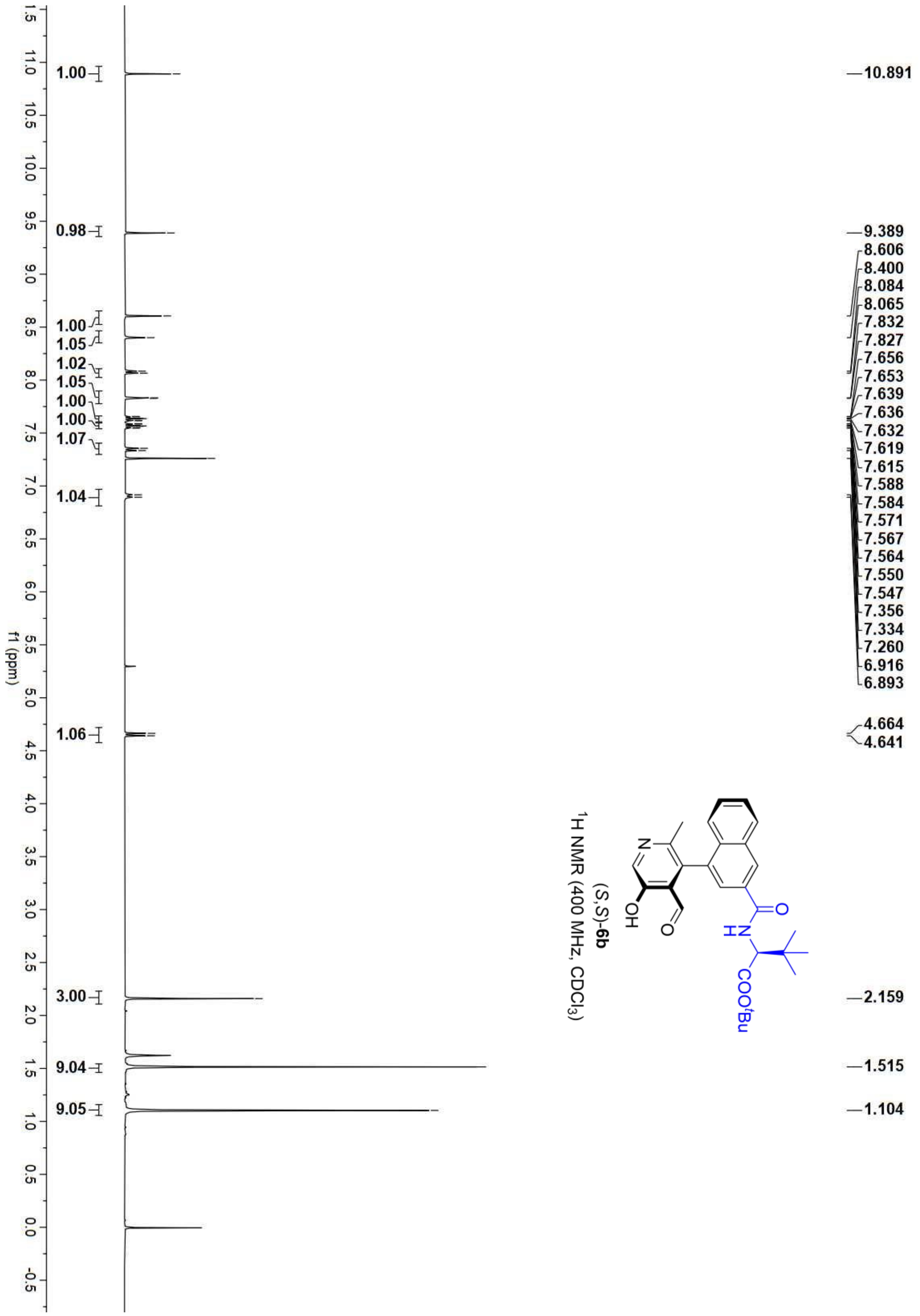
10.891
9.389
8.606
8.400
8.084
8.065
7.832
7.827
7.656
7.653
7.639
7.636
7.632
7.619
7.615
7.588
7.584
7.571
7.567
7.564
7.550
7.547
7.356
7.334
7.260
6.916
6.893
4.664
4.641
2.159
1.515
1.104
(S,S)-6b
1H NMR (400 MHz, CDCl3)
f1 (ppm)

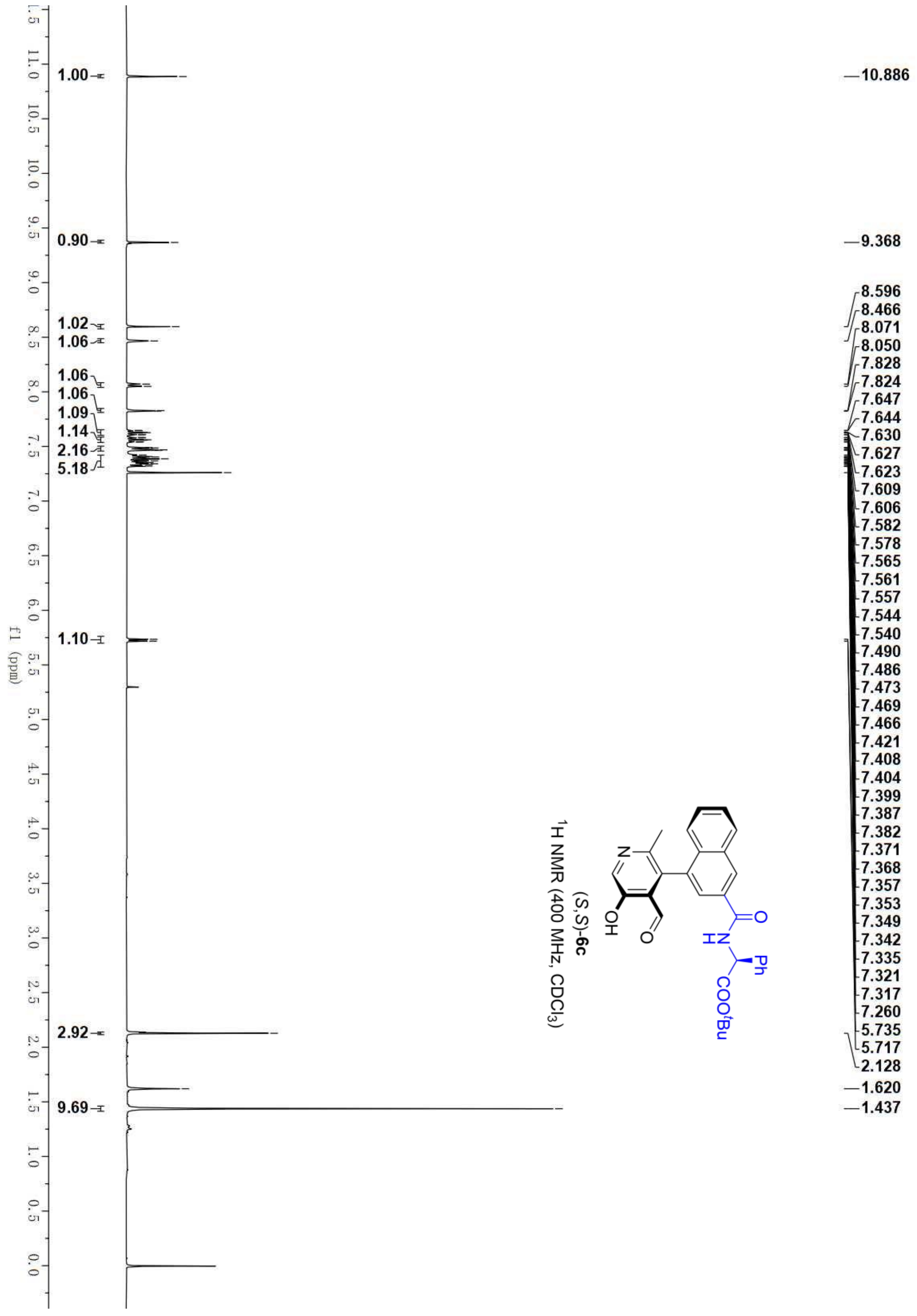
(S,S)-6c
1H NMR (400 MHz, CDCl3)
f1 (ppm)
10.886
9.368
8.596
8.466
8.071
8.050
7.828
7.824
7.647
7.644
7.630
7.627
7.623
7.609
7.606
7.582
7.578
7.565
7.561
7.557
7.544
7.540
7.490
7.486
7.473
7.469
7.466
7.421
7.408
7.404
7.399
7.387
7.382
7.371
7.368
7.357
7.353
7.349
7.342
7.335
7.321
7.317
7.260
5.735
5.717
2.128
1.620
1.437
1.00
0.90
1.02
1.06
1.06
1.06
1.09
1.14
2.16
5.18
1.10
2.92
9.69

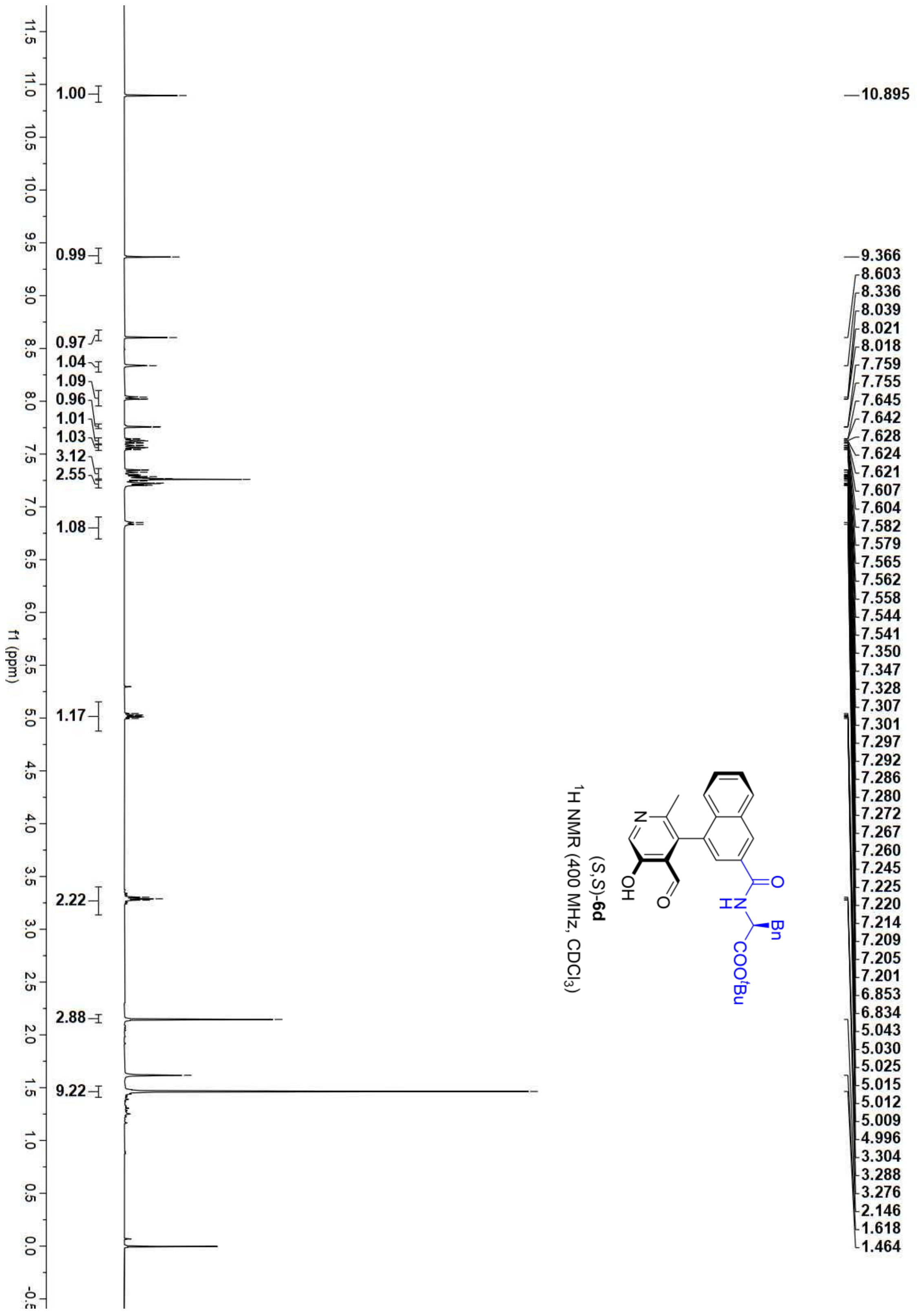
(S,S)-6d
1H NMR (400 MHz, CDCl3)
f1 (ppm)
1.00
0.99
0.97
1.04
1.09
0.96
1.01
1.03
3.12
2.55
1.08
1.17
2.22
2.88
9.22
10.895
9.366
8.603
8.336
8.039
8.021
8.018
7.759
7.755
7.645
7.642
7.628
7.624
7.621
7.607
7.604
7.582
7.579
7.565
7.562
7.558
7.544
7.541
7.350
7.347
7.328
7.307
7.301
7.297
7.292
7.286
7.280
7.272
7.267
7.260
7.245
7.225
7.220
7.214
7.209
7.205
7.201
6.853
6.834
5.043
5.030
5.025
5.015
5.012
5.009
4.996
3.304
3.288
3.276
2.146
1.618
1.464

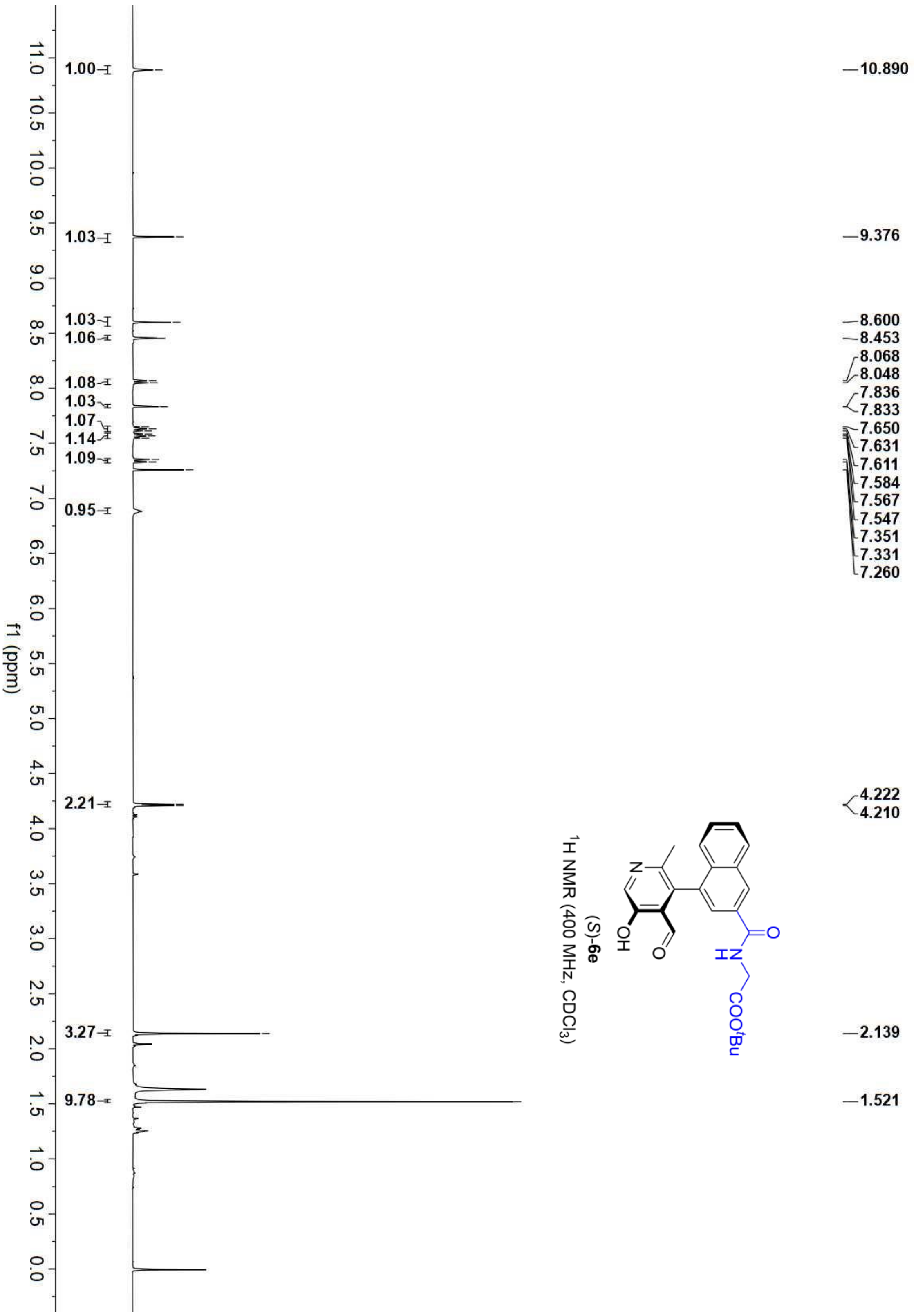
(S)-6e
¹H NMR (400 MHz, CDCl₃)
COOᵗBu
f1 (ppm)
10.890
9.376
8.600
8.453
8.068
8.048
7.836
7.833
7.650
7.631
7.611
7.584
7.567
7.547
7.351
7.331
7.260
4.222
4.210
2.139
1.521
1.00
1.03
1.03
1.06
1.08
1.03
1.07
1.14
1.09
0.95
2.21
3.27
9.78

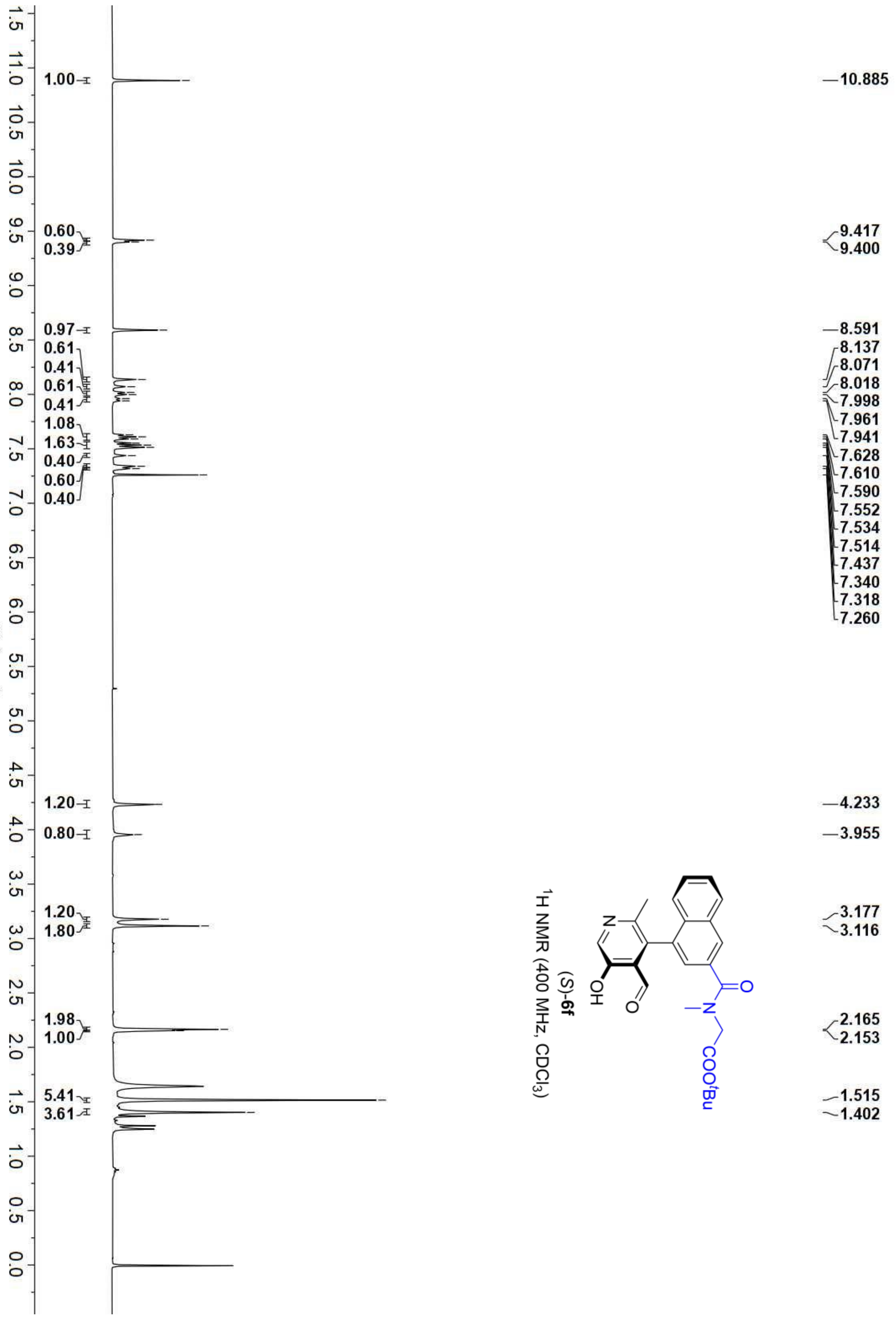

(S)-**6f**

^{1}H NMR (400 MHz, $CDCl_3$)

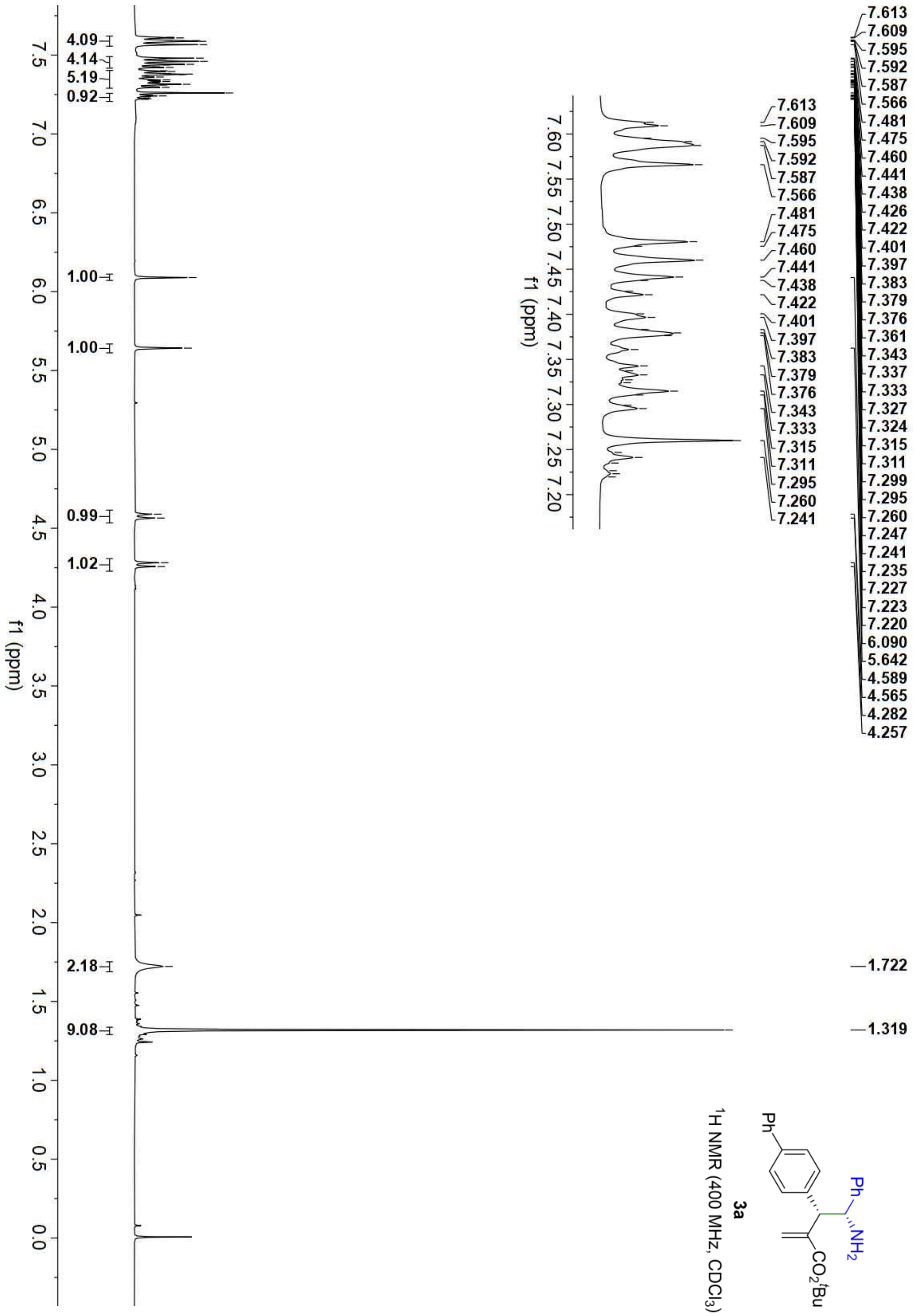
7.613
7.609
7.595
7.592
7.587
7.566
7.481
7.475
7.460
7.441
7.438
7.426
7.422
7.401
7.397
7.383
7.379
7.376
7.361
7.343
7.337
7.333
7.327
7.324
7.315
7.311
7.299
7.295
7.260
7.247
7.241
7.235
7.227
7.223
7.220
6.090
5.642
4.589
4.565
4.282
4.257
1.722
1.319
4.09
4.14
5.19
0.92
1.00
1.00
0.99
1.02
2.18
9.08
f1 (ppm)
Ph
NH2
CO2tBu
3a
1H NMR (400 MHz, CDCl3)

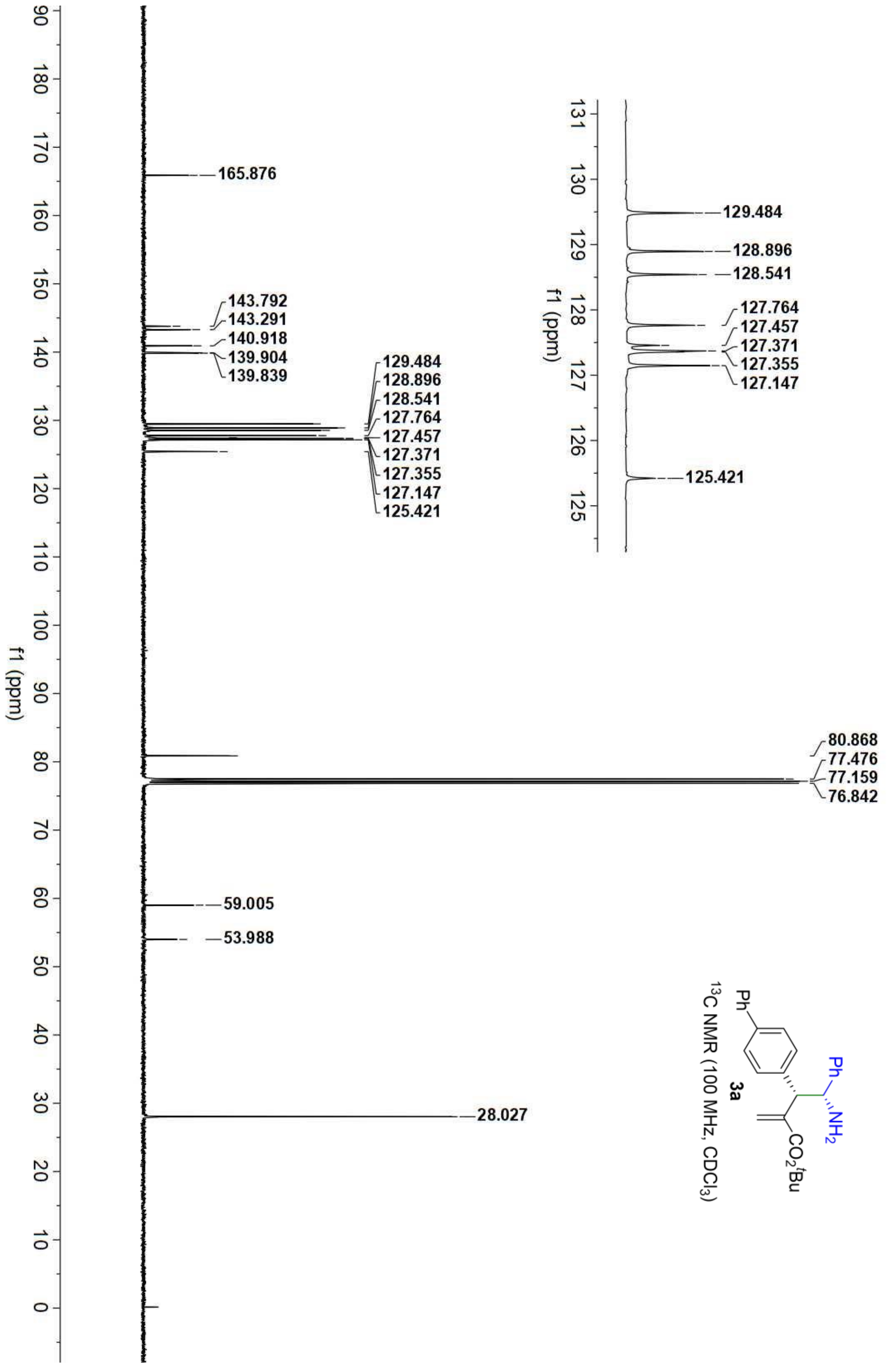

165.876
143.792
143.291
140.918
139.904
139.839
129.484
128.896
128.541
127.764
127.457
127.371
127.355
127.147
125.421
80.868
77.476
77.159
76.842
59.005
53.988
28.027
f1 (ppm)
Ph
NH2
CO2tBu
3a
13C NMR (100 MHz, CDCl3)

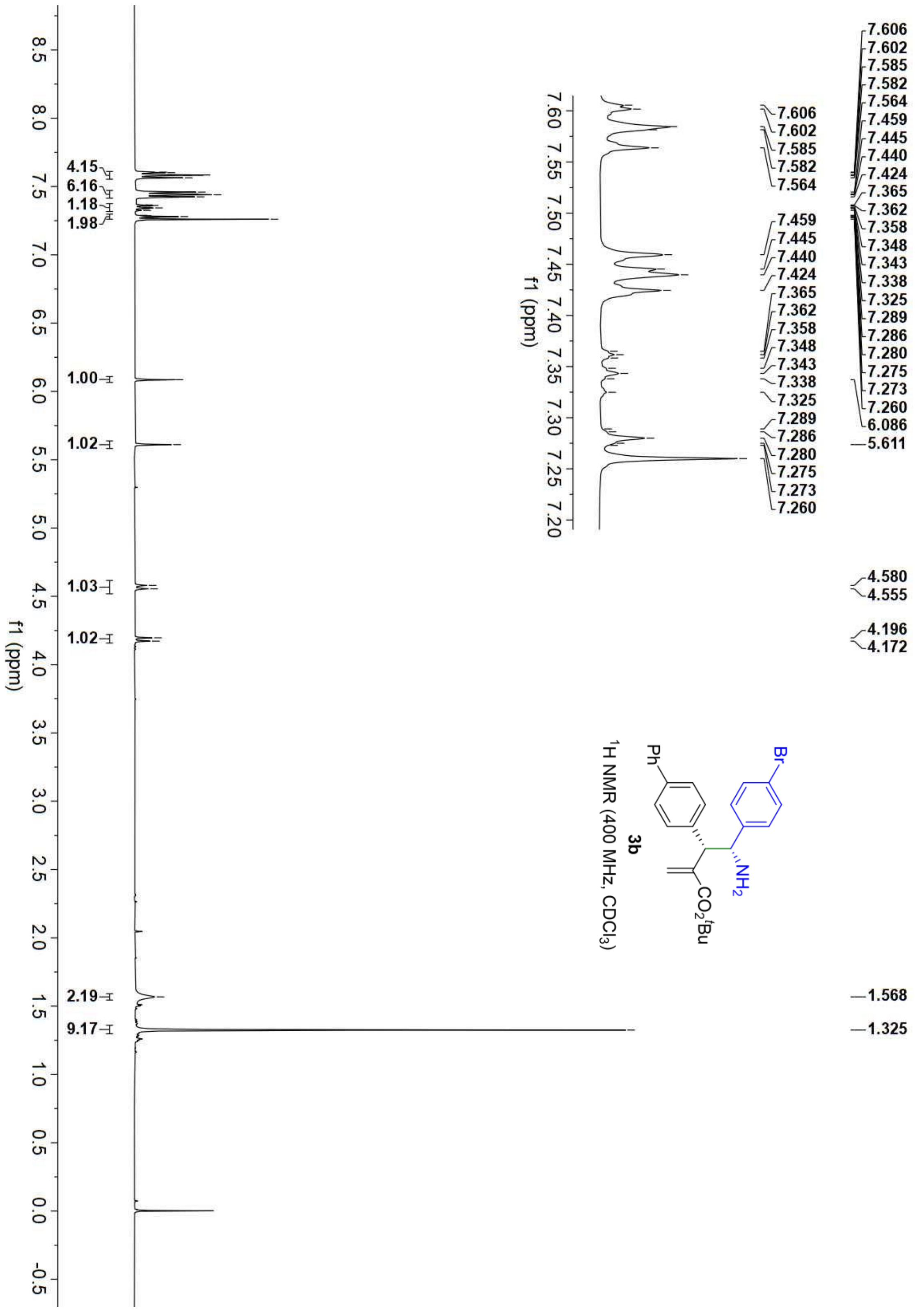

3b
1H NMR (400 MHz, CDCl3)
f1 (ppm)

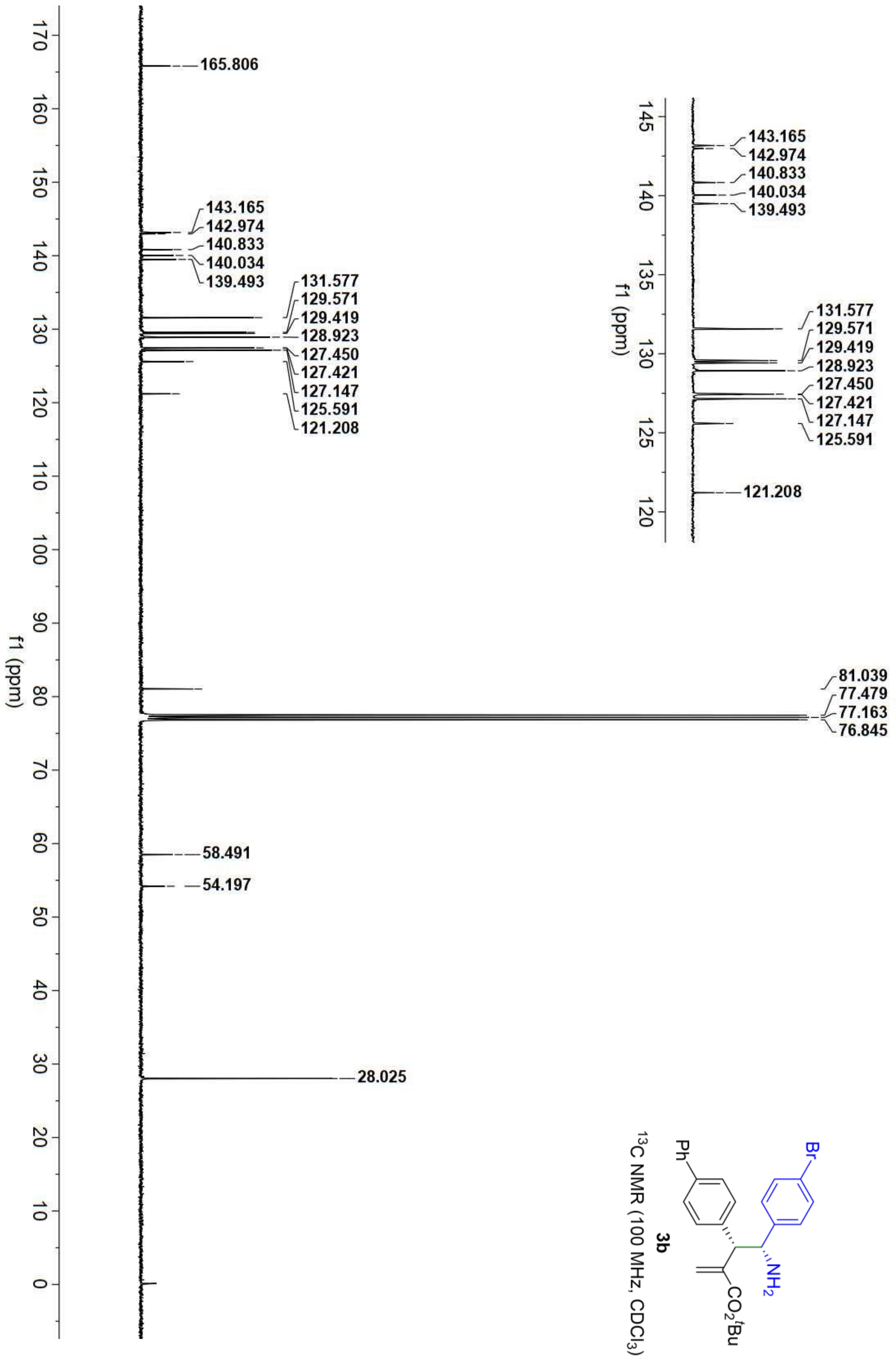
165.806
143.165
142.974
140.833
140.034
139.493
131.577
129.571
129.419
128.923
127.450
127.421
127.147
125.591
121.208
81.039
77.479
77.163
76.845
58.491
54.197
28.025
f1 (ppm)
Br
Ph
NH2
CO2tBu
3b
13C NMR (100 MHz, CDCl3)

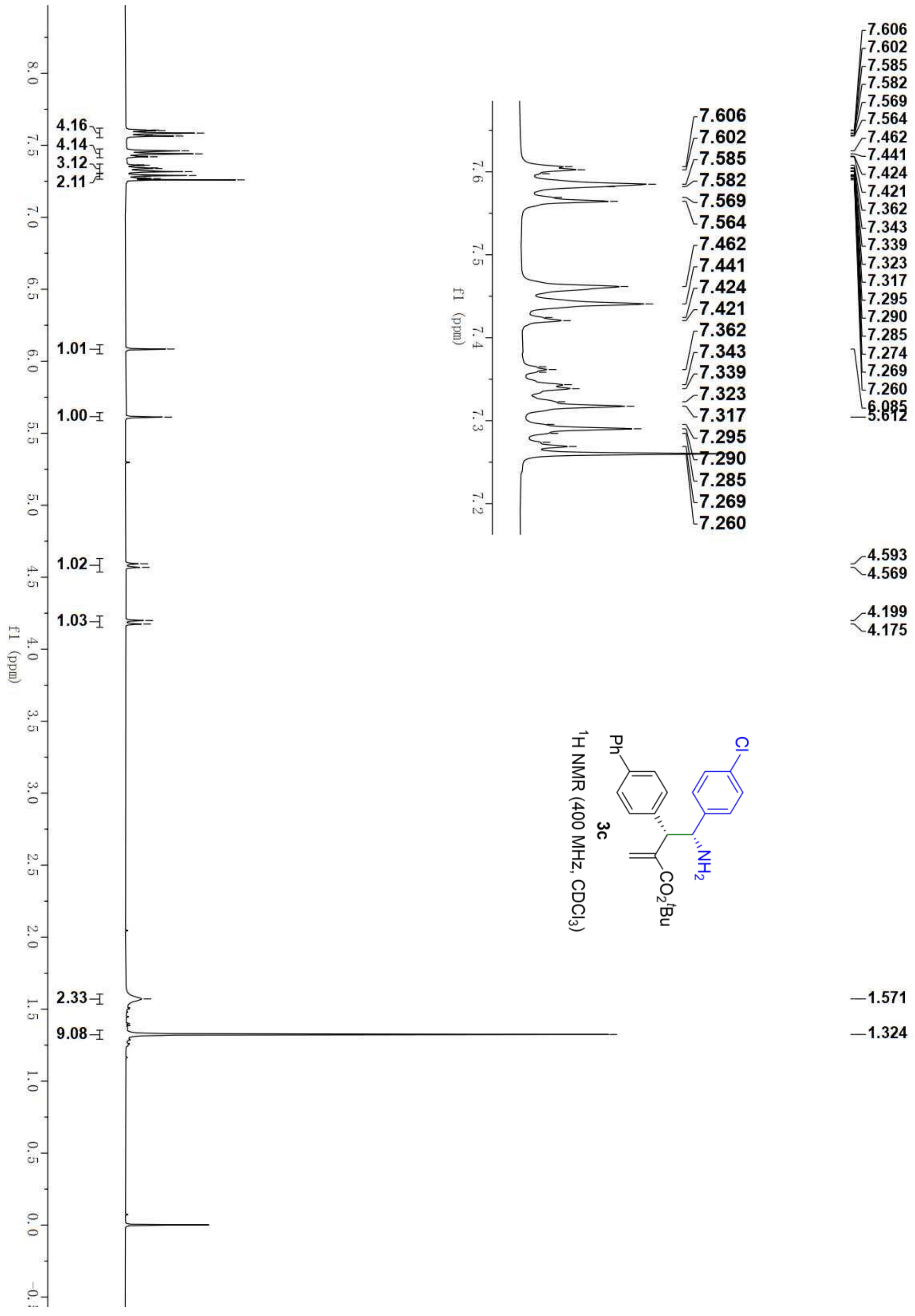
7.606
7.602
7.585
7.582
7.569
7.564
7.462
7.441
7.424
7.421
7.362
7.343
7.339
7.323
7.317
7.295
7.290
7.285
7.274
7.269
7.260
6.085
5.612
4.593
4.569
4.199
4.175
1.571
1.324
4.16
4.14
3.12
2.11
1.01
1.00
1.02
1.03
2.33
9.08
f1 (ppm)
Cl
Ph
NH2
CO2tBu
3c
1H NMR (400 MHz, CDCl3)

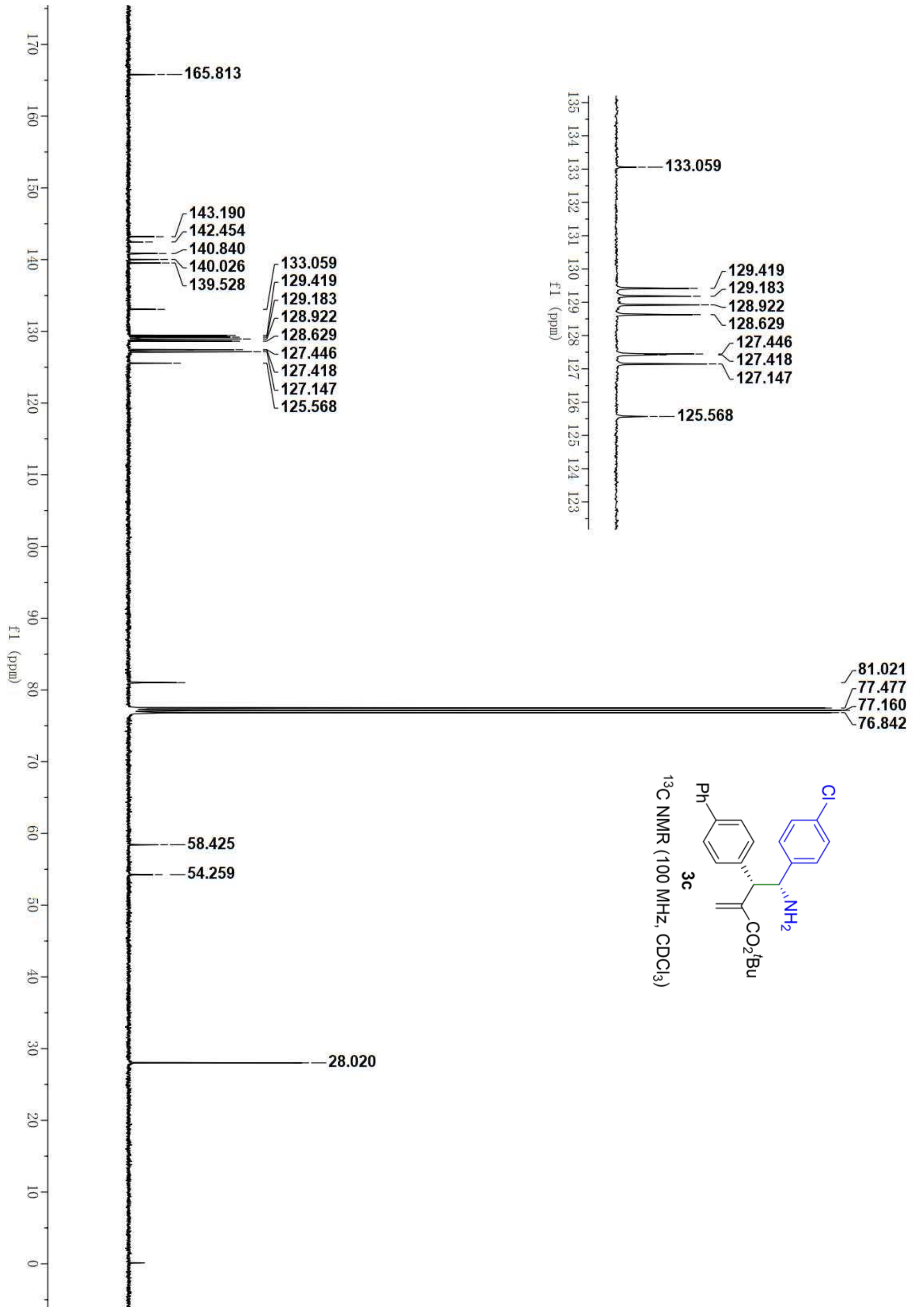
165.813
143.190
142.454
140.840
140.026
139.528
133.059
129.419
129.183
128.922
128.629
127.446
127.418
127.147
125.568
81.021
77.477
77.160
76.842
58.425
54.259
28.020
f1 (ppm)
Cl
Ph
NH2
CO2tBu
3c
13C NMR (100 MHz, CDCl3)

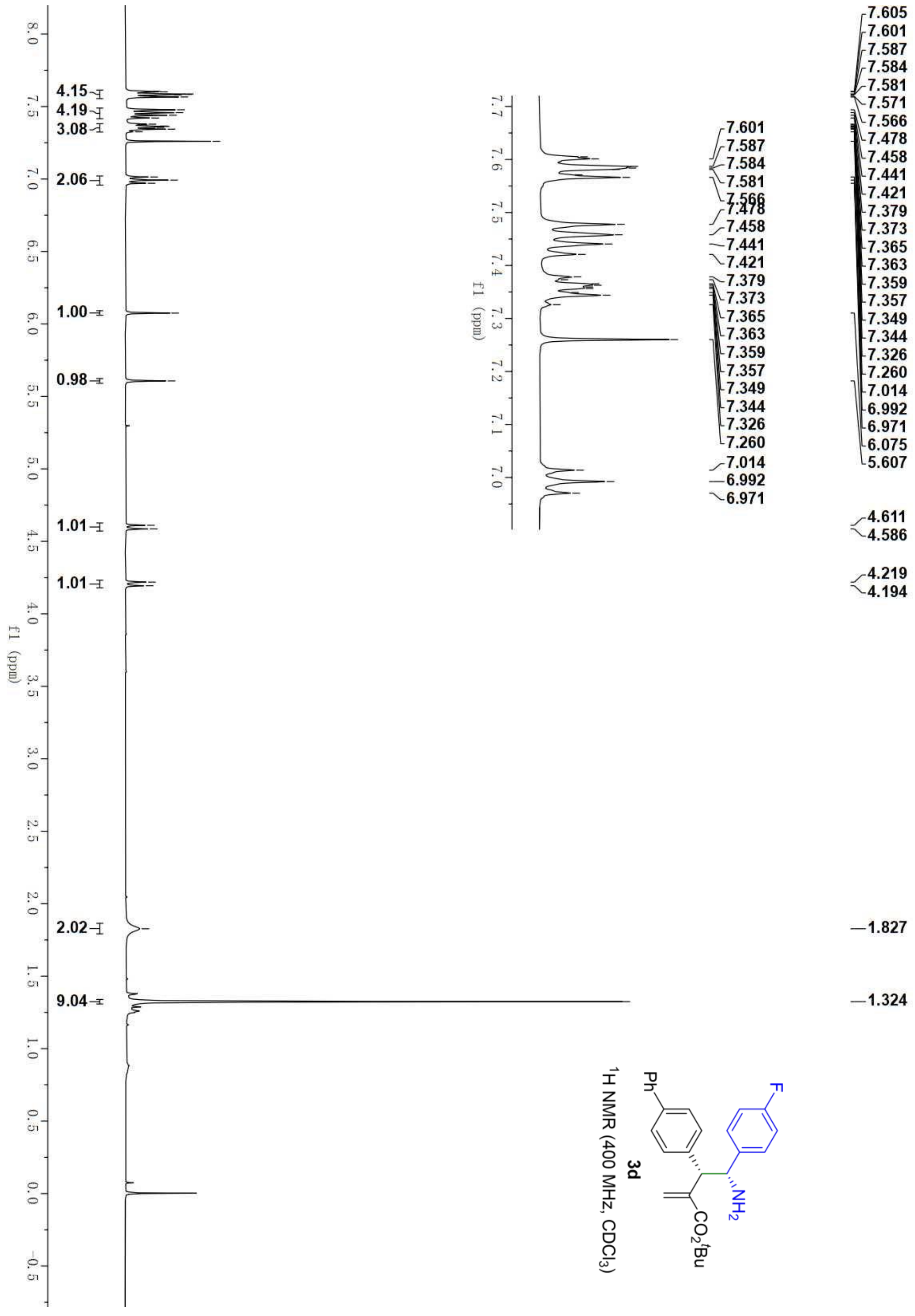
7.605
7.601
7.587
7.584
7.581
7.571
7.566
7.478
7.458
7.441
7.421
7.379
7.373
7.365
7.363
7.359
7.357
7.349
7.344
7.326
7.260
7.014
6.992
6.971
6.075
5.607
4.611
4.586
4.219
4.194
1.827
1.324
4.15
4.19
3.08
2.06
1.00
0.98
1.01
1.01
2.02
9.04
f1 (ppm)
F
NH_2
$CO_2{}^tBu$
Ph
3d
^1H NMR (400 MHz, $CDCl_3$)

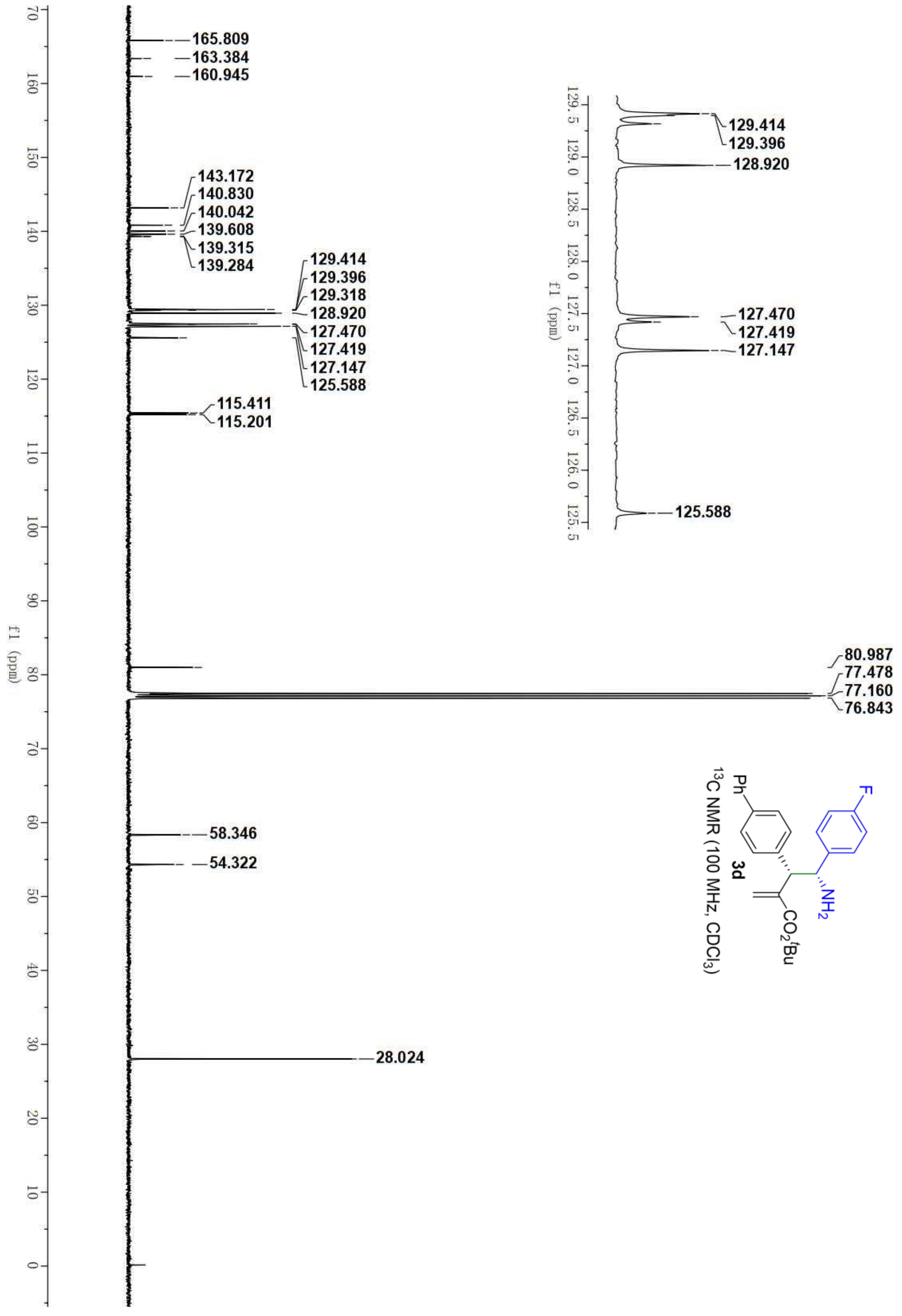
165.809
163.384
160.945
143.172
140.830
140.042
139.608
139.315
139.284
129.414
129.396
129.318
128.920
127.470
127.419
127.147
125.588
115.411
115.201
80.987
77.478
77.160
76.843
58.346
54.322
28.024
f1 (ppm)
3d
13C NMR (100 MHz, CDCl3)

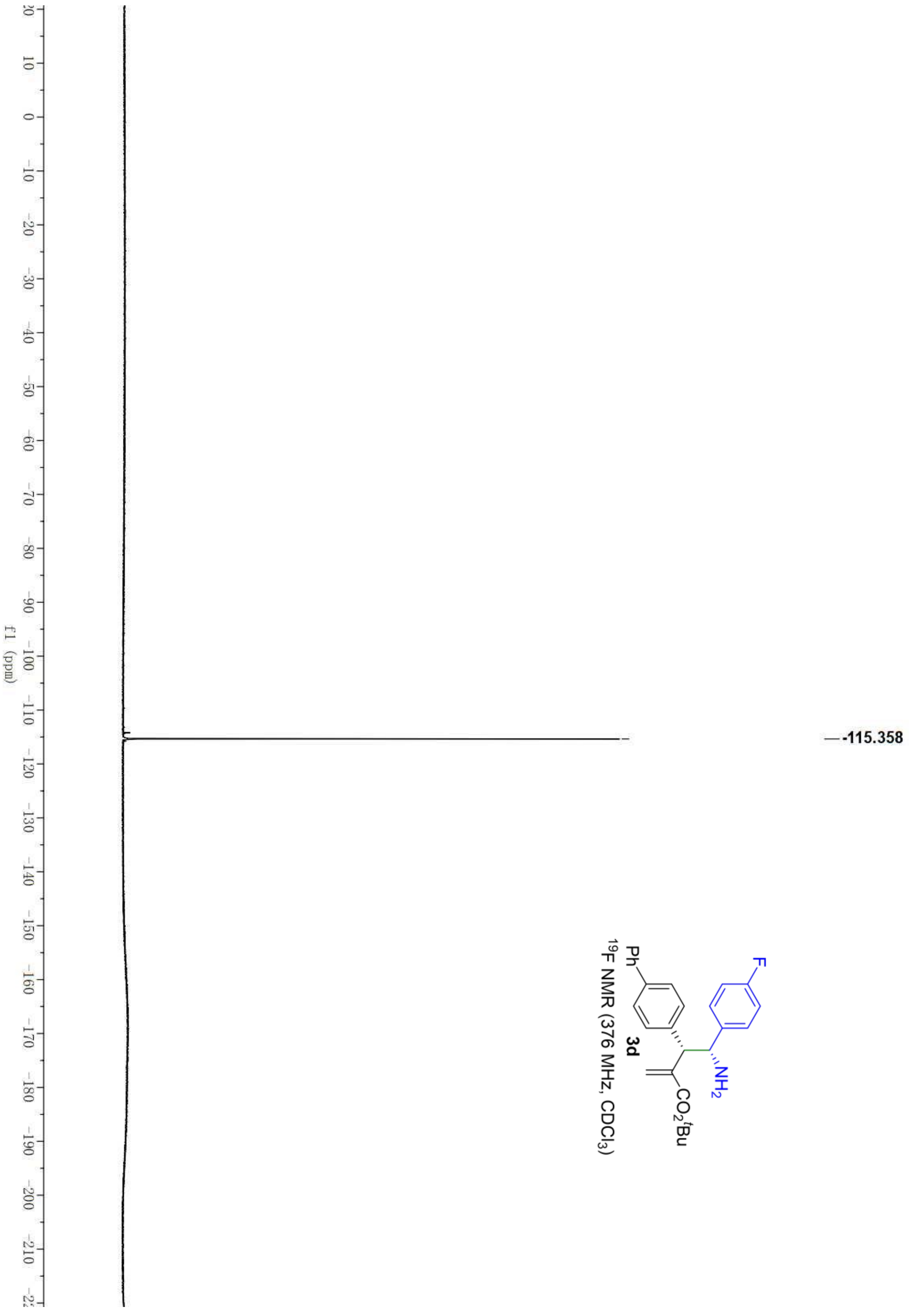

-115.358
F
NH2
CO2tBu
Ph
3d
19F NMR (376 MHz, CDCl3)
f1 (ppm)

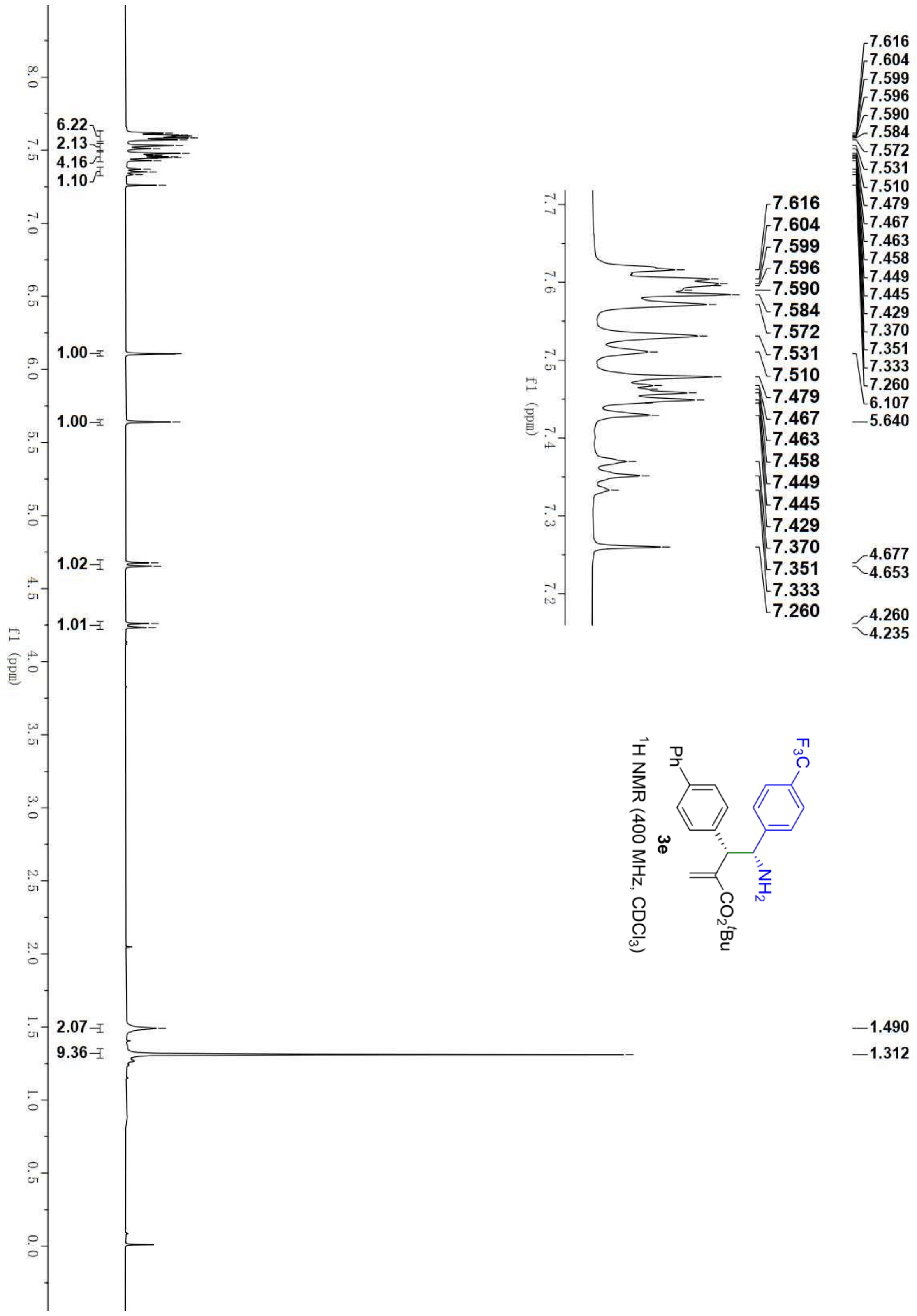

7.616
7.604
7.599
7.596
7.590
7.584
7.572
7.531
7.510
7.479
7.467
7.463
7.458
7.449
7.445
7.429
7.370
7.351
7.333
7.260
6.107
5.640
4.677
4.653
4.260
4.235
1.490
1.312
F3C
NH2
CO2tBu
Ph
3e
1H NMR (400 MHz, CDCl3)
f1 (ppm)

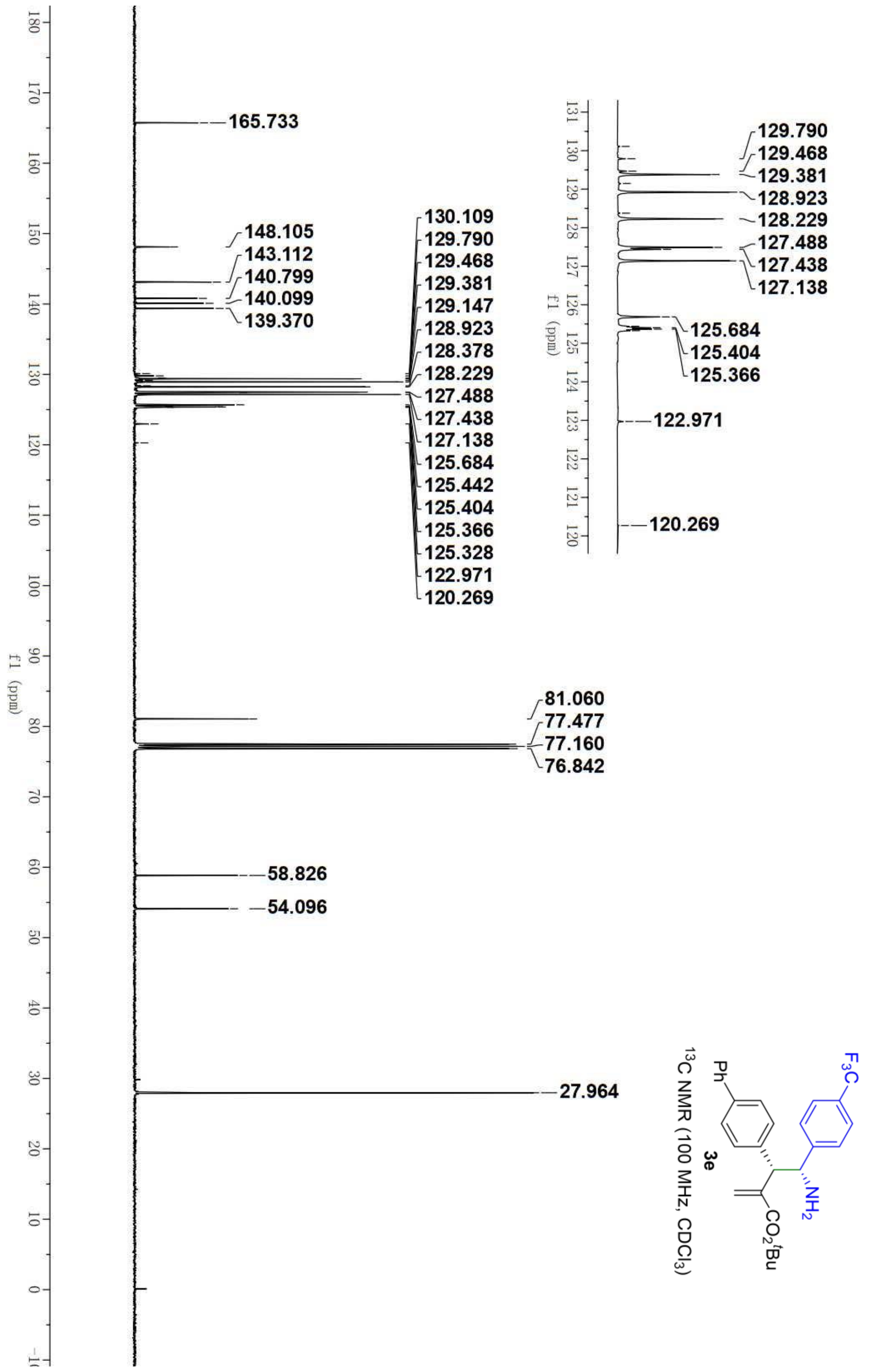
165.733
148.105
143.112
140.799
140.099
139.370
130.109
129.790
129.468
129.381
129.147
128.923
128.378
128.229
127.488
127.438
127.138
125.684
125.442
125.404
125.366
125.328
122.971
120.269
81.060
77.477
77.160
76.842
58.826
54.096
27.964
f1 (ppm)
3e
13C NMR (100 MHz, CDCl3)

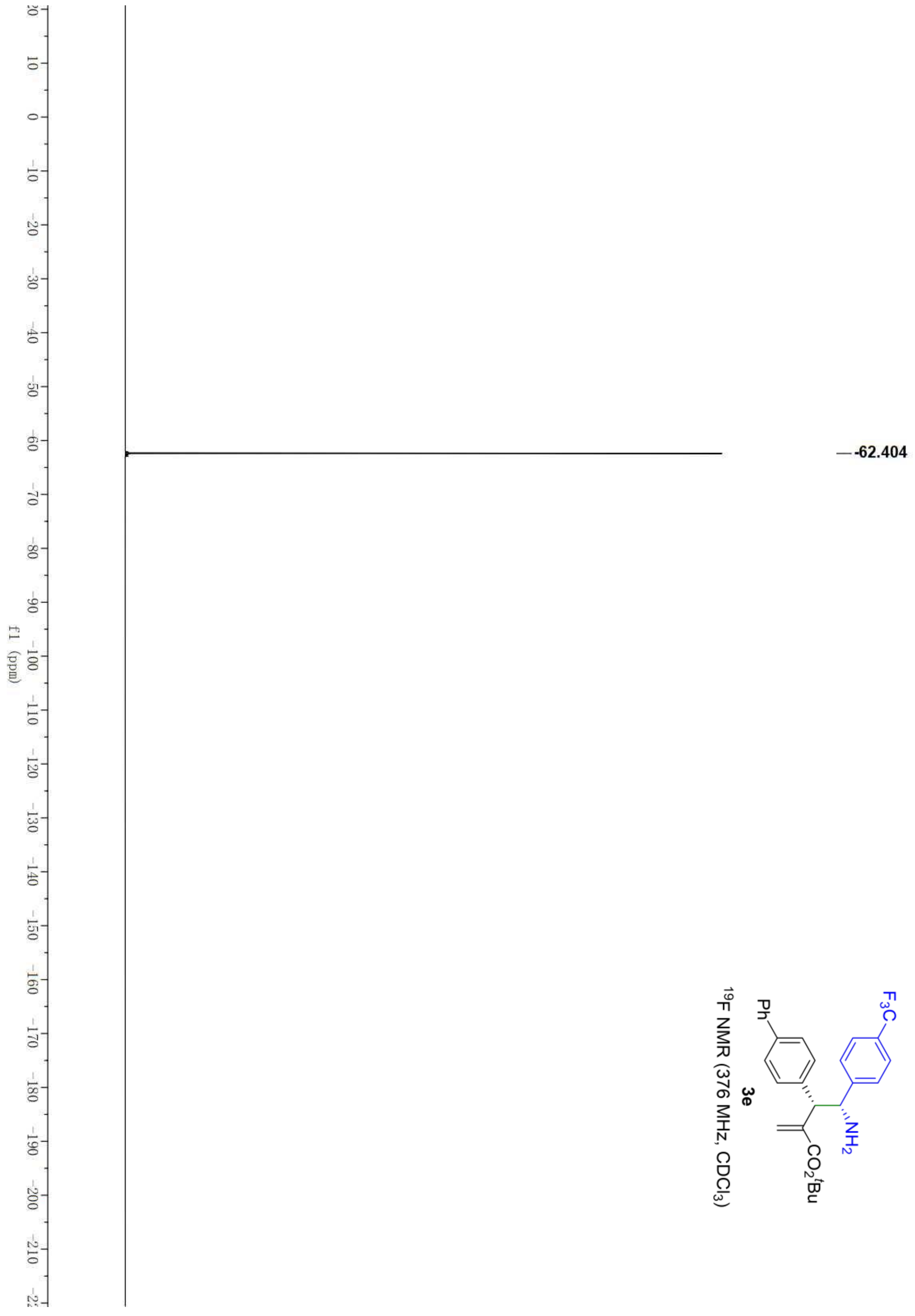

-62.404
f1 (ppm)
F3C
NH2
CO2tBu
Ph
3e
19F NMR (376 MHz, CDCl3)

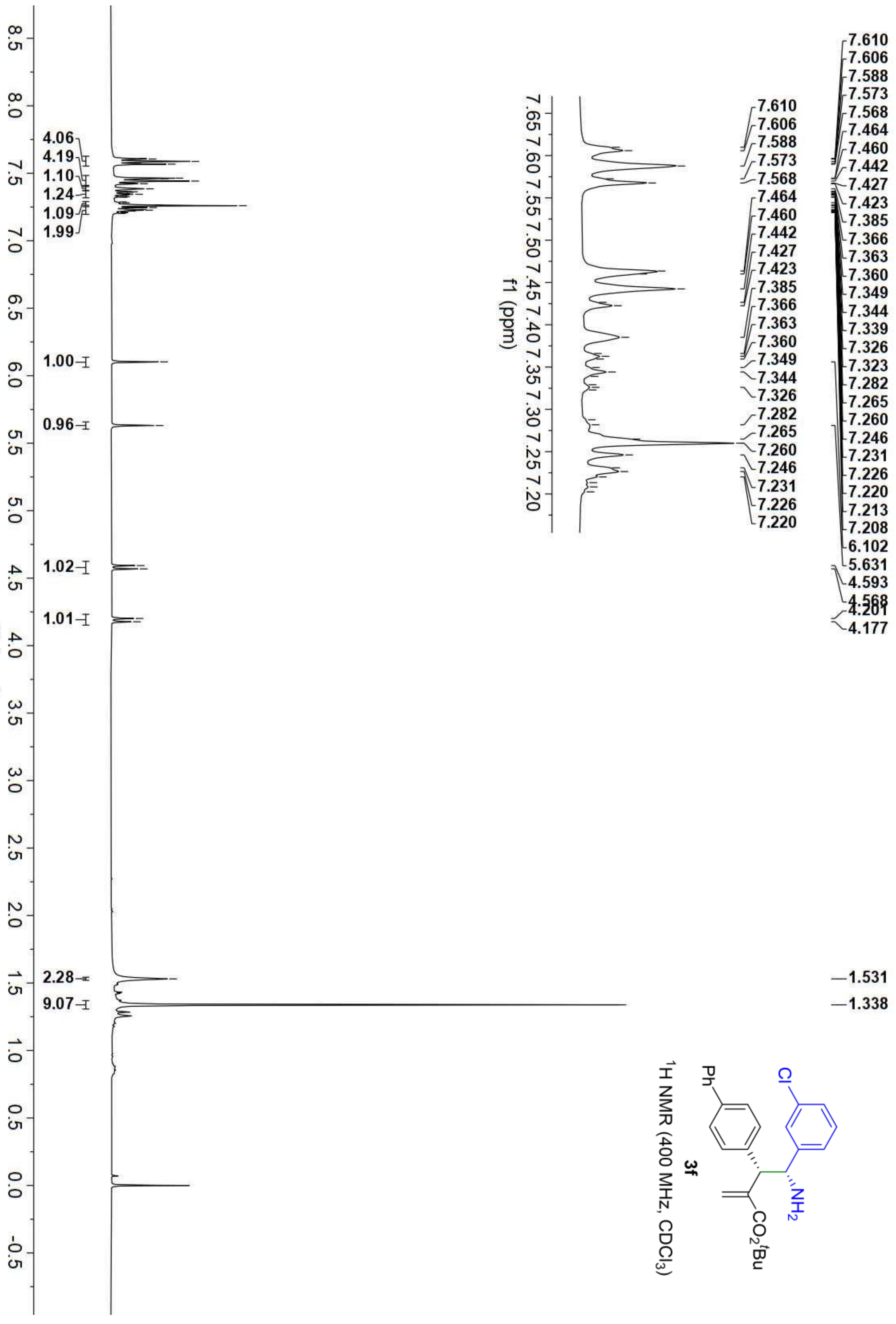

7.610
7.606
7.588
7.573
7.568
7.464
7.460
7.442
7.427
7.423
7.385
7.366
7.363
7.360
7.349
7.344
7.339
7.326
7.323
7.282
7.265
7.260
7.246
7.231
7.226
7.220
7.213
7.208
6.102
5.631
4.593
4.568
4.201
4.177
1.531
1.338
4.06
4.19
1.10
1.24
1.09
1.99
1.00
0.96
1.02
1.01
2.28
9.07
f1 (ppm)
Cl
NH2
Ph
CO2tBu
3f
1H NMR (400 MHz, CDCl3)

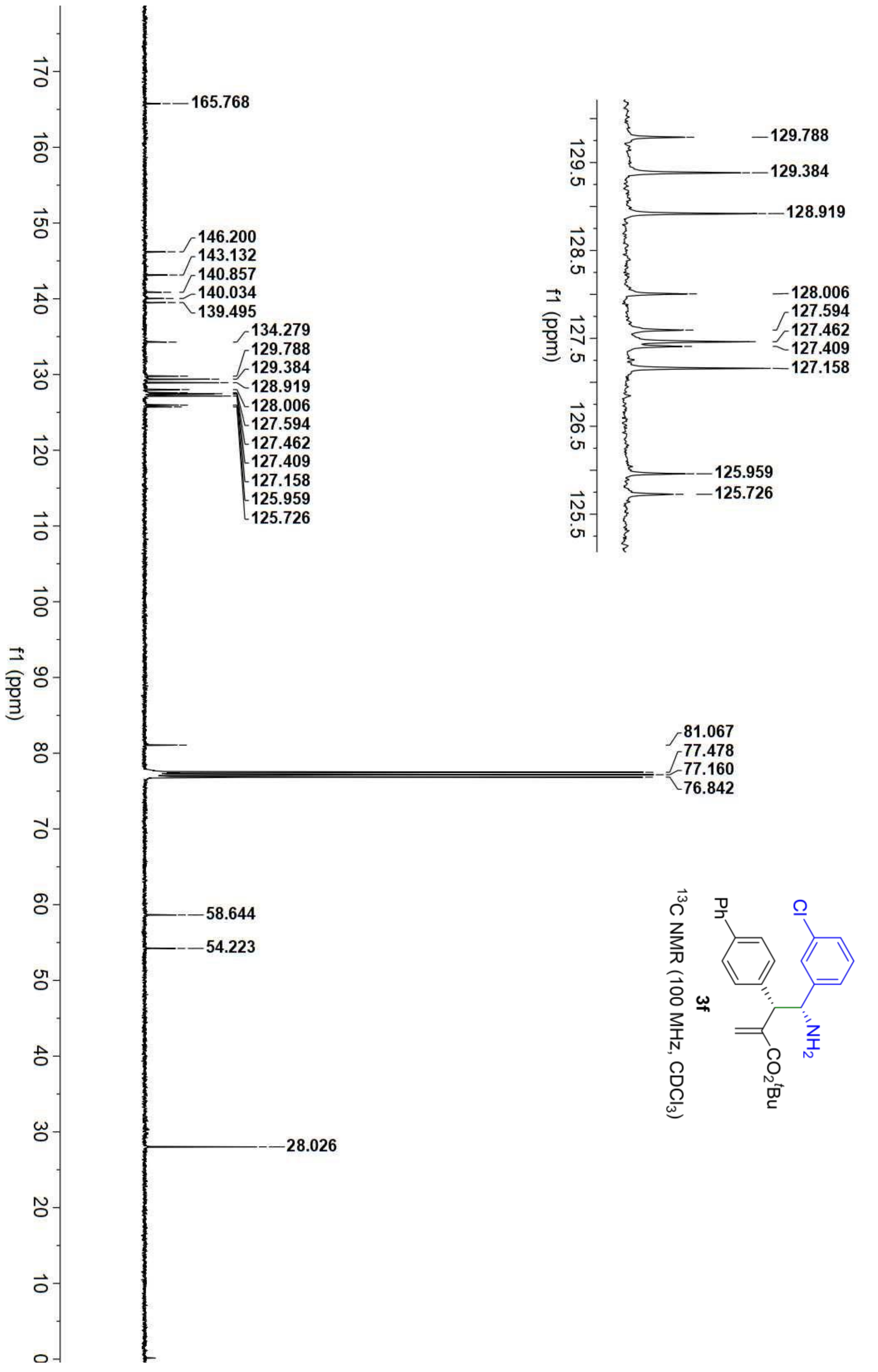
165.768
146.200
143.132
140.857
140.034
139.495
134.279
129.788
129.384
128.919
128.006
127.594
127.462
127.409
127.158
125.959
125.726
81.067
77.478
77.160
76.842
58.644
54.223
28.026
f1 (ppm)
Cl
Ph
NH2
CO2tBu
3f
13C NMR (100 MHz, CDCl3)

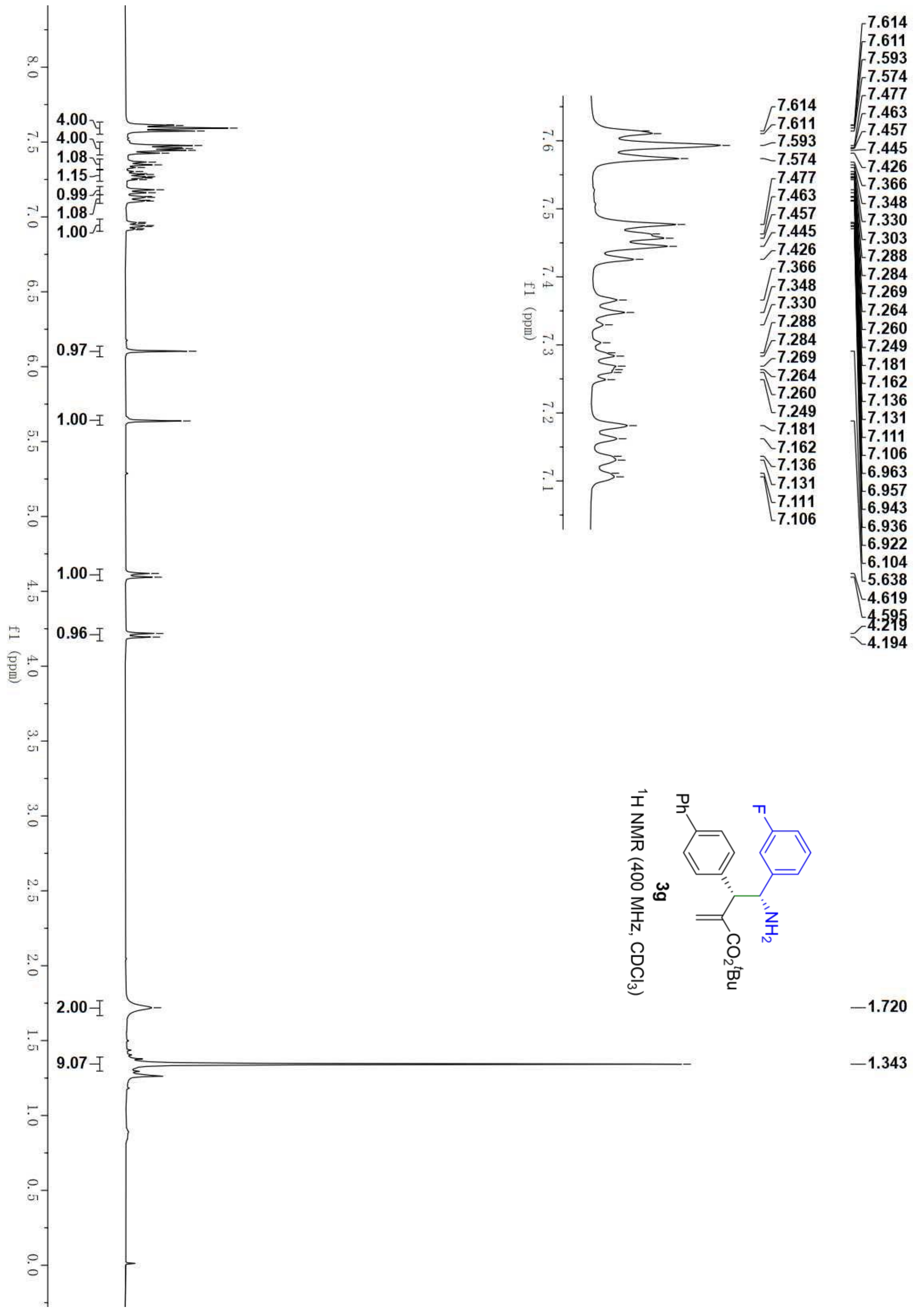

7.614
7.611
7.593
7.574
7.477
7.463
7.457
7.445
7.426
7.366
7.348
7.330
7.303
7.288
7.284
7.269
7.264
7.260
7.249
7.181
7.162
7.136
7.131
7.111
7.106
6.963
6.957
6.943
6.936
6.922
6.104
5.638
4.619
4.595
4.219
4.194
1.720
1.343
4.00
4.00
1.08
1.15
0.99
1.08
1.00
0.97
1.00
1.00
0.96
2.00
9.07
f1 (ppm)
F
NH2
Ph
CO2tBu
3g
1H NMR (400 MHz, CDCl3)

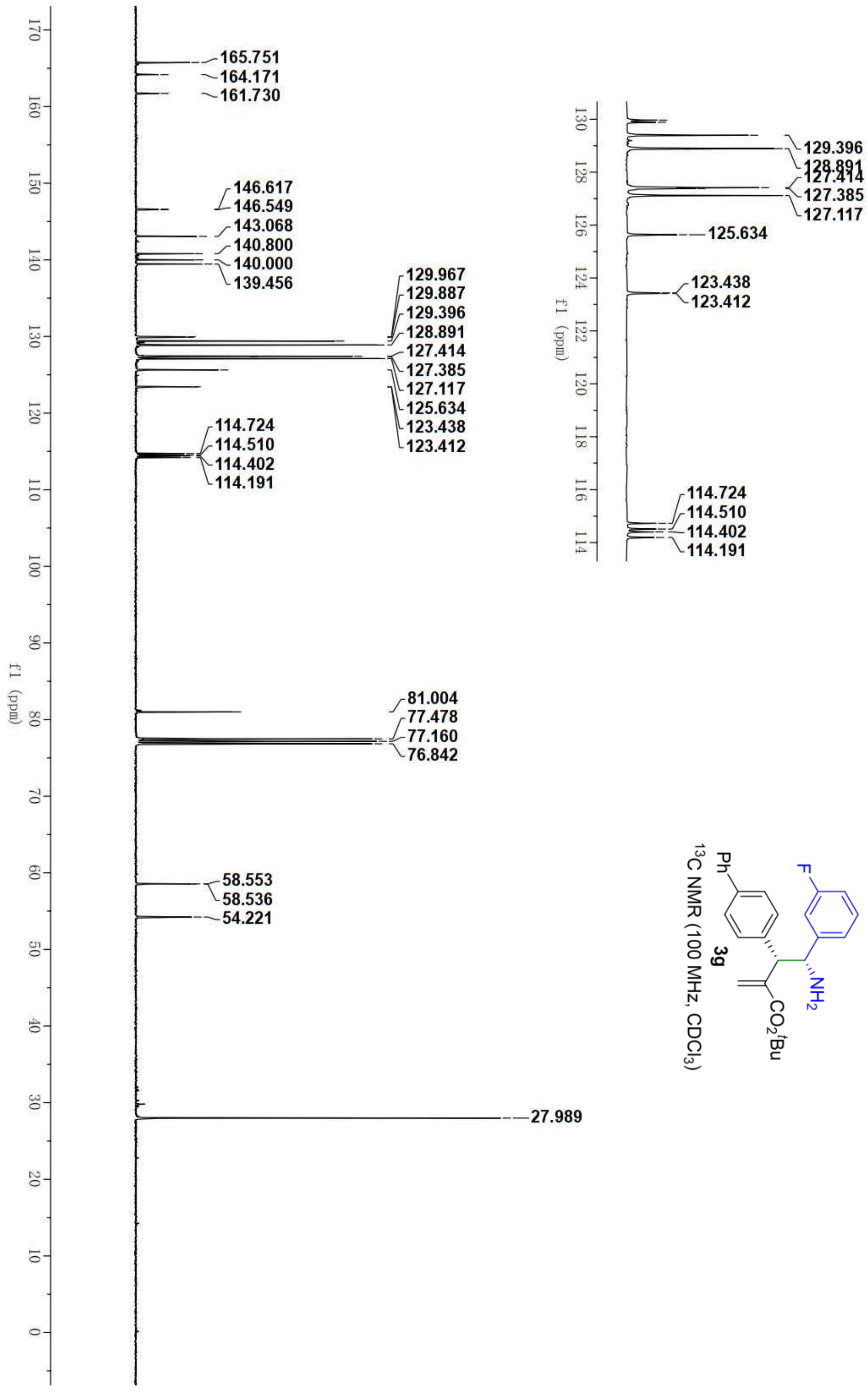
165.751
164.171
161.730
146.617
146.549
143.068
140.800
140.000
139.456
129.967
129.887
129.396
128.891
127.414
127.385
127.117
125.634
123.438
123.412
114.724
114.510
114.402
114.191
81.004
77.478
77.160
76.842
58.553
58.536
54.221
27.989
f1 (ppm)
F
NH2
Ph
CO2tBu
3g
13C NMR (100 MHz, CDCl3)

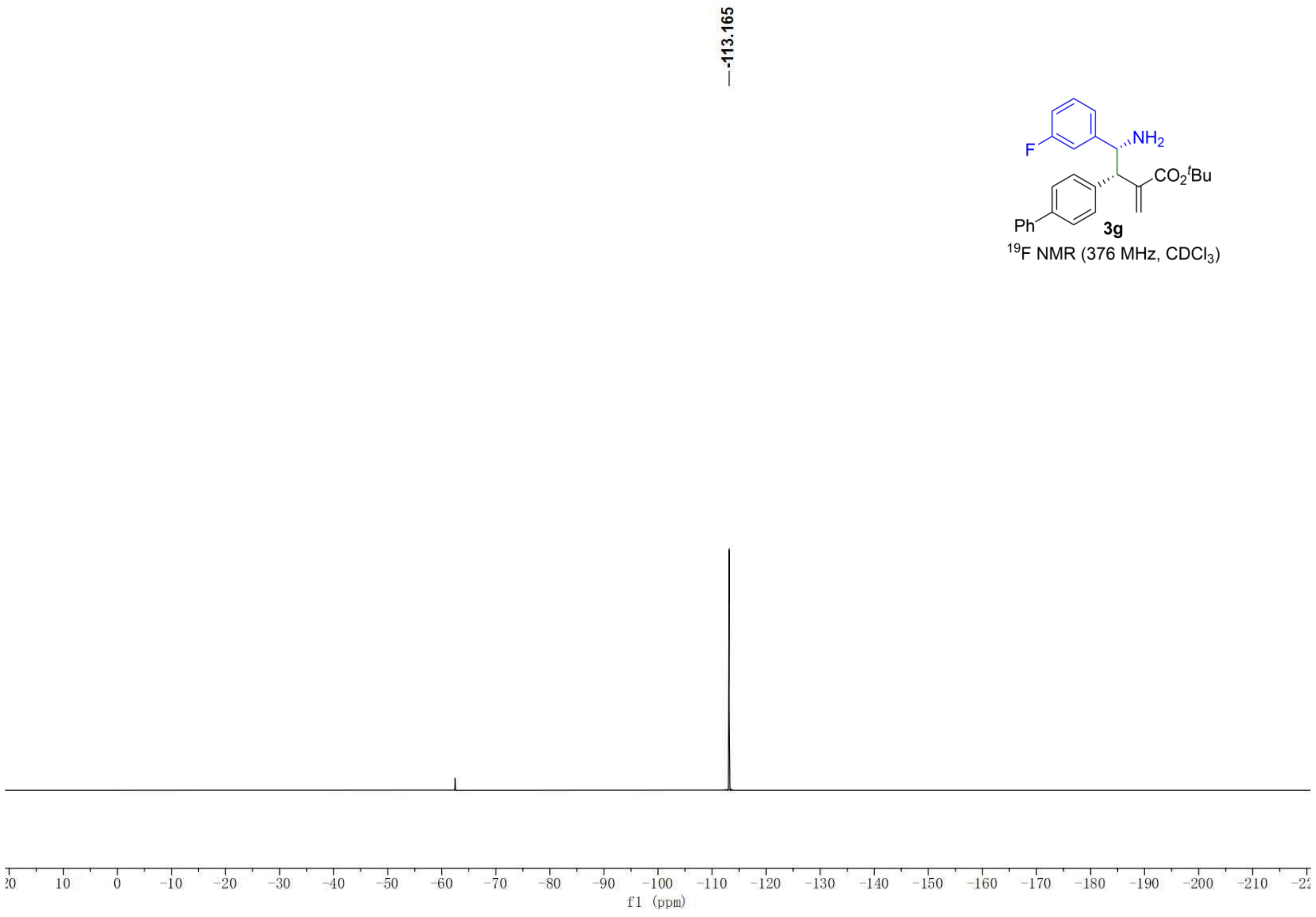
-113.165
F
NH2
CO2tBu
Ph
3g
19F NMR (376 MHz, CDCl3)
20 10 0 -10 -20 -30 -40 -50 -60 -70 -80 -90 -100 -110 -120 -130 -140 -150 -160 -170 -180 -190 -200 -210
f1 (ppm)

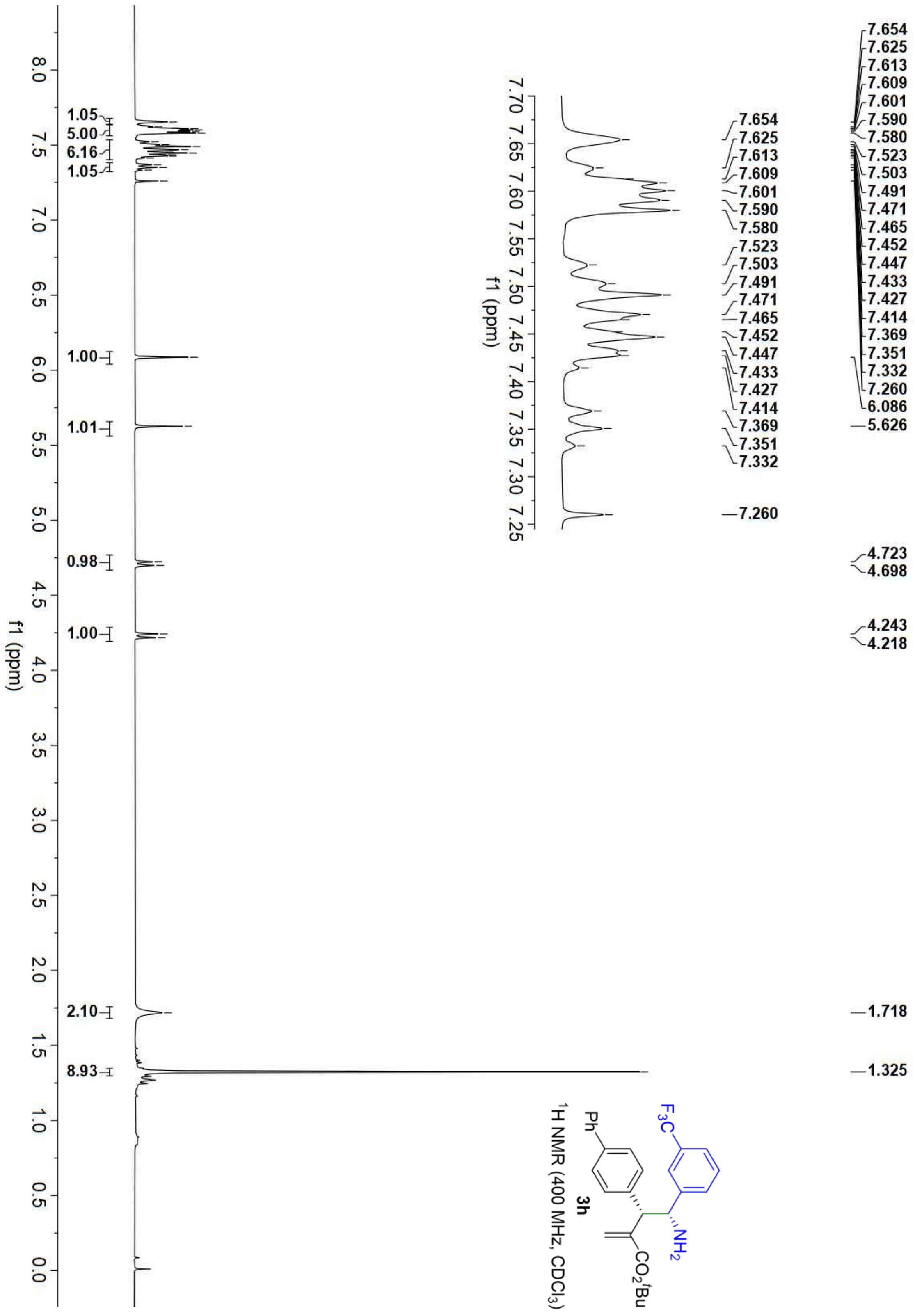

7.654
7.625
7.613
7.609
7.601
7.590
7.580
7.523
7.503
7.491
7.471
7.465
7.452
7.447
7.433
7.427
7.414
7.369
7.351
7.332
7.260
6.086
5.626
4.723
4.698
4.243
4.218
1.718
1.325
1.05
5.00
6.16
1.05
1.00
1.01
0.98
1.00
2.10
8.93
f1 (ppm)
F3C
NH2
Ph
CO2tBu
3h
1H NMR (400 MHz, CDCl3)

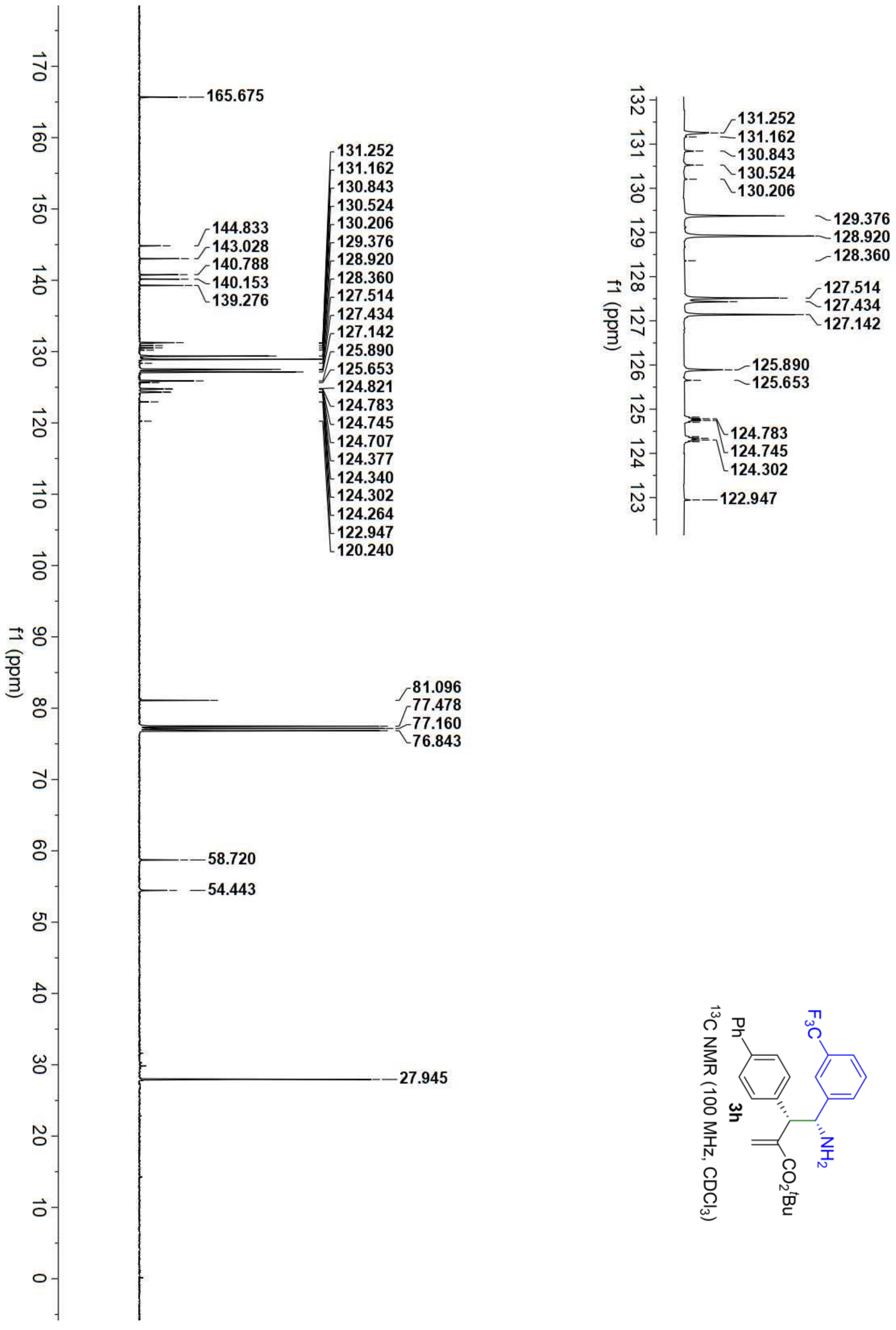
165.675
144.833
143.028
140.788
140.153
139.276
131.252
131.162
130.843
130.524
130.206
129.376
128.920
128.360
127.514
127.434
127.142
125.890
125.653
124.821
124.783
124.745
124.707
124.377
124.340
124.302
124.264
122.947
120.240
81.096
77.478
77.160
76.843
58.720
54.443
27.945
f1 (ppm)
F3C
NH2
Ph
CO2tBu
3h
13C NMR (100 MHz, CDCl3)

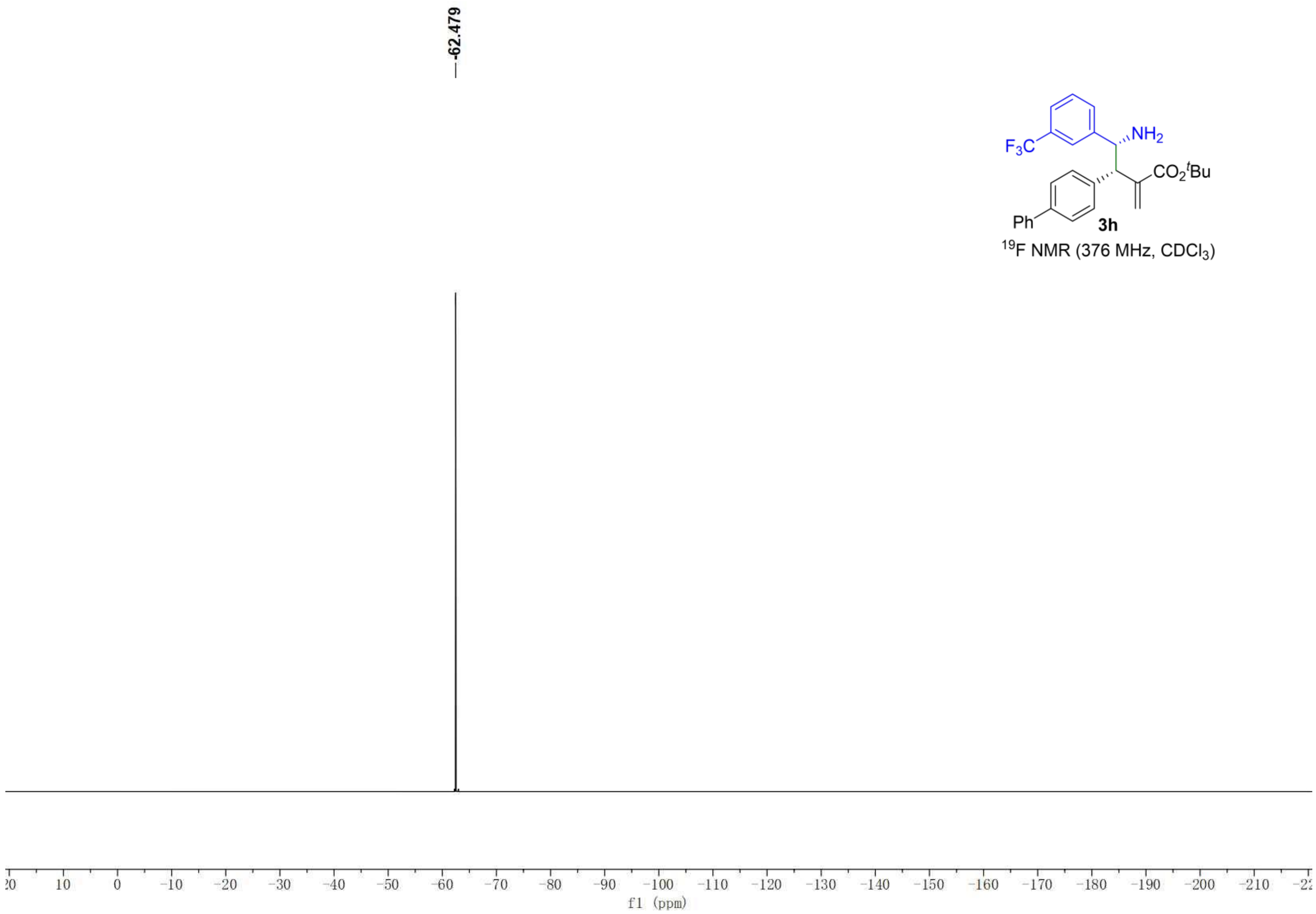

3h

^{19}F NMR (376 MHz, $CDCl_3$)

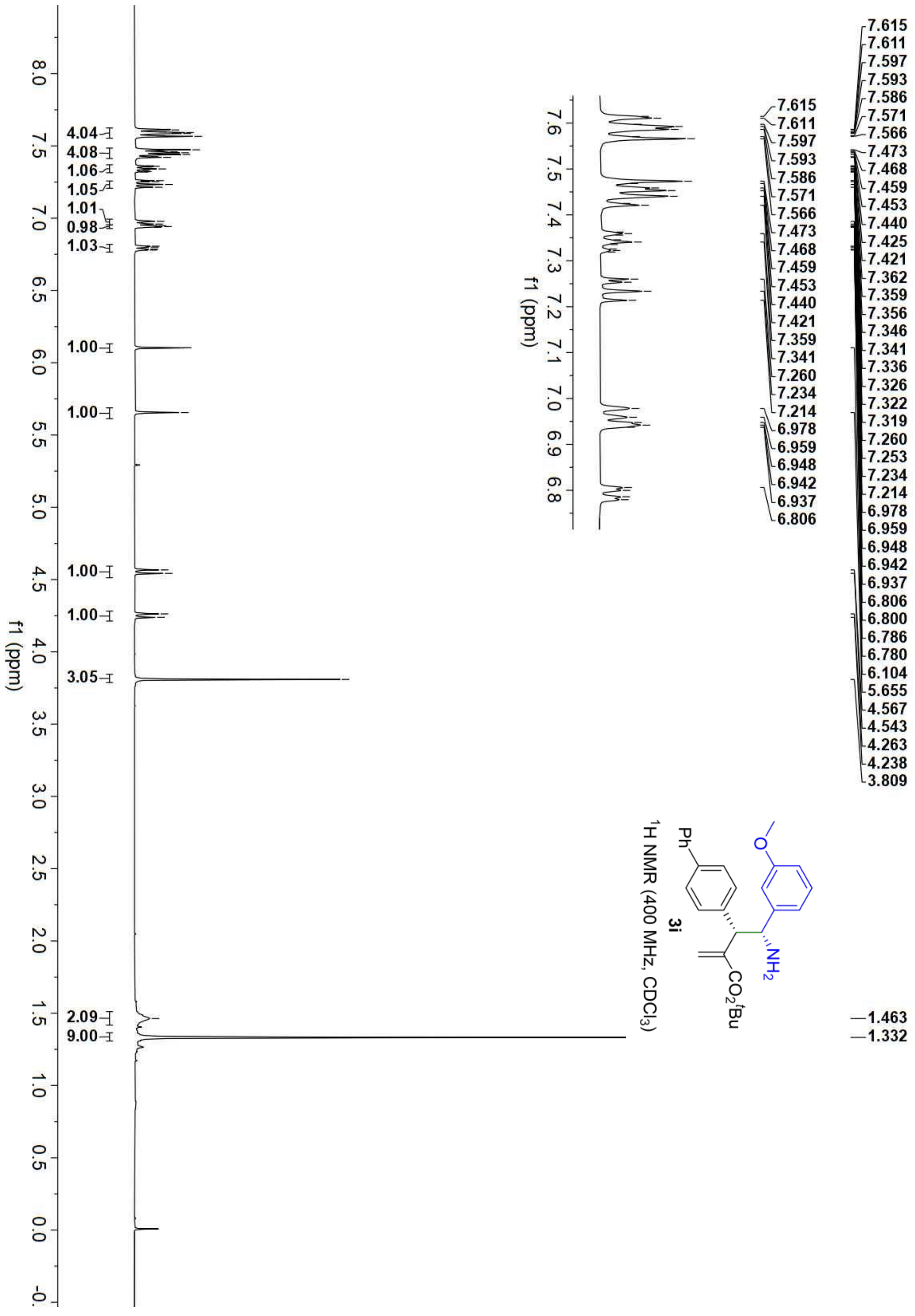
7.615
7.611
7.597
7.593
7.586
7.571
7.566
7.473
7.468
7.459
7.453
7.440
7.425
7.421
7.362
7.359
7.356
7.346
7.341
7.336
7.326
7.322
7.319
7.260
7.253
7.234
7.214
6.978
6.959
6.948
6.942
6.937
6.806
6.800
6.786
6.780
6.104
5.655
4.567
4.543
4.263
4.238
3.809
1.463
1.332
3i
^{1}H NMR (400 MHz, $CDCl_3$)
f1 (ppm)
4.04
4.08
1.06
1.05
1.01
0.98
1.03
1.00
1.00
1.00
1.00
3.05
2.09
9.00

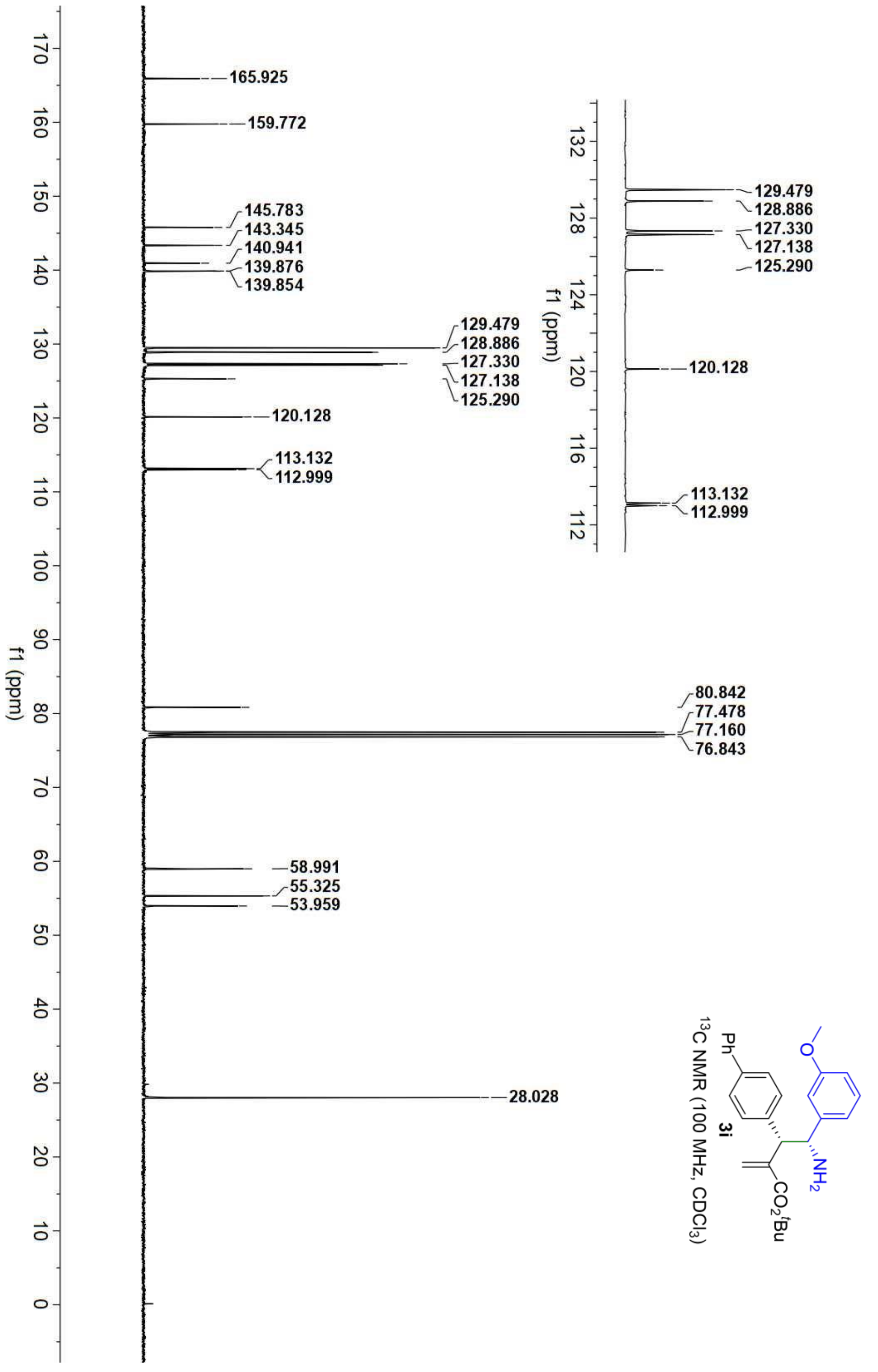
165.925
159.772
145.783
143.345
140.941
139.876
139.854
129.479
128.886
127.330
127.138
125.290
120.128
113.132
112.999
80.842
77.478
77.160
76.843
58.991
55.325
53.959
28.028
f1 (ppm)
Ph
NH2
CO2tBu
3i
13C NMR (100 MHz, CDCl3)

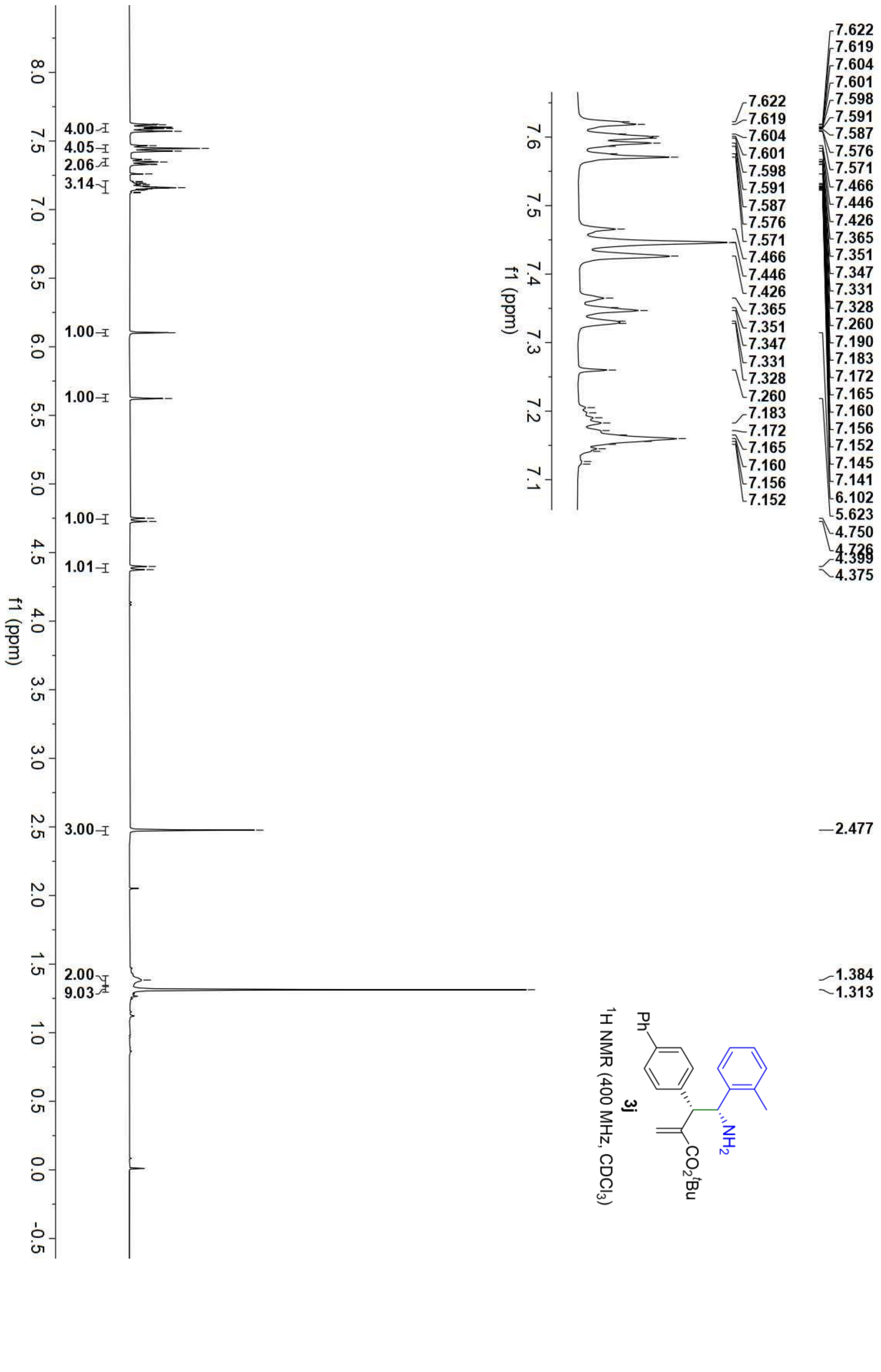
7.622
7.619
7.604
7.601
7.598
7.591
7.587
7.576
7.571
7.466
7.446
7.426
7.365
7.351
7.347
7.331
7.328
7.260
7.190
7.183
7.172
7.165
7.160
7.156
7.152
7.145
7.141
6.102
5.623
4.750
4.726
4.399
4.375
2.477
1.384
1.313
4.00
4.05
2.06
3.14
1.00
1.00
1.00
1.01
3.00
2.00
9.03
f1 (ppm)
Ph
NH2
CO2tBu
3j
1H NMR (400 MHz, CDCl3)

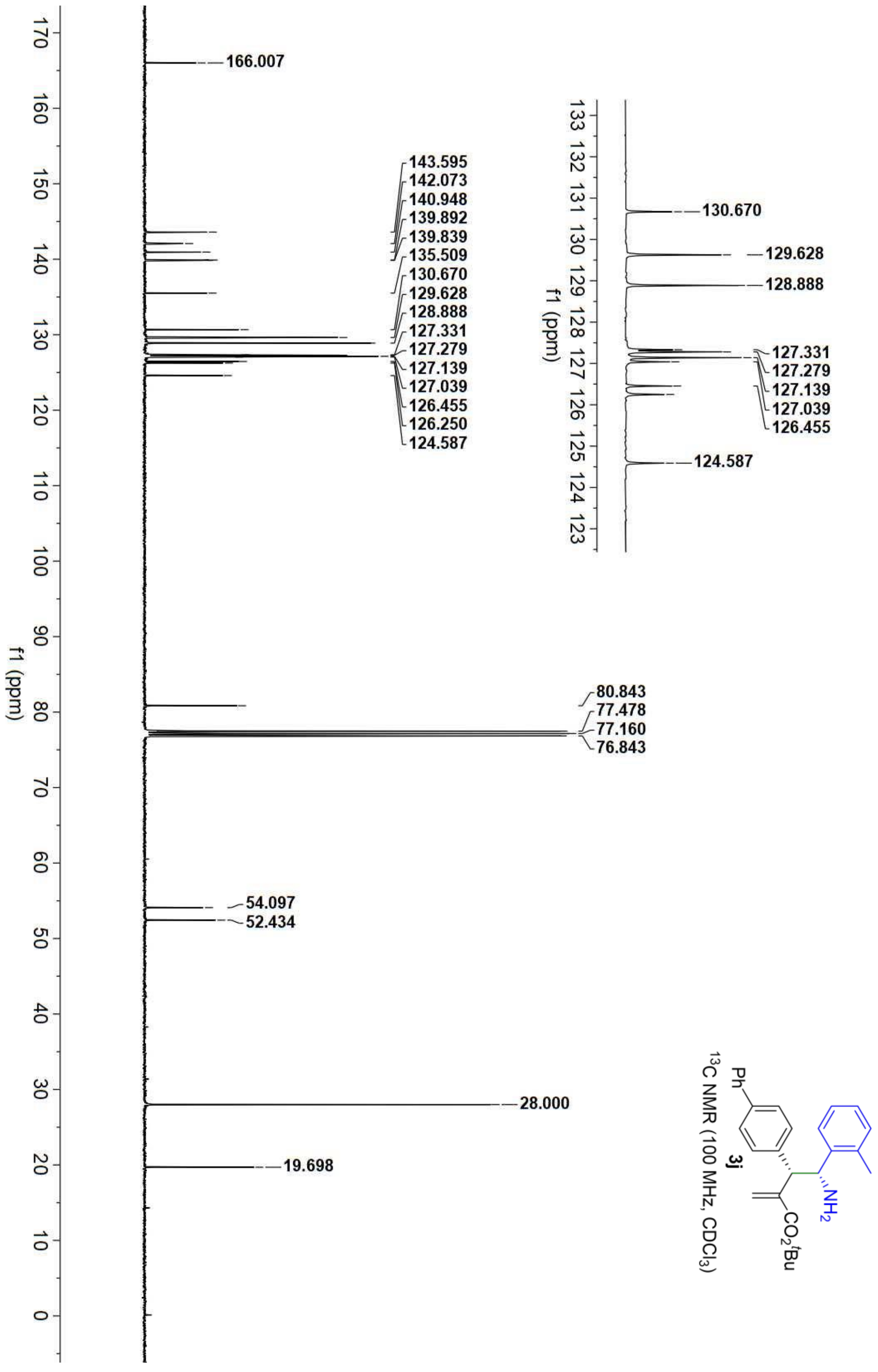
166.007
143.595
142.073
140.948
139.892
139.839
135.509
130.670
129.628
128.888
127.331
127.279
127.139
127.039
126.455
126.250
124.587
80.843
77.478
77.160
76.843
54.097
52.434
28.000
19.698
f1 (ppm)
NH2
CO2tBu
Ph
3j
13C NMR (100 MHz, CDCl3)

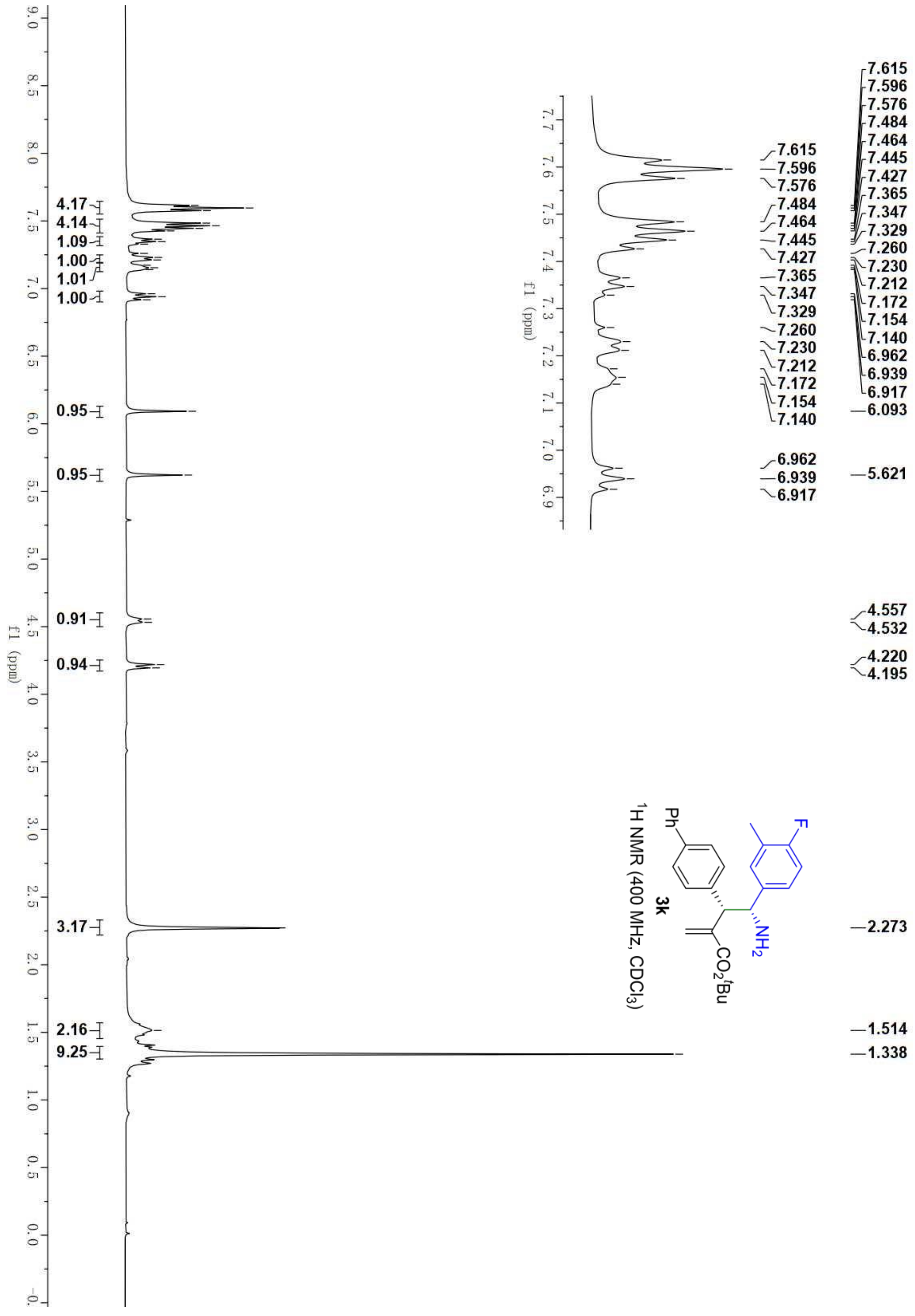

3k
1H NMR (400 MHz, CDCl3)
Ph
F
NH2
CO2tBu
7.615
7.596
7.576
7.484
7.464
7.445
7.427
7.365
7.347
7.329
7.260
7.230
7.212
7.172
7.154
7.140
6.962
6.939
6.917
6.093
5.621
4.557
4.532
4.220
4.195
2.273
1.514
1.338
4.17
4.14
1.09
1.00
1.01
1.00
0.95
0.95
0.91
0.94
3.17
2.16
9.25
f1 (ppm)

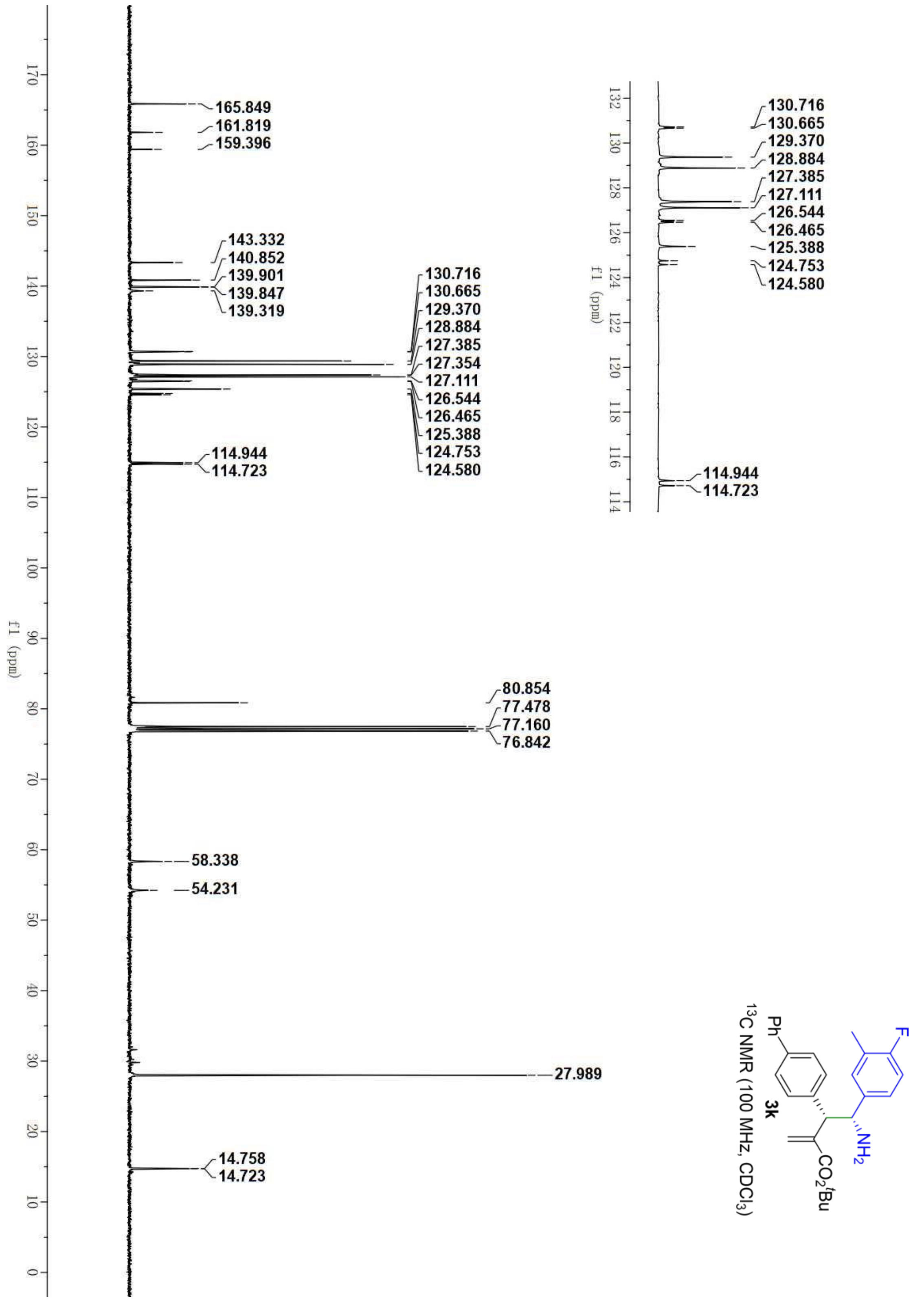
165.849
161.819
159.396
143.332
140.852
139.901
139.847
139.319
130.716
130.665
129.370
128.884
127.385
127.354
127.111
126.544
126.465
125.388
124.753
124.580
114.944
114.723
80.854
77.478
77.160
76.842
58.338
54.231
27.989
14.758
14.723
f1 (ppm)
130.716
130.665
129.370
128.884
127.385
127.111
126.544
126.465
125.388
124.753
124.580
114.944
114.723
f1 (ppm)
F
NH2
CO2tBu
Ph
3k
13C NMR (100 MHz, CDCl3)

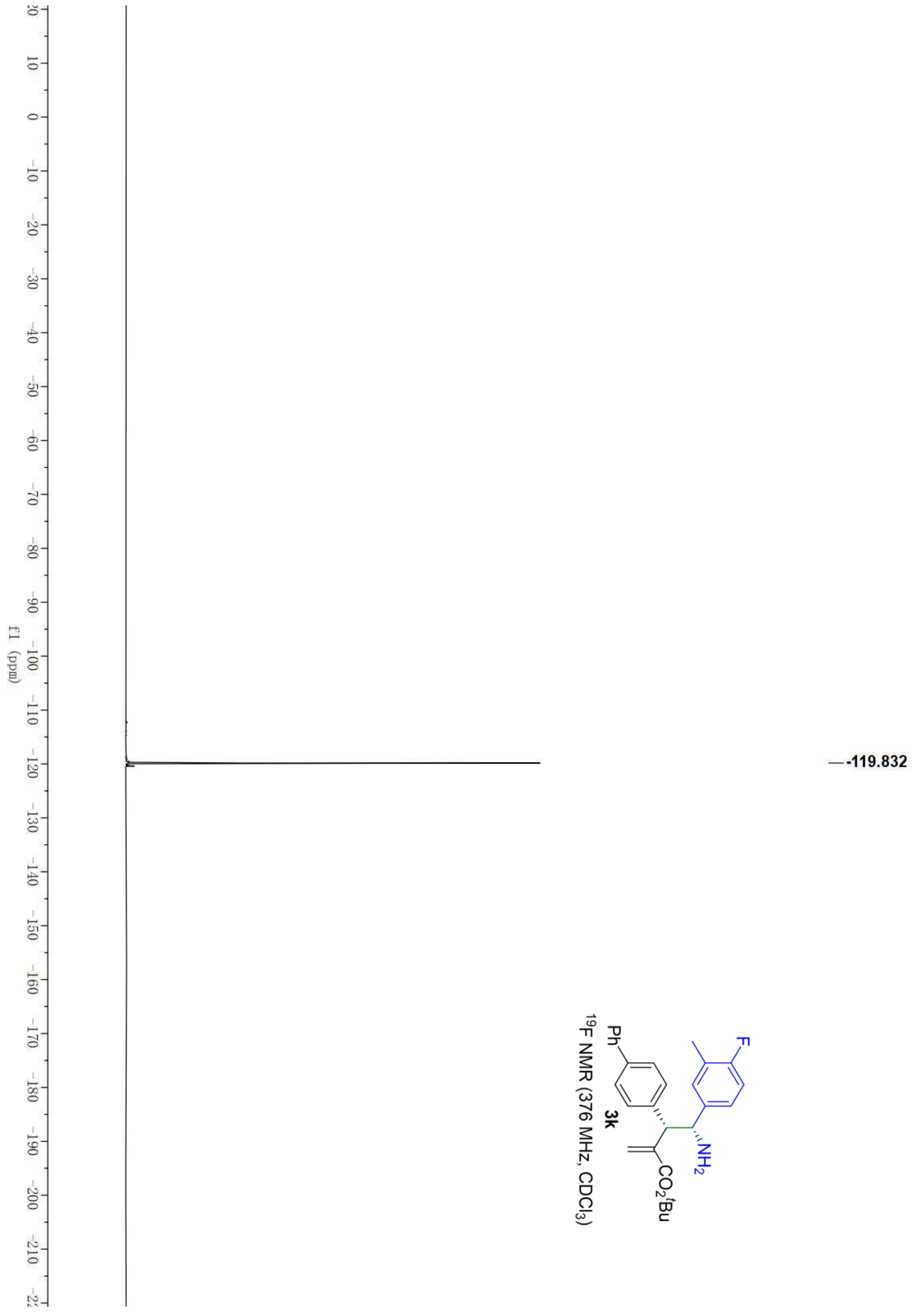
-119.832
3k
19F NMR (376 MHz, CDCl3)
f1 (ppm)

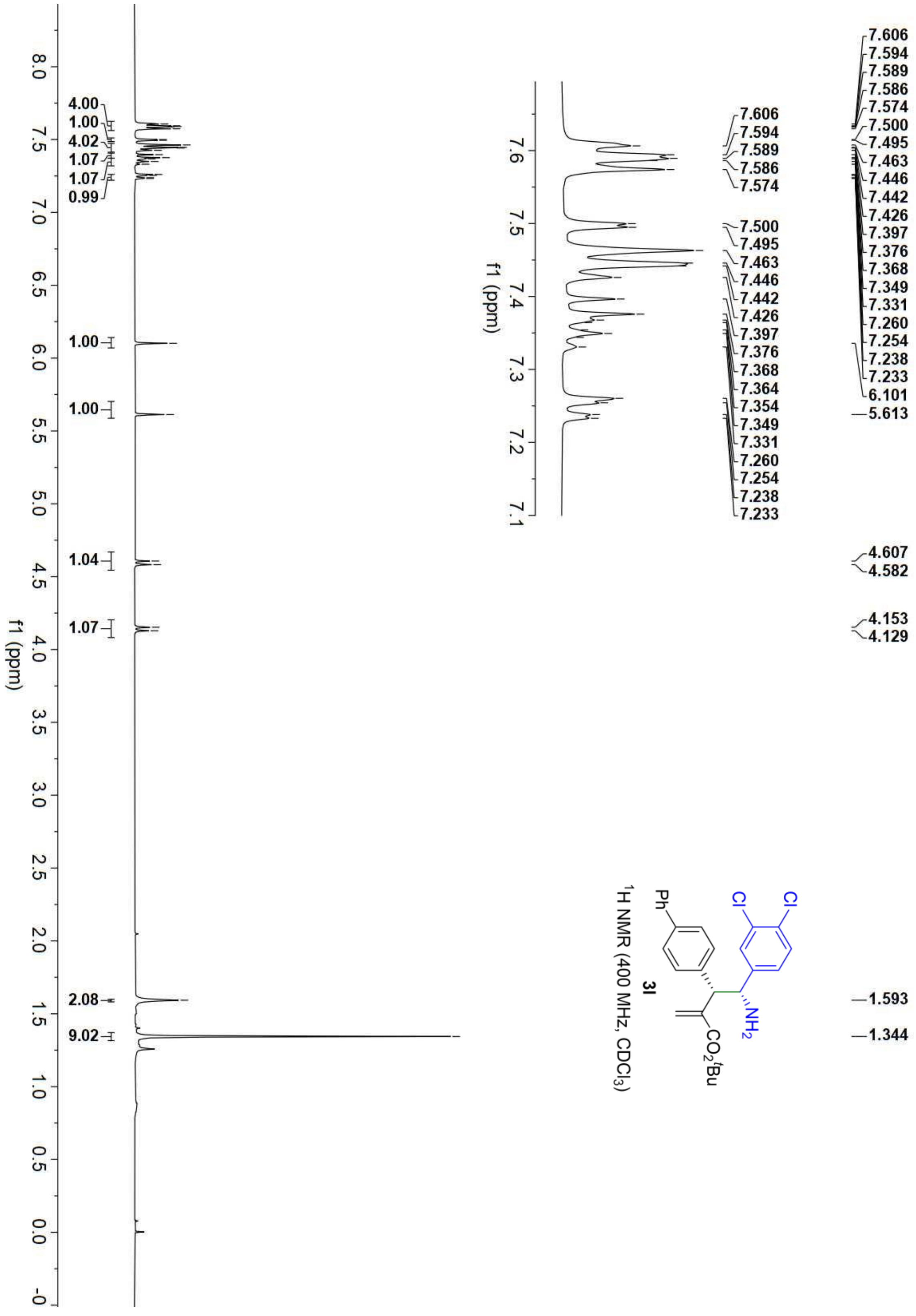
7.606
7.594
7.589
7.586
7.574
7.500
7.495
7.463
7.446
7.442
7.426
7.397
7.376
7.368
7.349
7.331
7.260
7.254
7.238
7.233
6.101
5.613
4.607
4.582
4.153
4.129
1.593
1.344
4.00
1.00
4.02
1.07
1.07
0.99
1.00
1.00
1.04
1.07
2.08
9.02
f1 (ppm)
Cl
Cl
Ph
NH2
CO2tBu
3l
1H NMR (400 MHz, CDCl3)

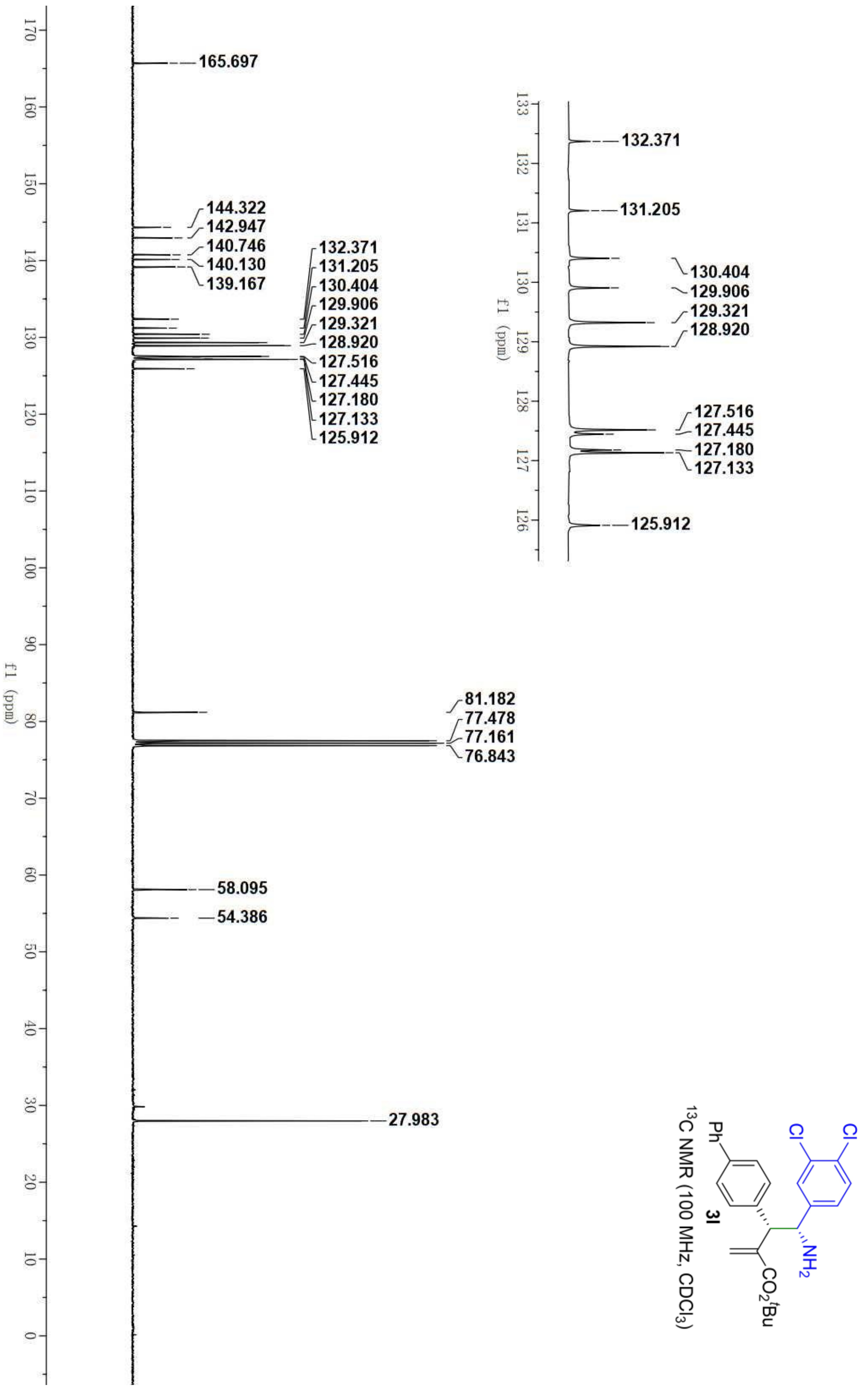

165.697
144.322
142.947
140.746
140.130
139.167
132.371
131.205
130.404
129.906
129.321
128.920
127.516
127.445
127.180
127.133
125.912
81.182
77.478
77.161
76.843
58.095
54.386
27.983
f1 (ppm)
Cl
Cl
NH2
CO2tBu
Ph
3l
13C NMR (100 MHz, CDCl3)

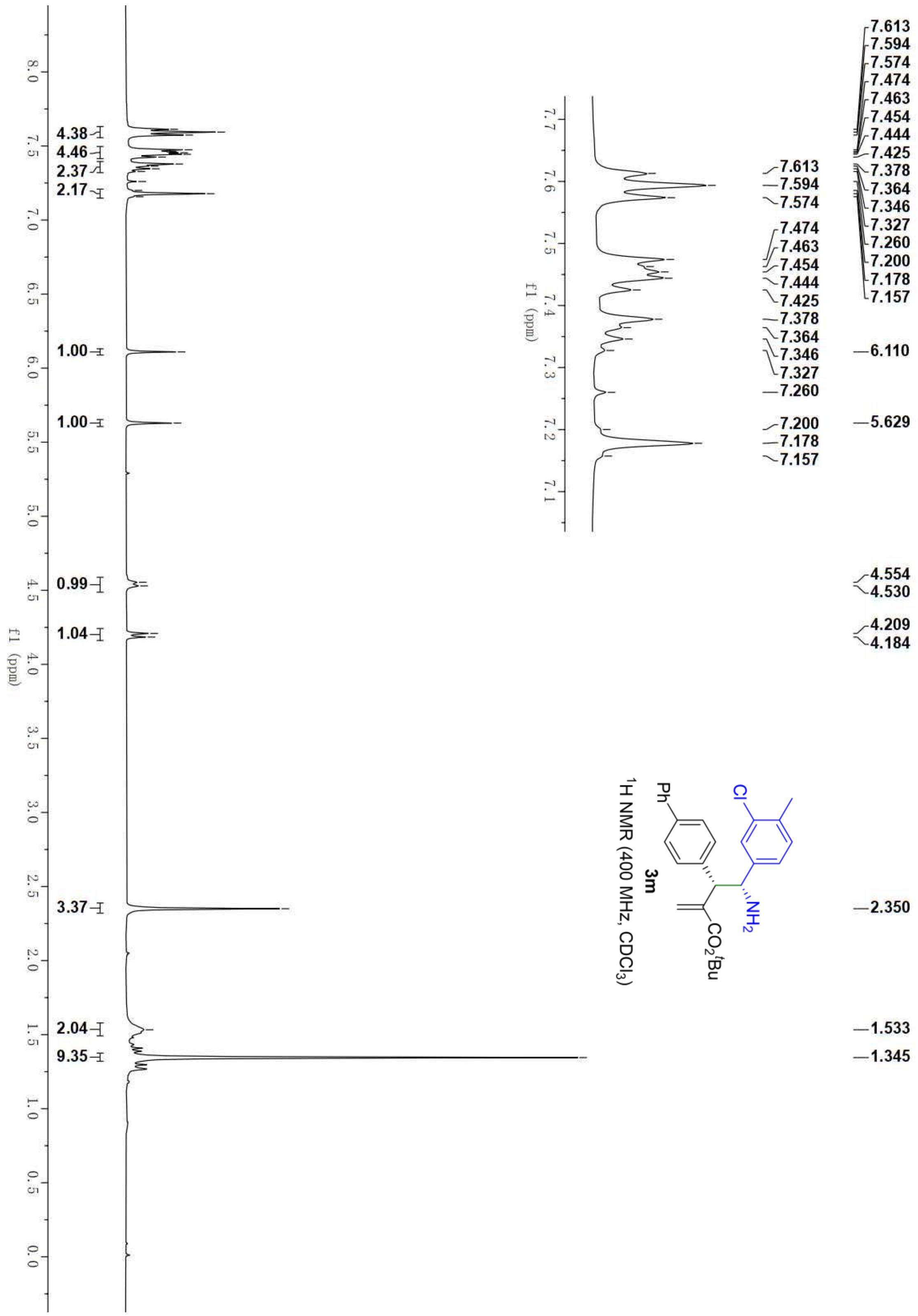
3m
¹H NMR (400 MHz, CDCl₃)
f1 (ppm)

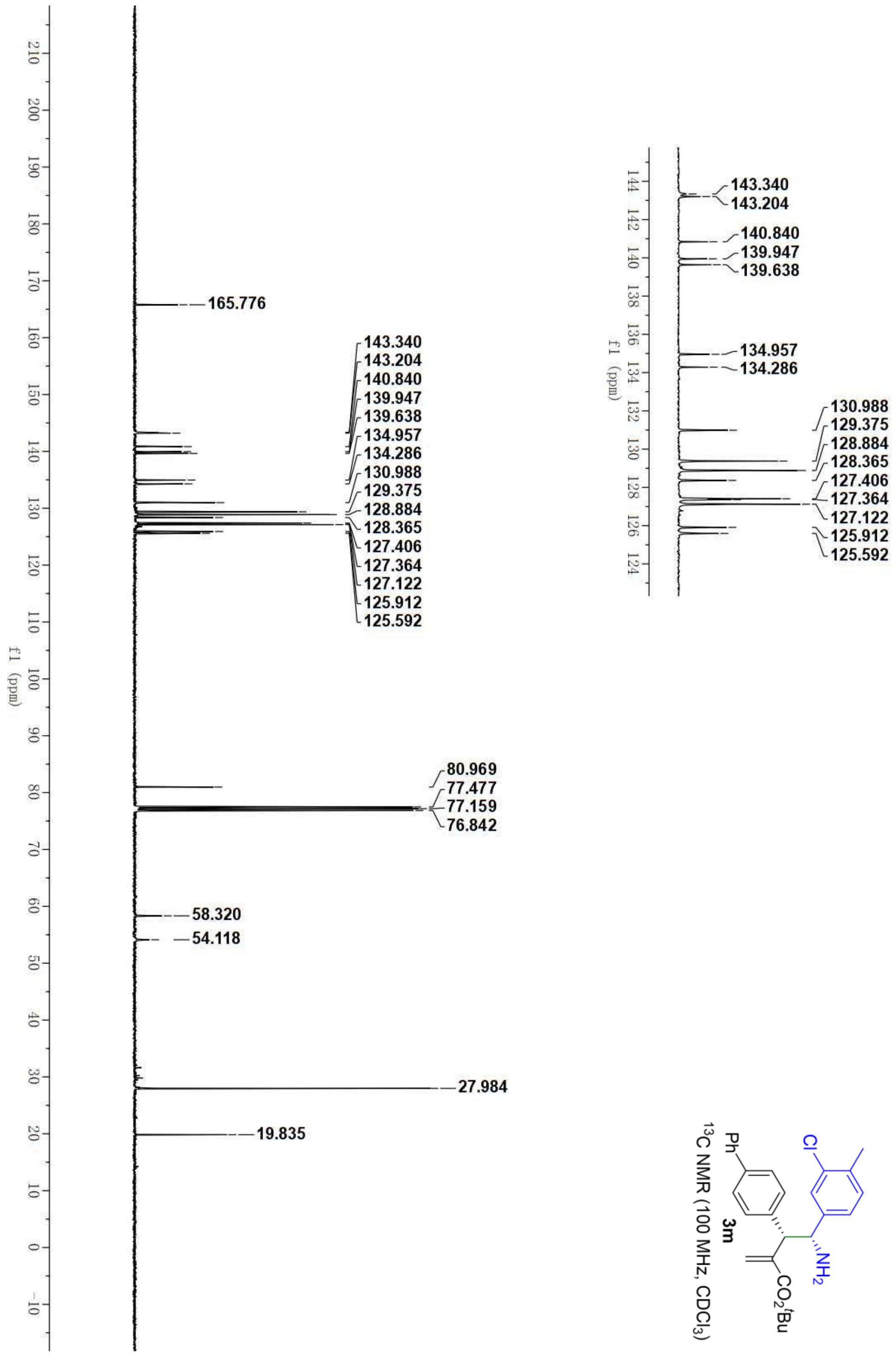
165.776
143.340
143.204
140.840
139.947
139.638
134.957
134.286
130.988
129.375
128.884
128.365
127.406
127.364
127.122
125.912
125.592
80.969
77.477
77.159
76.842
58.320
54.118
27.984
19.835
f1 (ppm)
Cl
Ph
NH_2
$CO_2{}^tBu$
3m
^{13}C NMR (100 MHz, $CDCl_3$)

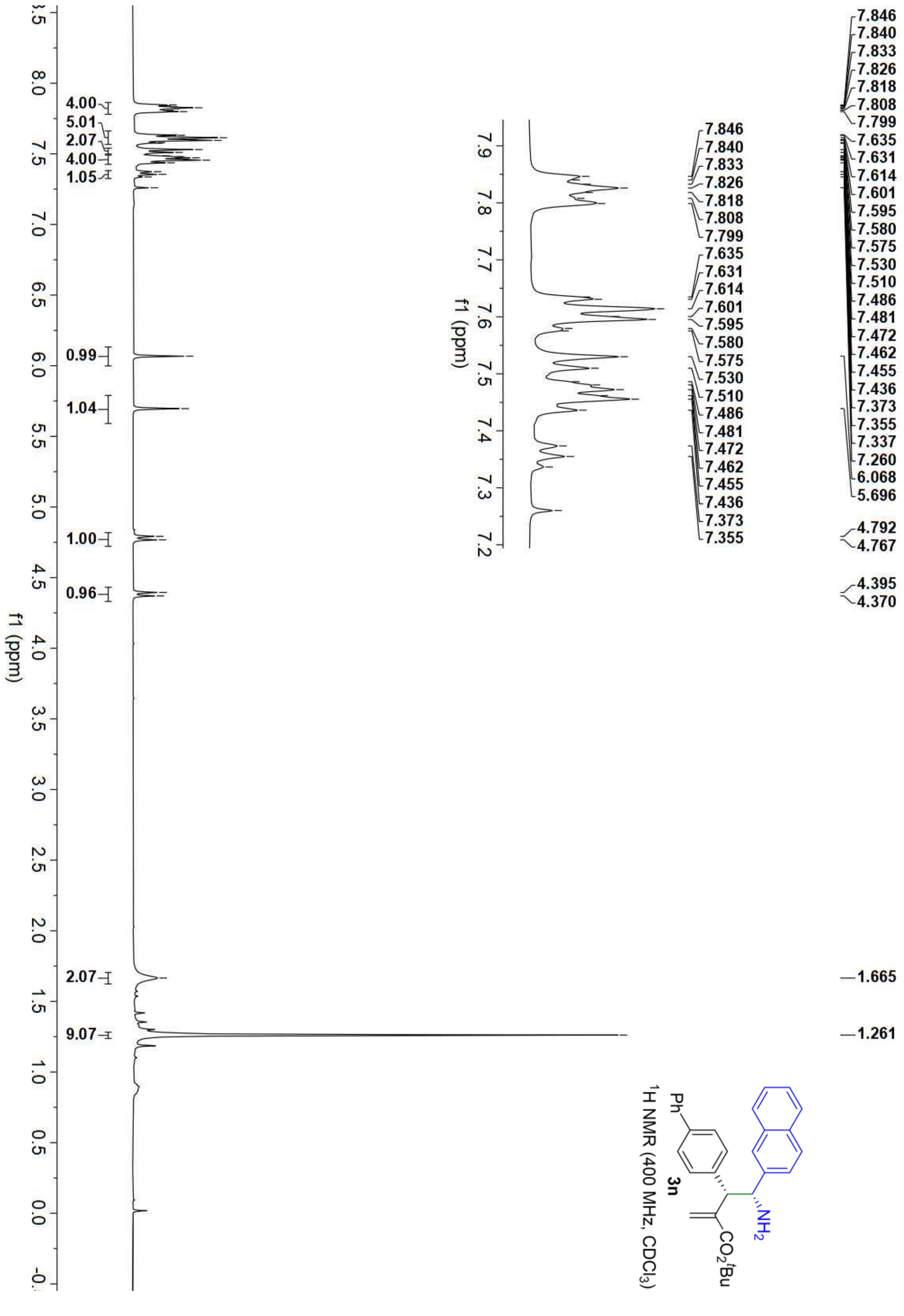
7.846
7.840
7.833
7.826
7.818
7.808
7.799
7.635
7.631
7.614
7.601
7.595
7.580
7.575
7.530
7.510
7.486
7.481
7.472
7.462
7.455
7.436
7.373
7.355
7.337
7.260
6.068
5.696
4.792
4.767
4.395
4.370
1.665
1.261
4.00
5.01
2.07
4.00
1.05
0.99
1.04
1.00
0.96
2.07
9.07
f1 (ppm)
NH2
Ph
CO2tBu
3n
1H NMR (400 MHz, CDCl3)

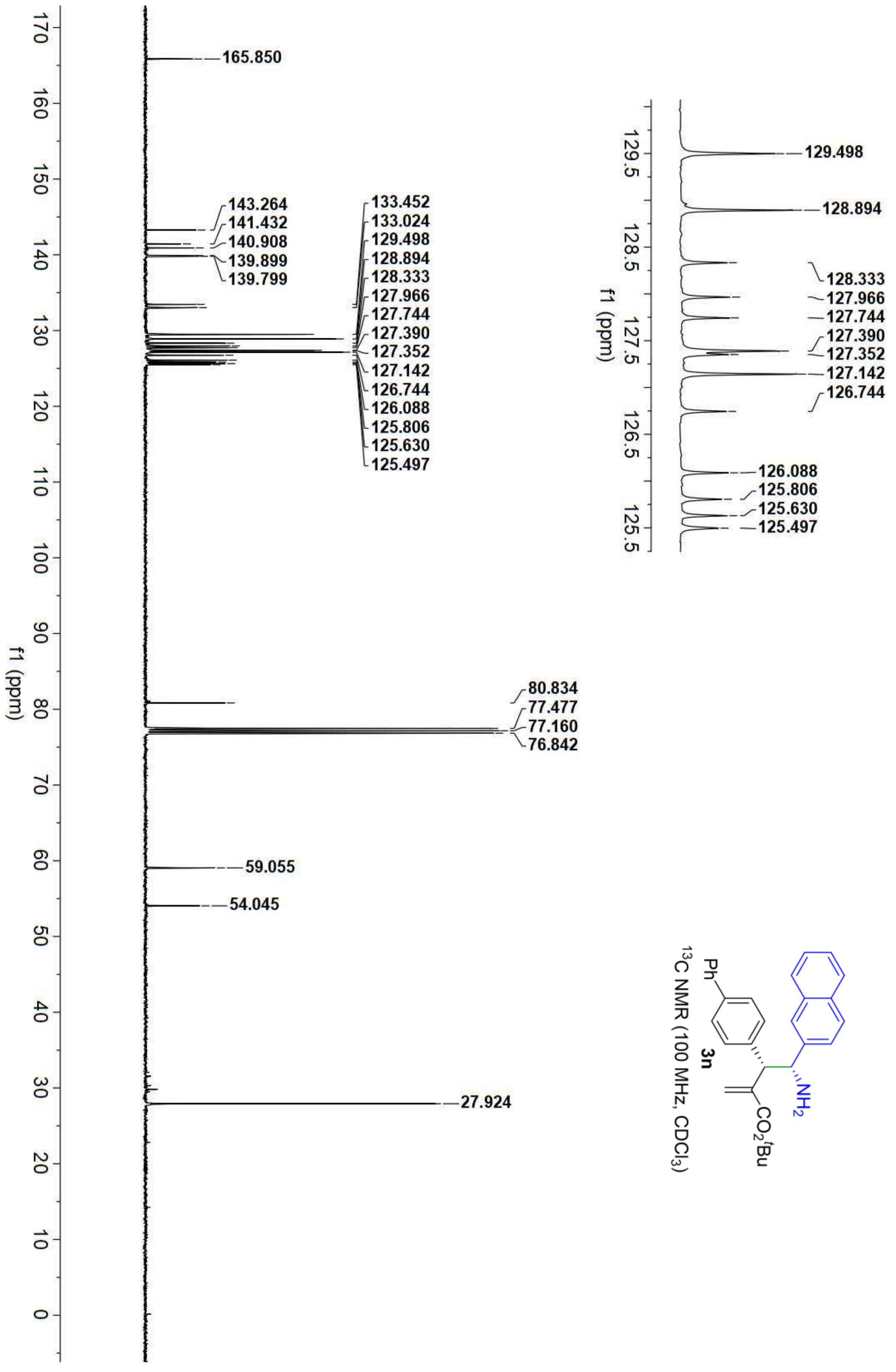
165.850
143.264
141.432
140.908
139.899
139.799
133.452
133.024
129.498
128.894
128.333
127.966
127.744
127.390
127.352
127.142
126.744
126.088
125.806
125.630
125.497
80.834
77.477
77.160
76.842
59.055
54.045
27.924
f1 (ppm)
NH2
CO2tBu
Ph
3n
13C NMR (100 MHz, CDCl3)

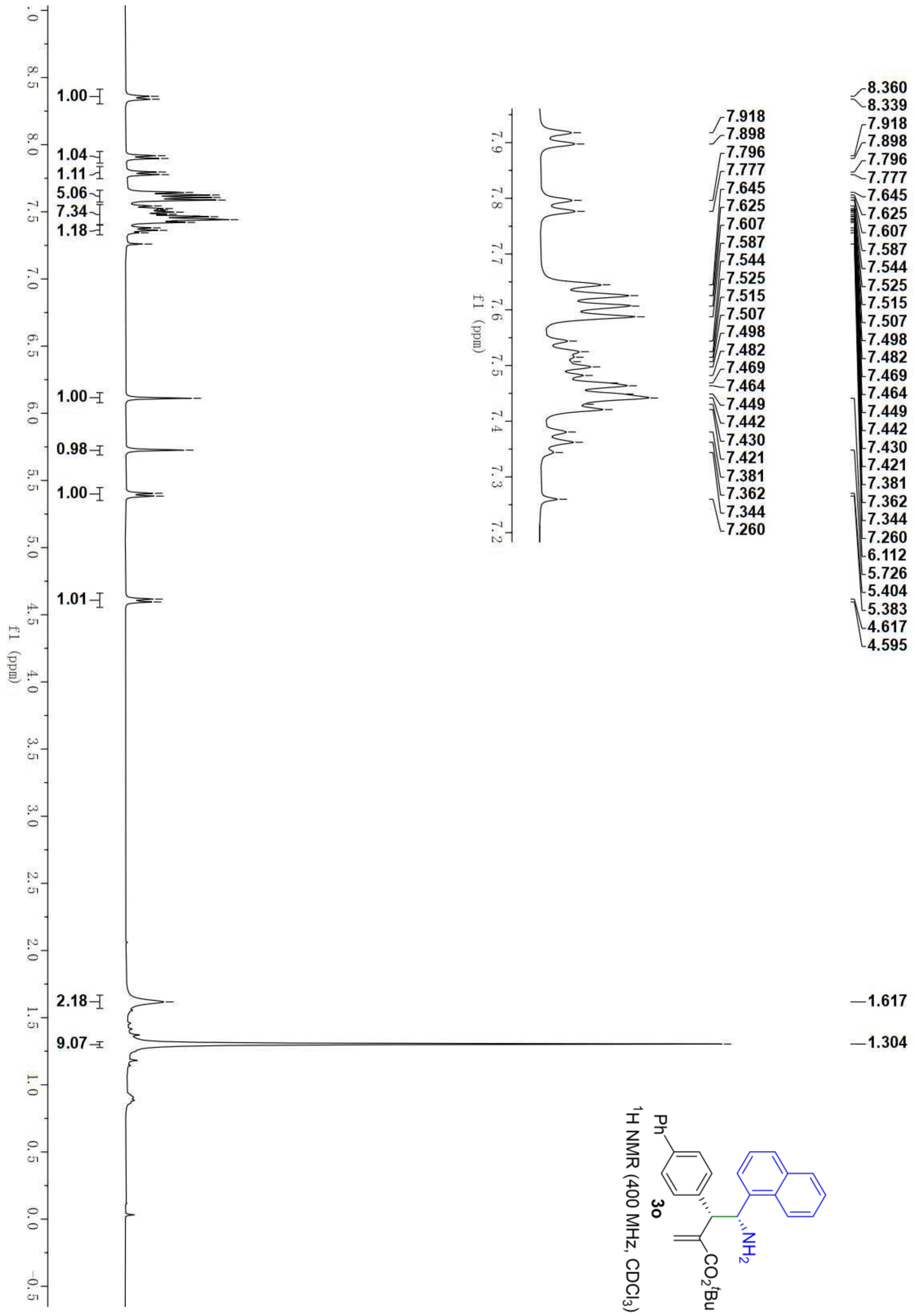
8.360
8.339
7.918
7.898
7.796
7.777
7.645
7.625
7.607
7.587
7.544
7.525
7.515
7.507
7.498
7.482
7.469
7.464
7.449
7.442
7.430
7.421
7.381
7.362
7.344
7.260
6.112
5.726
5.404
5.383
4.617
4.595
1.617
1.304
1.00
1.04
1.11
5.06
7.34
1.18
1.00
0.98
1.00
1.01
2.18
9.07
f1 (ppm)
NH2
CO2tBu
Ph
3o
1H NMR (400 MHz, CDCl3)

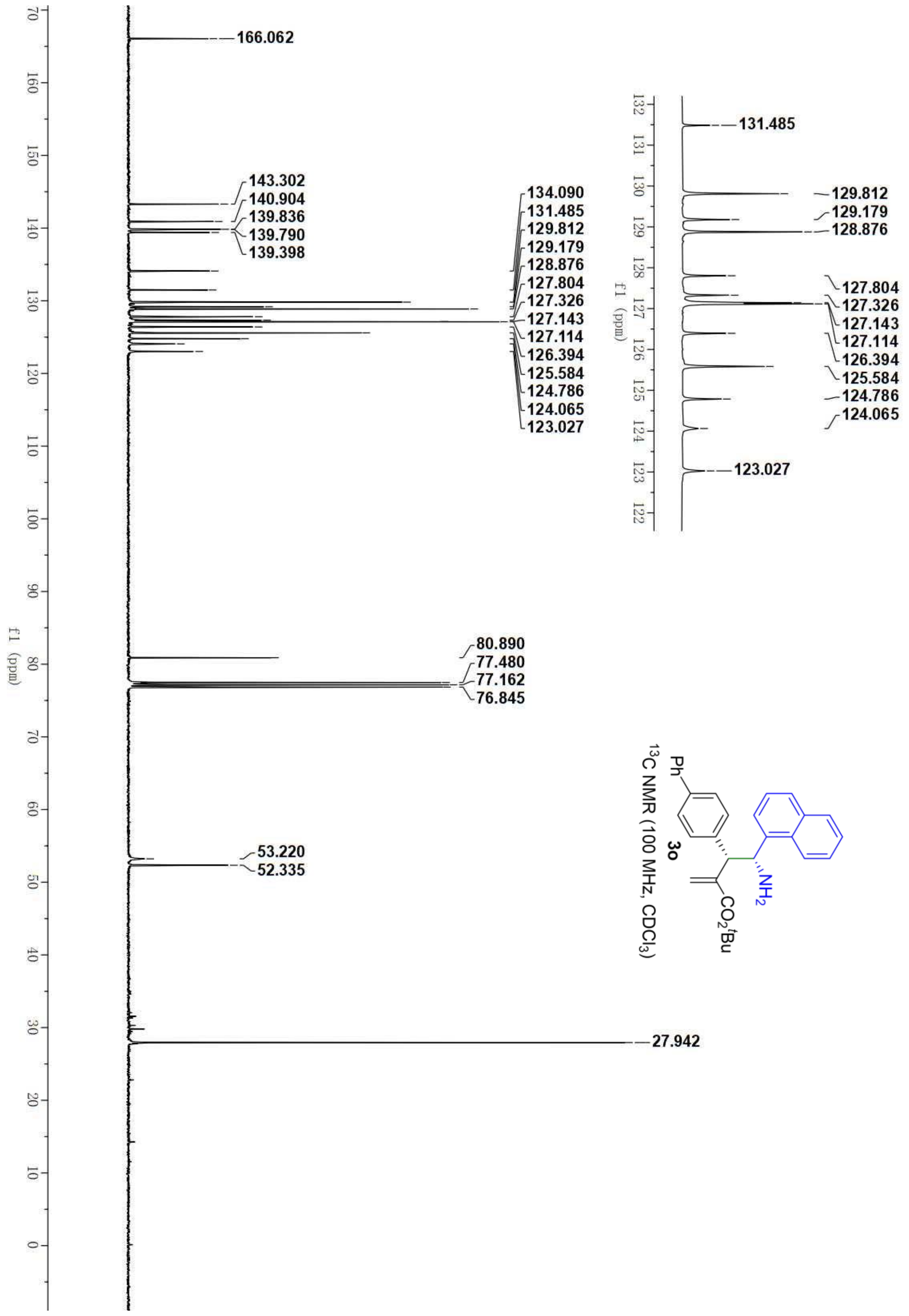
166.062
143.302
140.904
139.836
139.790
139.398
134.090
131.485
129.812
129.179
128.876
127.804
127.326
127.143
127.114
126.394
125.584
124.786
124.065
123.027
80.890
77.480
77.162
76.845
53.220
52.335
27.942
f1 (ppm)
NH2
CO2tBu
Ph
3o
13C NMR (100 MHz, CDCl3)

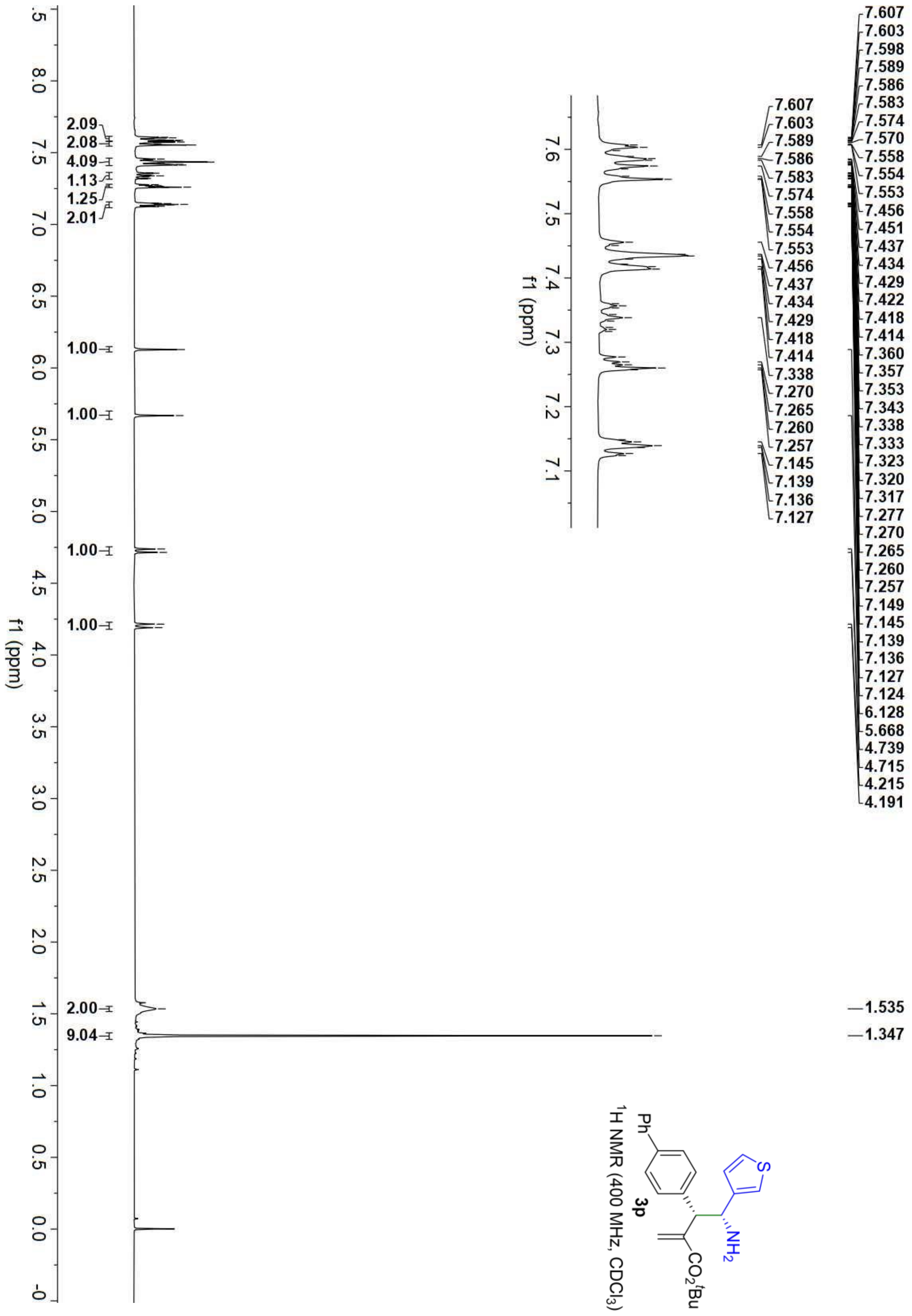
3p
1H NMR (400 MHz, CDCl3)
f1 (ppm)

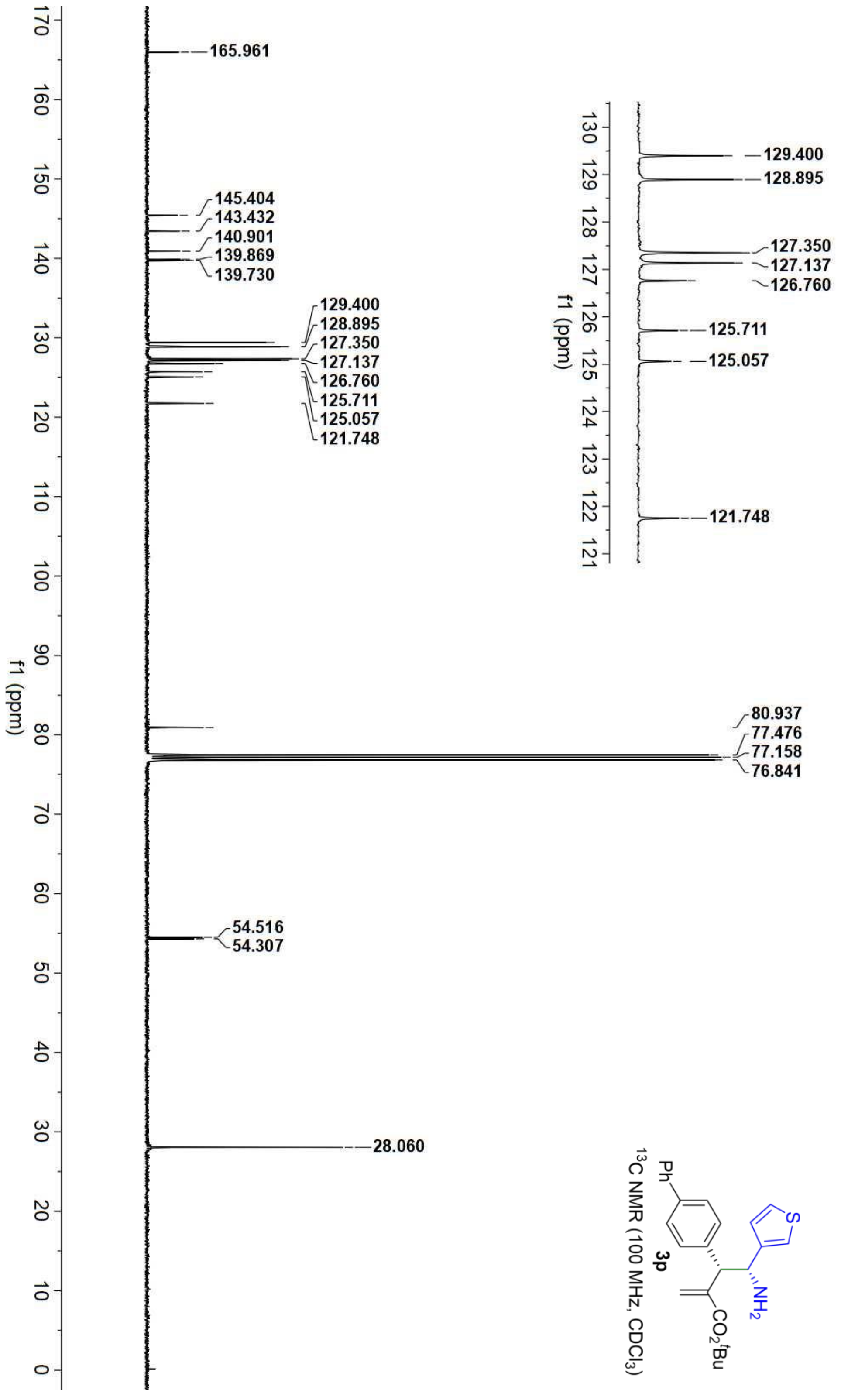
165.961
145.404
143.432
140.901
139.869
139.730
129.400
128.895
127.350
127.137
126.760
125.711
125.057
121.748
80.937
77.476
77.158
76.841
54.516
54.307
28.060
f1 (ppm)
S
NH2
CO2tBu
Ph
3p
13C NMR (100 MHz, CDCl3)

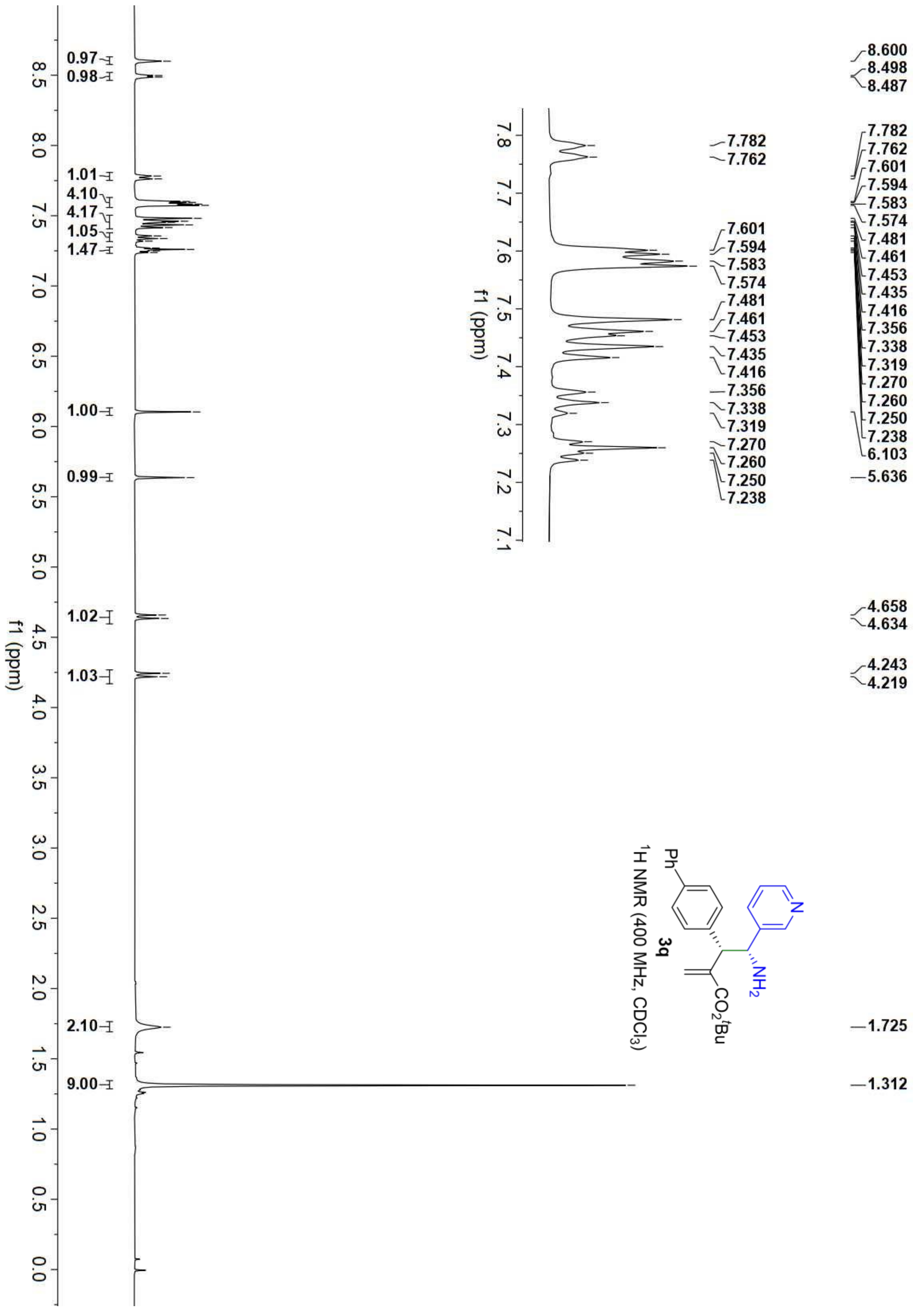

3q
1H NMR (400 MHz, CDCl3)
8.600
8.498
8.487
7.782
7.762
7.601
7.594
7.583
7.574
7.481
7.461
7.453
7.435
7.416
7.356
7.338
7.319
7.270
7.260
7.250
7.238
6.103
5.636
4.658
4.634
4.243
4.219
1.725
1.312
f1 (ppm)

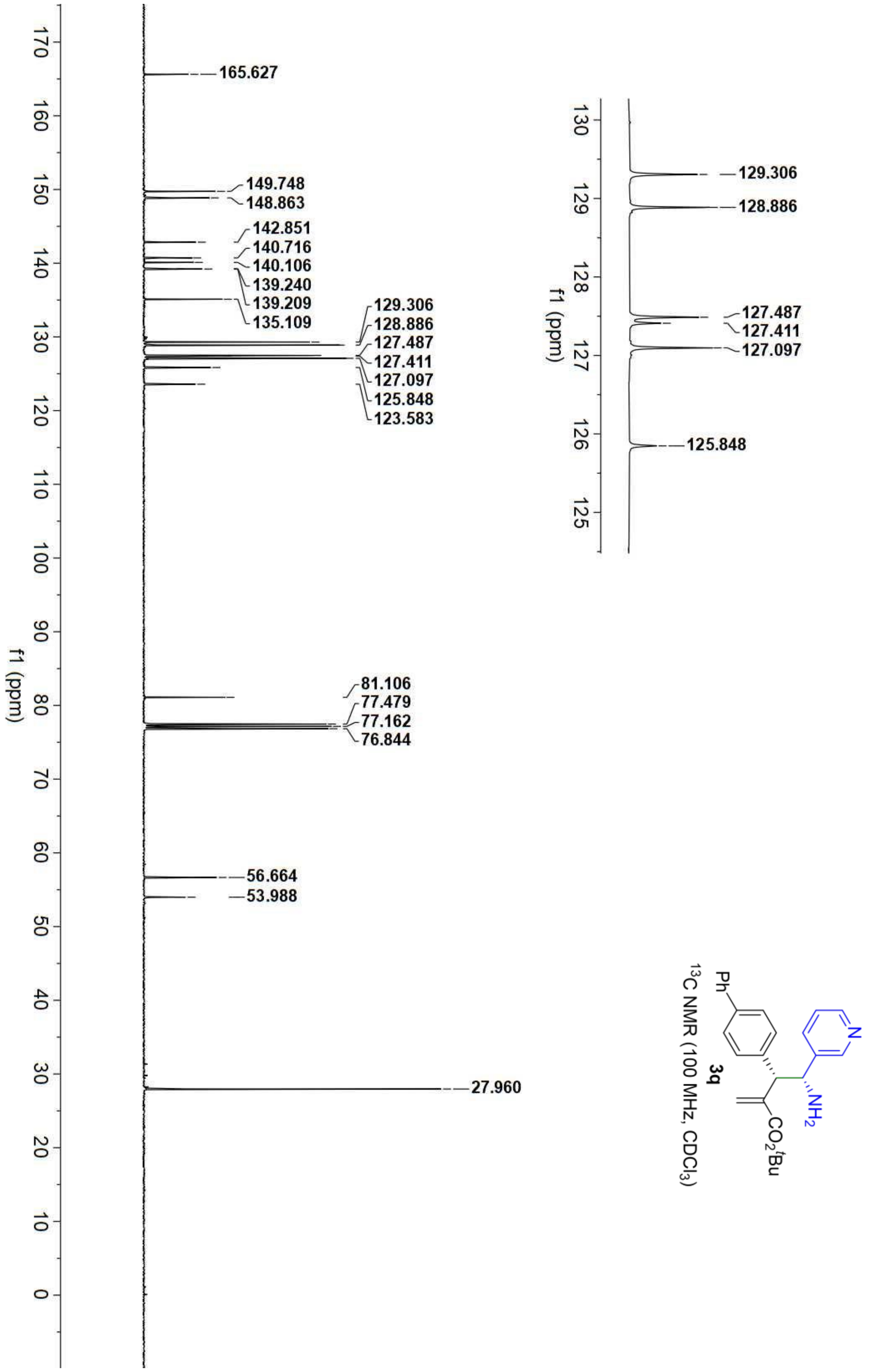

165.627
149.748
148.863
142.851
140.716
140.106
139.240
139.209
135.109
129.306
128.886
127.487
127.411
127.097
125.848
123.583
81.106
77.479
77.162
76.844
56.664
53.988
27.960
f1 (ppm)
129.306
128.886
127.487
127.411
127.097
125.848
f1 (ppm)
Ph
N
NH2
CO2tBu
3q
13C NMR (100 MHz, CDCl3)

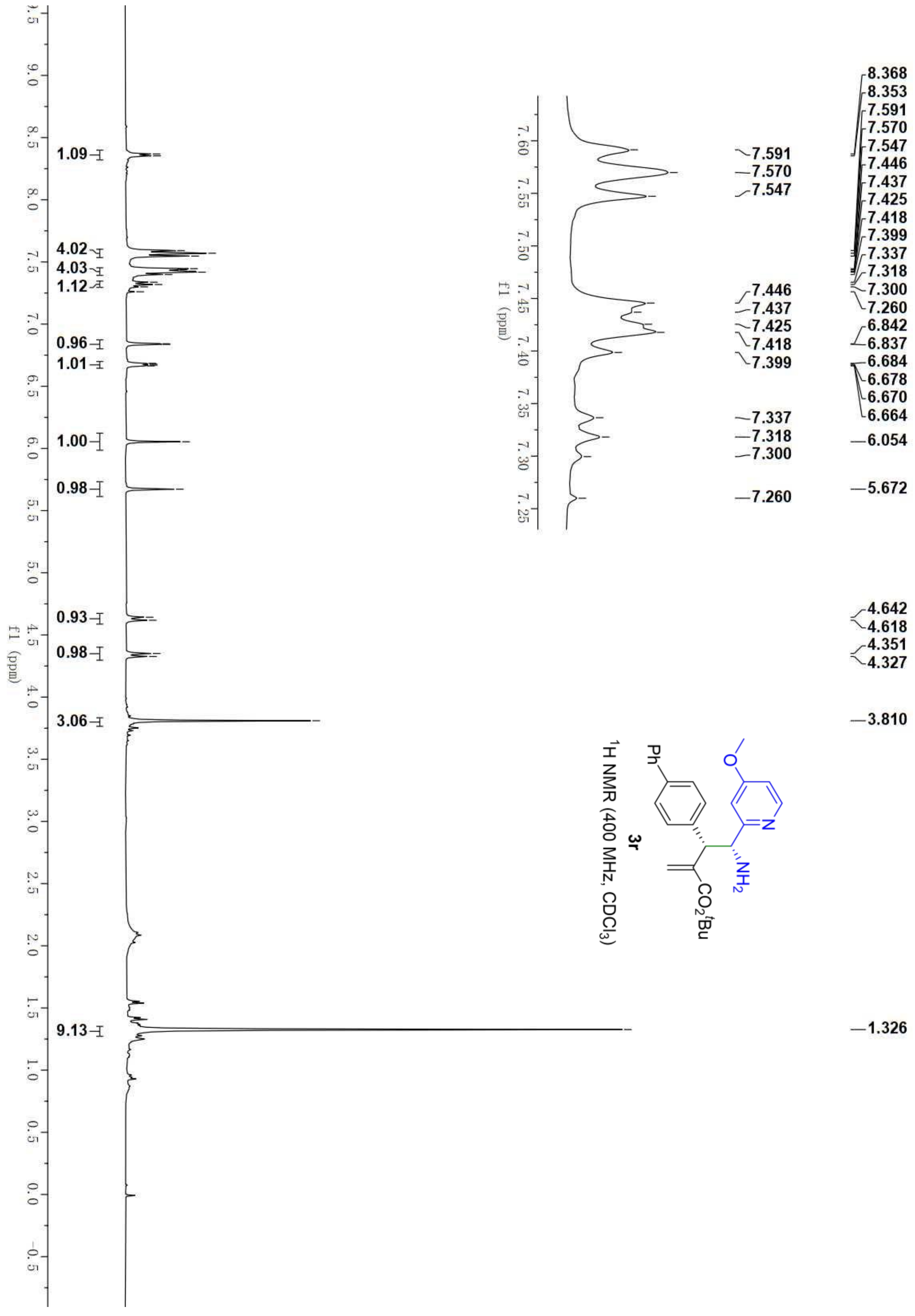
8.368
8.353
7.591
7.570
7.547
7.446
7.437
7.425
7.418
7.399
7.337
7.318
7.300
7.260
6.842
6.837
6.684
6.678
6.670
6.664
6.054
5.672
4.642
4.618
4.351
4.327
3.810
1.326
1.09
4.02
4.03
1.12
0.96
1.01
1.00
0.98
0.93
0.98
3.06
9.13
f1 (ppm)
Ph
O
N
NH2
CO2tBu
3r
1H NMR (400 MHz, CDCl3)

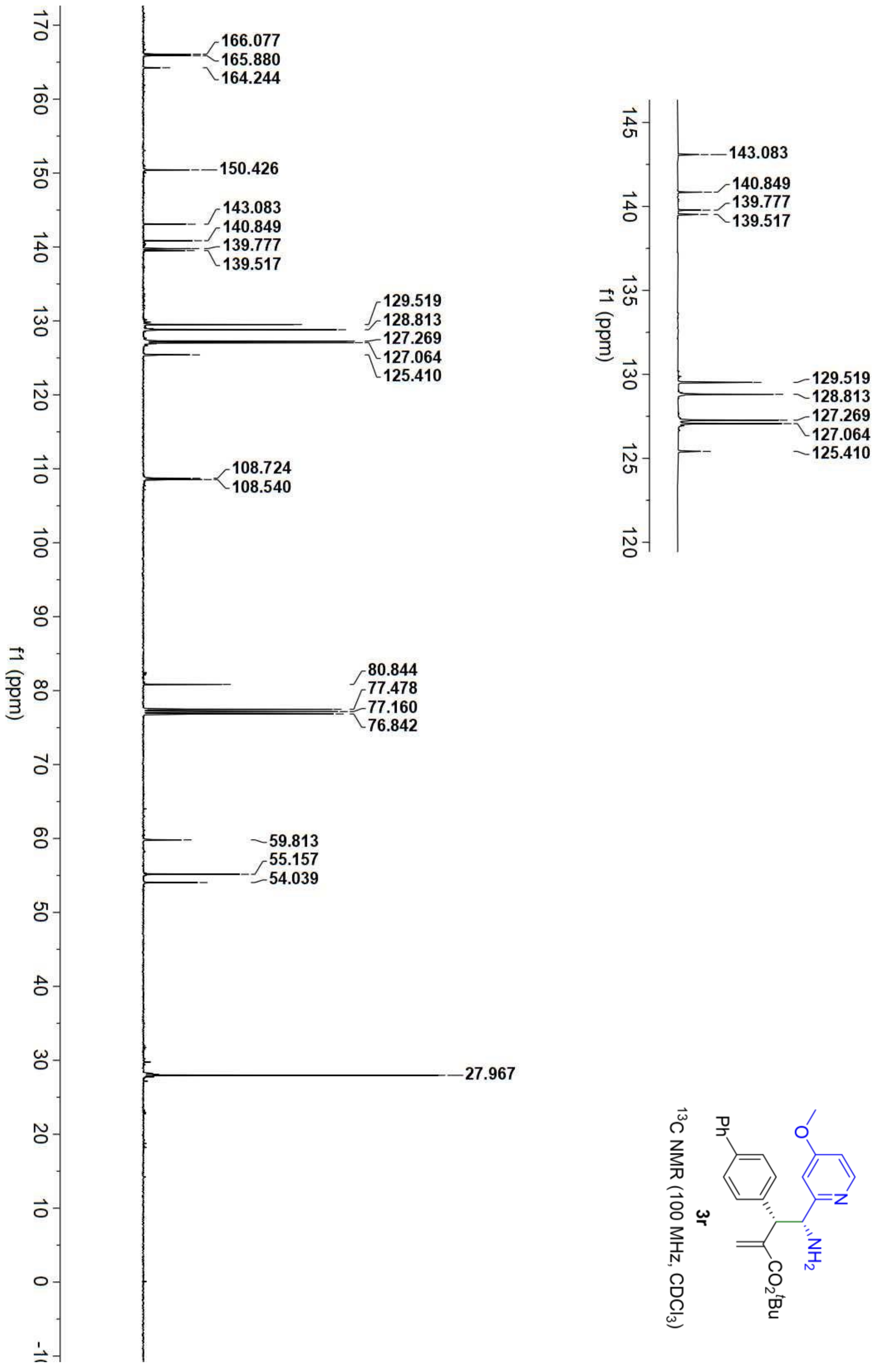
166.077
165.880
164.244
150.426
143.083
140.849
139.777
139.517
129.519
128.813
127.269
127.064
125.410
108.724
108.540
80.844
77.478
77.160
76.842
59.813
55.157
54.039
27.967
f1 (ppm)
143.083
140.849
139.777
139.517
129.519
128.813
127.269
127.064
125.410
f1 (ppm)
NH2
N
O
Ph
CO2tBu
3r
13C NMR (100 MHz, CDCl3)

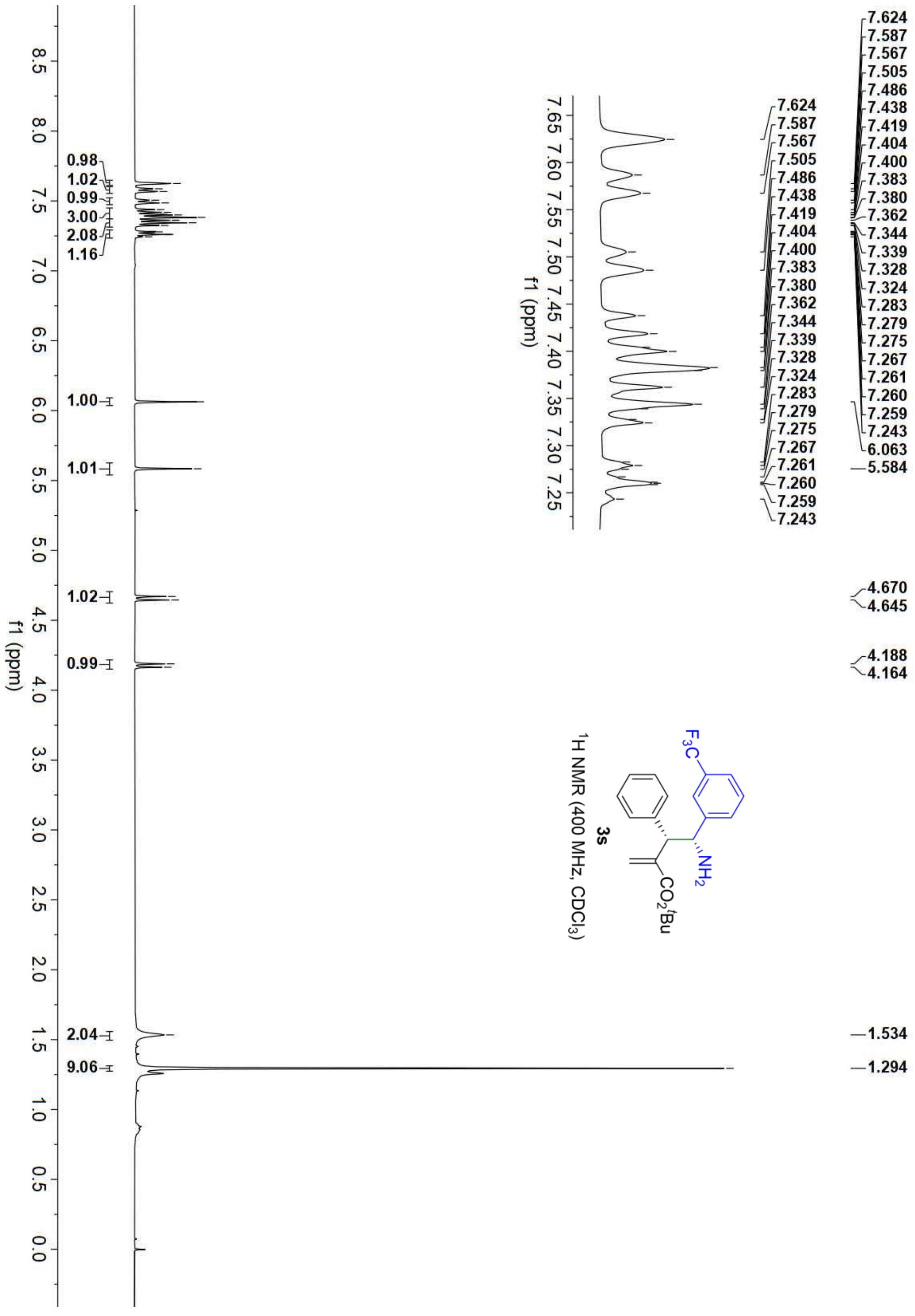
7.624
7.587
7.567
7.505
7.486
7.438
7.419
7.404
7.400
7.383
7.380
7.362
7.344
7.339
7.328
7.324
7.283
7.279
7.275
7.267
7.261
7.260
7.259
7.243
6.063
5.584
4.670
4.645
4.188
4.164
1.534
1.294
F3C
NH2
CO2tBu
3s
1H NMR (400 MHz, CDCl3)
f1 (ppm)
0.98
1.02
0.99
3.00
2.08
1.16
1.00
1.01
1.02
0.99
2.04
9.06

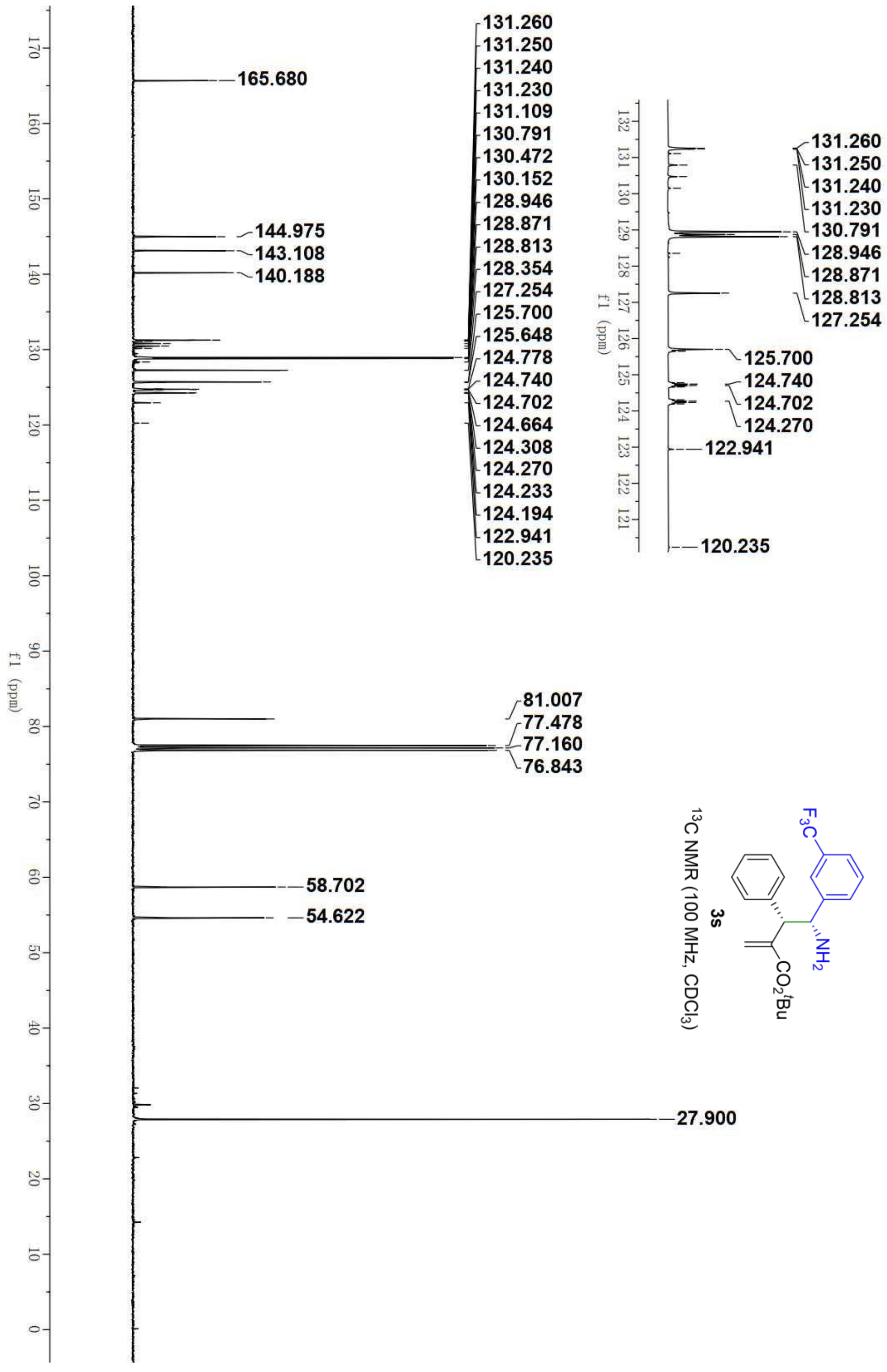
165.680
144.975
143.108
140.188
131.260
131.250
131.240
131.230
131.109
130.791
130.472
130.152
128.946
128.871
128.813
128.354
127.254
125.700
125.648
124.778
124.740
124.702
124.664
124.308
124.270
124.233
124.194
122.941
120.235
81.007
77.478
77.160
76.843
58.702
54.622
27.900
f1 (ppm)
F_3C
NH_2
$CO_2{}^tBu$
3s
^{13}C NMR (100 MHz, $CDCl_3$)

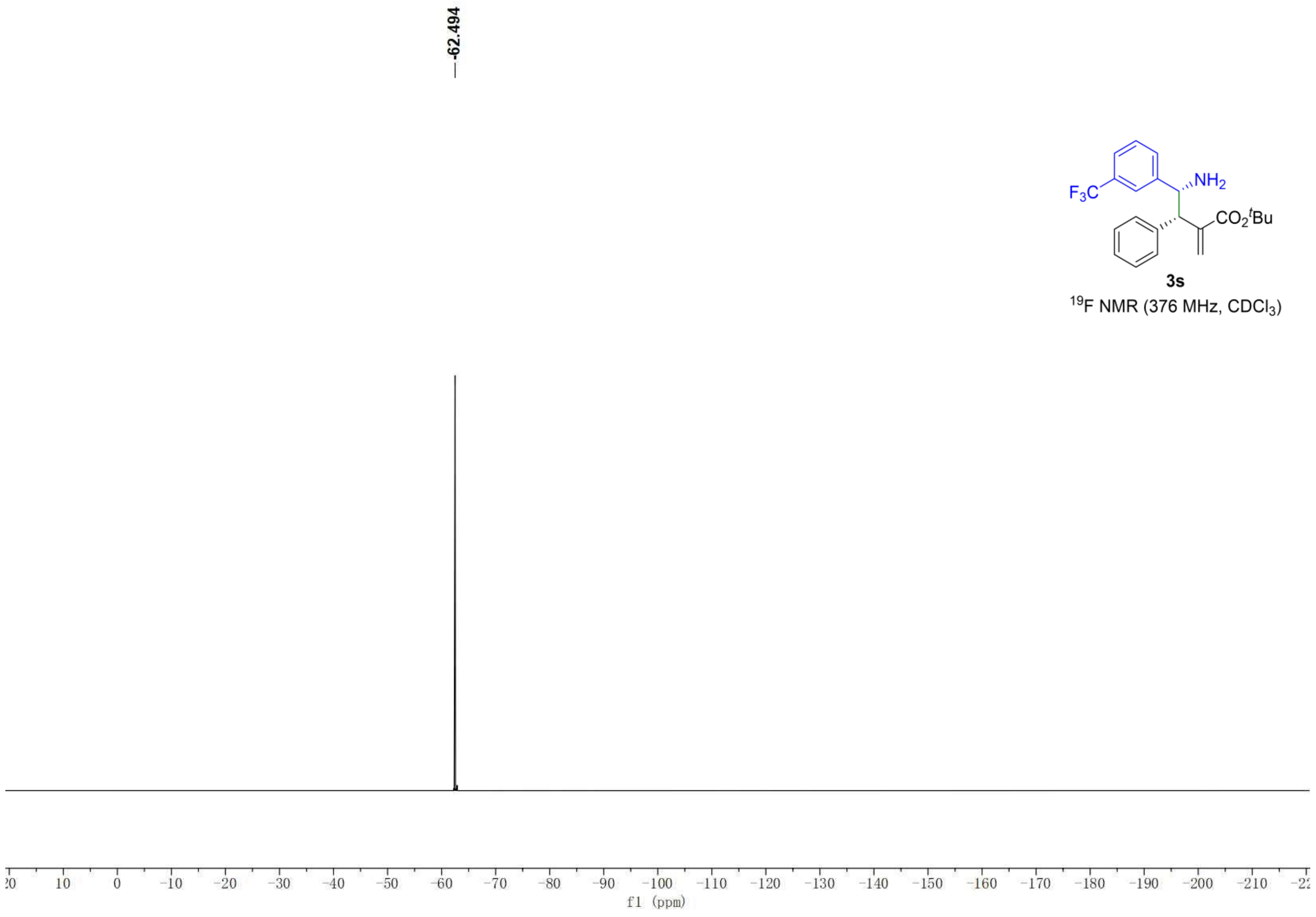

3s

^{19}F NMR (376 MHz, $CDCl_3$)

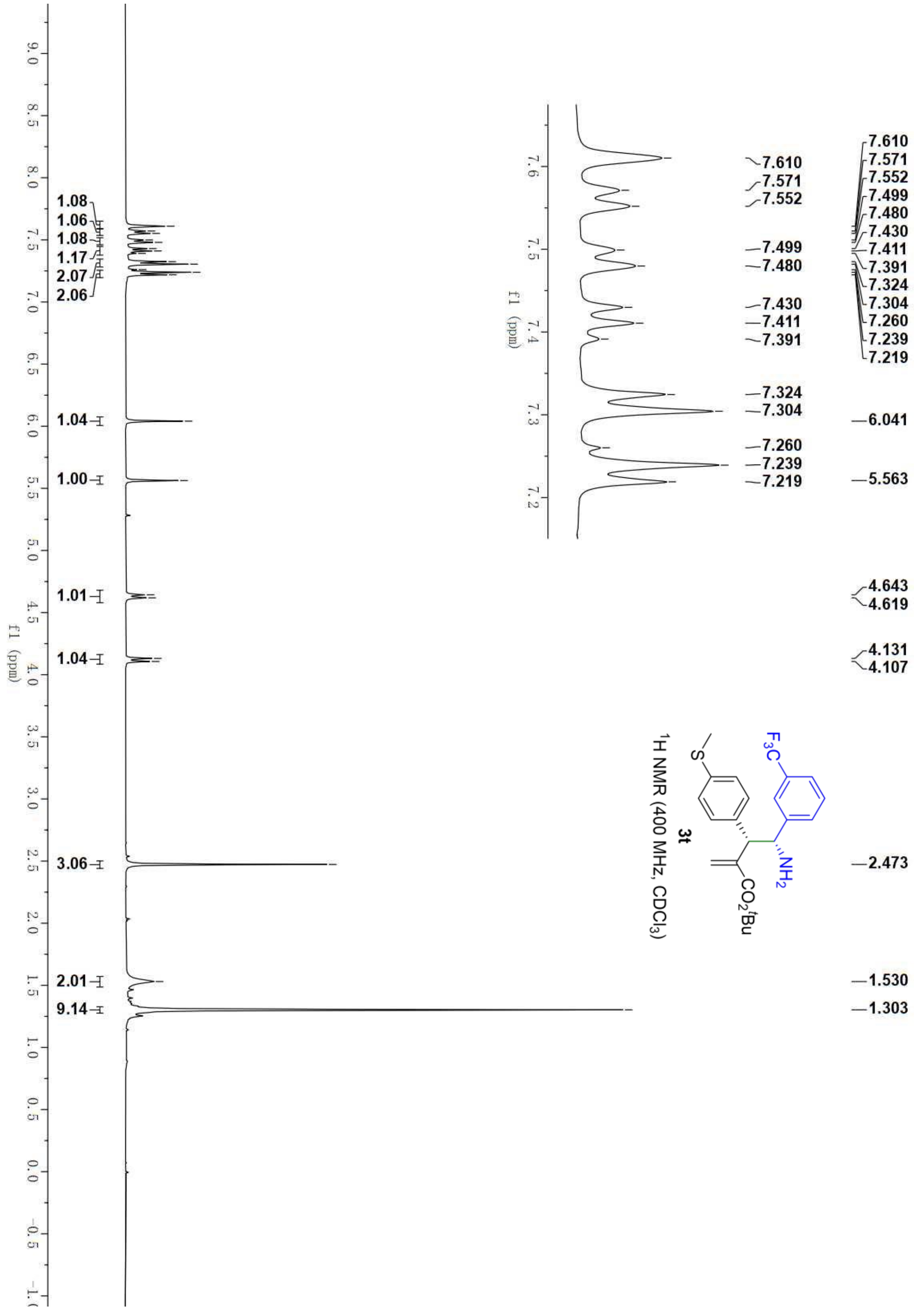

7.610
7.571
7.552
7.499
7.480
7.430
7.411
7.391
7.324
7.304
7.260
7.239
7.219
6.041
5.563
4.643
4.619
4.131
4.107
2.473
1.530
1.303
1.08
1.06
1.08
1.17
2.07
2.06
1.04
1.00
1.01
1.04
3.06
2.01
9.14
f1 (ppm)
F3C
NH2
CO2tBu
S
3t
1H NMR (400 MHz, CDCl3)

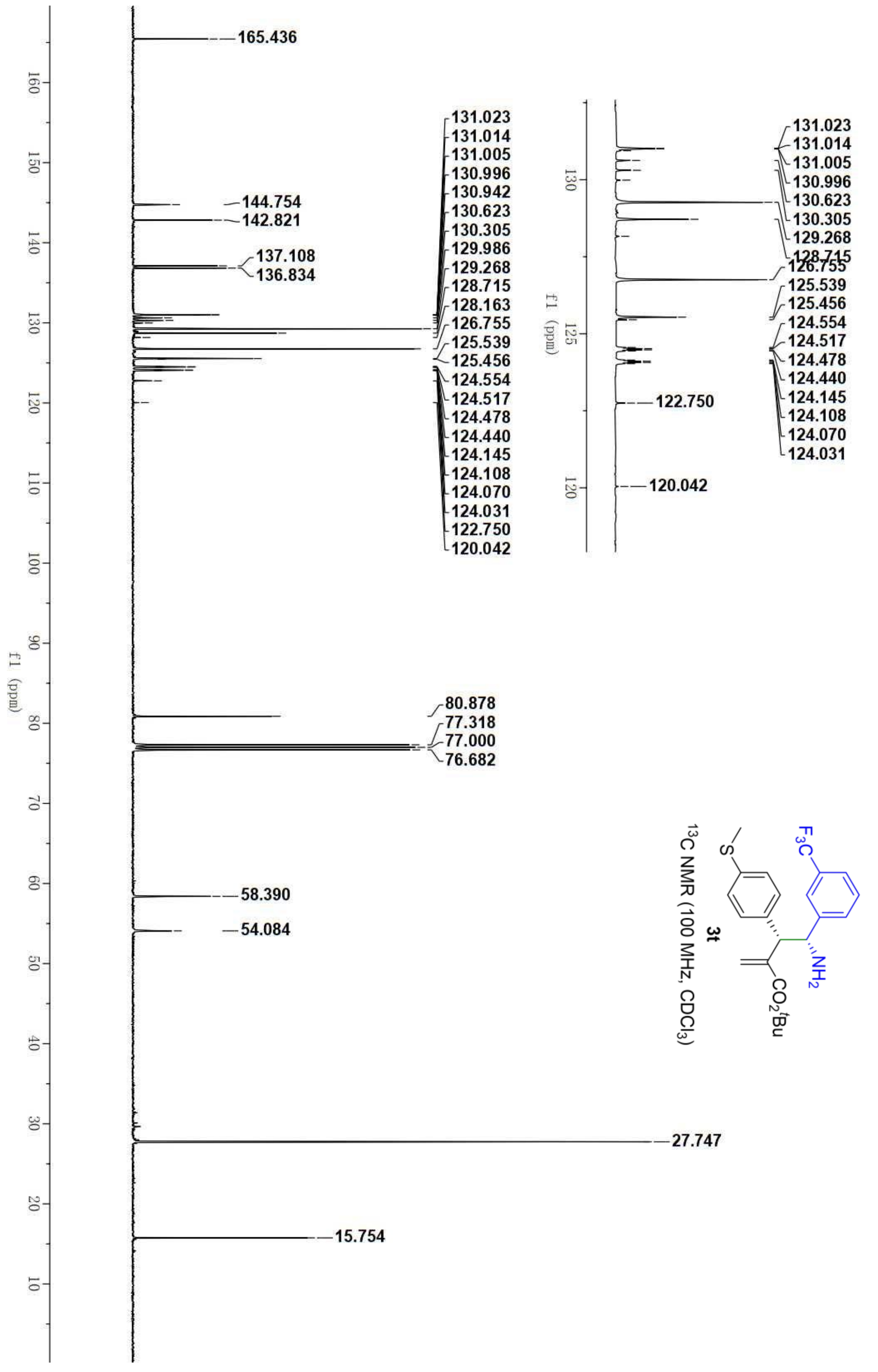
165.436
144.754
142.821
137.108
136.834
131.023
131.014
131.005
130.996
130.942
130.623
130.305
129.986
129.268
128.715
128.163
126.755
125.539
125.456
124.554
124.517
124.478
124.440
124.145
124.108
124.070
124.031
122.750
120.042
80.878
77.318
77.000
76.682
58.390
54.084
27.747
15.754
f1 (ppm)
3t
13C NMR (100 MHz, CDCl3)

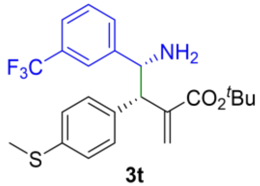

3t

^{19}F NMR (376 MHz, $CDCl_3$)

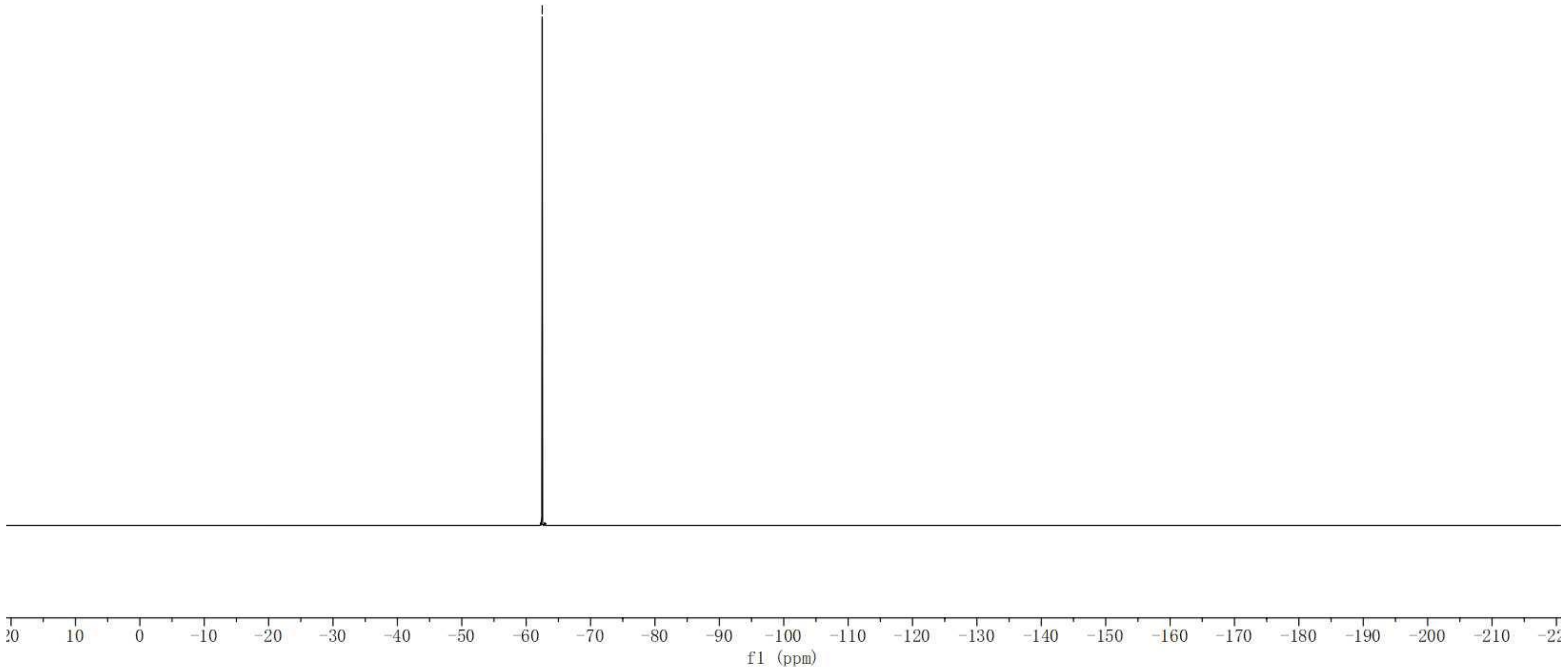

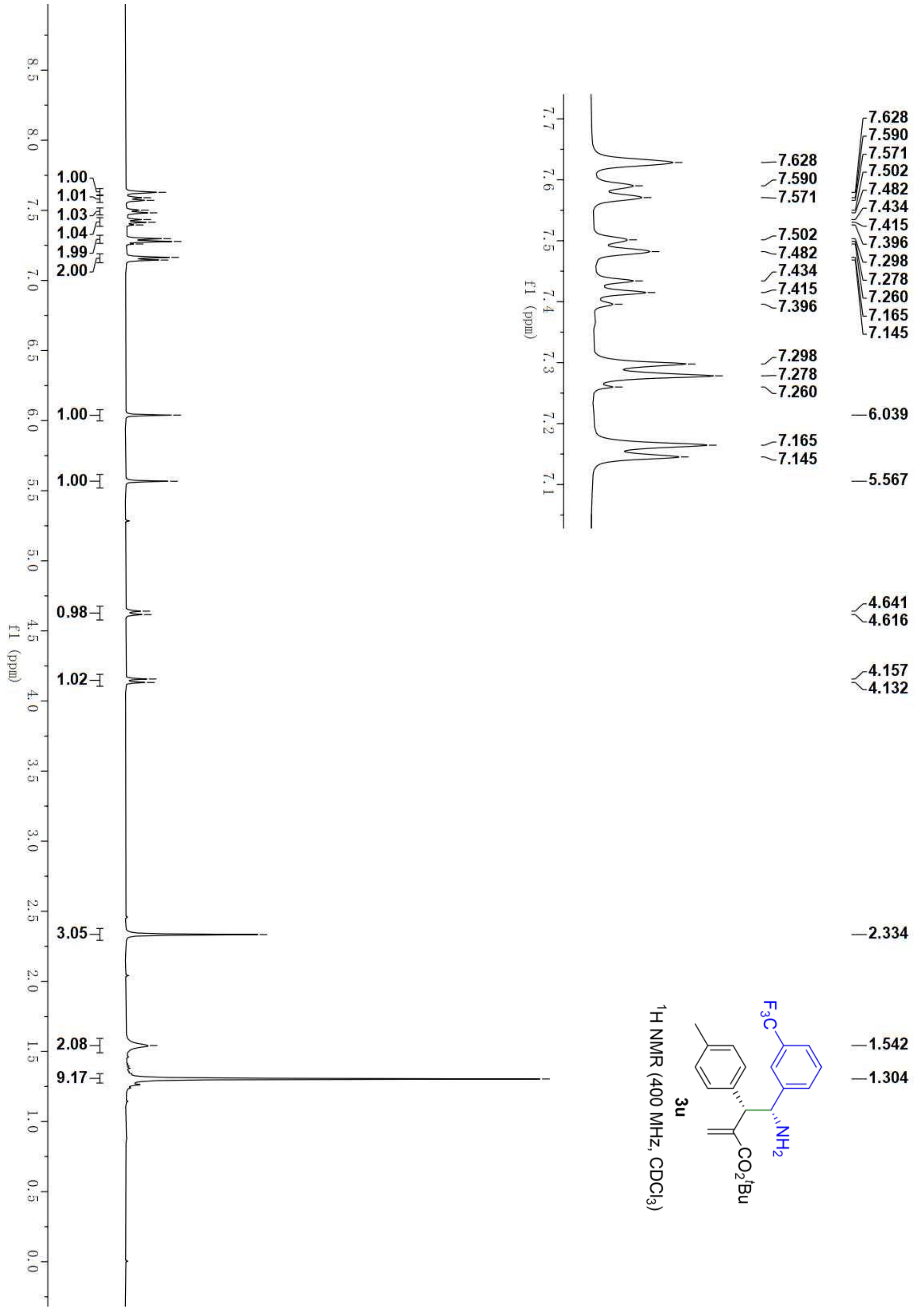

7.628
7.590
7.571
7.502
7.482
7.434
7.415
7.396
7.298
7.278
7.260
7.165
7.145
6.039
5.567
4.641
4.616
4.157
4.132
2.334
1.542
1.304
1.00
1.01
1.03
1.04
1.99
2.00
1.00
1.00
0.98
1.02
3.05
2.08
9.17
f1 (ppm)
F3C
NH2
CO2tBu
3u
1H NMR (400 MHz, CDCl3)

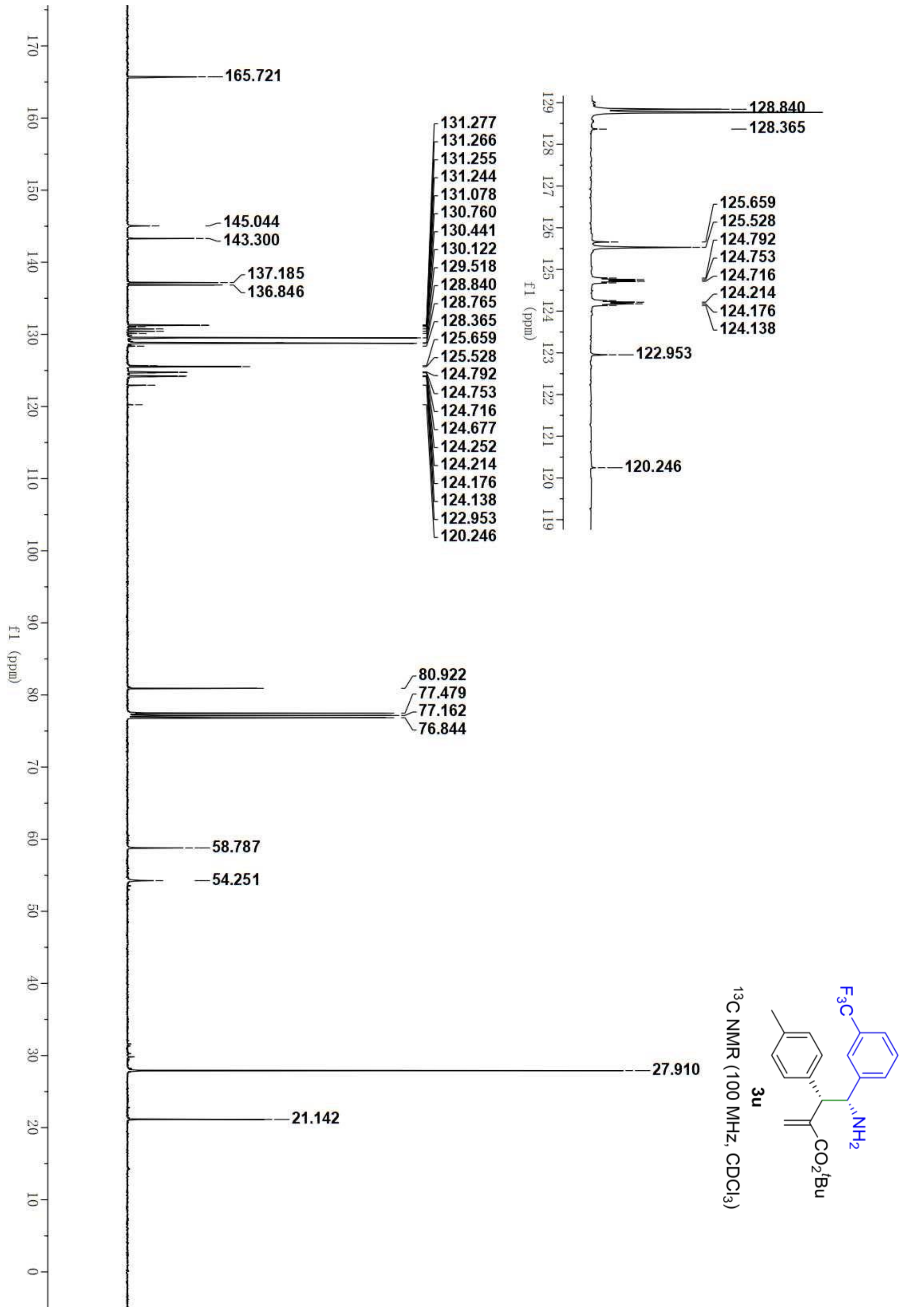
165.721
145.044
143.300
137.185
136.846
131.277
131.266
131.255
131.244
131.078
130.760
130.441
130.122
129.518
128.840
128.765
128.365
125.659
125.528
124.792
124.753
124.716
124.677
124.252
124.214
124.176
124.138
122.953
120.246
80.922
77.479
77.162
76.844
58.787
54.251
27.910
21.142
f1 (ppm)
F3C
NH2
CO2tBu
3u
13C NMR (100 MHz, CDCl3)

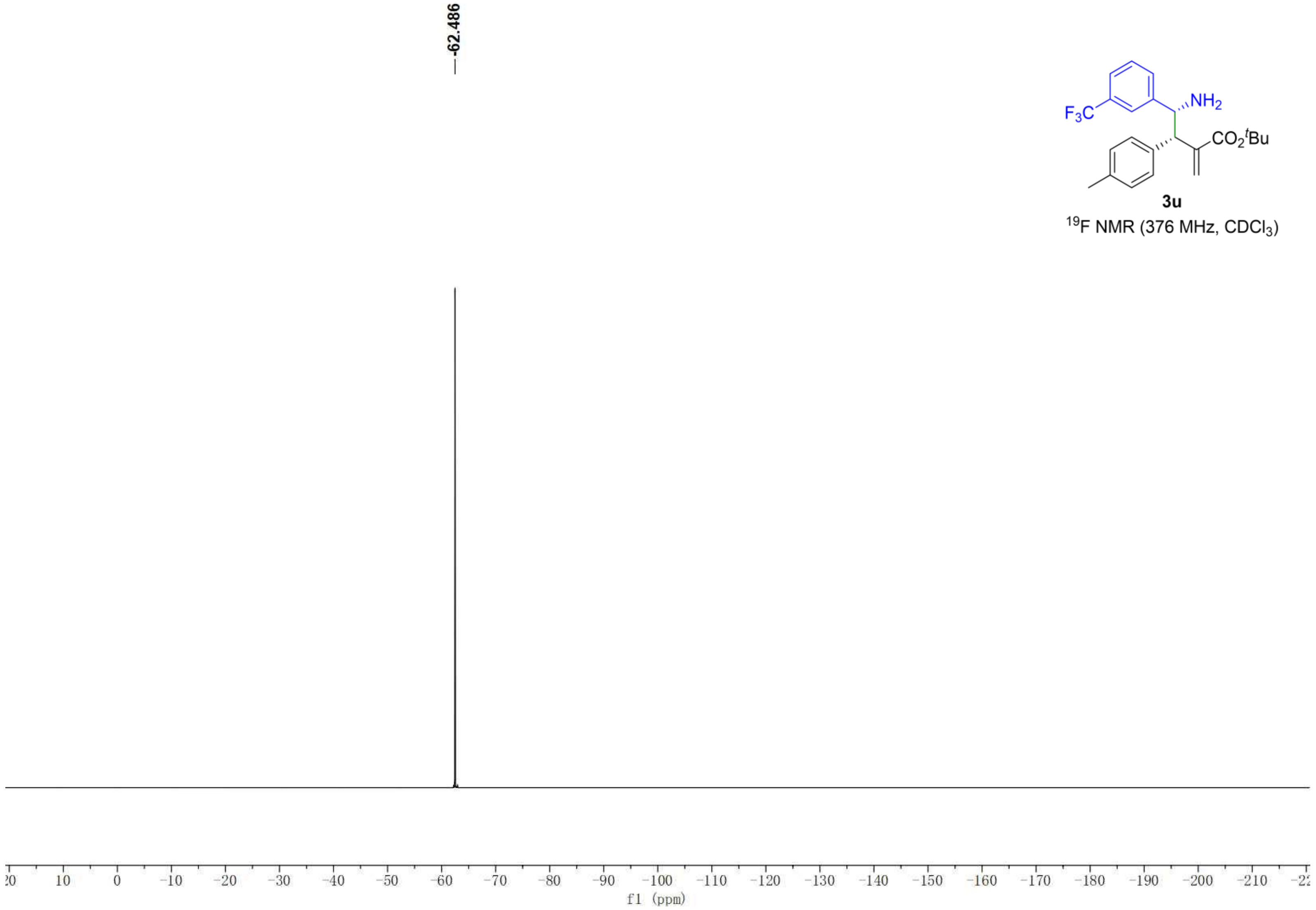

-62.486
F3C
NH2
CO2tBu
3u
19F NMR (376 MHz, CDCl3)
20 10 0 -10 -20 -30 -40 -50 -60 -70 -80 -90 -100 -110 -120 -130 -140 -150 -160 -170 -180 -190 -200 -210
f1 (ppm)

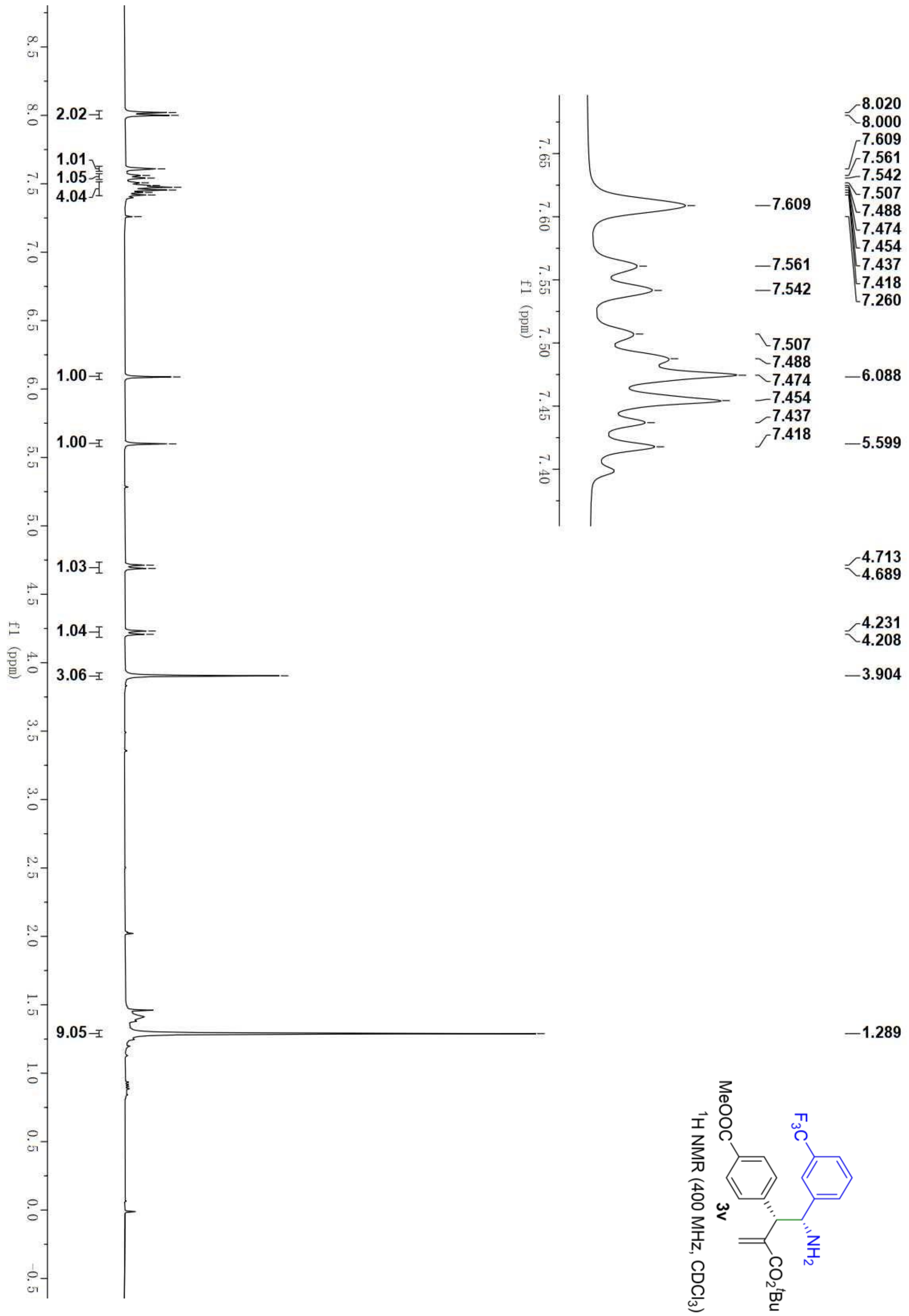
3v
^{1}H NMR (400 MHz, $CDCl_3$)

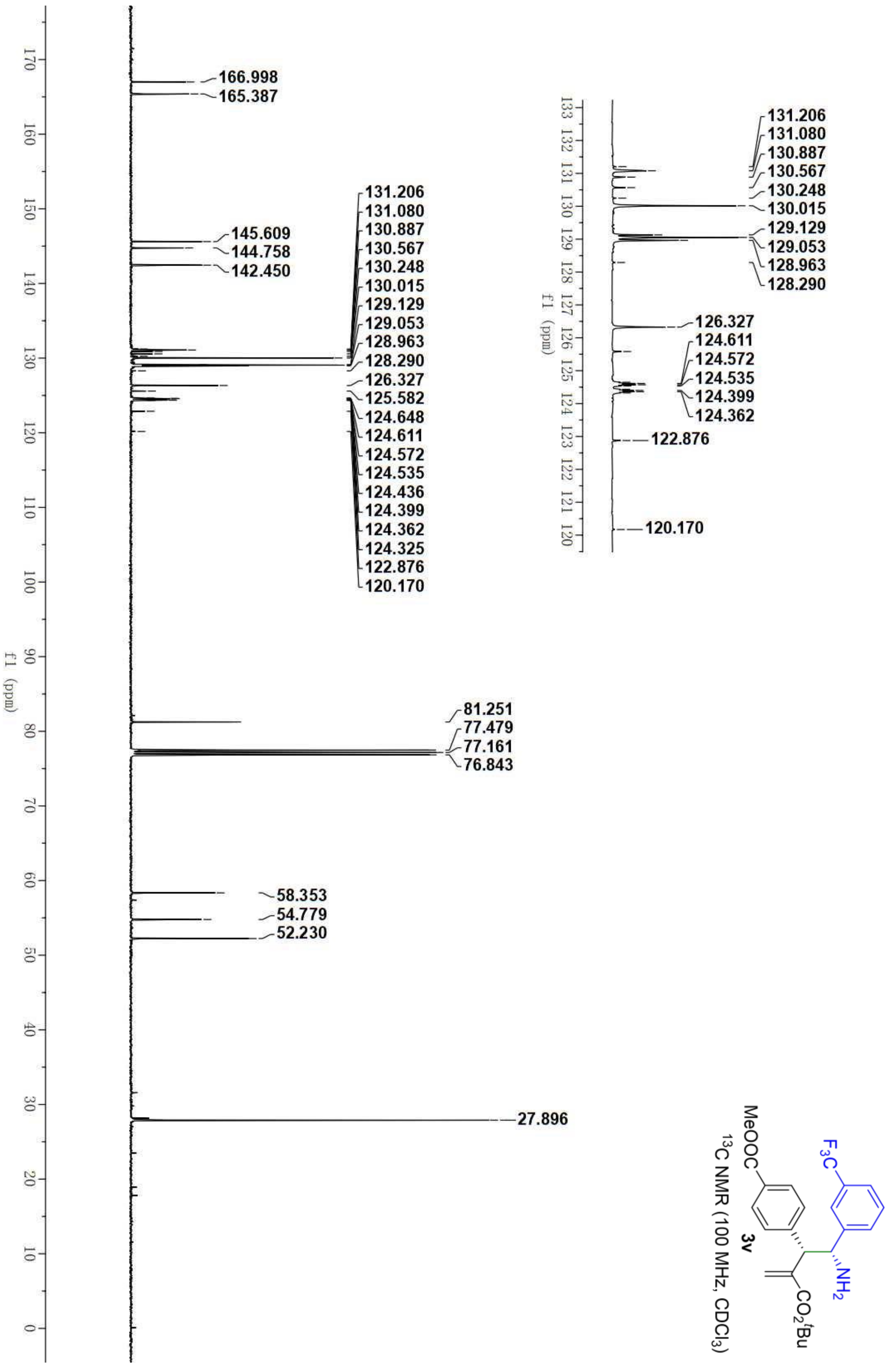
166.998
165.387
145.609
144.758
142.450
131.206
131.080
130.887
130.567
130.248
130.015
129.129
129.053
128.963
128.290
126.327
125.582
124.648
124.611
124.572
124.535
124.436
124.399
124.362
124.325
122.876
120.170
81.251
77.479
77.161
76.843
58.353
54.779
52.230
27.896
f1 (ppm)
F3C
NH2
MeOOC
CO2tBu
3v
13C NMR (100 MHz, CDCl3)

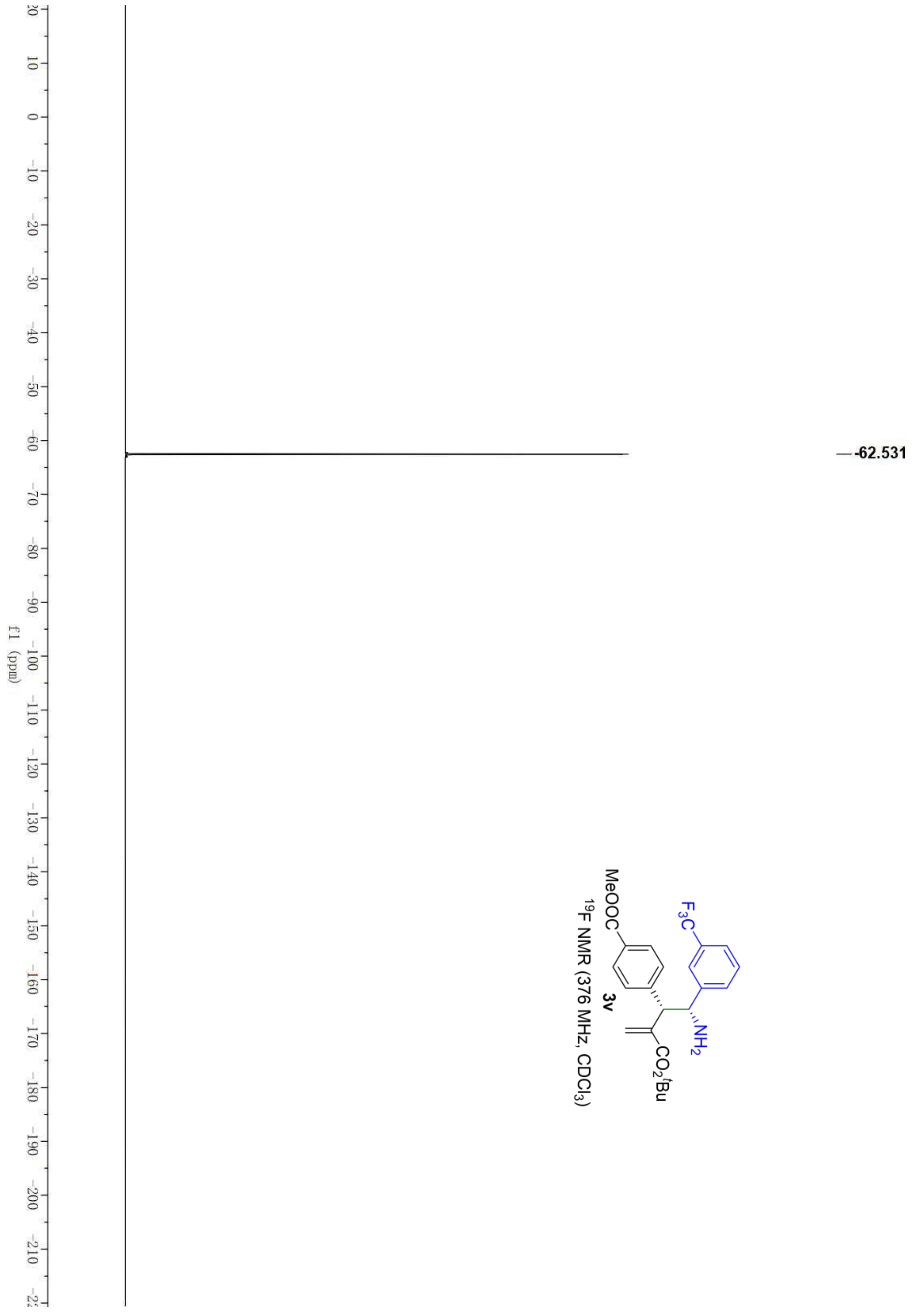
-62.531
f1 (ppm)
F3C
NH2
MeOOC
CO2tBu
3v
19F NMR (376 MHz, CDCl3)

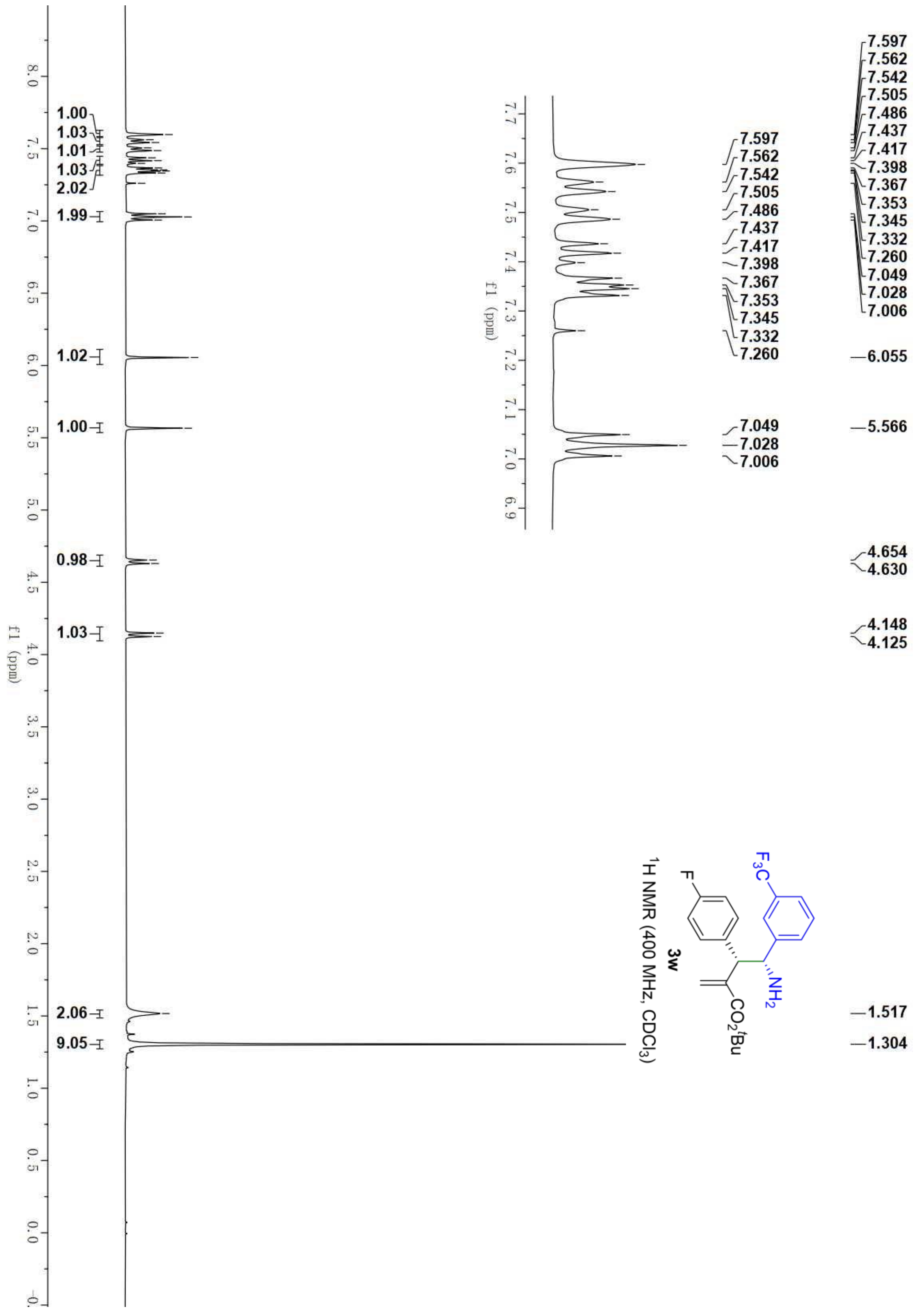

7.597
7.562
7.542
7.505
7.486
7.437
7.417
7.398
7.367
7.353
7.345
7.332
7.260
7.049
7.028
7.006
6.055
5.566
4.654
4.630
4.148
4.125
1.517
1.304
1.00
1.03
1.01
1.03
2.02
1.99
1.02
1.00
0.98
1.03
2.06
9.05
f1 (ppm)
F3C
NH2
F
CO2tBu
3w
1H NMR (400 MHz, CDCl3)

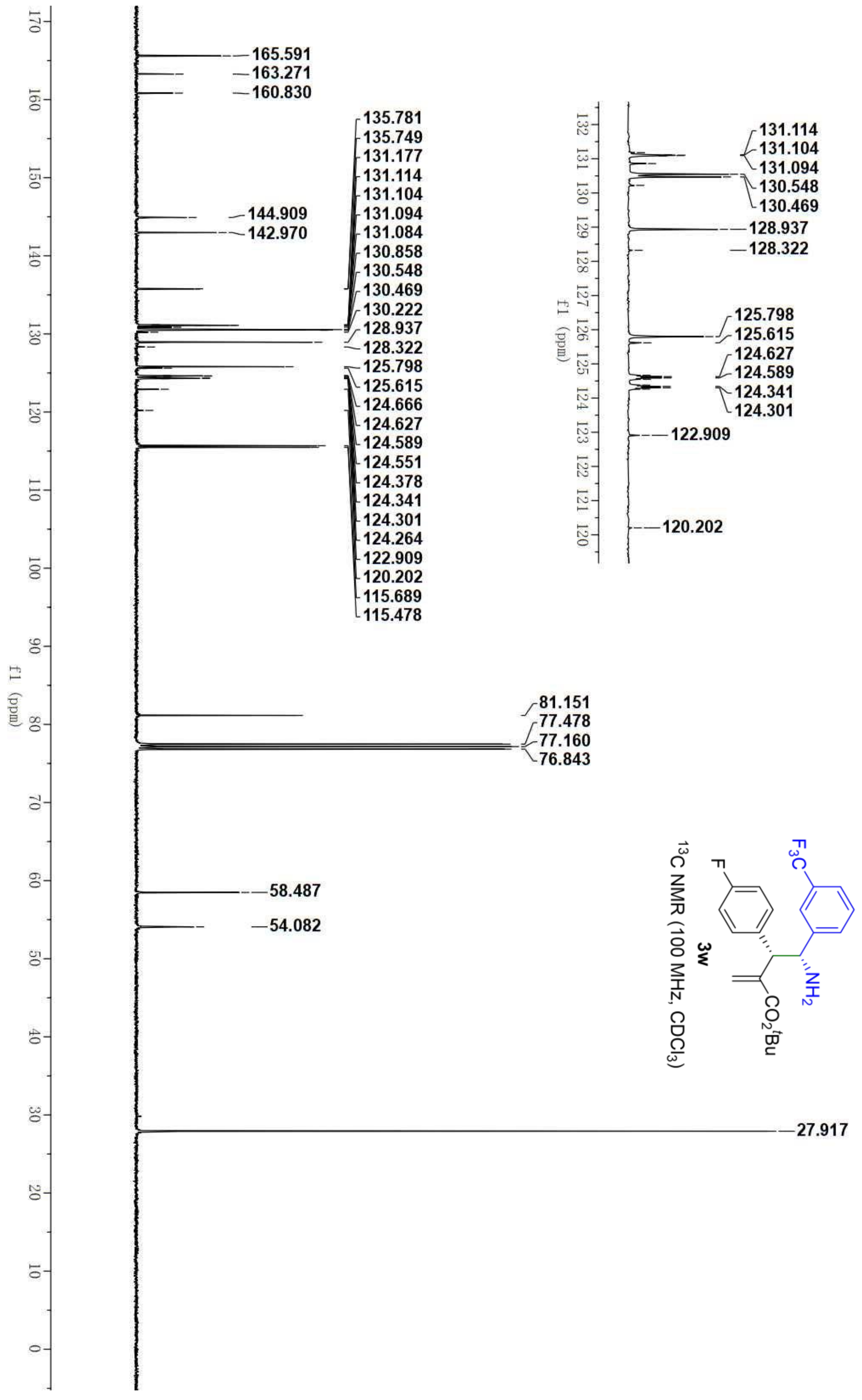

165.591
163.271
160.830
144.909
142.970
135.781
135.749
131.177
131.114
131.104
131.094
131.084
130.858
130.548
130.469
130.222
128.937
128.322
125.798
125.615
124.666
124.627
124.589
124.551
124.378
124.341
124.301
124.264
122.909
120.202
115.689
115.478
81.151
77.478
77.160
76.843
58.487
54.082
27.917
f1 (ppm)
131.114
131.104
131.094
130.548
130.469
128.937
128.322
125.798
125.615
124.627
124.589
124.341
124.301
122.909
120.202
f1 (ppm)
F
F3C
NH2
CO2tBu
3w
13C NMR (100 MHz, CDCl3)

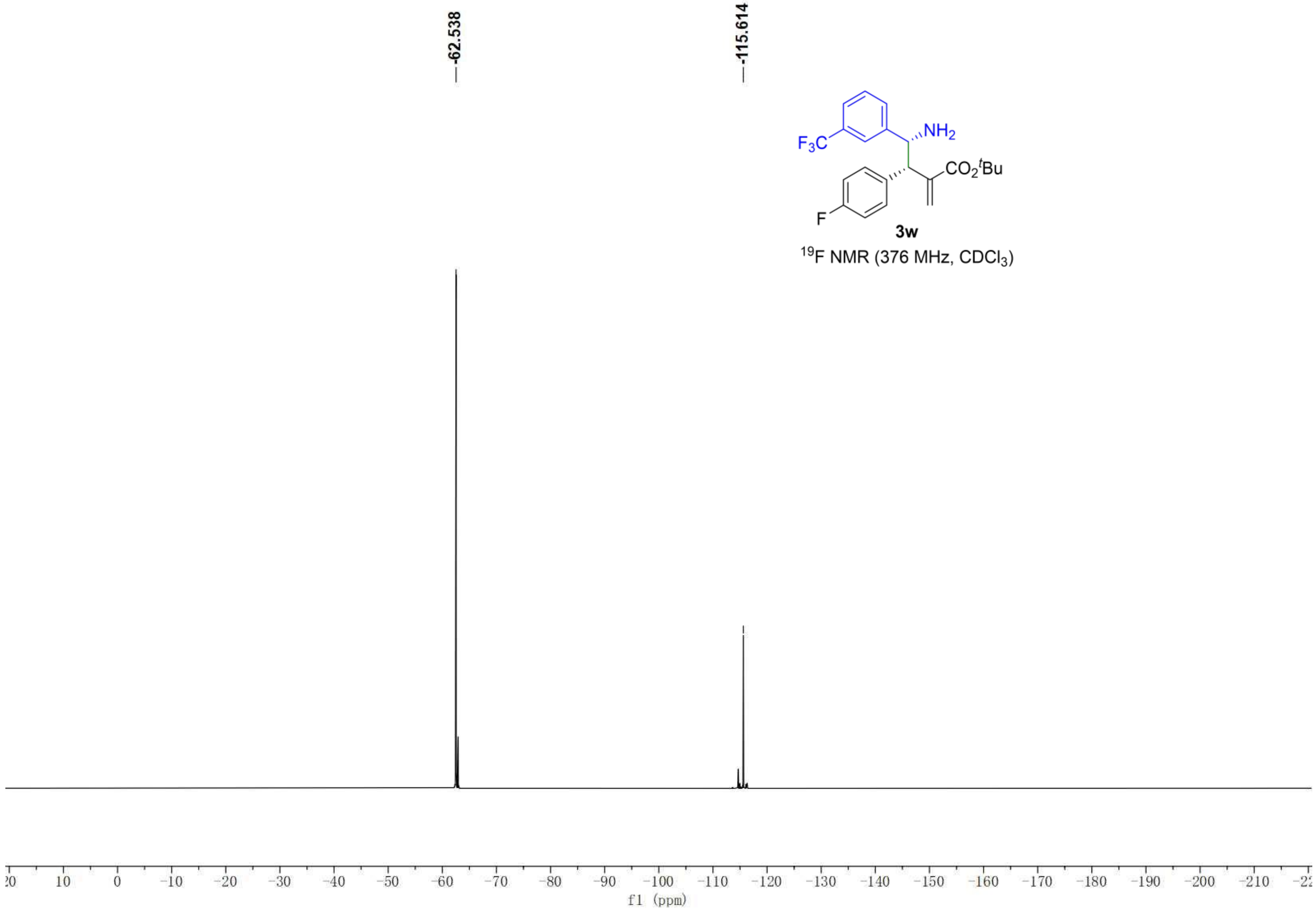
-62.538
-115.614
F_3C
NH_2
$CO_2{}^tBu$
F
3w
^{19}F NMR (376 MHz, $CDCl_3$)
20 10 0 -10 -20 -30 -40 -50 -60 -70 -80 -90 -100 -110 -120 -130 -140 -150 -160 -170 -180 -190 -200 -210
f1 (ppm)

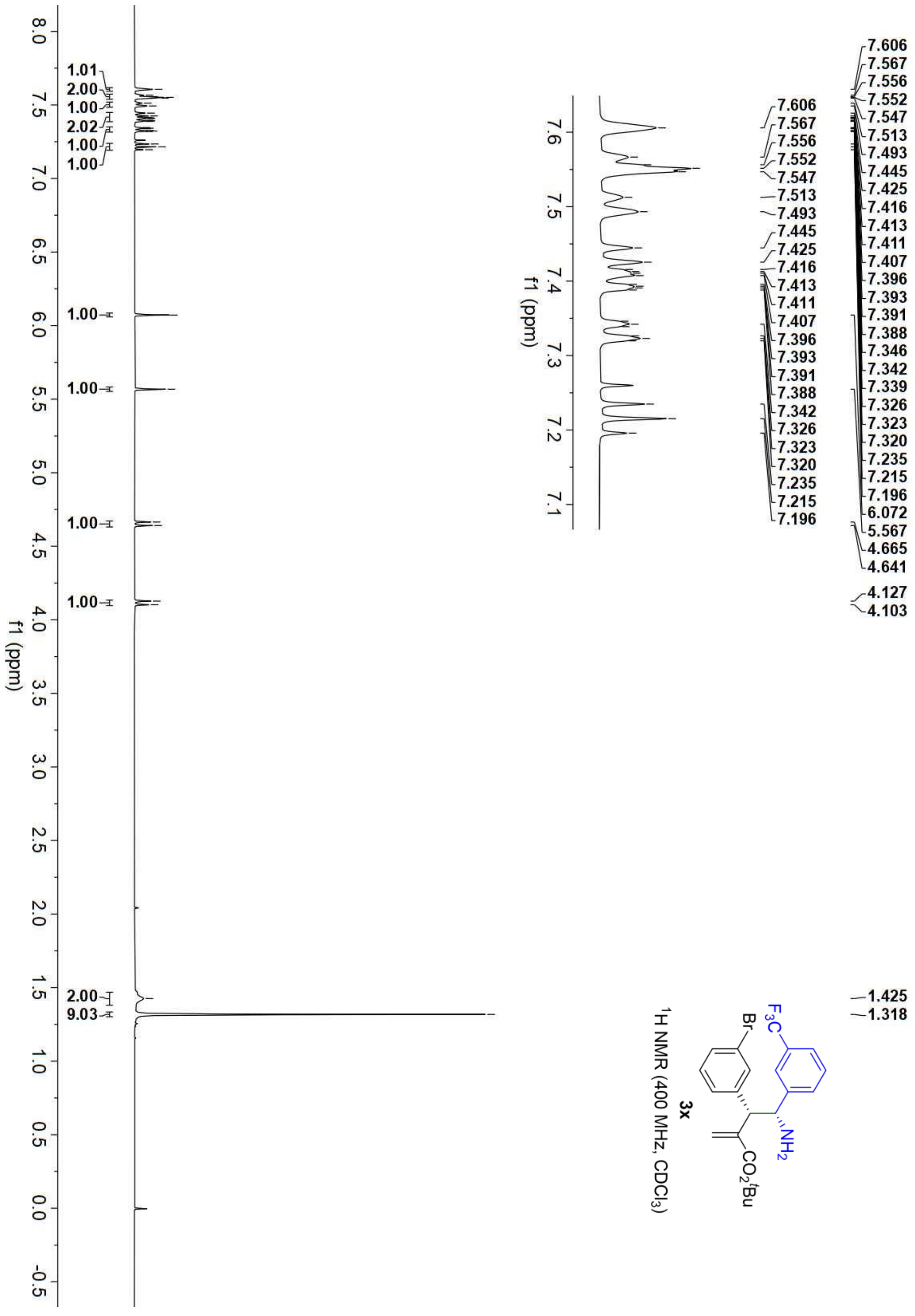
7.606
7.567
7.556
7.552
7.547
7.513
7.493
7.445
7.425
7.416
7.413
7.411
7.407
7.396
7.393
7.391
7.388
7.346
7.342
7.339
7.326
7.323
7.320
7.235
7.215
7.196
6.072
5.567
4.665
4.641
4.127
4.103
1.425
1.318
3x
1H NMR (400 MHz, CDCl3)
f1 (ppm)

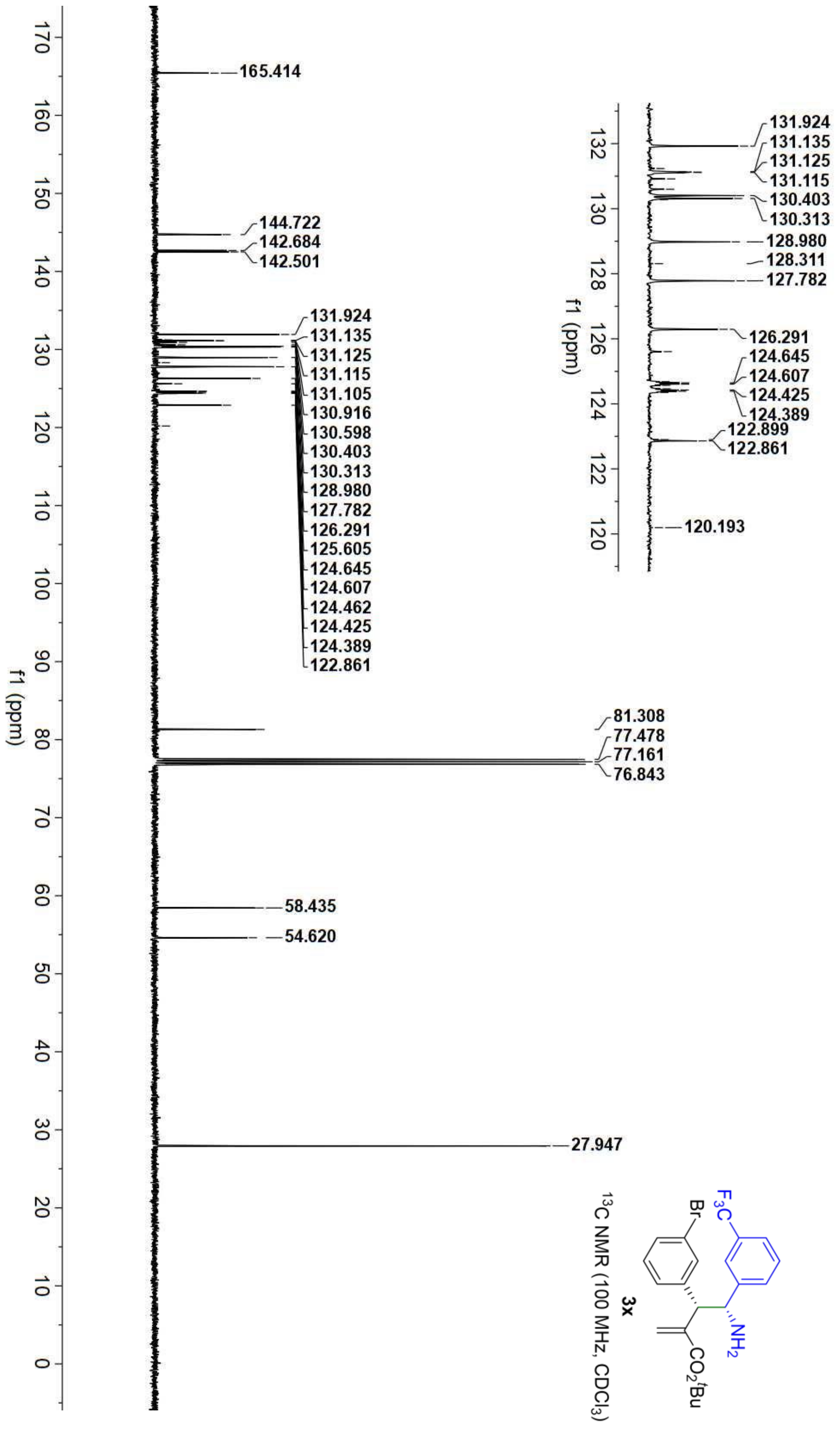
165.414
144.722
142.684
142.501
131.924
131.135
131.125
131.115
131.105
130.916
130.598
130.403
130.313
128.980
127.782
126.291
125.605
124.645
124.607
124.462
124.425
124.389
122.861
128.311
122.899
120.193
81.308
77.478
77.161
76.843
58.435
54.620
27.947
f1 (ppm)
F3C
Br
NH2
CO2tBu
3x
13C NMR (100 MHz, CDCl3)

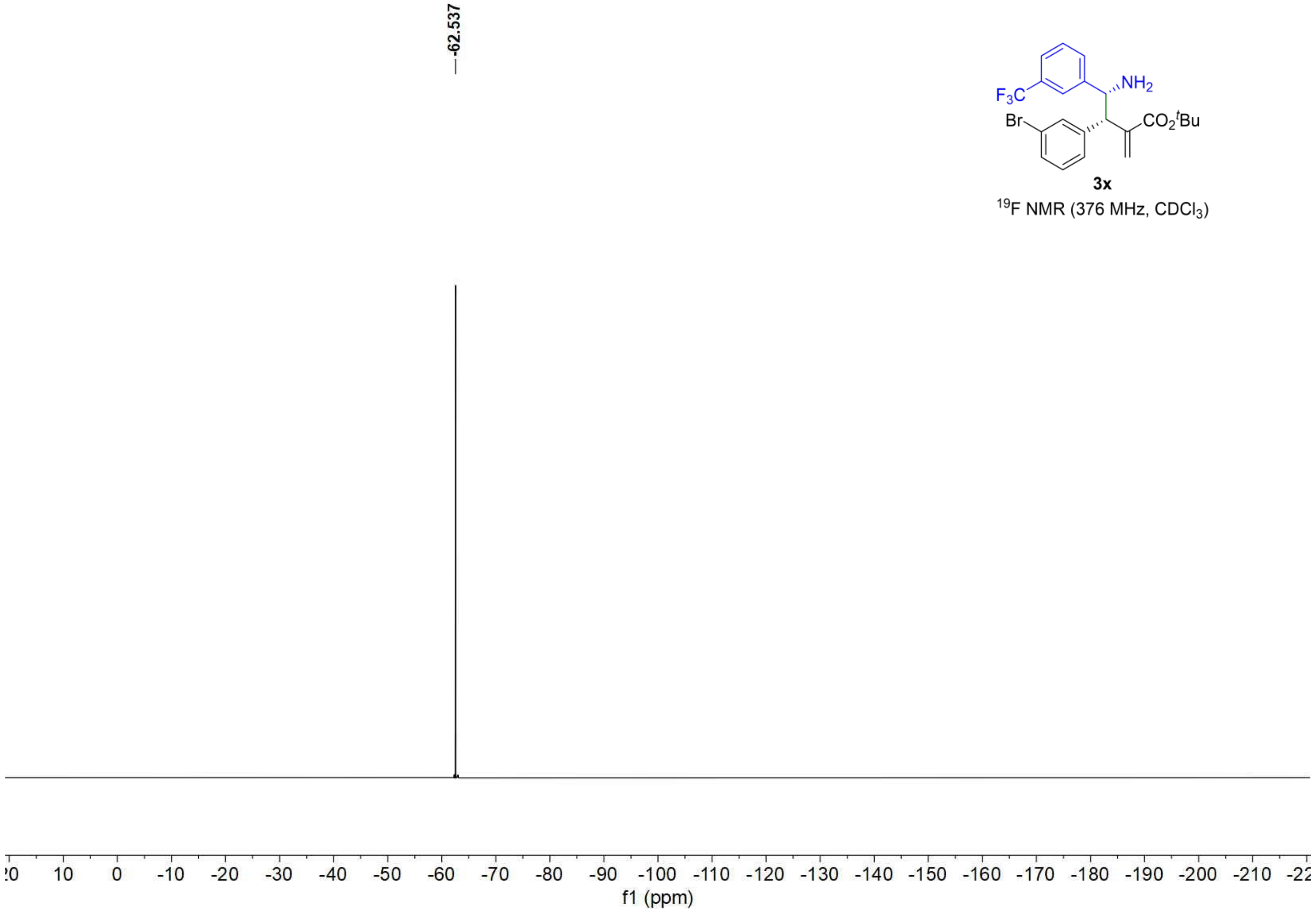
-62.537
NH2
F3C
Br
CO2tBu
3x
19F NMR (376 MHz, CDCl3)
10
0
-10
-20
-30
-40
-50
-60
-70
-80
-90
-100
-110
-120
-130
-140
-150
-160
-170
-180
-190
-200
-210
f1 (ppm)

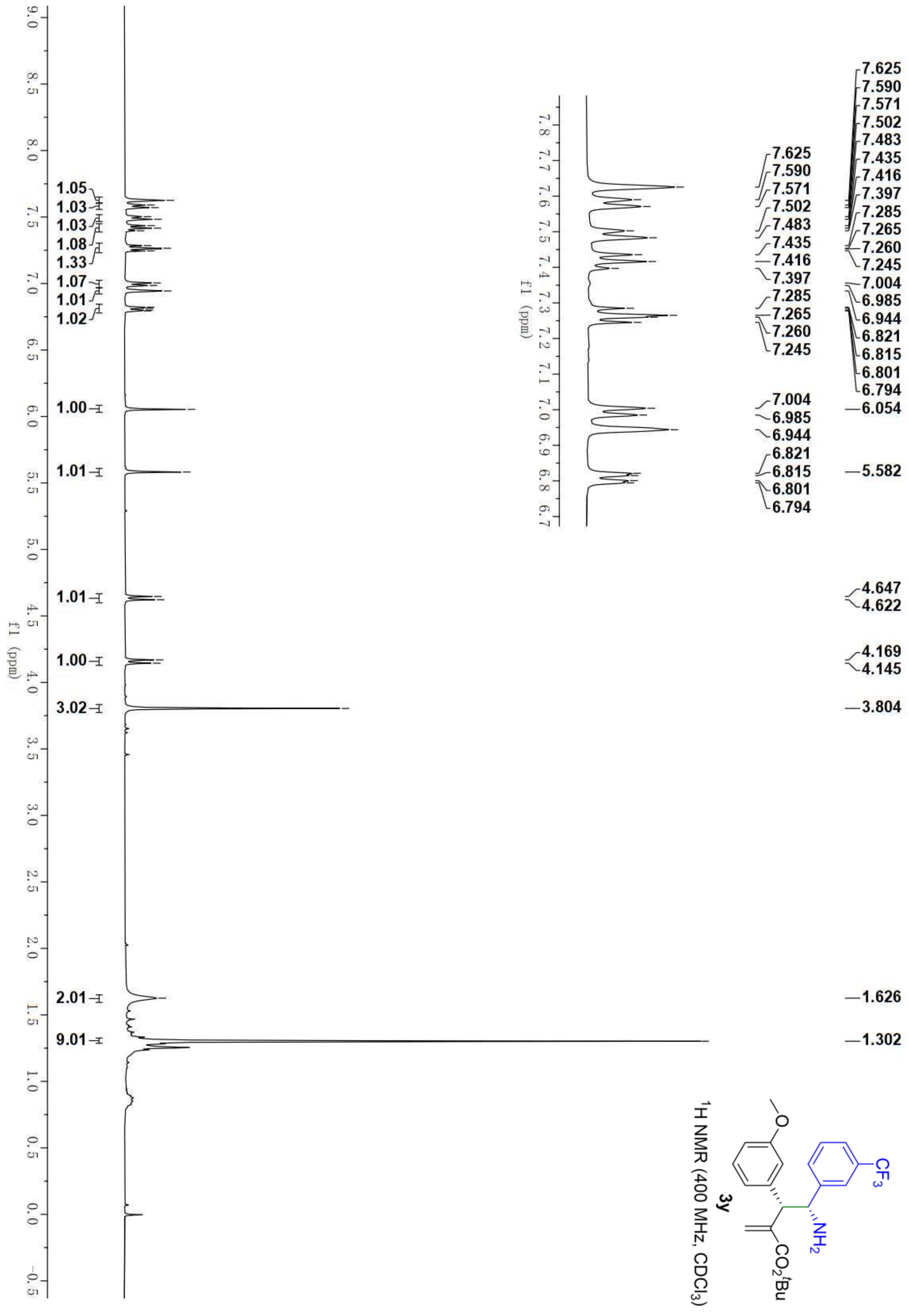
3y
1H NMR (400 MHz, CDCl3)

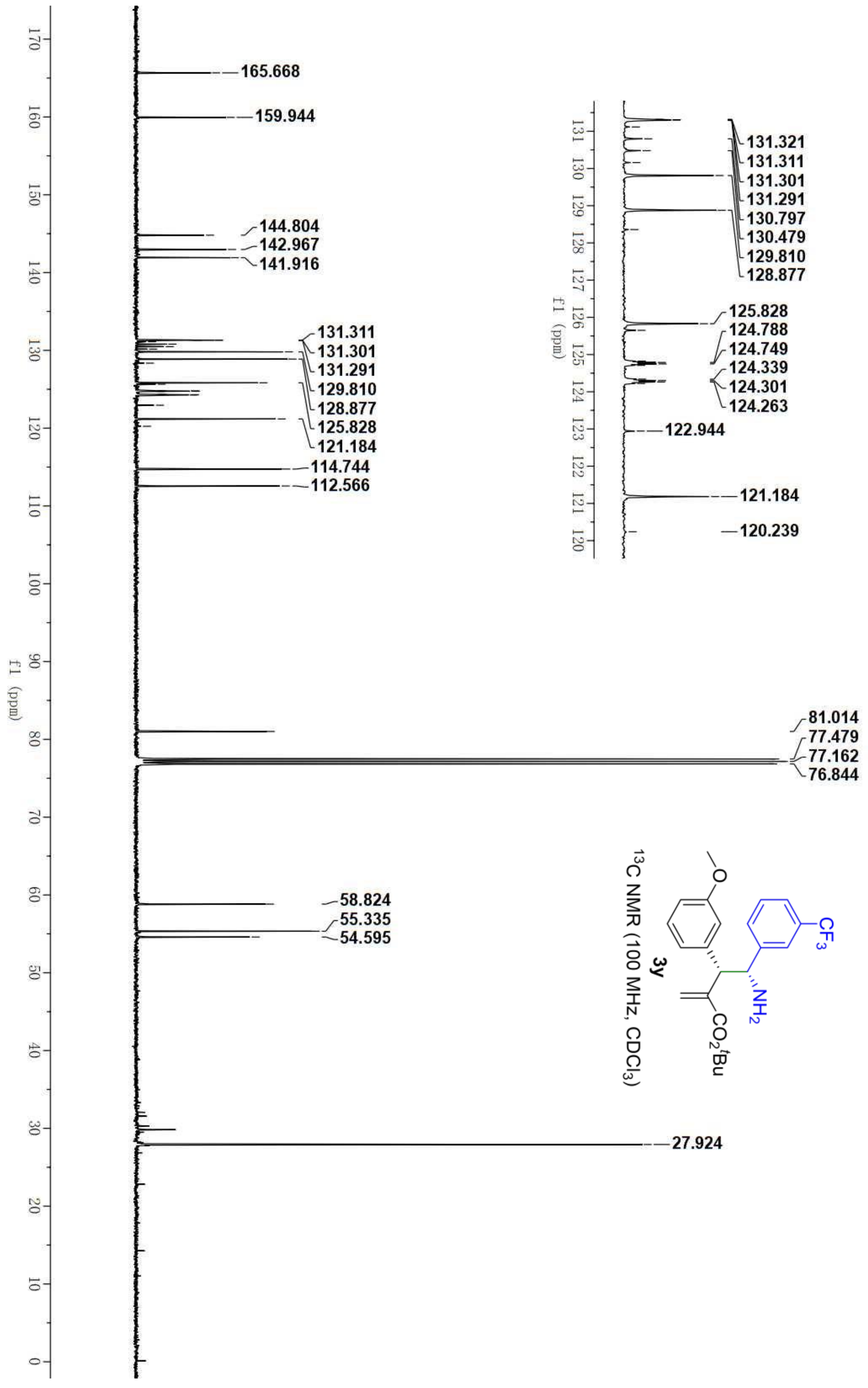
165.668
159.944
144.804
142.967
141.916
131.311
131.301
131.291
129.810
128.877
125.828
121.184
114.744
112.566
131.321
131.311
131.301
131.291
130.797
130.479
129.810
128.877
125.828
124.788
124.749
124.339
124.301
124.263
122.944
121.184
120.239
81.014
77.479
77.162
76.844
58.824
55.335
54.595
27.924
f1 (ppm)
3y
13C NMR (100 MHz, CDCl3)

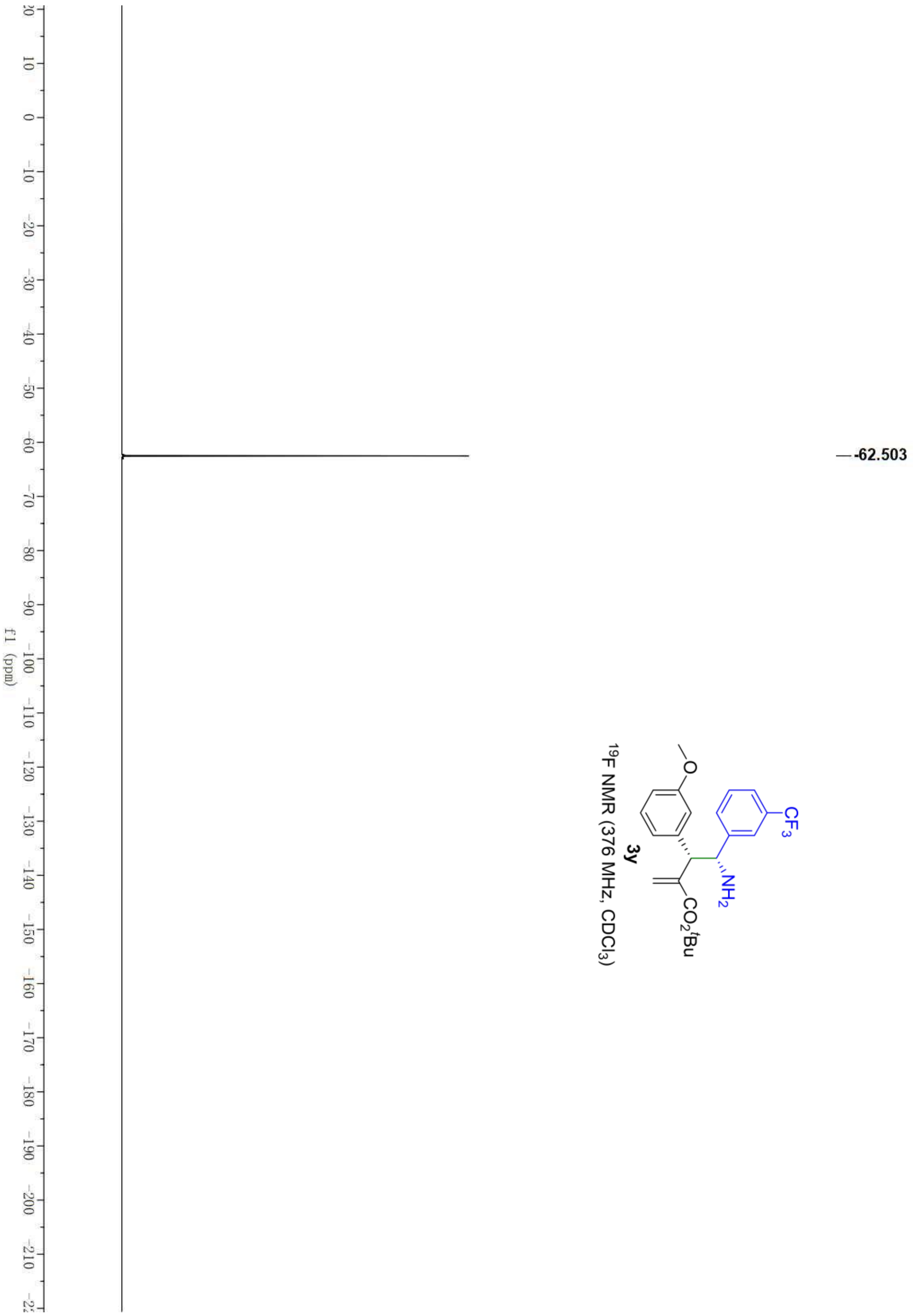
-62.503
3y
19F NMR (376 MHz, CDCl3)
f1 (ppm)

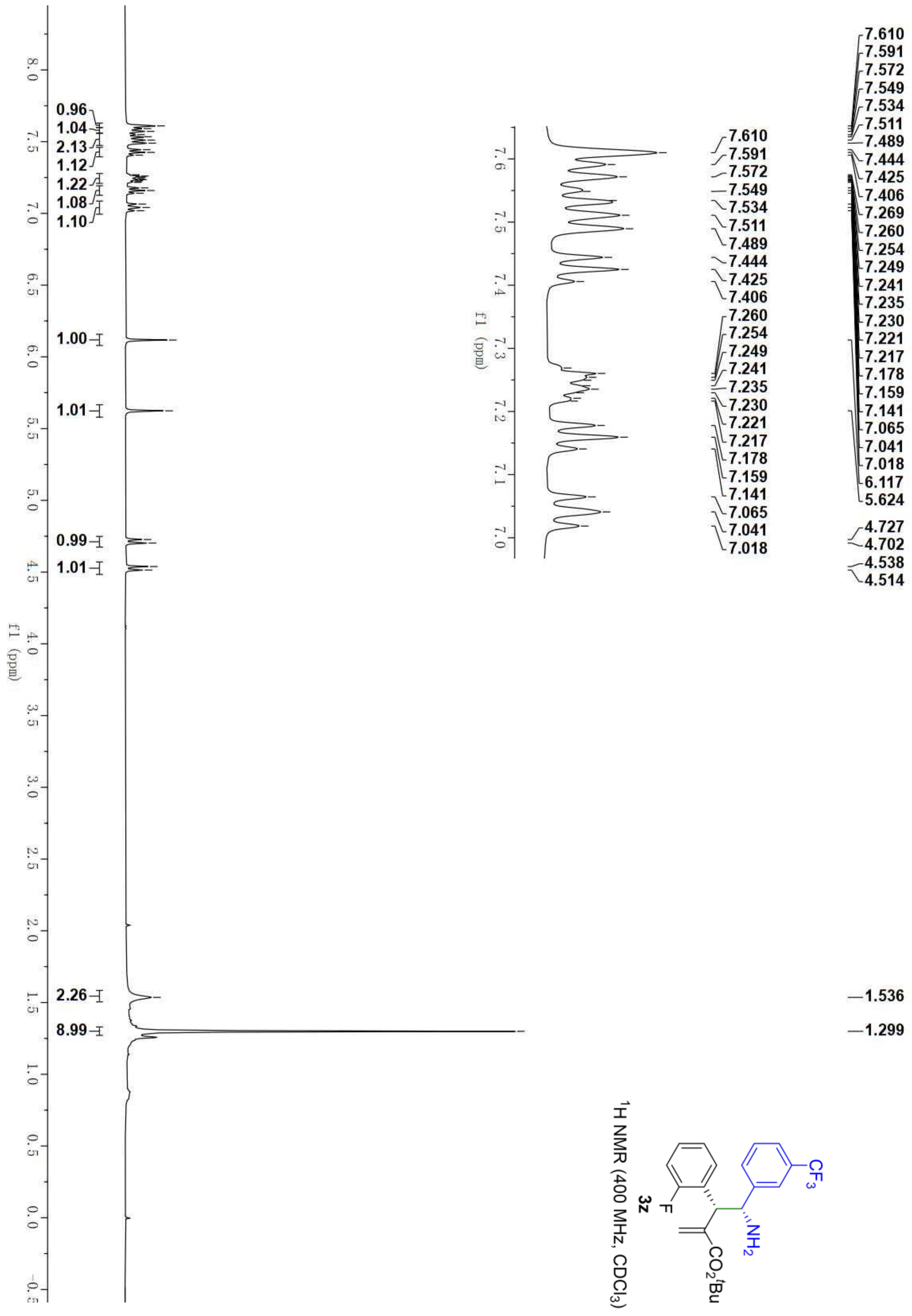
7.610
7.591
7.572
7.549
7.534
7.511
7.489
7.444
7.425
7.406
7.269
7.260
7.254
7.249
7.241
7.235
7.230
7.221
7.217
7.178
7.159
7.141
7.065
7.041
7.018
6.117
5.624
4.727
4.702
4.538
4.514
1.536
1.299
0.96
1.04
2.13
1.12
1.22
1.08
1.10
1.00
1.01
0.99
1.01
2.26
8.99
f1 (ppm)
CF3
NH2
F
CO2tBu
3z
1H NMR (400 MHz, CDCl3)

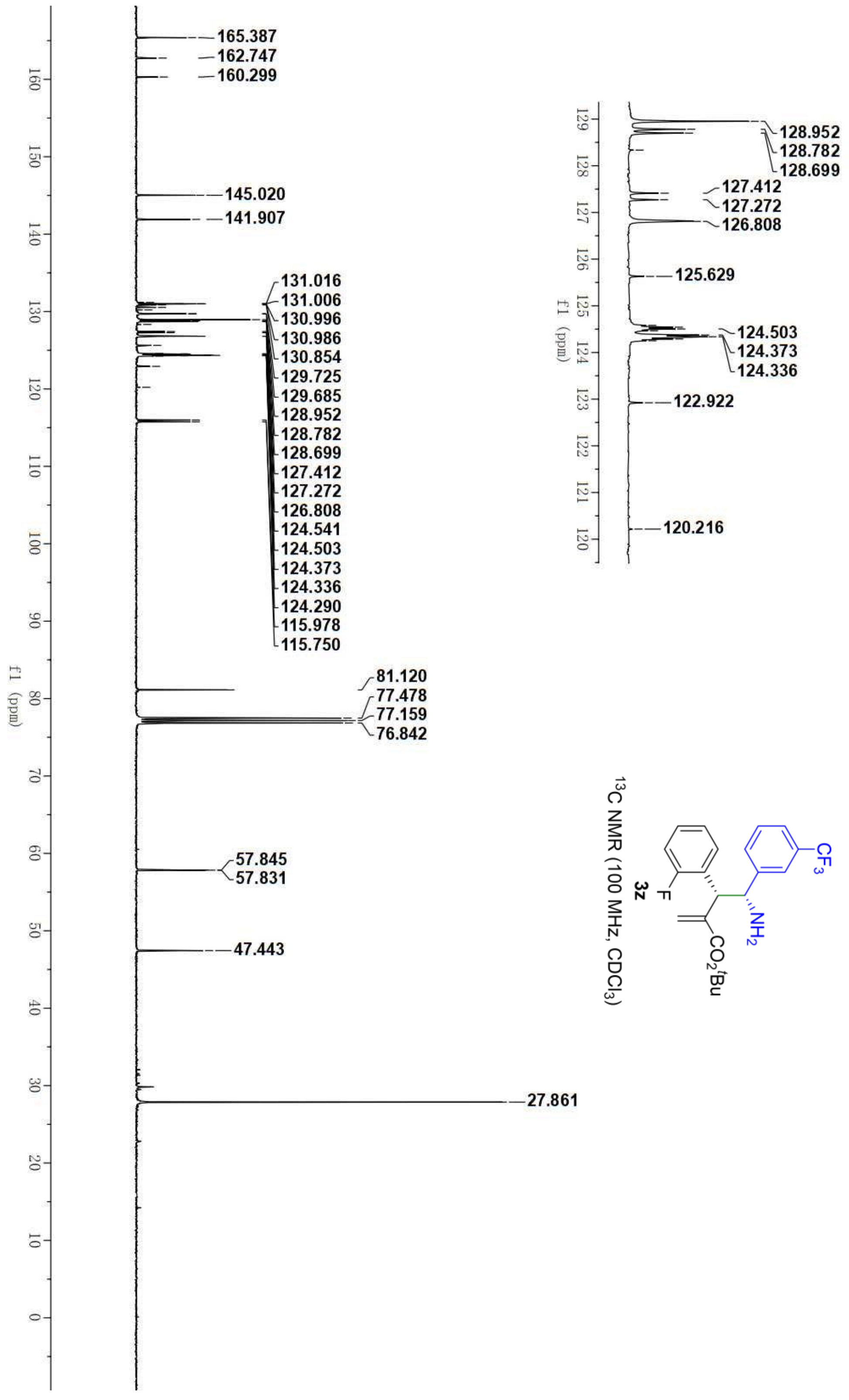

165.387
162.747
160.299
145.020
141.907
131.016
131.006
130.996
130.986
130.854
129.725
129.685
128.952
128.782
128.699
127.412
127.272
126.808
124.541
124.503
124.373
124.336
124.290
115.978
115.750
81.120
77.478
77.159
76.842
57.845
57.831
47.443
27.861
f1 (ppm)
125.629
122.922
120.216
CF3
NH2
F
CO2tBu
3z
13C NMR (100 MHz, CDCl3)

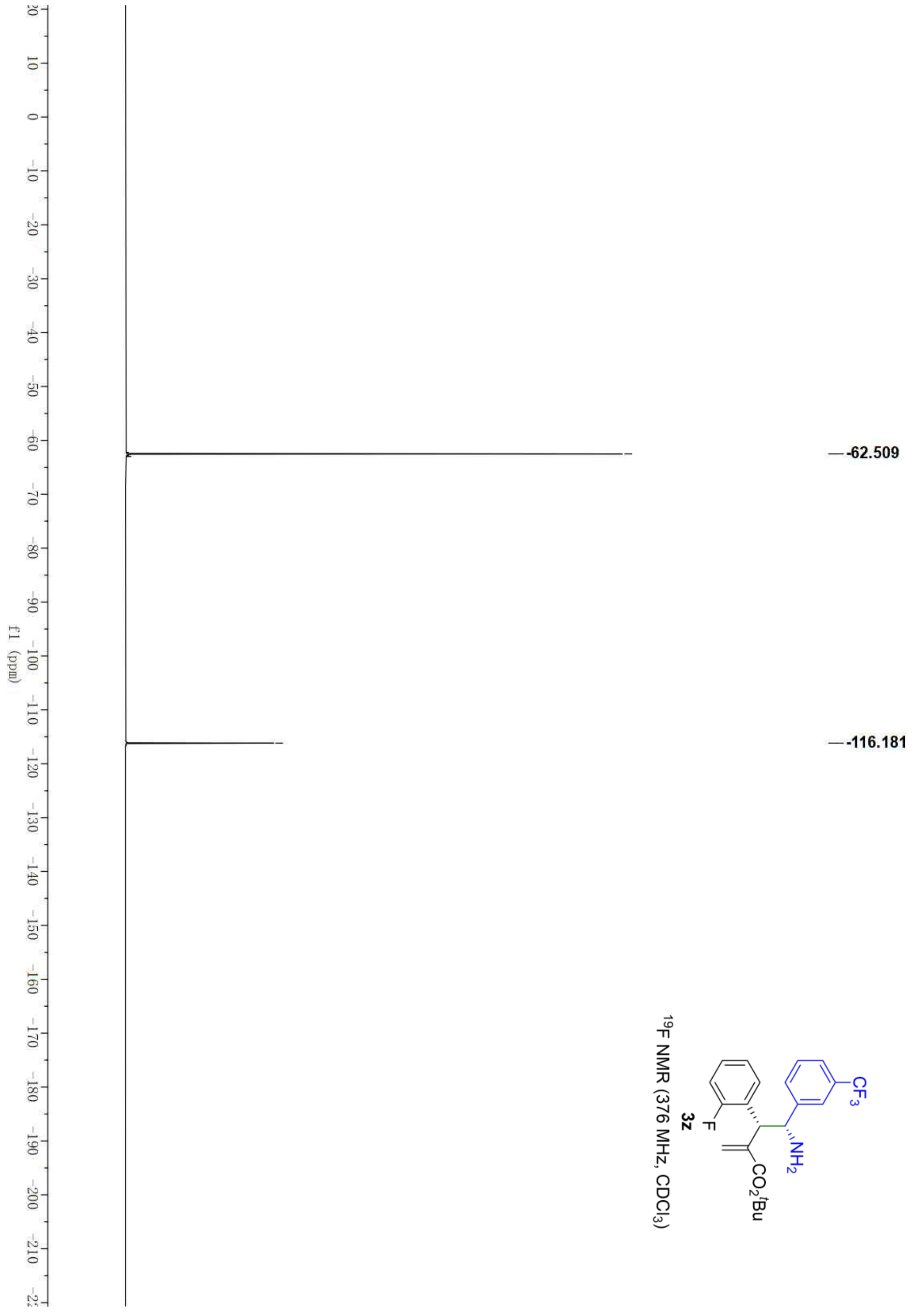
-62.509
-116.181
f1 (ppm)
3z
19F NMR (376 MHz, CDCl3)

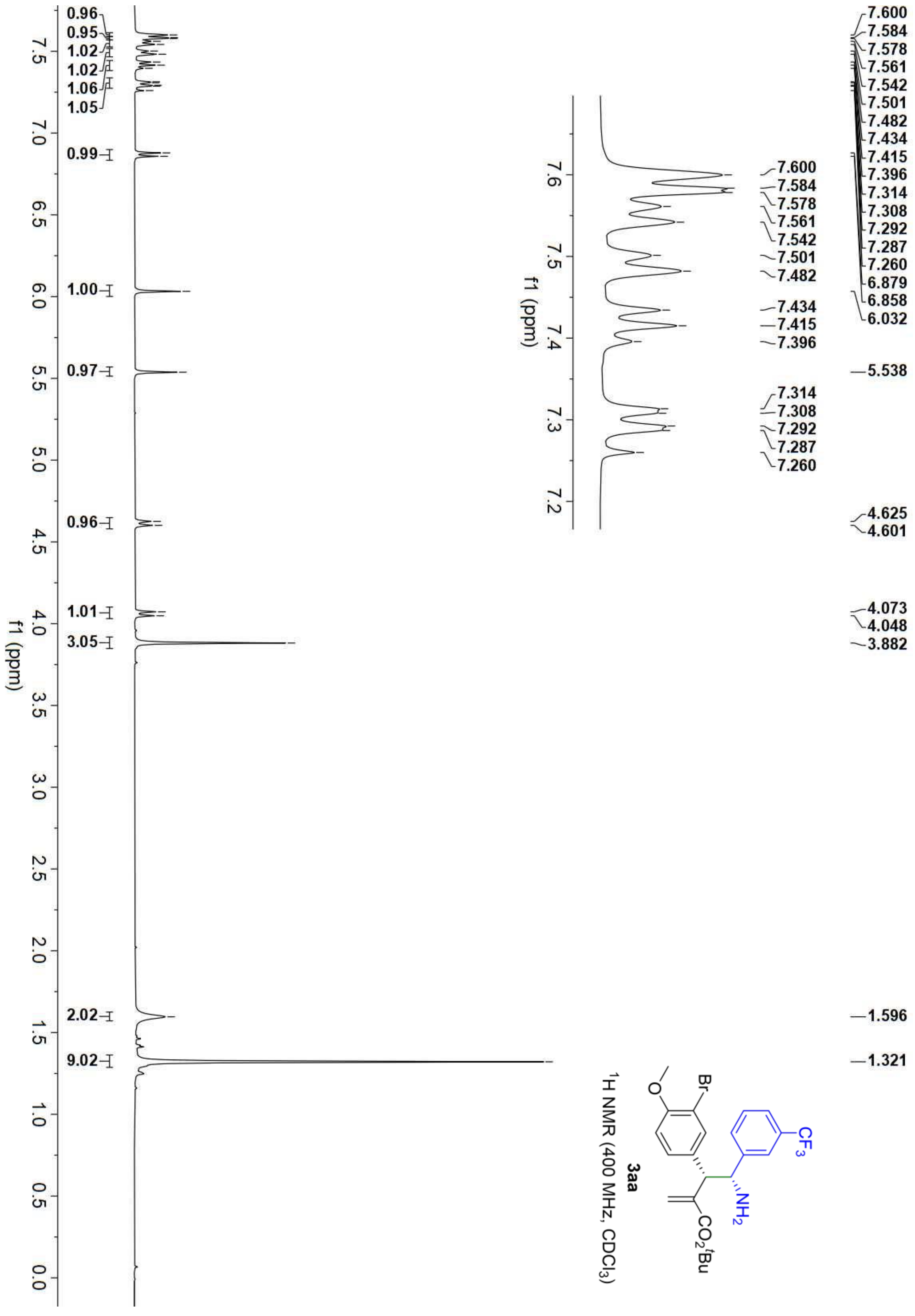

7.600
7.584
7.578
7.561
7.542
7.501
7.482
7.434
7.415
7.396
7.314
7.308
7.292
7.287
7.260
6.879
6.858
6.032
5.538
4.625
4.601
4.073
4.048
3.882
1.596
1.321
0.96
0.95
1.02
1.02
1.06
1.05
0.99
1.00
0.97
0.96
1.01
3.05
2.02
9.02
f1 (ppm)
CF3
NH2
Br
O
CO2tBu
3aa
1H NMR (400 MHz, CDCl3)

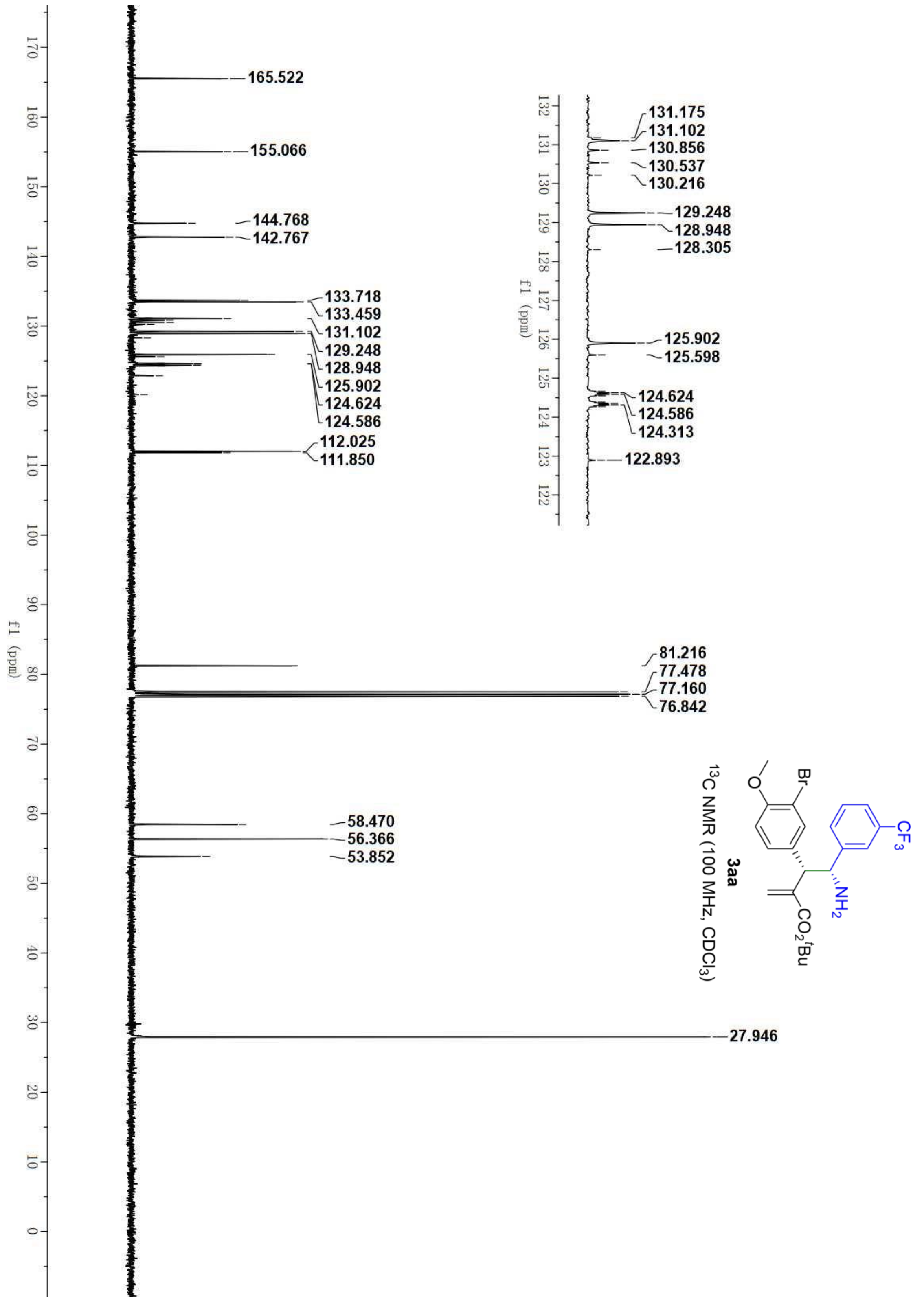
165.522
155.066
144.768
142.767
133.718
133.459
131.102
129.248
128.948
125.902
124.624
124.586
112.025
111.850
131.175
131.102
130.856
130.537
130.216
129.248
128.948
128.305
125.902
125.598
124.624
124.586
124.313
122.893
81.216
77.478
77.160
76.842
58.470
56.366
53.852
27.946
f1 (ppm)
Br
CF_3
NH_2
$CO_2{}^tBu$
3aa
^{13}C NMR (100 MHz, $CDCl_3$)

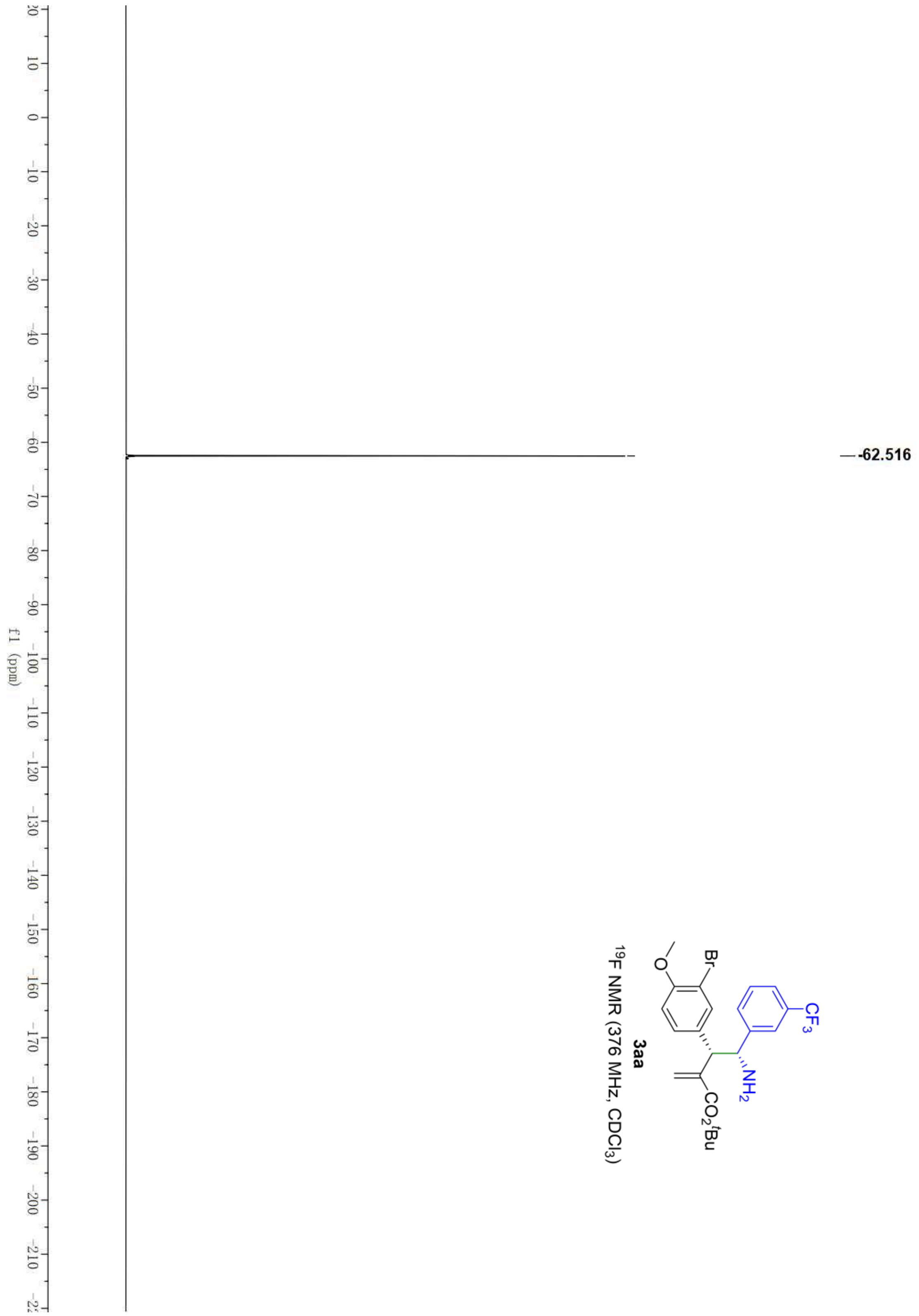

-62.516
3aa
^{19}F NMR (376 MHz, CDCl$_3$)
f1 (ppm)

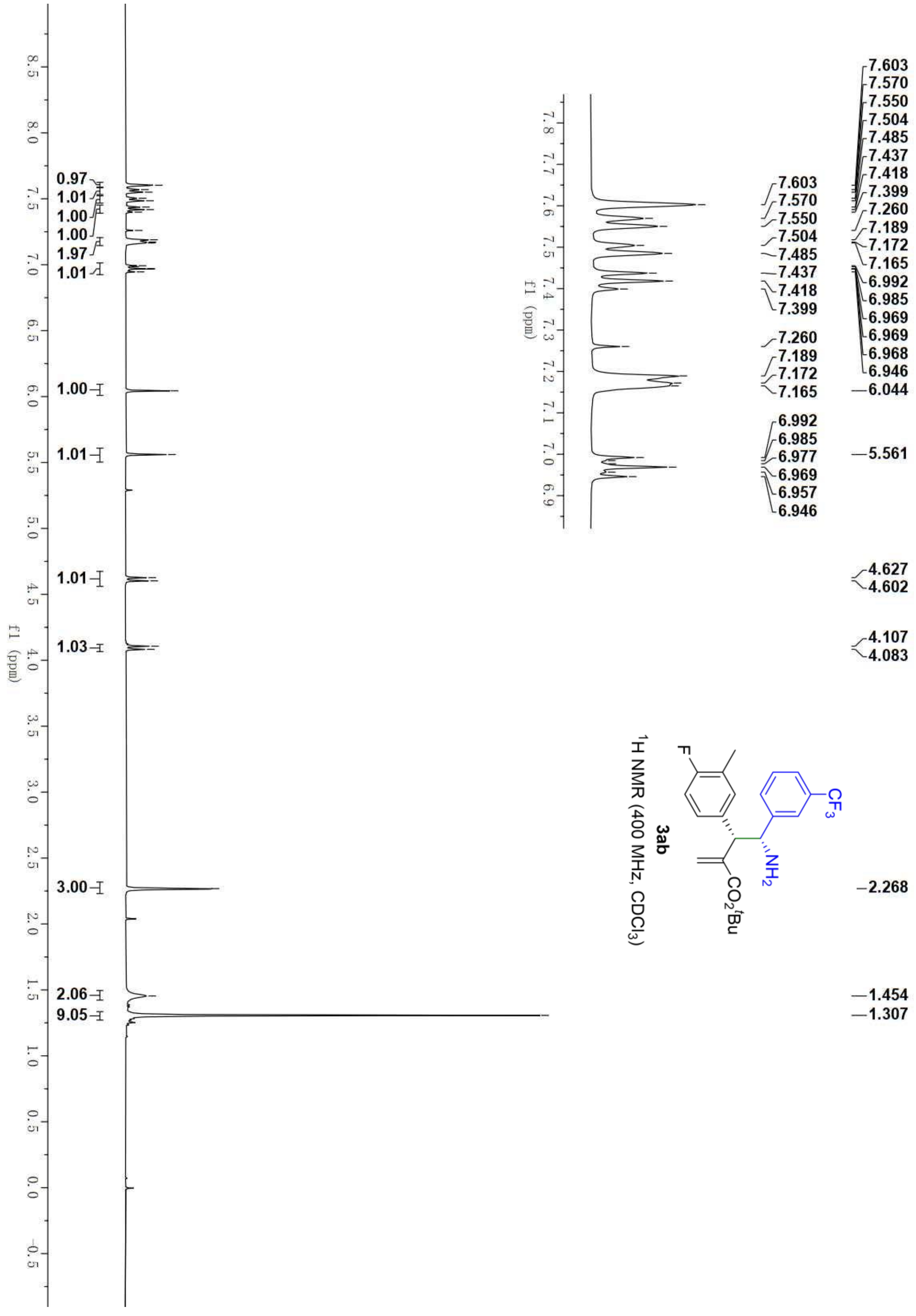

7.603
7.570
7.550
7.504
7.485
7.437
7.418
7.399
7.260
7.189
7.172
7.165
6.992
6.985
6.969
6.969
6.968
6.946
6.044
5.561
4.627
4.602
4.107
4.083
2.268
1.454
1.307
CF3
NH2
CO2tBu
F
3ab
1H NMR (400 MHz, CDCl3)
f1 (ppm)

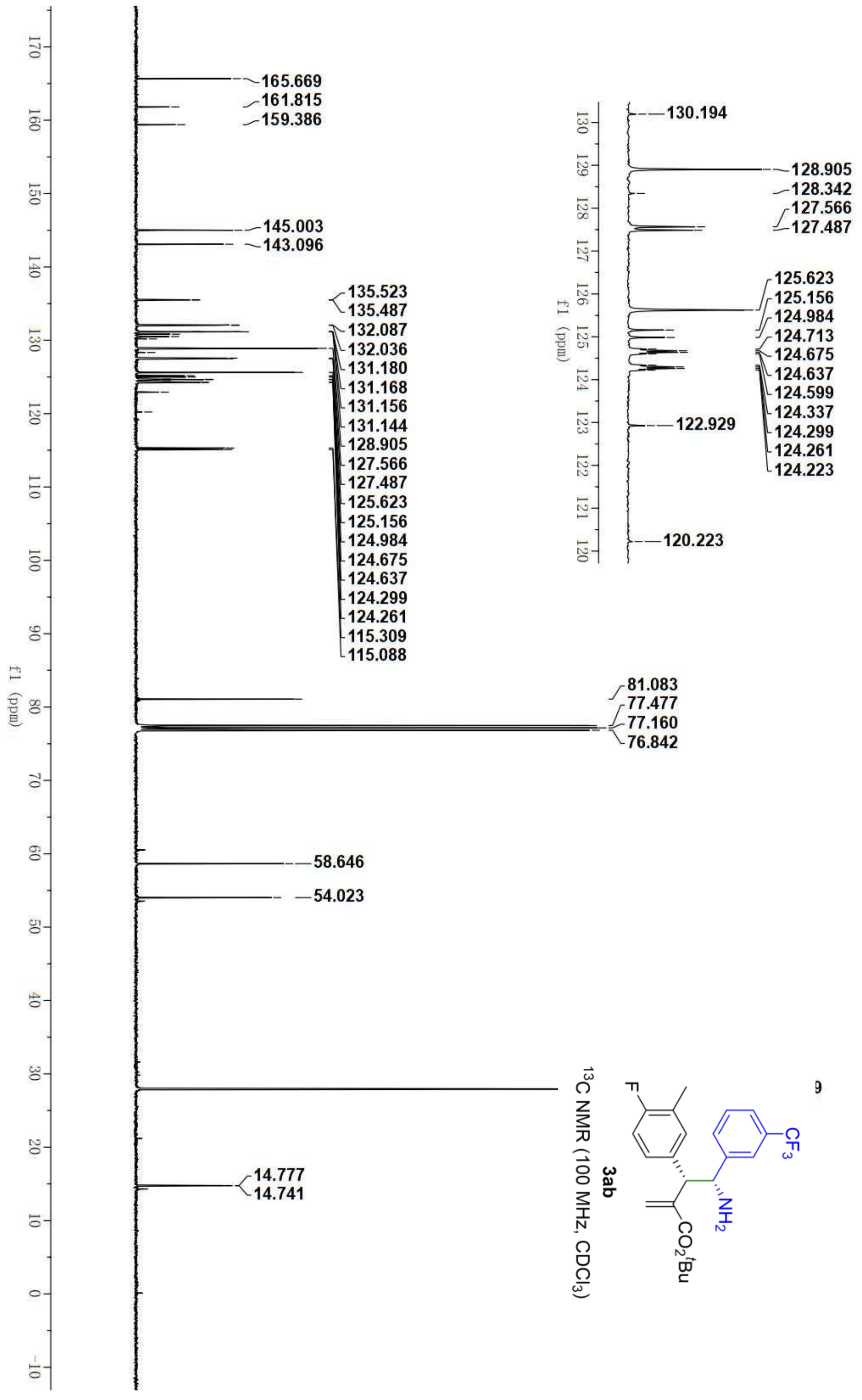
165.669
161.815
159.386
145.003
143.096
135.523
135.487
132.087
132.036
131.180
131.168
131.156
131.144
128.905
127.566
127.487
125.623
125.156
124.984
124.675
124.637
124.299
124.261
115.309
115.088
81.083
77.477
77.160
76.842
58.646
54.023
14.777
14.741
130.194
128.905
128.342
127.566
127.487
125.623
125.156
124.984
124.713
124.675
124.637
124.599
124.337
124.299
124.261
124.223
122.929
120.223
f1 (ppm)
F
CF_3
NH_2
$CO_2{}^tBu$
3ab
^{13}C NMR (100 MHz, $CDCl_3$)

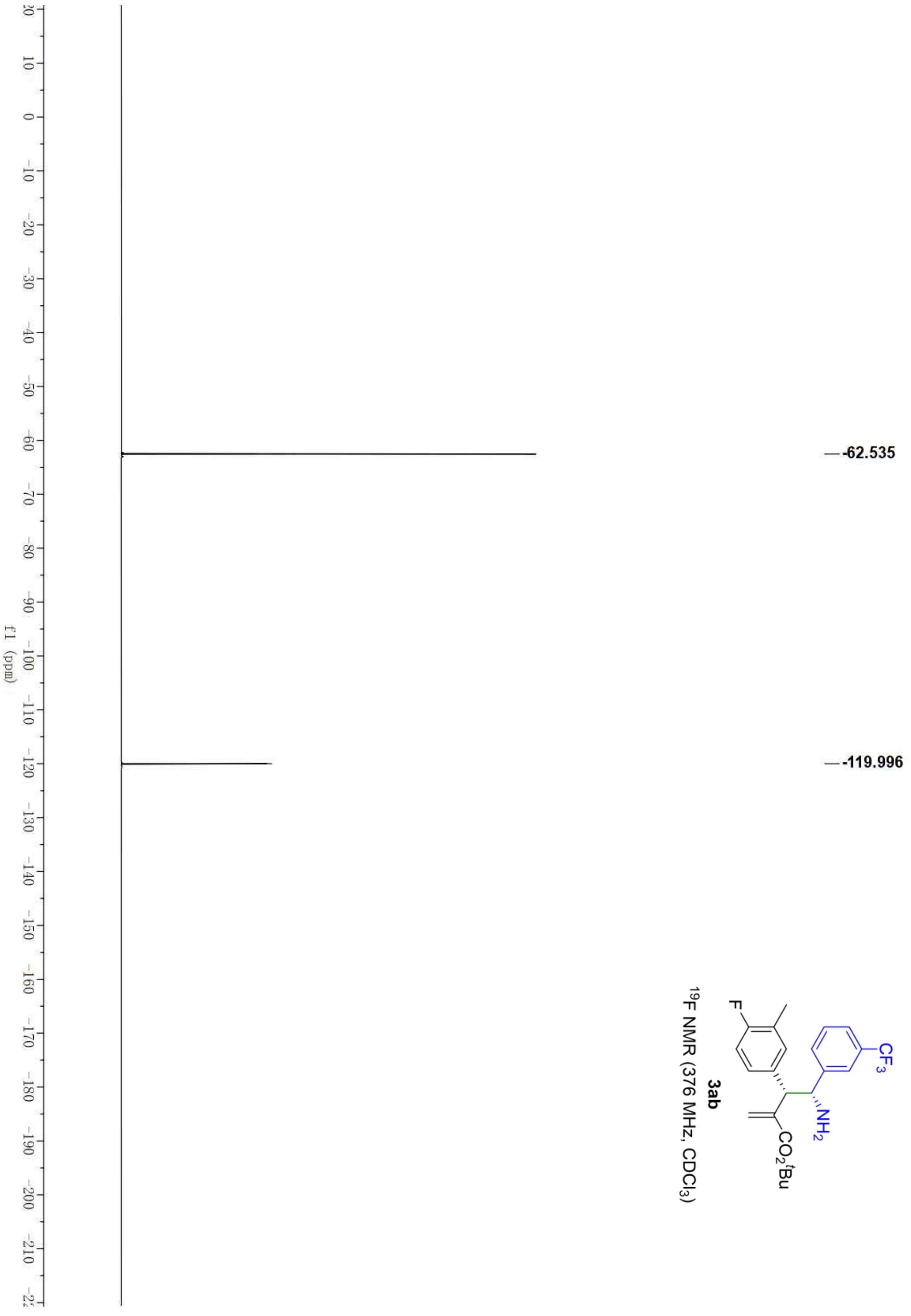
-62.535
-119.996
CF3
NH2
F
CO2tBu
3ab
19F NMR (376 MHz, CDCl3)
f1 (ppm)

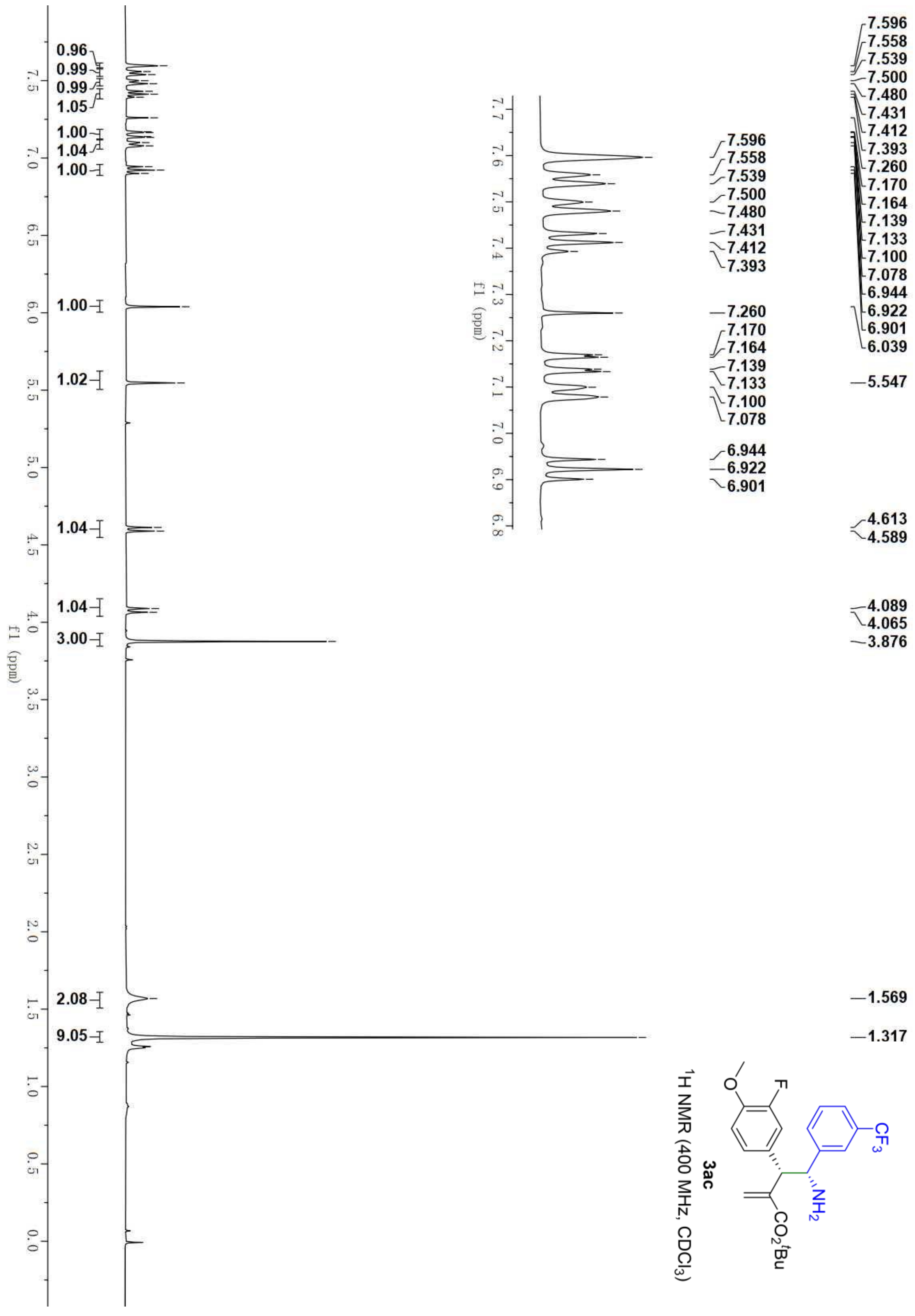
7.596
7.558
7.539
7.500
7.480
7.431
7.412
7.393
7.260
7.170
7.164
7.139
7.133
7.100
7.078
6.944
6.922
6.901
6.039
5.547
4.613
4.589
4.089
4.065
3.876
1.569
1.317
0.96
0.99
0.99
1.05
1.00
1.04
1.00
1.00
1.02
1.04
1.04
3.00
2.08
9.05
f1 (ppm)
F
O
CF3
NH2
CO2tBu
3ac
1H NMR (400 MHz, CDCl3)

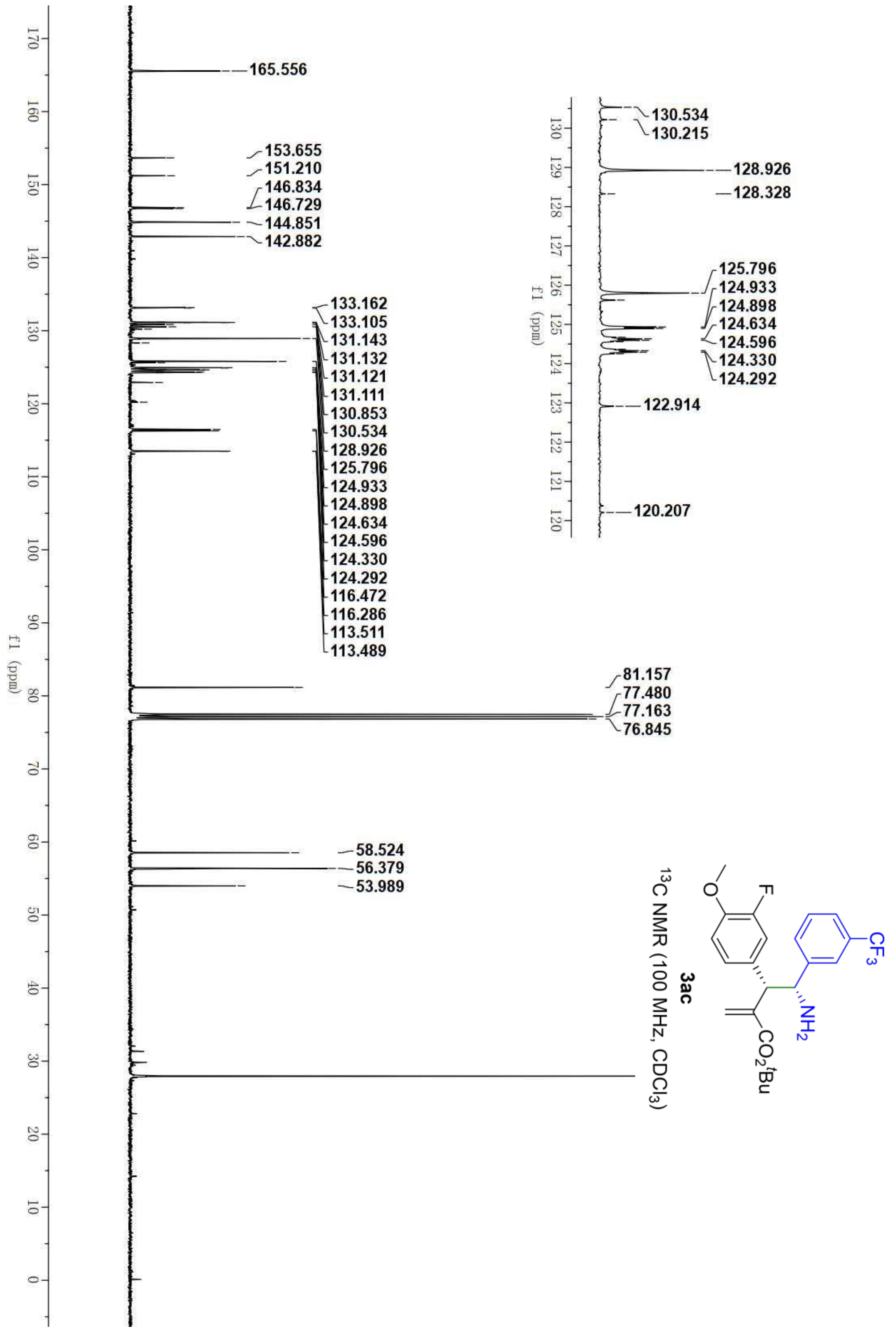
165.556
153.655
151.210
146.834
146.729
144.851
142.882
133.162
133.105
131.143
131.132
131.121
131.111
130.853
130.534
128.926
125.796
124.933
124.898
124.634
124.596
124.330
124.292
116.472
116.286
113.511
113.489
81.157
77.480
77.163
76.845
58.524
56.379
53.989
130.534
130.215
128.926
128.328
125.796
124.933
124.898
124.634
124.596
124.330
124.292
122.914
120.207
f1 (ppm)
F
O
CF3
NH2
CO2tBu
3ac
13C NMR (100 MHz, CDCl3)

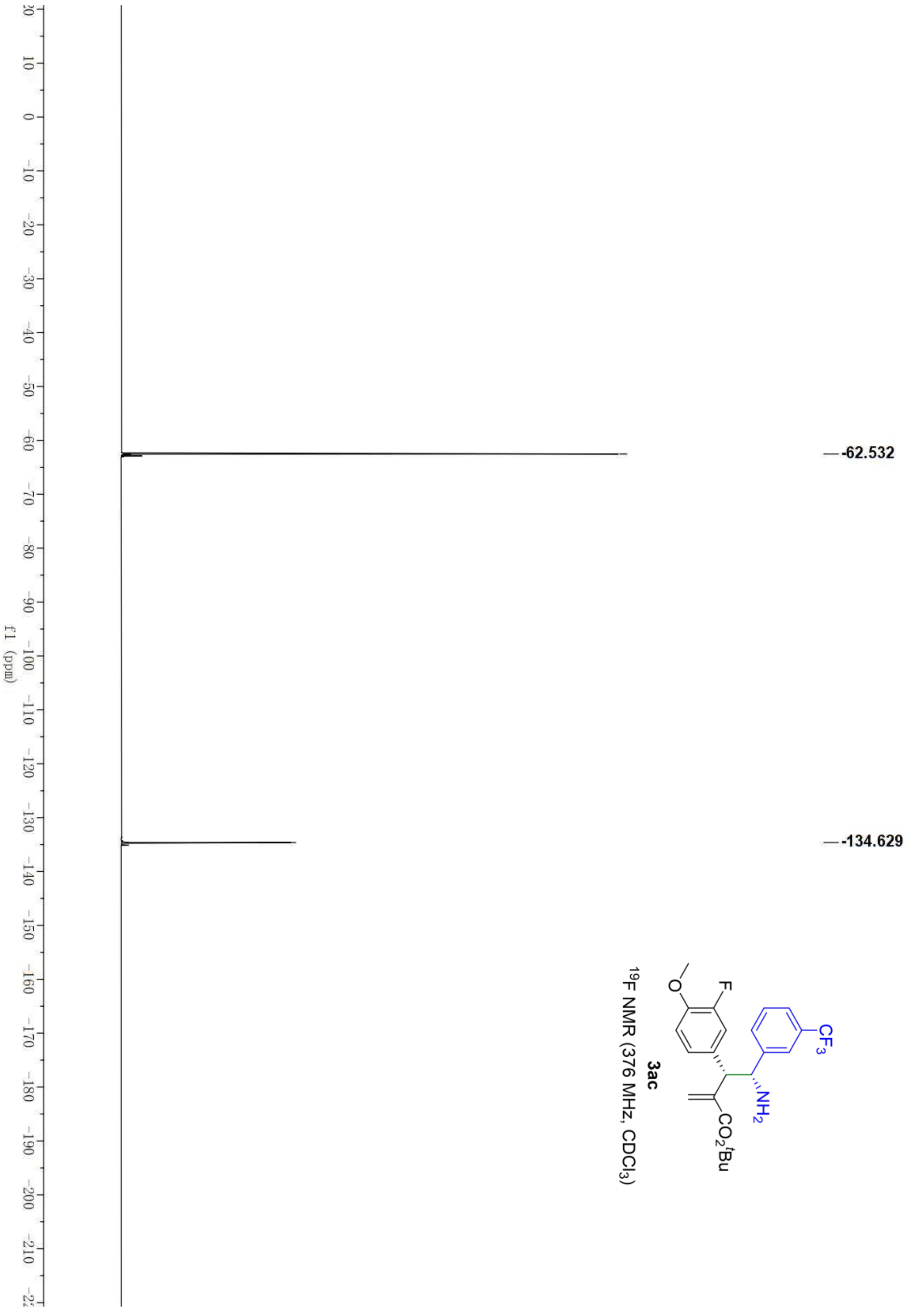
-62.532
-134.629
CF3
NH2
F
O
CO2tBu
3ac
19F NMR (376 MHz, CDCl3)
f1 (ppm)

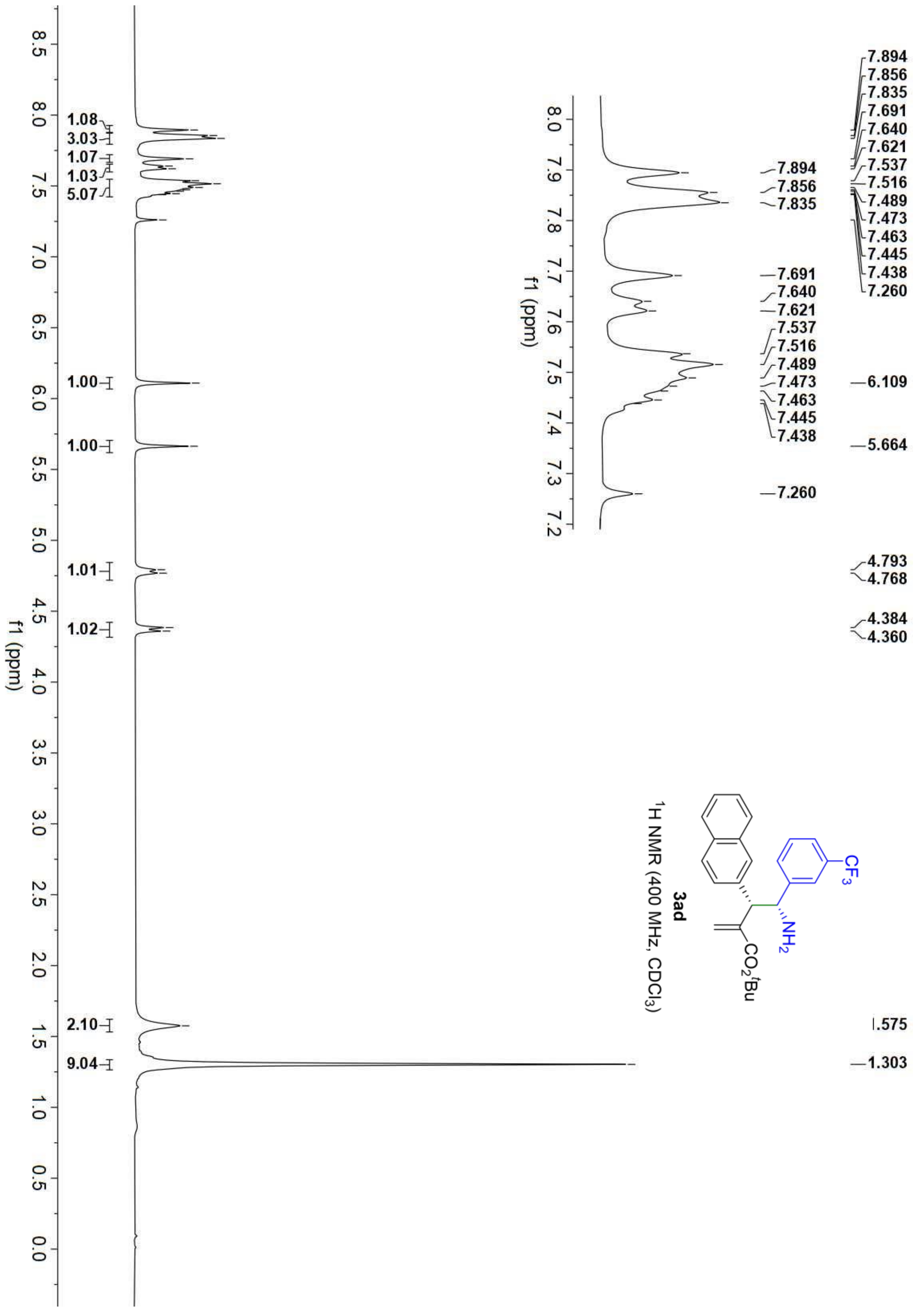

3ad
1H NMR (400 MHz, CDCl3)
CF3
NH2
CO2tBu
7.894
7.856
7.835
7.691
7.640
7.621
7.537
7.516
7.489
7.473
7.463
7.445
7.438
7.260
6.109
5.664
4.793
4.768
4.384
4.360
.575
1.303
1.08
3.03
1.07
1.03
5.07
1.00
1.00
1.01
1.02
2.10
9.04
f1 (ppm)

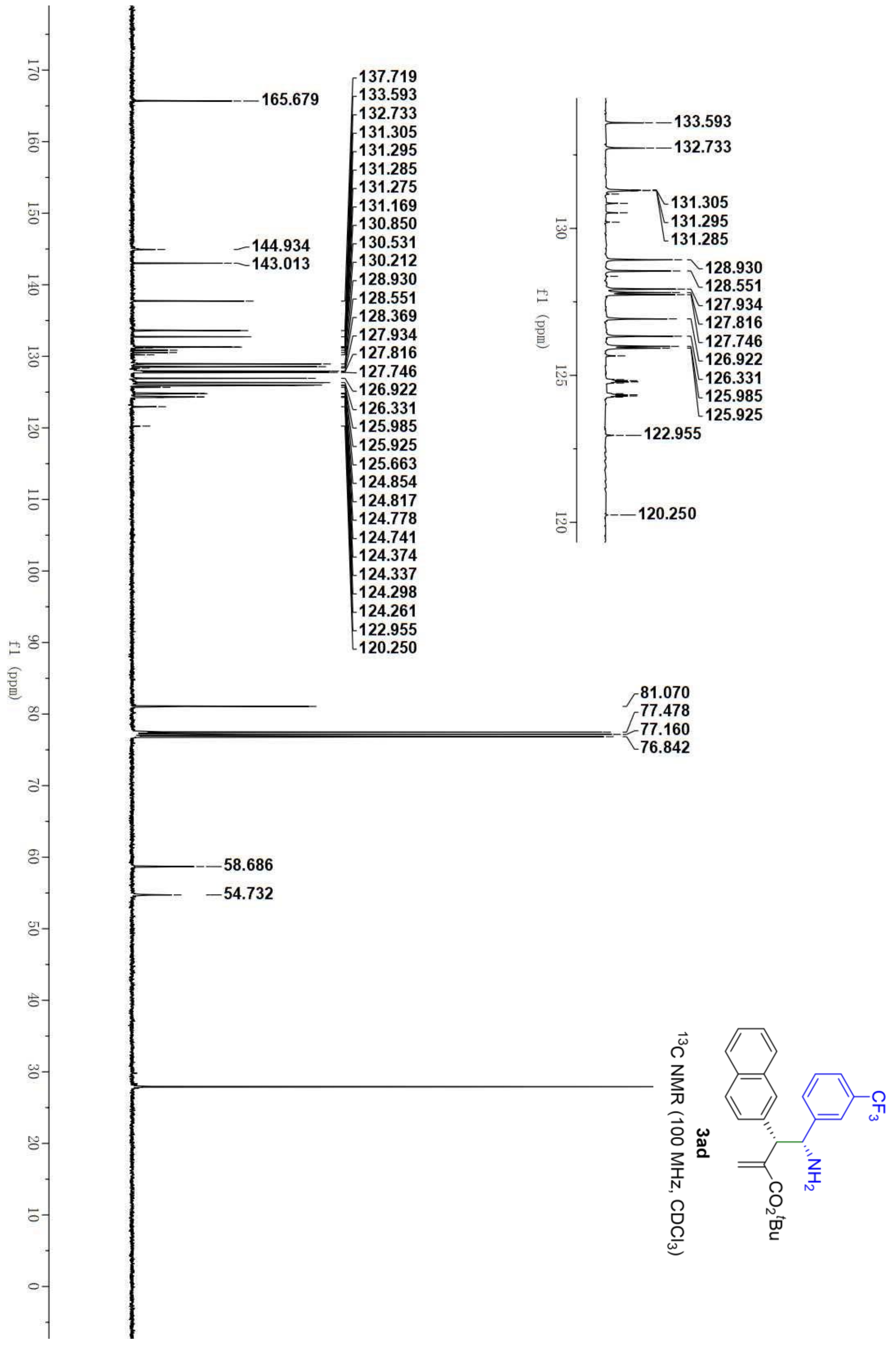
165.679
144.934
143.013
137.719
133.593
132.733
131.305
131.295
131.285
131.275
131.169
130.850
130.531
130.212
128.930
128.551
128.369
127.934
127.816
127.746
126.922
126.331
125.985
125.925
125.663
124.854
124.817
124.778
124.741
124.374
124.337
124.298
124.261
122.955
120.250
81.070
77.478
77.160
76.842
58.686
54.732
f1 (ppm)
CF3
NH2
CO2tBu
3ad
13C NMR (100 MHz, CDCl3)

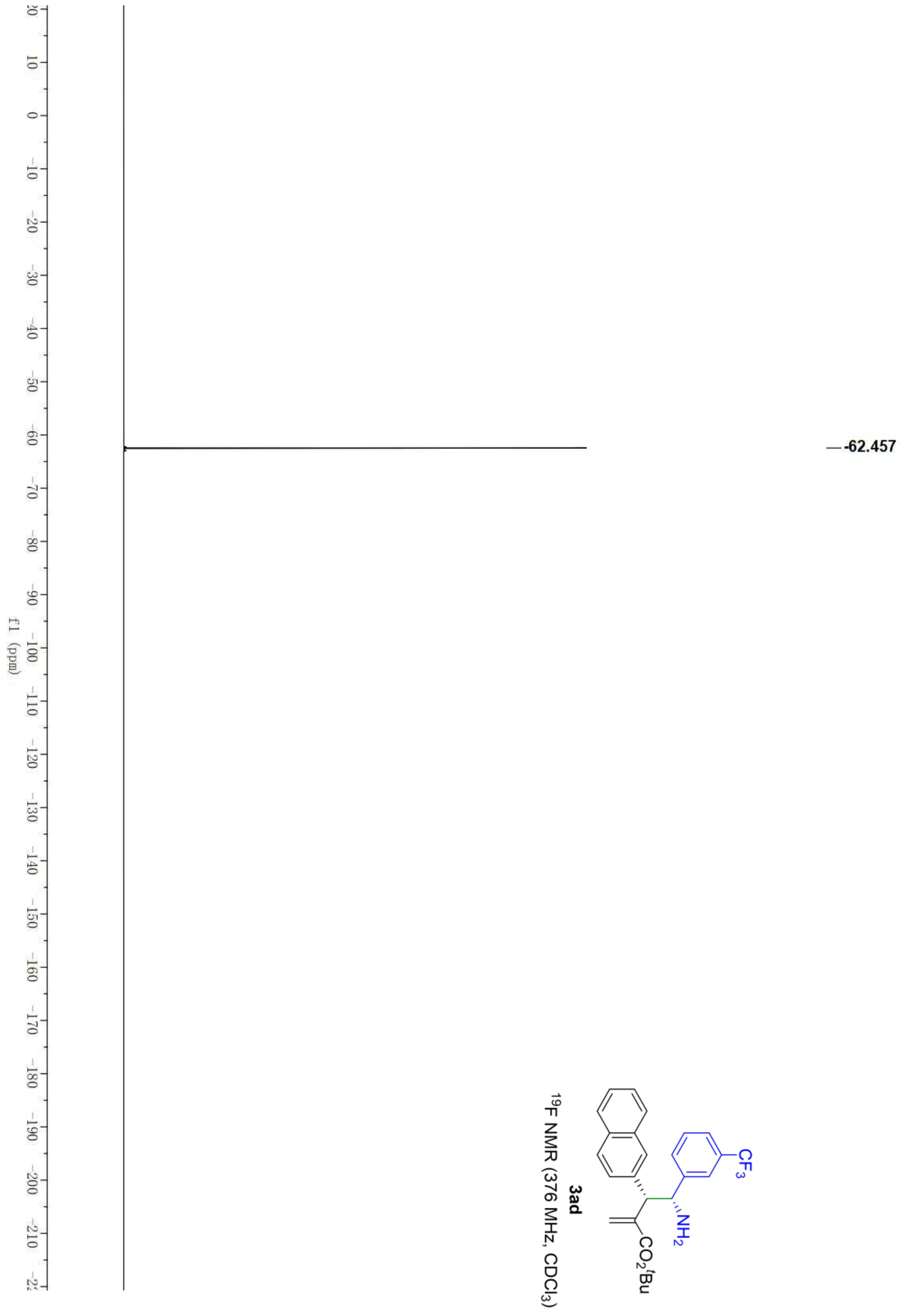

-62.457
f1 (ppm)
CF3
NH2
CO2tBu
3ad
19F NMR (376 MHz, CDCl3)

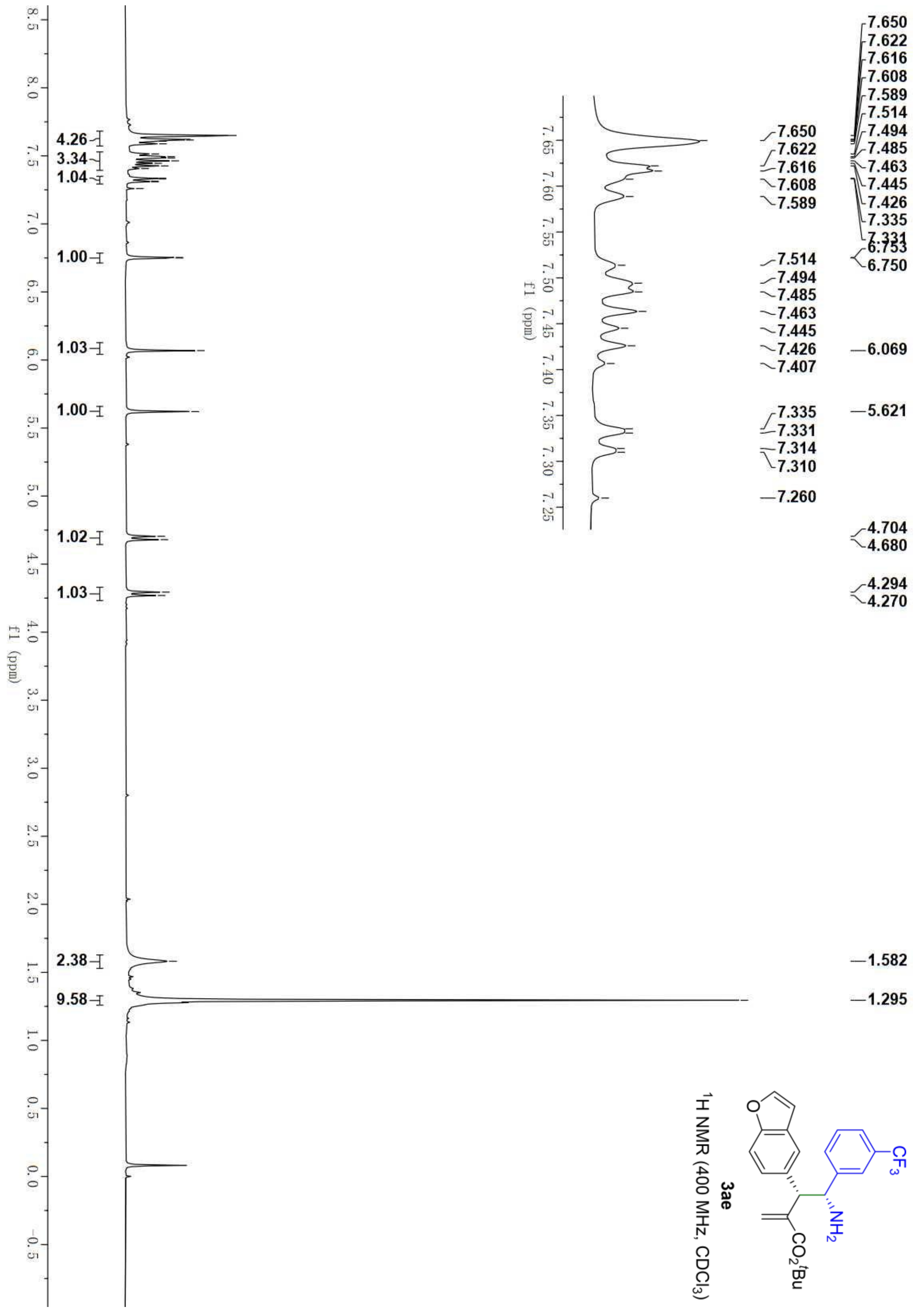
3ae
^{1}H NMR (400 MHz, CDCl$_3$)
f1 (ppm)
4.26
3.34
1.04
1.00
1.03
1.00
1.02
1.03
2.38
9.58
7.650
7.622
7.616
7.608
7.589
7.514
7.494
7.485
7.463
7.445
7.426
7.407
7.335
7.331
7.314
7.310
7.260
6.753
6.750
6.069
5.621
4.704
4.680
4.294
4.270
1.582
1.295

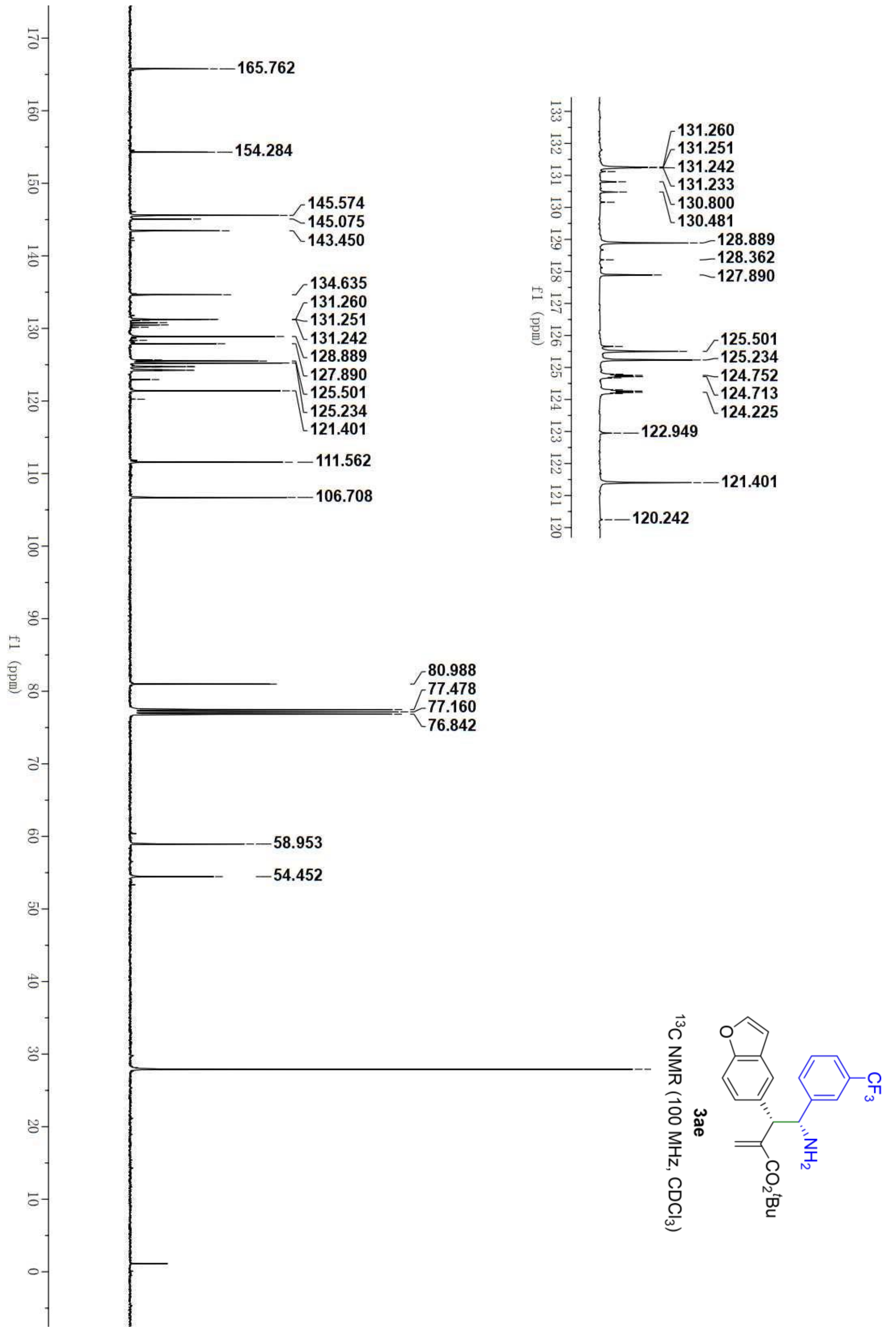
165.762
154.284
145.574
145.075
143.450
134.635
131.260
131.251
131.242
128.889
127.890
125.501
125.234
121.401
111.562
106.708
80.988
77.478
77.160
76.842
58.953
54.452
131.260
131.251
131.242
131.233
130.800
130.481
128.889
128.362
127.890
125.501
125.234
124.752
124.713
124.225
122.949
121.401
120.242
f1 (ppm)
CF3
NH2
CO2tBu
3ae
13C NMR (100 MHz, CDCl3)

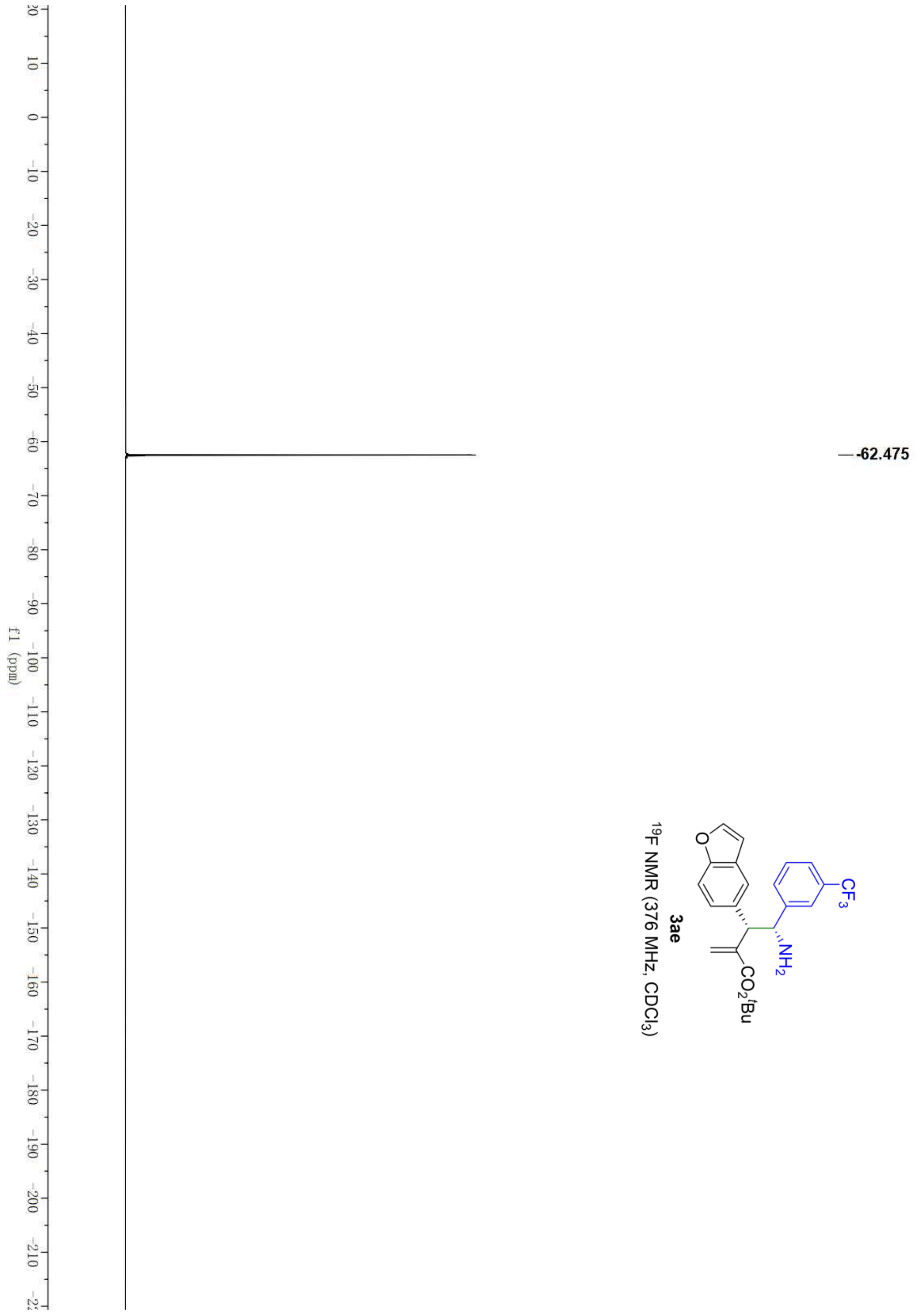
-62.475
CF3
NH2
CO2tBu
O
3ae
19F NMR (376 MHz, CDCl3)
f1 (ppm)

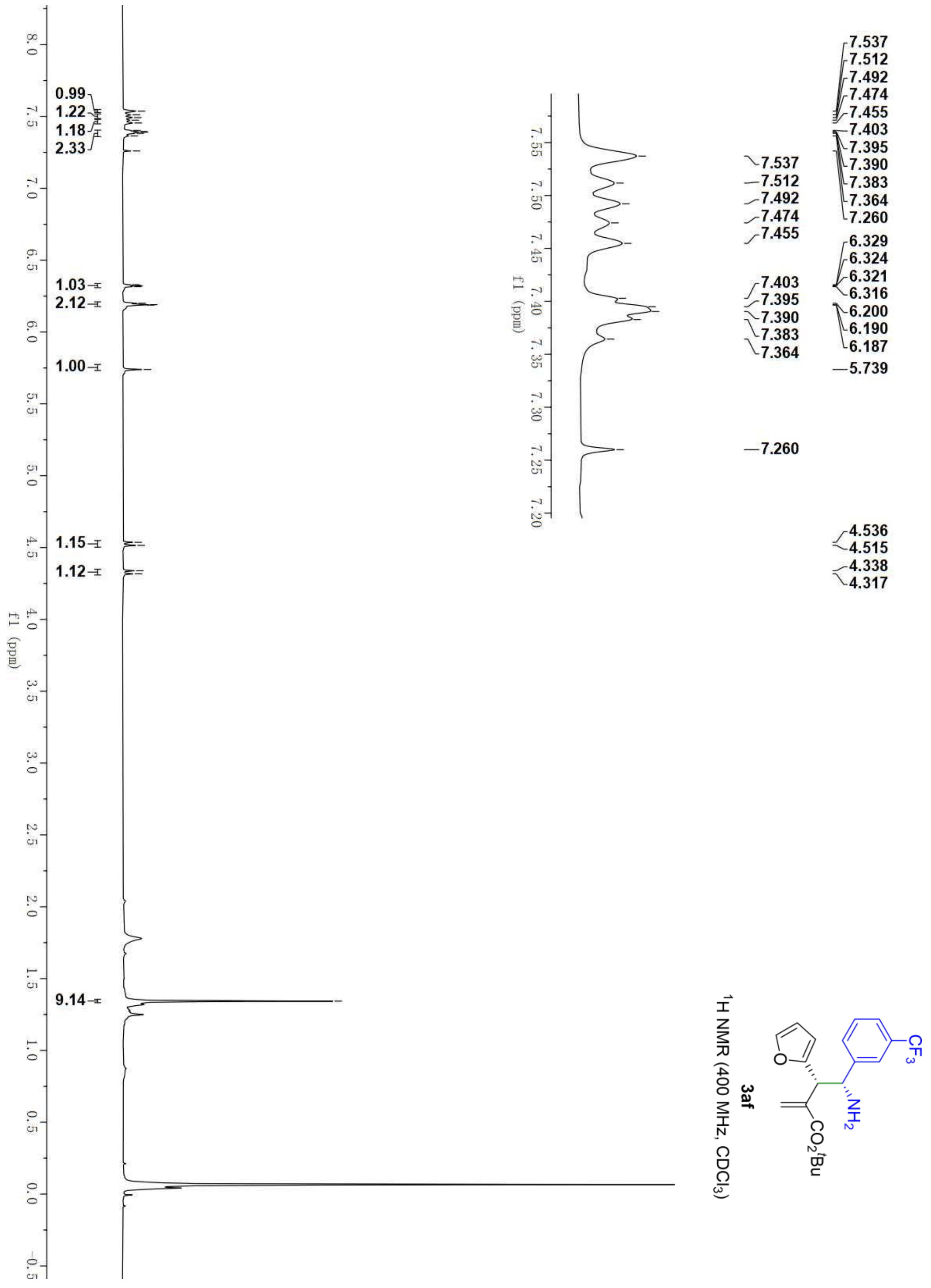

7.537
7.512
7.492
7.474
7.455
7.403
7.395
7.390
7.383
7.364
7.260
6.329
6.324
6.321
6.316
6.200
6.190
6.187
5.739
4.536
4.515
4.338
4.317
0.99
1.22
1.18
2.33
1.03
2.12
1.00
1.15
1.12
9.14
f1 (ppm)
CF3
NH2
CO2tBu
O
3af
1H NMR (400 MHz, CDCl3)

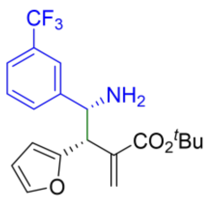

3af

^{13}C NMR (100 MHz, $CDCl_3$)

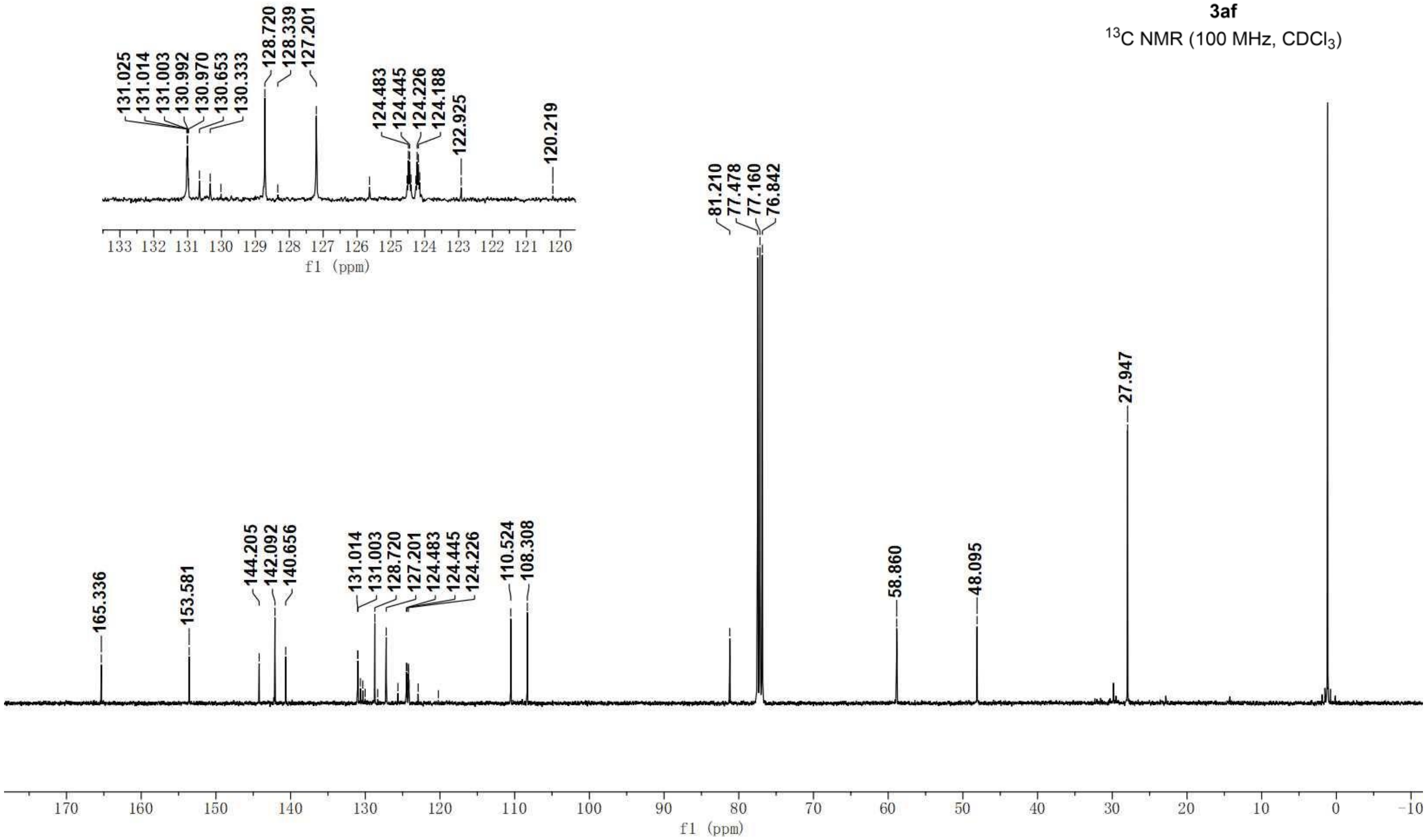

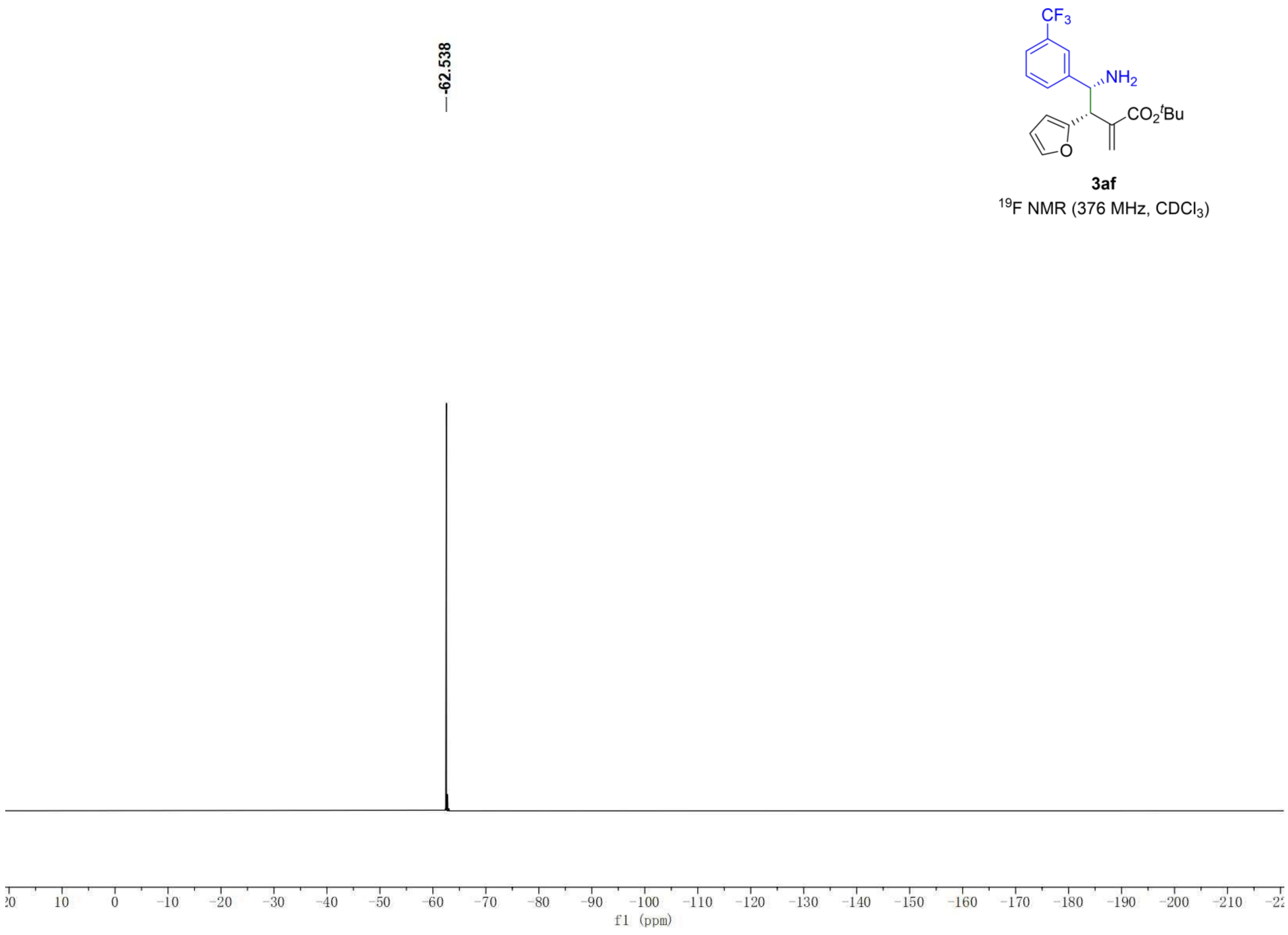

3af

^{19}F NMR (376 MHz, $CDCl_3$)

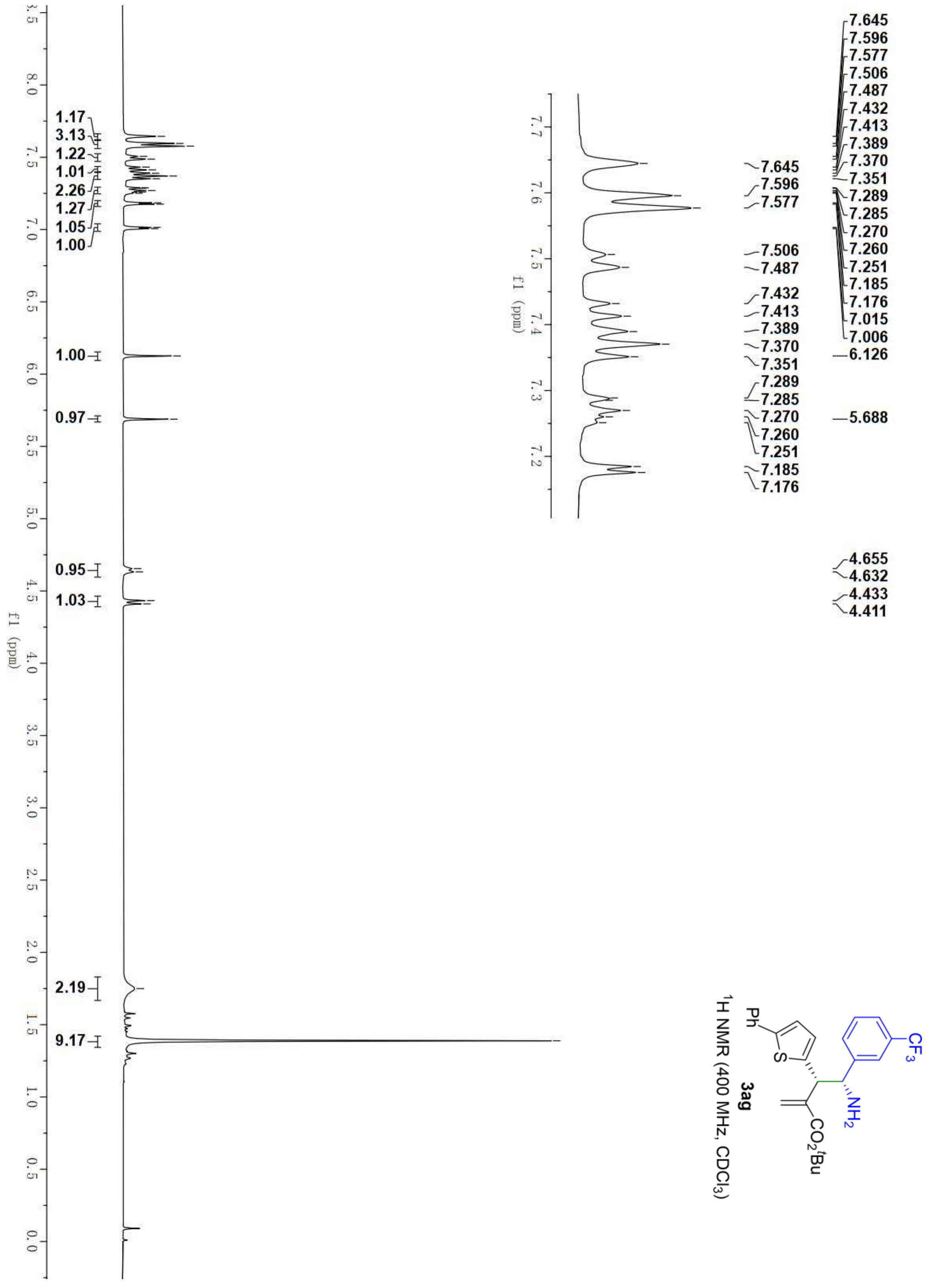
7.645
7.596
7.577
7.506
7.487
7.432
7.413
7.389
7.370
7.351
7.289
7.285
7.270
7.260
7.251
7.185
7.176
7.015
7.006
6.126
5.688
4.655
4.632
4.433
4.411
1.17
3.13
1.22
1.01
2.26
1.27
1.05
1.00
1.00
0.97
0.95
1.03
2.19
9.17
f1 (ppm)
CF3
NH2
CO2tBu
Ph
S
3ag
1H NMR (400 MHz, CDCl3)

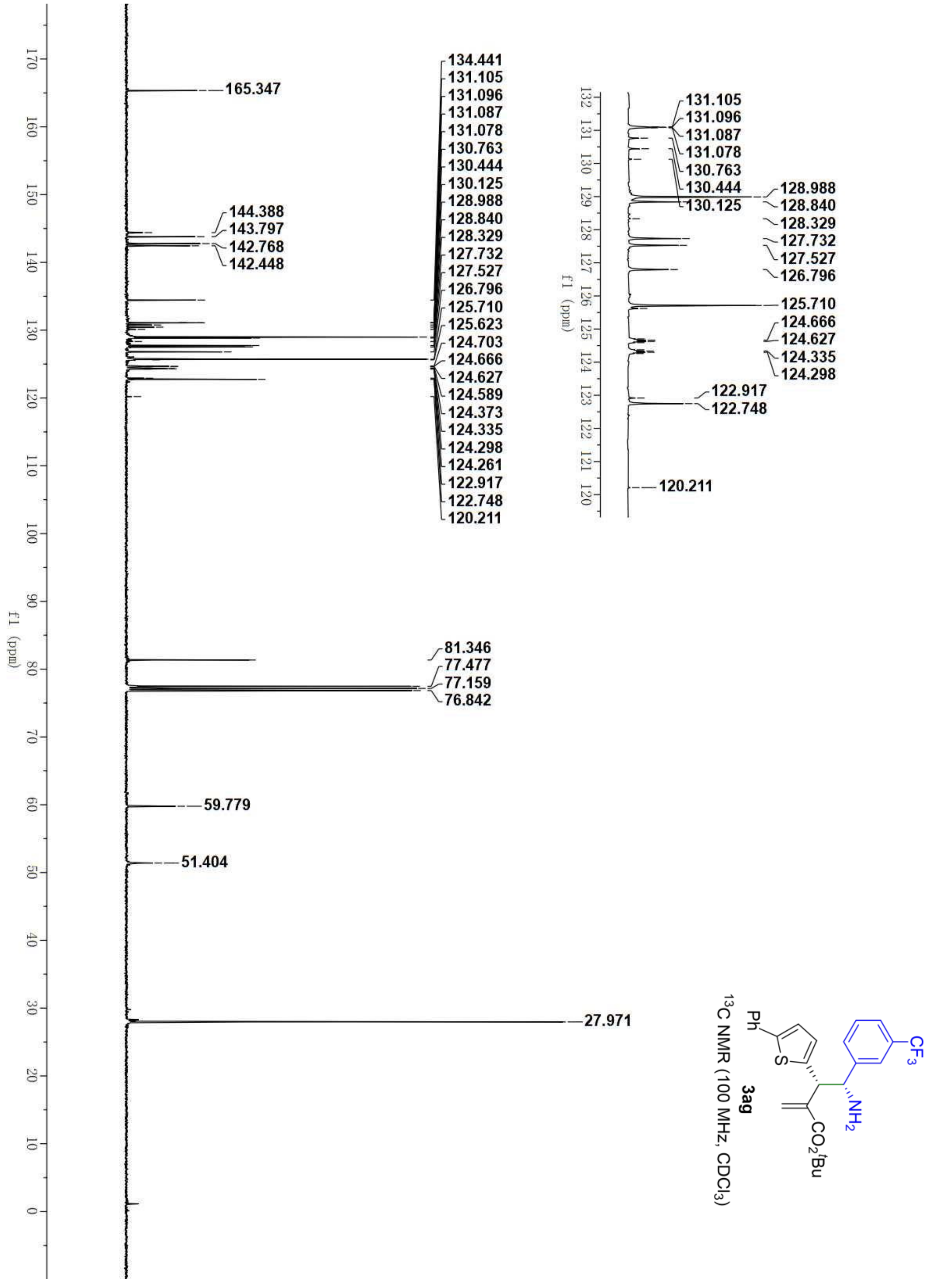

165.347
144.388
143.797
142.768
142.448
134.441
131.105
131.096
131.087
131.078
130.763
130.444
130.125
128.988
128.840
128.329
127.732
127.527
126.796
125.710
125.623
124.703
124.666
124.627
124.589
124.373
124.335
124.298
124.261
122.917
122.748
120.211
81.346
77.477
77.159
76.842
59.779
51.404
27.971
f1 (ppm)
CF3
NH2
CO2tBu
Ph
S
3ag
13C NMR (100 MHz, CDCl3)

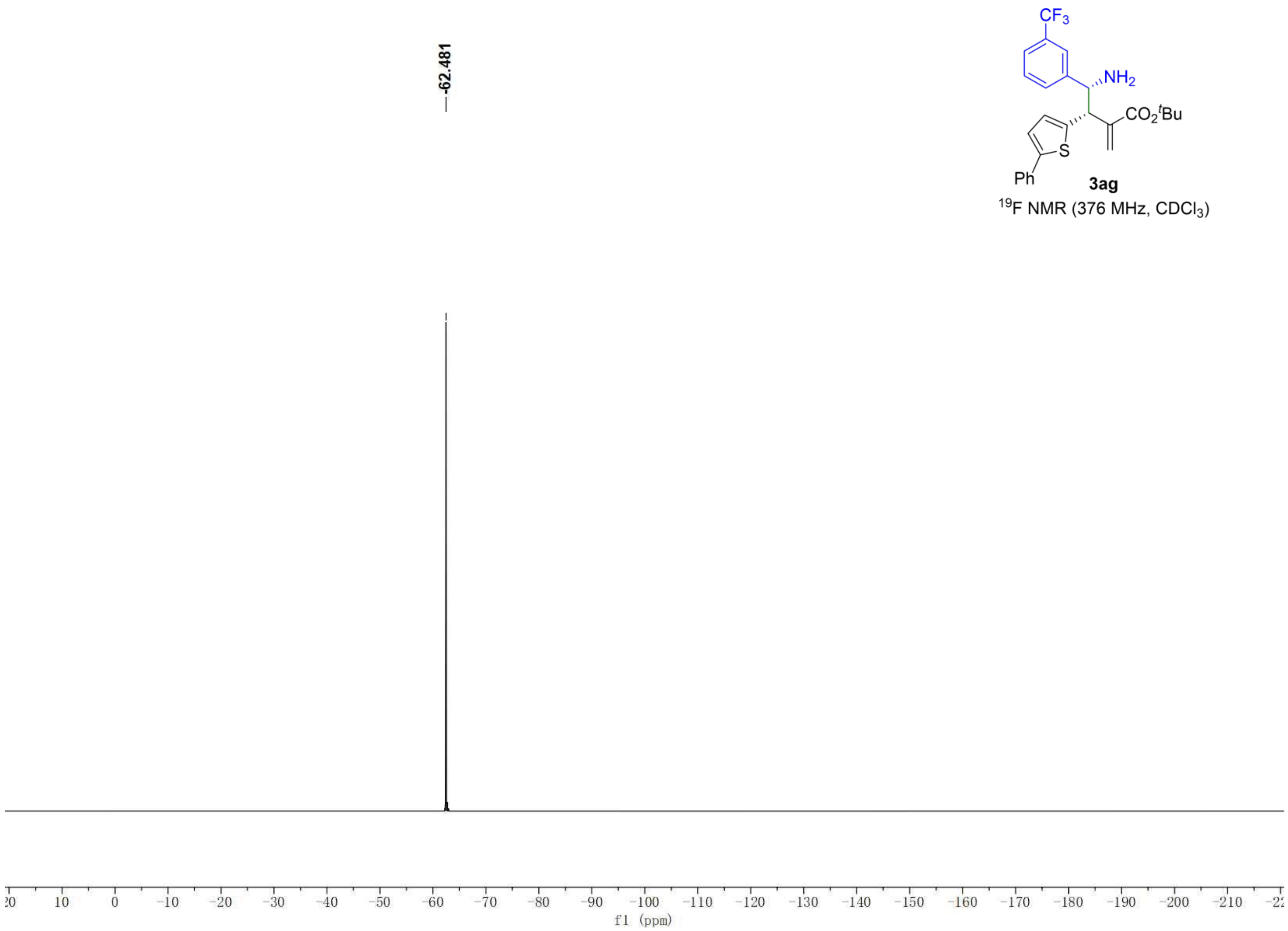

3ag

^{19}F NMR (376 MHz, $CDCl_3$)

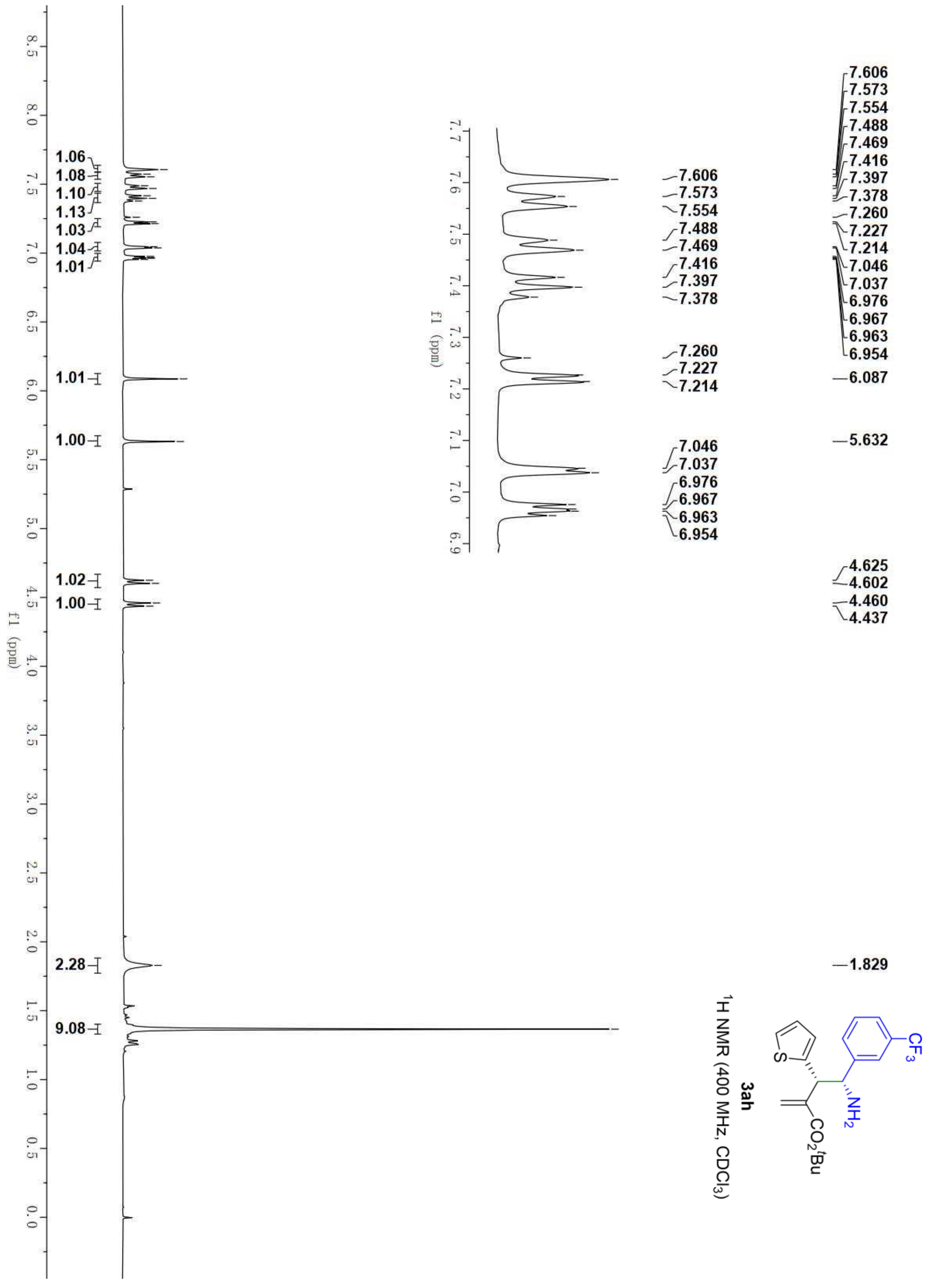

7.606
7.573
7.554
7.488
7.469
7.416
7.397
7.378
7.260
7.227
7.214
7.046
7.037
6.976
6.967
6.963
6.954
6.087
5.632
4.625
4.602
4.460
4.437
1.829
1.06
1.08
1.10
1.13
1.03
1.04
1.01
1.01
1.00
1.02
1.00
2.28
9.08
f1 (ppm)
CF3
NH2
S
CO2tBu
3ah
1H NMR (400 MHz, CDCl3)

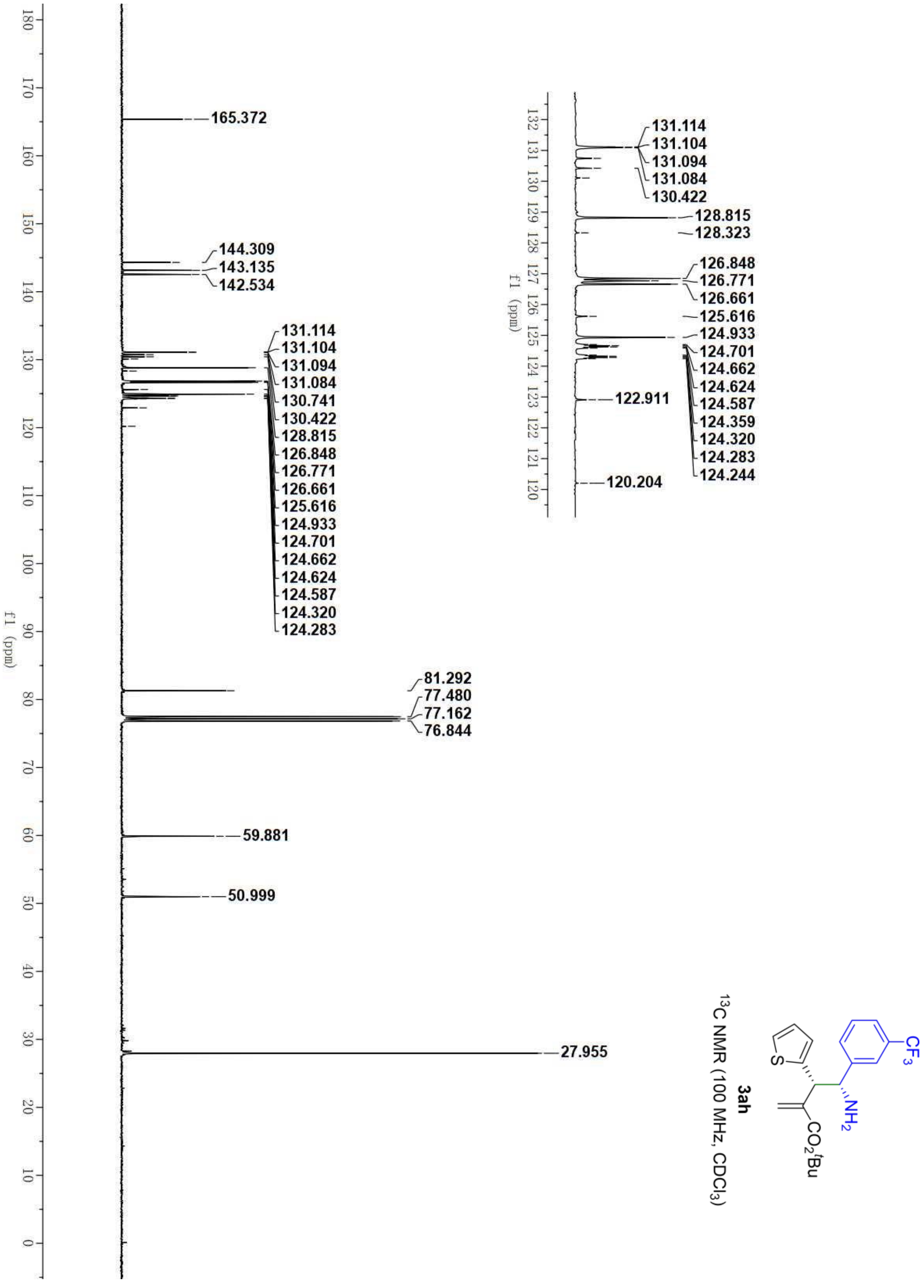
165.372
144.309
143.135
142.534
131.114
131.104
131.094
131.084
130.741
130.422
128.815
126.848
126.771
126.661
125.616
124.933
124.701
124.662
124.624
124.587
124.320
124.283
81.292
77.480
77.162
76.844
59.881
50.999
27.955
131.114
131.104
131.094
131.084
130.422
128.815
128.323
126.848
126.771
126.661
125.616
124.933
124.701
124.662
124.624
124.587
124.359
124.320
124.283
124.244
122.911
120.204
f1 (ppm)
CF3
NH2
S
CO2tBu
3ah
13C NMR (100 MHz, CDCl3)

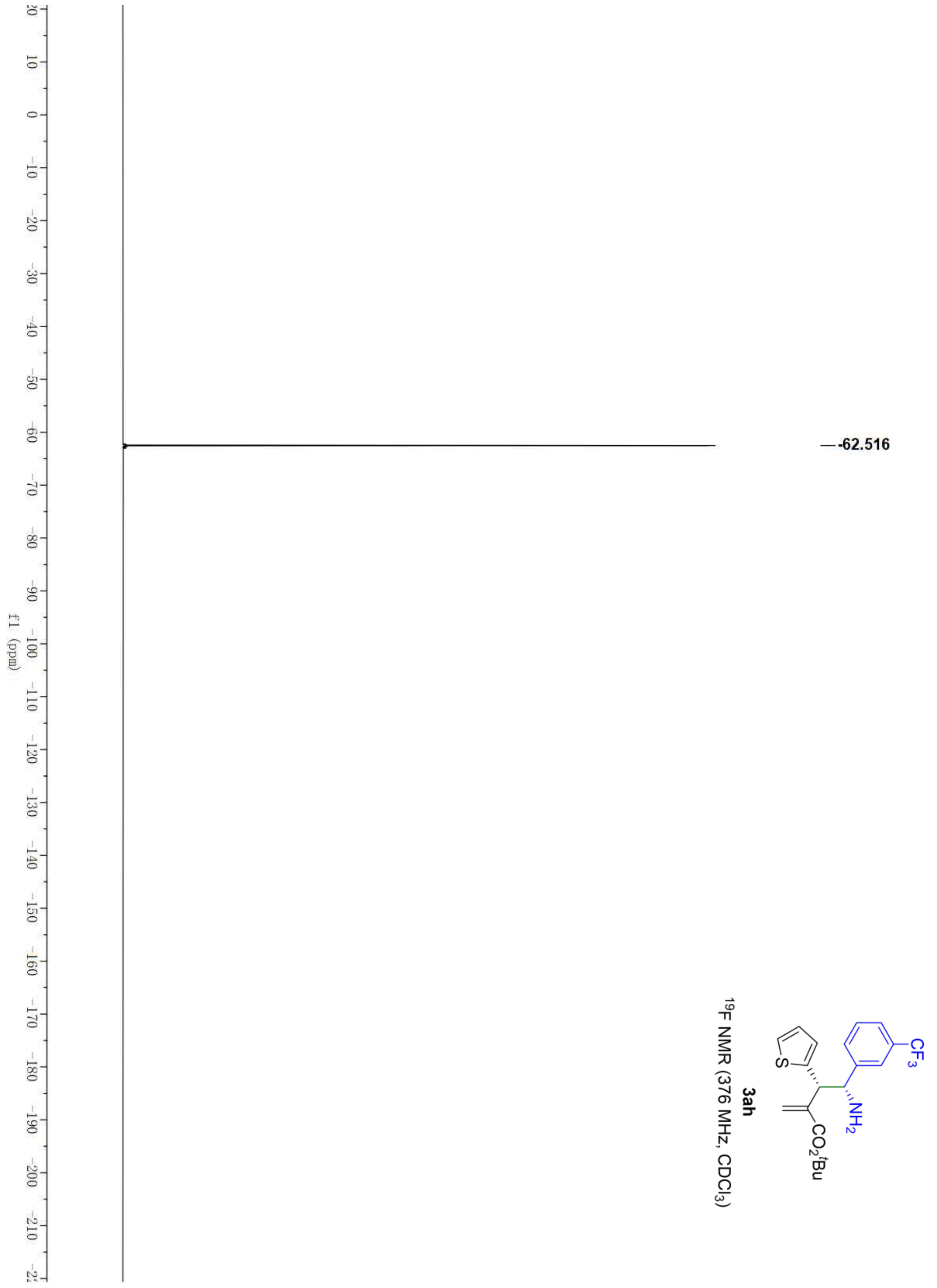
-62.516
CF3
NH2
CO2tBu
S
3ah
19F NMR (376 MHz, CDCl3)
f1 (ppm)

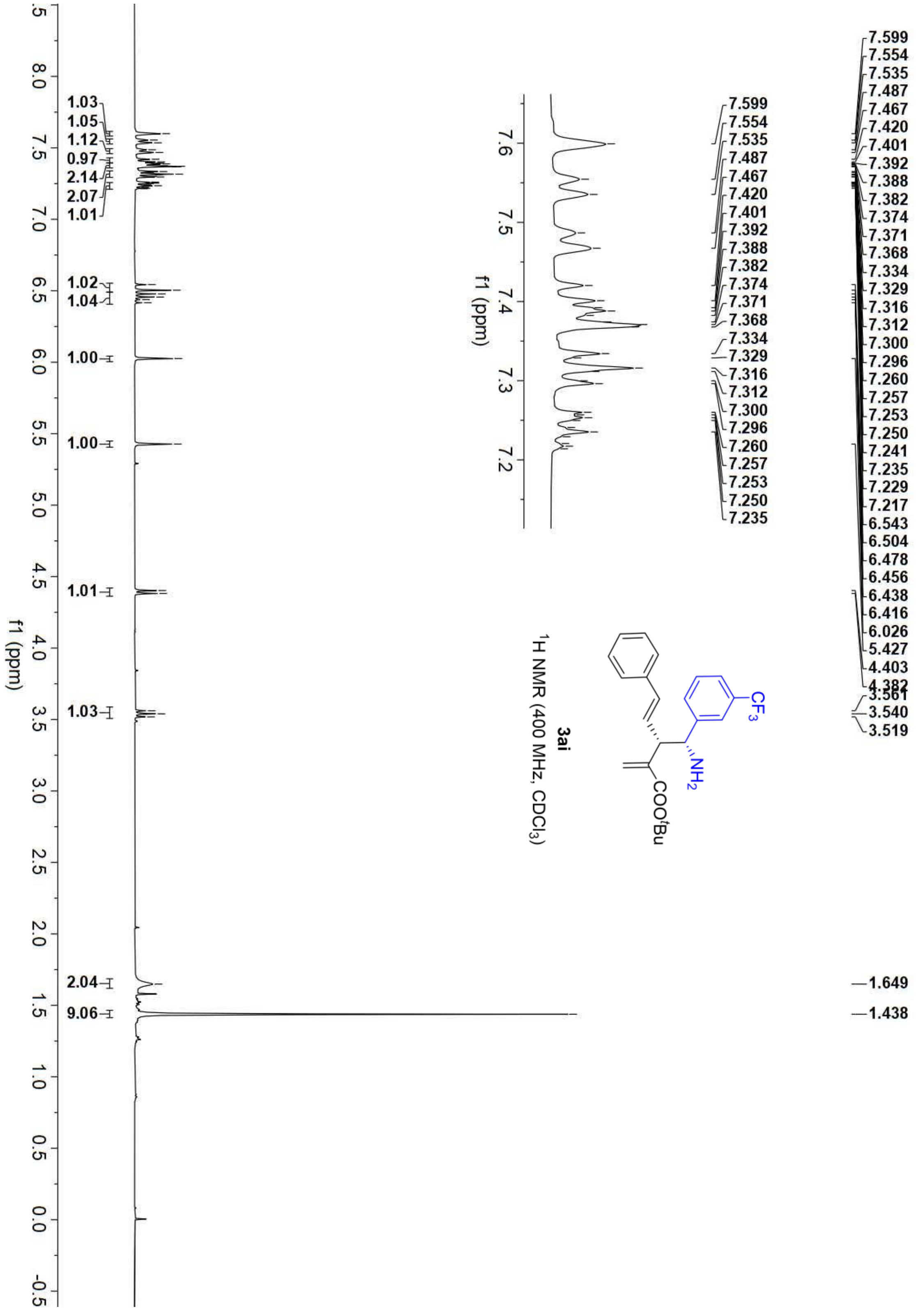
3ai
1H NMR (400 MHz, CDCl3)
COOtBu
NH2
CF3
f1 (ppm)

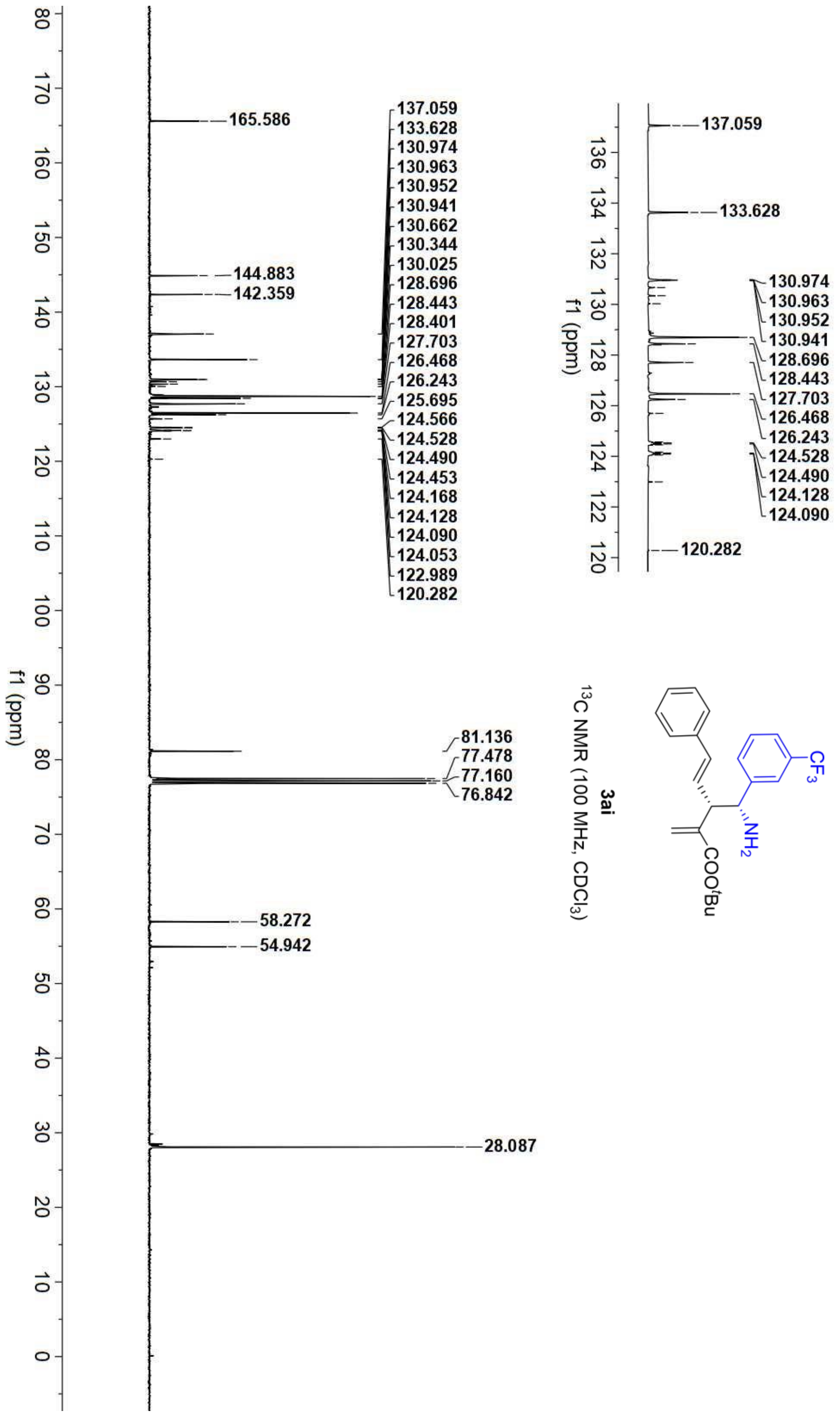
165.586
144.883
142.359
137.059
133.628
130.974
130.963
130.952
130.941
130.662
130.344
130.025
128.696
128.443
128.401
127.703
126.468
126.243
125.695
124.566
124.528
124.490
124.453
124.168
124.128
124.090
124.053
122.989
120.282
81.136
77.478
77.160
76.842
58.272
54.942
28.087
f1 (ppm)
CF3
NH2
COOtBu
3ai
13C NMR (100 MHz, CDCl3)

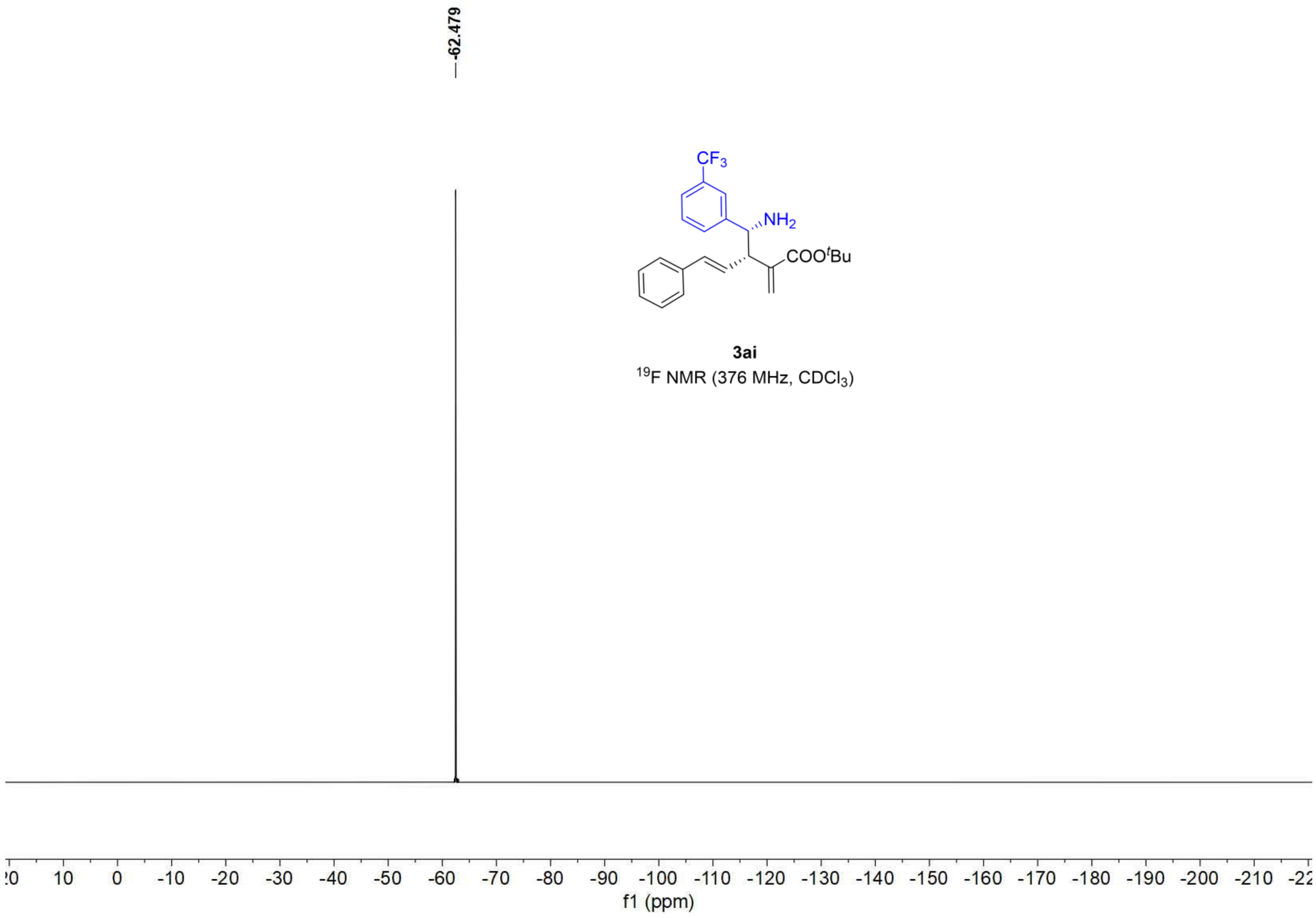

3ai

^{19}F NMR (376 MHz, $CDCl_3$)

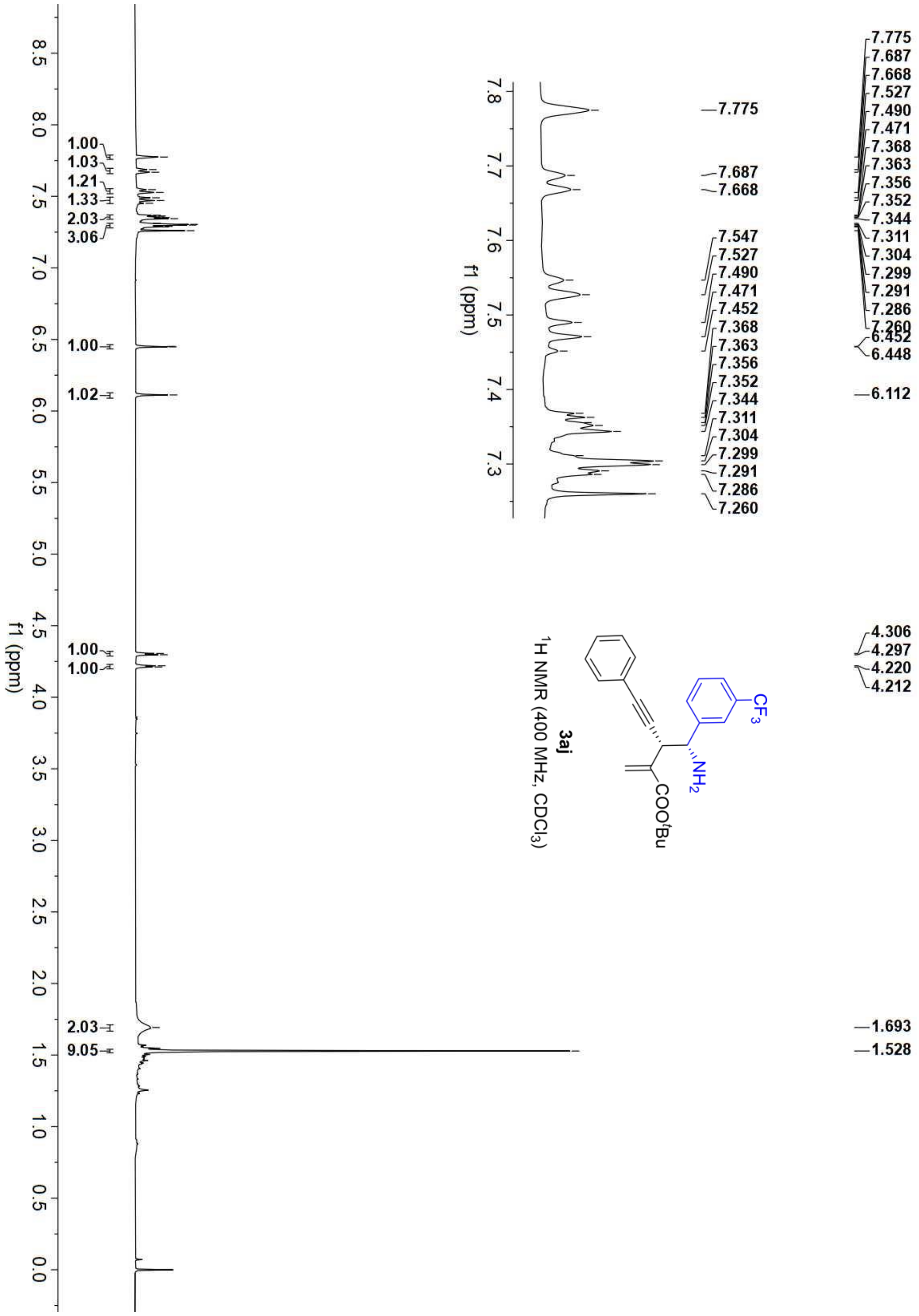

3aj

^{1}H NMR (400 MHz, $CDCl_3$)

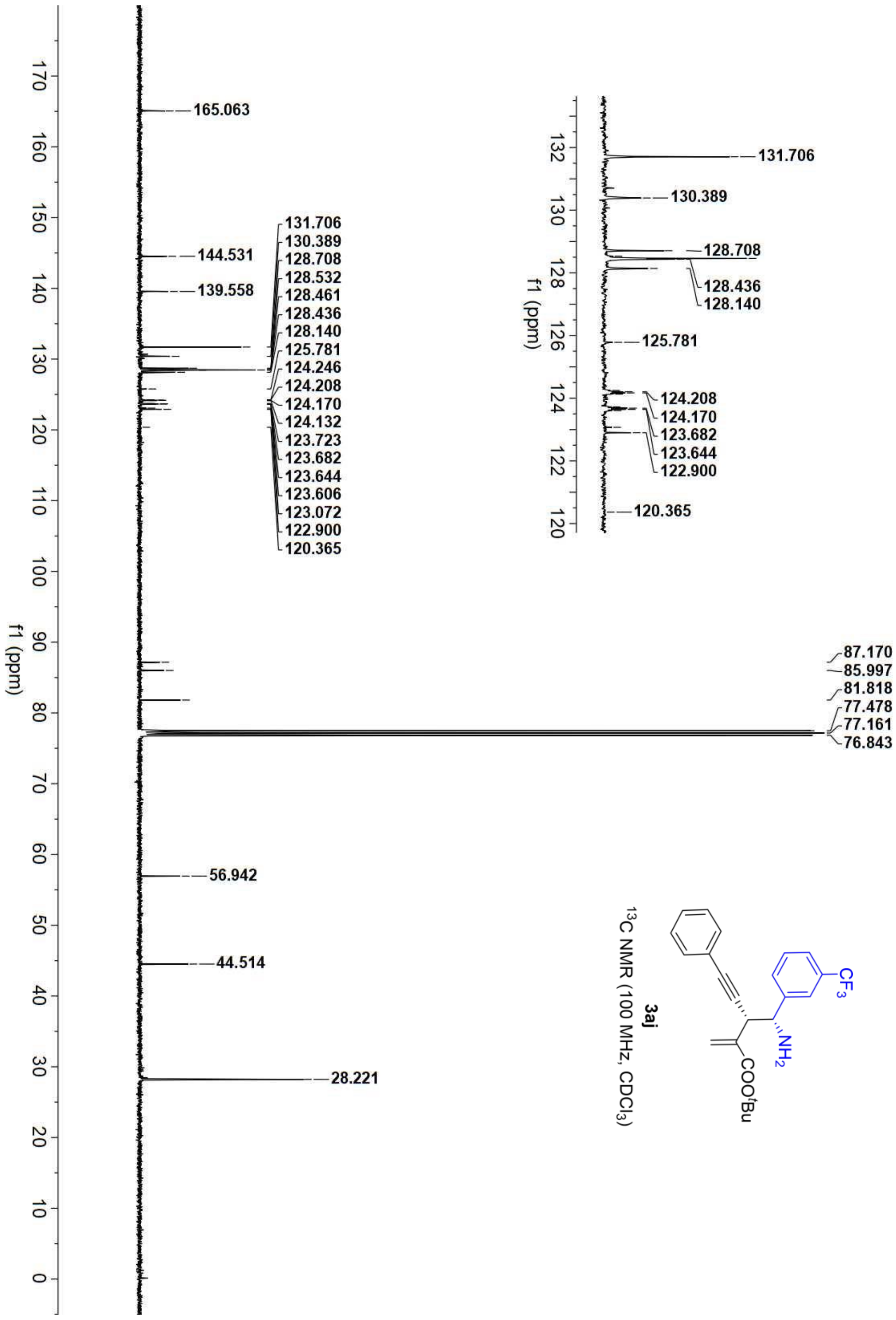
165.063
144.531
139.558
131.706
130.389
128.708
128.532
128.461
128.436
128.140
125.781
124.246
124.208
124.170
124.132
123.723
123.682
123.644
123.606
123.072
122.900
120.365
87.170
85.997
81.818
77.478
77.161
76.843
56.942
44.514
28.221
f1 (ppm)
CF3
NH2
COO^tBu
3aj
13C NMR (100 MHz, CDCl3)

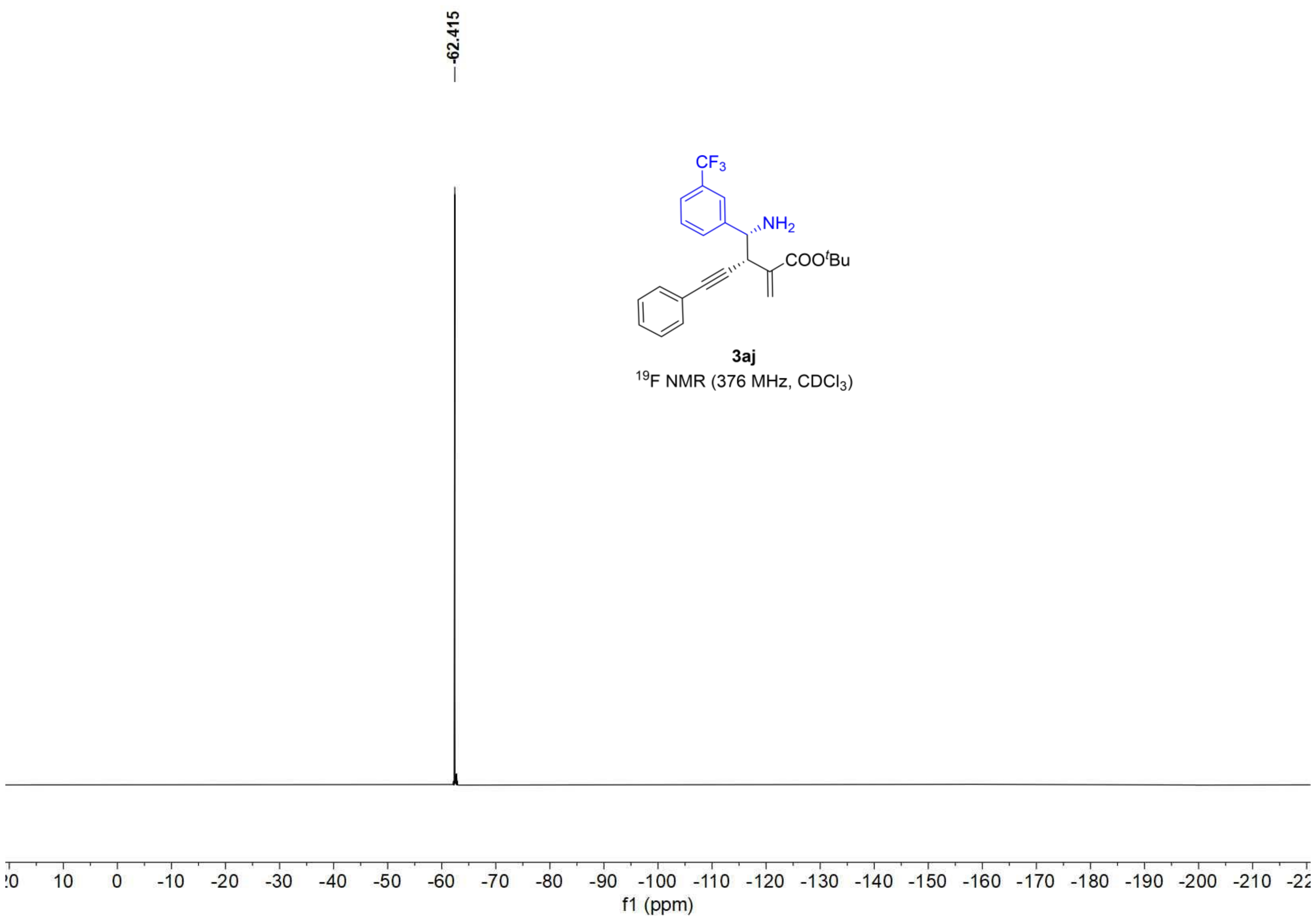
-62.415
CF3
NH2
COOtBu
3aj
19F NMR (376 MHz, CDCl3)
20 10 0 -10 -20 -30 -40 -50 -60 -70 -80 -90 -100 -110 -120 -130 -140 -150 -160 -170 -180 -190 -200 -210 -22
f1 (ppm)

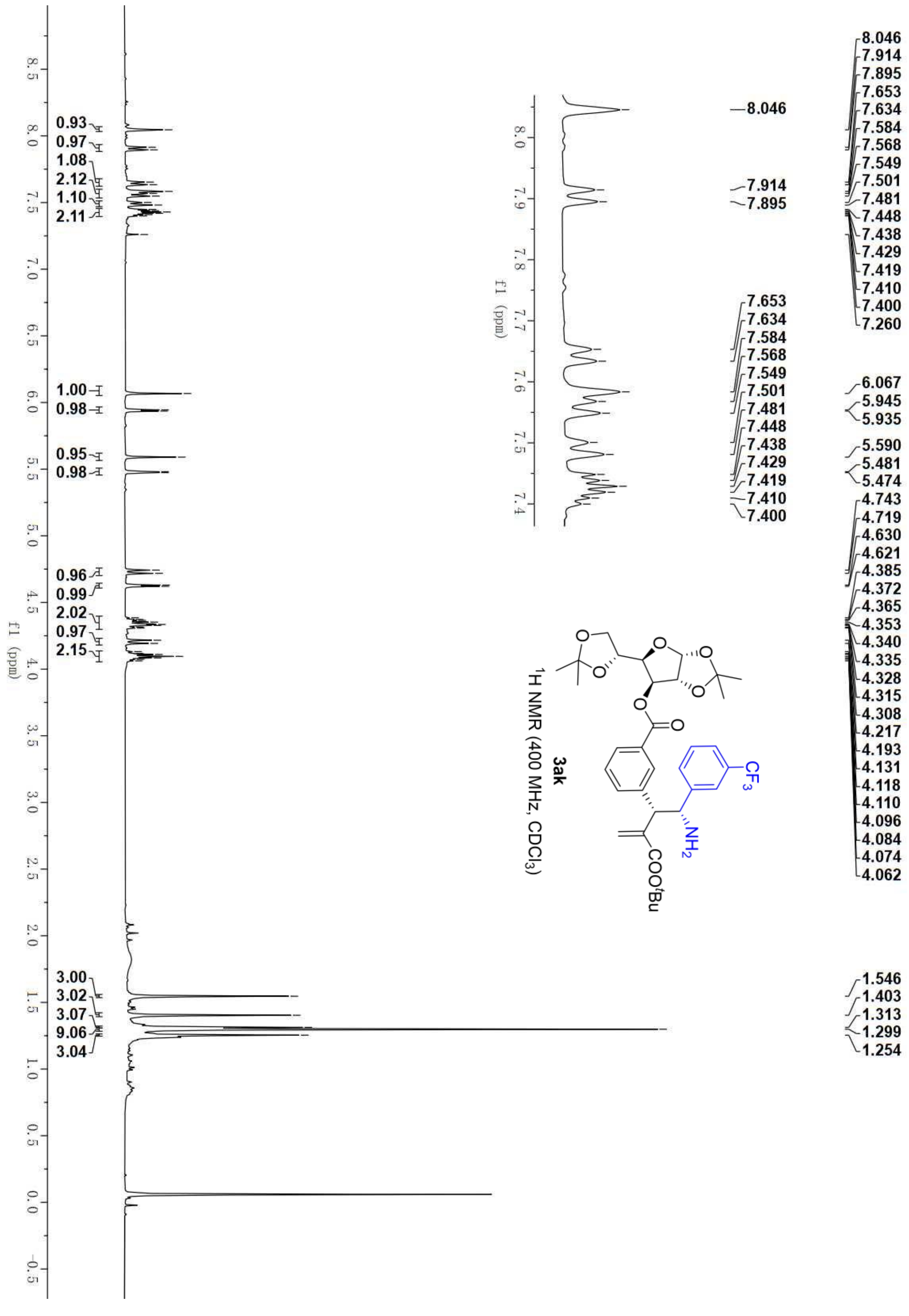
8.046
7.914
7.895
7.653
7.634
7.584
7.568
7.549
7.501
7.481
7.448
7.438
7.429
7.419
7.410
7.400
7.260
6.067
5.945
5.935
5.590
5.481
5.474
4.743
4.719
4.630
4.621
4.385
4.372
4.365
4.353
4.340
4.335
4.328
4.315
4.308
4.217
4.193
4.131
4.118
4.110
4.096
4.084
4.074
4.062
1.546
1.403
1.313
1.299
1.254
CF3
NH2
COOtBu
3ak
1H NMR (400 MHz, CDCl3)
f1 (ppm)

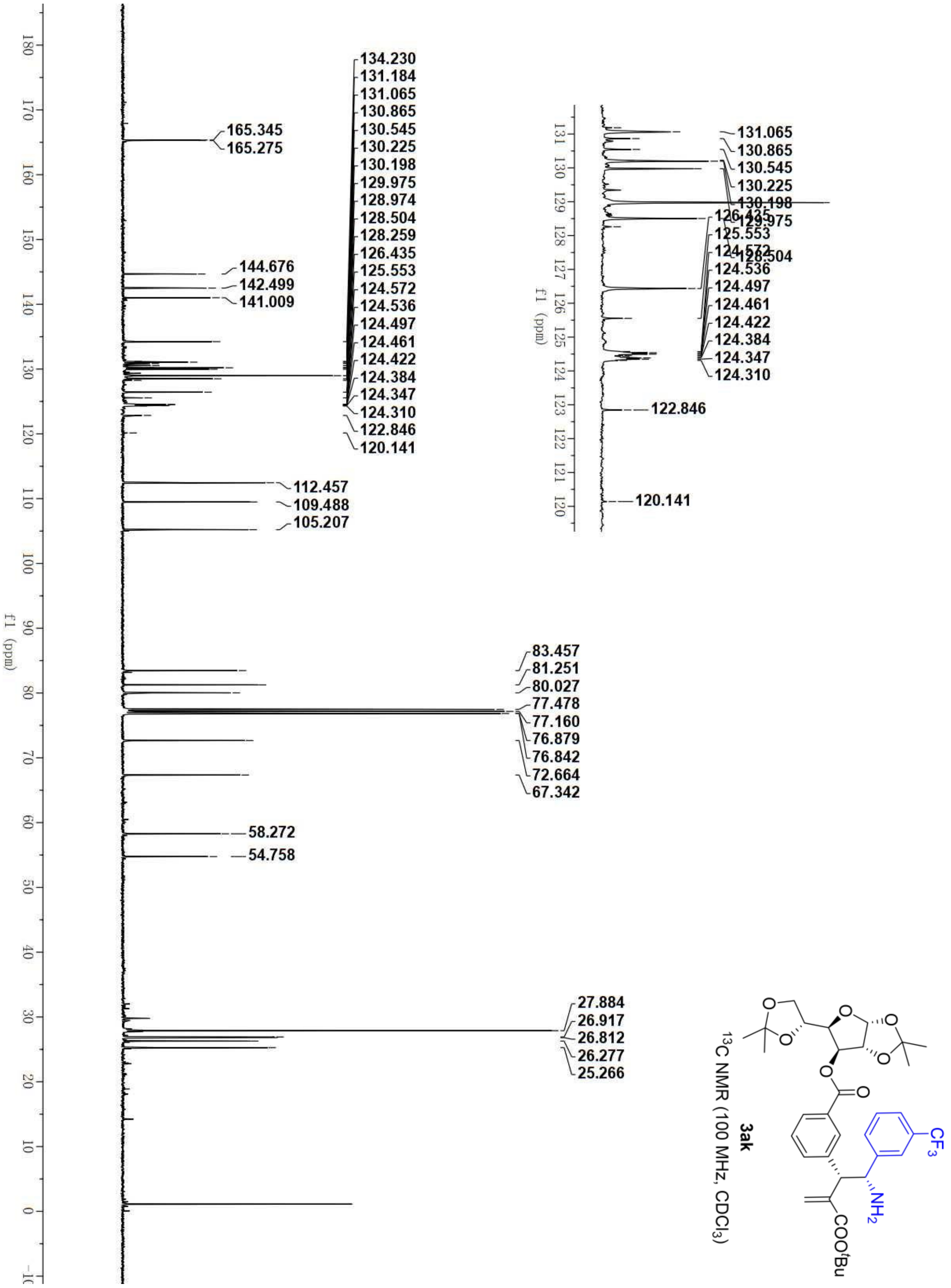

3ak
^{13}C NMR (100 MHz, CDCl$_3$)
f1 (ppm)
165.345
165.275
144.676
142.499
141.009
134.230
131.184
131.065
130.865
130.545
130.225
130.198
129.975
128.974
128.504
128.259
126.435
125.553
124.572
124.536
124.497
124.461
124.422
124.384
124.347
124.310
122.846
120.141
112.457
109.488
105.207
83.457
81.251
80.027
77.478
77.160
76.879
76.842
72.664
67.342
58.272
54.758
27.884
26.917
26.812
26.277
25.266
CF$_3$
NH$_2$
COO^{t}Bu

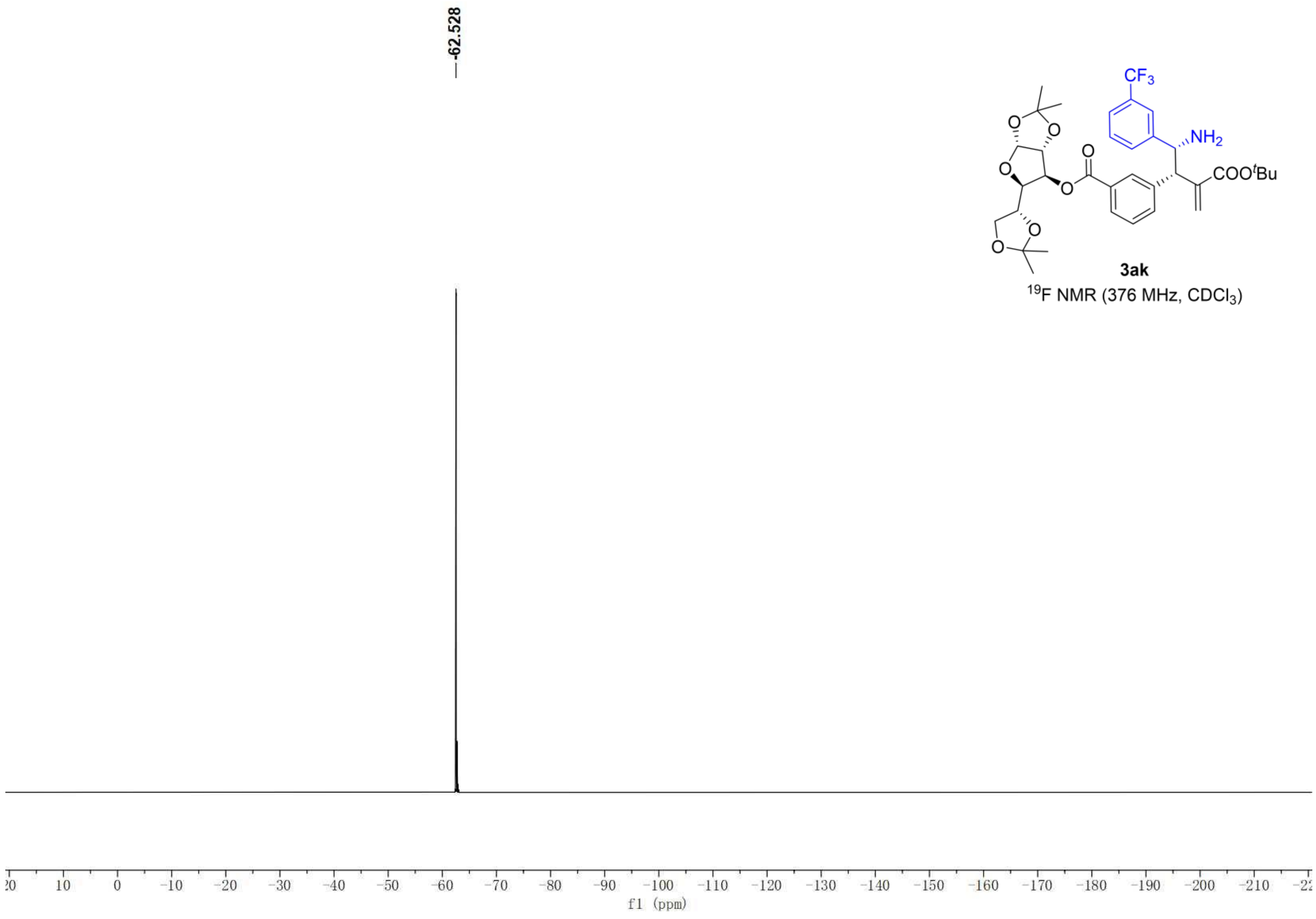
-62.528
CF3
NH2
COOtBu
O
3ak
19F NMR (376 MHz, CDCl3)
20 10 0 −10 −20 −30 −40 −50 −60 −70 −80 −90 −100 −110 −120 −130 −140 −150 −160 −170 −180 −190 −200 −210
f1 (ppm)

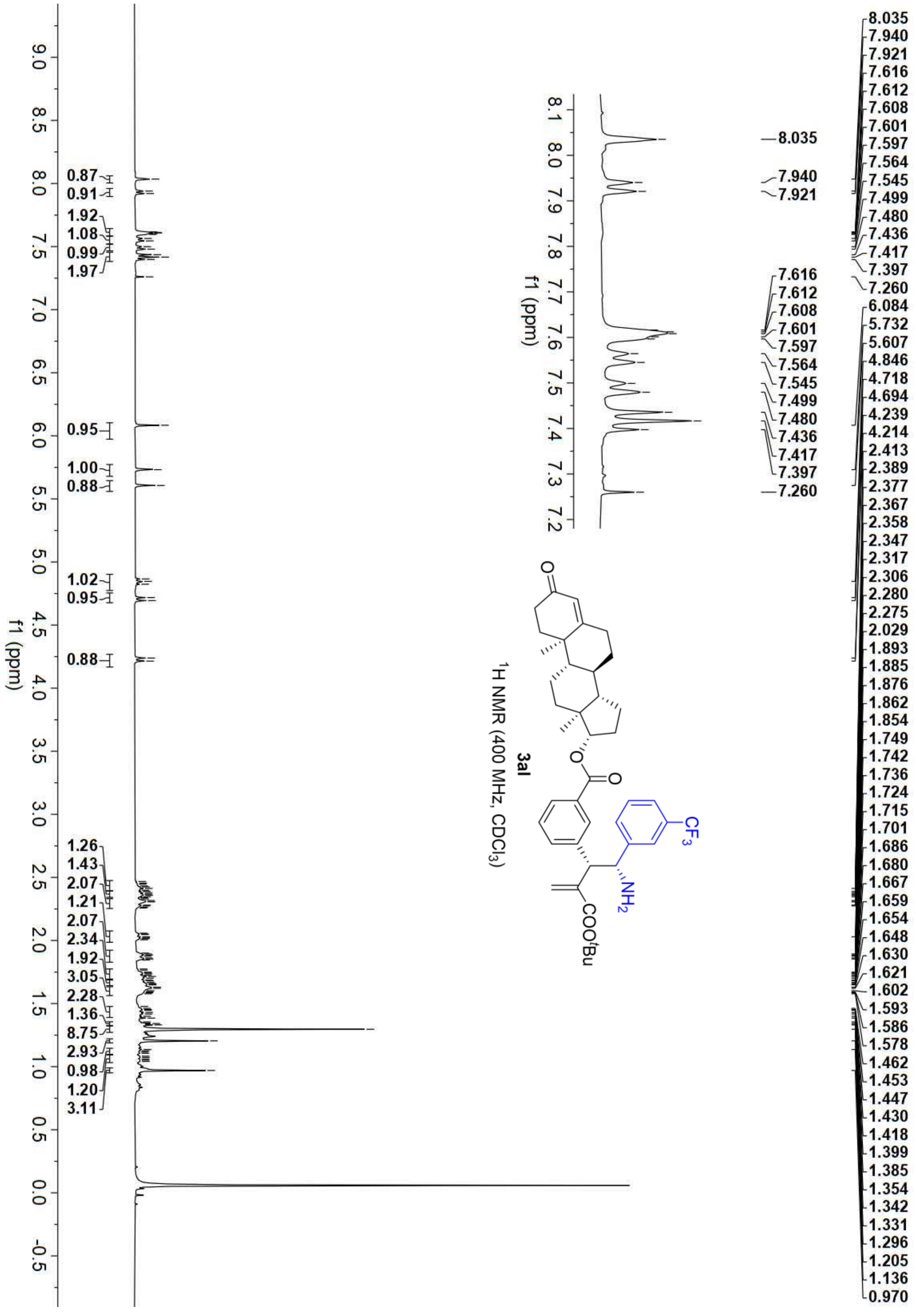
8.035
7.940
7.921
7.616
7.612
7.608
7.601
7.597
7.564
7.545
7.499
7.480
7.436
7.417
7.397
7.260
6.084
5.732
5.607
4.846
4.718
4.694
4.239
4.214
2.413
2.389
2.377
2.367
2.358
2.347
2.317
2.306
2.280
2.275
2.029
1.893
1.885
1.876
1.862
1.854
1.749
1.742
1.736
1.724
1.715
1.701
1.686
1.680
1.667
1.659
1.654
1.648
1.630
1.621
1.602
1.593
1.586
1.578
1.462
1.453
1.447
1.430
1.418
1.399
1.385
1.354
1.342
1.331
1.296
1.205
1.136
0.970
CF3
NH2
COOtBu
3al
1H NMR (400 MHz, CDCl3)
f1 (ppm)

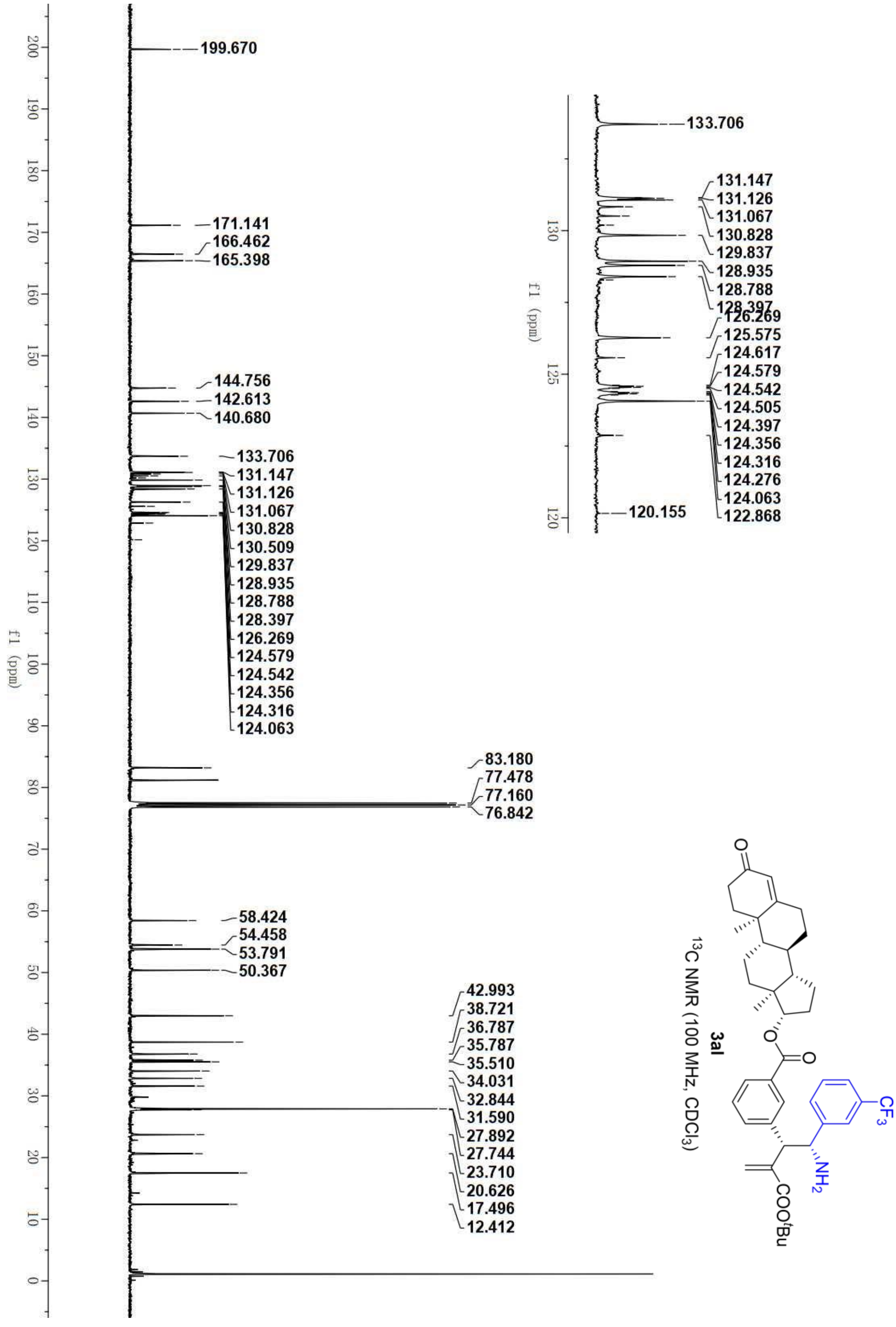

199.670
171.141
166.462
165.398
144.756
142.613
140.680
133.706
131.147
131.126
131.067
130.828
130.509
129.837
128.935
128.788
128.397
126.269
124.579
124.542
124.356
124.316
124.063
83.180
77.478
77.160
76.842
58.424
54.458
53.791
50.367
42.993
38.721
36.787
35.787
35.510
34.031
32.844
31.590
27.892
27.744
23.710
20.626
17.496
12.412
133.706
131.147
131.126
131.067
130.828
129.837
128.935
128.788
128.397
126.269
125.575
124.617
124.579
124.542
124.505
124.397
124.356
124.316
124.276
124.063
122.868
120.155
f1 (ppm)
3al
13C NMR (100 MHz, CDCl3)

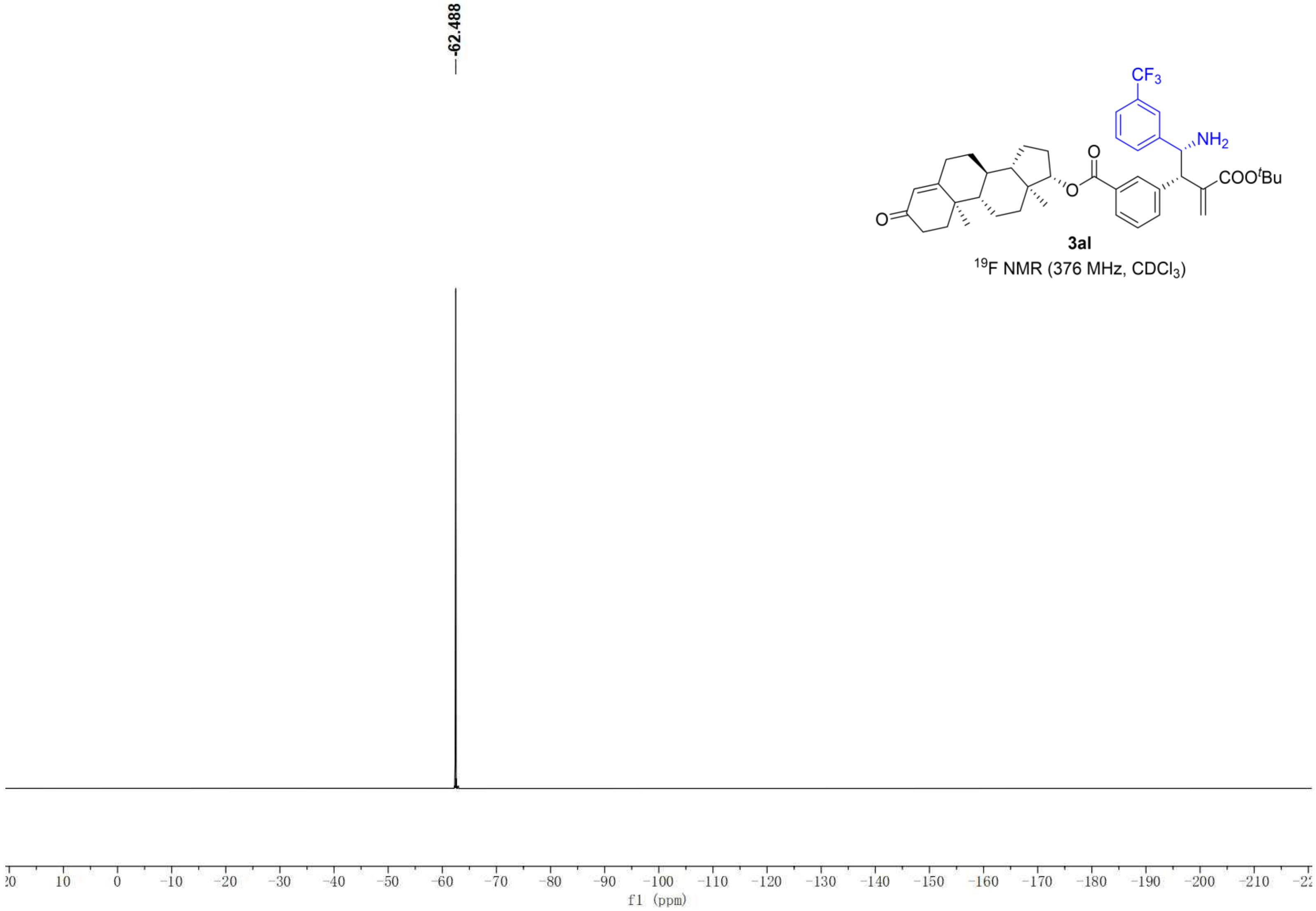
-62.488
CF3
NH2
COOtBu
O
O
O
3al
19F NMR (376 MHz, CDCl3)
f1 (ppm)

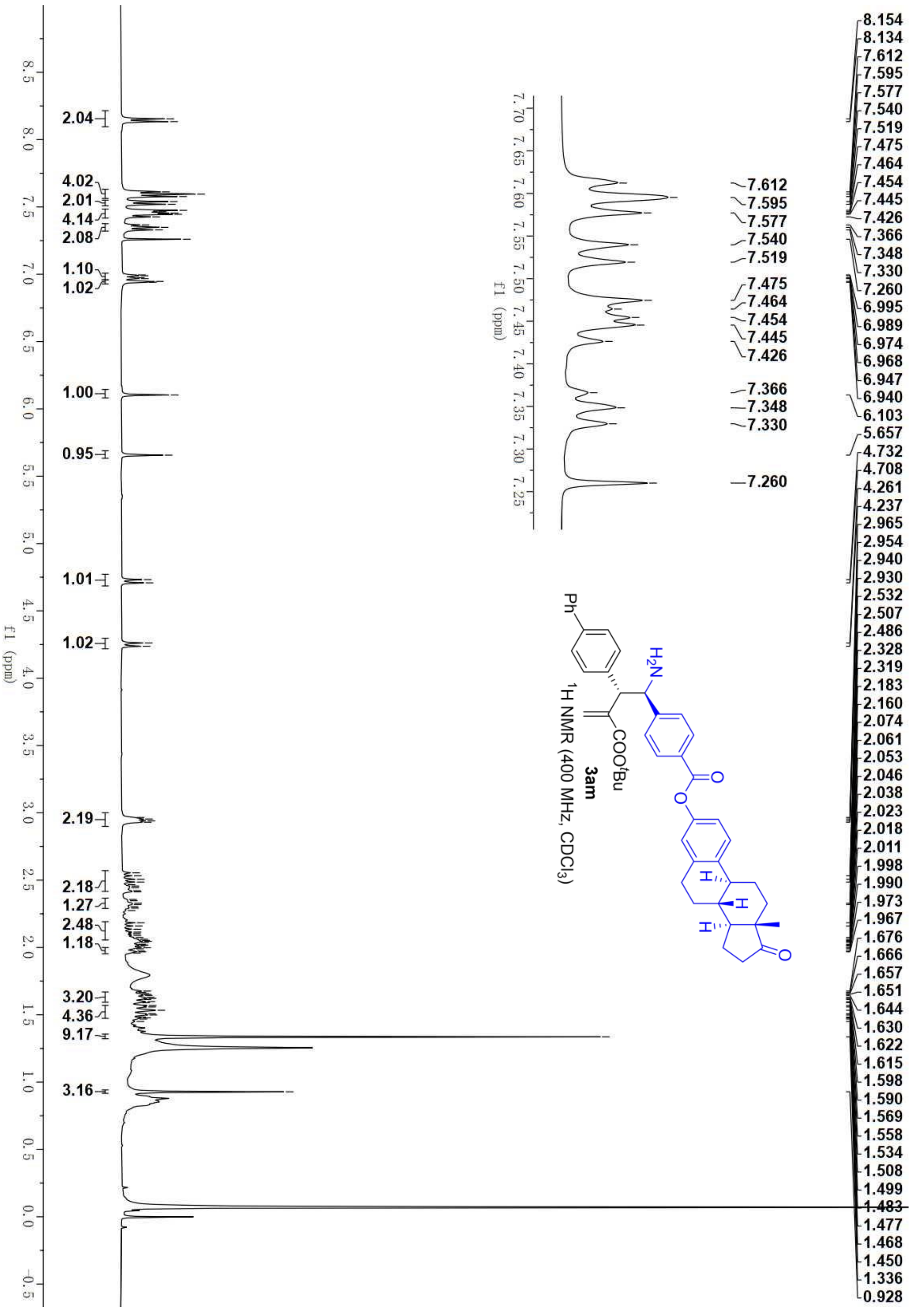

8.154
8.134
7.612
7.595
7.577
7.540
7.519
7.475
7.464
7.454
7.445
7.426
7.366
7.348
7.330
7.260
6.995
6.989
6.974
6.968
6.947
6.940
6.103
5.657
4.732
4.708
4.261
4.237
2.965
2.954
2.940
2.930
2.532
2.507
2.486
2.328
2.319
2.183
2.160
2.074
2.061
2.053
2.046
2.038
2.023
2.018
2.011
1.998
1.990
1.973
1.967
1.676
1.666
1.657
1.651
1.644
1.630
1.622
1.615
1.598
1.590
1.569
1.558
1.534
1.508
1.499
1.483
1.477
1.468
1.450
1.336
0.928
3am
^{1}H NMR (400 MHz, CDCl$_3$)
f1 (ppm)

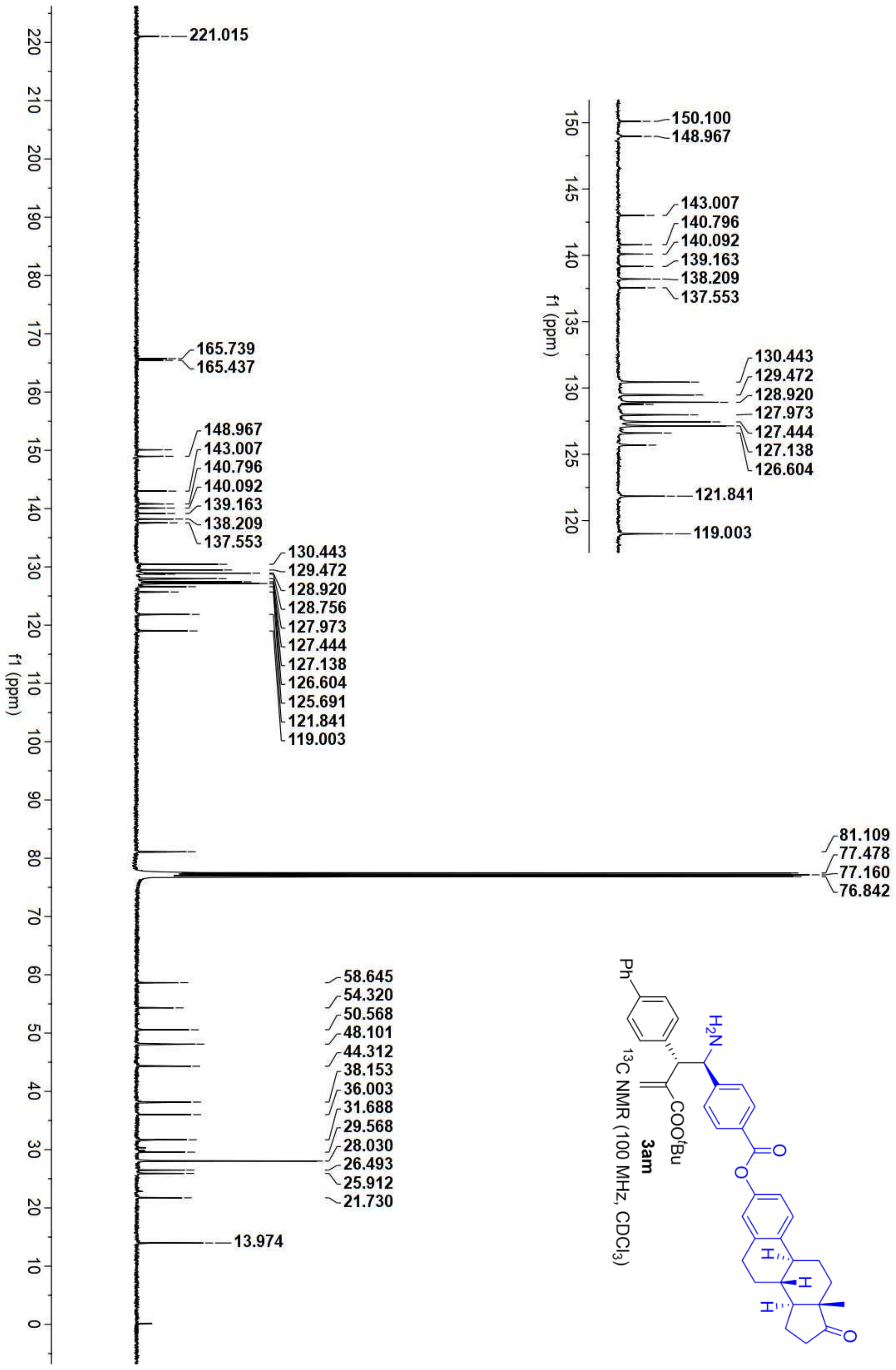

221.015
165.739
165.437
148.967
143.007
140.796
140.092
139.163
138.209
137.553
130.443
129.472
128.920
128.756
127.973
127.444
127.138
126.604
125.691
121.841
119.003
81.109
77.478
77.160
76.842
58.645
54.320
50.568
48.101
44.312
38.153
36.003
31.688
29.568
28.030
26.493
25.912
21.730
13.974
150.100
f1 (ppm)
H_2N
Ph
COO^tBu
3am
^{13}C NMR (100 MHz, $CDCl_3$)

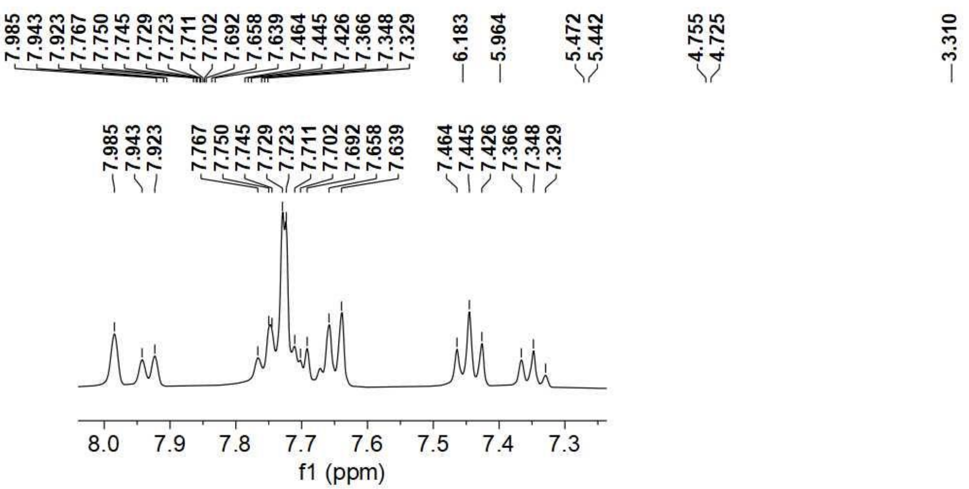

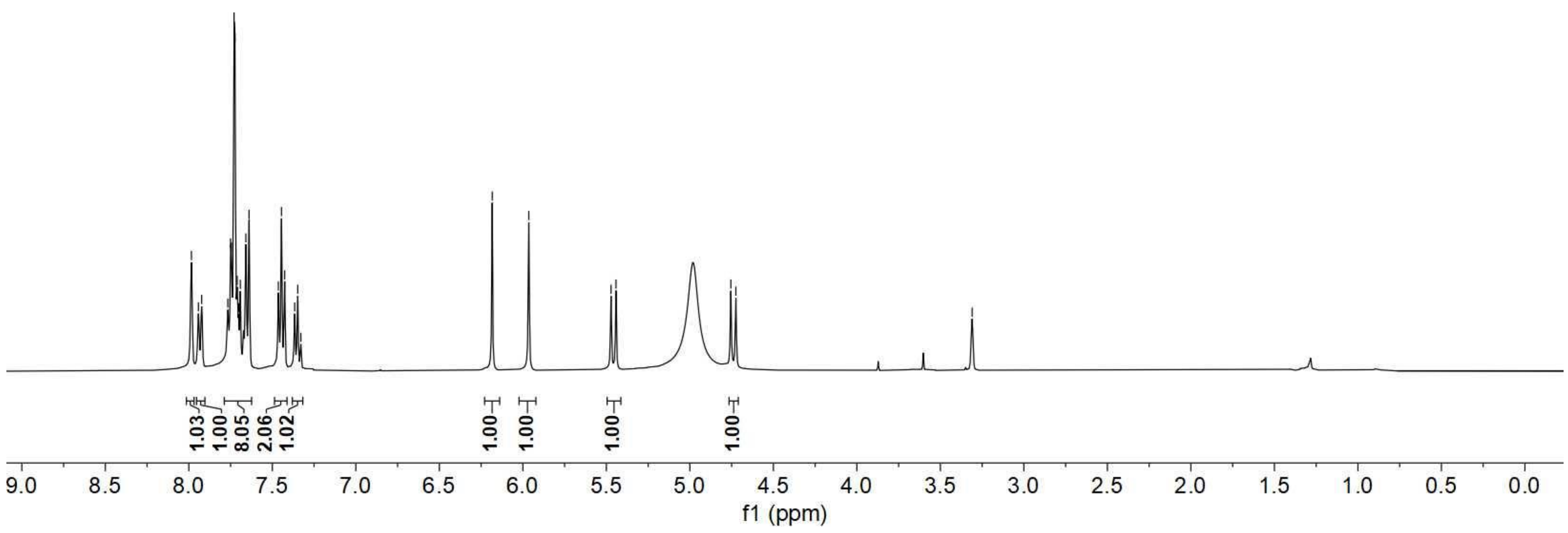

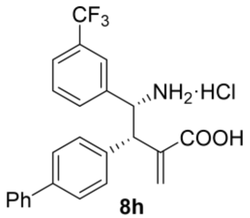

8h

^{1}H NMR (400 MHz, CD_3OD)

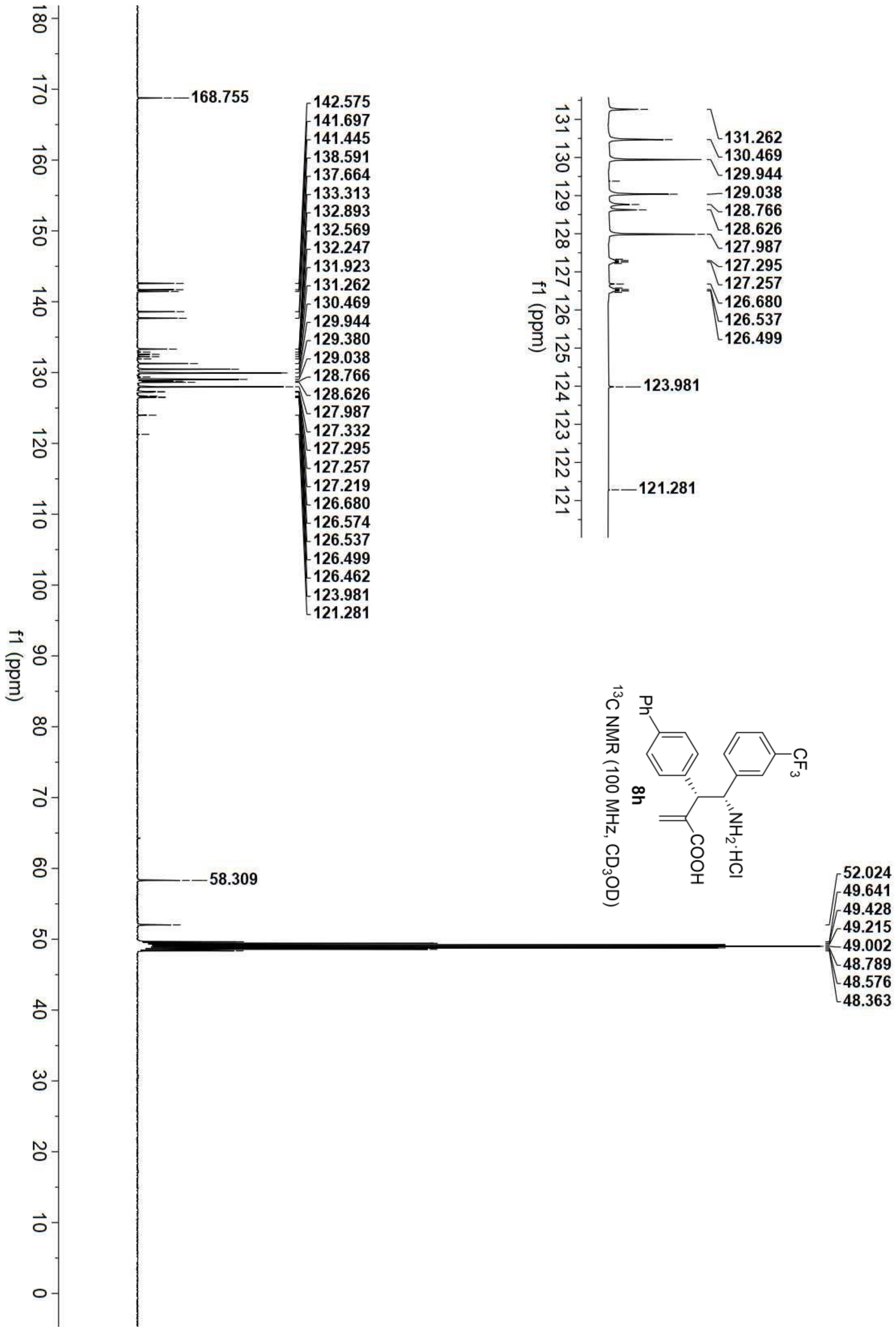

168.755
142.575
141.697
141.445
138.591
137.664
133.313
132.893
132.569
132.247
131.923
131.262
130.469
129.944
129.380
129.038
128.766
128.626
127.987
127.332
127.295
127.257
127.219
126.680
126.574
126.537
126.499
126.462
123.981
121.281
58.309
52.024
49.641
49.428
49.215
49.002
48.789
48.576
48.363
131.262
130.469
129.944
129.038
128.766
128.626
127.987
127.295
127.257
126.680
126.537
126.499
123.981
121.281
f1 (ppm)
CF3
NH2·HCl
COOH
Ph
8h
13C NMR (100 MHz, CD3OD)

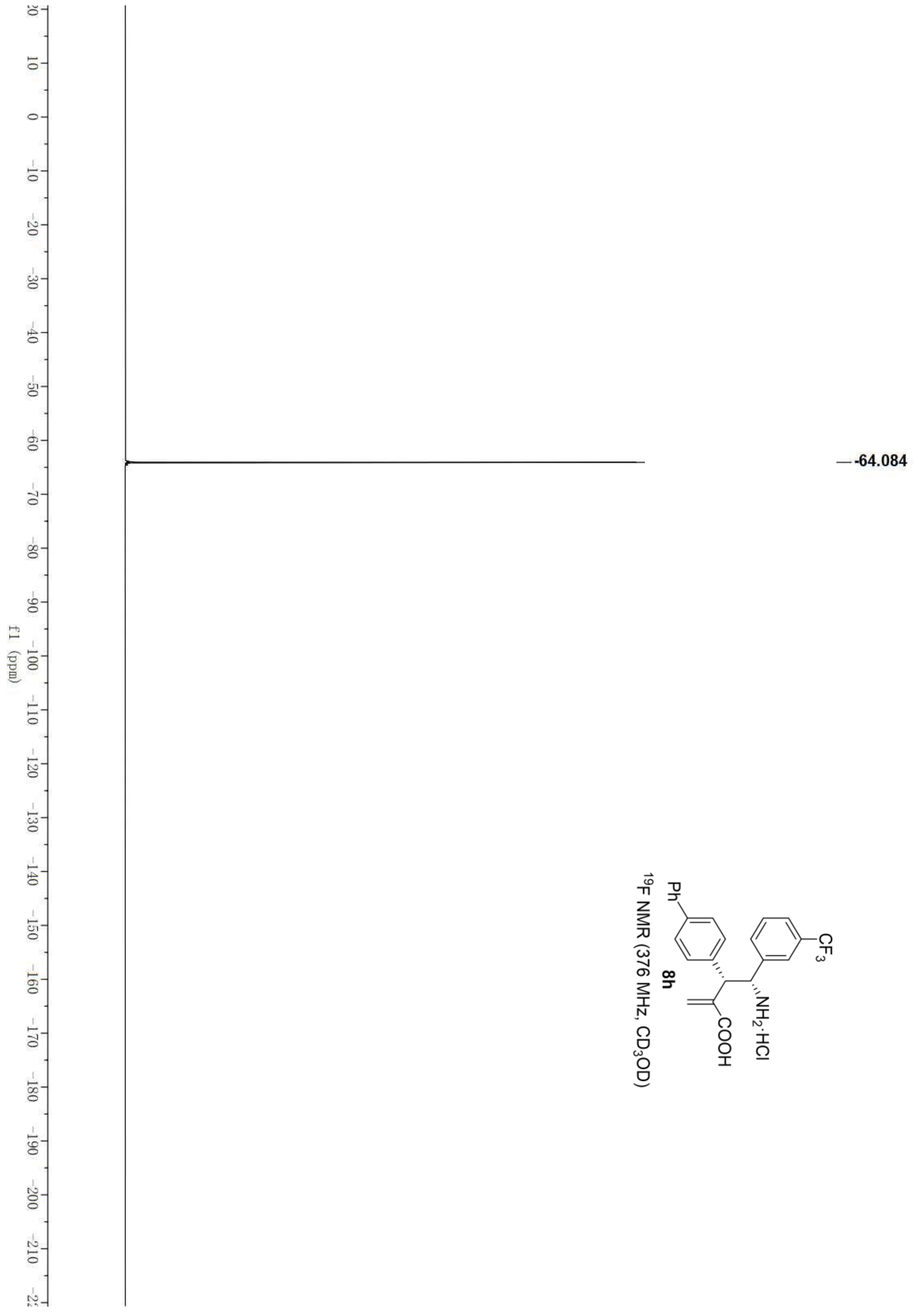

-64.084
f1 (ppm)
20
10
0
-10
-20
-30
-40
-50
-60
-70
-80
-90
-100
-110
-120
-130
-140
-150
-160
-170
-180
-190
-200
-210
CF3
NH2·HCl
COOH
Ph
8h
19F NMR (376 MHz, CD3OD)

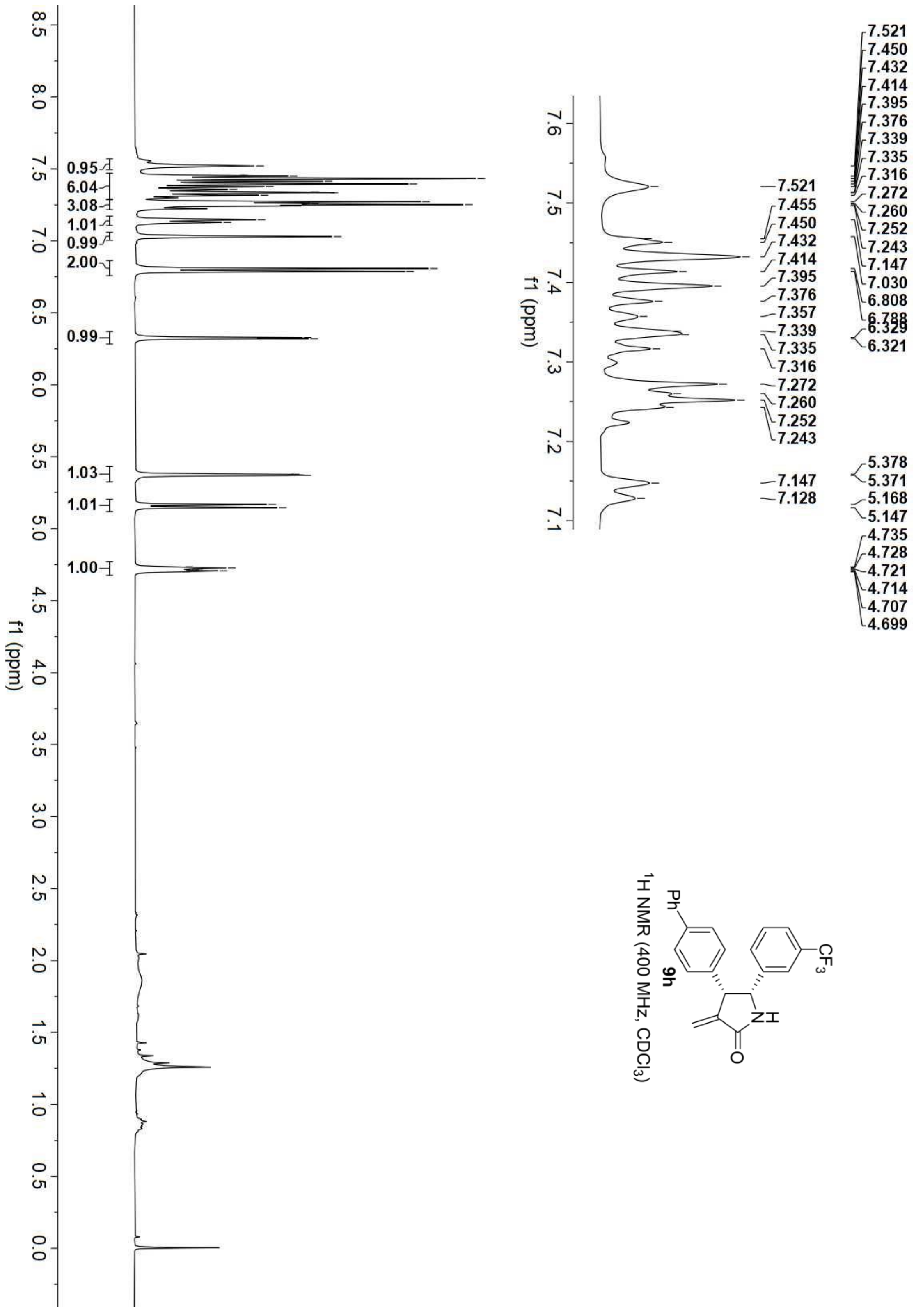

7.521
7.450
7.432
7.414
7.395
7.376
7.339
7.335
7.316
7.272
7.260
7.252
7.243
7.147
7.030
6.808
6.788
6.329
6.321
5.378
5.371
5.168
5.147
4.735
4.728
4.721
4.714
4.707
4.699
7.521
7.455
7.450
7.432
7.414
7.395
7.376
7.357
7.339
7.335
7.316
7.272
7.260
7.252
7.243
7.147
7.128
0.95
6.04
3.08
1.01
0.99
2.00
0.99
1.03
1.01
1.00
f1 (ppm)
f1 (ppm)
CF3
Ph
9h
1H NMR (400 MHz, CDCl3)

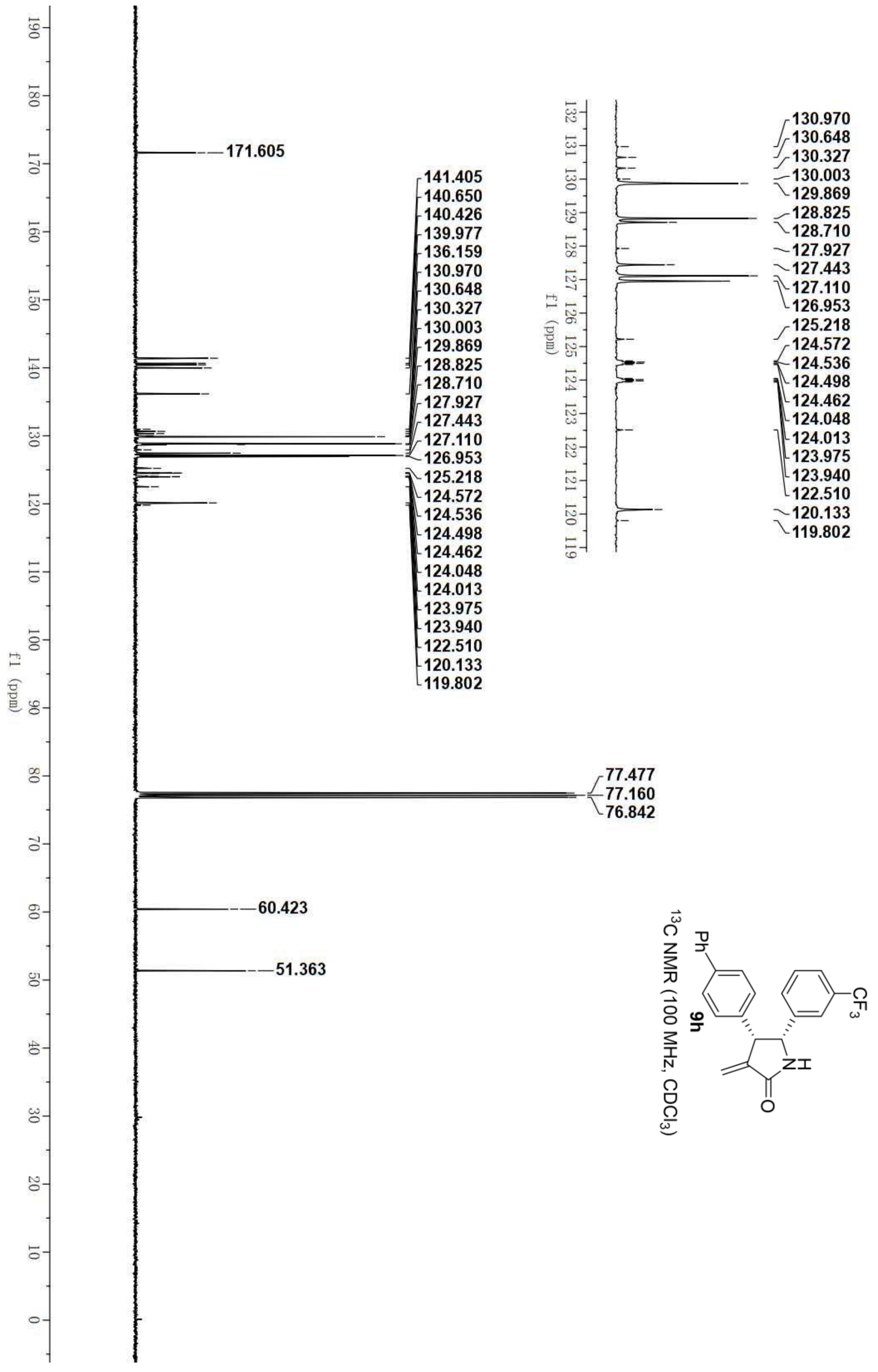
171.605
141.405
140.650
140.426
139.977
136.159
130.970
130.648
130.327
130.003
129.869
128.825
128.710
127.927
127.443
127.110
126.953
125.218
124.572
124.536
124.498
124.462
124.048
124.013
123.975
123.940
122.510
120.133
119.802
77.477
77.160
76.842
60.423
51.363
f1 (ppm)
9h
^{13}C NMR (100 MHz, $CDCl_3$)

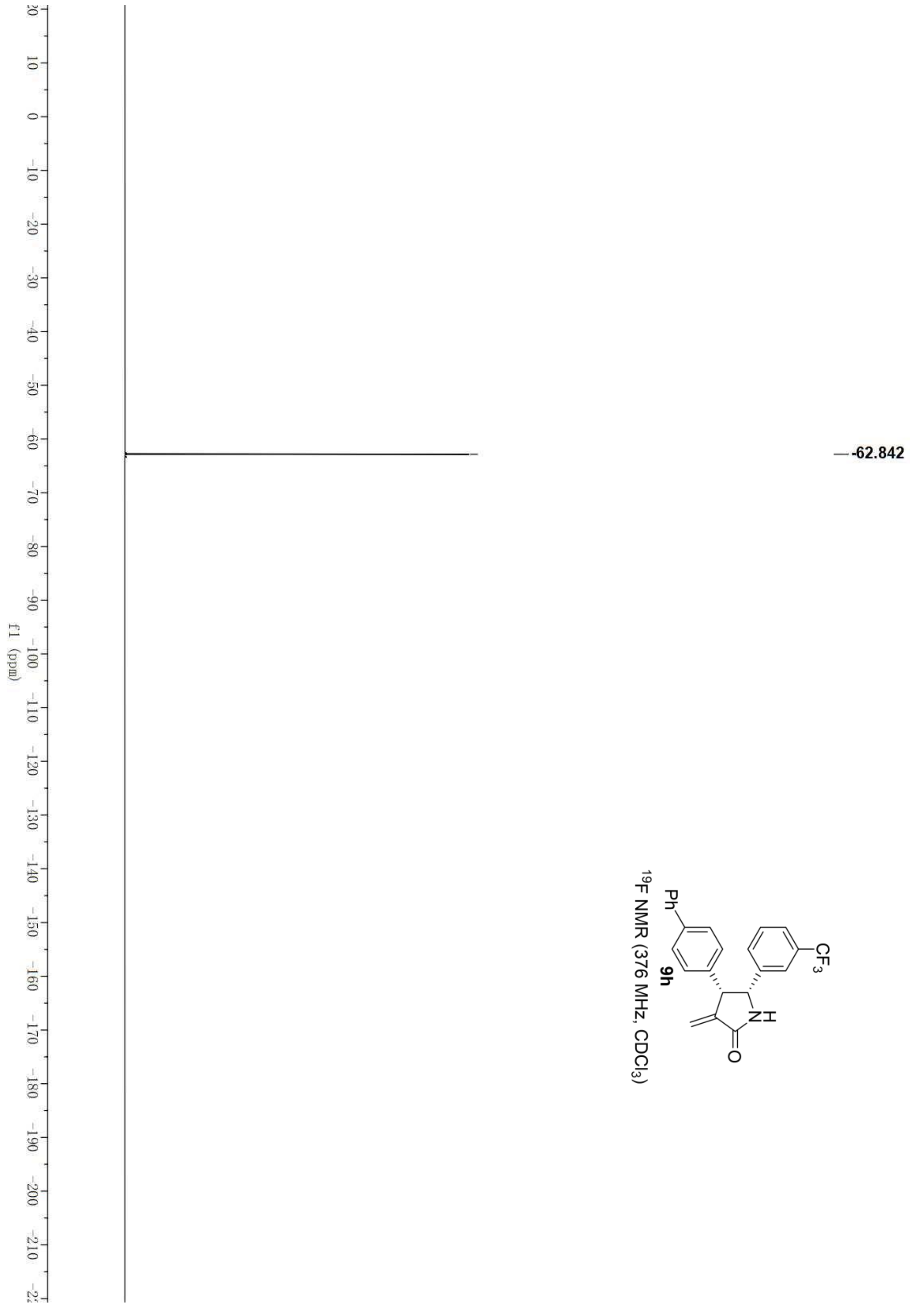

-62.842
CF3
H
N
O
Ph
9h
19F NMR (376 MHz, CDCl3)
20 10 0 -10 -20 -30 -40 -50 -60 -70 -80 -90 -100 -110 -120 -130 -140 -150 -160 -170 -180 -190 -200 -210
f1 (ppm)

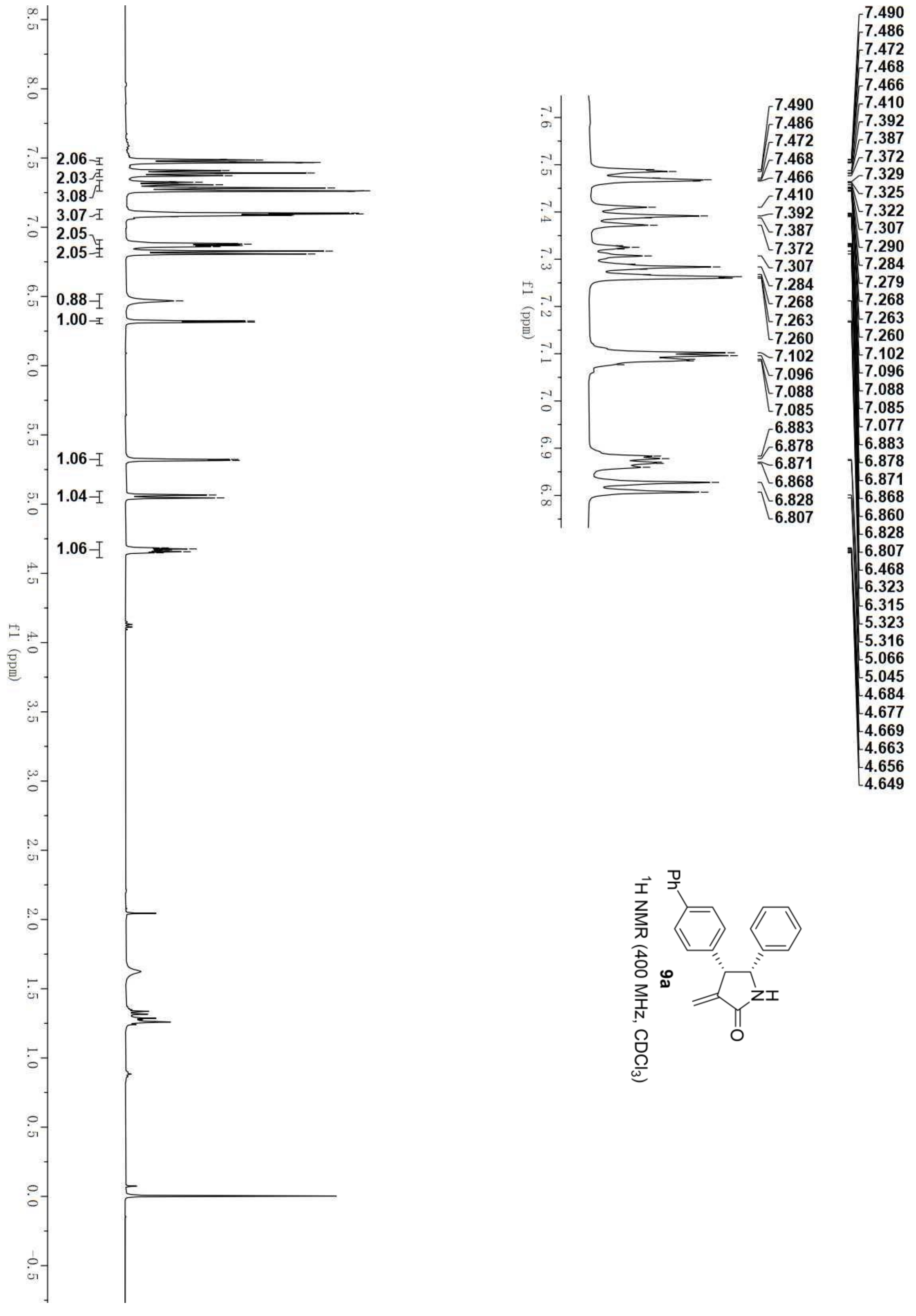

Ph
9a
¹H NMR (400 MHz, CDCl3)
f1 (ppm)
2.06
2.03
3.08
3.07
2.05
2.05
0.88
1.00
1.06
1.04
1.06
7.490
7.486
7.472
7.468
7.466
7.410
7.392
7.387
7.372
7.329
7.325
7.322
7.307
7.290
7.284
7.279
7.268
7.263
7.260
7.102
7.096
7.088
7.085
7.077
6.883
6.878
6.871
6.868
6.860
6.828
6.807
6.468
6.323
6.315
5.323
5.316
5.066
5.045
4.684
4.677
4.669
4.663
4.656
4.649

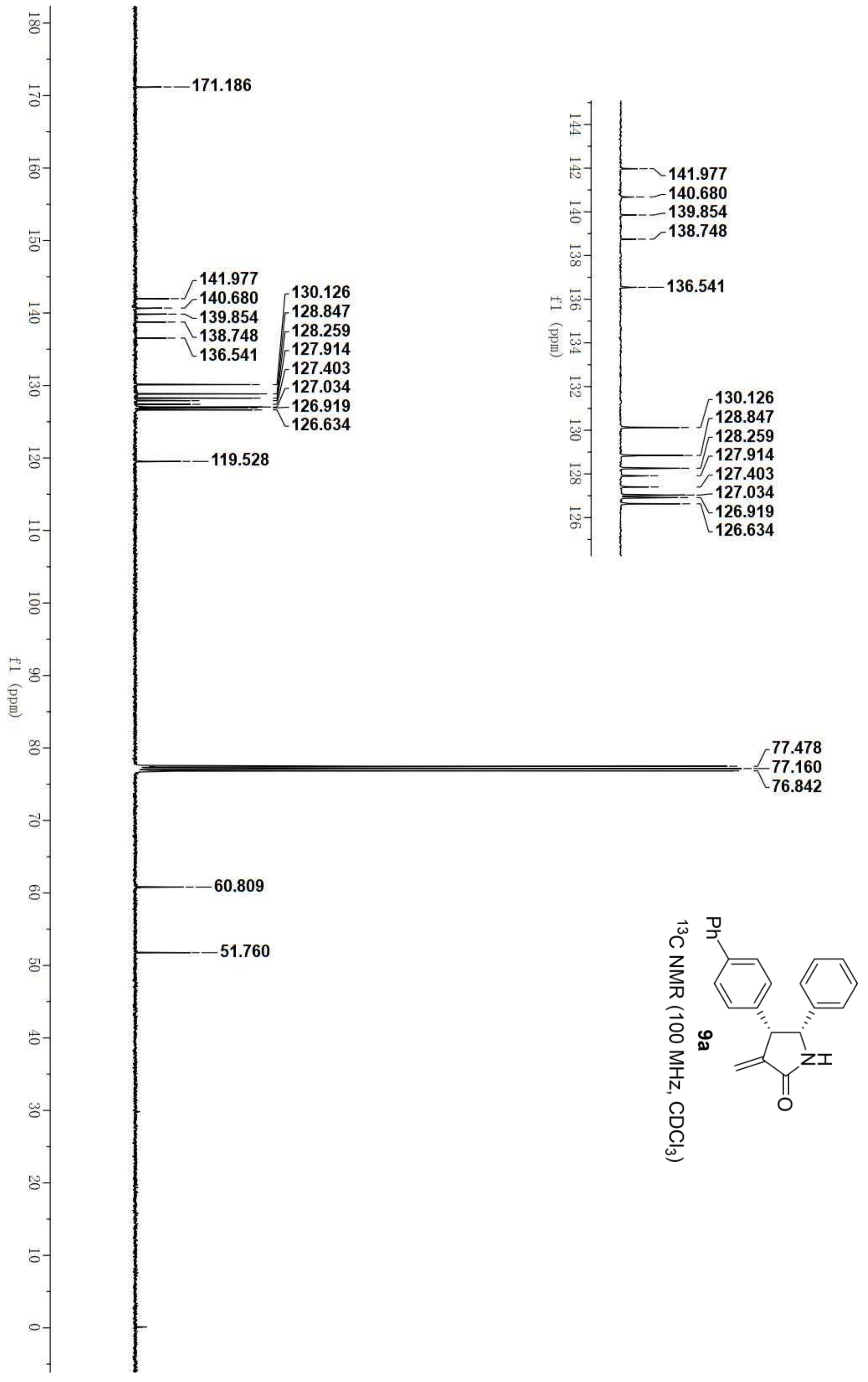

171.186
141.977
140.680
139.854
138.748
136.541
130.126
128.847
128.259
127.914
127.403
127.034
126.919
126.634
119.528
77.478
77.160
76.842
60.809
51.760
f1 (ppm)
Ph
NH
O
9a
13C NMR (100 MHz, CDCl3)

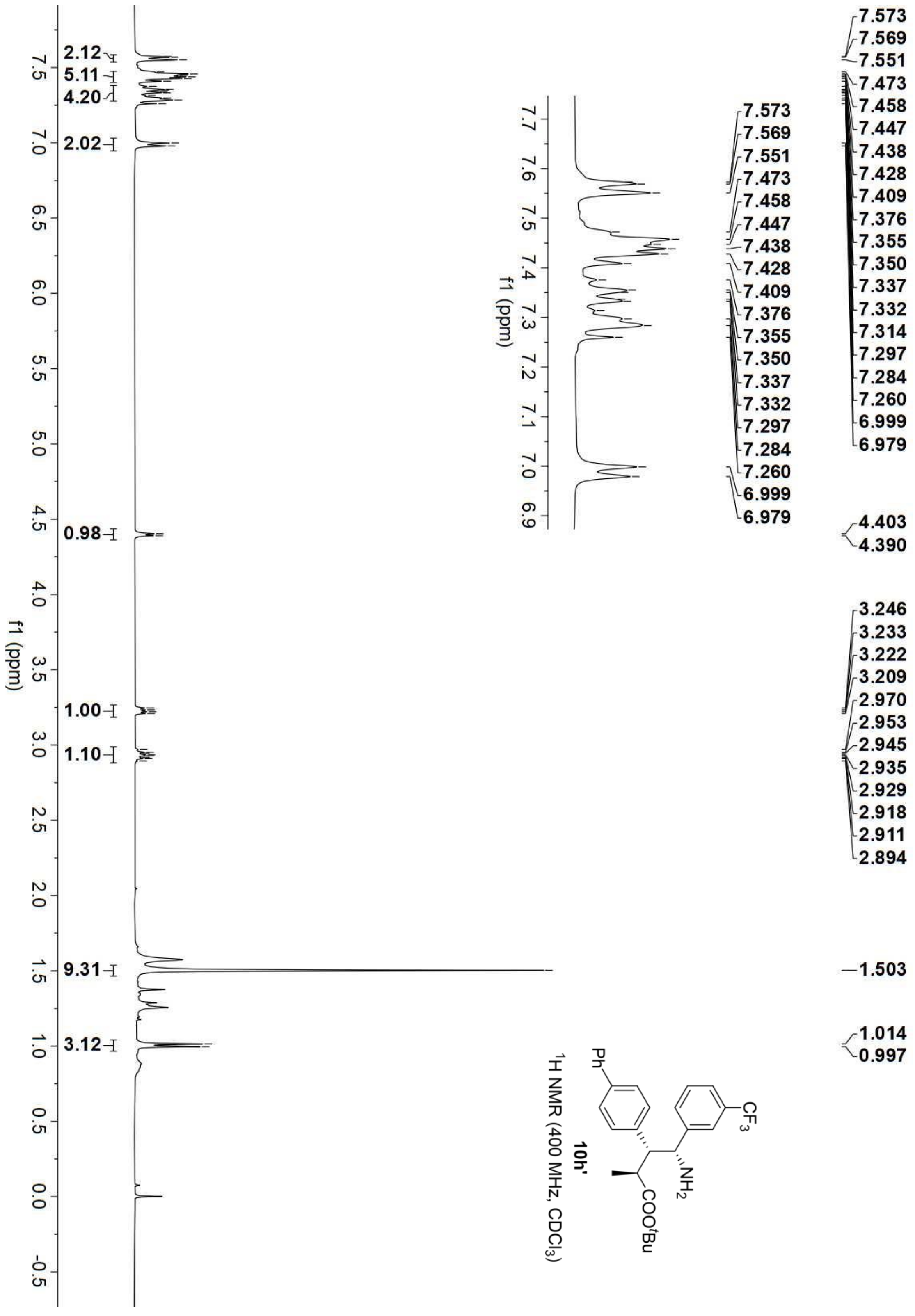

7.573
7.569
7.551
7.473
7.458
7.447
7.438
7.428
7.409
7.376
7.355
7.350
7.337
7.332
7.314
7.297
7.284
7.260
6.999
6.979
4.403
4.390
3.246
3.233
3.222
3.209
2.970
2.953
2.945
2.935
2.929
2.918
2.911
2.894
1.503
1.014
0.997
2.12
5.11
4.20
2.02
0.98
1.00
1.10
9.31
3.12
f1 (ppm)
CF3
NH2
COO^{t}Bu
Ph
10h'
^{1}H NMR (400 MHz, CDCl$_3$)

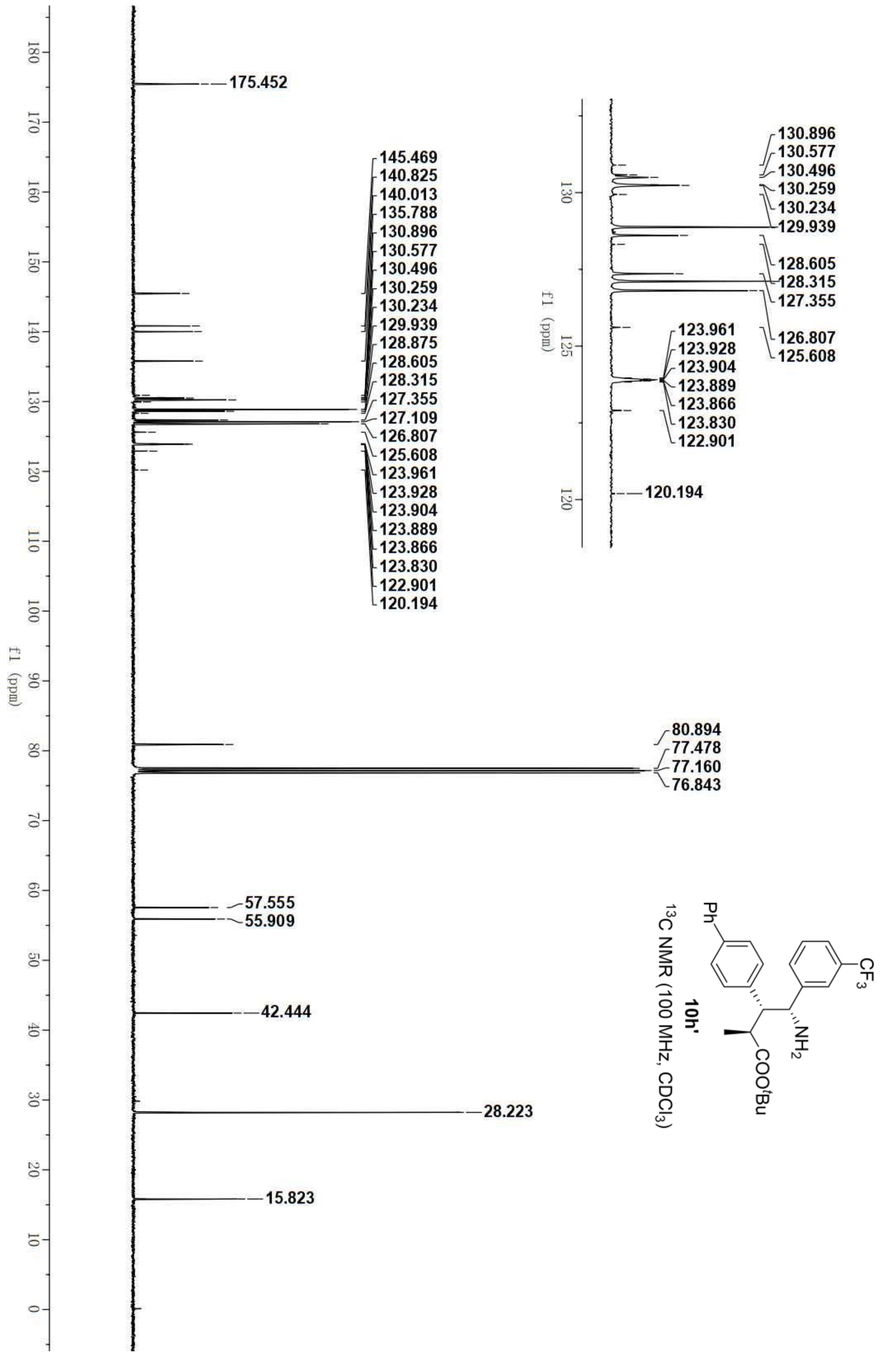
175.452
145.469
140.825
140.013
135.788
130.896
130.577
130.496
130.259
130.234
129.939
128.875
128.605
128.315
127.355
127.109
126.807
125.608
123.961
123.928
123.904
123.889
123.866
123.830
122.901
120.194
80.894
77.478
77.160
76.843
57.555
55.909
42.444
28.223
15.823
f1 (ppm)
Ph
CF3
NH2
COOtBu
10h'
13C NMR (100 MHz, CDCl3)

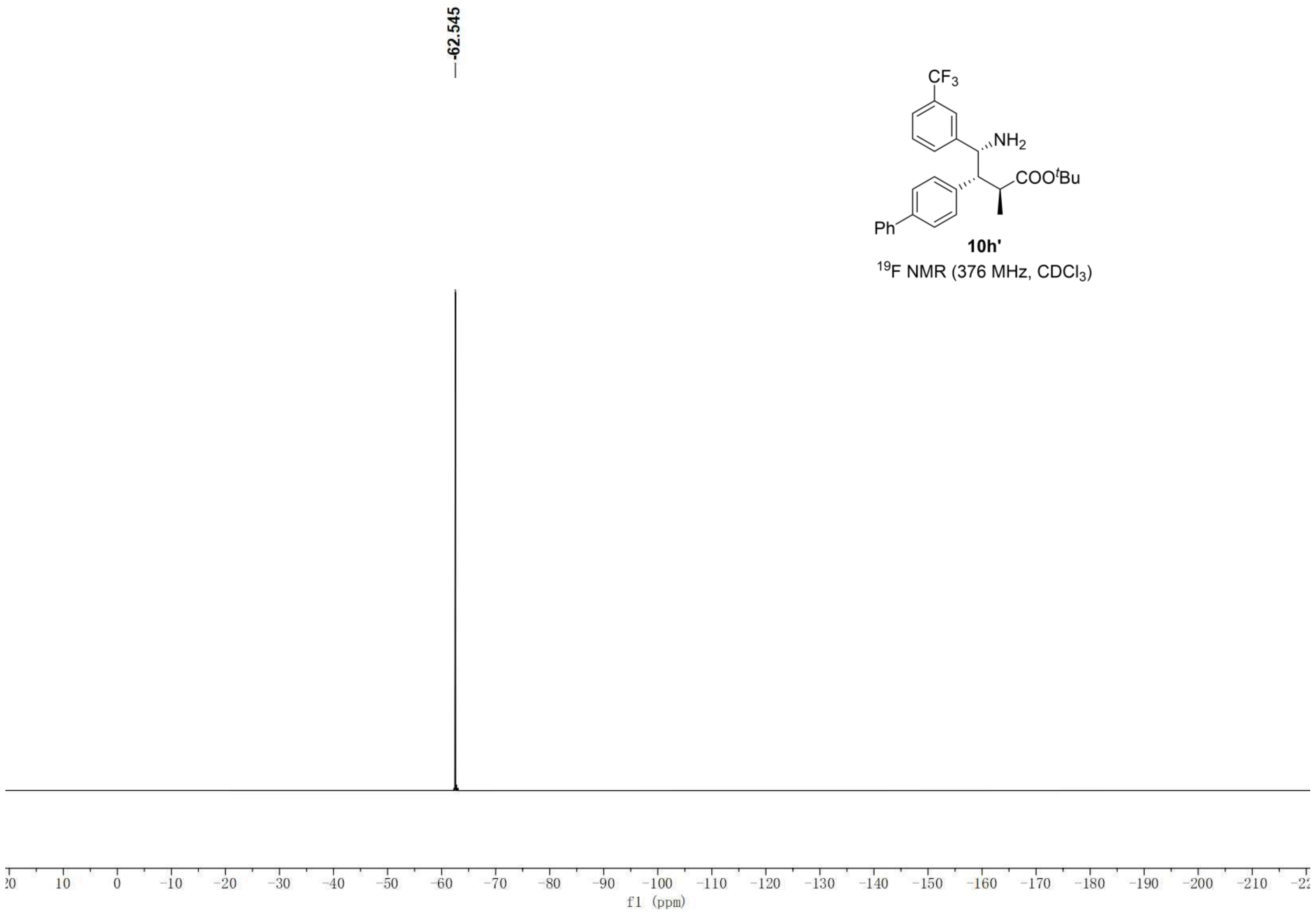

10h'

^{19}F NMR (376 MHz, $CDCl_3$)

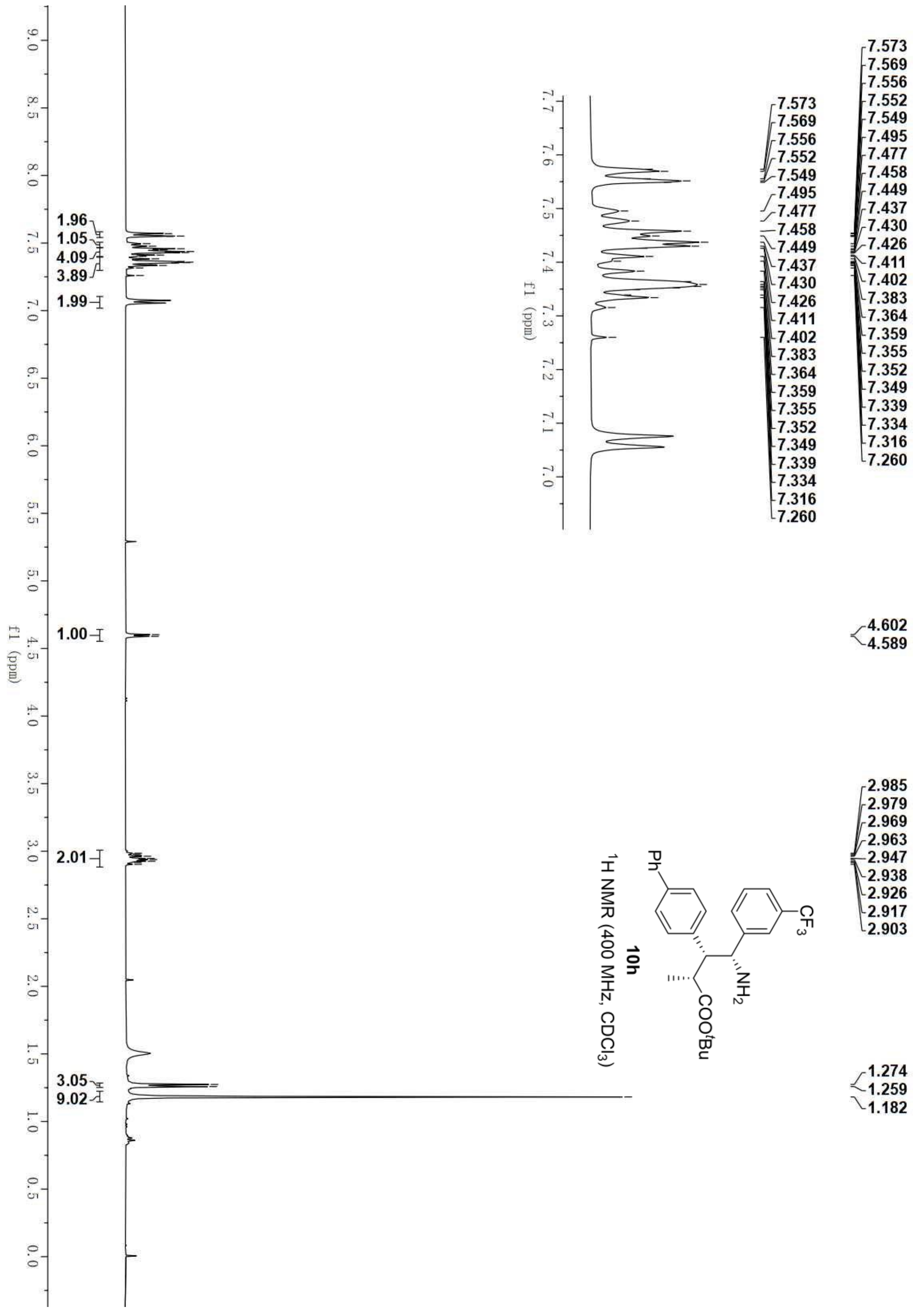
10h
^{1}H NMR (400 MHz, CDCl$_3$)
Ph
CF$_3$
NH$_2$
COO^{t}Bu
7.573
7.569
7.556
7.552
7.549
7.495
7.477
7.458
7.449
7.437
7.430
7.426
7.411
7.402
7.383
7.364
7.359
7.355
7.352
7.349
7.339
7.334
7.316
7.260
4.602
4.589
2.985
2.979
2.969
2.963
2.947
2.938
2.926
2.917
2.903
1.274
1.259
1.182
1.96
1.05
4.09
3.89
1.99
1.00
2.01
3.05
9.02
f1 (ppm)

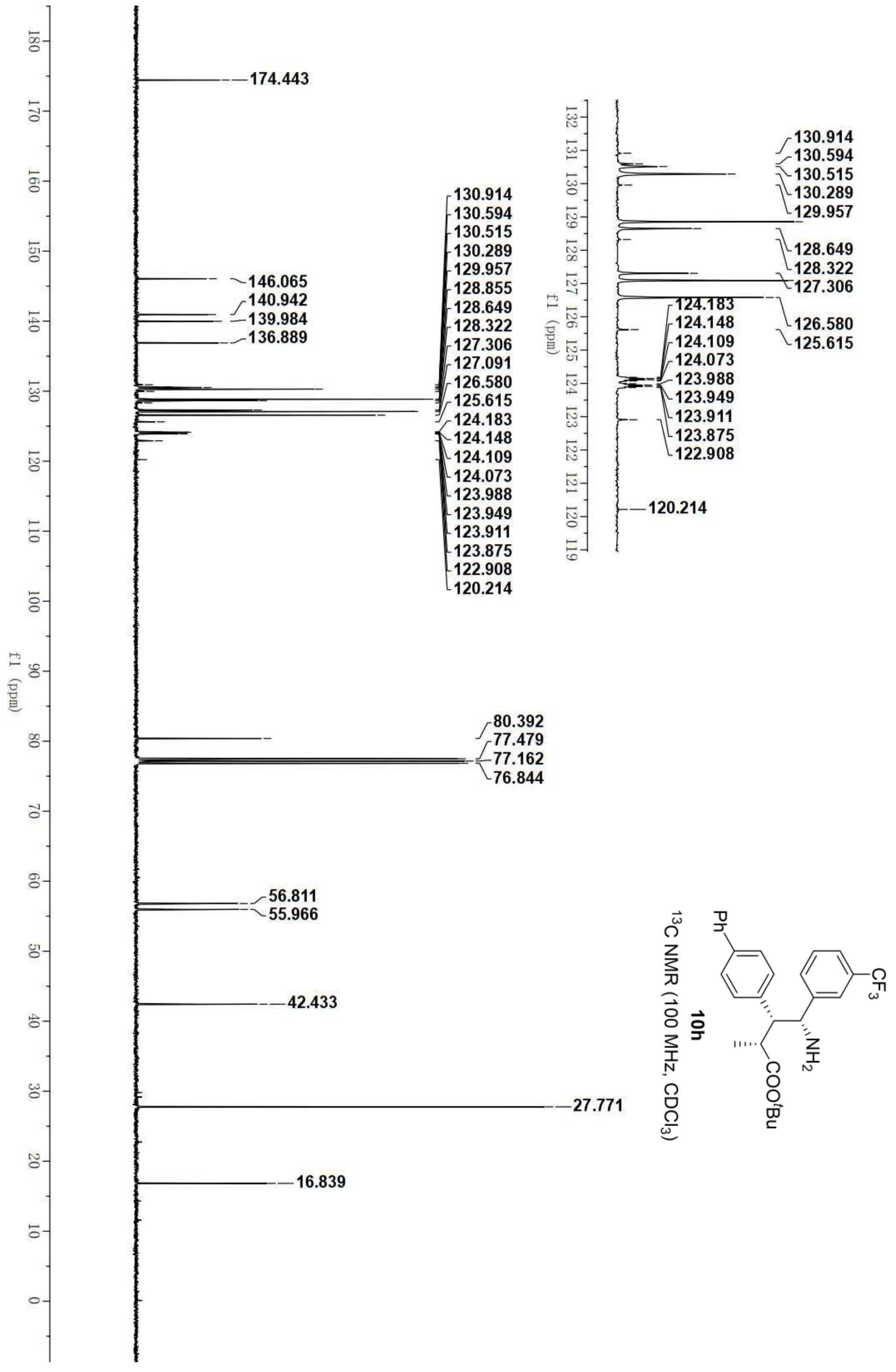

174.443
146.065
140.942
139.984
136.889
130.914
130.594
130.515
130.289
129.957
128.855
128.649
128.322
127.306
127.091
126.580
125.615
124.183
124.148
124.109
124.073
123.988
123.949
123.911
123.875
122.908
120.214
80.392
77.479
77.162
76.844
56.811
55.966
42.433
27.771
16.839
f1 (ppm)
10h
13C NMR (100 MHz, CDCl3)

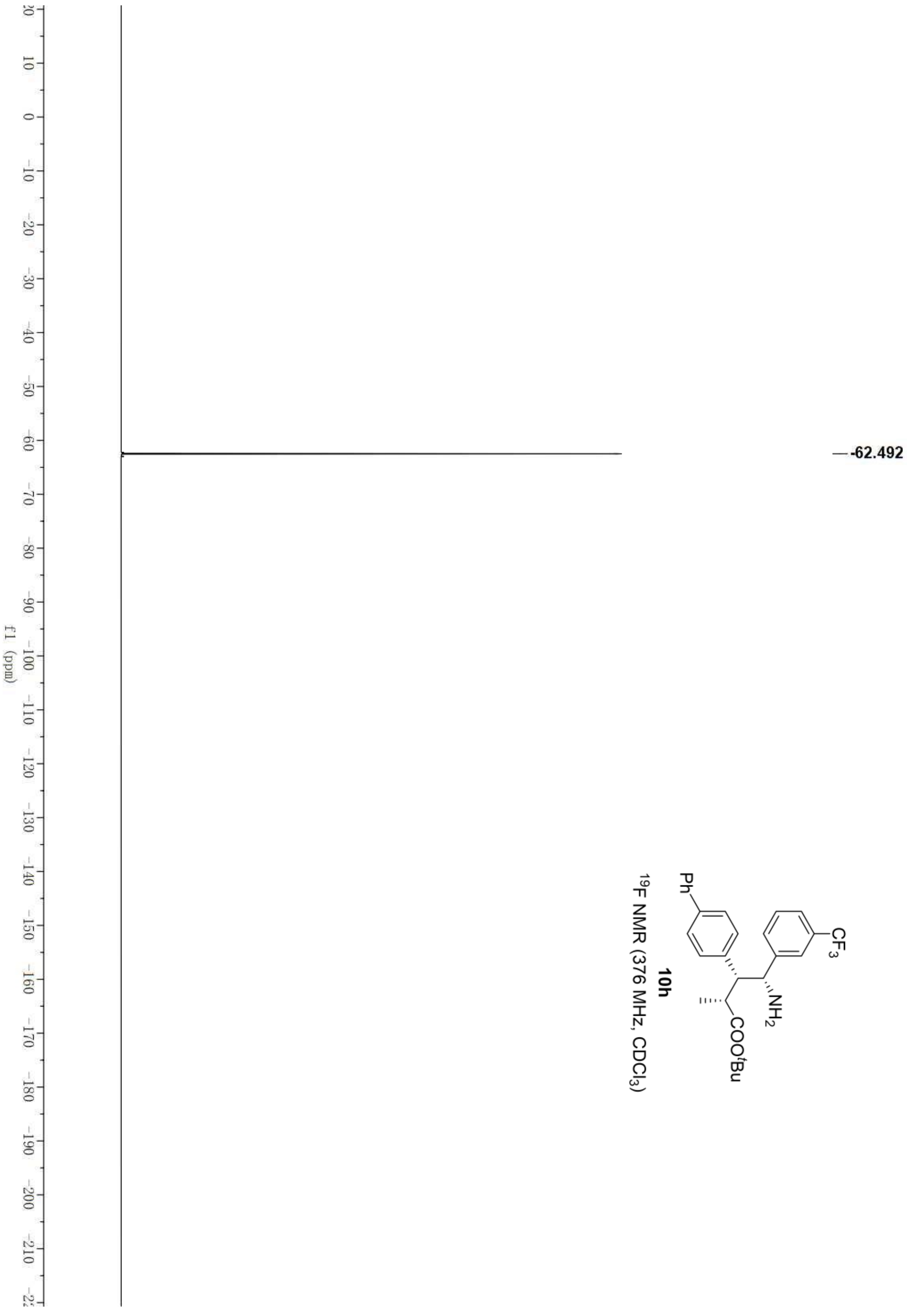
-62.492
CF3
NH2
COOtBu
Ph
10h
19F NMR (376 MHz, CDCl3)
f1 (ppm)

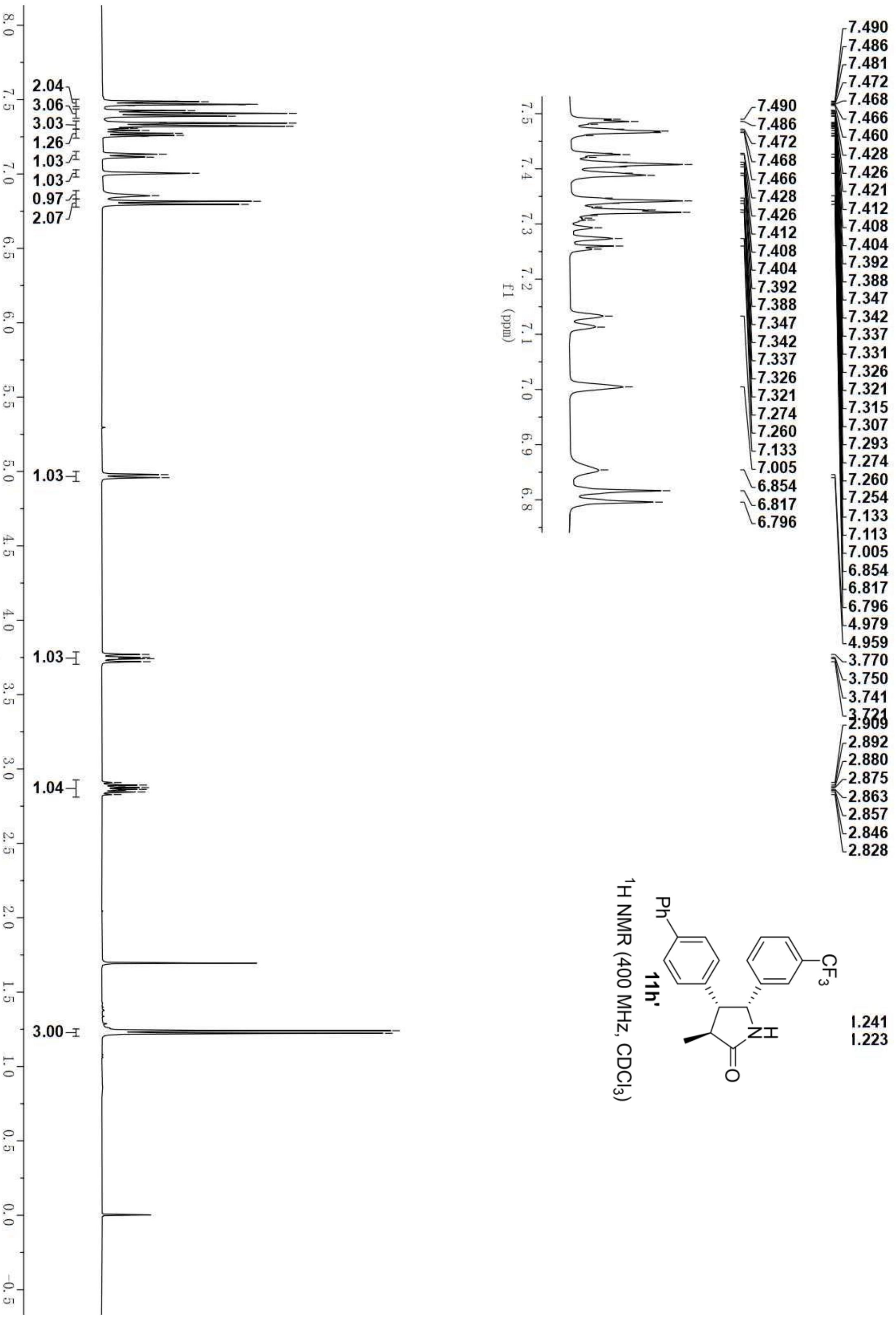

7.490
7.486
7.481
7.472
7.468
7.466
7.460
7.428
7.426
7.421
7.412
7.408
7.404
7.392
7.388
7.347
7.342
7.337
7.331
7.326
7.321
7.315
7.307
7.293
7.274
7.260
7.254
7.133
7.113
7.005
6.854
6.817
6.796
4.979
4.959
3.770
3.750
3.741
3.721
2.909
2.892
2.880
2.875
2.863
2.857
2.846
2.828
1.241
1.223
CF$_3$
Ph
11h'
^{1}H NMR (400 MHz, CDCl$_3$)
f1 (ppm)

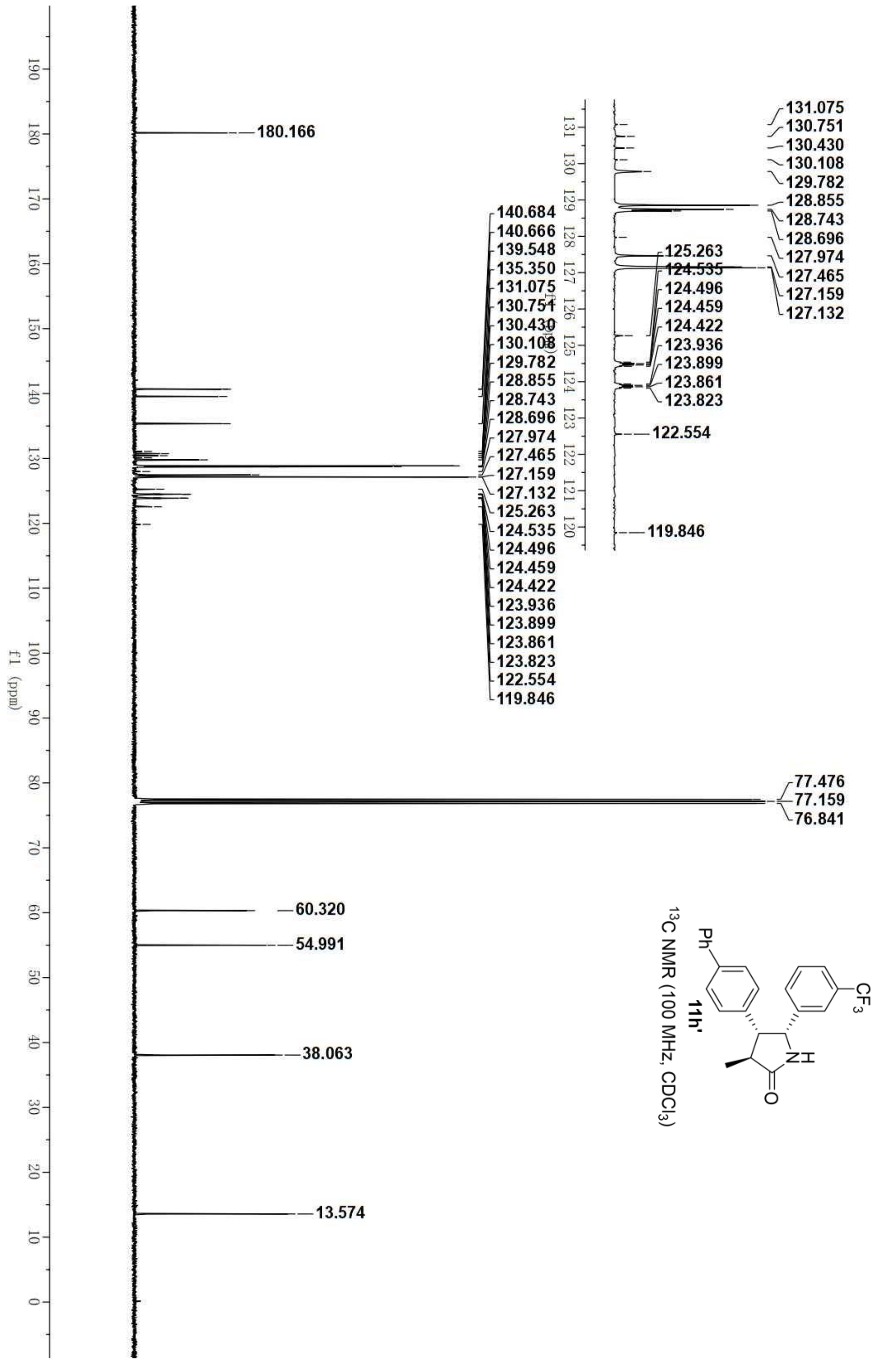
180.166
140.684
140.666
139.548
135.350
131.075
130.751
130.430
130.108
129.782
128.855
128.743
128.696
127.974
127.465
127.159
127.132
125.263
124.535
124.496
124.459
124.422
123.936
123.899
123.861
123.823
122.554
119.846
77.476
77.159
76.841
60.320
54.991
38.063
13.574
f1 (ppm)
Ph
CF3
NH
O
11h'
13C NMR (100 MHz, CDCl3)

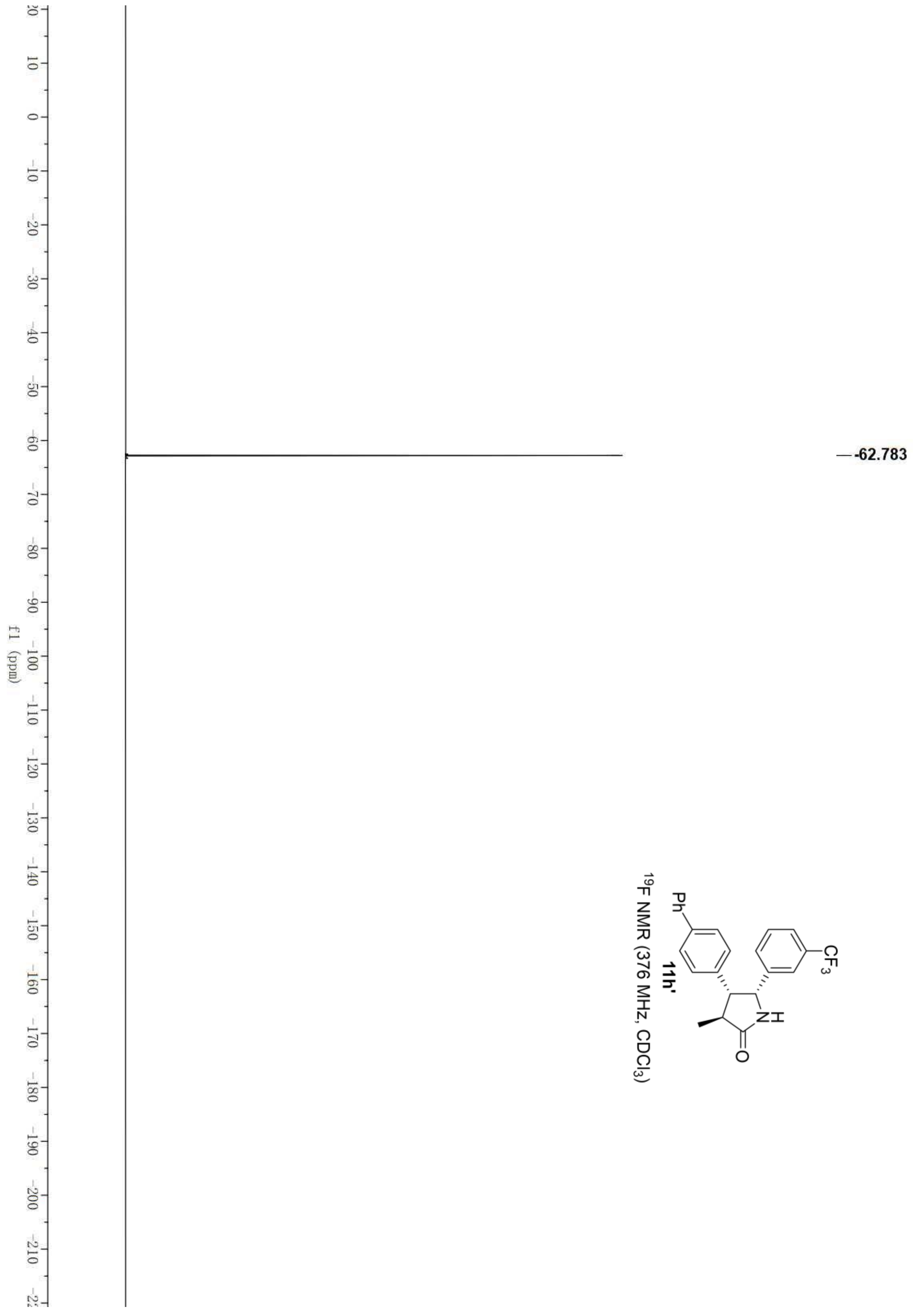

-62.783
Ph
CF3
H
N
O
11h'
19F NMR (376 MHz, CDCl3)
f1 (ppm)

13. Chromatograms for Determination of Enantiomeric Excesses

Compound 3a

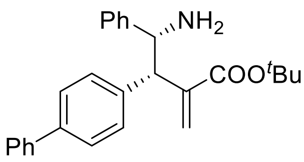

HPLC Conditions
Column: Chiralcel AD-H, Daicel Chemical Industries, Ltd.
Eluent: Hexanes/Isopropanol (94:6)
Flow rate: 0.5 mL/min
Detection: UV 254 nm

Racemic

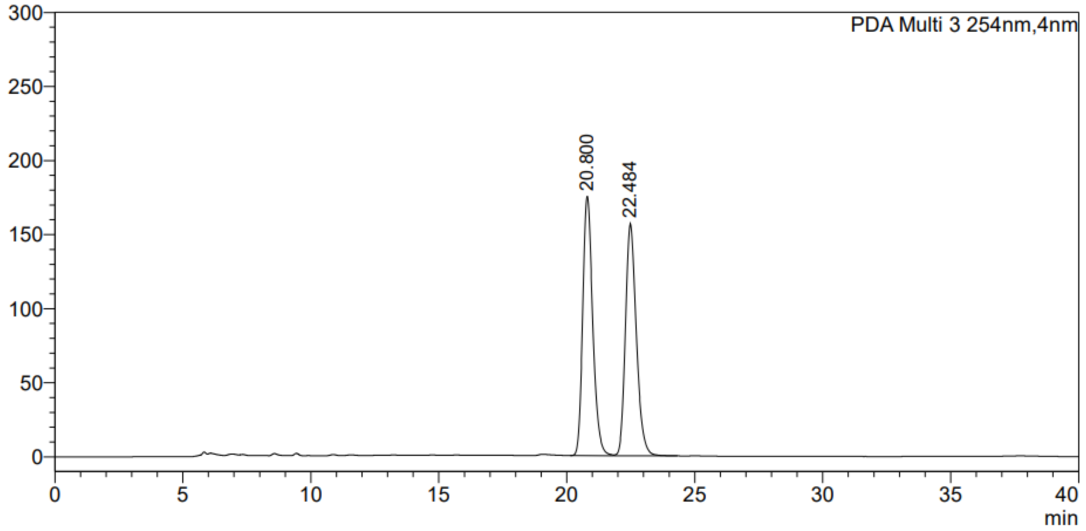

PDA Ch3 254nm

Peak#	Ret. Time	Height	Area	Area%
1	20.800	175109	4756607	50.563
2	22.484	156612	4650655	49.437
Total		331721	9407262	100.000

Chiral

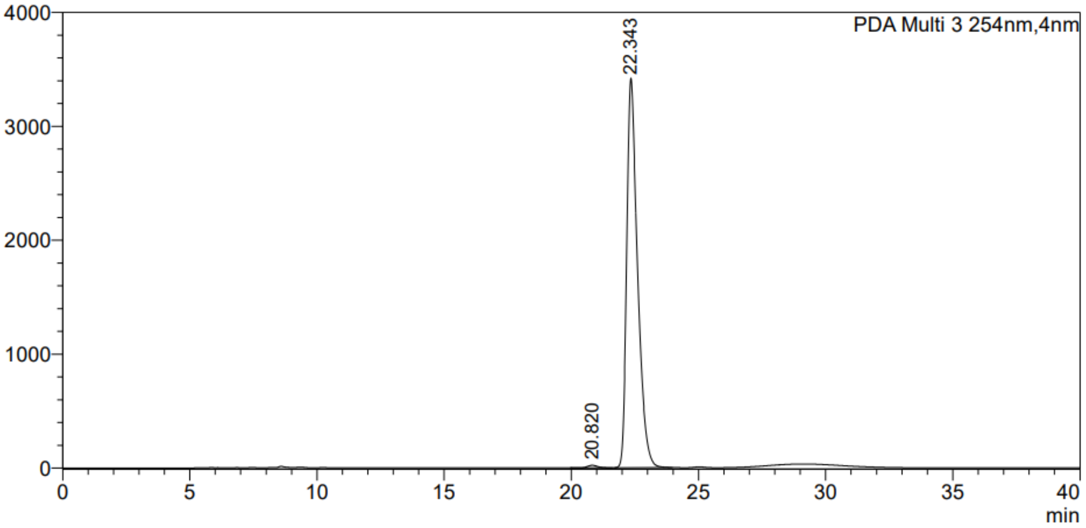

PDA Ch3 254nm

Peak#	Ret. Time	Height	Area	Area%
1	20.820	21839	613727	0.605
2	22.343	3417022	100804090	99.395
Total		3438861	101417817	100.000

Compound 3b

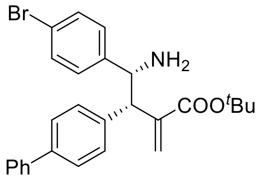

HPLC Conditions
Column: Chiralcel AD-H, Daicel Chemical Industries, Ltd.
Eluent: Hexanes/Isopropanol (94:6)
Flow rate: 0.5 mL/min
Detection: UV 254 nm

Racemic

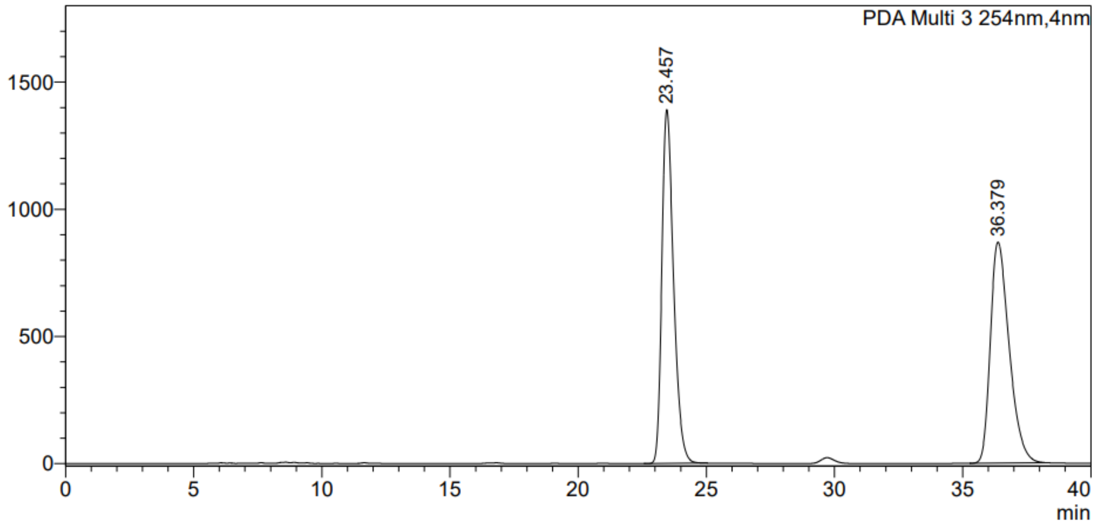

PDA Ch3 254nm

Peak#	Ret. Time	Height	Area	Area%
1	23.457	1390864	44049508	49.860
2	36.379	869009	44297428	50.140
Total		2259872	88346937	100.000

Chiral

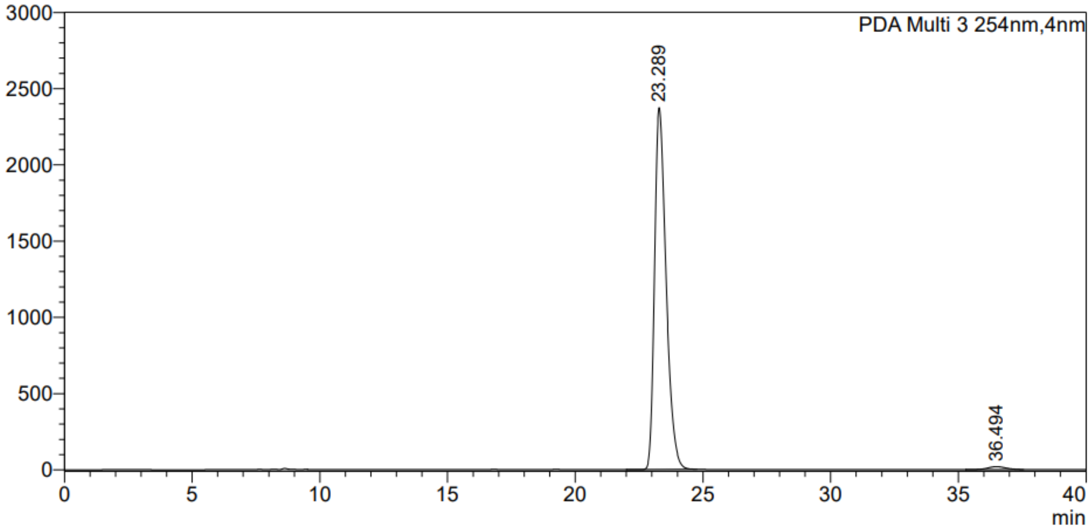

PDA Ch3 254nm

Peak#	Ret. Time	Height	Area	Area%
1	23.289	2372324	74590578	98.791
2	36.494	18829	912552	1.209
Total		2391153	75503130	100.000

Compound 3c

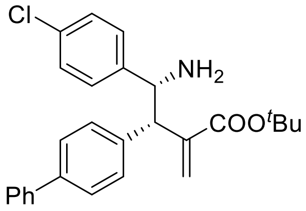

HPLC Conditions
Column: Chiralcel AD-H, Daicel Chemical Industries, Ltd.
Eluent: Hexanes/Isopropanol (94:6)
Flow rate: 0.5 mL/min
Detection: UV 254 nm

Racemic

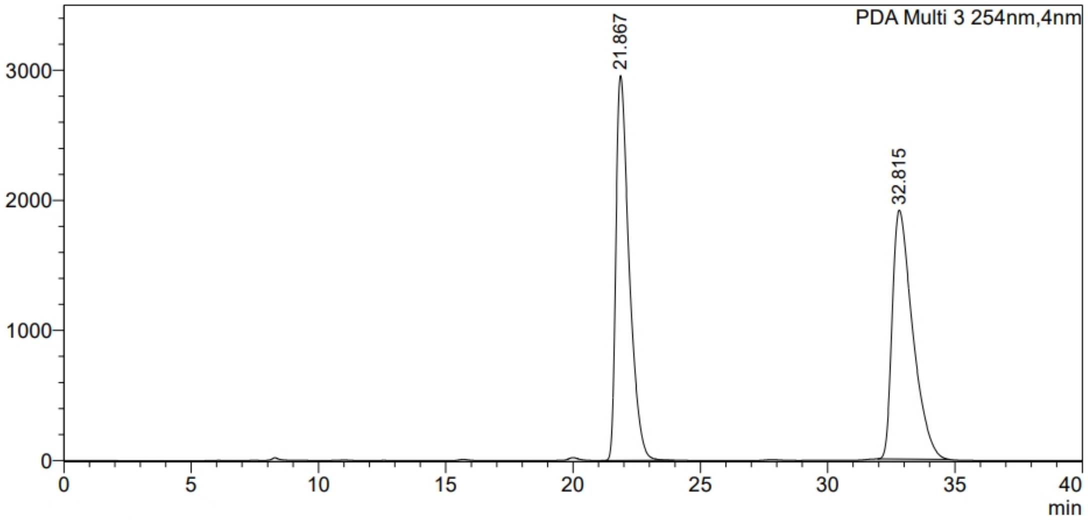

PDA Ch3 254nm

Peak#	Ret. Time	Height	Area	Area%
1	21.867	2959999	107149898	50.085
2	32.815	1914229	106787344	49.915
Total		4874227	213937242	100.000

Chiral

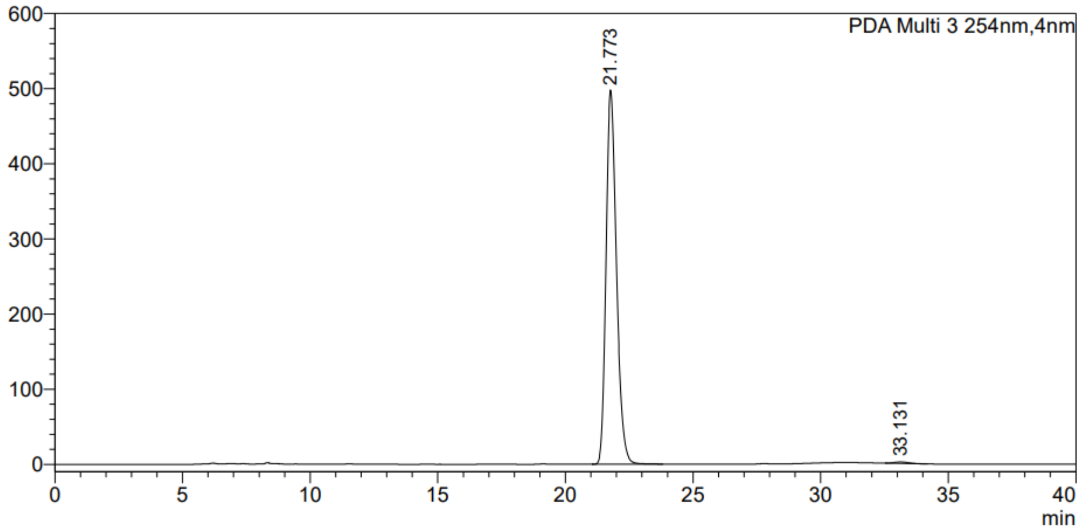

PDA Ch3 254nm

Peak#	Ret. Time	Height	Area	Area%
1	21.773	497646	14165497	99.464
2	33.131	1910	76364	0.536
Total		499557	14241861	100.000

Compound 3d

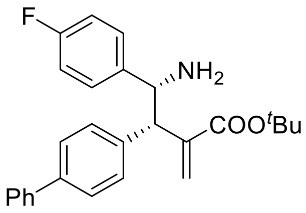

HPLC Conditions

Column: Chiralcel AD-H, Daicel Chemical Industries, Ltd.
Eluent: Hexanes/Isopropanol (94:6)
Flow rate: 0.5 mL/min
Detection: UV 254 nm

Racemic

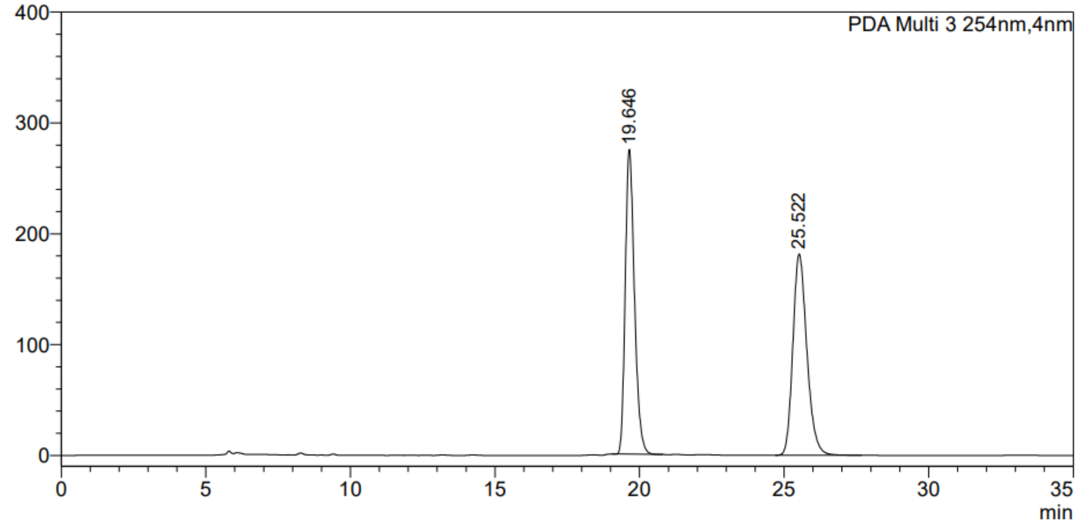

PDA Ch3 254nm

Peak#	Ret. Time	Height	Area	Area%
1	19.646	274725	6082170	49.860
2	25.522	181592	6116273	50.140
Total		456317	12198443	100.000

Chiral

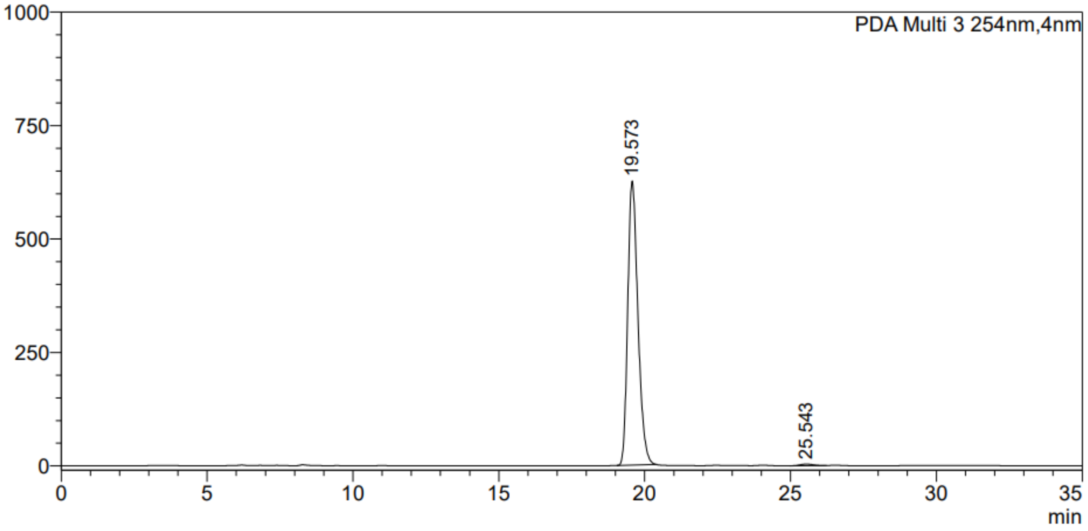

PDA Ch3 254nm

Peak#	Ret. Time	Height	Area	Area%
1	19.573	626094	15175579	99.325
2	25.543	3415	103086	0.675
Total		629510	15278665	100.000

Compound 3e

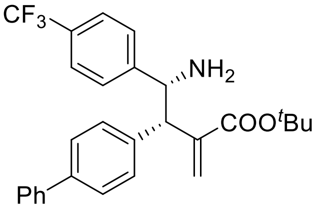

HPLC Conditions
Column: Chiralcel AD-H, Daicel Chemical Industries, Ltd.
Eluent: Hexanes/Isopropanol (94:6)
Flow rate: 0.5 mL/min
Detection: UV 254 nm

Racemic

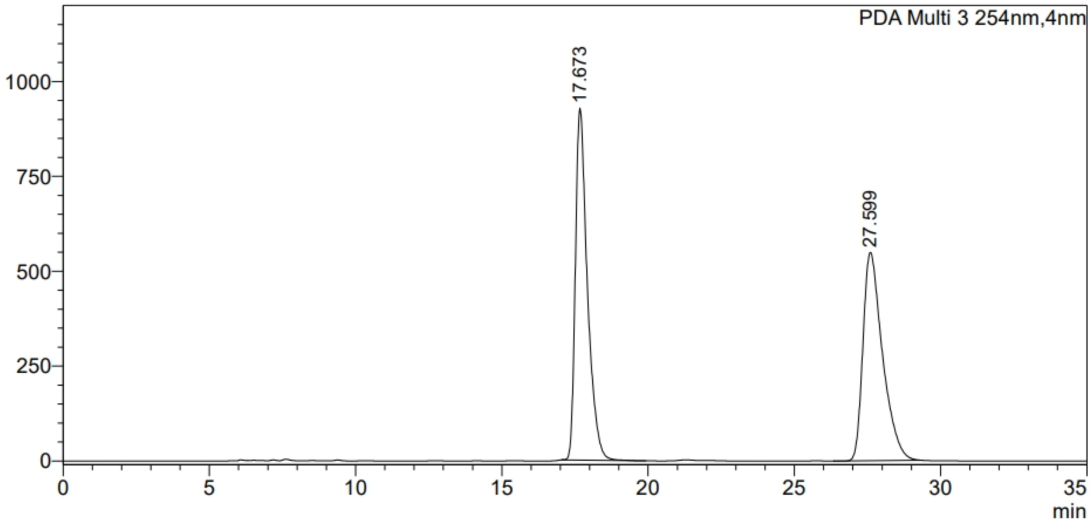

PDA Ch3 254nm

Peak#	Ret. Time	Height	Area	Area%
1	17.673	925797	26440833	50.878
2	27.599	548854	25528415	49.122
Total		1474651	51969248	100.000

Chiral

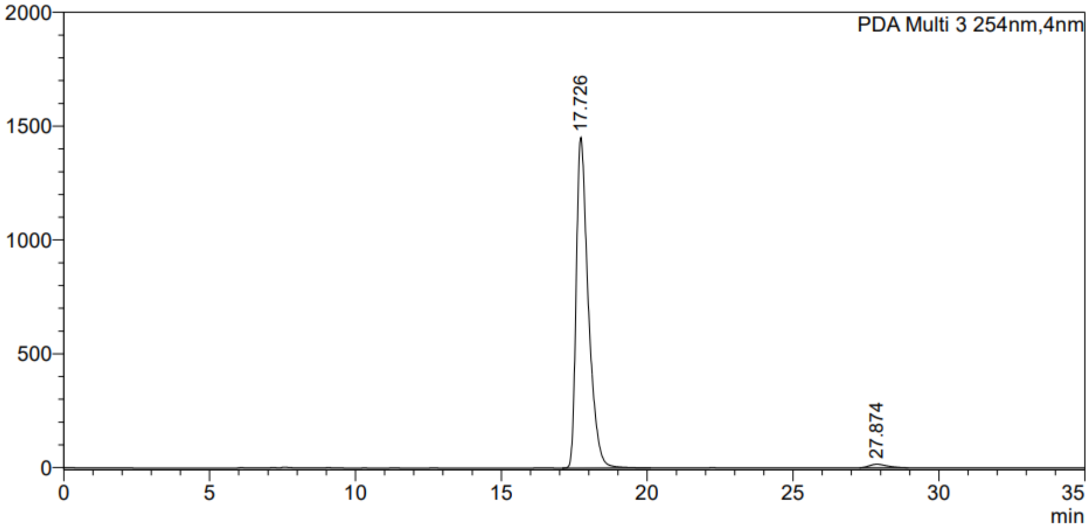

PDA Ch3 254nm

Peak#	Ret. Time	Height	Area	Area%
1	17.726	1451676	41739858	98.454
2	27.874	15476	655298	1.546
Total		1467152	42395156	100.000

Compound 3f

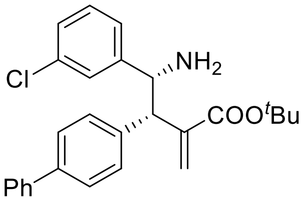

HPLC Conditions
Column: Chiralcel AD-H, Daicel Chemical Industries, Ltd.
Eluent: Hexanes/Isopropanol (94:6)
Flow rate: 0.5 mL/min
Detection: UV 254 nm

Racemic

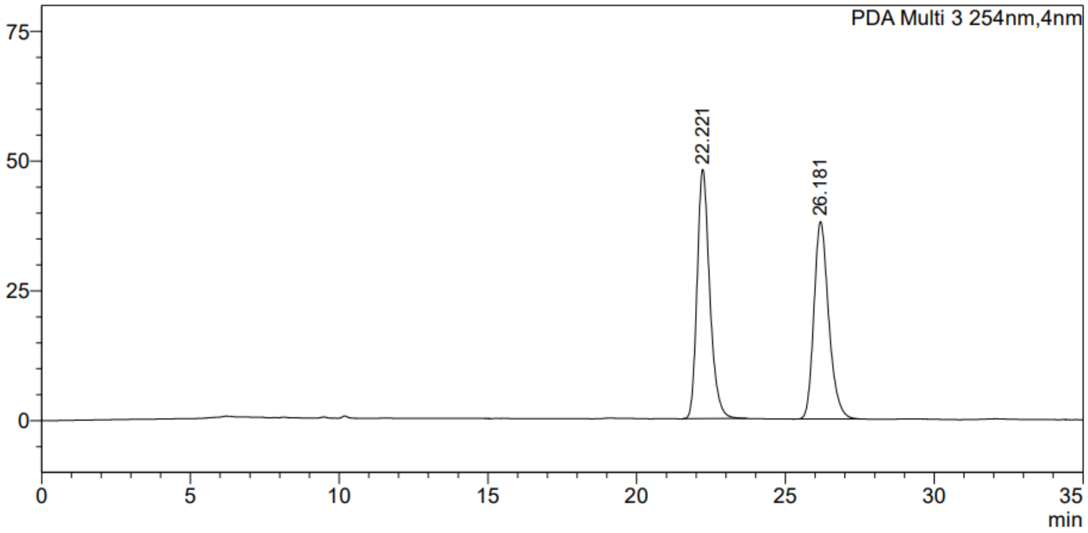

PDA Ch3 254nm

Peak#	Ret. Time	Height	Area	Area%
1	22.221	48039	1415184	51.844
2	26.181	38015	1314492	48.156
Total		86054	2729675	100.000

Chiral

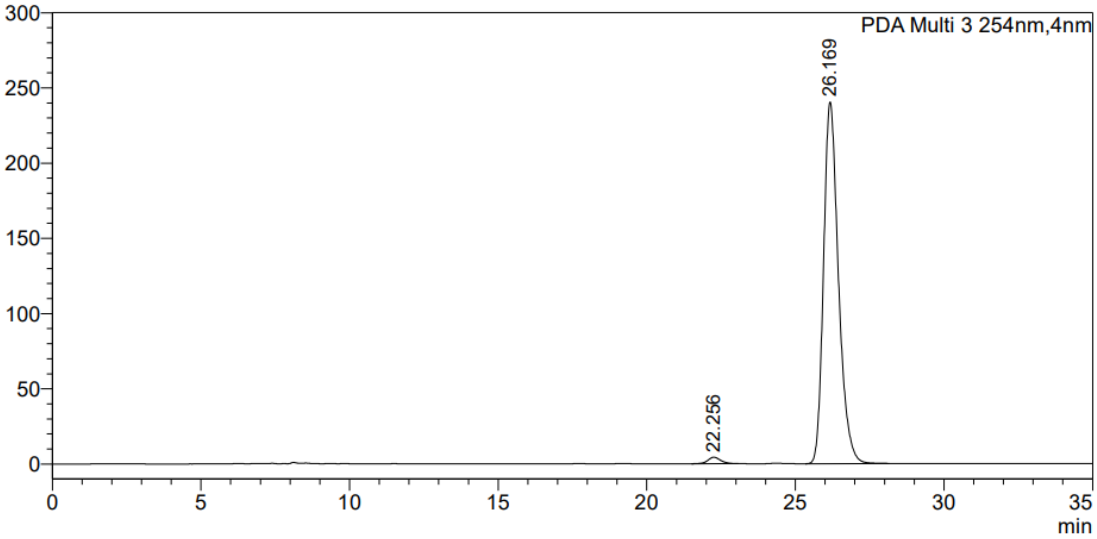

PDA Ch3 254nm

Peak#	Ret. Time	Height	Area	Area%
1	22.256	4296	127472	1.500
2	26.169	240288	8369115	98.500
Total		244584	8496586	100.000

Compound 3g

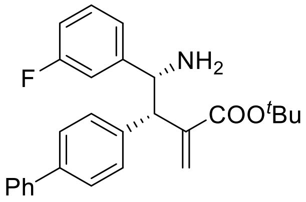

HPLC Conditions
Column: Chiralcel AD-H, Daicel Chemical Industries, Ltd.
Eluent: Hexanes/Isopropanol (94:6)
Flow rate: 0.5 mL/min
Detection: UV 254 nm

Racemic

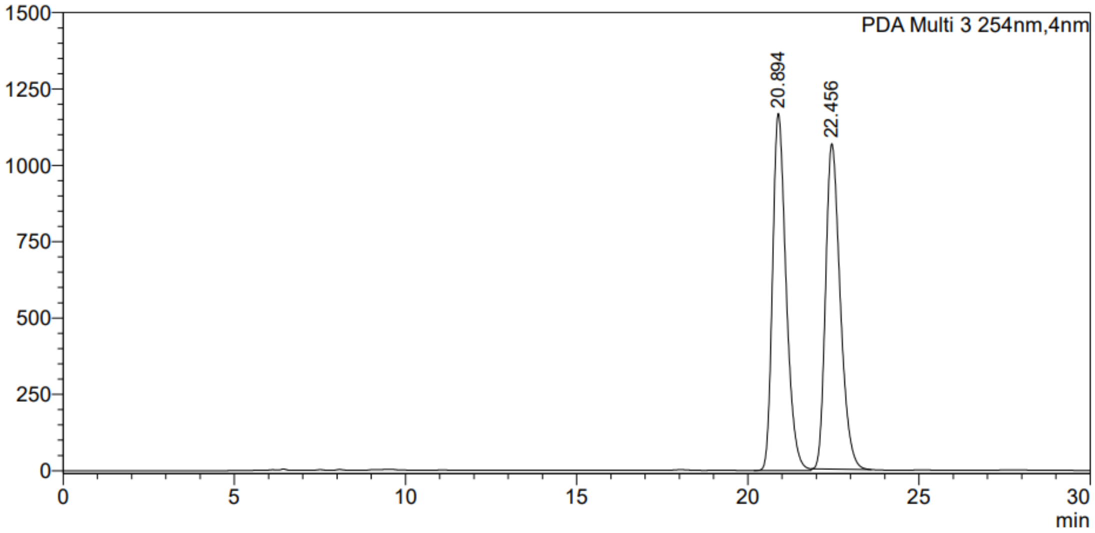

PDA Ch3 254nm

Peak#	Ret. Time	Height	Area	Area%
1	20.894	1168611	32198825	50.355
2	22.456	1065676	31744570	49.645
Total		2234287	63943395	100.000

Chiral

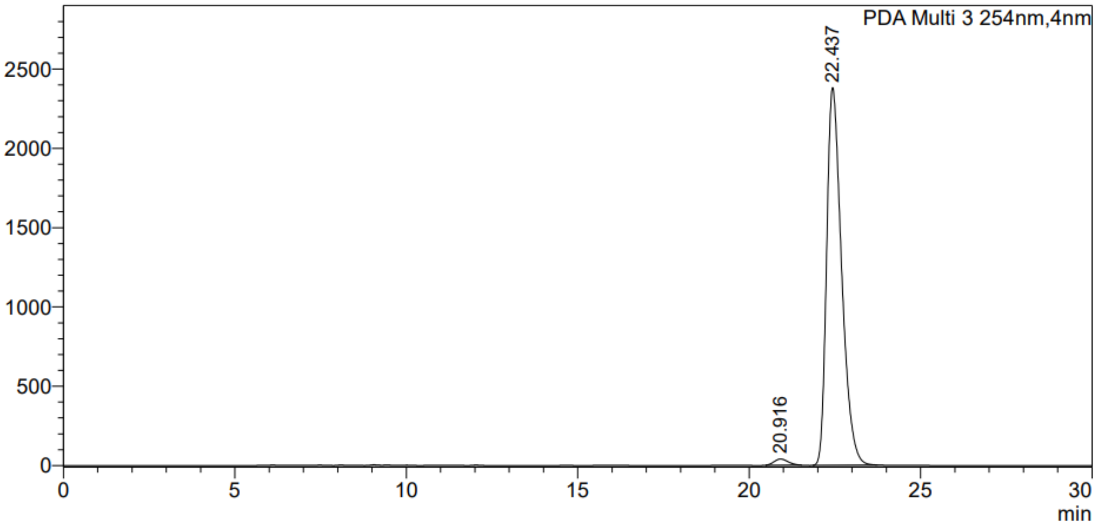

PDA Ch3 254nm

Peak#	Ret. Time	Height	Area	Area%
1	20.916	38764	1093245	1.493
2	22.437	2380462	72115780	98.507
Total		2419226	73209025	100.000

Compound 3h

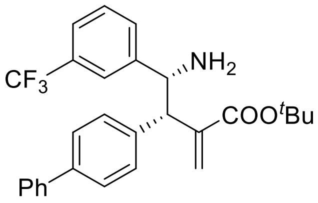

HPLC Conditions
Column: Chiralcel OD-H, Daicel Chemical Industries, Ltd.
Eluent: Hexanes/Isopropanol (94:6)
Flow rate: 0.5 mL/min
Detection: UV 254 nm

Racemic

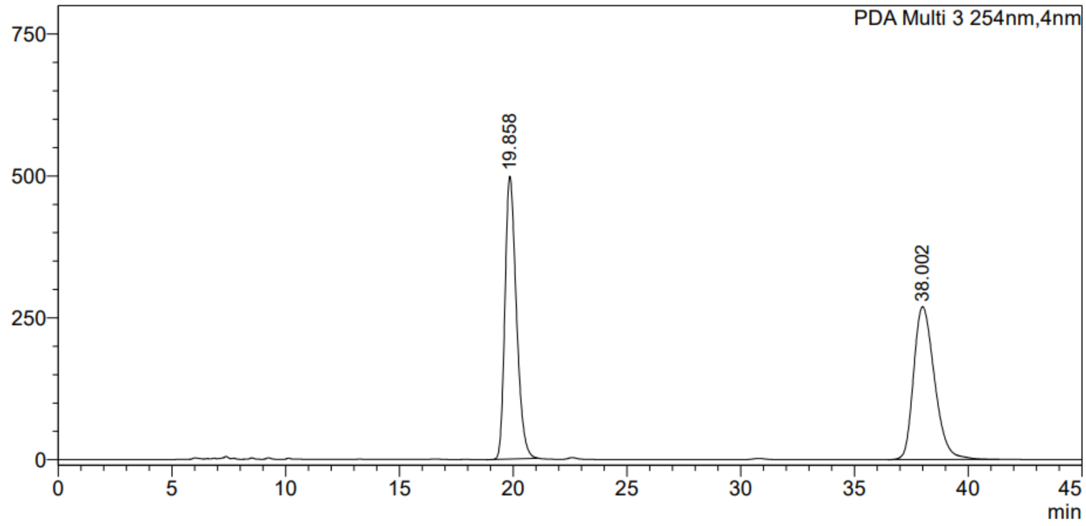

PDA Ch3 254nm

Peak#	Ret. Time	Height	Area	Area%
1	19.858	498276	17360878	50.203
2	38.002	269284	17220789	49.797
Total		767560	34581667	100.000

Chiral

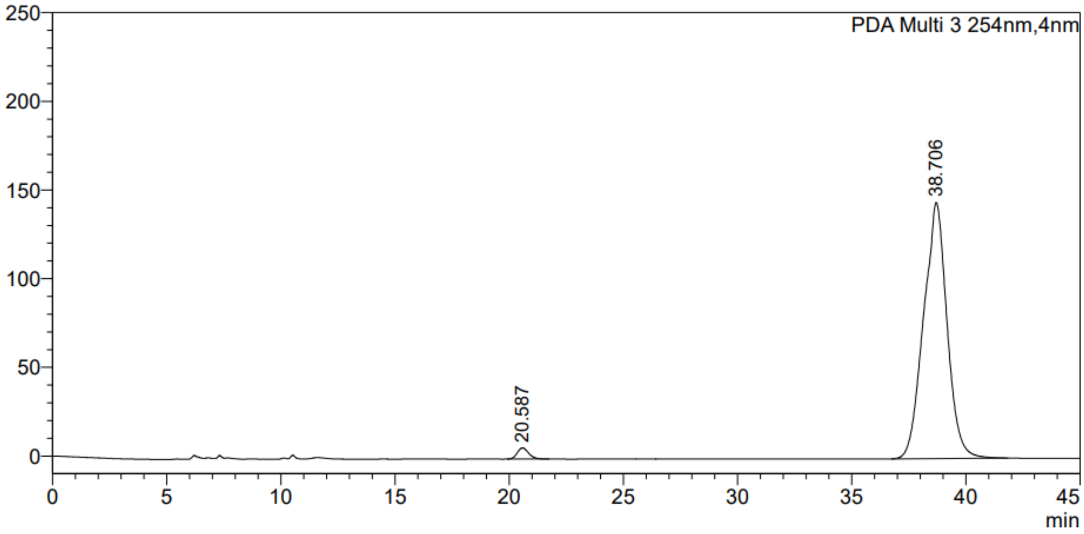

PDA Ch3 254nm

Peak#	Ret. Time	Height	Area	Area%
1	20.587	6286	218090	2.070
2	38.706	144404	10316158	97.930
Total		150691	10534248	100.000

Compound 3i

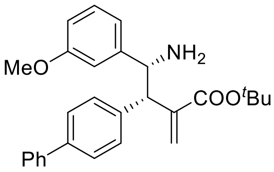

HPLC Conditions
Column: Chiralcel AD-H, Daicel Chemical Industries, Ltd.
Eluent: Hexanes/Isopropanol (94:6)
Flow rate: 0.5 mL/min
Detection: UV 254 nm

Racemic

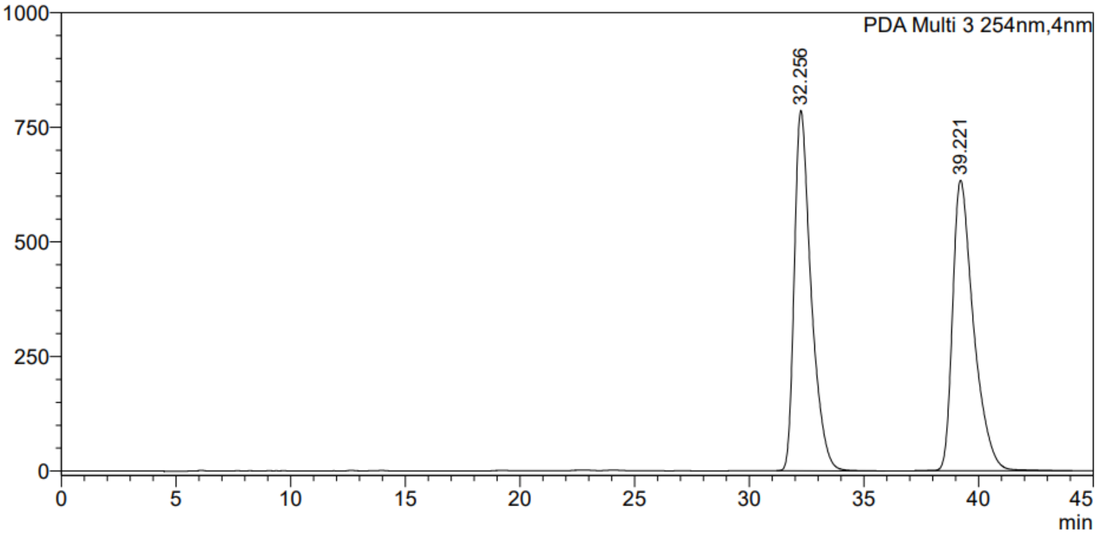

PDA Ch3 254nm

Peak#	Ret. Time	Height	Area	Area%
1	32.256	786126	40042450	49.803
2	39.221	634039	40360003	50.197
Total		1420165	80402453	100.000

Chiral

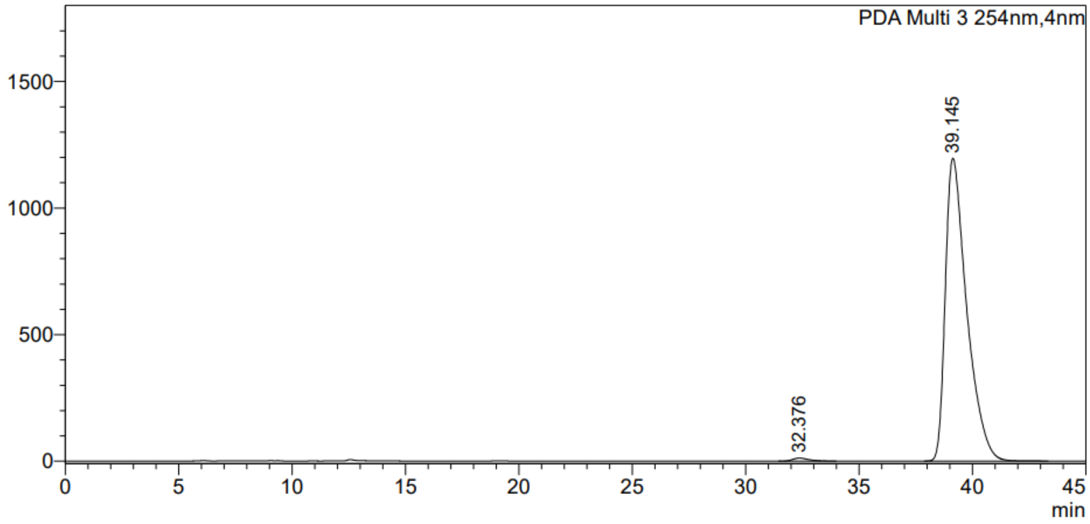

PDA Ch3 254nm

Peak#	Ret. Time	Height	Area	Area%
1	32.376	12127	606734	0.768
2	39.145	1195899	78386501	99.232
Total		1208026	78993235	100.000

Compound 3j

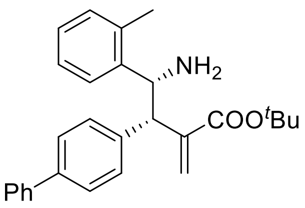

HPLC Conditions
Column: Chiralcel OD-H, Daicel Chemical Industries, Ltd.
Eluent: Hexanes/Isopropanol (94:6)
Flow rate: 0.6 mL/min
Detection: UV 254 nm

Racemic

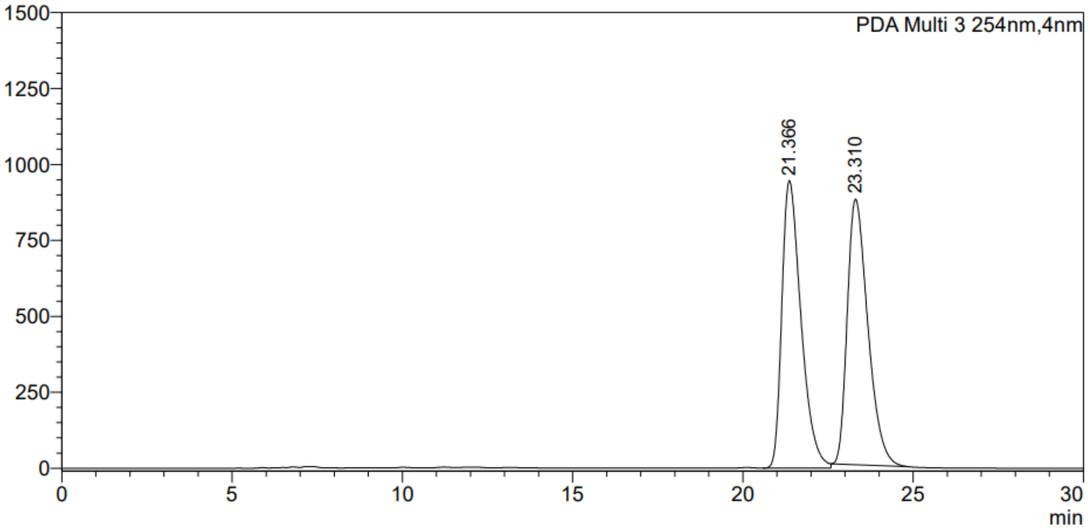

PDA Ch3 254nm

Peak#	Ret. Time	Height	Area	Area%
1	21.366	946514	37043879	50.191
2	23.310	874178	36761252	49.809
Total		1820692	73805131	100.000

Chiral

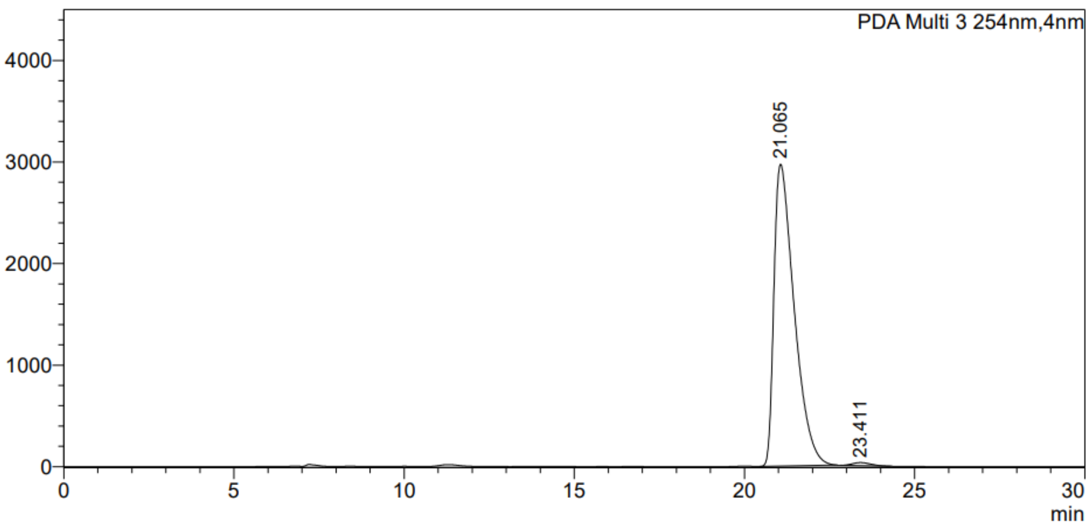

PDA Ch3 254nm

Peak#	Ret. Time	Height	Area	Area%
1	21.065	2972895	122885965	99.072
2	23.411	30302	1150510	0.928
Total		3003197	124036474	100.000

Compound 3k

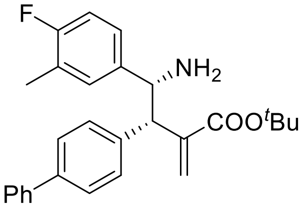

HPLC Conditions
Column: Chiralcel AD-H, Daicel Chemical Industries, Ltd.
Eluent: Hexanes/Isopropanol (94:6)
Flow rate: 0.5 mL/min
Detection: UV 254 nm

Racemic

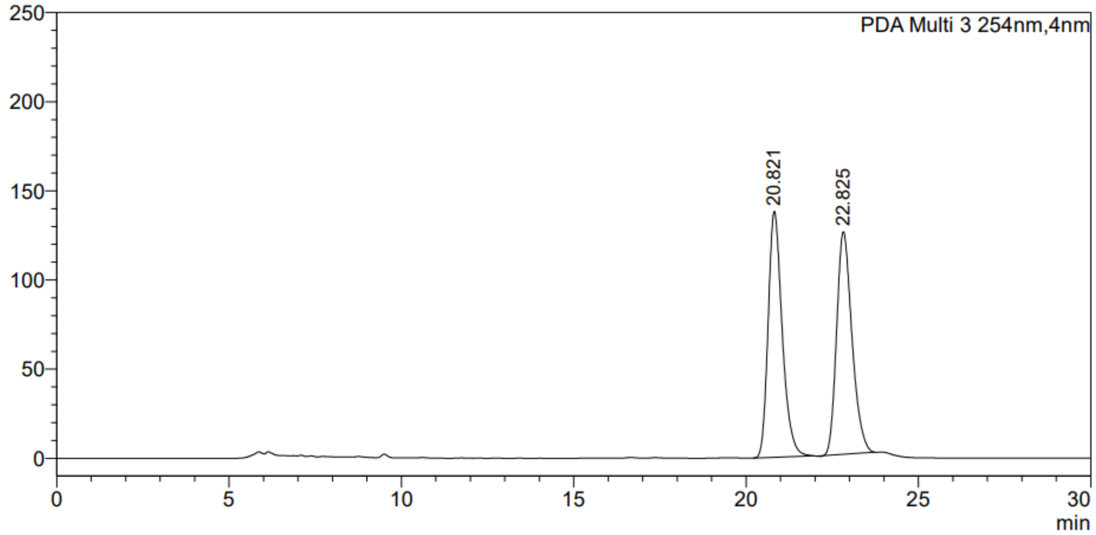

PDA Ch3 254nm

Peak#	Ret. Time	Height	Area	Area%
1	20.821	138058	3904704	50.246
2	22.825	124779	3866393	49.754
Total		262837	7771097	100.000

Chiral

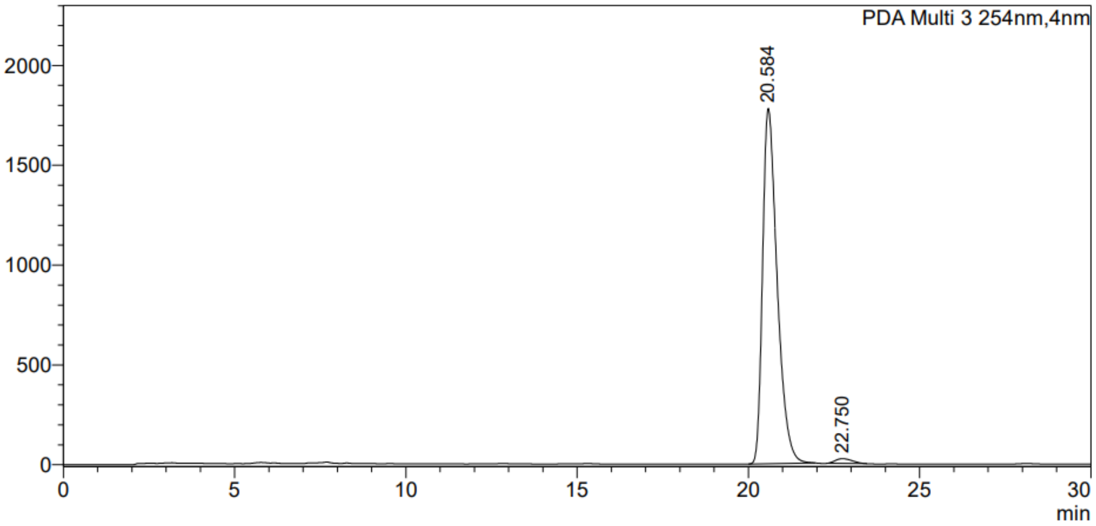

PDA Ch3 254nm

Peak#	Ret. Time	Height	Area	Area%
1	20.584	1779228	52571105	98.497
2	22.750	23726	802246	1.503
Total		1802954	53373352	100.000

Compound 3l

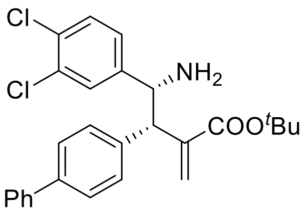

HPLC Conditions
Column: Chiralcel AD-H, Daicel Chemical Industries, Ltd.
Eluent: Hexanes/Isopropanol (94:6)
Flow rate: 0.5 mL/min
Detection: UV 254 nm

Racemic

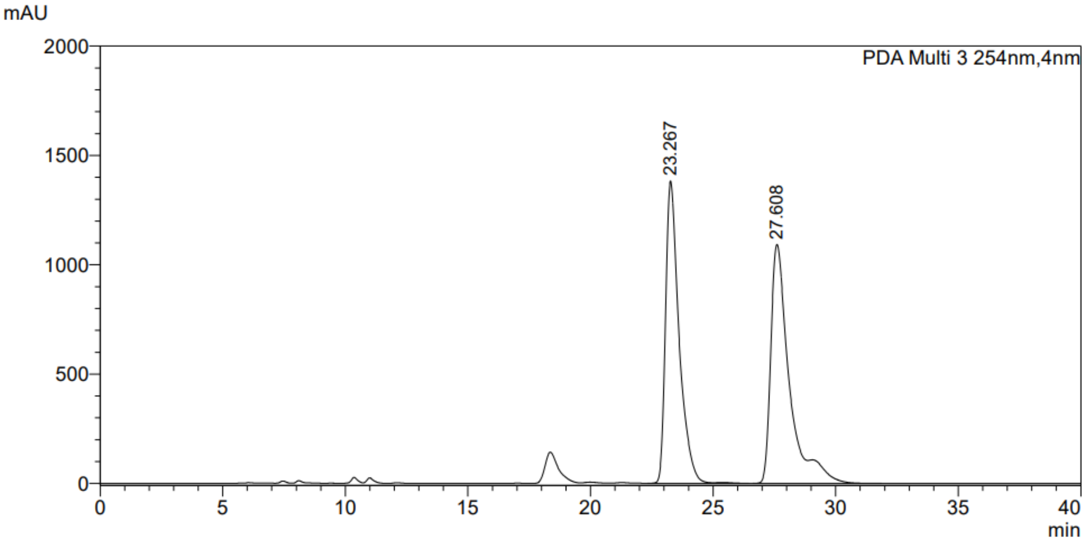

PDA Ch3 254nm

Peak#	Ret. Time	Height	Area	Area%
1	23.267	1382906	52970878	47.881
2	27.608	1092674	57660376	52.119
Total		2475580	110631254	100.000

Chiral

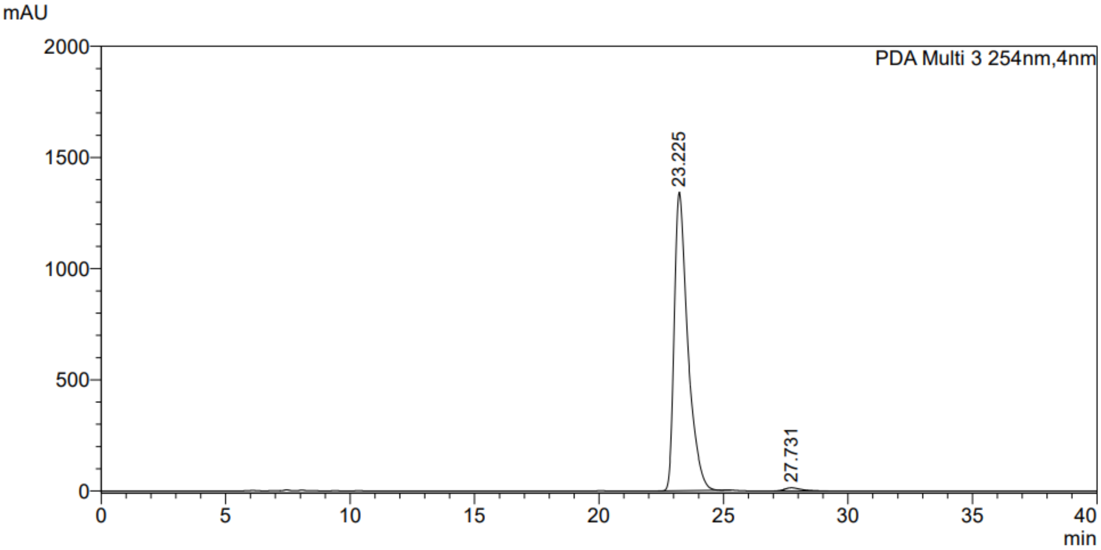

PDA Ch3 254nm

Peak#	Ret. Time	Height	Area	Area%
1	23.225	1342690	50718710	98.729
2	27.731	14373	653023	1.271
Total		1357064	51371733	100.000

Compound 3m

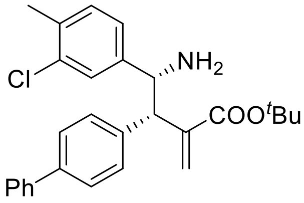

HPLC Conditions
Column: Chiralcel OD-H, Daicel Chemical Industries, Ltd.
Eluent: Hexanes/Isopropanol (94:6)
Flow rate: 0.6 mL/min
Detection: UV 254 nm

Racemic

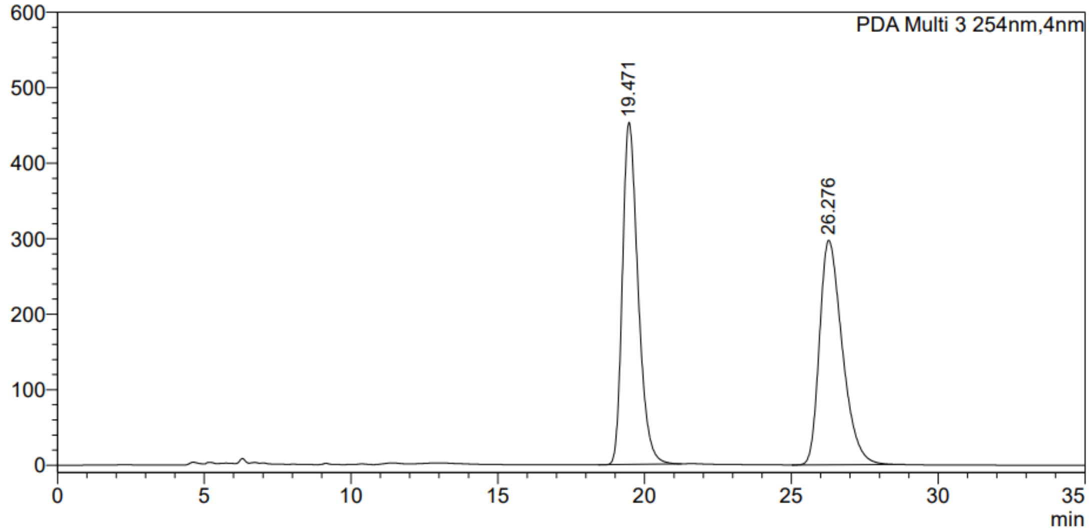

PDA Ch3 254nm

Peak#	Ret. Time	Height	Area	Area%
1	19.471	453172	16786500	51.096
2	26.276	297333	16066619	48.904
Total		750505	32853120	100.000

Chiral

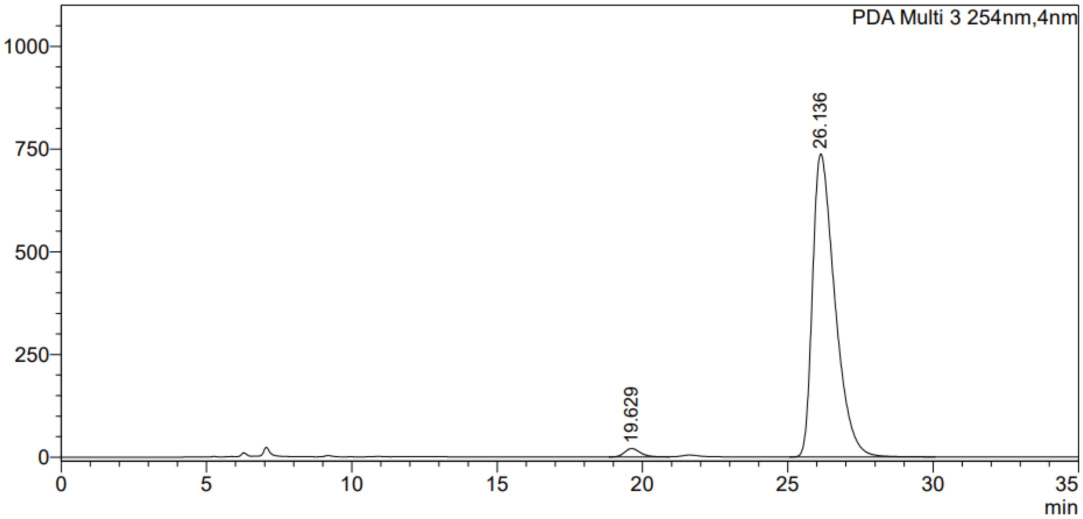

PDA Ch3 254nm

Peak#	Ret. Time	Height	Area	Area%
1	19.629	20599	785148	1.952
2	26.136	737886	39437578	98.048
Total		758485	40222726	100.000

Compound 3n

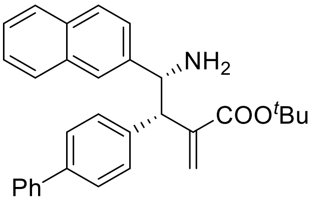

HPLC Conditions
Column: Chiralcel AD-H, Daicel Chemical Industries, Ltd.
Eluent: Hexanes/Isopropanol (94:6)
Flow rate: 0.5 mL/min
Detection: UV 254 nm

Racemic

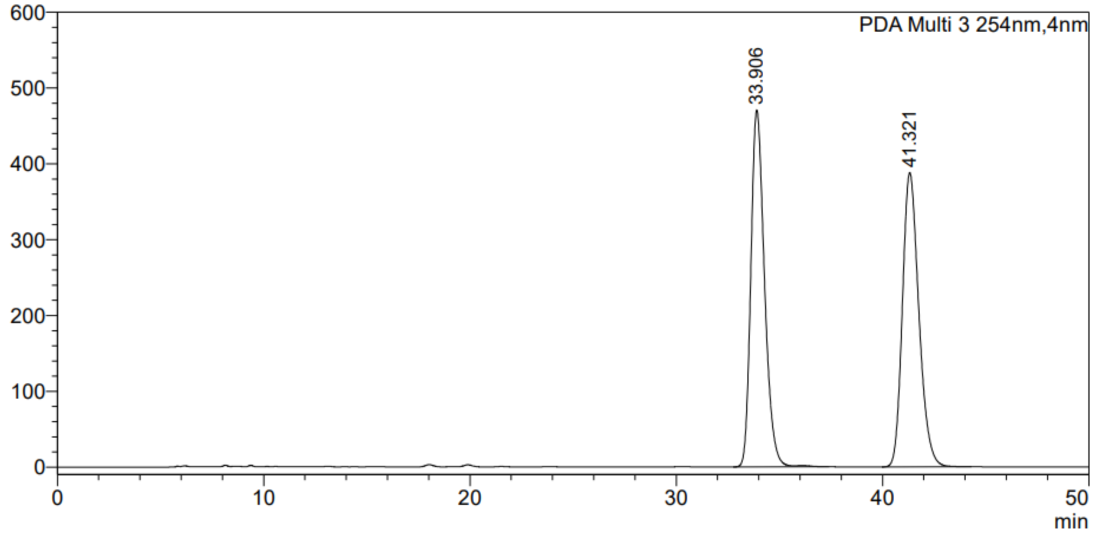

PDA Ch3 254nm

Peak#	Ret. Time	Height	Area	Area%
1	33.906	470765	21688029	50.240
2	41.321	388186	21480627	49.760
Total		858951	43168656	100.000

Chiral

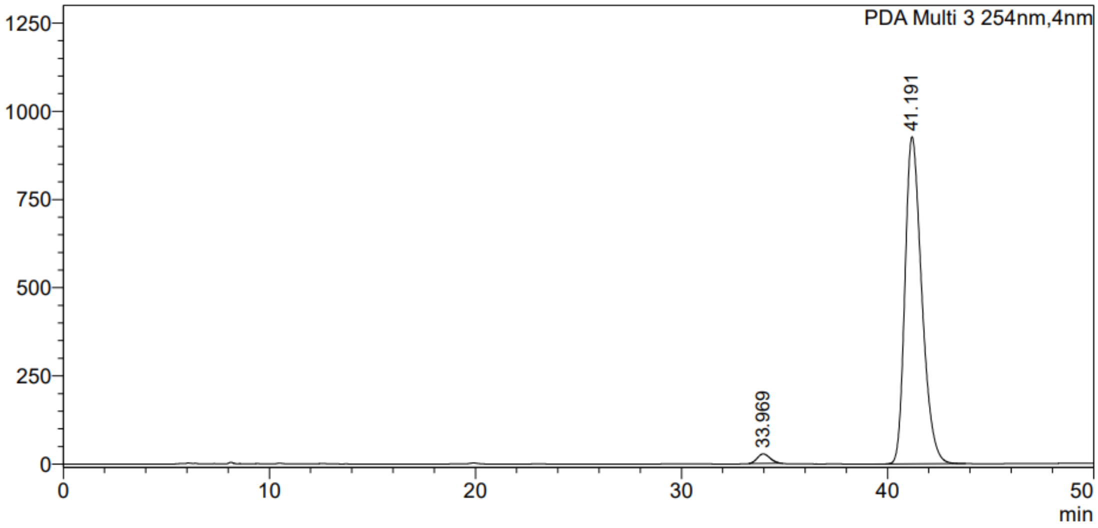

PDA Ch3 254nm

Peak#	Ret. Time	Height	Area	Area%
1	33.969	27192	1167490	2.178
2	41.191	927080	52428171	97.822
Total		954272	53595660	100.000

Compound 3o

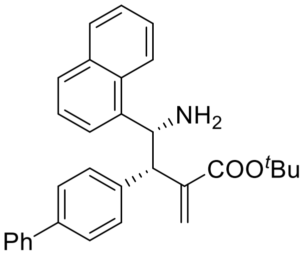

HPLC Conditions
Column: Chiralcel AD-H, Daicel Chemical Industries, Ltd.
Eluent: Hexanes/Isopropanol (94:6)
Flow rate: 0.5 mL/min
Detection: UV 254 nm

Racemic

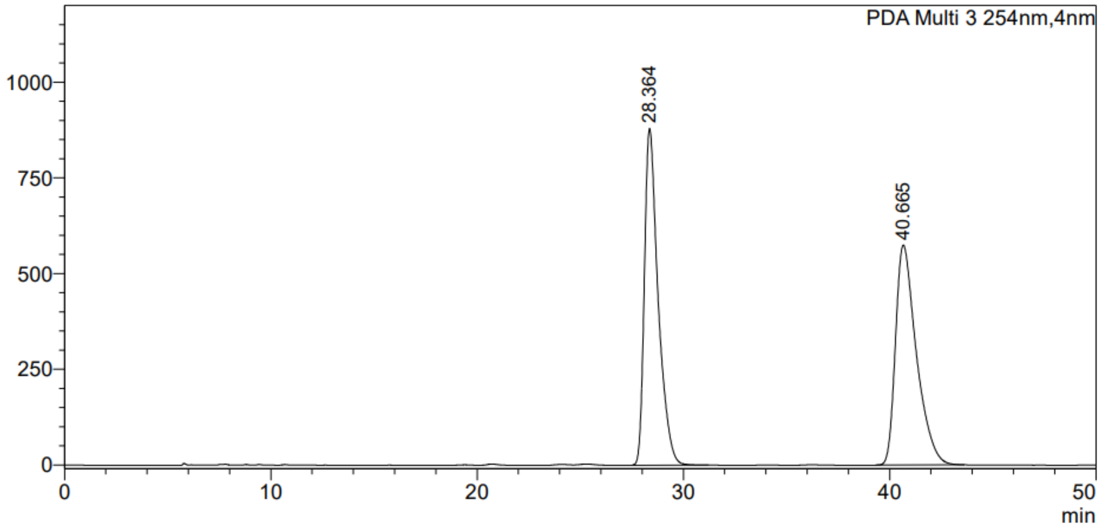

PDA Ch3 254nm

Peak#	Ret. Time	Height	Area	Area%
1	28.364	879512	40687735	50.165
2	40.665	574495	40419349	49.835
Total		1454006	81107084	100.000

Chiral

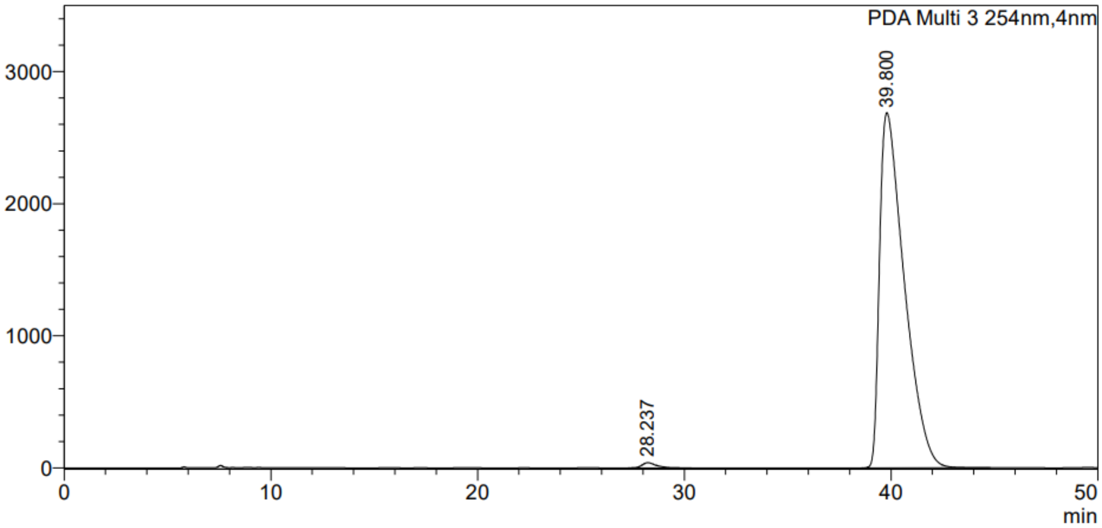

PDA Ch3 254nm

Peak#	Ret. Time	Height	Area	Area%
1	28.237	39143	1844967	0.820
2	39.800	2688305	223186967	99.180
Total		2727448	225031934	100.000

Compound 3p

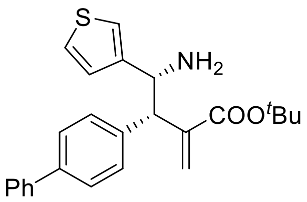

HPLC Conditions
Column: Chiralcel OJ-H, Daicel Chemical Industries, Ltd.
Eluent: Hexanes/Isopropanol (94:6)
Flow rate: 0.5 mL/min
Detection: UV 254 nm

Racemic

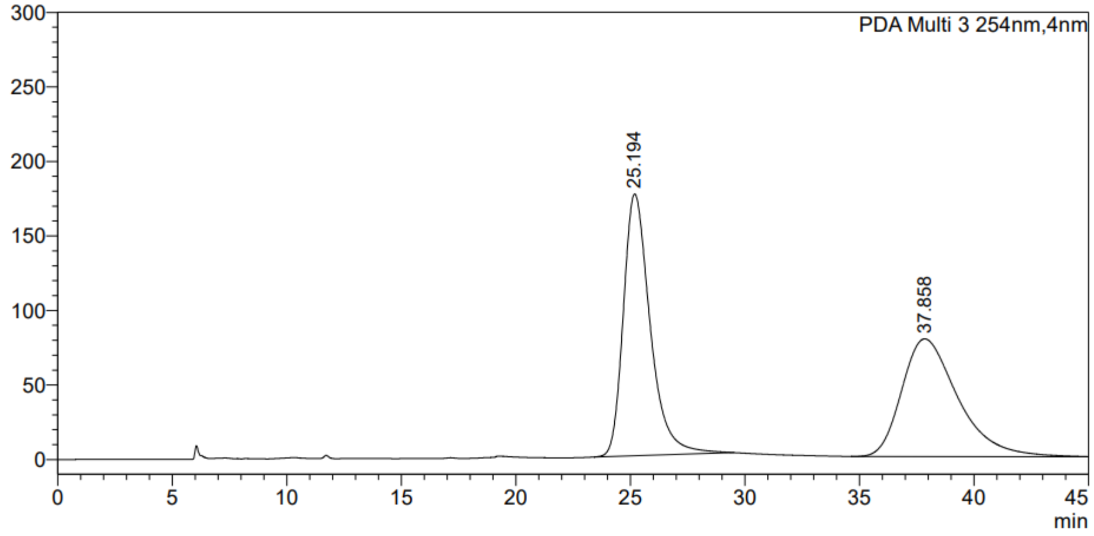

PDA Ch3 254nm

Peak#	Ret. Time	Height	Area	Area%
1	25.194	175604	14317282	51.539
2	37.858	78944	13462340	48.461
Total		254548	27779622	100.000

Chiral

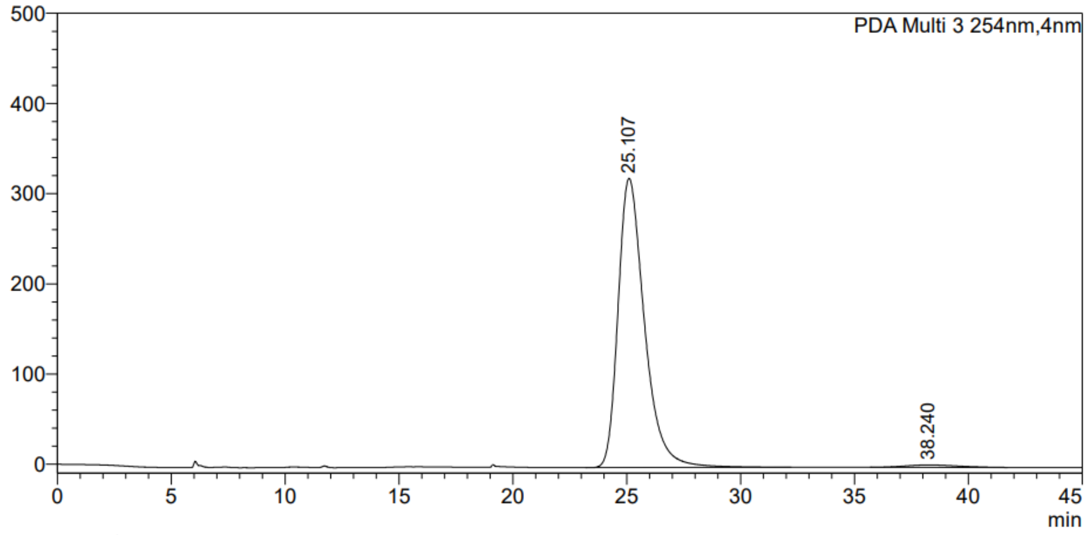

PDA Ch3 254nm

Peak#	Ret. Time	Height	Area	Area%
1	25.107	320629	25823770	98.346
2	38.240	2669	434348	1.654
Total		323298	26258118	100.000

Compound 3q

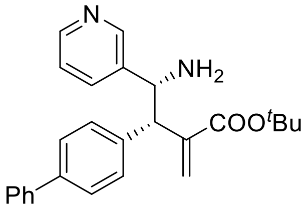

HPLC Conditions
Column: Chiralcel AD-H, Daicel Chemical Industries, Ltd.
Eluent: Hexanes/Isopropanol (90:10)
Flow rate: 1.0 mL/min
Detection: UV 254 nm

Racemic

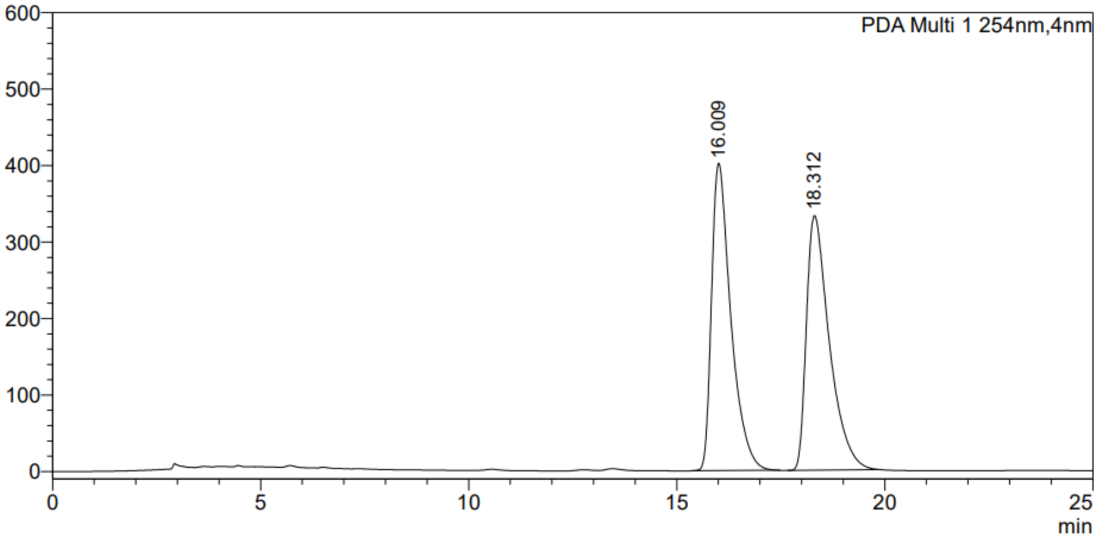

PDA Ch1 254nm

Peak#	Ret. Time	Height	Area	Area%
1	16.009	401900	12977101	50.352
2	18.312	333301	12795699	49.648
Total		735202	25772800	100.000

Chiral

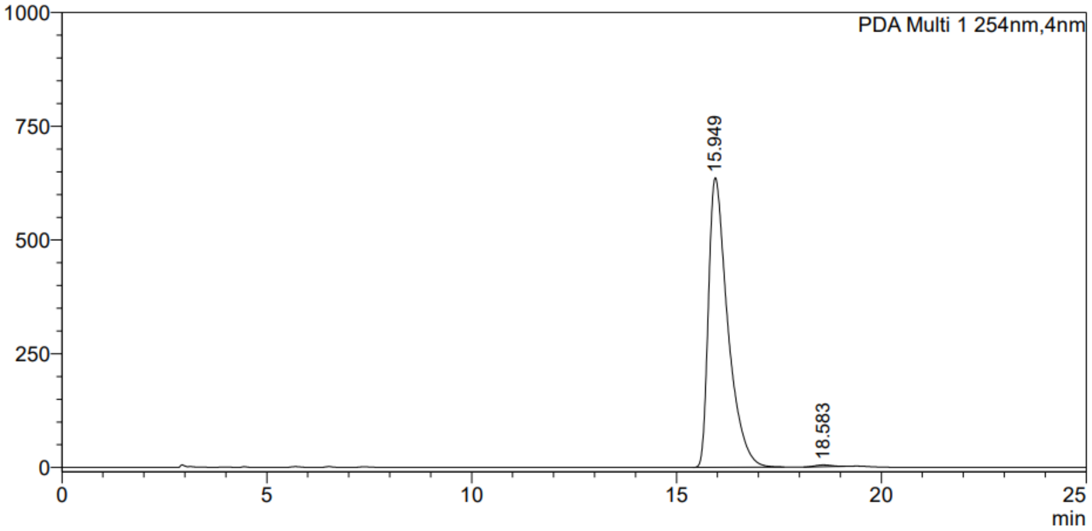

PDA Ch1 254nm

Peak#	Ret. Time	Height	Area	Area%
1	15.949	636370	20813524	99.460
2	18.583	4003	112987	0.540
Total		640372	20926511	100.000

Compound 3r

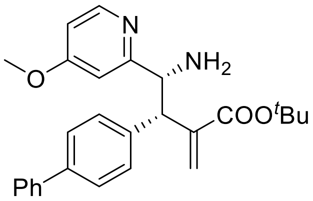

HPLC Conditions
Column: Chiralcel OJ-H, Daicel Chemical Industries, Ltd.
Eluent: Hexanes/Isopropanol (94:6)
Flow rate: 0.5 mL/min
Detection: UV 254 nm

Racemic

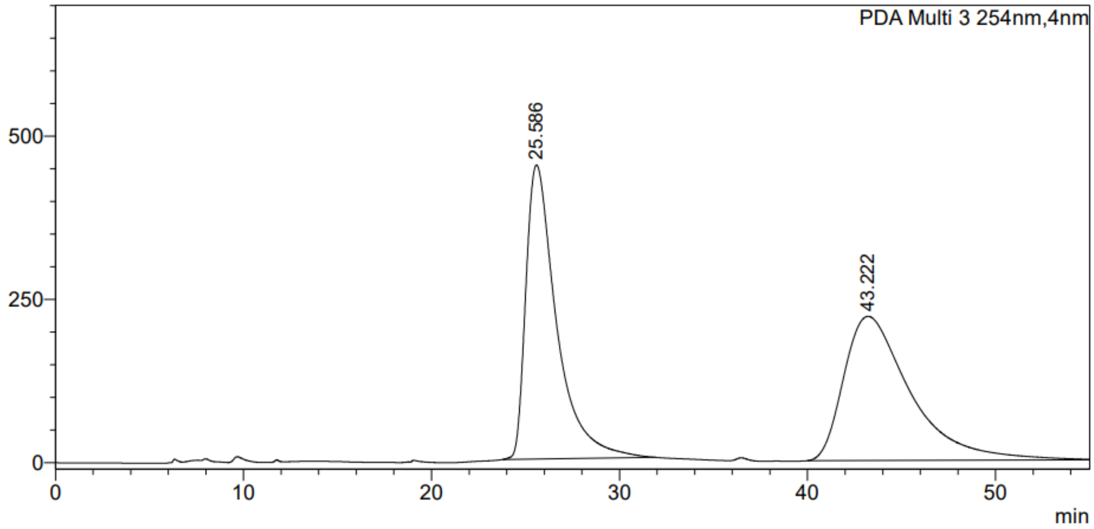

PDA Ch3 254nm

Peak#	Ret. Time	Height	Area	Area%
1	25.586	450056	51054562	48.461
2	43.222	220630	54297136	51.539
Total		670687	105351698	100.000

Chiral

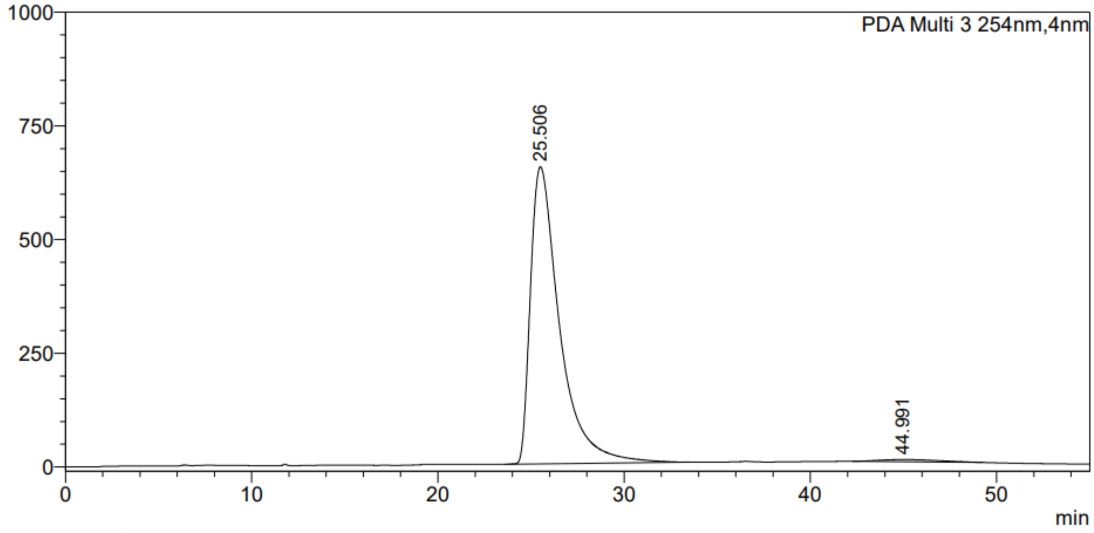

PDA Ch3 254nm

Peak#	Ret. Time	Height	Area	Area%
1	25.506	653533	71286160	98.495
2	44.991	4735	1088952	1.505
Total		658268	72375112	100.000

Compound 3s

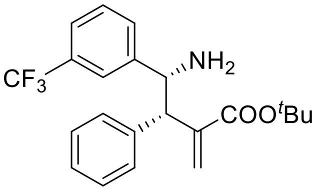

HPLC Conditions
Column: Chiralcel AD-H, Daicel Chemical Industries, Ltd.
Eluent: Hexanes/Isopropanol (97:3)
Flow rate: 0.3 mL/min
Detection: UV 254 nm

Racemic

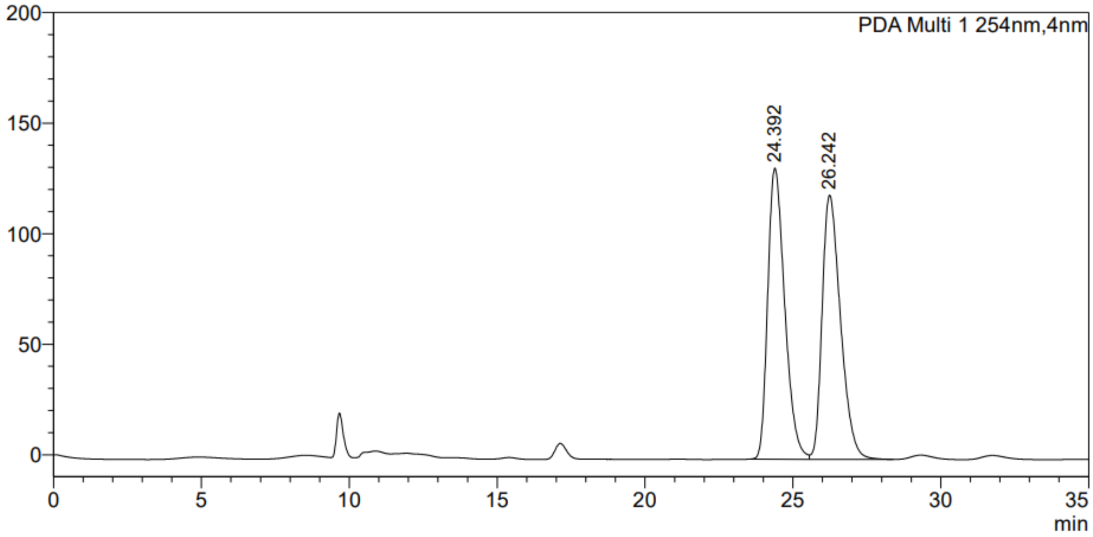

PDA Ch1 254nm

Peak#	Ret. Time	Height	Area	Area%
1	24.392	131665	5263053	50.481
2	26.242	119497	5162699	49.519
Total		251162	10425752	100.000

Chiral

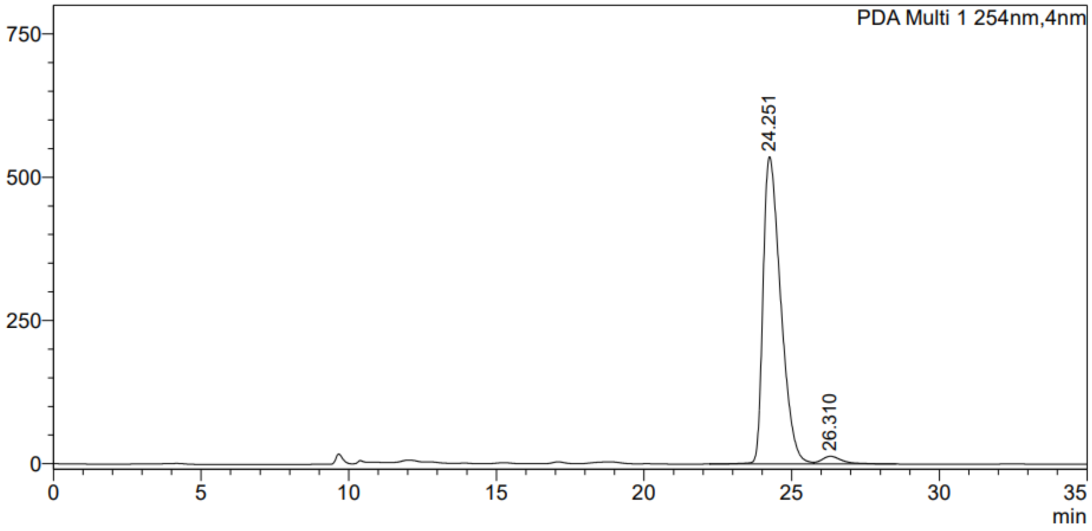

PDA Ch1 254nm

Peak#	Ret. Time	Height	Area	Area%
1	24.251	536082	22316592	97.103
2	26.310	13511	665701	2.897
Total		549593	22982293	100.000

Compound 3t

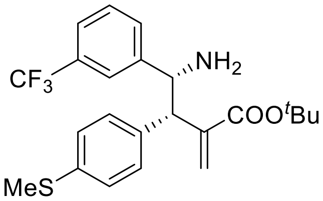

HPLC Conditions
Column: Chiralcel AD-H, Daicel Chemical Industries, Ltd.
Eluent: Hexanes/Isopropanol (94:6)
Flow rate: 0.5 mL/min
Detection: UV 254 nm

Racemic

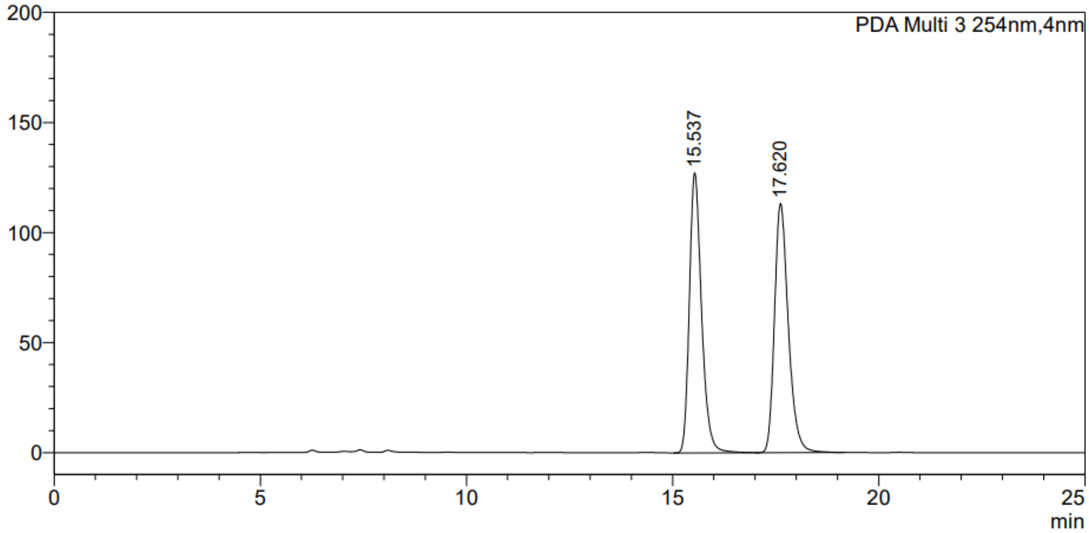

PDA Ch3 254nm

Peak#	Ret. Time	Height	Area	Area%
1	15.537	127151	2613359	49.703
2	17.620	113269	2644635	50.297
Total		240421	5257994	100.000

Chiral

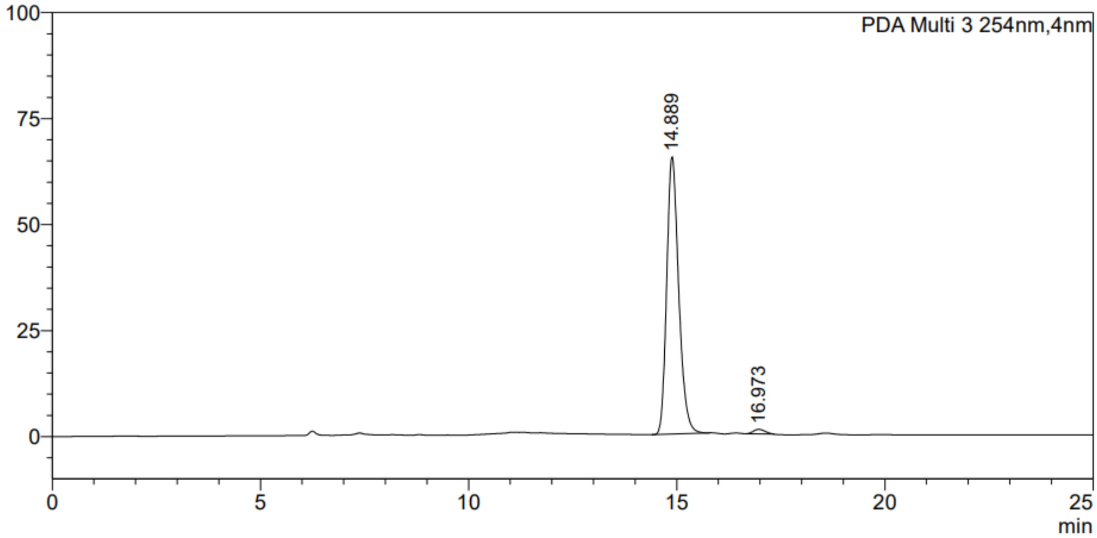

PDA Ch3 254nm

Peak#	Ret. Time	Height	Area	Area%
1	14.889	65383	1309185	98.438
2	16.973	1084	20769	1.562
Total		66467	1329954	100.000

Compound 3u

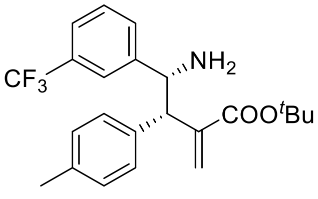

HPLC Conditions
Column: Chiralcel OD-H, Daicel Chemical Industries, Ltd.
Eluent: Hexanes/Isopropanol (97:3)
Flow rate: 0.3 mL/min
Detection: UV 254 nm

Racemic

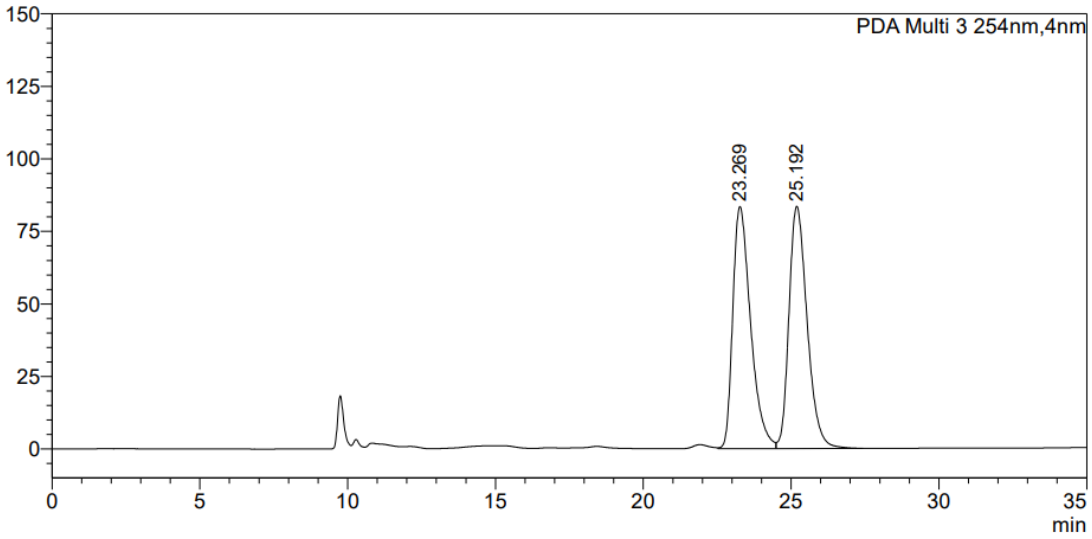

PDA Ch3 254nm

Peak#	Ret. Time	Height	Area	Area%
1	23.269	83374	3506574	49.565
2	25.192	83445	3568133	50.435
Total		166819	7074707	100.000

Chiral

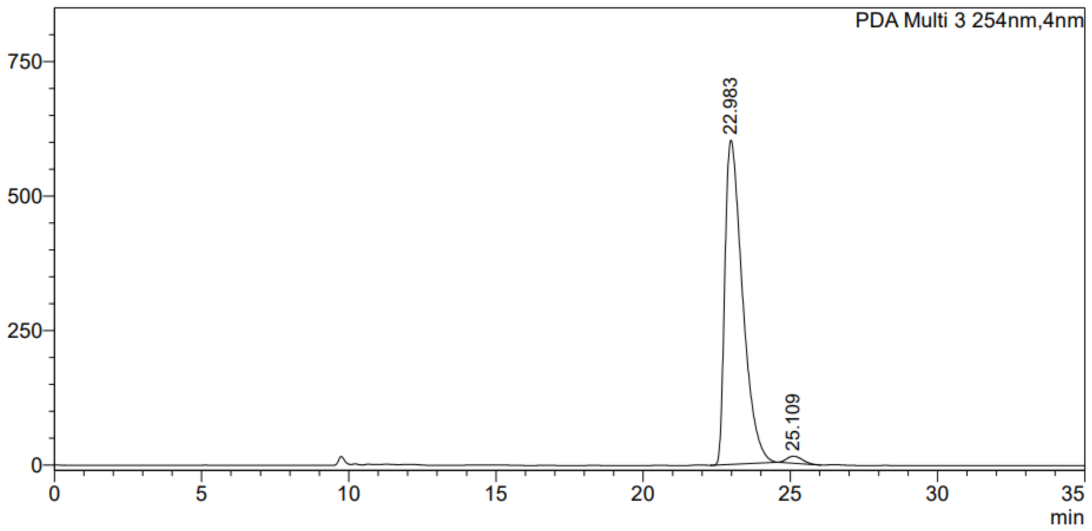

PDA Ch3 254nm

Peak#	Ret. Time	Height	Area	Area%
1	22.983	602377	25195742	98.132
2	25.109	12983	479746	1.868
Total		615360	25675487	100.000

Compound 3v

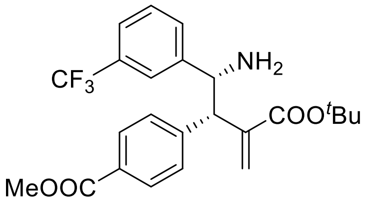

HPLC Conditions
Column: Chiralcel AD-H, Daicel Chemical Industries, Ltd.
Eluent: Hexanes/Isopropanol (96:4)
Flow rate: 0.3 mL/min
Detection: UV 254 nm

Racemic

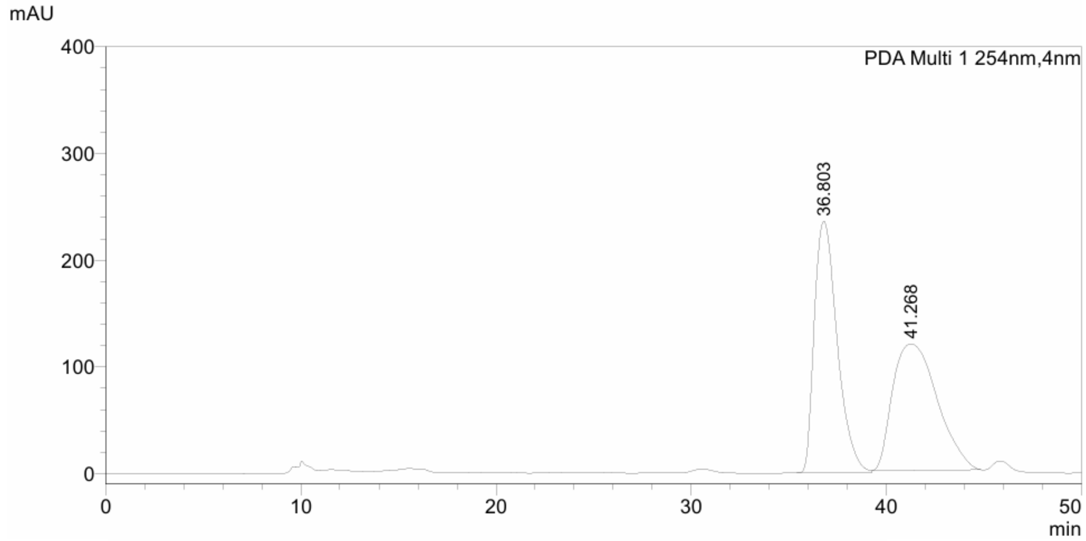

PDA Ch1 254nm

Peak#	Ret. Time	Height	Area	Area%
1	36.803	235843	19257724	50.618
2	41.268	118354	18787593	49.382
Total		354197	38045317	100.000

Chiral

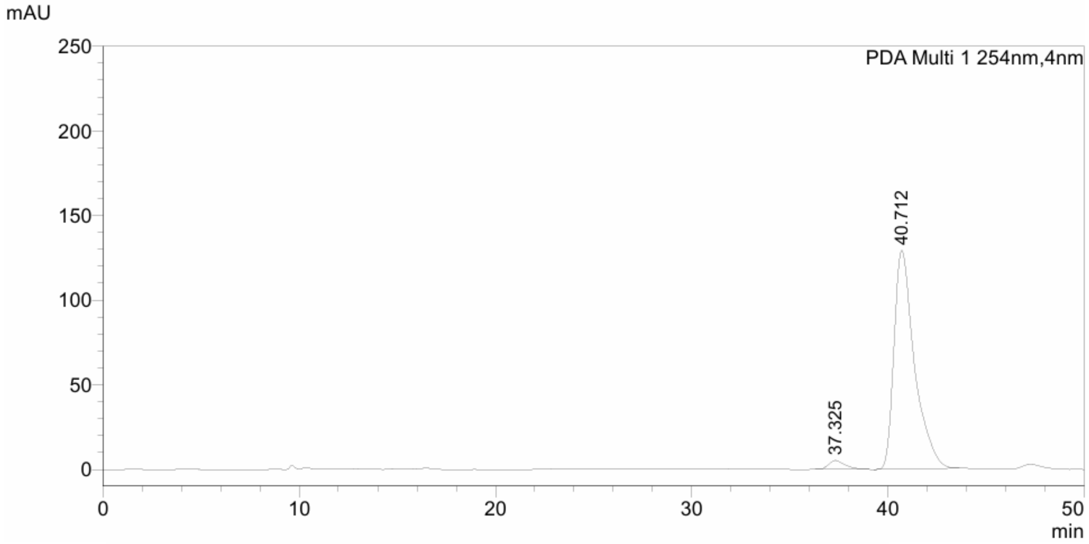

PDA Ch1 254nm

Peak#	Ret. Time	Height	Area	Area%
1	37.325	5252	305324	3.159
2	40.712	129223	9361037	96.841
Total		134475	9666362	100.000

Compound 3w

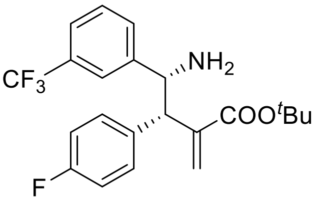

HPLC Conditions
Column: Chiralcel OJ-H, Daicel Chemical Industries, Ltd.
Eluent: Hexanes/Isopropanol (97:3)
Flow rate: 0.5 mL/min
Detection: UV 254 nm

Racemic

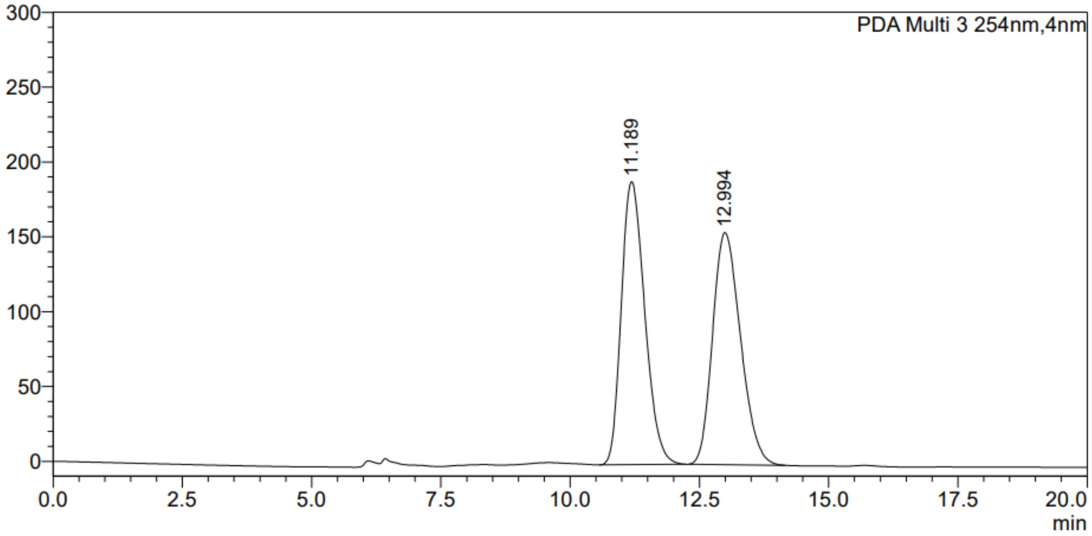

PDA Ch3 254nm

Peak#	Ret. Time	Height	Area	Area%
1	11.189	189073	5954779	50.549
2	12.994	155059	5825533	49.451
Total		344132	11780312	100.000

Chiral

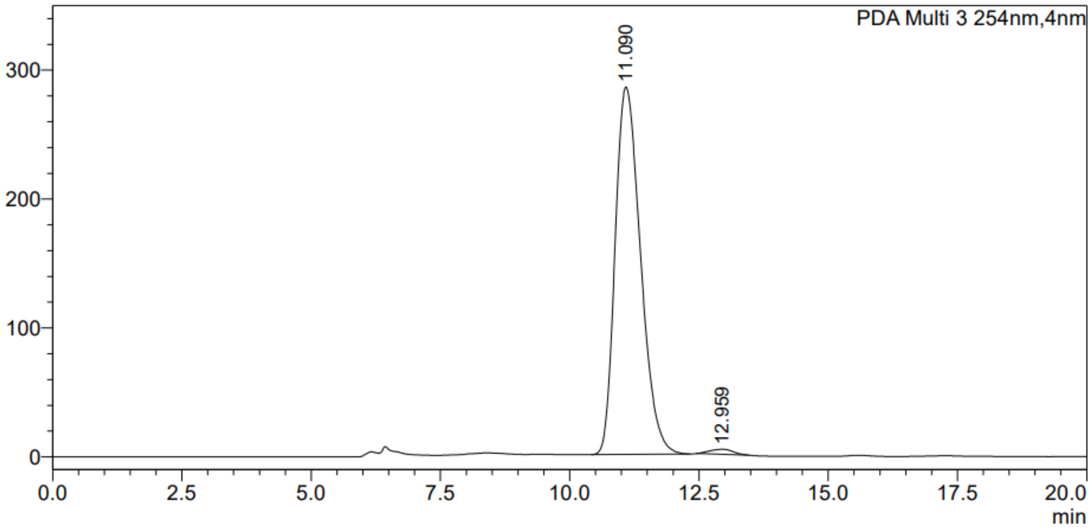

PDA Ch3 254nm

Peak#	Ret. Time	Height	Area	Area%
1	11.090	284936	9724829	98.759
2	12.959	3922	122218	1.241
Total		288858	9847047	100.000

Compound 3x

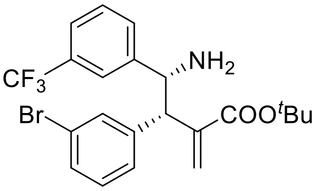

HPLC Conditions
Column: Chiralcel OD-H, Daicel Chemical Industries, Ltd.
Eluent: Hexanes/Isopropanol (94:6)
Flow rate: 0.6 mL/min
Detection: UV 230 nm

Racemic

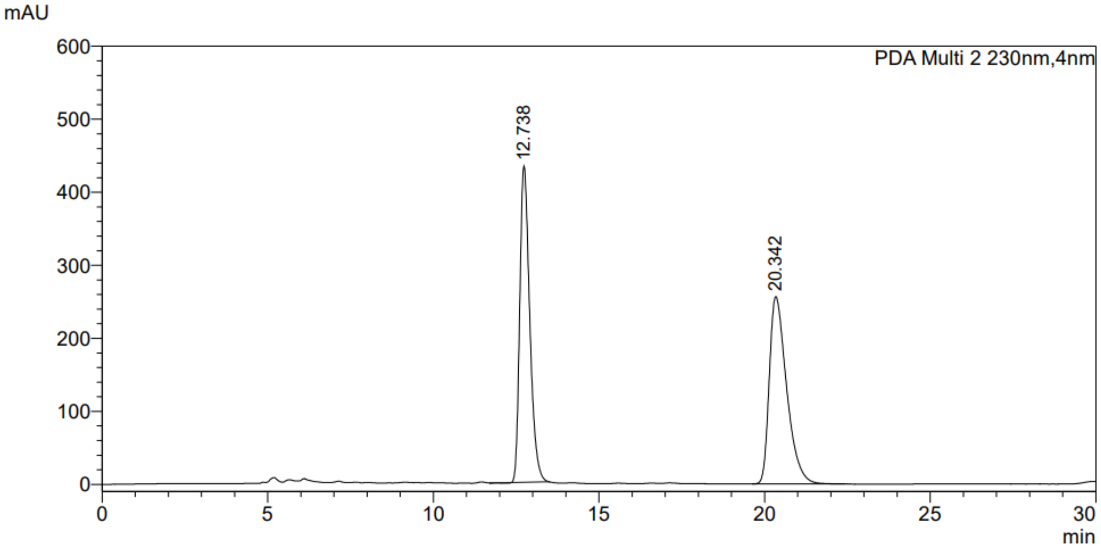

PDA Ch2 230nm

Peak#	Ret. Time	Height	Area	Area%
1	12.738	433175	9042457	49.164
2	20.342	256737	9350050	50.836
Total		689911	18392507	100.000

Chiral

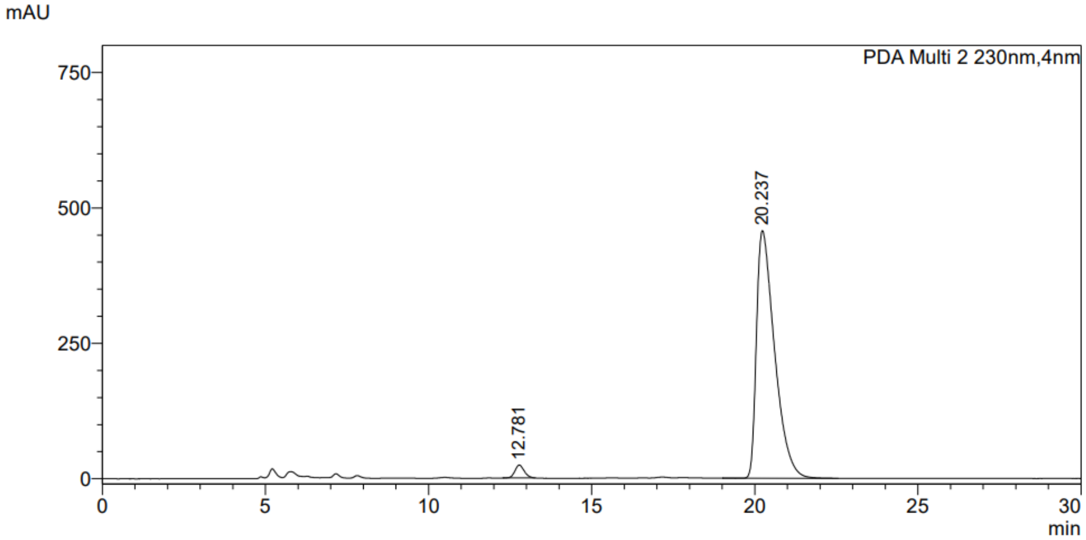

PDA Ch2 230nm

Peak#	Ret. Time	Height	Area	Area%
1	12.781	23739	467966	2.583
2	20.237	457273	17646468	97.417
Total		481012	18114433	100.000

Compound 3y

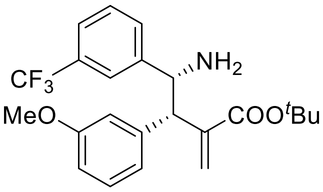

HPLC Conditions
Column: Chiralcel AD-H, Daicel Chemical Industries, Ltd.
Eluent: Hexanes/Isopropanol (94:6)
Flow rate: 0.5 mL/min
Detection: UV 254 nm

Racemic

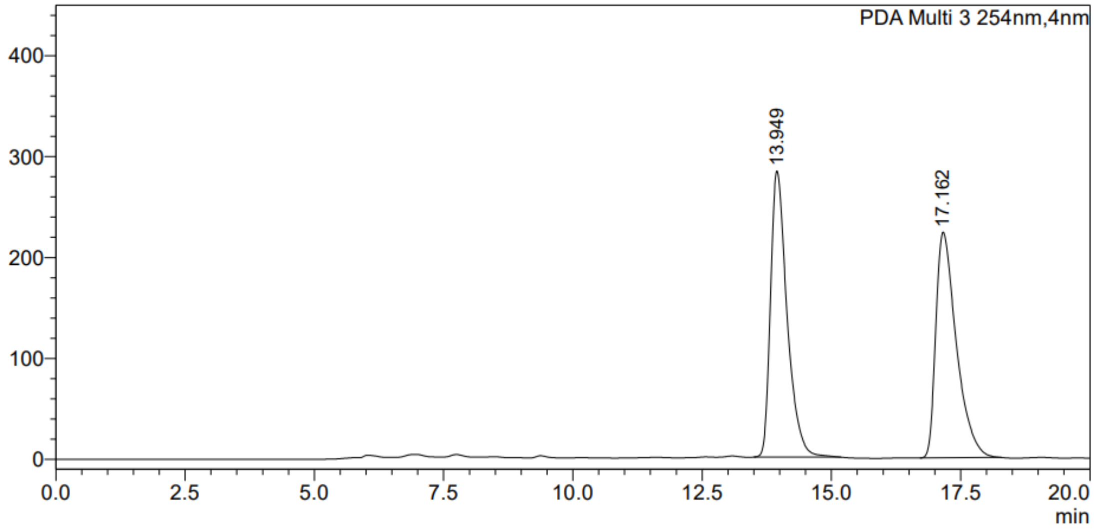

PDA Ch3 254nm

Peak#	Ret. Time	Height	Area	Area%
1	13.949	283510	6315741	50.553
2	17.162	223814	6177524	49.447
Total		507324	12493265	100.000

Chiral

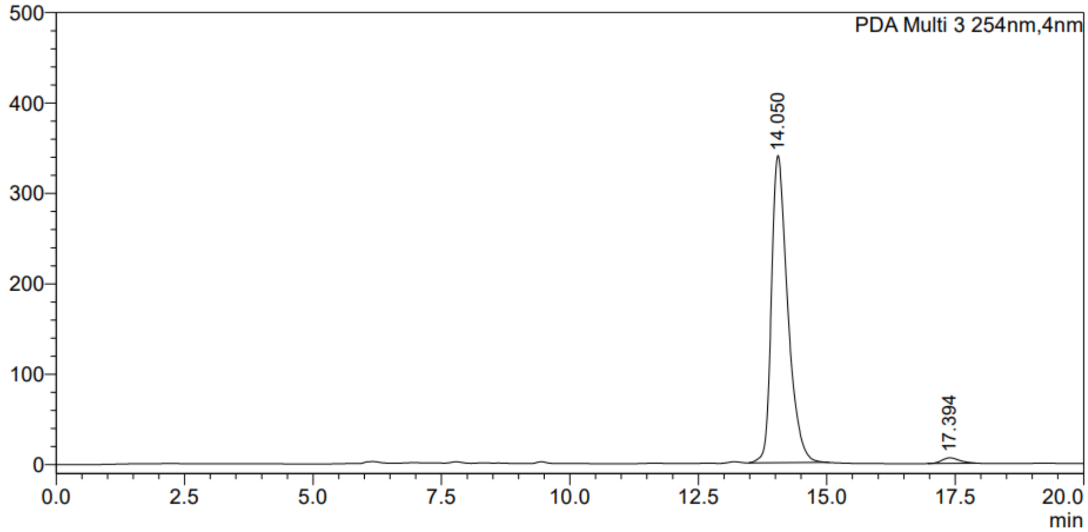

PDA Ch3 254nm

Peak#	Ret. Time	Height	Area	Area%
1	14.050	339694	7482070	98.094
2	17.394	6089	145408	1.906
Total		345783	7627479	100.000

Compound 3z

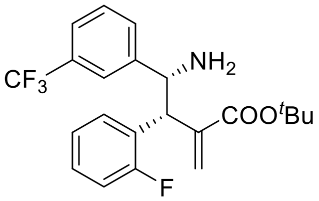

HPLC Conditions
Column: Chiralcel AD-H, Daicel Chemical Industries, Ltd.
Eluent: Hexanes/Isopropanol (94:6)
Flow rate: 0.5 mL/min
Detection: UV 254 nm

Racemic

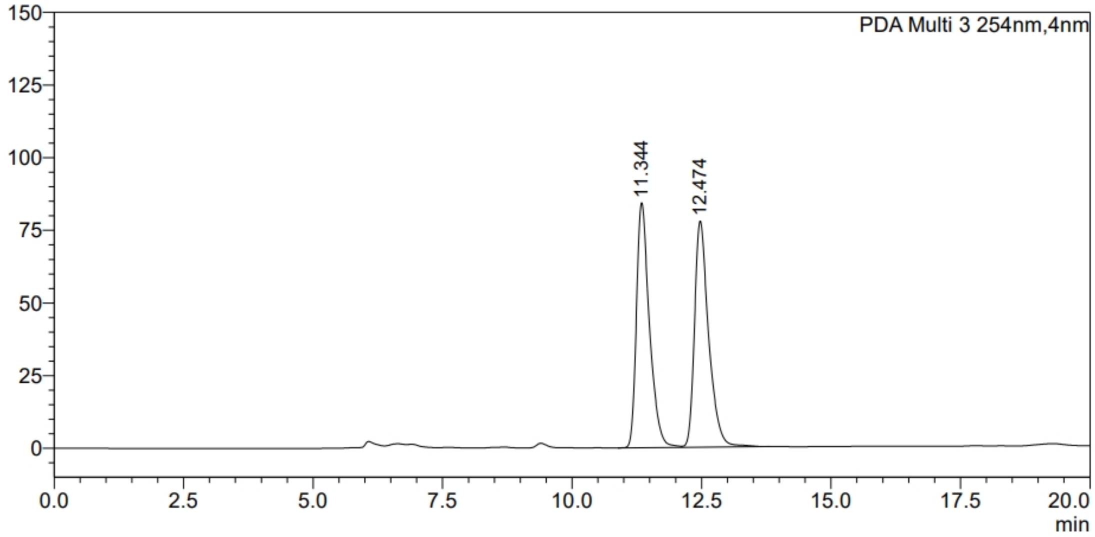

PDA Ch3 254nm

Peak#	Ret. Time	Height	Area	Area%
1	11.344	84270	1469739	49.851
2	12.474	77766	1478543	50.149
Total		162037	2948282	100.000

Chiral

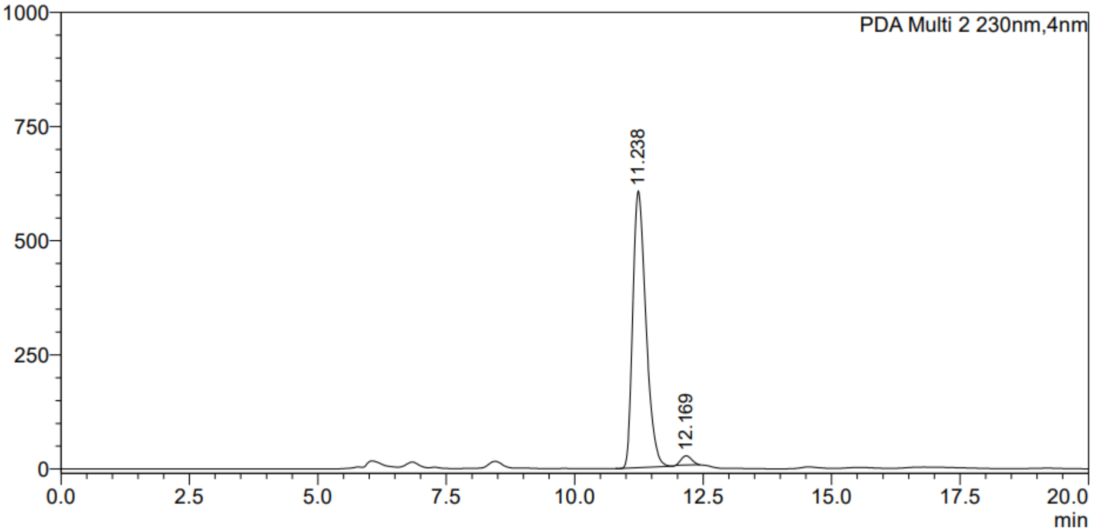

PDA Ch2 230nm

Peak#	Ret. Time	Height	Area	Area%
1	11.238	605906	10786668	97.453
2	12.169	19986	281875	2.547
Total		625892	11068543	100.000

Compound 3aa

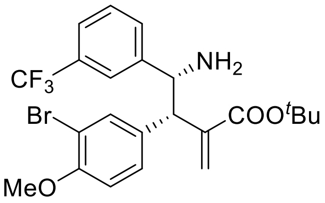

HPLC Conditions
Column: Chiralcel AD-H, Daicel Chemical Industries, Ltd.
Eluent: Hexanes/Isopropanol (94:6)
Flow rate: 0.5 mL/min
Detection: UV 254 nm

Racemic

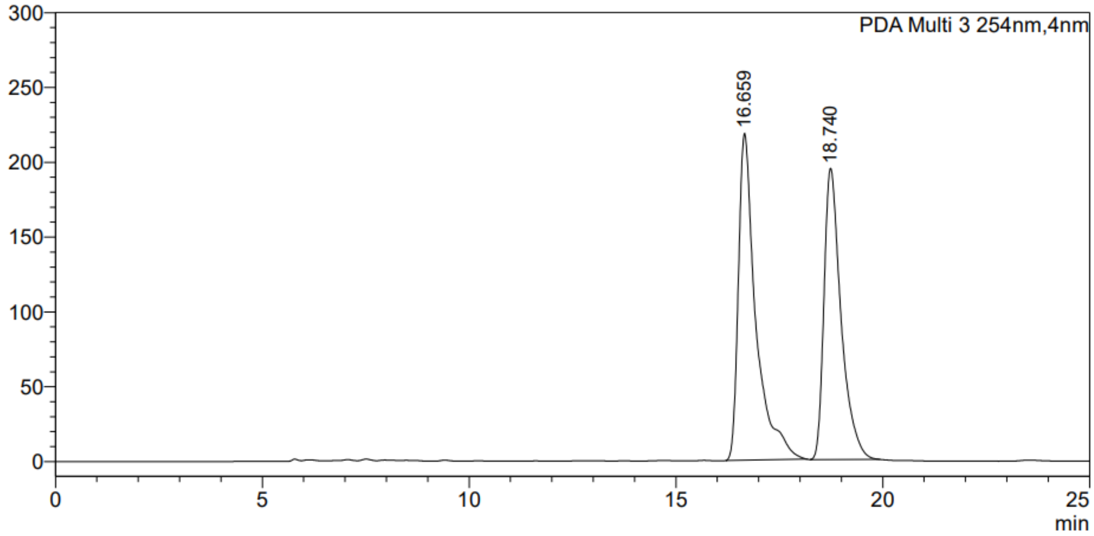

PDA Ch3 254nm

Peak#	Ret. Time	Height	Area	Area%
1	16.659	218268	6405469	53.487
2	18.740	194507	5570273	46.513
Total		412775	11975742	100.000

Chiral

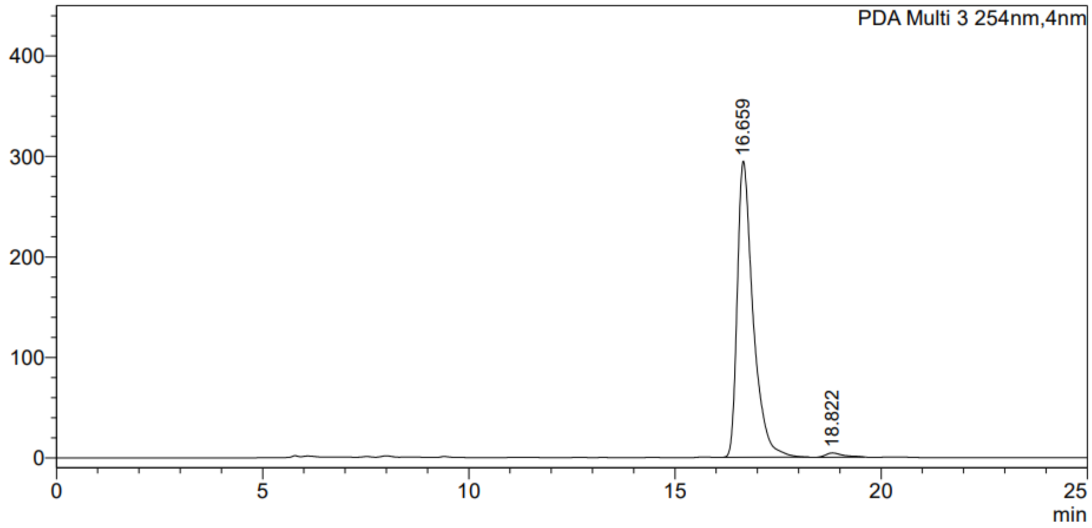

PDA Ch3 254nm

Peak#	Ret. Time	Height	Area	Area%
1	16.659	294701	7867303	98.480
2	18.822	4203	121464	1.520
Total		298904	7988768	100.000

Compound 3ab

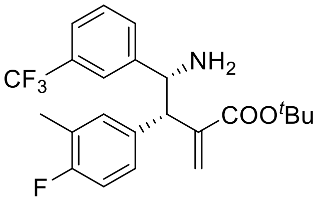

HPLC Conditions
Column: Chiralcel AD-H, Daicel Chemical Industries, Ltd.
Eluent: Hexanes/Isopropanol (96:4)
Flow rate: 0.3 mL/min
Detection: UV 254 nm

Racemic

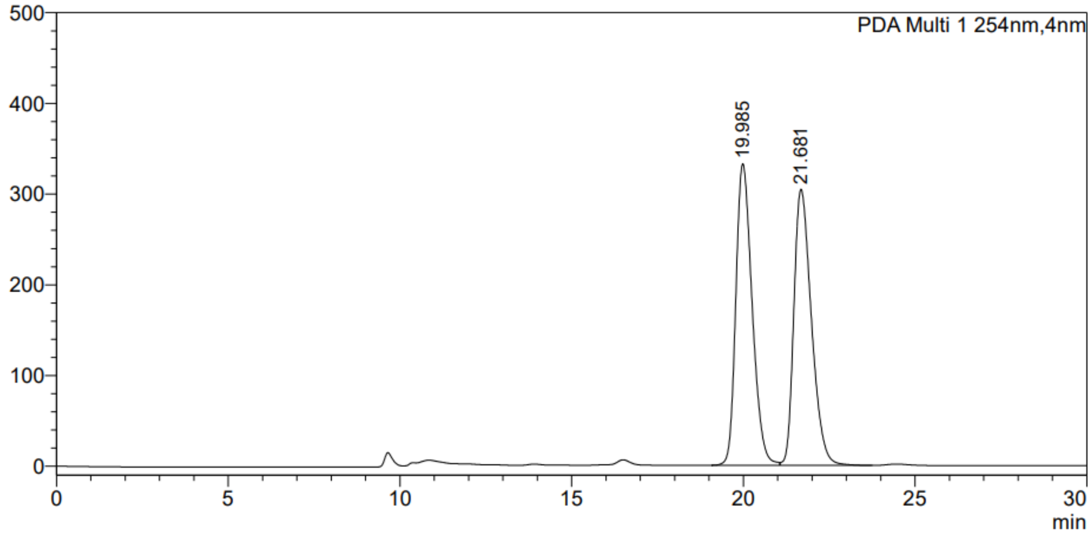

PDA Ch1 254nm

Peak#	Ret. Time	Height	Area	Area%
1	19.985	332257	10961023	50.341
2	21.681	304256	10812637	49.659
Total		636513	21773660	100.000

Chiral

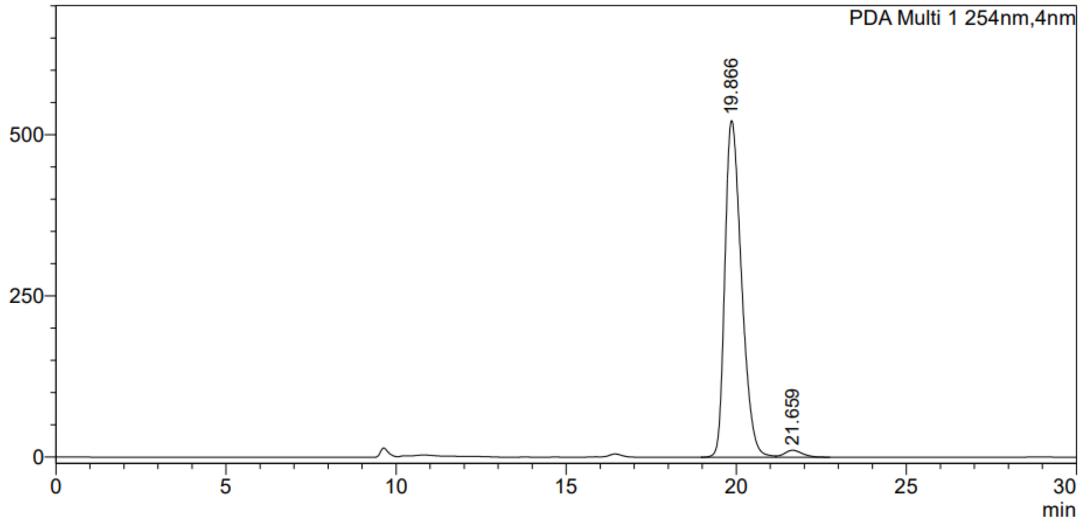

PDA Ch1 254nm

Peak#	Ret. Time	Height	Area	Area%
1	19.866	521943	17751733	97.843
2	21.659	10709	391337	2.157
Total		532652	18143070	100.000

Compound 3ac

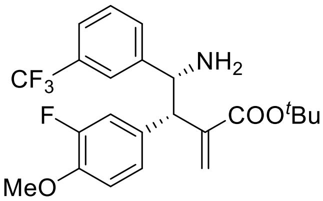

HPLC Conditions
Column: Chiralcel AD-H, Daicel Chemical Industries, Ltd.
Eluent: Hexanes/Isopropanol (94:6)
Flow rate: 0.5 mL/min
Detection: UV 254 nm

Racemic

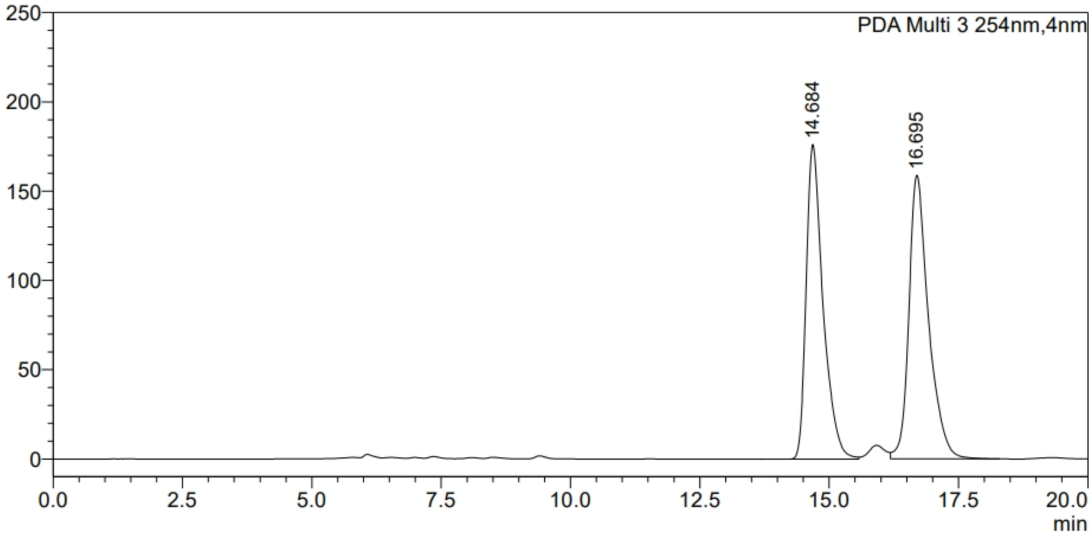

PDA Ch3 254nm

Peak#	Ret. Time	Height	Area	Area%
1	14.684	176163	3987458	48.727
2	16.695	158926	4195807	51.273
Total		335088	8183265	100.000

Chiral

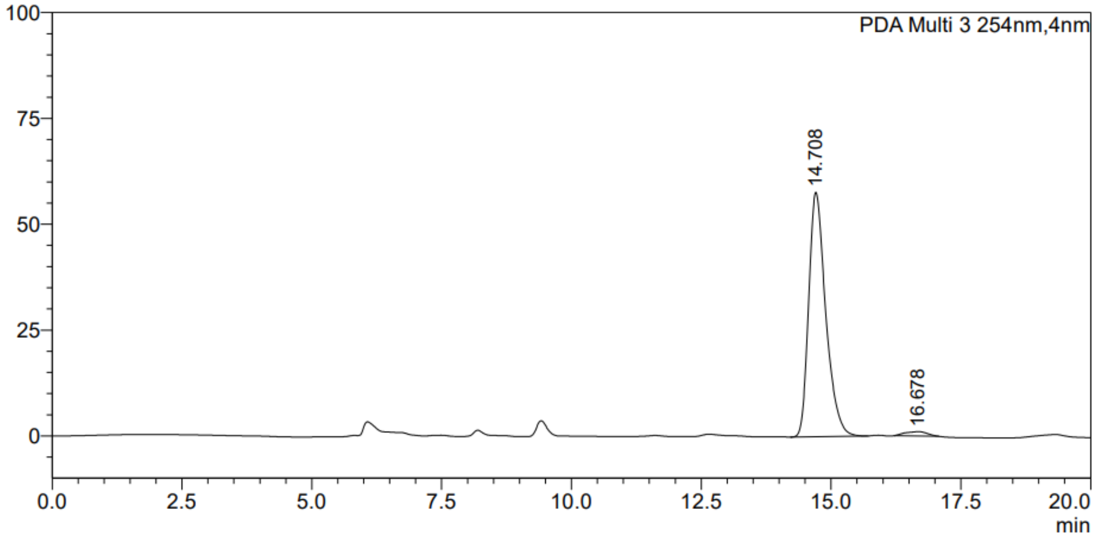

PDA Ch3 254nm

Peak#	Ret. Time	Height	Area	Area%
1	14.708	57702	1318128	97.769
2	16.678	1014	30081	2.231
Total		58716	1348209	100.000

Compound 3ad

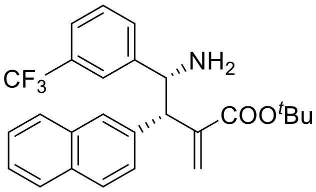

HPLC Conditions
Column: Chiralcel AD-H, Daicel Chemical Industries, Ltd.
Eluent: Hexanes/Isopropanol (94:6)
Flow rate: 0.5 mL/min
Detection: UV 254 nm

Racemic

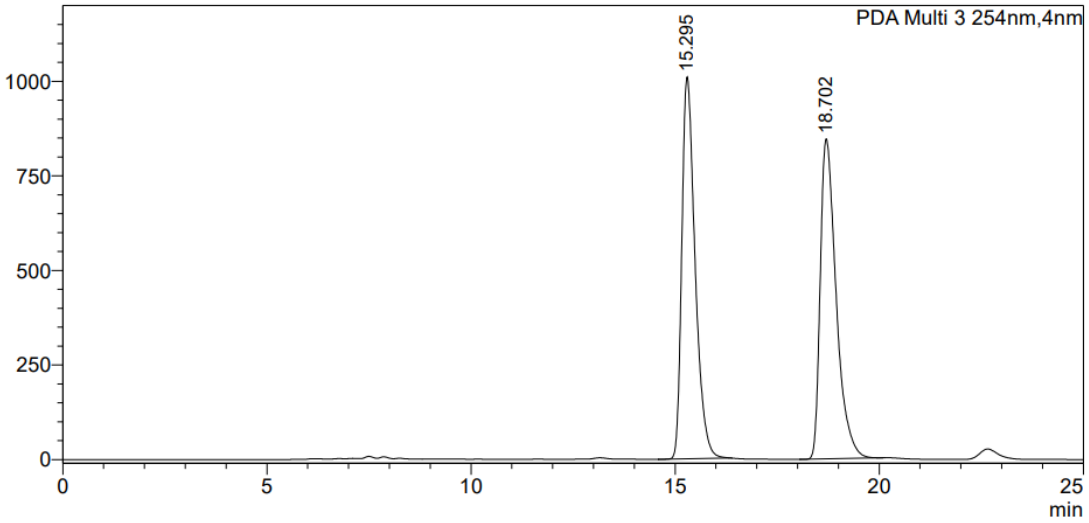

PDA Ch3 254nm

Peak#	Ret. Time	Height	Area	Area%
1	15.295	1009457	22848486	49.964
2	18.702	846133	22881061	50.036
Total		1855590	45729547	100.000

Chiral

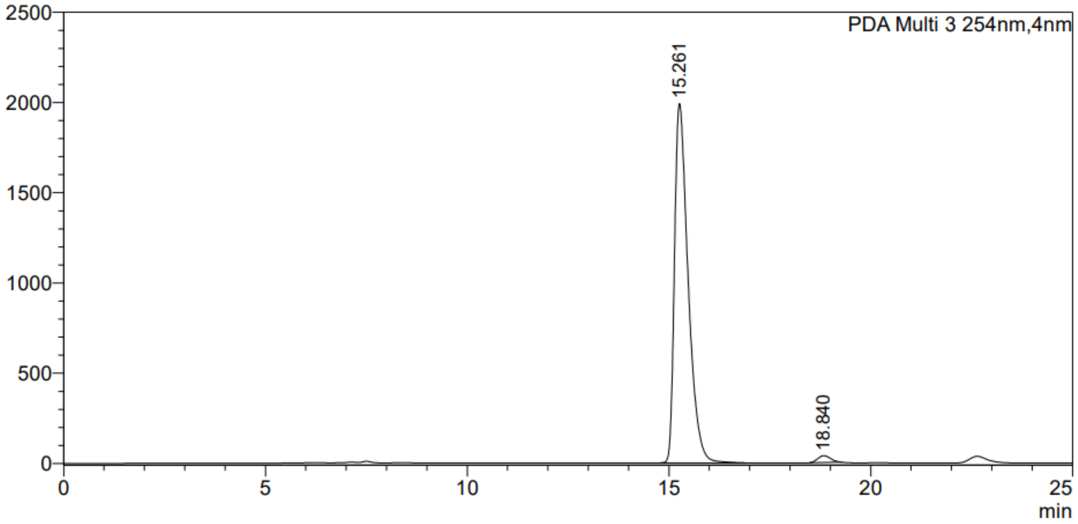

PDA Ch3 254nm

Peak#	Ret. Time	Height	Area	Area%
1	15.261	1991224	46726753	98.294
2	18.840	37309	811228	1.706
Total		2028533	47537980	100.000

Compound 3ae

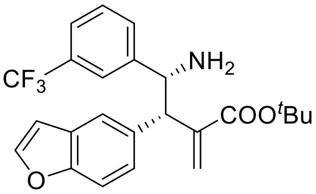

HPLC Conditions
Column: Chiralcel AD-H, Daicel Chemical Industries, Ltd.
Eluent: Hexanes/Isopropanol (94:6)
Flow rate: 0.5 mL/min
Detection: UV 254 nm

Racemic

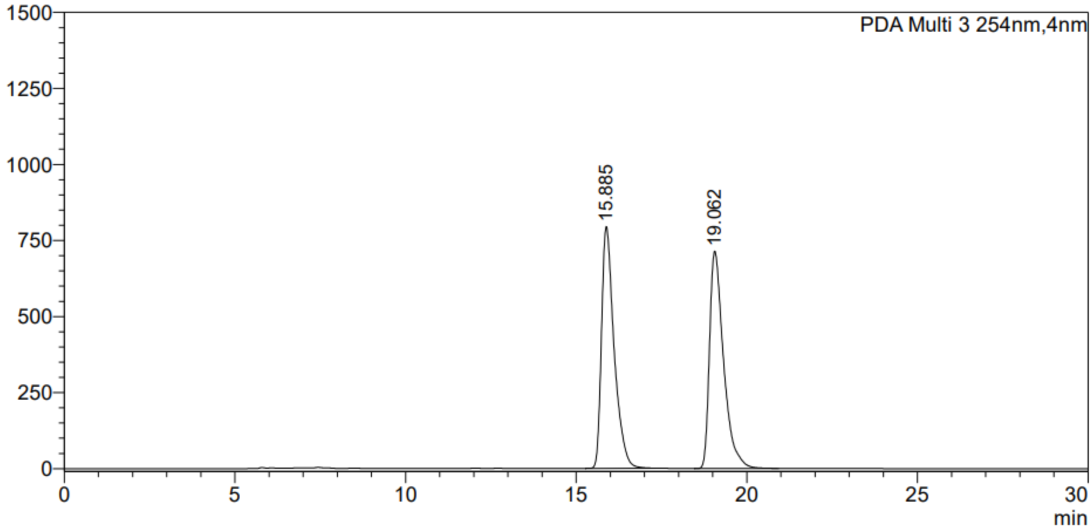

PDA Ch3 254nm

Peak#	Ret. Time	Height	Area	Area%
1	15.885	794262	20406831	49.931
2	19.062	715030	20463227	50.069
Total		1509292	40870058	100.000

Chiral

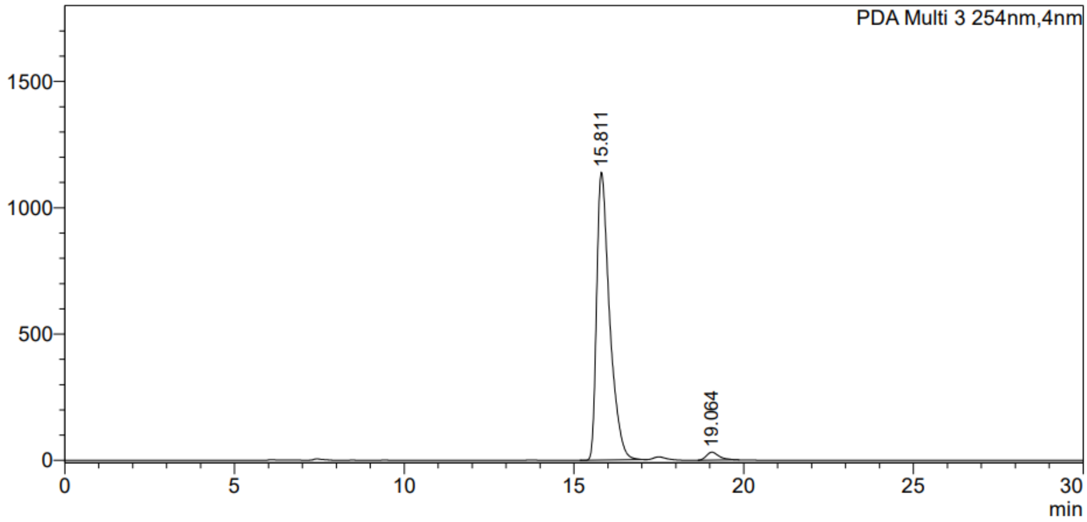

PDA Ch3 254nm

Peak#	Ret. Time	Height	Area	Area%
1	15.811	1138612	30273909	97.384
2	19.064	31392	813237	2.616
Total		1170004	31087146	100.000

Compound 3af

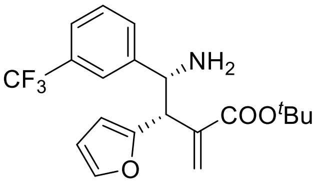

HPLC Conditions
Column: Chiralcel AD-H, Daicel Chemical Industries, Ltd.
Eluent: Hexanes/Isopropanol (94:6)
Flow rate: 0.5 mL/min
Detection: UV 230 nm

Racemic

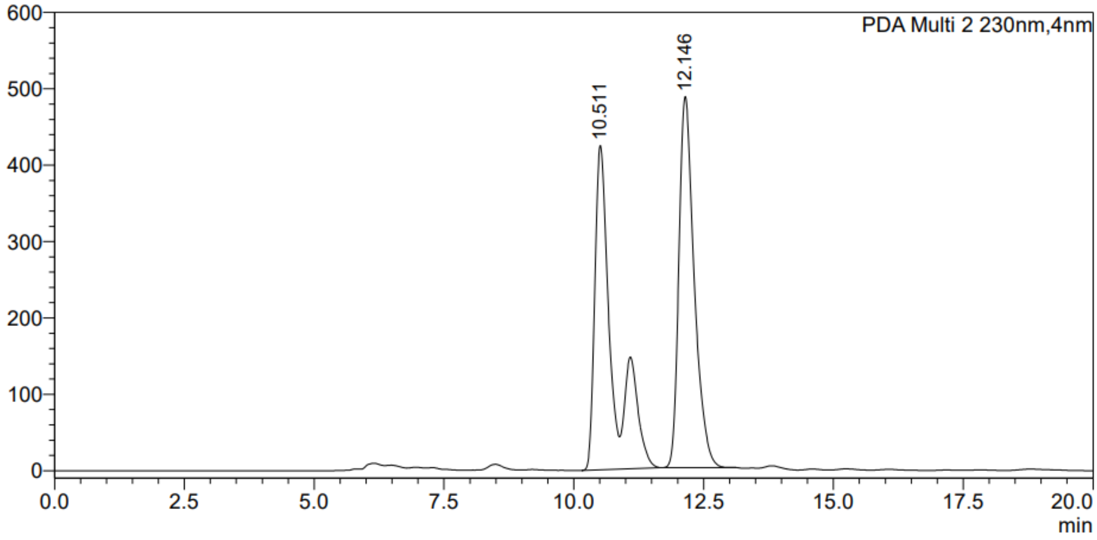

PDA Ch2 230nm

Peak#	Ret. Time	Height	Area	Area%
1	10.511	424383	10201416	50.528
2	12.146	485901	9988359	49.472
Total		910284	20189775	100.000

Chiral

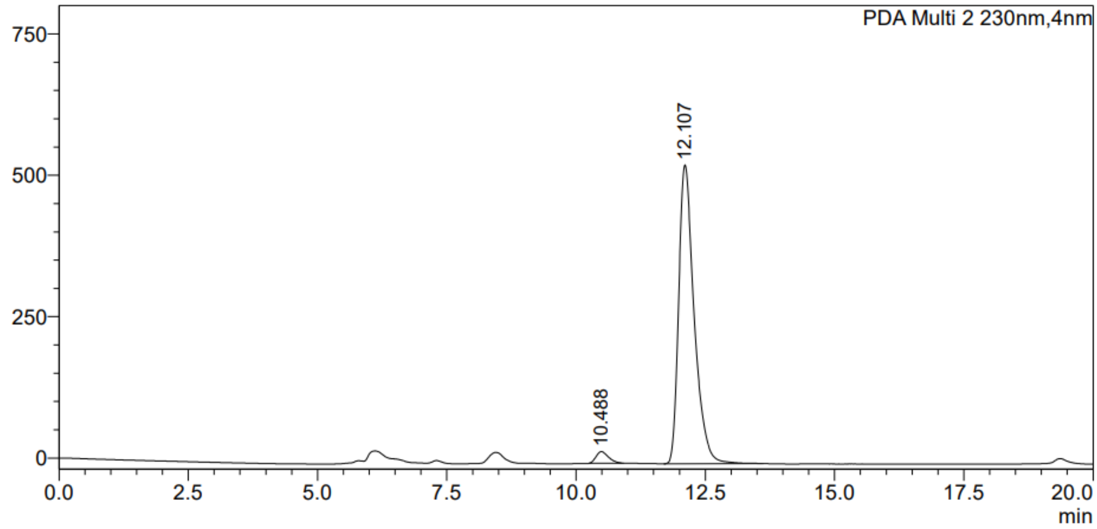

PDA Ch2 230nm

Peak#	Ret. Time	Height	Area	Area%
1	10.488	20684	341449	3.015
2	12.107	528475	10982248	96.985
Total		549159	11323697	100.000

Compound 3ag

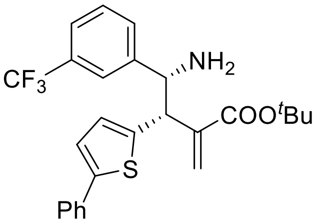

HPLC Conditions
Column: Chiralcel OD-H, Daicel Chemical Industries, Ltd.
Eluent: Hexanes/Isopropanol (94:6)
Flow rate: 0.6 mL/min
Detection: UV 254 nm

Racemic

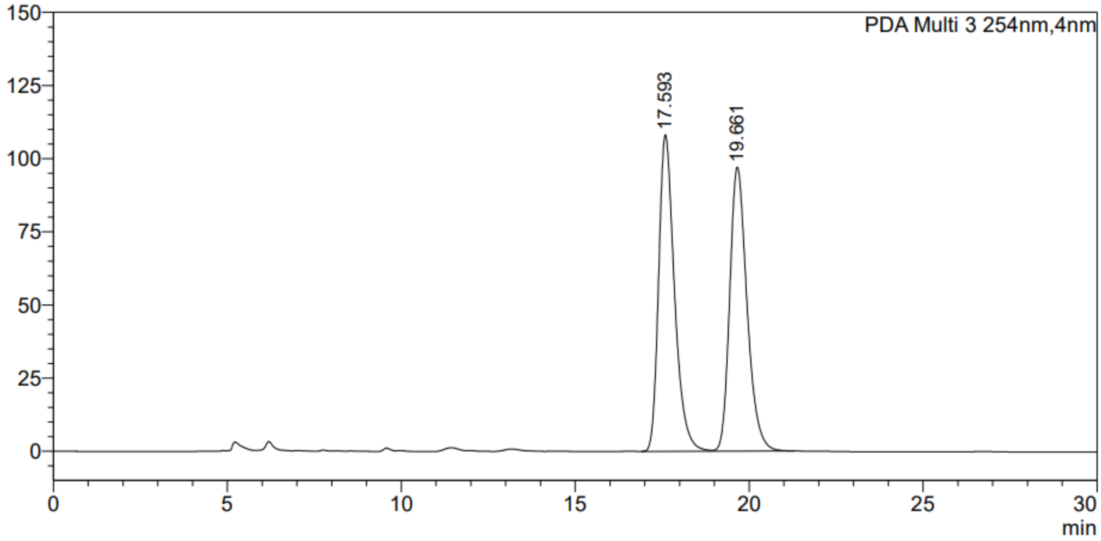

PDA Ch3 254nm

Peak#	Ret. Time	Height	Area	Area%
1	17.593	108156	3417531	50.481
2	19.661	97061	3352337	49.519
Total		205217	6769867	100.000

Chiral

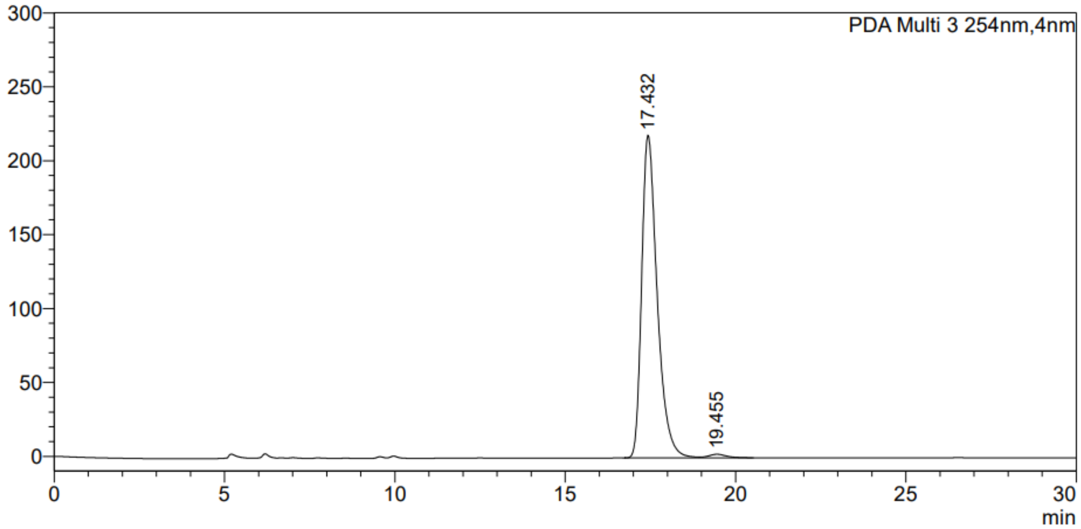

PDA Ch3 254nm

Peak#	Ret. Time	Height	Area	Area%
1	17.432	218292	6905783	98.710
2	19.455	2433	90281	1.290
Total		220725	6996065	100.000

Compound 3ah

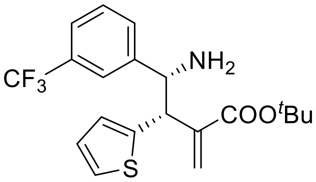

HPLC Conditions
Column: Chiralcel OD-H, Daicel Chemical Industries, Ltd.
Eluent: Hexanes/Isopropanol (94:6)
Flow rate: 0.6 mL/min
Detection: UV 254 nm

Racemic

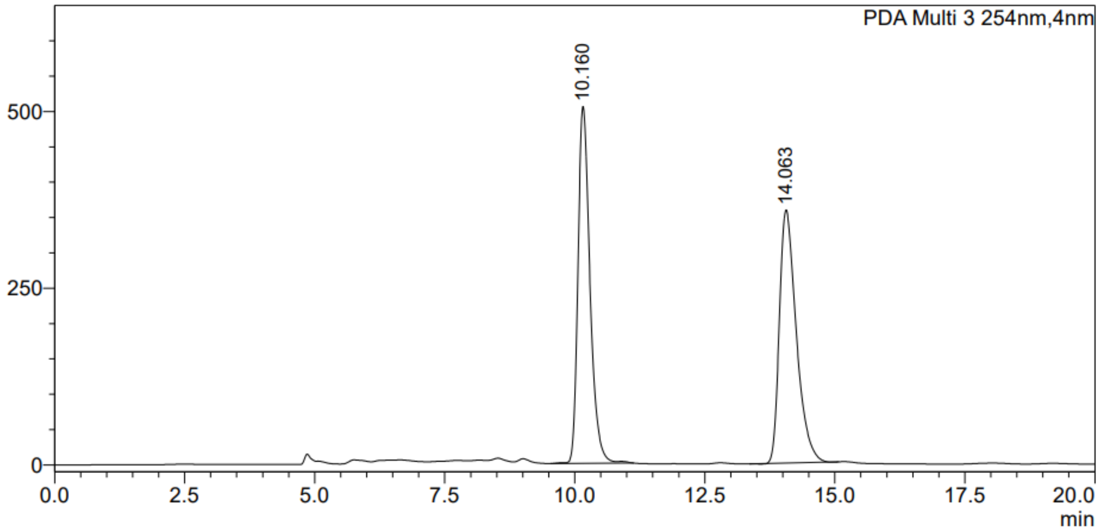

PDA Ch3 254nm

Peak#	Ret. Time	Height	Area	Area%
1	10.160	504531	8103194	50.090
2	14.063	358105	8073971	49.910
Total		862636	16177165	100.000

Chiral

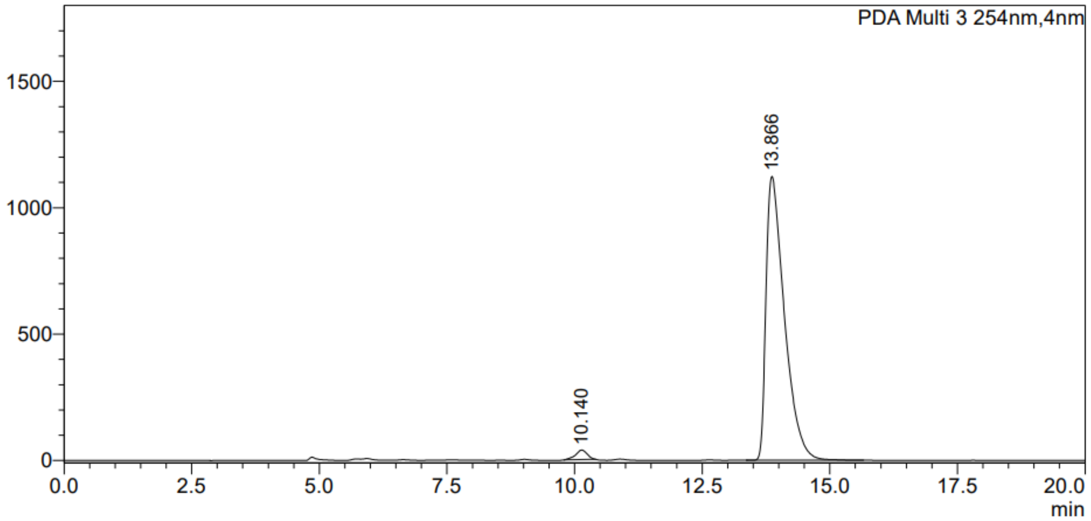

PDA Ch3 254nm

Peak#	Ret. Time	Height	Area	Area%
1	10.140	37210	590557	2.061
2	13.866	1122738	28068689	97.939
Total		1159948	28659246	100.000

Compound 3ai

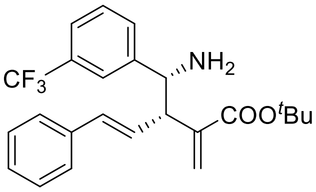

HPLC Conditions
Column: Chiralcel OD-H, Daicel Chemical Industries, Ltd.
Eluent: Hexanes/Isopropanol (94:6)
Flow rate: 0.6 mL/min
Detection: UV 254 nm

Racemic

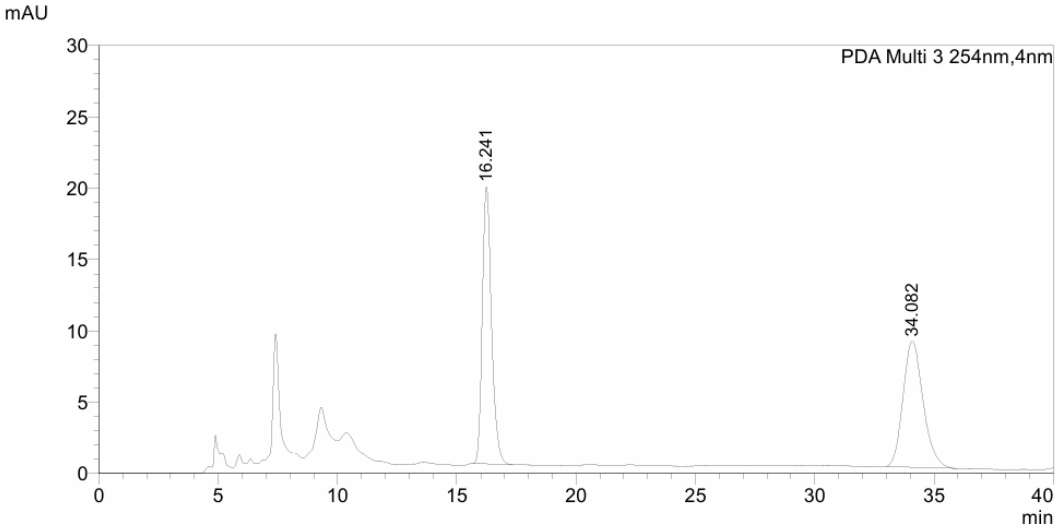

PDA Ch3 254nm

Peak#	Ret. Time	Height	Area	Area%
1	16.241	19391	520981	50.106
2	34.082	8819	518768	49.894
Total		28210	1039749	100.000

Chiral

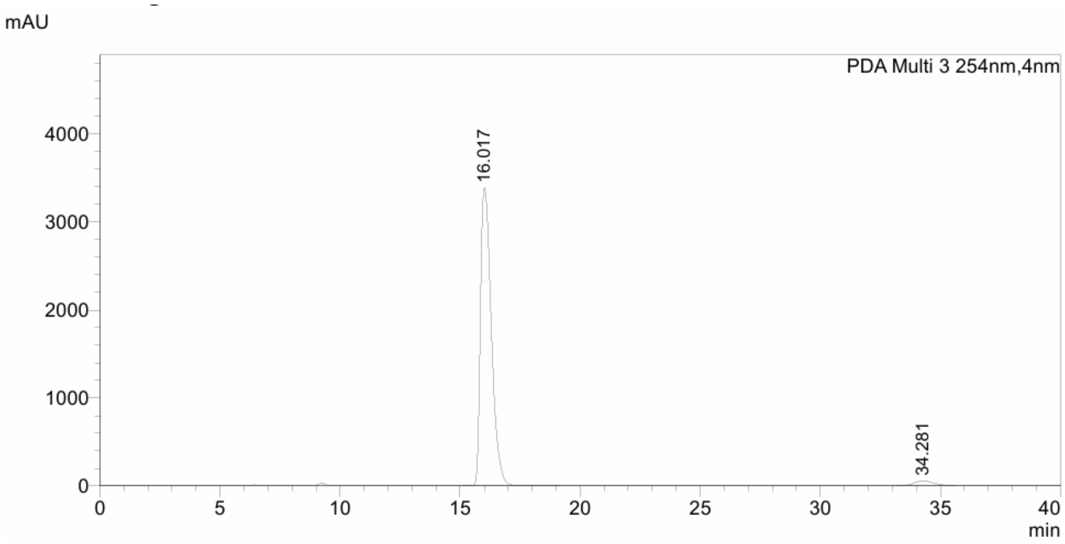

PDA Ch3 254nm

Peak#	Ret. Time	Height	Area	Area%
1	16.017	3388216	106938597	96.796
2	34.281	59629	3539306	3.204
Total		3447845	110477903	100.000

Compound 3aj

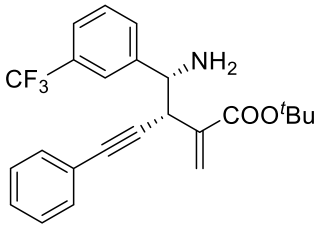

HPLC Conditions
Column: Chiralcel AD-H, Daicel Chemical Industries, Ltd.
Eluent: Hexanes/Isopropanol (94:6)
Flow rate: 0.5 mL/min
Detection: UV 254 nm

Racemic

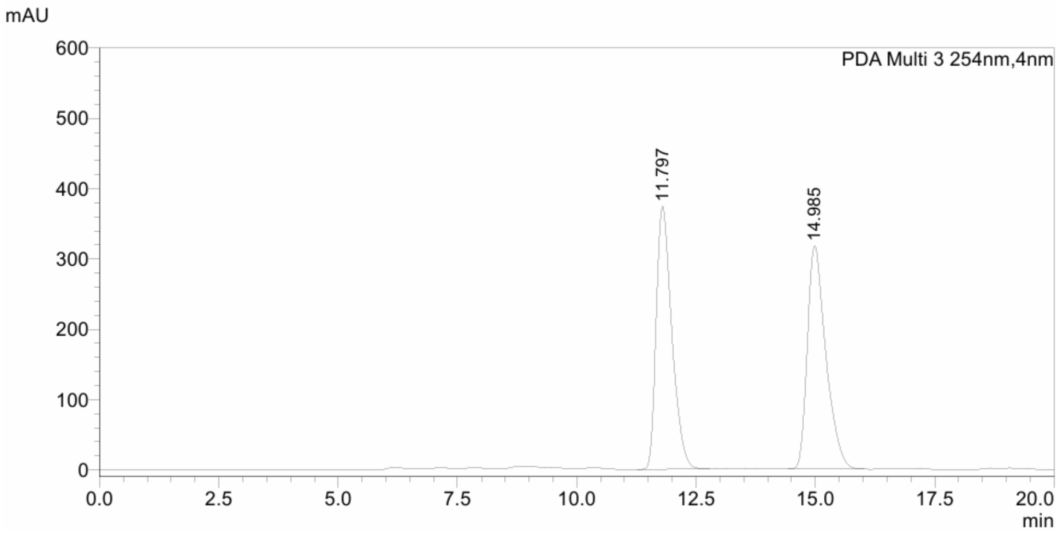

PDA Ch3 254nm

Peak#	Ret. Time	Height	Area	Area%
1	11.797	373906	8489168	49.814
2	14.985	317211	8552467	50.186
Total		691117	17041635	100.000

Chiral

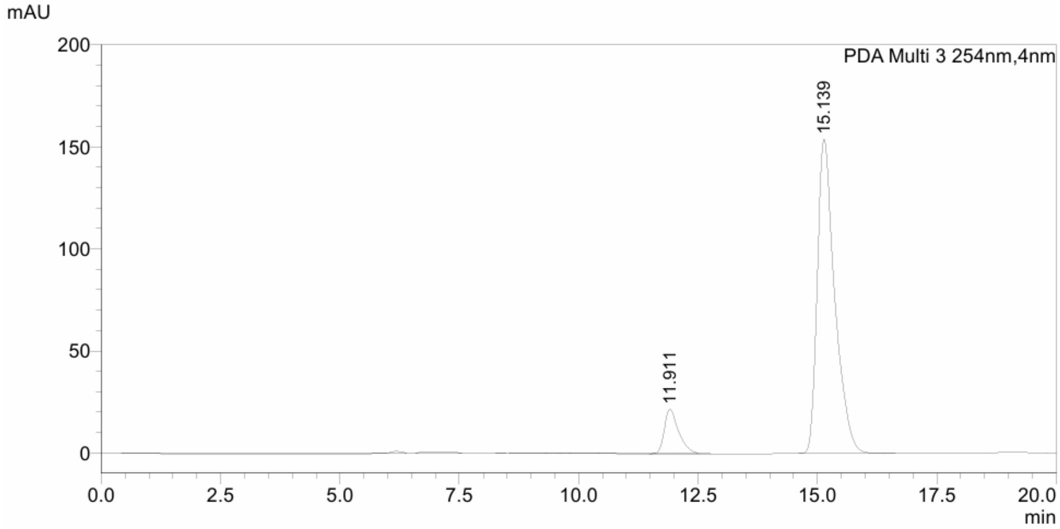

PDA Ch3 254nm

Peak#	Ret. Time	Height	Area	Area%
1	11.911	21805	452999	10.376
2	15.139	154041	3912673	89.624
Total		175846	4365672	100.000

Compound 3ak

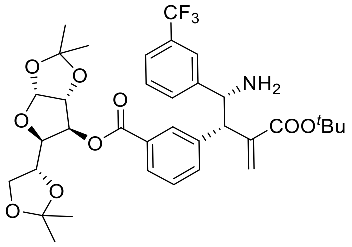

HPLC Conditions
Column: Chiralcel OD-H, Daicel Chemical Industries, Ltd.
Eluent: Hexanes/Isopropanol (94:6)
Flow rate: 0.6 mL/min
Detection: UV 230 nm

Racemic

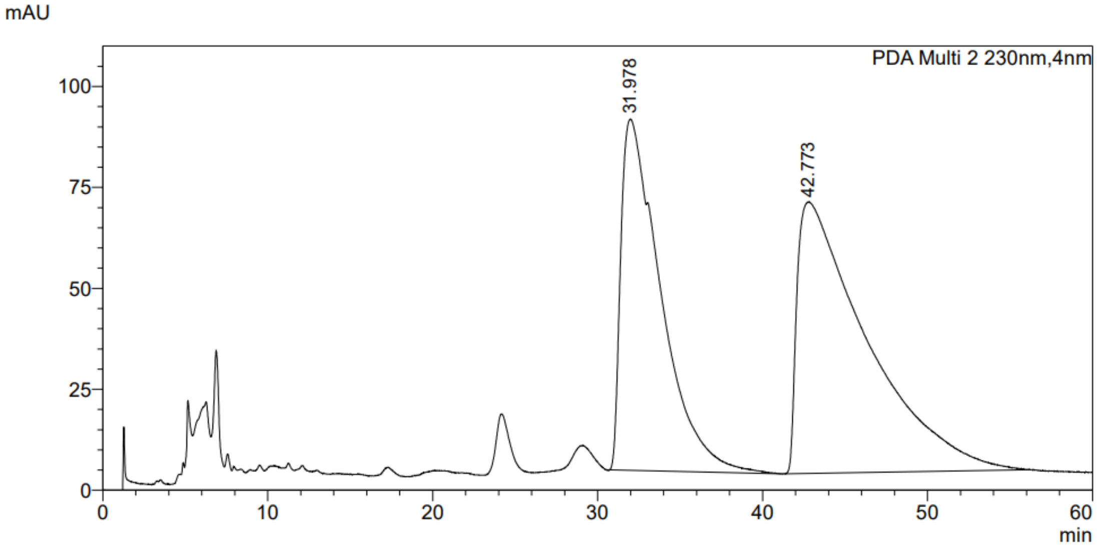

PDA Ch2 230nm

Peak#	Ret. Time	Height	Area	Area%
1	31.978	87010	14631044	42.546
2	42.773	67240	19757398	57.454
Total		154250	34388442	100.000

Chiral

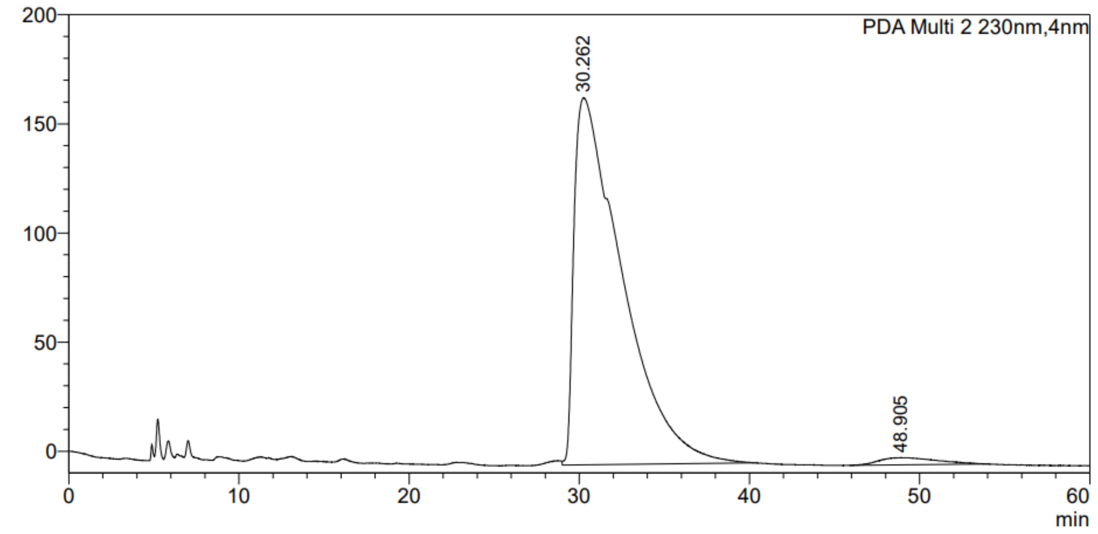

PDA Ch2 230nm

Peak#	Ret. Time	Height	Area	Area%
1	30.262	168151	33130732	97.684
2	48.905	3316	785394	2.316
Total		171468	33916125	100.000

Compound 3al

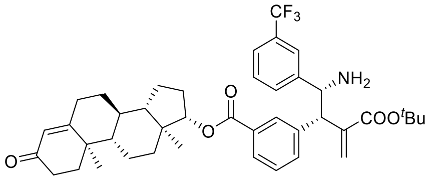

HPLC Conditions
Column: ChiralcelAD-H, Daicel Chemical Industries, Ltd.
Eluent: Hexanes/Isopropanol (90:10)
Flow rate: 1.0 mL/min
Detection: UV 230 nm

Racemic

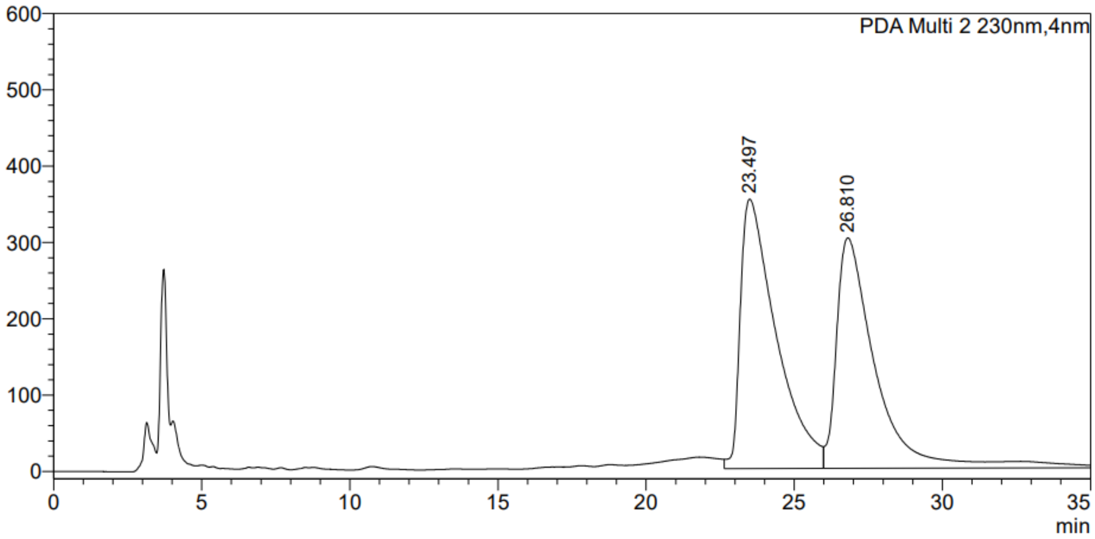

PDA Ch2 230nm

Peak#	Ret. Time	Height	Area	Area%
1	23.497	353257	29714222	50.579
2	26.810	302123	29034081	49.421
Total		655380	58748304	100.000

Chiral

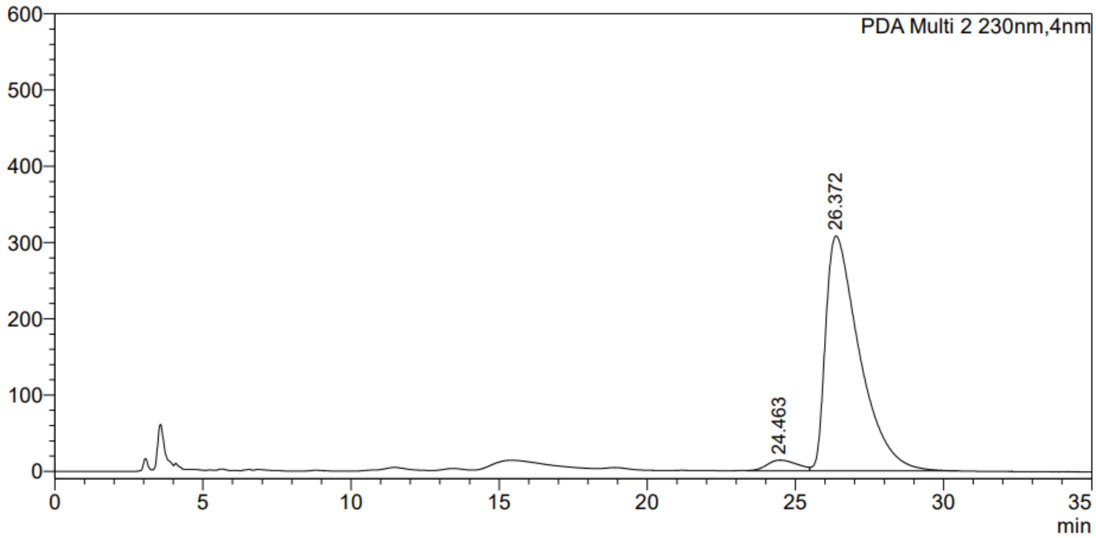

PDA Ch2 230nm

Peak#	Ret. Time	Height	Area	Area%
1	24.463	13953	991626	3.890
2	26.372	307906	24498261	96.110
Total		321859	25489887	100.000

Compound 3am

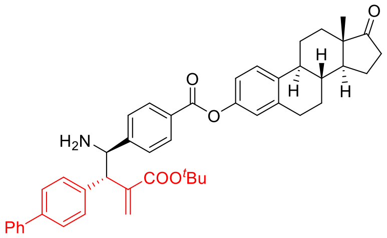

HPLC Conditions

Column: Chiralcel AD-H, Daicel Chemical Industries, Ltd.
Eluent: Hexanes/Isopropanol (85:15)
Flow rate: 1.0 mL/min
Detection: UV 254 nm

Racemic

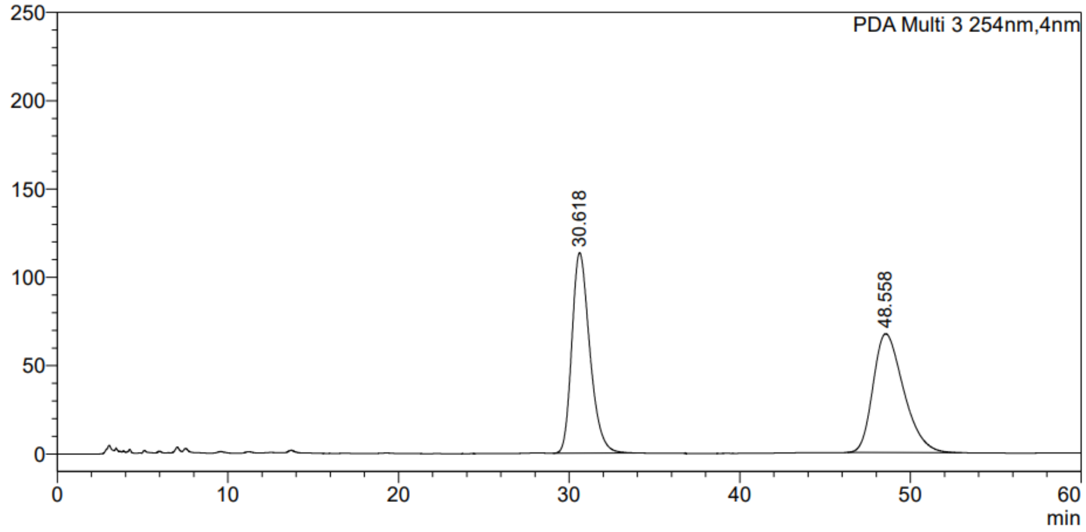

PDA Ch3 254nm

Peak#	Ret. Time	Height	Area	Area%
1	30.618	113481	8286116	49.747
2	48.558	67262	8370307	50.253
Total		180743	16656423	100.000

Chiral

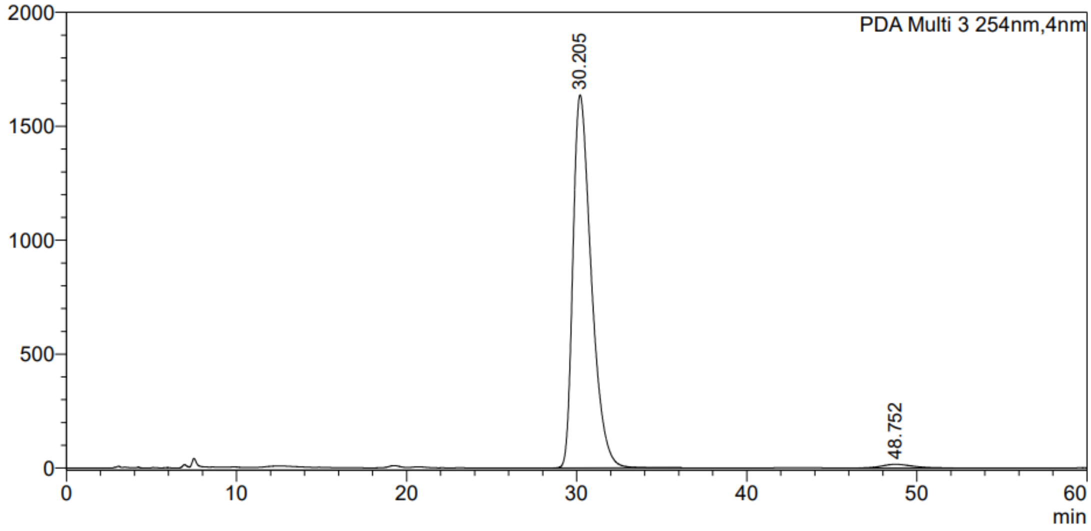

PDA Ch3 254nm

Peak#	Ret. Time	Height	Area	Area%
1	30.205	1637257	122536260	98.461
2	48.752	15777	1915619	1.539
Total		1653034	124451879	100.000

Compound 9h

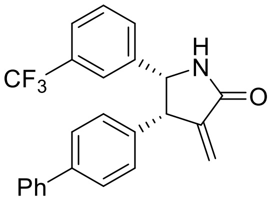

HPLC Conditions
Column: Chiralcel AD-H, Daicel Chemical Industries, Ltd.
Eluent: Hexanes/Isopropanol (94:6)
Flow rate: 0.5 mL/min
Detection: UV 254 nm

Racemic

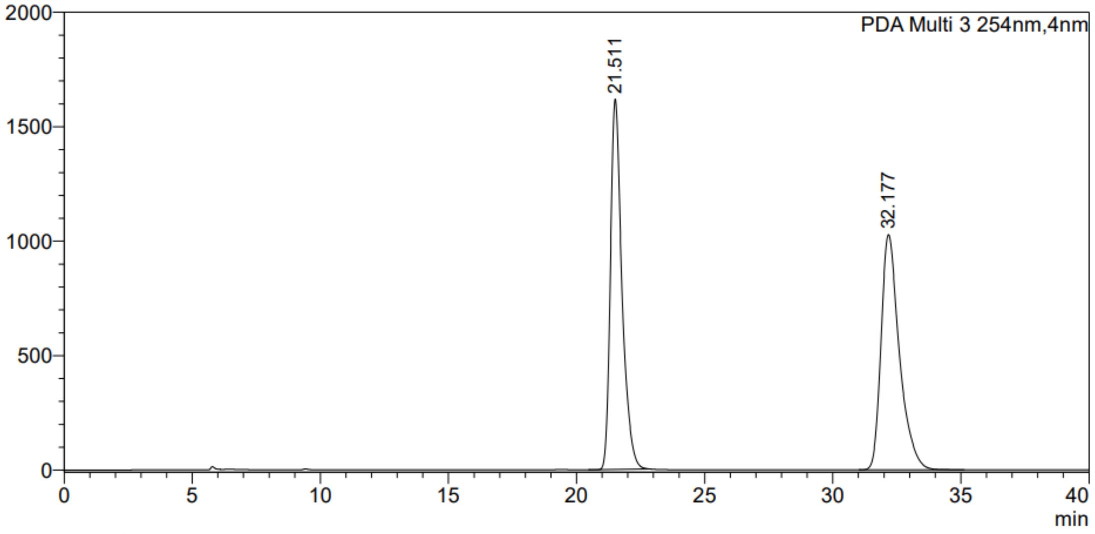

PDA Ch3 254nm

Peak#	Ret. Time	Height	Area	Area%
1	21.511	1617748	50515559	49.777
2	32.177	1026936	50968276	50.223
Total		2644684	101483835	100.000

Chiral

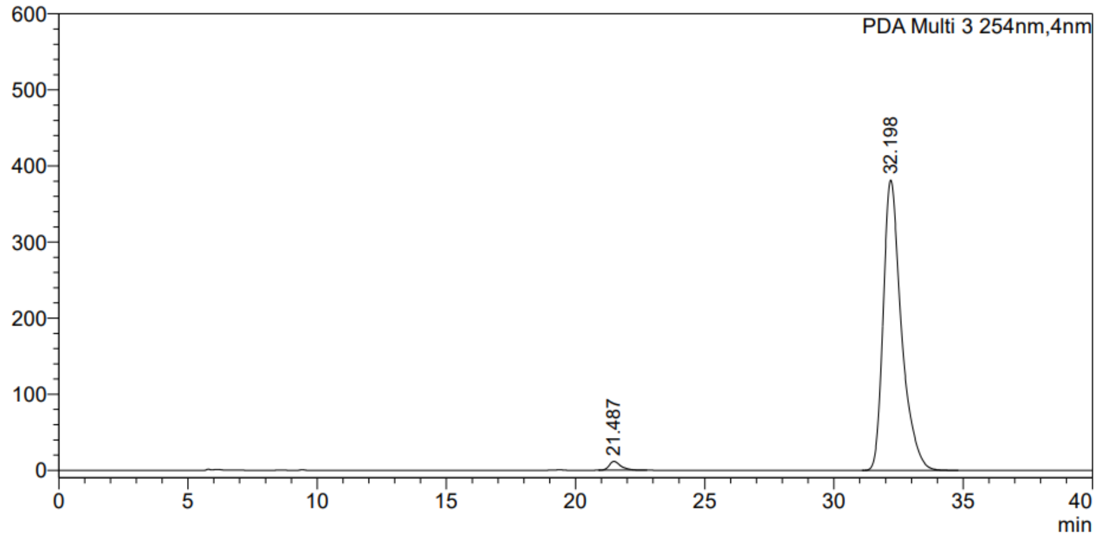

PDA Ch3 254nm

Peak#	Ret. Time	Height	Area	Area%
1	21.487	11603	366193	1.924
2	32.198	381730	18669831	98.076
Total		393333	19036024	100.000

Compound 9a

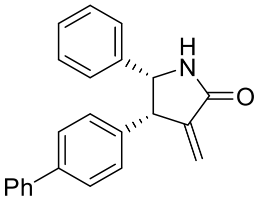

HPLC Conditions
Column: Chiralcel AD-H, Daicel Chemical Industries, Ltd.
Eluent: Hexanes/Isopropanol (94:6)
Flow rate: 1.0 mL/min
Detection: UV 254 nm

Racemic

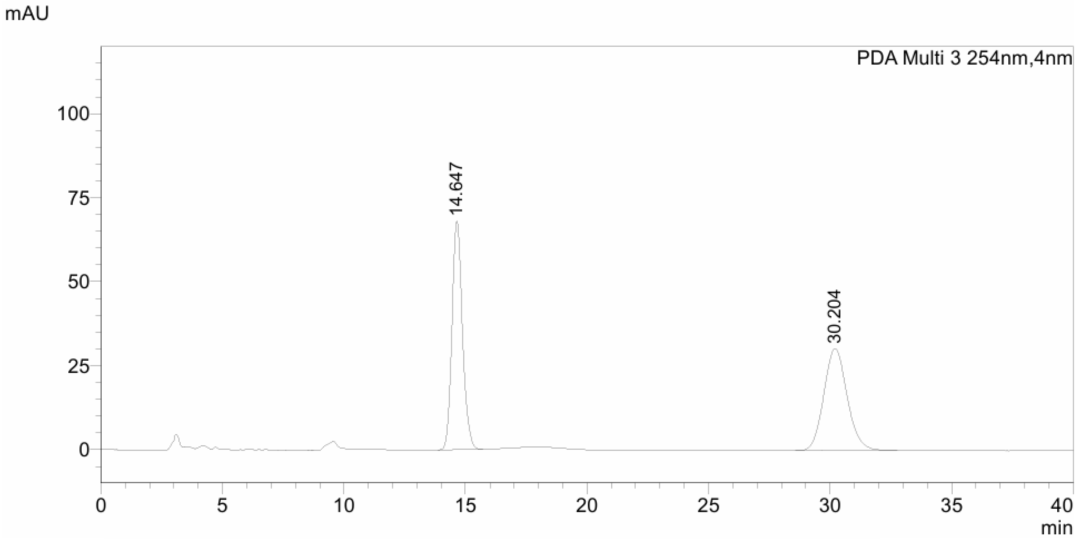

PDA Ch3 254nm

Peak#	Ret. Time	Height	Area	Area%
1	14.647	68071	2042301	50.216
2	30.204	30335	2024734	49.784
Total		98406	4067034	100.000

Chiral

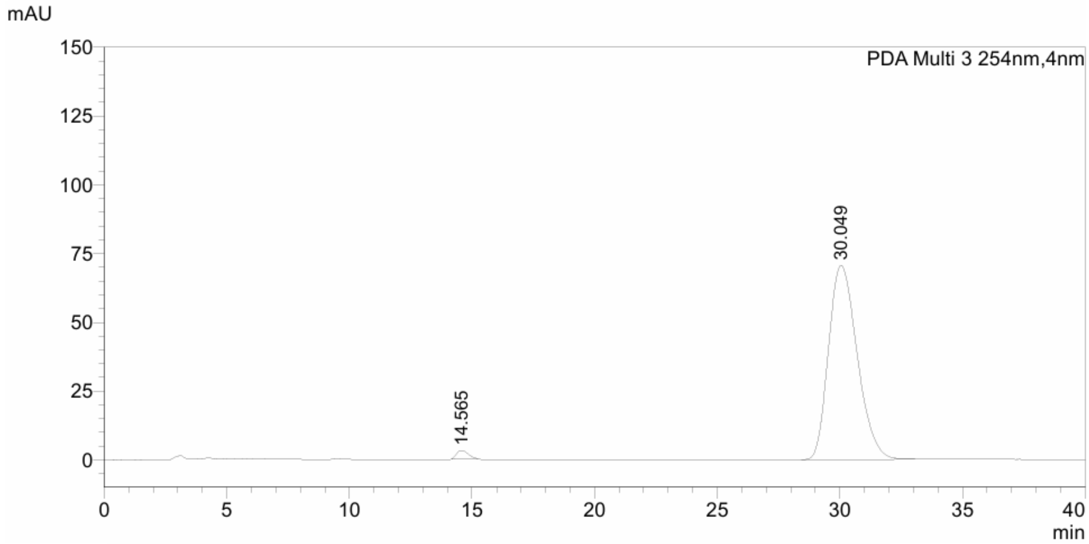

PDA Ch3 254nm

Peak#	Ret. Time	Height	Area	Area%
1	14.565	3046	99116	1.698
2	30.049	70575	5736553	98.302
Total		73621	5835670	100.000

Compound 10h'

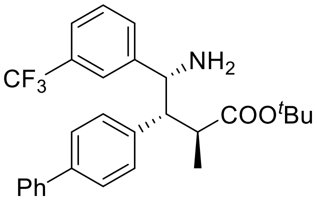

HPLC Conditions
Column: Chiralcel AD-H, Daicel Chemical Industries, Ltd.
Eluent: Hexanes/Isopropanol (94:6)
Flow rate: 0.5 mL/min
Detection: UV 254 nm

Racemic

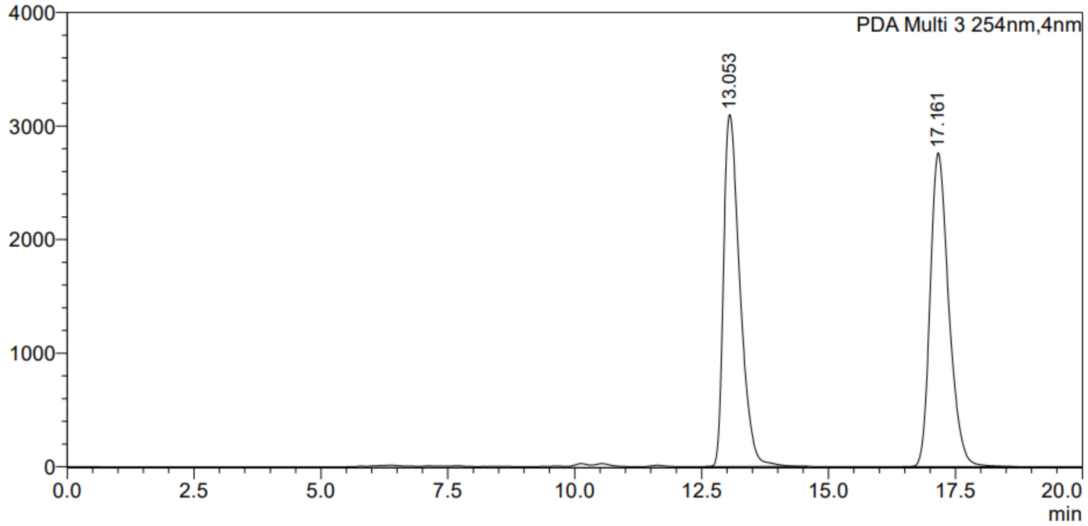

PDA Ch3 254nm

Peak#	Ret. Time	Height	Area	Area%
1	13.053	3097309	69475301	49.689
2	17.161	2763071	70345690	50.311
Total		5860380	139820990	100.000

Chiral

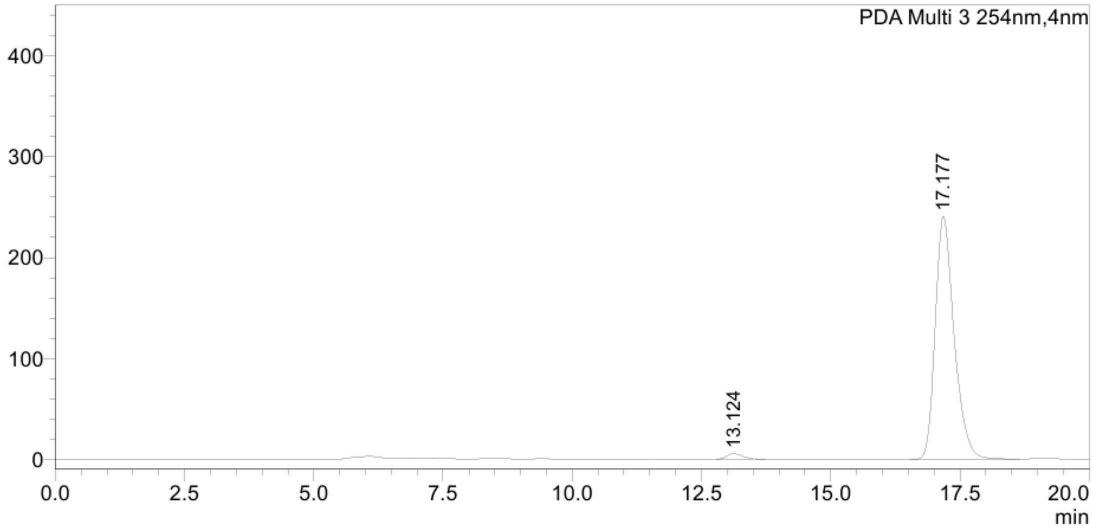

PDA Ch3 254nm

Peak#	Ret. Time	Height	Area	Area%
1	13.124	5408	111005	1.780
2	17.177	240642	6124091	98.220
Total		246049	6235096	100.000

Compound 10h

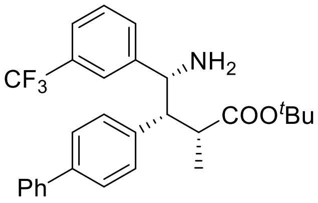

HPLC Conditions
Column: Chiralcel AD-H, Daicel Chemical Industries, Ltd.
Eluent: Hexanes/Isopropanol (98:2)
Flow rate: 0.2 mL/min
Detection: UV 254 nm

Racemic

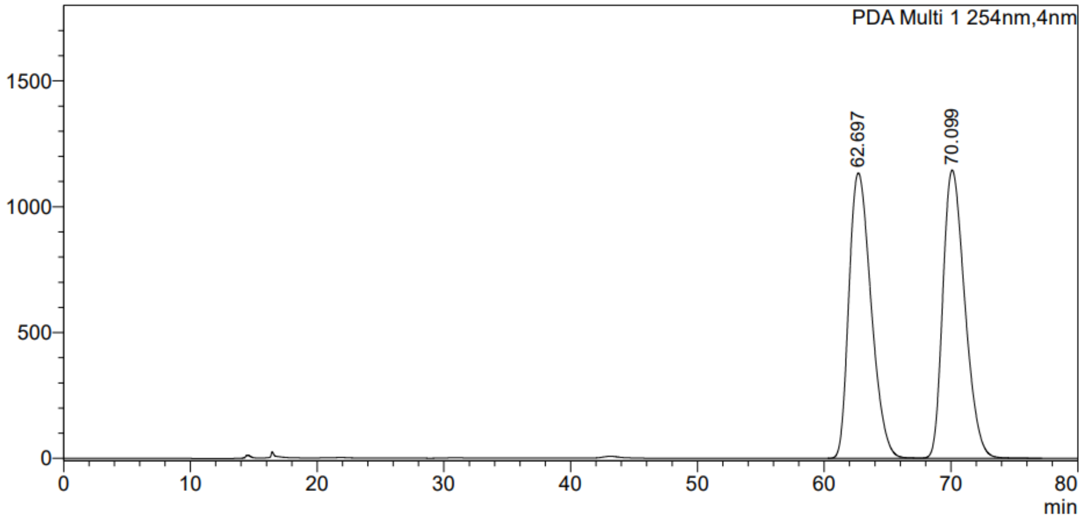

PDA Ch1 254nm

Peak#	Ret. Time	Height	Area	Area%
1	62.697	1133731	137547081	49.936
2	70.099	1145526	137897572	50.064
Total		2279258	275444652	100.000

Chiral

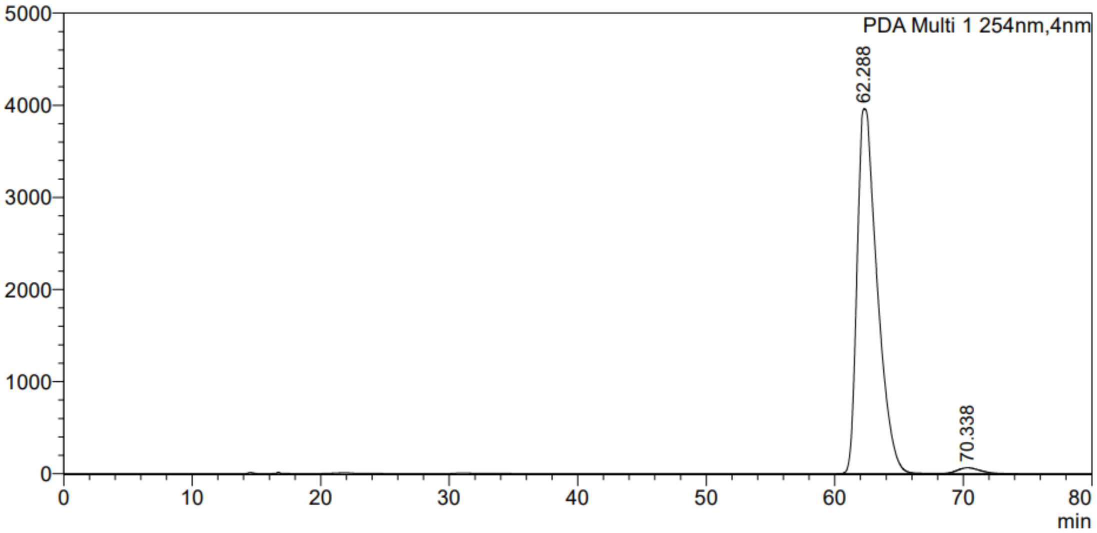

PDA Ch1 254nm

Peak#	Ret. Time	Height	Area	Area%
1	62.288	3961129	429838758	98.163
2	70.338	63797	8043194	1.837
Total		4024926	437881952	100.000

Compound 11h'

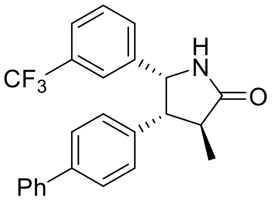

HPLC Conditions
Column: Chiralcel OD-H, Daicel Chemical Industries, Ltd.
Eluent: Hexanes/Isopropanol (94:6)
Flow rate: 0.6 mL/min
Detection: UV 254 nm

Racemic

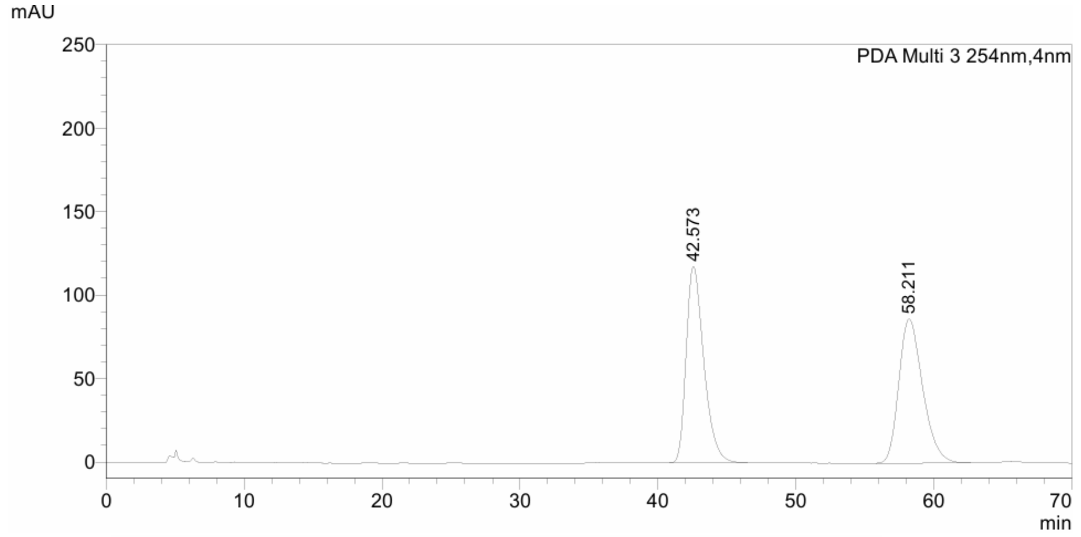

PDA Ch3 254nm

Peak#	Ret. Time	Height	Area	Area%
1	42.573	117615	10494860	50.216
2	58.211	86348	10404713	49.784
Total		203964	20899573	100.000

Chiral

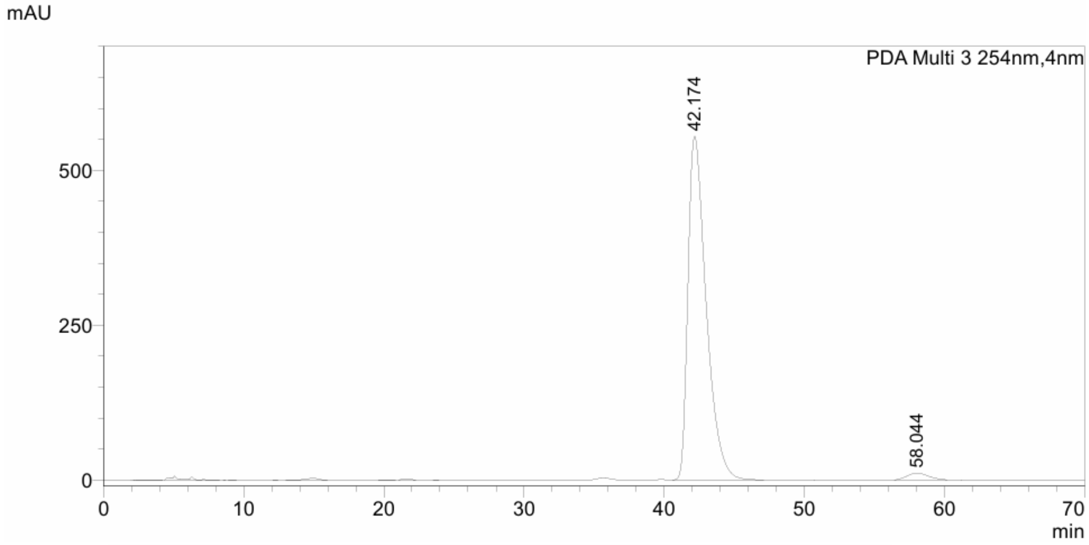

PDA Ch3 254nm

Peak#	Ret. Time	Height	Area	Area%
1	42.174	554568	50072188	97.775
2	58.044	10815	1139506	2.225
Total		565383	51211695	100.000

14. References

1. Ma, J. G.; Gao, B.; Song, G. S.; Zhang, R. X.; Wang, Q. F.; Ye, Z.; Chen, W. W.; Zhao, B. G., Asymmetric α-Allylation of Glycinate with Switched Chemoselectivity Enabled by Customized Bifunctional Pyridoxal Catalysts. *Angew. Chem. Int. Ed.* **2022**, *61,* e202200850.

2. Mendoza-Espinosa, D.; González-Olvera, R.; Osornio, C.; Negrón-Silva, G. E.; Santillan, R., Versatile O- and S-functionalized 1,2,3-Triazoliums: Ionic Liquids for the Baylis-Hillman Reaction and Ligand Precursors for Stable MIC-transition Metal Complexes. *New J. Chem.* **2015**, *39*, 1587-1591.

3. Furukawa, T.; Nishimine, T.; Tokunaga, E.; Hasegawa, K.; Shiro, M.; Shibata, N., Organocatalyzed Regio- and Enantioselective Allylic Trifluoromethylation of Morita–Baylis–Hillman Adducts Using Ruppert–Prakash Reagent. *Org. Lett.* **2011**, *13*, 3972-3975.

4. Basavaiah, D.; Krishnamacharyulu, M.; Hyma, R. S.; Sarma, P. K. S.; Kumaragurubaran, N., A Facile One-pot Conversion of Acetates of the Baylis-Hillman Adducts to *E*-α-Methylcinnamic Acids. *J. Org. Chem.* **1999**, *64*, 1197-1200.

5. Badkar, P. A.; Rath, N. P.; Spilling, C. D., Asymmetric Synthesis of 2-Alkyl-3-Phosphonopropanoic Acids via P−C Bond Formation and Hydrogenation. *Org. Lett.* **2007**, *9*, 3619-3622.

6. Park, S. P.; Ahn, S.-H.; Lee, K.-J., 3,5-Disubstituted 6H-Pyrrolo[1,2-c][1,2,3]triazoles from Morita–Baylis–Hillman Adducts of Propargyl Aldehydes. *Tetrahedron* **2010**, *66*, 3490-3498.

7. Liu, X. Y.; Li, X. Y.; Yang, F. L.; Li, Y.; Jiao, X. Z.; Xie, P., Design, Synthesis of a Novel 4-O-methylsaucerneol Analogue LXY7824 as Potent HIF-1 Inhibitor and Anti-cancer Agent. *J. Asian Nat. Prod. Res.* **2018**, *20*, 545-558.

8. Yang, C.; Xue, X.-S.; Jin, J.-L.; Li, X.; Cheng, J.-P., Theoretical Study on the Acidities of Chiral Phosphoric Acids in Dimethyl Sulfoxide: Hints for Organocatalysis. *J. Org. Chem.* **2013**, *78*, 7076-7085.

9. Frisch, M. J.; Trucks, G. W.; Schlegel, H. B.; Scuseria, G. E.; Robb, M. A.; Cheeseman, J. R.; Scalmani, G.; Barone, V.; Petersson, G. A.; Nakatsuji, H.; Li, X.; Caricato, M.; Marenich, A. V.; Bloino, J.; Janesko, B. G.; Gomperts, R.; Mennucci, B.; Hratchian, H. P.; Ortiz, J. V.; Izmaylov, A. F.; Sonnenberg, J. L.; Williams; Ding, F.; Lipparini, F.; Egidi, F.; Goings, J.; Peng, B.; Petrone, A.; Henderson, T.;

Ranasinghe, D.; Zakrzewski, V. G.; Gao, J.; Rega, N.; Zheng, G.; Liang, W.; Hada, M.; Ehara, M.; Toyota, K.; Fukuda, R.; Hasegawa, J.; Ishida, M.; Nakajima, T.; Honda, Y.; Kitao, O.; Nakai, H.; Vreven, T.; Throssell, K.; Montgomery Jr., J. A.; Peralta, J. E.; Ogliaro, F.; Bearpark, M. J.; Heyd, J. J.; Brothers, E. N.; Kudin, K. N.; Staroverov, V. N.; Keith, T. A.; Kobayashi, R.; Normand, J.; Raghavachari, K.; Rendell, A. P.; Burant, J. C.; Iyengar, S. S.; Tomasi, J.; Cossi, M.; Millam, J. M.; Klene, M.; Adamo, C.; Cammi, R.; Ochterski, J. W.; Martin, R. L.; Morokuma, K.; Farkas, O.; Foresman, J. B.; Fox, D. J. *Gaussian 16 Rev. C.01*, Wallingford, CT, 2016.

10. Stephens, P. J.; Devlin, F. J.; Chabalowski, C. F.; Frisch, M. J., Ab Initio Calculation of Vibrational Absorption and Circular Dichroism Spectra Using Density Functional Force Fields. *J. Phys. Chem.* **1994**, *98*, 11623-11627.

11. Becke, A. D., Density-functional thermochemistry. III. The Role of Exact Exchange. *J. Chem. Phys.* **1993**, *98*, 5648-5652.

12. Lee, C.; Yang, W.; Parr, R. G., Development of the Colle-Salvetti Correlation-energy Formula into a Functional of the Electron Density. *Phys. Rev. B* **1988,** *37*, 785-789.

13. Becke, A. D., A New Mixing of Hartree–Fock and Local Density-functional Theories. *J Chem. Phys.* **1993**, *98*, 1372-1377.

14. Grimme, S.; Ehrlich, S.; Goerigk, L., Effect of the Damping Function in Dispersion Corrected Density Functional Theory. *J. Comput. Chem.* **2011**, *32*, 1456-1465.

15. Weigend, F.; Ahlrichs, R., Balanced Basis Sets of Split Valence, Triple Zeta Valence and Quadruple Zeta Valence Quality for H to Rn: Design and Assessment of Accuracy. *Phys. Chem. Chem. Phys.* **2005**, *7*, 3297-3305.

16. Tomasi, J.; Mennucci, B.; Cammi, R., Quantum Mechanical Continuum Solvation Models. *Chem. Rev.* **2005**, *105*, 2999-3094.

17. Ditchfield, R.; Hehre, W. J.; Pople, J. A., Self-Consistent Molecular-Orbital Methods. IX. An Extended Gaussian-Type Basis for Molecular-Orbital Studies of Organic Molecules. *J. Chem. Phys.* **1971**, *54*, 724-728.

18. Hariharan, P. C.; Pople, J. A., The Influence of Polarization Functions on Molecular Orbital Hydrogenation Energies. *Theor. Chem. Acc.* **1973**, *28*, 213-222.

19. Hehre, W. J.; Ditchfield, R.; Pople, J. A., Self—consistent Molecular Orbital Methods. XII. Further Extensions of Gaussian—type Basis Sets for Use in Molecular Orbital Studies of Organic Molecules. *J. Chem. Phys.* **1972**, *56*, 2257-2261.

20. Te Velde, G.; Bickelhaupt, F. M.; Baerends, E. J.; Fonseca Guerra, C.; Van Gisbergen, S. J. A.; Snijders, J. G.; Ziegler, T., Chemistry with ADF. *J. Comput. Chem.* **2001**, *22*, 931-967.
21. Fonseca Guerra, C.; Snijders, J. G.; te Velde, G.; Baerends, E. J., Towards an Order-N DFT method. *Theor. Chem. Acc.* **1998**, *99*, 391-403.
22. Van Lenthe, E.; Baerends, E. J., Optimized Slater-type Basis Sets for the Elements 1–118. *J. Comput. Chem.* **2003**, *24*, 1142-1156.
23. Lu, T., A Comprehensive Electron Wavefunction Analysis Toolbox for Chemists, Multiwfn. *J. Chem. Phys.* **2024**, *161*, 082503.
24. Humphrey, W.; Dalke, A.; Schulten, K., VMD: Visual Molecular Dynamics, *J. Mol. Graph.* **1996**, *14*, 33-38.
25. Chai, J.-D.; Head-Gordon, M., Long-range Corrected Hybrid Density Functionals with Damped Atom–atom Dispersion Corrections. *Phys. Chem. Chem. Phys.* **2008**, *10*, 6615-6620.
26. Krishnan, R.; Binkley, J. S.; Seeger, R.; Pople, J. A., Self-consistent Molecular Orbital Methods. XX. A Basis Set for Correlated Wave Functions. *J. Chem. Phys.* **1980**, *72*, 650-654.
27. Lu, T.; Chen, Q., Shermo: A General Code for Calculating Molecular Thermochemistry Properties. *Comput. Theor. Chem.* **2021**, *1200*, 113249.
