# Supplementary material for: Direct asymmetric α C(sp3)‒H alkylation of benzylamines with MBH acetates enabled by bifunctional pyridoxal catalysts
Source: Nat Commun. 2025 Nov 27;16:10645. doi: 10.1038/s41467-025-65648-5 (PMC12660838; doi:10.1038/s41467-025-65648-5)
Supplement: Supplementary file 2 — Description of Additional Supplementary Files [file 41467_2025_65648_MOESM2_ESM.pdf]

Description of Additional Supplementary Files

File Name: Supplementary Data 1

Description: Coordinates of the optimized structures for the computational studies.
